# Supplementary material for: Plasmodium falciparum surf4.1 in clinical isolates: From genetic variation and variant diversity to in silico design immunopeptides for vaccine development
Source: PLoS One. 2024 Dec 30;19(12):e0312091. doi: 10.1371/journal.pone.0312091 (PMC11684625; doi:10.1371/journal.pone.0312091)
Supplement: S1 Table — (PDF) [file pone.0312091.s001.pdf]

S1 Table. Deposited *Pfsurf$_{4.1}$* in GenBank and PlasmoDB database.

GenBank/PlasmoDB	References strains	Country of origin
402200.1, AB759917.1	3D7 CDS, 3D7A	Netherlands
40006900	KH01	Cambodia
40007200	Dd2	Indochina
40006800	GA01	Gabon
40006900	SN01	Senegal
40007200	GN01	Guinea
40008500	KE01	Kenya
40006700	KH02	Cambodia
40007300	GB4	Ghana
40006900	CD01	Congo
40007100, AB516457.1	7G8	Brazil
40006000	HB3	Honduras
40006600	IT	Brazil
70017400	SD01	Sudan
AB759921.1, AB759919.1	MS822 Gene, MS822 mRNA	Thailand
AB759920.1, AB759918.1	FCR3 Gene, FCR3 mRNA	Gambia
AB516467.1	D10	Papua New Guinea
KU358974.1 - KU358951.1	Gitaka,J.N.Malar. J. 16 (1), 98 (2017)	Kenya
AB480068.1 - AB480049.1	Xangsayarath,P. Trop Med Health 40 (3), 79-89 (2012)	Thailand
AB712335.1 -AB712293.1	Xangsayarath,P.,Trop Med Health 40 (3), 79-89 (2012)	Thailand
HM000393.1-HM000443.1	Ochola,L.I.,Mol. Biol. Evol. 27 (10), 2344-2351 (2010)	Kenya
