# Supplementary material for: Plasmodium falciparum surf4.1 in clinical isolates: From genetic variation and variant diversity to in silico design immunopeptides for vaccine development
Source: PLoS One. 2024 Dec 30;19(12):e0312091. doi: 10.1371/journal.pone.0312091 (PMC11684625; doi:10.1371/journal.pone.0312091)
Supplement: S2 Table — (PDF) [file pone.0312091.s002.pdf]

S2 Table. Alanine insertion causing different $SURFIN_{4.1}$ variants.

Type of $SURFIN_{4.1}$	Genotype (Nucleotide position)						This study	Deposited sequences
	Alanine Insertion				Alanine Insertion	Alanine Insertion		
	2503/2504	2531	2540	2584	3903	4536/4537		
TM1<br>(NO WRD)	No	Stop (TGA)	Stop (TAA)	Stop (TAA)	Yes/No	No	AM1737, AA264, SC03, SC52, SC53, TAB123, TAB130, TAB131, TAB138, TAB151, TAB152	3D7, KH01, Dd2, GA01, SN01, GN01, KE01, KH02, GB4, MS822
TM2<br>(NO WRD, mutation)<br>* This study	No	R (AGA)	Stop (TAA)	Stop (TAA)	Yes/No	No	AM1898, AA234, AA243, AA258, SC55, SC74, TAB141, TAB166	-
WD1	Yes	Frameshift E (GAG)	Read through	S (TCA)	Yes	No	TAB136	CD01
WD2	Yes	Frameshift E (GAG)	Read through	S (TCA)	No	Yes		FCR3
WD3	Yes	frameshift E (GAG)	Read through	S (TCA)	No	No	AM1802, AM1803, AM1811, AM1814, AM1880, SC50, SC56, TAB153, TAB154, TAB156	IT, 7G8, SD01, HB3
