# Supplementary material for: Plasmodium falciparum surf4.1 in clinical isolates: From genetic variation and variant diversity to in silico design immunopeptides for vaccine development
Source: PLoS One. 2024 Dec 30;19(12):e0312091. doi: 10.1371/journal.pone.0312091 (PMC11684625; doi:10.1371/journal.pone.0312091)
Supplement: S3 Table — (PDF) [file pone.0312091.s003.pdf]

S3 Table. Difference of frequency between $SURFIN_{4.1}$, CNV and clinical outcomes.

Clinical outcomes	TMs variant	WDs variant	Total	Statistical significance
Mild malaria	12 (52.2%)	11 (47.8%)	23	Fisher's exact test (P = 0.1095, two-tailed)
Complicated malaria	39 (73.6%)	14 (26.4%)	53	
Total	51 (67.1%)	25 (32.9%)	76	
**Clinical outcomes**	**1CNV**	**≥2CNV**	**Total**	**Statistical significance**
Mild malaria	20 (90.9%)	2 (9.1%)	22	Fisher's exact test (P = 0.7094, two-tailed)
Complicated malaria	41 (85.4%)	7 (14.5%)	48	
Total	61 (87.1%)	9 (12.9%)	70	
**$SURFIN_{4.1}$ variants**	**1CNV**	**≥2CNV**	**Total**	**Statistical significance**
TMs variants	43 (89.6%)	5 (10.4%)	48	Fisher's exact test (P = 0.4350, two-tailed)
WDs variants	18 (81.8%)	4 (18.2%)	22	
Total	61(87%)	9 (13%)	70	
