# Supplementary material for: Plasmodium falciparum surf4.1 in clinical isolates: From genetic variation and variant diversity to in silico design immunopeptides for vaccine development
Source: PLoS One. 2024 Dec 30;19(12):e0312091. doi: 10.1371/journal.pone.0312091 (PMC11684625; doi:10.1371/journal.pone.0312091)
Supplement: S4 Table — (PDF) [file pone.0312091.s004.pdf]

S4 Table. Transmembrane topology location of $SURFIN_{4.1}$

Predicted Topologies Type	Subtype	Outside	Transmembrane	Inside
1. TMs	TM1<br>(TAB123)<br><br>TM2<br>(TAB141)	1-770<br><br>1-770	771-787<br>VPVALAVFGVLFVFILF<br>771-787<br>VPVALAVFGVLFVFILF	788-811<br><br><br>788-814
2. WD1 (TAB136)		1-770	771-787<br>VPVALAVFGVLFVFILF	788-1283
3. WD2 (FCR3)		1-778	779-788<br>FGVLFVFILF	789-1488
4. WD3 (TM156)		1-778	779-788<br>FGVLFVFILF	789-2224
