# Supplementary material for: Plasmodium falciparum surf4.1 in clinical isolates: From genetic variation and variant diversity to in silico design immunopeptides for vaccine development
Source: PLoS One. 2024 Dec 30;19(12):e0312091. doi: 10.1371/journal.pone.0312091 (PMC11684625; doi:10.1371/journal.pone.0312091)
Supplement: S1 File — (PDF) [file pone.0312091.s005.pdf]

```
                     10        20        30        40        50        60        70        80        90
             ....|....|....|....|....|....|....|....|....|....|....|....|....|....|....|....|....|....|
PF3D7 CDS    ATGCATTTTGTAGTTGAATTGGATAATACAGGTGATGATTTTAATGATAAGGCTATATCCATTGAAAGATTTAGAAATGTTTTTGAGGTT
MS822 mRNA   ---CATTTTGTAGTTGAATTGGATAATACAGGTGATGATTTTAATGATAAGGCTATATCCATTGAAAGATTTAGAAATGTTTTTGAGGTT
FCR3 mRNA    ---CATTTTGTAGTTGAATTGGATAATACAGGTGATGATTTTAATGATAAGGCTATATCCATTGAAAGATTTAGAAATGTTTTTGAGGTT
AM1898       ATGCATTTTGTAGTTGAATTGGATAATACAGGTGATGATTTTAATGATAAGGCTATATCCACTGAAAGATTTAGAAATGTTTTTGAGGTT
AA235        ATGCATTTTGTAGTTGAATTGGATAATACAGGTGATGATTTTAATGATAAGGCTATATCCATTGAAAGATTTAGAAATGTTTTTGAGGTT
AA243        ATGCATTTTGTAGTTGAATTGGATAATACAGGTGATGATTTTAATGATAAGGCTATATCCACTGAAAGATTTAGAAATGTTTTTGAGGTT
AA258        ATGCATTTTGTAGTTGAATTGGATAATACAGGTGATGATTTTAATGATAAGGCTATATCCACTGAAAGATTTAGAAATGTTTTTGAGGTT
AA264        ATGCATTTTGTAGTTGAATTGGATAATACAGGTGATGATTTTAATGATAAGGCTATATCCACTGAAAGATTTAGAAATGTTTTTGAGGTT
AM1737       ATGCATTTTGTAGTTGAATTGGATAATACAGGTGATGATTTTAATGATAAGGCTATATCCACTGAAAGATTTAGAAATGTTTTTGAGGTT
SC74         ATGCATTTTGTAGTTGAATTGGATAATACAGGTGATGATTTTAATGATAAGGCTATATCCACTGAAAGATTTAGAAATGTTTTTGAGGTT
TAB166       ATGCATTTTGTAGTTGAATTGGATAATACAGGTGATGATTTTAATGATAAGGCTATATCCACTGAAAGATTTAGAAATGTTTTTGAGGTT
TAB138       ATGCATTTTGTAGTTGAATTGGATAATACAGGTGATGATTTTAATGATAAGGCTATATCCACTGAAAGATTTAGAAATGTTTTTGAGGTT
TAB130       ATGCATTTTGTAGTTGAATTGGATAATACAGGTGATGATTTTAATGATAAGGCTATATCCACTGAAAGATTTAGAAATGTTTTTGAGGTT
TAB131       ATGCATTTTGTAGTTGAATTGGATAATACAGGTGATGATTTTAATGATAAGGCTATATCCACTGAAAGATTTAGAAATGTTTTTGAGGTT
TAB151       ATGCATTTTGTAGTTGAATTGGATAATACAGGTGATGATTTTAATGATAAGGCTATATCCACTGAAAGATTTAGAAATGTTTTTGAGGTT
TAB152       ATGCATTTTGTAGTTGAATTGGATAATACAGGTGATGATTTTAATGATAAGGCTATATCCATTGAAAGATTTAGAAATGTTTTTGAGGTT
SC52         ATGCATTTTGTAGTTGAATTGGATAATACAGGTGATGATTTTAATGATAAGGCTATATCCACTGAAAGATTTAGAAATGTTTTTGAGGTT
SC55         ATGCATTTTGTAGTTGCATTGGATAATACAGGTGATGATTTTAATGATAAGGCTATATCCACTGAAAGATTTAGAAATGTTTTTGAGGTT
SC03         ATGCATTTTGTAGTTGAATTGGATAATACAGGTGATGATTTTAATGATAAGGCTATATCCATTGAAAGATTTAGAAATGTTTTTGAGGTT
SC53         ATGCATTTTGTAGTTGAATTGGATAATACAGGTGATGATTTTAATGATAAGGCTATATCCATTGAAAGATTTAGAAATGTTTTTGAGGTT
TAB141       ATGCATTTTGTAGTTGAATTGGATAATACAGGTGATGATTTTAATGATAAGGCTATATCCATTGAAAGATTTAGAAATGTTTTTGAGGTT
TAB123       ATGCATTTTGTAGTTGAATTGGATAATACAGGTGATGATTTTAATGATAAGGCTATATCCACTGAAAGATTTAGAAATGTTTTTGAGGTT
AM1803       ATGCATTTTGTAGTTGCATTGGATAATACAGGTGATGATTTTAATGATAAGGCTATATCCACTGAAAGATTTAGAAATGTTTTTGAGGTT
AM1814       ATGCATTTTGTAGTTGCATTGGATAATACAGGTGATGATTTTAATGATAAGGCTATATCCACTGAAAGATTTAGAAATGTTTTTGAGGTT
AM1802       ATGCATTTTGTAGTTGAATTGGATAATACAGGTGATGATTTTAATGATAAGGCTATATCCACTGAAAGATTTAGAAATGTTTTTGAGGTT
AM1811       ATGCATTTTGTAGTTGCATTGGATAATACAGGTGATGATTTTAATGATAAGGCTATATCCACTGAAAGATTTAGAAATGTTTTTGAGGTT
SC50         ATGCATTTTGTAGTTGCATTGGATAATACAGGTGATGATTTTAATGATAAGGCTATATCCACTGAAAGATTTAGAAATGTTTTTGAGGTT
SC56         ATGCATTTTGTAGTTGCATTGGATAATACAGGTGATGATTTTAATGATAAGGCTATATCCACTGAAAGATTTAGAAATGTTTTTGAGGTT
TAB153       ATGCATTTTGTAGTTGAATTGGATAATACAGGTGATGATTTTAATGATAAGGCTATATCCACTGAAAGATTTAGAAATGTTTTTGAGGTT
TAB154       ATGCATTTTGTAGTTGAATTGGATAATACAGGTGATGATTTTAATGATAAGGCTATATCCACTGAAAGATTTAGAAATGTTTTTGAGGTT
TAB156       ATGCATTTTGTAGTTGCATTGGATAATACAGGTGATGATTTTAATGATAAGGCTATATCCACTGAAAGATTTAGAAATGTTTTTGAGGTT
TAB136       ATGCATTTTGTAGTTGCATTGGATAATACAGGTGATGATTTTAATGATAAGGCTATATCCACTGAAAGATTTAGAAATGTTTTTGAGGTT
AM1880       ATGCATTTTGTAGTTGCATTGGATAATACAGGTGATGATTTTAATGATAAGGCTATATCCACTGAAAGATTTAGAAATGTTTTTGAGGTT
PF3D7_       ATGCATTTTGTAGTTGAATTGGATAATACAGGTGATGATTTTAATGATAAGGCTATATCCATTGAAAGATTTAGAAATGTTTTTGAGGTT
PfSD01       ATGCATTTTGTAGTTGCATTGGATAATACAGGTGATGATTTTAATGATAAGGCTATATCCACTGAAAGATTTAGAAATGTTTTTGAGGTT
Pf7G8        ATGCATTTTGTAGTTGAATTGGATAATACAGGTGATGATTTTAATGATAAGGCTATATCCATTGAAAGATTTAGAAATGTTTTTGAGGTT
FCR3 GENE    ---CATTTTGTAGTTGAATTGGATAATACAGGTGATGATTTTAATGATAAGGCTATATCCATTGAAAGATTTAGAAATGTTTTTGAGGTT
MS822 GENE   ---CATTTTGTAGTTGAATTGGATAATACAGGTGATGATTTTAATGATAAGGCTATATCCATTGAAAGATTTAGAAATGTTTTTGAGGTT
PfCD01_      ATGCATTTTGTAGTTGAATTGGATAATACAGGTGATGATTTTAATGATAAGGCTATATCCACTGAAAGATTTAGAAATGTTTTTGAGGTT
PfKH01__     ATGCATTTTGTAGTTGAATTGGATAATACAGGTGATGATTTTAATGATAAGGCTATATCCACTGAAAGATTTAGAAATGTTTTTGAGGTT
PfDd2__      ATGCATTTTGTAGTTGAATTGGATAATACAGGTGATGATTTTAATGATAAGGCTATATCCACTGAAAGATTTAGAAATGTTTTTGAGGTT
PfSN01_      ATGCATTTTGTAGTTGAATTGGATAATACAGGTGATGATTTTAATGATAAGGCTATATCCACTGAAAGATTTAGAAATGTTTTTGAGGTT
PfGN01       ATGCATTTTGTAGTTGAATTGGATAATACAGGTGATGATTTTAATGATAAGGCTATATCCATTGAAAGATTTAGAAATGTTTTTGAGGTT
PfKE01       ATGCATTTTGTAGTTGAATTGGATAATACAGGTGATGATTTTAATGATAAGGCTATATCCATTGAAAGATTTAGAAATGTTTTTGAGGTT
PfKH02       ATGCATTTTGTAGTTGAATTGGATAATACAGGTGATGATTTTAATGATAAGGCTATATCCATTGAAAGATTTAGAAATGTTTTTGAGGTT
PfGB4        ATGCATTTTGTAGTTGAATTGGATAATACAGGTGATGATTTTAATGATAAGGCTATATCCATTGAAAGATTTAGAAATGTTTTTGAGGTT
PfGA01_      ATGCATTTTGTAGTTGAATTGGATAATACAGGTGATGATTTTAATGATAAGGCTATATCCACTGAAAGATTTAGAAATGTTTTTGAGGTT
PfHB3_       ATGCATTTTGTAGTTGCATTGGATAATACAGGTGATGATTTTAATGATAAGGCTATATCCACTGAAAGATTTAGAAATGTTTTTGAGGTT
PfIT_        ATGCATTTTGTAGTTGCATTGGATAATACAGGTGATGATTTTAATGATAAGGCTATATCCACTGAAAGATTTAGAAATGTTTTTGAGGTT
```

	100 110 120 130 140 150 160 170 180
PF3D7 CDS	TATGTTGAAGACAAAATAGATGAATTAGATACAAAACCATCAGAAATACTTAATAAAGAATGTCGACATTTTAATTATTTTATTGATGAT
MS822 mRNA	TATGTTGAAGACAAAATAGATGAATTAGATACAAAACCATCAGAAATACTTAATAAAGAATGTCGACATTTTAATTATTTTATTGATGAT
FCR3 mRNA	TATGTTGAAGACAAAATAGATGAATTAGATAGAAAACCATCAGAAATTCTTAATAAAGAATGTCGACATTTTAATTATTTTATTGATGAT
AM1898	TATGTTGAAGACAAAATAGATGAATTAGATACAAAACCATCAGAAATACTTAATAAAGAATGTCGACATTTTAATTATTTTATTGATGAT
AA235	TATGTTGAAGACAAAATAGATGAATTAGATACAAAACCATCAGAAATACTTAATAAAGAATGTCGACATTTTAATTATTTTATTGATGAT
AA243	TATGTTGAAGACAAAATAGATGAATTAGATACAAAACCATCAGAAATACTTAATAAAGAATGTCGACATTTTAATTATTTTATTGATGAT
AA258	TATGTTGAAGACAAAATAGATGAATTAGATACAAAACCATCAGAAATACTTAATAAAGAATGTCGACATTTTAATTATTTTATTGATGAT
AA264	TATGTTGAAGACAAAATAGATGAATTAGATACAAAACCATCAGAAATACTTAATAAAGAATGTCGACATTTTAATTATTTTATTGATGAT
AM1737	TATGTTGAAGACAAAATAGATGAATTAGATACAAAACCATCAGAAATACTTAATAAAGAATGTCGACATTTTAATTATTTTATTGATGAT
SC74	TATGTTGAAGACAAAATAGATGAATTAGATACAAAACCATCAGAAATACTTAATAAAGAATGTCGACATTTTAATTATTTTATTGATGAT
TAB166	TATGTTGAAGACAAAATAGATGAATTAGATACAAAACCATCAGAAATACTTAATAAAGAATGTCGACATTTTAATTATTTTATTGATGAT
TAB138	TATGTTGAAGACAAAATAGATGAATTAGATACAAAACCATCAGAAATACTTAATAAAGAATGTCGACATTTTAATTATTTTATTGATGAT
TAB130	TATGTTGAAGACAAAATAGATGAATTAGATACAAAACCATCAGAAATACTTAATAAAGAATGTCGACATTTTAATTATTTTATTGATGAT
TAB131	TATGTTGAAGACAAAATAGATGAATTAGATACAAAACCATCAGAAATACTTAATAAAGAATGTCGACATTTTAATTATTTTATTGATGAT
TAB151	TATGTTGAAGACAAAATAGATGAATTAGATACAAAACCATCAGAAATACTTAATAAAGAATGTCGACATTTTAATTATTTTATTGATGAT
TAB152	TATGTTGAAGACAAAATAGATGAATTAGATACAAAACCATCAGAAATACTTAATAAAGAATGTCGACATTTTAATTATTTTATTGATGAT
SC52	TATGTTGAAGACAAAATAGATGAATTAGATACAAAACCATCAGAAATACTTAATAAAGAATGTCGACATTTTAATTATTTTATTGATGAT
SC55	TATGTTGAAGACAAAATAGATGAATTAGATACAAAACCATCAGAAATACTTAATAAAGAATGTCGACATTTTAATTATTTTATTGATGAT
SC03	TATGTTGAAGACAAAATAGATGAATTAGATACAAAACCATCAGAAATACTTAATAAAGAATGTCGACATTTTAATTATTTTATTGATGAT
SC53	TATGTTGAAGACAAAATAGATGAATTAGATACAAAACCATCAGAAATACTTAATAAAGAATGTCGACATTTTAATTATTTTATTGATGAT
TAB141	TATGTTGAAGACAAAATAGATGAATTAGATACAAAACCATCAGAAATACTTAATAAAGAATGTCGACATTTTAATTATTTTATTGATGAT
TAB123	TATGTTGAAGACAAAATAGATGAATTAGATACAAAACCATCAGAAATACTTAATAAAGAATGTCGACATTTTAATTATTTTATTGATGAT
AM1803	TATGTTGAAGACAAAATAGATGAATTAGATACAAAACCATCAGAAATACTTAATAAAGAATGTCGACATTTTAATTATTTTATTGATGAT
AM1814	TATGTTGAAGACAAAATAGATGAATTAGATACAAAACCATCAGAAATACTTAATAAAGAATGTCGACATTTTAATTATTTTATTGATGAT
AM1802	TATGTTGAAGACAAAATAGATGAATTAGATACAAAACCATCAGAAATACTTAATAAAGAATGTCGACATTTTAATTATTTTATTGATGAT
AM1811	TATGTTGAAGACAAAATAGATGAATTAGATACAAAACCATCAGAAATACTTAATAAAGAATGTCGACATTTTAATTATTTTATTGATGAT
SC50	TATGTTGAAGACAAAATAGATGAATTAGATACAAAACCATCAGAAATACTTAATAAAGAATGTCGACATTTTAATTATTTTATTGATGAT
SC56	TATGTTGAAGACAAAATAGATGAATTAGATACAAAACCATCAGAAATACTTAATAAAGAATGTCGACATTTTAATTATTTTATTGATGAT
TAB153	TATGTTGAAGACAAAATAGATGAATTAGATACAAAACCATCAGAAATACTTAATAAAGAATGTCGACATTTTAATTATTTTATTGATGAT
TAB154	TATGTTGAAGACAAAATAGATGAATTAGATACAAAACCATCAGAAATACTTAATAAAGAATGTCGACATTTTAATTATTTTATTGATGAT
TAB156	TATGTTGAAGACAAAATAGATGAATTAGATACAAAACCATCAGAAATACTTAATAAAGAATGTCGACATTTTAATTATTTTATTGATGAT
TAB136	TATGTTGAAGACAAAATAGATGAATTAGATACAAAACCATCAGAAATACTTAATAAAGAATGTCGACATTTTAATTATTTTATTGATGAT
AM1880	TATGTTGAAGACAAAATAGATGAATTAGATACAAAACCATCAGAAATACTTAATAAAGAATGTCGACATTTTAATTATTTTATTGATGAT
PF3D7_	TATGTTGAAGACAAAATAGATGAATTAGATACAAAACCATCAGAAATACTTAATAAAGAATGTCGACATTTTAATTATTTTATTGATGAT
PfSD01	TATGTTGAAGACAAAATAGATGAATTAGATACAAAACCATCAGAAATACTTAATAAAGAATGTCGACATTTTAATTATTTTATTGATGAT
Pf7G8	TATGTTGAAGACAAAATAGATGAATTAGATACAAAACCATCAGAAATACTTAATAAAGAATGTCGACATTTTAATTATTTTATTGATGAT
FCR3 GENE	TATGTTGAAGACAAAATAGATGAATTAGATAGAAAACCATCAGAAATTCTTAATAAAGAATGTCGACATTTTAATTATTTTATTGATGAT
MS822 GENE	TATGTTGAAGACAAAATAGATGAATTAGATACAAAACCATCAGAAATACTTAATAAAGAATGTCGACATTTTAATTATTTTATTGATGAT
PfCD01_	TATGTTGAAGACAAAATAGATGAATTAGATACAAAACCATCAGAAATACTTAATAAAGAATGTCGACATTTTAATTATTTTATTGATGAT
PfKH01__	TATGTTGAAGACAAAATAGATGAATTAGATACAAAACCATCAGAAATACTTAATAAAGAATGTCGACATTTTAATTATTTTATTGATGAT
PfDd2__	TATGTTGAAGACAAAATAGATGAATTAGATACAAAACCATCAGAAATACTTAATAAAGAATGTCGACATTTTAATTATTTTATTGATGAT
PfSN01_	TATGTTGAAGACAAAATAGATGAATTAGATACAACACCATCAGAAATACTTAATAAAGAATGTCGACATTTTAATTATTTTATTGATGAT
PfGN01	TATGTTGAAGACAAAATAGATGAATTAGATACAAAACCATCAGAAATACTTAATAAAGAATGTCGACATTTTAATTATTTTATTGATGAT
PfKE01	TATGTTGAAGACAAAATAGATGAATTAGATACAAAACCATCAGAAATACTTAATAAAGAATGTCGACATTTTAATTATTTTATTGATGAT
PfKH02	TATGTTGAAGACAAAATAGATGAATTAGATACAAAACCATCAGAAATACTTAATAAAGAATGTCGACATTTTAATTATTTTATTGATGAT
PfGB4	TATGTTGAAGACAAAATAGATGAATTAGATACAAAACCATCAGAAATACTTAATAAAGAATGTCGACATTTTAATTATTTTATTGATGAT
PfGA01_	TATGTTGAAGACAAAATAGATGAATTAGATACAACACCATCAGAAATACTTAATAAAGAATGTCGACATTTTAATTATTTTATTGATGAT
PfHB3_	TATGTTGAAGACAAAATAGATGAATTAGATACAAAACCATCAGAAATACTTAATAAAGAATGTCGACATTTTAATTATTTTATTGATGAT
PfIT_	TATGTTGAAGACAAAATAGATGAATTAGATAGAAAACCATCAGAAATTCTTAATAAAGAATGTCGACATTTTAATTATTTTATTGATGAT

```
                      190       200       210       220       230       240       250       260       270
              ....|....|....|....|....|....|....|....|....|....|....|....|....|....|....|....|....|....|
PF3D7 CDS     ATGAAAGATGAGTTTCTCACGTCTTCATTAATACGTCTCCCCAAAAAATTAAGAAAGCAATTATGGGAAAGTGAAGTTGATGAAAATTTA
MS822 mRNA    ATGAAAGATGAGTTTCTCACGTCTTCATTAATACGTCTCCCCAAAAAATTAAGAAAGCAATTATGGGAAAGTGAAGTTGATGAAAATTTA
FCR3 mRNA     ATGAAAGATGAGTTTCTCACGTCTTCATTAATACGTCTCCCTAAAGAATTAAGAAAGCAATTATGGGAAAGTGAAGTTGATGAAAAATTA
AM1898        ATGAAAGATGAGTTTCTCACGTCTTCATTAATACGTCTCTCTAAAGAATTAAGAAAGCAATTATGGGAAAGTGAAGTTGATGAAAAATTA
AA235         ATGAAAGATGAGTTTCTCACGTCTTCATTAATACGTCTCCCTAAAAAATTAAGAAAGCAATTATGGGAAAGTGAAGTTGATGAAAATTTA
AA243         ATGAAAGATGAGTTTCTCACGTCTTCATTAATACGTCTCCCTAAAAAATTAAGAAAGCAATTATGGGAAAGTGAAGTTGATGAAAACTTA
AA258         ATGAAAGATGAGTTTCTCACGTCTTCATTAATACGTCTCCCTAAAAAATTAAGAAAGCAATTATGGGAAAGTGAAGTTGATGAAAACTTA
AA264         ATGAAAGATGAGTTTCTCACGTCTTCATTAATACGTCTCCCTAAAAAATTAAGAAAGCAATTATGGGAAAGTGAAGTTGATGAAAACTTA
AM1737        ATGAAAGATGAGTTTCTCACGTCTTCATTAATACGTCTCCCTAAAAAATTAAGAAAGCAATTATGGGAAAGTGAAGTTGATGAAAACTTA
SC74          ATGAAAGATGAGTTTCTCACGTCTTCATTAATACGTCTCCCTAAAAAATTAAGAAAGCAATTATGGGAAAGTGAAGTTGATGAAAACTTA
TAB166        ATGAAAGATGAGTTTCTCACGTCTTCATTAATACGTCTCCCTAAAAAATTAAGAAAGCAATTATGGGAAAGTGAAGTTGATGAAAACTTA
TAB138        ATGAAAGATGAGTTTCTCACGTCTTCATTAATACGTCTCCCTAAAAAATTAAGAAAGCAATTATGGGAAAGTGAAGTTGATGAAAACTTA
TAB130        ATGAAAGATGAGTTTCTCACGTCTTCATTAATACGTCTCCCTAAAAAATTAAGAAAGCAATTATGGGAAAGTGAAGTTGATGAAAACTTA
TAB131        ATGAAAGATGAGTTTCTCACGTCTTCATTAATACGTCTCCCTAAAAAATTAAGAAAGCAATTATGGGAAAGTGAAGTTGATGAAAACTTA
TAB151        ATGAAAGATGAGTTTCTCACGTCTTCATTAATACGTCTCCCTAAAAAATTAAGAAAGCAATTATGGGAAAGTGAAGTTGATGAAAACTTA
TAB152        ATGAAAGATGAGTTTCTCACGTCTTCATTAATACGTCTCCCTAAAAAATTAAGAAAGCAATTATGGGAAAGTGAAGTTGATGAAAACTTA
SC52          ATGAAAGATGAGTTTCTCACGTCTTCATTAATACGTCTCCCTAAAAAATTAAGAAAGCAATTATGGGAAAGTGAAGTTGATGAAAACTTA
SC55          ATGAAAGATGAGTTTCTCACGTCTTCATTAATACGTCTCCCTAAAAAATTAAGAAAGCAATTATGGGAAAGTGAAGTTGATGAAAACTTA
SC03          ATGAAAGATGAGTTTCTCACGTCTTCATTAATACGTCTCCCCAAAAAATTAAGAAAGCAATTATGGGAAAGTGAAGTTGATAAAAACTTA
SC53          ATGAAAGATGAGTTTCTCACGTCTTCATTAATACGTCTCCCTAAAAAATTAAGAAAGCAATTATGGGAAAGTGAAGTTGATGAAAACTTA
TAB141        ATGAAAGATGAGTTTCTCACGTCTTCATTAATACGTCTCCCCAAAAAATTAAGAAAGCAATTATGGGAAAGTGAAGTTGATGAAAATTTA
TAB123        ATGAAAGATGAGTTTCTCACGTCTTCATTAATACGTCTCCCTAAAAAATTAAGAAAGCAATTATGGGAAAGTGAAGTTGATGAAAACTTA
AM1803        ATGAAAGATGAGTTTCTCAAGTCTTCATTAATACGTCTCTCTAAAGAATTAAGAAAGCAATTATGGGAAAGTGAAGTTGATGAAAAATTA
AM1814        ATGAAAGATGAGTTTCTCACGTCTTCATTAATACGTCTCTCTAAAGAATTAAGAAAGCAATTATGGGAAAGTGAAGTTGATGAAAAATTA
AM1802        ATGAAAGATGAGTTTCTCACGTCTTCATTAATACGTCTCCCTAAAAAATTAAGAAAGCAATTATGGGAAAGTGAAGTTGATGAAAACTTA
AM1811        ATGAAAGATGAGTTTCTCAAGTCTTCATTAATACGTCTCTCTAAAGAATTAAGAAAGCAATTATGGGAAAGTGAAGTTGATGAAAAATTA
SC50          ATGAAAGATGAGTTTCTCAAGTCTTCATTAATACGTCTCTCTAAAGAATTAAGAAAGCAATTATGGGAAAGTGAAGTTGATGAAAAATTA
SC56          ATGAAAGATGAGTTTCTCAAGTCTTCATTAATACGTCTCTCTAAAGAATTAAGAAAGCAATTATGGGAAAGTGAAGTTGATGAAAAATTA
TAB153        ATGAAAGATGAGTTTCTCACGTCTTCATTAATACGTCTCCCTAAAAAATTAAGAAAGCAATTATGGGAAAGTGAAGTTGATGAAAACTTA
TAB154        ATGAAAGATGAGTTTCTCACGTCTTCATTAATACGTCTCCCTAAAAAATTAAGAAAGCAATTATGGGAAAGTGAAGTTGATGAAAACTTA
TAB156        ATGAAAGATGAGTTTCTCAAGTCTTCATTAATACGTCTCTCTAAAGAATTAAGAAAGCAATTATGGGAAAGTGAAGTTGATGAAAAATTA
TAB136        ATGAAAGATGAGTTTCTCACGTCTTCATTAATACGTCTCTCTAAAGAATTAAGAAAGCAATTATGGGAAAGTGAAGTTGATGAAAAATTA
AM1880        ATGAAAGATGAGTTTCTCACGTCTTCATTAATACGTCTCTCTAAAGAATTAAGAAAGCAATTATGGGAAAGTGAAGTTGATGAAAAATTA
PF3D7_        ATGAAAGATGAGTTTCTCACGTCTTCATTAATACGTCTCCCCAAAAAATTAAGAAAGCAATTATGGGAAAGTGAAGTTGATGAAAATTTA
PfSD01        ATGAAAGATGAGTTTCTCAAGTCTTCATTAATACGTCTCTCTAAAGAATTAAGAAAGCAATTATGGGAAAGTGAAGTTGATGAAAAATTA
Pf7G8         ATGAAAGATGAGTTTCTCACGTCTTCATTAATACGTCTCCCCAAAAAATTAAGAAAGCAATTATGGGAAAGTGAAGTTGATGAAAATTTA
FCR3 GENE     ATGAAAGATGAGTTTCTCACGTCTTCATTAATACGTCTCCCTAAAGAATTAAGAAAGCAATTATGGGAAAGTGAAGTTGATGAAAAATTA
MS822 GENE    ATGAAAGATGAGTTTCTCACGTCTTCATTAATACGTCTCCCCAAAAAATTAAGAAAGCAATTATGGGAAAGTGAAGTTGATGAAAATTTA
PfCD01_       ATGAAAGATGAGTTTCTCACGTCTTCATTAATACGTCTCCCTAAAGAATTAAGAAAGCAATTATGGGAAAGTGAAGTTGATAAAAACTTA
PfKH01__      ATGAAAGATGAGTTTCTCACGTCTTCATTAATACGTCTCCCTAAAAAATTAAGAAAGCAATTATGGGAAAGTGAAGTTGATGAAAACTTA
PfDd2__       ATGAAAGATGAGTTTCTCACGTCTTCATTAATACGTCTCCCTAAAAAATTAAGAAAGCAATTATGGGAAAGTGAAGTTGATGAAAACTTA
PfSN01_       ATGAAAGATGAGTTTCTCACGTCTTCATTAATACGTCTCCCCAAAAAATTAAGAAAGCAATTATGGGAAAGTGAAGTTGATGAAAATTTA
PfGN01        ATGAAAGATGAGTTTCTCACGTCTTCATTAATACGTCTCCCCAAAAAATTAAGAAAGCAATTATGGGAAAGTGAAGTTGATAAAAACTTA
PfKE01        ATGAAAGATGAGTTTCTCACGTCTTCATTAATACGTCTCCCCAAAAAATTAAGAAAGCAATTATGGGAAAGTGAAGTTGATGAAAATTTA
PfKH02        ATGAAAGATGAGTTTCTCACGTCTTCATTAATACGTCTCCCCAAAAAATTAAGAAAGCAATTATGGGAAAGTGAAGTTGATAAAAACTTA
PfGB4         ATGAAAGATGAGTTTCTCACGTCTTCATTAATACGTCTCCCCAAAAAATTAAGAAAGCAATTATGGGAAAGTGAAGTTGATGAAAATTTA
PfGA01_       ATGAAAGATGAGTTTCTCACGTCTTCATTAATACGTCTCCCCAAAAAATTAAGAAAGCAATTATGGGAAAGTGAAGTTGATAAAAACTTA
PfHB3_        ATGAAAGATGAGTTTCTCAAGTCTTCATTAATACGTCTCTCTAAAGAATTAAGAAAGCAATTATGGGAAAGTGAAGTTGATGAAAAATTA
PfIT_         ATGAAAGATGAGTTTCTCACGTCTTCATTAATACGTCTCCCTAAAGAATTAAGAAAGCAATTATGGGAAAGTGAAGTTGATGAAAAATTA
```

	280 290 300 310 320 330 340 350 360
PF3D7 CDS	CCAAATTTAATGGCTACAACTACACATAATAAGTGTCTAAGAACAGAACATAATTATGATAAAAAGTATAGGGACGTCATTAAAATATTA
MS822 mRNA	CCAAATTTAATGGCTACAACTACACATAATAAGTGTCTAAGAACAGAACATAATTATGATAAAAAGTATAGGGACGTCATTAAAATATTA
FCR3 mRNA	CCAAATTTAATGGCTACAACTACACATAATAAATGTCTAAGAACAGAACATAATTATGATAAAAAGTATAGGGACGTCATTAAAATATTA
AM1898	CCAAATTTAATGGCTACAACTACACATAATAAATGTCTAAGAACAGAACATAATTATGATAAAAAGTATAGGGACGTCATTAAAATATTA
AA235	CCAAATTTAATGGCTACAACTACACATAATAAGTGTCTAAGAACAGAACATAATTATGATAAAAAGTATAGGGACGTCATTAAAATATTA
AA243	CCAAATTTAATGGCTACAACTACACATAATAAATGTCTAAGAACAGAACATAATTATGATAAAAAGTATAGGGACGTCATTAAAATATTA
AA258	CCAAATTTAATGGCTACAACTACACATAATAAATGTCTAAGAACAGAACATAATTATGATAAAAAGTATAGGGACGTCATTAAAATATTA
AA264	CCAAATTTAATGGCTACAACTACACATAATAAATGTCTAAGAACAGAACATAATTATGATAAAAAGTATAGGGACGTCATTAAAATATTA
AM1737	CCAAATTTAATGGCTACAACTACACATAATAAATGTCTAAGAACAGAACATAATTATGATAAAAAGTATAGGGACGTCATTAAAATATTA
SC74	CCAAATTTAATGGCTACAACTACACATAATAAATGTCTAAGAACAGAACATAATTATGATAAAAAGTATAGGGACGTCATTAAAATATTA
TAB166	CCAAATTTAATGGCTACAACTACACATAATAAATGTCTAAGAACAGAACATAATTATGATAAAAAGTATAGGGACGTCATTAAAATATTA
TAB138	CCAAATTTAATGGCTACAACTACACATAATAAATGTCTAAGAACAGAACATAATTATGATAAAAAGTATAGGGACGTCATTAAAATATTA
TAB130	CCAAATTTAATGGCTACAACTACACATAATAAATGTCTAAGAACAGAACATAATTATGATAAAAAGTATAGGGACGTCATTAAAATATTA
TAB131	CCAAATTTAATGGCTACAACTACACATAATAAATGTCTAAGAACAGAACATAATTATGATAAAAAGTATAGGGACGTCATTAAAATATTA
TAB151	CCAAATTTAATGGCTACAACTACACATAATAAATGTCTAAGAACAGAACATAATTATGATAAAAAGTATAGGGACGTCATTAAAATATTA
TAB152	CCAAATTTAATGGCTACAACTACACATAATAAGTGTCTAAGAACAGAACATAATTATGATAAAAAGTATAGGGACGTCATTAAAATATTA
SC52	CCAAATTTAATGGCTACAACTACACATAATAAATGTCTAAGAACAGAACATAATTATGATAAAAAGTATAGGGACGTCATTAAAATATTA
SC55	CCAAATTTAATGGCTACAACTACACATAATAAATGTCTAAGAACAGAACATAATTATGATAAAAAGTATAGGGACGTCATTAAAATATTA
SC03	CCAAATTTAATGGCTACAACTACACATAATAAGTGTCTAAGAACAGAACATAATTATGATAAAAAGTATAGGGACGTCATTAAAATATTA
SC53	CCAAATTTAATGGCTACAACTACACATAATAAGTGTCTAAGAACAGAACATAATTATGATAAAAAGTATAGGGACGTCATTAAAATATTA
TAB141	CCAAATTTAATGGCTACAACTACACATAATAAGTGTCTAAGAACAGAACATAATTATGATAAAAAGTATAGGGACGTCATTAAAATATTA
TAB123	CCAAATTTAATGGCTACAACTACACATAATAAATGTCTAAGAACAGAACATAATTATGATAAAAAGTATAGGGACGTCATTAAAATATTA
AM1803	CCAAATTTAATGGCTACAACTACACATAATAAATGTCTAAGAACAGAACATAATTATGATAAAAAGTATAGGGACGTCATTAAAATATTA
AM1814	CCAAATTTAATGGCTACAACTACACATAATAAATGTCTAAGAACAGAACATAATTATGATAAAAAGTATAGGGACGTCATTAAAATATTA
AM1802	CCAAATTTAATGGCTACAACTACACATAATAAATGTCTAAGAACAGAACATAATTATGATAAAAAGTATAGGGACGTCATTAAAATATTA
AM1811	CCAAATTTAATGGCTACAACTACACATAATAAATGTCTAAGAACAGAACATAATTATGATAAAAAGTATAGGGACGTCATTAAAATATTA
SC50	CCAAATTTAATGGCTACAACTACACATAATAAATGTCTAAGAACAGAACATAATTATGATAAAAAGTATAGGGACGTCATTAAAATATTA
SC56	CCAAATTTAATGGCTACAACTACACATAATAAATGTCTAAGAACAGAACATAATTATGATAAAAAGTATAGGGACGTCATTAAAATATTA
TAB153	CCAAATTTAATGGCTACAACTACACATAATAAATGTCTAAGAACAGAACATAATTATGATAAAAAGTATAGGGACGTCATTAAAATATTA
TAB154	CCAAATTTAATGGCTACAACTACACATAATAAATGTCTAAGAACAGAACATAATTATGATAAAAAGTATAGGGACGTCATTAAAATATTA
TAB156	CCAAATTTAATGGCTACAACTACACATAATAAATGTCTAAGAACAGAACATAATTATGATAAAAAGTATAGGGACGTCATTAAAATATTA
TAB136	CCAAATTTAATGGCTACAACTACACATAATAAATGTCTAAGAACAGAACATAATTATGATAAAAAGTATAGGGACGTCATTAAAATATTA
AM1880	CCAAATTTAATGGCTACAACTACACATAATAAATGTCTAAGAACAGAACATAATTATGATAAAAAGTATAGGGACGTCATTAAAATATTA
PF3D7_	CCAAATTTAATGGCTACAACTACACATAATAAGTGTCTAAGAACAGAACATAATTATGATAAAAAGTATAGGGACGTCATTAAAATATTA
PfSD01	CCAAATTTAATGGCTACAACTACACATAATAAATGTCTAAGAACAGAACATAATTATGATAAAAAGTATAGGGACGTCATTAAAATATTA
Pf7G8	CCAAATTTAATGGCTACAACTACACATAATAAGTGTCTAAGAACAGAACATAATTATGATAAAAAGTATAGGGACGTCATTAAAATATTA
FCR3 GENE	CCAAATTTAATGGCTACAACTACACATAATAAATGTCTAAGAACAGAACATAATTATGATAAAAAGTATAGGGACGTCATTAAAATATTA
MS822 GENE	CCAAATTTAATGGCTACAACTACACATAATAAGTGTCTAAGAACAGAACATAATTATGATAAAAAGTATAGGGACGTCATTAAAATATTA
PfCD01_	CCAAATTTAATGGCTACAACTACACATAATAAGTGTCTAAGAACAGAACATAATTATGATAAAAAGTATAGGGACGTCATTAAAATATTA
PfKH01__	CCAAATTTAATGGCTACAACTACACATAATAAATGTCTAAGAACAGAACATAATTATGATAAAAAGTATAGGGACGTCATTAAAATATTA
PfDd2__	CCAAATTTAATGGCTACAACTACACATAATAAATGTCTAAGAACAGAACATAATTATGATAAAAAGTATAGGGACGTCATTAAAATATTA
PfSN01_	CCAAATTTAATGGCTACAACTACACATAATAAGTGTCTAAGAACAGAACATAATTATGATAAAAAGTATAGGGACGTCATTAAAATATTA
PfGN01	CCAAATTTAATGGCTACAACTACACATAATAAATGTCTAAGAACAGAACATAATTATGATAAAAAGTATAGGGACGTCATTAAAATATTA
PfKE01	CCAAATTTAATGGCTACAACTACACATAATAAATGTCTAAGAACAGAACATAATTATGATAAAAAGTATAGGGACGTCATTAAAATATTA
PfKH02	CCAAATTTAATGGCTACAACTACACATAATAAGTGTCTAAGAACAGAACATAATTATGATAAAAAGTATAGGGACGTCATTAAAATATTA
PfGB4	CCAAATTTAATGGCTACAACTACACATAATAAGTGTCTAAGAACAGAACATAATTATGATAAAAAGTATAGGGACGTCATTAAAATATTA
PfGA01_	CCAAATTTAATGGCTACAACTACACATAATAAATGTCTAAGAACAGAACATAATTATGATAAAAAGTATAGGGACGTCATTAAAATATTA
PfHB3_	CCAAATTTAATGGCTACAACTACACATAATAAATGTCTAAGAACAGAACATAATTATGATAAAAAGTATAGGGACGTCATTAAAATATTA
PfIT_	CCAAATTTAATGGCTACAACTACACATAATAAATGTCTAAGAACAGAACATAATTATGATAAAAAGTATAGGGACGTCATTAAAATATTA

```
                   370       380       390       400       410       420       430       440       450
               ....|....|....|....|....|....|....|....|....|....|....|....|....|....|....|....|....|....|
PF3D7 CDS      GAAGATTATTGTGAAGATAGGGCTACTAAATTGAGGGATTTAAAAGCCATAAAATATGCAGAACAGGATTGTATTAATTTTAATACCTGG
MS822 mRNA     GAAGATTATTGTGAAGATAGGGCTACTAAATTGAGGGATTTAAAAGCCATAAAATATGCAGAACAGGATTGTATTAATTTTAATACCTGG
FCR3 mRNA      GAAGATTATTGTGAAGATAGGGCTACTAAATTGAGGGATTTAAAAGCCATAAAATATGCAGAACAGGATTGTATTAATTTTAATACCTGG
AM1898         GAAGATTATTGTGAAGATAGGGCTACTAAATTGAGGGATTTAAAAGCCATAAAATATGCAGAACAGGATTGTATTAATTTTAATACCTGG
AA235          GAAGATTATTGTGAAGATAGGGCTACTAAATTGAGGGATTTAAAAGCCATAAAATATGCAGAACAGGATTGTATTAATTTTAATACCTGG
AA243          GAAGATTATTGTGAAGATAGGGCTACTAAATTGAGGGATTTAAAAGCCATAAAATATGCAGAACAGGATTGTATTAATTTTAATACCTGG
AA258          GAAGATTATTGTGAAGATAGGGCTACTAAATTGAGGGATTTAAAAGCCATAAAATATGCAGAACAGGATTGTATTAATTTTAATACCTGG
AA264          GAAGATTATTGTGAAGATAGGGCTACTAAATTGAGGGATTTAAAAGCCATAAAATATGCAGAACAGGATTGTATTAATTTTAATACCTGG
AM1737         GAAGATTATTGTGAAGATAGGGCTACTAAATTGAGGGATTTAAAAGCCATAAAATATGCAGAACAGGATTGTATTAATTTTAATACCTGG
SC74           GAAGATTATTGTGAAGATAGGGCTACTAAATTGAGGGATTTAAAAGCCATAAAATATGCAGAACAGGATTGTATTAATTTTAATACCTGG
TAB166         GAAGATTATTGTGAAGATAGGGCTACTAAATTGAGGGATTTAAAAGCCATAAAATATGCAGAACAGGATTGTATTAATTTTAATACCTGG
TAB138         GAAGATTATTGTGAAGATAGGGCTACTAAATTGAGGGATTTAAAAGCCATAAAATATGCAGAACAGGATTGTATTAATTTTAATACCTGG
TAB130         GAAGATTATTGTGAAGATAGGGCTACTAAATTGAGGGATTTAAAAGCCATAAAATATGCAGAACAGGATTGTATTAATTTTAATACCTGG
TAB131         GAAGATTATTGTGAAGATAGGGCTACTAAATTGAGGGATTTAAAAGCCATAAAATATGCAGAACAGGATTGTATTAATTTTAATACCTGG
TAB151         GAAGATTATTGTGAAGATAGGGCTACTAAATTGAGGGATTTAAAAGCCATAAAATATGCAGAACAGGATTGTATTAATTTTAATACCTGG
TAB152         GAAGATTATTGTGAAGATAGGGCTACTAAATTGAGGGATTTAAAAGCCATAAAATATGCAGAACAGGATTGTATTAATTTTAATACCTGG
SC52           GAAGATTATTGTGAAGATAGGGCTACTAAATTGAGGGATTTAAAAGCCATAAAATATGCAGAACAGGATTGTATTAATTTTAATACCTGG
SC55           GAAGATTATTGTGAAGATAGGGCTACTAAATTGAGGGATTTAAAAGCCATAAAATATGCAGAACAGGATTGTATTAATTTTAATACCTGG
SC03           GAAGATTATTGTGAAGATAGGGCTACTAAATTGAGGGATTTAAAAGCCATAAAATATGCAGAACAGGATTGTATTAATTTTAATACCTGG
SC53           GAAGATTATTGTGAAGATAGGGCTACTAAATTGAGGGATTTAAAAGCCATAAAATATGCAGAACAGGATTGTATTAATTTTAATACCTGG
TAB141         GAAGATTATTGTGAAGATAGGGCTACTAAATTGAGGGATTTAAAAGCCATAAAATATGCAGAACAGGATTGTATTAATTTTAATACCTGG
TAB123         GAAGATTATTGTGAAGATAGGGCTACTAAATTGAGGGATTTAAAAGCCATAAAATATGCAGAACAGGATTGTATTAATTTTAATACCTGG
AM1803         GAAGATTATTGTGAAGATAGGGCTACTAAATTGAGGGATTTAAAAGCCATAAAATATGCAGAACAGGATTGTATTAATTTTAATACCTGG
AM1814         GAAGATTATTGTGAAGATAGGGCTACTAAATTGAGGGATTTAAAAGCCATAAAATATGCAGAACAGGATTGTATTAATTTTAATACCTGG
AM1802         GAAGATTATTGTGAAGATAGGGCTACTAAATTGAGGGATTTAAAAGCCATAAAATATGCAGAACAGGATTGTATTAATTTTAATACCTGG
AM1811         GAAGATTATTGTGAAGATAGGGCTACTAAATTGAGGGATTTAAAAGCCATAAAATATGCAGAACAGGATTGTATTAATTTTAATACCTGG
SC50           GAAGATTATTGTGAAGATAGGGCTACTAAATTGAGGGATTTAAAAGCCATAAAATATGCAGAACAGGATTGTATTAATTTTAATACCTGG
SC56           GAAGATTATTGTGAAGATAGGGCTACTAAATTGAGGGATTTAAAAGCCATAAAATATGCAGAACAGGATTGTATTAATTTTAATACCTGG
TAB153         GAAGATTATTGTGAAGATAGGGCTACTAAATTGAGGGATTTAAAAGCCATAAAATATGCAGAACAGGATTGTATTAATTTTAATACCTGG
TAB154         GAAGATTATTGTGAAGATAGGGCTACTAAATTGAGGGATTTAAAAGCCATAAAATATGCAGAACAGGATTGTATTAATTTTAATACCTGG
TAB156         GAAGATTATTGTGAAGATAGGGCTACTAAATTGAGGGATTTAAAAGCCATAAAATATGCAGAACAGGATTGTATTAATTTTAATACCTGG
TAB136         GAAGATTATTGTGAAGATAGGGCTACTAAATTGAGGGATTTAAAAGCCATAAAATATGCAGAACAGGATTGTATTAATTTTAATACCTGG
AM1880         GAAGATTATTGTGAAGATAGGGCTACTAAATTGAGGGATTTAAAAGCCATAAAATATGCAGAACAGGATTGTATTAATTTTAATACCTGG
PF3D7_         GAAGATTATTGTGAAGATAGGGCTACTAAATTGAGGGATTTAAAAGCCATAAAATATGCAGAACAGGATTGTATTAATTTTAATACCTGG
PfSD01         GAAGATTATTGTGAAGATAGGGCTACTAAATTGAGGGATTTAAAAGCCATAAAATATGCAGAACAGGATTGTATTAATTTTAATACCTGG
Pf7G8          GAAGATTATTGTGAAGATAGGGCTACTAAATTGAGGGATTTAAAAGCCATAAAATATGCAGAACAGGATTGTATTAATTTTAATACCTGG
FCR3 GENE      GAAGATTATTGTGAAGATAGGGCTACTAAATTGAGGGATTTAAAAGCCATAAAATATGCAGAACAGGATTGTATTAATTTTAATACCTGG
MS822 GENE     GAAGATTATTGTGAAGATAGGGCTACTAAATTGAGGGATTTAAAAGCCATAAAATATGCAGAACAGGATTGTATTAATTTTAATACCTGG
PfCD01_        GAAGATTATTGTGAAGATAGGGCTACTAAATTGAGGGATTTAAAAGCCATAAAATATGCAGAACAGGATTGTATTAATTTTAATACCTGG
PfKH01__       GAAGATTATTGTGAAGATAGGGCTACTAAATTGAGGGATTTAAAAGCCATAAAATATGCAGAACAGGATTGTATTAATTTTAATACCTGG
PfDd2__        GAAGATTATTGTGAAGATAGGGCTACTAAATTGAGGGATTTAAAAGCCATAAAATATGCAGAACAGGATTGTATTAATTTTAATACCTGG
PfSN01_        GAAGATTATTGTGAAGATAGGGCTACTAAATTGAGGGATTTAAAAGCCATAAAATATGCAGAACAGGATTGTATTAATTTTAATACCTGG
PfGN01         GAAGATTATTGTGAAGATAGGGCTACTAAATTGAGGGATTTAAAAGCCATAAAATATGCAGAACAGGATTGTATTAATTTTAATACCTGG
PfKE01         GAAGATTATTGTGAAGATAGGGCTACTAAATTGAGGGATTTAAAAGCCATAAAATATGCAGAACAGGATTGTATTAATTTTAATACCTGG
PfKH02         GAAGATTATTGTGAAGATAGGGCTACTAAATTGAGGGATTTAAAAGCCATAAAATATGCAGAACAGGATTGTATTAATTTTAATACCTGG
PfGB4          GAAGATTATTGTGAAGATAGGGCTACTAAATTGAGGGATTTAAAAGCCATAAAATATGCAGAACAGGATTGTATTAATTTTAATACCTGG
PfGA01_        GAAGATTATTGTGAAGATAGGGCTACTAAATTGAGGGATTTAAAAGCCATAAAATATGCAGAACAGGATTGTATTAATTTTAATACCTGG
PfHB3_         GAAGATTATTGTGAAGATAGGGCTACTAAATTGAGGGATTTAAAAGCCATAAAATATGCAGAACAGGATTGTATTAATTTTAATACCTGG
PfIT_          GAAGATTATTGTGAAGATAGGGCTACTAAATTGAGGGATTTAAAAGCCATAAAATATGCAGAACAGGATTGTATTAATTTTAATACCTGG
```

	460 470 480 490 500 510 520 530 540
PF3D7 CDS	GTAAGTAGTTGGGACTATGAAATAAAAAGGCAAATGAATAAATTAGATATTTCAAAAATAAAACAATATTTGGAAAAGTCAAAGTTCAAA
MS822 mRNA	GTAAGTAGTTGGGACTATGAAATAAAAAGGCAAATGAATAAATTAGATATTTCAAAAATAAAACAATATTTGGAAAAGTCAAAGTTCAAA
FCR3 mRNA	GTAAGTAGTTGGGACTATGAAATAAAAAGGCAAATGAATAAATTAGATATTTCAAAAATAAAACAATATTTGGAAAAGTCAAAGTTCAAA
AM1898	GTAAGTAGTTGGGACTATGAAATAAAAAGGCAAATGAATAAATTAGATATTTCAAAAATAAAACAATATTTGGAAAAGTCAAAGTTCAAA
AA235	GTAAGTAGTTGGGACTATGAAATAAAAAGGCAAATGAATAAATTAGATATTTCAAAAATAAAACAATATTTGGAAAAGTCAAAGTTCAAA
AA243	GTAAGTAGTTGGGACTATGAAATAAAAAGGCAAATGGATAAATTAGATATTTCAAAAATAAAACAATATTTGGAAAAGTCAAAGTTCAAA
AA258	GTAAGTAGTTGGGACTATGAAATAAAAAGGCAAATGAATAAATTAGATATTTCAAAAATAAAACAATATTTGGAAAAGTCAAAGTTCAAA
AA264	GTAAGTAGTTGGGACTATGAAATAAAAAGGCAAATGAATAAATTAGATATTTCAAAAATAAAACAATATTTGGAAAAGTCAAAGTTCAAA
AM1737	GTAAGTAGTTGGGACTATGAAATAAAAAGGCAAATGAATAAATTAGATATTTCAAAAATAAAACAATATTTGGAAAAGTCAAAGTTCAAA
SC74	GTAAGTAGTTGGGACTATGAAATAAAAAGGCAAATGAATAAATTAGATATTTCAAAAATAAAACAATATTTGGAAAAGTCAAAGTTCAAA
TAB166	GTAAGTAGTTGGGACTATGAAATAAAAAGGCAAATGAATAAATTAGATATTTCAAAAATAAAACAATATTTGGAAAAGTCAAAGTTCAAA
TAB138	GTAAGTAGTTGGGACTATGAAATAAAAAGGCAAATGAATAAATTAGATATTTCAAAAATAAAACAATATTTGGAAAAGTCAAAGTTCAAA
TAB130	GTAAGTAGTTGGGACTATGAAATAAAAAGGCAAATGAATAAATTAGATATTTCAAAAATAAAACAATATTTGGAAAAGTCAAAGTTCAAA
TAB131	GTAAGTAGTTGGGACTATGAAATAAAAAGGCAAATGAATAAATTAGATATTTCAAAAATAAAACAATATTTGGAAAAGTCAAAGTTCAAA
TAB151	GTAAGTAGTTGGGACTATGAAATAAAAAGGCAAATGAATAAATTAGATATTTCAAAAATAAAACAATATTTGGAAAAGTCAAAGTTCAAA
TAB152	GTAAGTAGTTGGGACTATGAAATAAAAAGGCAAATGAATAAATTAGATATTTCAAAAATAAAACAATATTTGGAAAAGTCAAAGTTCAAA
SC52	GTAAGTAGTTGGGACTATGAAATAAAAAGGCAAATGAATAAATTAGATATTTCAAAAATAAAACAATATTTGGAAAAGTCAAAGTTCAAA
SC55	GTAAGTAGTTGGGACTATGAAATAAAAAGGCAAATGAATAAATTAGATATTTCAAAAATAAAACAATATTTGGAAAAGTCAAAGTTCAAA
SC03	GTAAGTAGTTGGGACTATGAAATAAAAAGGCAAATGAATAAATTAGATATTTCAAAAATAAAACAATATTTGGAAAAGTCAAAGTTCAAA
SC53	GTAAGTAGTTGGGACTATGAAATAAAAAGGCAAATGAATAAATTAGATATTTCAAAAATAAAACAATATTTGGAAAAGTCAAAGTTCAAA
TAB141	GTAAGTAGTTGGGACTATGAAATAAAAAGGCAAATGAATAAATTAGATATTTCAAAAATAAAACAATATTTGGAAAAGTCAAAGTTCAAA
TAB123	GTAAGTAGTTGGGACTATGAAATAAAAAGGCAAATGAATAAATTAGATATTTCAAAAATAAAACAATATTTGGAAAAGTCAAAGTTCAAA
AM1803	GTAAGTAGTTGGGACTATGAAATAAAAAGGCAAATGGATAAATTAGATATTTCAAAAATAAAACAATATTTGGAAAAGTCAAAGTTCAAA
AM1814	GTAAGTAGTTGGGACTATGAAATAAAAAGGCAAATGGATAAATTAGATATTTCAAAAATAAAACAATATTTGGAAAAGTCAAAGTTCAAA
AM1802	GTAAGTAGTTGGGACTATGAAATAAAAAGGCAAATGAATAAATTAGATATTTCAAAAATAAAACAATATTTGGAAAAGTCAAAGTTCAAA
AM1811	GTAAGTAGTTGGGACTATGAAATAAAAAGGCAAATGGATAAATTAGATATTTCAAAAATAAAACAATATTTGGAAAAGTCAAAGTTCAAA
SC50	GTAAGTAGTTGGGACTATGAAATAAAAAGGCAAATGGATAAATTAGATATTTCAAAAATAAAACAATATTTGGAAAAGTCAAAGTTCAAA
SC56	GTAAGTAGTTGGGACTATGAAATAAAAAGGCAAATGGATAAATTAGATATTTCAAAAATAAAACAATATTTGGAAAAGTCAAAGTTCAAA
TAB153	GTAAGTAGTTGGGACTATGAAATAAAAAGGCAAATGGATAAATTAGATATTTCAAAAATAAAACAATATTTGGAAAAGTCAAAGTTCAAA
TAB154	GTAAGTAGTTGGGACTATGAAATAAAAAGGCAAATGAATAAATTAGATATTTCAAAAATAAAACAATATTTGGAAAAGTCAAAGTTCAAA
TAB156	GTAAGTAGTTGGGACTATGAAATAAAAAGGCAAATGGATAAATTAGATATTTCAAAAATAAAACAATATTTGGAAAAGTCAAAGTTCAAA
TAB136	GTAAGTAGTTGGGACTATGAAATAAAAAGGCAAATGGATAAATTAGATATTTCAAAAATAAAACAATATTTGGAAAAGTCAAAGTTCAAA
AM1880	GTAAGTAGTTGGGACTATGAAATAAAAAGGCAAATGGATAAATTAGATATTTCAAAAATAAAACAATATTTGGAAAAGTCAAAGTTCAAA
PF3D7_	GTAAGTAGTTGGGACTATGAAATAAAAAGGCAAATGAATAAATTAGATATTTCAAAAATAAAACAATATTTGGAAAAGTCAAAGTTCAAA
PfSD01	GTAAGTAGTTGGGACTATGAAATAAAAAGGCAAATGAATAAATTAGATATTTCAAAAATAAAACAATATTTGGAAAAGTCAAAGTTCAAA
Pf7G8	GTAAGTAGTTGGGACTATGAAATAAAAAGGCAAATGGATAAATTAGATATTTCAAAAATAAAACAATATTTGGAAAAGTCAAAGTTCAAA
FCR3 GENE	GTAAGTAGTTGGGACTATGAAATAAAAAGGCAAATGAATAAATTAGATATTTCAAAAATAAAACAATATTTGGAAAAGTCAAAGTTCAAA
MS822 GENE	GTAAGTAGTTGGGACTATGAAATAAAAAGGCAAATGAATAAATTAGATATTTCAAAAATAAAACAATATTTGGAAAAGTCAAAGTTCAAA
PfCD01_	GTAAGTAGTTGGGACTATGAAATAAAAAGGCAAATGAATAAATTAGATATTTCAAAAATAAAACAATATTTGGAAAAGTCAAAGTTCAAA
PfKH01__	GTAAGTAGTTGGGACTATGAAATAAAAAGGCAAATGAATAAATTAGATATTTCAAAAATAAAACAATATTTGGAAAAGTCAAAGTTCAAA
PfDd2__	GTAAGTAGTTGGGACTATGAAATAAAAAGGCAAATGAATAAATTAGATATTTCAAAAATAAAACAATATTTGGAAAAGTCAAAGTTCAAA
PfSN01_	GTAAGTAGTTGGGACTATGAAATAAAAAGGCAAATGGATAAATTAGATATTTCAAAAATAAAACAATATTTGGAAAAGTCAAAGTTCAAA
PfGN01	GTAAGTAGTTGGGACTATGAAATAAAAAGGCAAATGAATAAATTAGATATTTCAAAAATAAAACAATATTTGGAAAAGTCAAAGTTCAAA
PfKE01	GTAAGTAGTTGGGACTATGAAATAAAAAGGCAAATGAATAAATTAGATATTTCAAAAATAAAACAATATTTGGAAAAGTCAAAGTTCAAA
PfKH02	GTAAGTAGTTGGGACTATGAAATAAAAAGGCAAATGAATAAATTAGATATTTCAAAAATAAAACAATATTTGGAAAAGTCAAAGTTCAAA
PfGB4	GTAAGTAGTTGGGACTATGAAATAAAAAGGCAAATGAATAAATTAGATATTTCAAAAATAAAACAATATTTGGAAAAGTCAAAGTTCAAA
PfGA01_	GTAAGTAGTTGGGACTATGAAATAAAAAGGCAAATGAATAAATTAGATATTTCAAAAATAAAACAATATTTGGAAAAGTCAAAGTTCAAA
PfHB3_	GTAAGTAGTTGGGACTATGAAATAAAAAGGCAAATGGATAAATTAGATATTTCAAAAATAAAACAATATTTGGAAAAGTCAAAGTTCAAA
PfIT_	GTAAGTAGTTGGGACTATGAAATAAAAAGGCAAATGAATAAATTAGATATTTCAAAAATAAAACAATATTTGGAAAAGTCAAAGTTCAAA

```
                      550       560       570       580       590       600       610       620       630
             ....|....|....|....|....|....|....|....|....|....|....|....|....|....|....|....|....|....|
PF3D7 CDS    TGTGATATAAATGATTTAGATAATTTAGATACATTTTTTTCCCATGTAAATCCAAAAGATATGAAGGGAAGTGAAGAGGATCCAAGAAAT
MS822 mRNA   TGTGATATAAATGATTTAGATAATTTAGATACATTTTTTTCCCATGTAAATCCAAAAGATATGAAGGGAAGTGAAGAGGATCCAAGAAAT
FCR3 mRNA    TGTGATATAAATGATTTAGATAATTTAGATACATTTTTTTCCCATGTAAATCCAAAAGATATGAAGGGAAGTGAAGAGGATCCAAGAAAT
AM1898       TGTGATATAAATGATTTAGATAATTTAGATACATTTTTTTCCCATGTAAATCCAAAAGATATGAAGGGAAGTGAAGAGGATCCAAGAAAT
AA235        TGTGATATAAATGATTTAGATAATTTAGATACATTTTTTTCCCATGTAAATCCAAAAGATATGAAGGGAAGTGAAGAGGATCCAAGAAAT
AA243        TGTGATATAAATGTTTTAGATAATTTAGATACATTTTTTTCCCATGTAAATTCAAAAGATATGAAGGGAAGTGAAGAGGATCCAAGAAAT
AA258        TGTGATATAAATGATTTAGATAATTTAGATACATTTTTTTCCCATGTAAATCCAAAAGATATGAAGGGAAGTGAAGAGGATCCAAGAAAT
AA264        TGTGATATAAATGATTTAGATAATTTAGATACATTTTTTTCCCATGTAAATCCAAAAGATATGAAGGGAAGTGAAGAGGATCCAAGAAAT
AM1737       TGTGATATAAATGATTTAGATAATTTAGATACATTTTTTTCCCATGTAAATCCAAAAGATATGAAGGGAAGTGAAGAGGATCCAAGAAAT
SC74         TGTGATATAAATGATTTAGATAATTTAGATACATTTTTTTCCCATGTAAATCCAAAAGATATGAAGGGAAGTGAAGAGGATCCAAGAAAT
TAB166       TGTGATATAAATGATTTAGATAATTTAGATACATTTTTTTCCCATGTAAATCCAAAAGATATGAAGGGAAGTGAAGAGGATCCAAGAAAT
TAB138       TGTGATATAAATGATTTAGATAATTTAGATACATTTTTTTCCCATGTAAATCCAAAAGATATGAAGGGAAGTGAAGAGGATCCAAGAAAT
TAB130       TGTGATATAAATGATTTAGATAATTTAGATACATTTTTTTCCCATGTAAATCCAAAAGATATGAAGGGAAGTGAAGAGGATCCAAGAAAT
TAB131       TGTGATATAAATGATTTAGATAATTTAGATACATTTTTTTCCCATGTAAATCCAAAAGATATGAAGGGAAGTGAAGAGGATCCAAGAAAT
TAB151       TGTGATATAAATGATTTAGATAATTTAGATACATTTTTTTCCCATGTAAATCCAAAAGATATGAAGGGAAGTGAAGAGGATCCAAGAAAT
TAB152       TGTGATATAAATGATTTAGATAATTTAGATACATTTTTTTCCCATGTAAATCCAAAAGATATGAAGGGAAGTGAAGAGGATCCAAGAAAT
SC52         TGTGATATAAATGATTTAGATAATTTAGATACATTTTTTTCCCATGTAAATCCAAAAGATATGAAGGGAAGTGAAGAGGATCCAAGAAAT
SC55         TGTGATATAAATGATTTAGATAATTTAGATACATTTTTTTCCCATGTAAATCCAAAAGATATGAAGGGAAGTGAAGAGGATCCAAGAAAT
SC03         TGTGATATAAATGATTTAGATAATTTAGATACATTTTTTTCCCATGTAAATCCAAAAGATATGAAGGGAAGTGAAGAGGATCCAAGAAAT
SC53         TGTGATATAAATGATTTAGATAATTTAGATACATTTTTTTCCCATGTAAATCCAAAAGATATGAAGGGAAGTGAAGAGGATCCAAGAAAT
TAB141       TGTGATATAAATGATTTAGATAATTTAGATACATTTTTTTCCCATGTAAATCCAAAAGATATGAAGGGAAGTGAAGAGGATCCAAGAAAT
TAB123       TGTGATATAAATGATTTAGATAATTTAGATACATTTTTTTCCCATGTAAATCCAAAAGATATGAAGGGAAGTGAAGAGGATCCAAGAAAT
AM1803       TGTGATATAAATGATTTAGATAATTTAGATACATTTTTTTCCCATGTAAATTCAAAAGATATGAAGGGAAGTGAAGAGGATCCAAGAAAT
AM1814       TGTGATATAAATGTTTTAGATAATTTAGATACATTTTTTTCCCATGTAAATTCAAAAGATATGAAGGGAAGTGAAGAGGATCCAAGAAAT
AM1802       TGTGATATAAATGATTTAGATAATTTAGATACATTTTTTTCCCATGTAAATCCAAAAGATATGAAGGGAAGTGAAGAGGATCCAAGAAAT
AM1811       TGTGATATAAATGTTTTAGATAATTTAGATACATTTTTTTCCCATGTAAATTCAAAAGATATGAAGGGAAGTGAAGAGGATCCAAGAAAT
SC50         TGTGATATAAATGTTTTAGATAATTTAGATACATTTTTTTCCCATGTAAATTCAAAAGATATGAAGGGAAGTGAAGAGGATCCAAGAAAT
SC56         TGTGATATAAATGTTTTAGATAATTTAGATACATTTTTTTCCCATGTAAATTCAAAAGATATGAAGGGAAGTGAAGAGGATCCAAGAAAT
TAB153       TGTGATATAAATGTTTTAGATAATTTAGATACATTTTTTTCCCATGTAAATTCAAAAGATATGAAGGGAAGTGAAGAGGATCCAAGAAAT
TAB154       TGTGATATAAATGATTTAGATAATTTAGATACATTTTTTTCCCATGTAAATCCAAAAGATATGAAGGGAAGTGAAGAGGATCCAAGAAAT
TAB156       TGTGATATAAATGTTTTAGATAATTTAGATACATTTTTTTCCCATGTAAATTCAAAAGATATGAAGGGAAGTGAAGAGGATCCAAGAAAT
TAB136       TGTGATATAAATGTTTTAGATAATTTAGATACATTTTTTTCCCATGTAAATTCAAAAGATATGAAGGGAAGTGAAGAGGATCCAAGAAAT
AM1880       TGTGATATAAATGTTTTAGATAATTTAGATACATTTTTTTCCCATGTAAATTCAAAAGATATGAAGGGAAGTGAAGAGGATCCAAGAAAT
PF3D7_       TGTGATATAAATGATTTAGATAATTTAGATACATTTTTTTCCCATGTAAATCCAAAAGATATGAAGGGAAGTGAAGAGGATCCAAGAAAT
PfSD01       TGTGATATAAATGATTTAGATAATTTAGATACATTTTTTTCCCATGTAAATCCAAAAGATATGAAGGGAAGTGAAGAGGATCCAAGAAAT
Pf7G8        TGTGATATAAATGATTTAGATAATTTAGATACATTTTTTTCCCATGTAAATCCAAAAGATATGAAGGGAAGTGAAGAGGATCCAAGAAAT
FCR3 GENE    TGTGATATAAATGATTTAGATAATTTAGATACATTTTTTTCCCATGTAAATCCAAAAGATATGAAGGGAAGTGAAGAGGATCCAAGAAAT
MS822 GENE   TGTGATATAAATGATTTAGATAATTTAGATACATTTTTTTCCCATGTAAATCCAAAAGATATGAAGGGAAGTGAAGAGGATCCAAGAAAT
PfCD01_      TGTGATATAAATGTTTTAGATAATTTAGATACATTTTTTTCCCATGTAAATCCAAAAGATATGAAGGGAAGTGAAGAGGATCCAAGAAAT
PfKH01__     TGTGATATAAATGATTTAGATAATTTAGATACATTTTTTTCCCATGTAAATCCAAAAGATATGAAGGGAAGTGAAGAGGATCCAAGAAAT
PfDd2__      TGTGATATAAATGATTTAGATAATTTAGATACATTTTTTTCCCATGTAAATCCAAAAGATATGAAGGGAAGTGAAGAGGATCCAAGAAAT
PfSN01_      TGTGATATAAATGTTTTAGATAATTTAGATACATTTTTTTCCCATGTAAATCCAAAAGATATGAAGGGAAGTGAAGAGGATCCAAGAAAT
PfGN01       TGTGATATAAATGATTTAGATAATTTAGATACATTTTTTTCCCATGTAAATCCAAAAGATATGAAGGGAAGTGAAGAGGATCCAAGAAAT
PfKE01       TGTGATATAAATGATTTAGATAATTTAGATACATTTTTTTCCCATGTAAATCCAAAAGATATGAAGGGAAGTGAAGAGGATCCAAGAAAT
PfKH02       TGTGATATAAATGATTTAGATAATTTAGATACATTTTTTTCCCATGTAAATCCAAAAGATATGAAGGGAAGTGAAGAGGATCCAAGAAAT
PfGB4        TGTGATATAAATGATTTAGATAATTTAGATACATTTTTTTCCCATGTAAATCCAAAAGATATGAAGGGAAGTGAAGAGGATCCAAGAAAT
PfGA01_      TGTGATATAAATGATTTAGATAATTTAGATACATTTTTTTCCCATGTAAATCCAAAAGATATGAAGGGAAGTGAAGAGGATCCAAGAAAT
PfHB3_       TGTGATATAAATGTTTTAGATAATTTAGATACATTTTTTTCCCATGTAAATTCAAAAGATATGAAGGGAAGTGAAGAGGATCCAAGAAAT
PfIT_        TGTGATATAAATGATTTAGATAATTTAGATACATTTTTTTCCCATGTAAATCCAAAAGATATGAAGGGAAGTGAAGAGGATCCAAGAAAT
```

```
                   640       650       660       670       680       690       700       710       720
            ....|....|....|....|....|....|....|....|....|....|....|....|....|....|....|....|....|....|
PF3D7 CDS   CCTCAGGAAGAAGAGCACAAAAGAGATGAATATCAAGTTTTAAATAGTACAGTTGAAGAAGGTGATGAATATGTTGATAGAATAAATATC
MS822 mRNA  CCTCAGGAAGAAGAGCACAAAAGAGATGAATATCAAGTTTTAAATAGTACAGTTGAAGAAGGTGATGAATATGTTGATAGAATAAATATC
FCR3 mRNA   CCTCAGGAAGAAGAGCACAAAAGAGATGAATATCAAGTTTTAAATAGTACAGTTGAAGAAGGTGATGAATATGTTGATAGAATAAATATC
AM1898      CCTCAGGAAGAAGAGCACAAAAGAGATGAATATCAAGTTTTAAATAGTACAGTTGAAGAAGGTGATGAATATGCTGATAGAATAAATATC
AA235       CCTCAGGAAGAAGAGCACAAAAGAGATGAATATCAAGTTTTAAATAGTACAGTTGAAGAAGGTGATGAATATGCTGATAGAAAAAATATC
AA243       CCTCAGGAAGAAGAGCACAAAAGAGATGAATATCAAGTTTTAAATAGTACAGTTGAAGAAGGTGATGAATATGCTGATAGAAAAAATATC
AA258       CCTCAGGAAGAAGAGCACAAAAGAGATGAATATCAAGTTTTAAATAGTACAGTTGAAGAAGGTGATGAATATGCTGATAGAAAAAATATC
AA264       CCTCAGGAAGAAGAGCACAAAAGAGATGAATATCAAGTTTTAAATAGTACAGTTGAAGAAGGTGATGAATATGTTGATAGAATAAATATC
AM1737      CCTCAGGAAGAAGAGCACAAAAGAGATGAATATCAAGTTTTAAATAGTACAGTTGAAGAAGGTGATGAATATGCTGATAGAATAAATATC
SC74        CCTCAGGAAGAAGAGCACAAAAGAGATGAATATCAAGTTTTAAATAGTACAGTTGAAGAAGGTGATGAATATGTTGATAGAATAAATATC
TAB166      CCTCAGGAAGAAGAGCACAAAAGAGATGAATATCAAGTTTTAAATAGTACAGTTGAAGAAGGTGATGAATATGCTGATAGAAAAAATATC
TAB138      CCTCAGGAAGAAGAGCACAAAAGAGATGAATATCAAGTTTTAAATAGTACAGTTGAAGAAGGTGATGAATATGTTGATAGAATAAATATC
TAB130      CCTCAGGAAGAAGAGCACAAAAGAGATGAATATCAAGTTTTAAATAGTACAGTTGAAGAAGGTGATGAATATGTTGATAGAATAAATATC
TAB131      CCTCAGGAAGAAGAGCACAAAAGAGATGAATATCAAGTTTTAAATAGTACAGTTGAAGAAGGTGATGAATATGCTGATAGAAAAAATATC
TAB151      CCTCAGGAAGAAGAGCACAAAAGAGATGAATATCAAGTTTTAAATAGTACAGTTGAAGAAGGTGATGAATATGCTGATAGAAAAAATATC
TAB152      CCTCAGGAAGAAGAGCACAAAAGAGATGAATATCAAGTTTTAAATAGTACAGTTGAAGAAGGTGATGAATATGTTGATAGAATAAATATC
SC52        CCTCAGGAAGAAGAGCACAAAAGAGATGAATATCAAGTTTTAAATAGTACAGTTGAAGAAGGTGATGAATATGCTGATAGAAAAAATATC
SC55        ACTCAGGAAGAAGAGCACAAAAGAGATGAATATCAAGTTTTAAATAGTACAGTTGAAGAAGGTGATGAATATGCTGATAGAATAAATATC
SC03        CCTCAGGAAGAAGAGCACAAAAGAGATGAATATCAAGTTTTAAATAGTACAGTTGAAGAAGGTGATGAATATGCTGATAGAAAAAATATC
SC53        CCTCAGGAAGAAGAGCACAAAAGAGATGAATATCAAGTTTTAAATAGTACAGTTGAAGAAGGTGATGAATATGTTGATAGAATAAATATC
TAB141      CCTCAGGAAGAAGAGCACAAAAGAGATGAATATCAAGTTTTAAATAGTACAGTTGAAGAAGGTGATGAATATGTTGATAGAATAAATATC
TAB123      CCTCAGGAAGAAGAGCACAAAAGAGATGAATATCAAGTTTTAAATAGTACAGTTGAAGAAGGTGATGAATATGTTGATAGAATAAATATC
AM1803      ACTCAGGAAGAAGAGCACAAAAGAGATGAATATCAAGTTTTAAATAGTACAGTTGAAGAAGGTGATGAATATGCTGATAGAATAAATATC
AM1814      ACTCAGGAAGAAGAGCACAAAAGAGATGAATATCAAGTTTTAAATAGTACAGTTGAAGAAGGTGATGAATATGCTGATAGAATAAATATC
AM1802      CCTCAGGAAGAAGAGCACAAAAGAGATGAATATCAAGTTTTAAATAGTACAGTTGAAGAAGGTGATGAATATGCTGATAGAAAAAATATC
AM1811      ACTCAGGAAGAAGAGCACAAAAGAGATGAATATCAAGTTTTAAATAGTACAGTTGAAGAAGGTGATGAATATGCTGATAGAATAAATATC
SC50        ACTCAGGAAGAAGAGCACAAAAGAGATGAATATCAAGTTTTAAATAGTACAGTTGAAGAAGGTGATGAATATGCTGATAGAAAAAATATC
SC56        ACTCAGGAAGAAGAGCACAAAAGAGATGAATATCAAGTTTTAAATAGTACAGTTGAAGAAGGTGATGAATATGCTGATAGAATAAATATC
TAB153      ACTCAGGAAGAAGAGCACAAAAGAGATGAATATCAAGTTTTAAATAGTACAGTTGAAGAAGGTGATGAATATGCTGATAGAATAAATATC
TAB154      CCTCAGGAAGAAGAGCACAAAAGAGATGAATATCAAGTTTTAAATAGTACAGTTGAAGAAGGTGATGAATATGCTGATAGAATAAATATC
TAB156      ACTCAGGAAGAAGAGCACAAAAGAGATGAATATCAAGTTTTAAATAGTACAGTTGAAGAAGGTGATGAATATGCTGATAGAAAAAATATC
TAB136      ACTCAGGAAGAAGAGCACAAAAGAGATGAATATCAAGTTTTAAATAGTACAGTTGAAGAAGGTGATGAATATGCTGATAGAATAAATATC
AM1880      ACTCAGGAAGAAGAGCACAAAAGAGATGAATATCAAGTTTTAAATAGTACAGTTGAAGAAGGTGATGAATATGCTGATAGAATAAATATC
PF3D7_      CCTCAGGAAGAAGAGCACAAAAGAGATGAATATCAAGTTTTAAATAGTACAGTTGAAGAAGGTGATGAATATGTTGATAGAATAAATATC
PfSD01      CCTCAGGAAGAAGAGCACAAAAGAGATGAATATCAAGTTTTAAATAGTACAGTTGAAGAAGGTGATGAATATGTTGATAGAATAAATATC
Pf7G8       CCTCAGGAAGAAGAGCACAAAAGAGATGAATATCAAGTTTTAAATAGTACAGTTGAAGAAGGTGATGAATATGCTGATAGAAAAAATATG
FCR3 GENE   CCTCAGGAAGAAGAGCACAAAAGAGATGAATATCAAGTTTTAAATAGTACAGTTGAAGAAGGTGATGAATATGTTGATAGAATAAATATC
MS822 GENE  CCTCAGGAAGAAGAGCACAAAAGAGATGAATATCAAGTTTTAAATAGTACAGTTGAAGAAGGTGATGAATATGTTGATAGAATAAATATC
PfCD01_     CCTCAGGAAGAAGAGCACAAAAGAGATGAATATCAAGTTTTAAATAGTACAGTTGAAGAAGGTGATGAATATGCTGATAGAAAAAATATC
PfKH01__    CCTCAGGAAGAAGAGCACAAAAGAGATGAATATCAAGTTTTAAATAGTACAGTTGAAGAAGGTGATGAATATGCTGATAGAAAAAATATC
PfDd2__     CCTCAGGAAGAAGAGCACAAAAGAGATGAATATCAAGTTTTAAATAGTACAGTTGAAGAAGGTGATGAATATGCTGATAGAAAAAATATC
PfSN01_     ACTCAGGAAGAAGAGCACAAAAGAGATGAATATCAAGTTTTAAATAGTACAGTTGAAGAAGGTGATGAATATGCTGATAGAAAAAATATG
PfGN01      CCTCAGGAAGAAGAGCACAAAAGAGATGAATATCAAGTTTTAAATAGTACAGTTGAAGAAGGTGATGAATATGTTGATAGAATAAATATC
PfKE01      CCTCAGGAAGAAGAGCACAAAAGAGATGAATATCAAGTTTTAAATAGTACAGTTGAAGAAGGTGATGAATATGTTGATAGAATAAATATC
PfKH02      CCTCAGGAAGAAGAGCACAAAAGAGATGAATATCAAGTTTTAAATAGTACAGTTGAAGAAGGTGATGAATATGCTGATAGAAAAAATATC
PfGB4       CCTCAGGAAGAAGAGCACAAAAGAGATGAATATCAAGTTTTAAATAGTACAGTTGAAGAAGGTGATGAATATGTTGATAGAATAAATATC
PfGA01_     CCTCAGGAAGAAGAGCACAAAAGAGATGAATATCAAGTTTTAAATAGTACAGTTGAAGAAGGTGATGAATATGTTGATAGAATAAATATC
PfHB3_      ACTCAGGAAGAAGAGCACAAAAGAGATGAATATCAAGTTTTAAATAGTACAGTTGAAGAAGGTGATGAATATGCTGATAGAATAAATATC
PfIT_       CCTCAGGAAGAAGAGCACAAAAGAGATGAATATCAAGTTTTAAATAGTACAGTTGAAGAAGGTGATGAATATGTTGATAGAATAAATATC
```

```
                     730       740       750       760       770       780       790       800       810
            ....|....|....|....|....|....|....|....|....|....|....|....|....|....|....|....|....|....|
PF3D7 CDS   CTTTCTACAGAAACAGGCGAAGCGACACCAAATATTCAAGAAGAACCTTTAAGGGTTACTCCTCATGGAGGGGATAATCATGTTAAAACA
MS822 mRNA  CTTTCTACAGAAACAGGCGAAGCGACACCAAATATTCAAGAAGAACCTTTAAGGGTTACTCCTCATGGAGGGGATAATCATGTTAAAACA
FCR3 mRNA   CCTTCTACAGAAACAGGCGAAGCGACACCAAATATTCAAGAAGAACCTTTAAGGGTTACTCCTCATGGAGGGGATGATCATGTTAAAACA
AM1898      CCTTCTATAGAAACAGGCGAAGAGACACCAAATGTTCAAGAAGAACCTTTAAGGGTTACTCCTCATGGAGGGGATGATCATGTTAAAACA
AA235       CCTTCTATAGAAACAGGCGAAGAGACACCAAATGTTCAAGAAGAACCTTTAAGGGTTACTCCTCATGGAGGGGATGATCATGTTAAAACA
AA243       CCTTCTATAGAAACAGGCGAAGAGACACCAAATGTTCAAGAAGAACCTTTAAGGGTTACTCCTCATGGAGGGGATGATCATGTTAAAACA
AA258       CCTTCTATAGAAACAGGCGAAGAGACACCAAATGTTCAAGAAGAACCTTTAAGGGTTACTCCTCATGGAGGGGATGATCATGTTAAAACA
AA264       CCTTCTACAGAAACAGGCGAAGCGACACCAAATATTCAAGAAGAACCTTTAAGGGTTACTCCTCATGGAGGGGATGATCATGTTAAAACA
AM1737      CCTTCTATAGAAACAGGCGAAGAGACACCAAATGTTCAAGAAGAACCTTTAAGGGTTACTCCTCATGGAGGGGATAATCATGTTAAAACA
SC74        CCTTCTACAGAAACAGGCGAAGCGACACCAAATATTCAAGAAGAACCTTTAAGGGTTACTCCTCATGGAGGGGATAATCATGTTAAAACA
TAB166      CCTTCTATAGAAACAGGCGAAGAGACACCAAATGTTCAAGAAGAACCTTTAAGGGTTACTCCTCATGGAGGGGATGATCATGTTAAAACA
TAB138      CCTTCTACAGAAACAGGCGAAGCGACACCAAATATTCAAGAAGAACCTTTAAGGGTTACTCCTCATGGAGGGGATGATCATGTTAAAACA
TAB130      CCTTCTACAGAAACAGGCGAAGCGACACCAAATATTCAAGAAGAACCTTTAAGGGTTACTCCTCATGGAGGGGATAATCATGTTAAAACA
TAB131      CCTTCTATAGAAACAGGCGAAGAGACACCAAATGTTCAAGAAGAACCTTTAAGGGTTACTCCTCATGGAGGGGATGATCATGTTAAAACA
TAB151      CCTTCTATAGAAACAGGCGAAGAGACACCAAATGTTCAAGAAGAACCTTTAAGGGTTACTCCTCATGGAGGGGATGATCATGTTAAAACA
TAB152      CTTTCTACAGAAACAGGCGAAGCGACACCAAATATTCAAGAAGAACCTTTAAGGGTTACTCCTCATGGAGGGGATAATCATGTTAAAACA
SC52        CCTTCTATAGAAACAGGCGAAGAGACACCAAATGTTCAAGAAGAACCTTTAAGGGTTACTCCTCATGGAGGGGATAATCATGTTAAAACA
SC55        CCTTCTATAGAAACAGGCGAAGAGACACCAAATGTTCAAGAAGAACCTTTAAGGGTTACTCCTCATGGAGGGGATGATCATGTTAAAACA
SC03        CCTTCTATAGAAACAGGCGAAGAGACACCAAATGTTCAAGAAGAACCTTTAAGGGTTACTCCTCATGGAGGGGATGATCATGTTAAAACA
SC53        CTTTCTACAGAAACAGGCGAAGCGACACCAAATATTCAAGAAGAACCTTTAAGGGTTACTCCTCATGGAGGGGATAATCATGTTAAAACA
TAB141      CCTTCTACAGAAACAGGCGAAGCGACACCAAATATTCAAGAAGAACCTTTAAGGGTTACTCCTCATGGAGGGGATAATCATGTTAAAACA
TAB123      CCTTCTACAGAAACAGGCGAAGCGACACCAAATATTCAAGAAGAACCTTTAAGGGTTACTCCTCATGGAGGGGATAATCATGTTAAAACA
AM1803      CCTTCTATAGAAACAGGCGAAGAGACACCAAATGTTCAAGAAGAACCTTTAAGGGTTACTCCTCATGGAGGGGATGATCATGTTAAAACA
AM1814      CCTTCTATAGAAACAGGCGAAGAGACACCAAATGTTCAAGAAGAACCTTTAAGGGTTACTCCTCATGGAGGGGATGATCATGTTAAAACA
AM1802      CCTTCTATAGAAACAGGCGAAGAGACACCAAATATTCAAGAAGAACCTTTAAGGGTTACTCCTCATGGAGGGGATGATCATGTTAAAACA
AM1811      CCTTCTATAGAAACAGGCGAAGAGACACCAAATGTTCAAGAAGAACCTTTAAGGGTTACTCCTCATGGAGGGGATGATCATGTTAAAACA
SC50        CCTTCTATAGAAACAGGCGAAGAGACACCAAATATTCAAGAAGAACCTTTAAGGGTTACTCCTCATGGAGGGGATGATCATGTTAAAACA
SC56        CCTTCTATAGAAACAGGCGAAGAGACACCAAATGTTCAAGAAGAACCTTTAAGGGTTACTCCTCATGGAGGGGATGATCATGTTAAAACA
TAB153      CCTTCTATAGAAACAGGCGAAGCGACACCAAATATTCAAGAAGAACCTTTAAGGGTTACTCCTCATGGAGGGGATAATCATGTTAAAACA
TAB154      CCTTCTATAGAAACAGGCGAAGAGACACCAAATGTTCAAGAAGAACCTTTAAGGGTTACTCCTCATGGAGGGGATGATCATGTTAAAACA
TAB156      CCTTCTATAGAAACAGGCGAAGAGACACCAAATATTCAAGAAGAACCTTTAAGGGTTACTCCTCATGGAGGGGATGATCATGTTAAAACA
TAB136      CCTTCTATAGAAACAGGCGAAGAGACACCAAATGTTCAAGAAGAACCTTTAAGGGTTACTCCTCATGGAGGGGATGATCATGTTAAAACA
AM1880      CCTTCTATAGAAACAGGCGAAGAGACACCAAATGTTCAAGAAGAACCTTTAAGGGTTACTCCTCATGGAGGGGATGATCATGTTAAAACA
PF3D7_      CTTTCTACAGAAACAGGCGAAGCGACACCAAATATTCAAGAAGAACCTTTAAGGGTTACTCCTCATGGAGGGGATAATCATGTTAAAACA
PfSD01      CTTTCTACAGAAACAGGCGAAGCGACACCAAATATTCAAGAAGAACCTTTAAGGGTTACTCCTCATGGAGGGGATAATCATGTTAAAACA
Pf7G8       CCTTCTATAGAAACAGGCGAAGAGACACCAAATATTCAAGAAGAACCTTTAAGGGTTACTCCTCATGGAGGGGATGATCATGTTAAAACA
FCR3 GENE   CCTTCTACAGAAACAGGCGAAGCGACACCAAATATTCAAGAAGAACCTTTAAGGGTTACTCCTCATGGAGGGGATGATCATGTTAAAACA
MS822 GENE  CTTTCTACAGAAACAGGCGAAGCGACACCAAATATTCAAGAAGAACCTTTAAGGGTTACTCCTCATGGAGGGGATAATCATGTTAAAACA
PfCD01_     CCTTCTATAGAAACAGGCGAAGAGACACCAAATGTTCAAGAAGAACCTTTAAGGGTTACTCCTCATGGAGGGGATGATCATGTTAAAACA
PfKH01__    CCTTCTATAGAAACAGGCGAAGAGACACCAAATGTTCAAGAAGAACCTTTAAGGGTTACTCCTCATGGAGGGGATGATCATGTTAAAACA
PfDd2__     CCTTCTATAGAAACAGGCGAAGAGACACCAAATGTTCAAGAAGAACCTTTAAGGGTTACTCCTCATGGAGGGGATGATCATGTTAAAACA
PfSN01_     CCTTCTATAGAAACAGGCGAAGCGACACCAAATATTCAAGAAGAACCTTTAAGGGTTACTCCTCATGGAGGGGATAATCATGTTAAAACA
PfGN01      CTTTCTACAGAAACAGGCGAAGCGACACCAAATATTCAAGAAGAACCTTTAAGGGTTACTCCTCATGGAGGGGATAATCATGTTAAAACA
PfKE01      CTTTCTACAGAAACAGGCGAAGCGACACCAAATATTCAAGAAGAACCTTTAAGGGTTACTCCTCATGGAGGGGATAATCATGTTAAAACA
PfKH02      CCTTCTATAGAAACAGGCGAAGAGACACCAAATGTTCAAGAAGAACCTTTAAGGGTTACTCCTCATGGAGGGGATGATCATGTTAAAACA
PfGB4       CTTTCTACAGAAACAGGCGAAGCGACACCAAATATTCAAGAAGAACCTTTAAGGGTTACTCCTCATGGAGGGGATAATCATGTTAAAACA
PfGA01_     CTTTCTACAGAAACAGGCGAAGCGACACCAAATATTCAAGAAGAACCTTTAAGGGTTACTCCTCATGGAGGGGATAATCATGTTAAAACA
PfHB3_      CCTTCTATAGAAACAGGCGAAGAGACACCAAATGTTCAAGAAGAACCTTTAAGGGTTACTCCTCATGGAGGGGATGATCATGTTAAAACA
PfIT_       CCTTCTACAGAAACAGGCGAAGCGACACCAAATATTCAAGAAGAACCTTTAAGGGTTACTCCTCATGGAGGGGATGATCATGTTAAAACA
```

```
                  820       830       840       850       860       870       880       890       900
              ....|....|....|....|....|....|....|....|....|....|....|....|....|....|....|....|....|....|
PF3D7 CDS     CCTCCTACAATTGTAAAGACTGAAGTAGGTAATGATAATCGAGAGAATGTAGTTTCTACTGCTGGTAAAAGATTAAATAGAGGTAAACCA
MS822 mRNA    CCTCCTACAATTGTAAAGACTGAAGTAGGTAATGATAATCGAGAGAATGTAGTTTCTACTGCTGGTAAAAGATTAAATAGAGGTAAACCA
FCR3 mRNA     CCTCCTACAATTGTAAAGACTGAAGAAGGTAATGATAATCGAGAGAATGTAGTTTCTACTGCTGGTAAAAGATTAAATAGAGGTAAACCA
AM1898        CCTCCTACAATTGTAAAGACTGAAGTAGGTAATGATAATCGAGAGAATGTAGTTTCTACTGCTGGTAAAAGATTAAATAAAGGTAAAGTA
AA235         CCTCCTACAATTGTAAAGACTGAAGTAGGTAATGATAATCGAGAGAATGTAGTTTCTACTGCTGGTAAAAGATTAAATAGAGGTAAAGCA
AA243         CCTCCTACAATTGTAAAGACTGAAGTAGGTAATGATAATCGAGAGAATGTAGTTTCTACTGCTGGTAAAAGATTAAATAGAGGTAAAGCA
AA258         CCTCCTACAATTGTAAAGACTGAAGTAGGTAATGATAATCGAGAGAATGTAGTTTCTACTGCTGGTAAAAGATTAAATAGAGGTAAAGCA
AA264         CCTCCTACAATTGTAAAGACTGAAGAAGGTAATGATAATCGAGAGAATGTAGTTTCTACTGCTGGTAAAAGATTAAATAGAGGTAAACCA
AM1737        CCTCCTACAATTGTAAAGACTGAAGTAGGTAATGATAATCGAGAGAATGTAGTTTCTACTGCTGGTAAAAGATTAAATAGAGGTAAAGCA
SC74          CCTCCTACAATTGTAAAGACTGAAGTAGGTAATGATAATCGAGAGAATGTAGTTTCTACTGCTGGTAAAAGATTAAATAGAGGTAAAGCA
TAB166        CCTCCTACAATTGTAAAGACTGAAGTAGGTAATGATAATCGAGAGAATGTAGTTTCTACTGCTGGTAAAAGATTAAATAGAGGTAAAGCA
TAB138        CCTCCTACAATTGTAAAGACTGAAGAAGGTAATGATAATCGAGAGAATGTAGTTTCTACTGCTGGTAAAAGATTAAATAGAGGTAAACCA
TAB130        CCTCCTACAATTGTAAAGACTGAAGTAGGTAATGATAATCGAGAGAATGTAGTTTCTACTGCTGGTAAAAGATTAAATAGAGGTAAAGCA
TAB131        TCTCCTACAATTGTAAAGACTGAAGTAGGTAATGATAATCGAGAGAATGTAGTTTCTACTGCTGGTAAAAGATTAAATAGAGGTAAAGCA
TAB151        TCTCCTACAATTGTAAAGACTGAAGTAGGTAATGATAATCGAGAGAATGTAGTTTCTACTGCTGGTAAAAGATTAAATAGAGGTAAAGCA
TAB152        CCTCCTACAATTGTAAAGACTGAAGTAGGTAATGATAATCGAGAGAATGTAGTTTCTACTGCTGGTAAAAGATTAAATAGAGGTAAACCA
SC52          CCTCCTACAATTGTAAAGACTGAAGTAGGTAATGATAATCGAGAGAATGTAGTTTCTACTGCTGGTAAAAGATTAAATAGAGGTAAAGCA
SC55          CCTCCTACAATTGTAAAGACTGAAGTAGGTAATGATAATCGAGAGAATGTAGTTTCTACTGCTGGTAAAAGATTAAATAGAGGTAAAGCA
SC03          CCTCCTACAATTGTAAAGACTGAAGTAGGTAATGATAATCGAGAGAATGTAGTTTCTACTGCTGGTAAAAGATTAAATAGAGGTAAAGCA
SC53          CCTCCTACAATTGTAAAGACTGAAGTAGGTAATGATAATCGAGAGAATGTAGTTTCTACTGCTGGTAAAAGATTAAATAGAGGTAAACCA
TAB141        CCTCCTACAATTGTAAAGACTGAAGTAGGTAATGATAATCGAGAGAATGTAGTTTCTACTGCTGGTAAAAGATTAAATAGAGGTAAAGCA
TAB123        CCTCCTACAATTGTAAAGACTGAAGTAGGTAATGGTAATCGAGAGAATGTAGTTTCTACTGCTGGTAAAAGATTAAATAGAGGTAAAGCA
AM1803        TCTCCTATAATTGTAAAGACTGAAGAAGGTAATGATAATCGAGAGAATGTAGTTTCTACTGCTGGTAAAAGATTAAATGGAGGTAAACTA
AM1814        CCTCCTACAATTGTAAAGACTGAAGTAGGTAATGATAATCGAGAGAATGTAGTTTCTACTGCTGGTAAAAGATTAAATAGAGGTAAACCA
AM1802        CCTCCTACAATTGTAAAGACTGAAGAAGGTAATGATAATCGAGAGAATGTAGTTTCTACTGCTGGTAAAAGATTAAATAGAGGTAAACCA
AM1811        TCTCCTATAATTGTAAAGACTGAAGAAGGTAATGATAATCGAGAGAATGTAGTTTCTACTGCTGGTAAAAGATTAAATGGAGGTAAACTA
SC50          CCTCCTACAATTGTAAAGACTGAAGAAGGTAATGATAATCGAGAGAATGTAGTTTCTACTGCTGGTAAAAGATTAAATAGAGGTAAACCA
SC56          TCTCCTATAATTGTAAAGACTGAAGAAGGTAATGATAATCGAGAGAATGTAGTTTCTACTGCTGGTAAAAGATTAAATGGAGGTAAACTA
TAB153        CCTCCTACAATTGTAAAGACTGAAGTAGGTAATGATAATCGAGAGAATGTAGTTTCTACTGCTGGTAAAAGATTAAATAGAGGTAAAGCA
TAB154        CCTCCTACAATTGTAAAGACTGAAGTAGGTAATGATAATCGAGAGAATGTAGTTTCTACTGCTGGTAAAAGATTAAATAGAGGTAAAGCA
TAB156        CCTCCTACAATTGTAAAGACTGAAGAAGGTAATGATAATCGAGAGAATGTAGTTTCTACTGCTGGTAAAAGATTAAATAGAGGTAAACCA
TAB136        CCTCCTACAATTGTAAAGACTGAAGTAGGTAATGATAATCGAGAGAATGTAGTTTCTACTGCTGGTAAAAGATTAAATAAAGGTAAACTA
AM1880        CCTCCTACAATTGTAAAGACTGAAGTAGGTAATGATAATCGAGAGAATGTAGTTTCTACTGCTGGTAAAAGATTAAATAAAGGTAAACTA
PF3D7_        CCTCCTACAATTGTAAAGACTGAAGTAGGTAATGATAATCGAGAGAATGTAGTTTCTACTGCTGGTAAAAGATTAAATAGAGGTAAACCA
PfSD01        CCTCCTACAATTGTAAAGACTGAAGTAGGTAATGATAATCGAGAGAATGTAGTTTCTACTGCTGGTAAAAGATTAAATAGAGGTAAAGCA
Pf7G8         CCTCCTACAATTGTAAAGACTGAAGTAGGTAATGATAATCGAGAGAATGTAGTTTCTACTGCTGGTAAAAGATTAAATAAAGGTAAACTA
FCR3 GENE     CCTCCTACAATTGTAAAGACTGAAGAAGGTAATGATAATCGAGAGAATGTAGTTTCTACTGCTGGTAAAAGATTAAATAGAGGTAAACCA
MS822 GENE    CCTCCTACAATTGTAAAGACTGAAGTAGGTAATGATAATCGAGAGAATGTAGTTTCTACTGCTGGTAAAAGATTAAATAGAGGTAAACCA
PfCD01_       TCTCCTATAATTGTAAAGACTGAAGAAGGTAATGATAATCGAGAGAATGTAGTTTCTACTGCTGGTAAAAGATTAAATGGAGGTAAACTA
PfKH01__      TCTCCTACAATTGTAAAGACTGAAGTAGGTAATGATAATCGAGAGAATGTAGTTTCTACTGCTGGTAAAAGATTAAATAGAGGTAAAGCA
PfDd2__       CCTCCTACAATTGTAAAGACTGAAGTAGGTAATGATAATCGAGAGAATGTAGTTTCTACTGCTGGTAAAAGATTAAATAGAGGTAAAGCA
PfSN01_       CCTCCTACAATTGTAAAGACTGAAGTAGGTAATGATAATCGAGAGAATGTAGTTTCTACTGCTGGTAAAAGATTAAATAGAGGTAAAGCA
PfGN01        CCTCCTACAATTGTAAAGACTGAAGTAGGTAATGATAATCGAGAGAATGTAGTTTCTACTGCTGGTAAAAGATTAAATAGAGGTAAACCA
PfKE01        CCTCCTACAATTGTAAAGACTGAAGTAGGTAATGATAATCGAGAGAATGTAGTTTCTACTGCTGGTAAAAGATTAAATAGAGGTAAACCA
PfKH02        CCTCCTACAATTGTAAAGACTGAAGTAGGTAATGATAATCGAGAGAATGTAGTTTCTACTGCTGGTAAAAGATTAAATAGAGGTAAAGCA
PfGB4         CCTCCTACAATTGTAAAGACTGAAGTAGGTAATGATAATCGAGAGAATGTAGTTTCTACTGCTGGTAAAAGATTAAATAGAGGTAAACCA
PfGA01_       CCTCCTACAATTGTAAAGACTGAAGTAGGTAATGATAATCGAGAGAATGTAGTTTCTACTGCTGGTAAAAGATTAAATAGAGGTAAAGCA
PfHB3_        CCTCCTACAATTGTAAAGACTGAAGTAGGTAATGATAATCGAGAGAATGTAGTTTCTACTGCTGGTAAAAGATTAAATAAAGGTAAACTA
PfIT_         CCTCCTACAATTGTAAAGACTGAAGAAGGTAATGATAATCGAGAGAATGTAGTTTCTACTGCTGGTAAAAGATTAAATAGAGGTAAACCA
```

```
                  910       920       930       940       950       960       970       980       990
          ....|....|....|....|....|....|....|....|....|....|....|....|....|....|....|....|....|....|
PF3D7 CDS   AATAAAGATGGGGTTCCACATACATCTAGATTTAAAACACCTAAAGTTAGTGCACCTGCTGGAGGGAAAAAATCTAAAAATGCAAGAGAT
MS822 mRNA  AATAAAGATGGGGTTCCACATACATCTAGATTTAAAACACCTAAAGTTAGTGCACCTGCTGGAGGGAAAAAATCTAAAAATGCAAGAGAT
FCR3 mRNA   AATAAAGAGGGGGGTCCACATACATCTAGATTTAAAACACCTAAAGTTAGTGCACCTGCTGGAGGGAAAAAATCTAAAAATGCAAGAGAT
AM1898      AATAAAGATGGGGTTCCACATACAACTAAGTTTAAAACACCTAAAGTGAGTGCATCTGCTGAAGGGAAAAAATCTAAAAATGCAAGAGAT
AA235       AATAAAGATGGGGTTCCACATACAACTAAGTTTAAAACACCTAAAGTGAGGGCACCTGCTGAAGGGAAAAAATCTAAAAATGCAAGAGAT
AA243       AATAAAGATGGGGTTCCACATACAACTAAGTTTAAAACACCTAAAGTGAGGGCACCTGCTGAAGGGAAAAAATCTAAAAATGCAAGAGAT
AA258       AATAAAGATGGGGTTCCACATACAACTAAGTTTAAAACACCTAAAGTGAGGGCACCTGCTGAAGGGAAAAAATCTAAAAATGCAAGAGAT
AA264       AATAAAGAGGGGGGTCCACATACAACTAAGTTTAAAACACCTAAAGTTAGTGCACCTGCTGGAGGGAAAAAATCTAAAAATGCAAGAGAT
AM1737      AATAAAGATGGGGTTCCACATACAACTAAGTTTAAAACACCTAAAGTGAGGGCACCTGCTGAAGGGAAAAAATCTAAAAATGCAAGAGAT
SC74        AATAAAGATGGGGTTCCACATACAACTAAGTTTAAAACACCTAAAGTGAGGGCACCTGCTGAAGGGAAAAAATCTAAAAATGCAAGAGAT
TAB166      AATAAAGATGGGGTTCCACATACAACTAAGTTTAAAACACCTAAAGTGAGGGCACCTGCTGAAGGGAAAAAATCTAAAAATGCAAGAGAT
TAB138      AATAAAGAGGGGGTTCCACATACAACTAAGTTTAAAACACCTAAAGTGAGGGCACCTGCTGAAGGGAAAAAATCTAAAAATGCAAGAGAT
TAB130      AATAAAGATGGGGTTCCACATACAACTAAGTTTAAAACACCTAAAGTGAGGGCACCTGCTGAAGGGAAAAAATCTAAAAATGCAAGAGAT
TAB131      AATAAAGATGGGGTTCCACATACAACTAAGTTTAAAACACCTAAAGTGAGGGCACCTGCTGAAGGGAAAAAATCTAAAAATGCAAGAGAT
TAB151      AATAAAGATGGGGTTCCACATACAACTAAGTTTAAAACACCTAAAGTGAGGGCACCTGCTGAAGGGAAAAAATCTAAAAATGCAAGAGAT
TAB152      AATAAAGATGGGGTTCCACATACAACTAAGTTTAAAACACCTAAAGTGAGGGCACCTGCTGAAGGGAAAAAATCTAAAAATGCAAGAGAT
SC52        AATAAAGATGGGGTTCCACATACAACTAAGTTTAAAACACCTAAAGTGAGGGCACCTGCTGAAGGGAAAAAATCTAAAAATGCAAGAGAT
SC55        AATAAAGATGGGGTTCCACATACAACTAAGTTTAAAACACCTAAAGTGAGTGCATCTGCTGAAGGGAAAAAATCTAAAAATGCAAGAGAT
SC03        AATAAAGATGGGGTTCCACATACAACTAAGTTTAAAACACCTAAAGTGAGGGCACCTGCTGAAGGGAAAAAATCTAAAAATGCAAGAGAT
SC53        AATAAAGATGGGGTTCCACATACAACTAAGTTTAAAACACCTAAAGTGAGGGCACCTGCTGAAGGGAAAAAATCTAAAAATGCAAGAGAT
TAB141      AATAAAGATGGGGTTCCACATACAACTAAGTTTAAAACACCTAAAGTGAGGGCACCTGCTGAAGGGAAAAAATCTAAAAATGCAAGAGAT
TAB123      AATAAAGATGGGGTTCCACATACAACTAAGTTTAAAACACCTAAAGTGAGGGCACCTGCTGAAGGGAAAAAATCTAAAAATGCAAGAGAT
AM1803      AATAAAGAGGGAGGTCCACATACAACTAAGTTTAGAACACCTAAAGTGAGGGCACCTGCTGAAGGGAAAAAATCTAAAAATGCAAGAGAT
AM1814      AATAAAGAGGGGGGTCCACATACAACTAAGTTTAAAACACCTAAAGTGAGTGCACCTGCTGGAGGGAAAAAATCTAAAAATTTAAGAGAT
AM1802      AATAAAGAGGGGGGTCCACATACAACTAAGTTTAAAACACCTAAAGTTAGTGCACCTGCTGGAGGGAAAAAATCTAAAAATTTAAGAGGG
AM1811      AATAAAGAGGGAGGTCCACATACAACTAAGTTTAGAACACCTAAAGTGAGGGCACCTGCTGAAGGGAAAAAATCTAAAAATGCAAGAGAT
SC50        AATAAAGAGGGGGGTCCACATACAACTAAGTTTAAAACACCTAAAGTTAGTGCACCTGCTGGAGGGAAAAAATCTAAAAATTTAAGAGGG
SC56        AATAAAGAGGGAGGTCCACATACAACTAAGTTTAGAACACCTAAAGTGAGGGCACCTGCTGAAGGGAAAAAATCTAAAAATGCAAGAGAT
TAB153      AATAAAGATGGGGTTCCACATACAACTAAGTTTAAAACACCTAAAGTGAGGGCACCTGCTGAAGGGAAAAAATCTAAAAATGCAAGAGAT
TAB154      AATAAAGATGGGGTTCCACATACAACTAAGTTTAAAACACCTAAAGTGAGGGCACCTGCTGAAGGGAAAAAATCTAAAAATGCAAGAGAT
TAB156      AATAAAGAGGGGGGTCCACATACAACTAAGTTTAAAACACCTAAAGTTAGTGCACCTGCTGGAGGGAAAAAATCTAAAAATTTAAGAGGG
TAB136      AATAATGAGGGGGGTCAACATACAACTAAGTTTAAAACACCTAAAGTGAGTGCATCTGTTGGAGGGAAAAAATCTAAAAATTTAAGAGGG
AM1880      AATAATGAGGGGGGTCAACATACAACTAAGTTTAAAACACCTAAAGTGAGTGCATCTGTTGGAGGGAAAAAATCTAAAAATTTAAGAGGG
PF3D7_      AATAAAGATGGGGTTCCACATACATCTAGATTTAAAACACCTAAAGTTAGTGCACCTGCTGGAGGGAAAAAATCTAAAAATGCAAGAGAT
PfSD01      AATAAAGATGGGGTTCCACATACATCTAGATTTAAAACACCTAAAGTTAGTGCACCTGCTGGAGGGAAAAAATCTAAAAATGCAAGAGAT
Pf7G8       AATAAAGAGGGGGGTCAACATACAACTAAGTTTAAAACACCTAAAGTGAGTGCATCTGTTGGAGGGAAAAAATCTAAAAATTTAAGAGGG
FCR3 GENE   AATAAAGAGGGGGGTCCACATACATCTAGATTTAAAACACCTAAAGTTAGTGCACCTGCTGGAGGGAAAAAATCTAAAAATGCAAGAGAT
MS822 GENE  AATAAAGATGGGGTTCCACATACATCTAGATTTAAAACACCTAAAGTTAGTGCACCTGCTGGAGGGAAAAAATCTAAAAATGCAAGAGAT
PfCD01_     AATAAAGAGGGGGGTCAACATACAACTAAGTTTAAAACACCTAAAGTGAGTGCATCTGTTGGAGGGAAAAAATCTAAAAATTTAAGAGGG
PfKH01__    AATAAAGATGGGGTTCCACATACAACTAAGTTTAAAACACCTAAAGTGAGGGCACCTGCTGAAGGGAAAAAATCTAAAAATGCAAGAGAT
PfDd2__     AATAAAGATGGGGTTCCACATACAACTAAGTTTAAAACACCTAAAGTGAGGGCACCTGCTGAAGGGAAAAAATCTAAAAATGCAAGAGAT
PfSN01_     AATAAAGATGGGGTTCCACATACAACTAAGTTTAAAACACCTAAAGTGAGGGCACCTGCTGAAGGGAAAAAATCTAAAAATGCAAGAGAT
PfGN01      AATAAAGATGGGGTTCCACATACAACTAAGTTTAAAACACCTAAAGTGAGGGCACCTGCTGAAGGGAAAAAATCTAAAAATGCAAGAGAT
PfKE01      AATAAAGATGGGGTTCCACATACAACTAAGTTTAAAACACCTAAAGTGAGGGCACCTGCTGAAGGGAAAAAATCTAAAAATGCAAGAGAT
PfKH02      AATAAAGATGGGGTTCCACATACAACTAAGTTTAAAACACCTAAAGTGAGGGCACCTGCTGAAGGGAAAAAATCTAAAAATGCAAGAGAT
PfGB4       AATAAAGATGGGGTTCCACATACAACTAAGTTTAAAACACCTAAAGTGAGGGCACCTGCTGAAGGGAAAAAATCTAAAAATGCAAGAGAT
PfGA01_     AATAAAGATGGGGTTCCACATACAACTAAGTTTAAAACACCTAAAGTGAGGGCACCTGCTGAAGGGAAAAAATCTAAAAATGCAAGAGAT
PfHB3_      AATAAAGAGGGGGGTCAACATACAACTAAGTTTAAAACACCTAAAGTGAGTGCATCTGTTGGAGGGAAAAAATCTAAAAATTTAAGAGGG
PfIT_       AATAAAGAGGGGGGTCCACATACATCTAGATTTAAAACACCTAAAGTTAGTGCACCTGCTGGAGGGAAAAAATCTAAAAATGCAAGAGAT
```

```
                     1000      1010      1020      1030      1040      1050      1060      1070      1080
             ....|....|....|....|....|....|....|....|....|....|....|....|....|....|....|....|....|
PF3D7 CDS    AGTACTGAACCAAAACATCATAAGGAAAAAGGAAAATATGATAGGATTAAAAAAACAAGCGAACAAATAAATAAATACGCTCATCCTAAT
MS822 mRNA   AGTACTGAACCAAAACATCATAAGGAAAAAGGAAAATATGATAGGATTAAAAAAACAAGCGAACAAATAAATAAATACGCTCATCCTAAT
FCR3 mRNA    AGTACTGAACCAAAACATCATAAGGAAAAAGGAAAATATGATAGGATTAAAAAAACAAGTGAACAAATAAATAAATACGCTCATCCTGAT
AM1898       AGTACTGAACCAAAACATCAAAAAGAAAAAAGACCATATGATAGGATTAAAAAAACAAGTGAACAAATAAATAAATACGCTCATCCTAAT
AA235        AGTACTGAACCAAAACATCAAAAAGAAAAAAGACCATATGATAGGATTAAAAAAACAAGTGAACAAATAAATAAATACGCTCATCCTGAT
AA243        AGTACTGAACCAAAACATCAAAAAGAAAAAAGACCATATGATAGGATTAAAAAAACAAGTGAACAAATAAATAAATACGCTCATCCTGAT
AA258        AGTACTGAACCAAAACATCAAAAAGAAAAAAGACCATATGATAGGATTAAAAAAACAAGTGAACAAATAAATAAATACGCTCATCCTGAT
AA264        AGTACTGAACCAAAACATCAAAAAGAAAAAAGACCATATGATAGGATTAAAAAAACAAGTGAACAAATAAATAAATACGCTCATCCTGAT
AM1737       AGTACTGAACCAAAACATCAAAAAGAAAAAAGACCATATGATAGGATTAAAAAAACAAGCGAACAAATAAATAAATACGCTCATCCTAAT
SC74         AGTACTGAACCAAAACATCAAAAAGAAAAAAGACCATATGATAGGATTAAAAAAACAAGTGAACAAATAAATAAATACGCTCATCCTGAT
TAB166       AGTACTGAACCAAAACATCAAAAAGAAAAAAGACCATATGATAGGATTAAAAAAACAAGTGAACAAATAAATAAATACGCTCATCCTGAT
TAB138       AGTACTGAACCAAAACATCAAAAAGAAAAAAGACCATATGATAGGATTAAAAAAACAAGCGAACAAATAAATAAATACGCTCATCCTGAT
TAB130       AGTACTGAACCAAAACATCAAAAAGAAAAAAGACCATATGATAGGATTAAAAAAACAAGTGAACAAATAAATAAATACGCTCATCCTGAT
TAB131       AGTACTGAACCAAAACATCAAAAAGAAAAAAGACCATATGATAGGATTAAAAAAACACGTGAACAAATAAATAAATACGCTCATCCTGAT
TAB151       AGTACTGAACCAAAACATCAAAAAGAAAAAAGACCATATGATAGGATTAAAAAAACACGTGAACAAATAAATAAATACGCTCATCCTGAT
TAB152       AGTACTGAACCAAAACATCAAAAAGAAAAAAGACCATATGATAGGATTAAAAAAACAAGTGAACAAATAAATAAATACGCTCATCCTGAT
SC52         AGTACTGAACCAAAACATCAAAAAGAAAAAAGACCATATGATAGGATTAAAAAAACAAGTGAACAAATAAATAAATACGCTCATCCTAAT
SC55         AGTACTGAACCAAAACATCAAAAAGAAAAAAGACCATATGATAGGATTAAAAAAACAAGTGAACAAATAAATAAATACGCTCATCCTAAT
SC03         AGTACTGAACCAAAACATCAAAAAGAAAAAAGACCATATGATAGGATTAAAAAAACAAGTGAACAAATAAATAAATACGCTCATCCTGAT
SC53         AGTACTGAACCAAAACATCAAAAAGAAAAAAGACCATATGATAGGATTAAAAAAACAAGTGAACAAATAAATAAATACGCTCATCCTGAT
TAB141       AGTACTGAACCAAAACATCAAAAAGAAAAAAGACCATATGATAGGATTAAAAAAACAAGTGAACAAATAAATAAATACGCTCATCCTGAT
TAB123       AGTACTGAACCAAAACATCAAAAAGAAAAAAGACCATATGATAGGATTAAAAAAACACGTGAACAAATAAATAAATACGCTCATCCTGAT
AM1803       AGTACTGAACCAAAACATCAAAAAGAAAAAAGACCATATGATAGGATTAAAAAAACAAGTGAACAAATAAATAAATACGCTCATCCTGAT
AM1814       AATACTGAACCAAAACATCAAAAAGAAAAAAGACCATATGATAGGACTAGGAAAACAAGTGAACAAATAAATAAATACGCTCATCCTAAT
AM1802       AATACTGAACCAAAACATCAAAAAGCAAAAACATCATATGATAGGACTAGGAAAACAAGCGAACAAATAAATAAATACGCTCATCCTAAT
AM1811       AGTACTGAACCAAAACATCAAAAAGAAAAAAGACCATATGATAGGATTAAAAAAACAAGTGAACAAATAAATAAATACGCTCATCCTGAT
SC50         AATACTGAACCAAAACATCAAAAAGCAAAAACATCATATGATAGGACTAGGAAAACAAGCGAACAAATAAATAAATACGCTCATCCTAAT
SC56         AGTACTGAACCAAAACATCAAAAAGAAAAAAGACCATATGATAGGATTAAAAAAACAAGTGAACAAATAAATAAATACGCTCATCCTGAT
TAB153       AGTACTGAACCAAAACATCAAAAAGAAAAAAGACCATATGATAGGATTAAAAAAACAAGCGAACAAATAAATAAATACGCTCATCCTAAT
TAB154       AGTACTGAACCAAAACATCAAAAAGAAAAAAGACCATATGATAGGATTAAAAAAACAAGTGAACAAATAAATAAATACGCTCATCCTAAT
TAB156       AATACTGAACCAAAACATCAAAAAGCAAAAACATCATATGATAGGACTAGGAAAACAAGCGAACAAATAAATAAATACGCTCATCCTAAT
TAB136       AATACTGAACCAAAACATCAAAAAGAAAAAACATCATATGATAGGACTAGGAAAACAAGTGAACAAATAAATAAATACGCTCATCCTAAT
AM1880       AATACTGAACCAAAACATCAAAAAGAAAAAACATCATATGATAGGACTAGGAAAACAAGTGAACAAATAAATAAATACGCTCATCCTAAT
PF3D7_       AGTACTGAACCAAAACATCATAAGGAAAAAGGAAAATATGATAGGATTAAAAAAACAAGCGAACAAATAAATAAATACGCTCATCCTAAT
PfSD01       AGTACTGAACCAAAACATCATAAGGAAAAAGGAAAATATGATAGGATTAAAAAAACAAGCGAACAAATAAATAAATACGCTCATCCTAAT
Pf7G8        AATACTGAACCAAAACATCAAAAAGAAAAAACATCATATGATAGGACTAGGAAAACAAGTGAACAAATAAATAAATACGCTCATCCTAAT
FCR3 GENE    AGTACTGAACCAAAACATCATAAGGAAAAAGGAAAATATGATAGGATTAAAAAAACAAGTGAACAAATAAATAAATACGCTCATCCTGAT
MS822 GENE   AGTACTGAACCAAAACATCATAAGGAAAAAGGAAAATATGATAGGATTAAAAAAACAAGCGAACAAATAAATAAATACGCTCATCCTAAT
PfCD01_      AATACTGAACCAAAACATCAAAAAGAAAAAACATCATATGATAGGACTAGGAAAACAAGTGAACAAATAAATAAATACGCTCATCCTAAT
PfKH01__     AGTACTGAACCAAAACATCAAAAAGAAAAAAGACCATATGATAGGATTAAAAAAACACGTGAACAAATAAATAAATACGCTCATCCTGAT
PfDd2__      AGTACTGAACCAAAACATCATAAGGAAAAAGGAAAATATGATAGGATTAAAAAAACAAGCGAACAAATAAATAAATACGCTCATCCTAAT
PfSN01_      AATACTGAACCAAAACATCAAAAAGAAAAAAGACCATATGATAGGATTAAAAAAACACGTGAACAAATAAATAAATACGCTCATCCTAAT
PfGN01       AATACTGAACCAAAACATCAAAAAGAAAAAAGACCATATGATAGGATTAAAAAAACACGTGAACAAATAAATAAATACGCTCATCCTAAT
PfKE01       AGTACTGAACCAAAACATCAAAAAGAAAAAAGACCATATGATAGGATTAAAAAAACACGTGAACAAATAAATAAATACGCTCATCCTGAT
PfKH02       AGTACTGAACCAAAACATCAAAAAGAAAAAAGACCATATGATAGGATTAAAAAAACACGTGAACAAATAAATAAATACGCTCATCCTGAT
PfGB4        AGTACTGAACCAAAACATCAAAAAGAAAAAAGACCATATGATAGGATTAAAAAAACACGTGAACAAATAAATAAATACGCTCATCCTGAT
PfGA01_      AGTACTGAACCAAAACATCAAAAAGAAAAAAGACCATATGATAGGATTAAAAAAACACGTGAACAAATAAATAAATACGCTCATCCTAAT
PfHB3_       AATACTGAACCAAAACATCAAAAAGAAAAAACATCATATGATAGGACTAGGAAAACAAGTGAACAAATAAATAAATACGCTCATCCTAAT
PfIT_        AGTACTGAACCAAAACATCATAAGGAAAAAGGAAAATATGATAGGATTAAAAAAACAAGTGAACAAATAAATAAATACGCTCATCCTGAT
```

```
                    1090      1100      1110      1120      1130      1140      1150      1160      1170
            ....|....|....|....|....|....|....|....|....|....|....|....|....|....|....|....|....|....|
PF3D7 CDS   AATAAATATACTATCTTTCAGTGTACTGATTTAGATTGTAAATGGCTGAAACCTAAACATATTAAATATCCATATGAAGGTTATGATATA
MS822 mRNA  AATAAATATACTATCTTTCAGTGTACTGATTTAGATTGTAAATGGCTGAAACCTAAACATATTAAATATCCATATGAAGGTTATGATATA
FCR3 mRNA   AATAAATATACTATCTTTCAGTGTACTGATTTAGATTGTAAATGGCTGAAACCTAAAAATATTAAATATCCATATGAAGGTTATGATATA
AM1898      AATAAATATACTATCTTTCAGTGTACTGATTTAGATTGTAAATGGCTGAAACCTAAACATATTAAATATCCATATGAAGGTTATGATATA
AA235       AATAAATATACTATCTTTCAGTGTACTGATTTAGATTGTAAATGGCTGAAACCTAAACATATTAAATATCCATATGAAGGTTATGATATA
AA243       AATAAATATACTATCTTTCAGTGTACTGATTTAGATTGTAAATGGCTGAAACCTAAACATATTAAATATCCATATGAAGGTTATGATATA
AA258       AATAAATATACTATCTTTCAGTGTACTGATTTAGATTGTAAATGGCTGAAACCTAAACATATTAAATATCCATATGAAGGTTATGATATA
AA264       AATAAATATACTATCTTTCAGTGTACTGATTTAGATTGTAAATGGCTGAAACCTAAACATATTAAATATCCATATGAAGGTTATGATATA
AM1737      AATAAATATACTATCTTTCAGTGTACTGATTTAGATTGTAAAAGGCTGAAACCTAAACATATTAAATATCCATATGAAGGTTATGATATA
SC74        AATAAATATACTATCTTTCAGTGTACTGATTTAGATTGTAAATGGCTGAAACCTAAACATATTAAATATCCATATGAAGGTTATGATATA
TAB166      AATAAATATACTATCTTTCAGTGTACTGATTTAGATTGTAAATGGCTGAAACCTAAACATATTAAATATCCATATGAAGGTTATGATATA
TAB138      AATAAATATACTATCTTTCAGTGTACTGATTTAGATTGTAAATGGCTGAAACCTAAACATATTAAATATCCATATGAAGGTTATGATATA
TAB130      AATAAATATACTATCTTTCAGTGTACTGATTTAGATTGTAAATGGCTGAAACCTAAACATATTAAATATCCATATGAAGGTTATGATATA
TAB131      AATAAATATACTATCTTTCAGTGTACTGATTTAGATTGTAAATGGCTGAAACCTAAACATATTAAATATCCATATGAAGGTTATGATATA
TAB151      AATAAATATACTATCTTTCAGTGTACTGATTTAGATTGTAAATGGCTGAAACCTAAACATATTAAATATCCATATGAAGGTTATGATATA
TAB152      AATAAATATACTATCTTTCAGTGTACTGATTTAGATTGTAAATGGCTGAAACCTAAACATATTAAATATCCATATGAAGGTTATGATATA
SC52        AATAAATATACTATCTTTCAGTGTACTGATTTAGATTGTAAATGGCTGAAACCTAAACATATTAAATATCCATATGAAGGTTATGATATA
SC55        AATAAATATACTATCTTTCAGTGTACTGATTTAGATTGTAAATGGCTGAAACCTAAACATATTAAATATCCATATGAAGGTTATGATATA
SC03        AATAAATATACTATCTTTCAGTGTACTGATTTAGATTGTAAATGGCTGAAACCTAAACATATTAAATATCCATATGAAGGTTATGATATA
SC53        AATAAATATACTATCTTTCAGTGTACTGATTTAGATTGTAAATGGCTGAAACCTAAACATATTAAATATCCATATGAAGGTTATGATATA
TAB141      AATAAATATACTATCTTTCAGTGTACTGATTTAGATTGTAAATGGCTGAAACCTAAACATATTAAATATCCATATGAAGGTTATGATATA
TAB123      AATAAATATACTATCTTTCAGTGTACTGATTTAGATTGTAAATGGCTGAAACCTAAACATATTAAATATCCATATGAAGGTTATGATATA
AM1803      AATAAATATACTATCTTTCAGTGTACTGATTTAGATTGTAAATGTCTGAAACCTAAAAATATTAAATATCCATATGAAGGTTATGATATA
AM1814      AATAAATATACTATCTTTCAGTGTACTGATTTAGATTGTAAATGGCTGAAACCTAAAAATATTAAATATCCATATGAAGGTTATGATATA
AM1802      AATAAATATACTATCTTTCAGTGTACTGATTTAGATTGTAAATGGCTGAAACCTAAAAATATTAAATATCCATATGAAAGTTATGATATA
AM1811      AATAAATATACTATCTTTCAGTGTACTGATTTAGATTGTAAATGTCTGAAACCTAAAAATATTAAATATCCATATGAAGGTTATGATATA
SC50        AATAAATATACTATCTTTCAGTGTACTGATTTAGATTGTAAATGGCTGAAACCTAAAAATATTAAATATCCATATGAAAGTTATGATATA
SC56        AATAAATATACTATCTTTCAGTGTACTGATTTAGATTGTAAATGTCTGAAACCTAAAAATATTAAATATCCATATGAAGGTTATGATATA
TAB153      AATAAATATACTATCTTTCAGTGTACTGATTTAGATTGTAAAAGGCTGAAACCTAAACATATTAAATATCCATATGAAGGTTATGATATA
TAB154      AATAAATATACTATCTTTCAGTGTACTGATTTAGATTGTAAATGGCTGAAACCTAAACATATTAAATATCCATATGAAGGTTATGATATA
TAB156      AATAAATATACTATCTTTCAGTGTACTGATTTAGATTGTAAATGGCTGAAACCTAAAAATATTAAATATCCATATGAAAGTTATGATATA
TAB136      AATAAATATACTATCTTTCAGTGTACTGATTTAGATTGTAAATGGCTGAAACCTAAAAATATTAAATATACATATGAAGGTTATGATATA
AM1880      AATAAATATACTATCTTTCAGTGTACTGATTTAGATTGTAAATGGCTGAAACCTAAAAATATTAAATATACATATGAAGGTTATGATATA
PF3D7_      AATAAATATACTATCTTTCAGTGTACTGATTTAGATTGTAAATGGCTGAAACCTAAACATATTAAATATCCATATGAAGGTTATGATATA
PfSD01      AATAAATATACTATCTTTCAGTGTACTGATTTAGATTGTAAATGGCTGAAACCTAAACATATTAAATATCCATATGAAGGTTATGATATA
Pf7G8       AATAAATATACTATCTTTCAGTGTACTGATTTAGATTGTAAATGGCTGAAACCTAAAAATATTAAATATACATATGAAGGTCATGATATA
FCR3 GENE   AATAAATATACTATCTTTCAGTGTACTGATTTAGATTGTAAATGGCTGAAACCTAAAAATATTAAATATCCATATGAAGGTTATGATATA
MS822 GENE  AATAAATATACTATCTTTCAGTGTACTGATTTAGATTGTAAATGGCTGAAACCTAAACATATTAAATATCCATATGAAGGTTATGATATA
PfCD01_     AATAAATATACTATCTTTCAGTGTACTGATTTAGATTGTAAATGGCTGAAACCTAAAAATATTAAATATACATATGAAGGTCATGATATA
PfKH01__    AATAAATATACTATCTTTCAGTGTACTGATTTAGATTGTAAATGGCTGAAACCTAAACATATTAAATATCCATATGAAGGTTATGATATA
PfDd2__     AATAAATATACTATCTTTCAGTGTACTGATTTAGATTGTAAATGGCTGAAACCTAAACATATTAAATATCCATATGAGGGTTATGATATA
PfSN01_     AATAAATATACTATCTTTCAGTGTACTGATTTAGATTGTAAATGGCTGAAACCTAAACATATTAAATATCCATATGAAGGTTATGATATA
PfGN01      AATAAATATACTATCTTTCAGTGTACTGATTTAGATTGTAAATGGCTGAAACCTAAACATATTAAATATCCATATGAAGGTTATGATATA
PfKE01      AATAAATATACTATCTTTCAGTGTACTGATTTAGATTGTAAATGGCTGAAACCTAAACATATTAAATATCCATATGAAGGTTATGATATA
PfKH02      AATAAATATACTATCTTTCAGTGTACTGATTTAGATTGTAAATGGCTGAAACCTAAACATATTAAATATCCATATGAAGGTTATGATATA
PfGB4       AATAAATATACTATCTTTCAGTGTACTGATTTAGATTGTAAATGGCTGAAACCTAAACATATTAAATATCCATATGAAGGTTATGATATA
PfGA01_     AATAAATATACTATCTTTCAGTGTACTGATTTAGATTGTAAATGGCTGAAACCTAAACATATTAAATATCCATATGAAGGTTATGATATA
PfHB3_      AATAAATATACTATCTTTCAGTGTACTGATTTAGATTGTAAATGGCTGAAACCTAAAAATATTAAATATACATATGAAGGTTATGATATA
PfIT_       AATAAATATACTATCTTTCAGTGTACTGATTTAGATTGTAAATGGCTGAAACCTAAAAATATTAAATATCCATATGAAGGTTATGATATA
```

```
                  1180      1190      1200      1210      1220      1230      1240      1250      1260
              ....|....|....|....|....|....|....|....|....|....|....|....|....|....|....|....|....|....|
PF3D7 CDS     GTTGATAATGATAAACTTGTAGATACAGGAAAGGGAGATTATGAAGATCATCATACATATTGTTCTGGAGATGAATGTGCATTTGGTAAT
MS822 mRNA    GTTGATAATGATAAACTTGTAGATACAGGAAAGGGAGATTATGAAGATCATCATACATATTGTTCTGGAGATGAATGTGCATTTGGTAAT
FCR3 mRNA     GTTGATAATGATAAACTTGTAGATACAGGAAAGGGAGATTATGAAGATCATCATACATATTGTTCTGGAGATGAATGTGCATTTGGTAAT
AM1898        GTTGATAATGATAAACTTGTAGATACAGGAAAGGGAGTTTATGAAGATCATCATACATATTGTTCTGGAGATGAATGTGCATTTGGTAAT
AA235         GTTGATAATGATAAACTTGTAGATACAGGAAAGGGAGATTATGAAGATCATCATACATATTGTTCTGGAGATGAATGTGCATTTGGTAAT
AA243         GTTGATAATGATAAACTTGTAGATACAGGAAAGGGAGATTATGAAGATCATCATACATATTGTTCTGGAGATGAATGTGCATTTGGTAAT
AA258         GTTGATAATGATAAACTTGTAGATACAGGAAAGGGAGATTATGAAGATCATCATACATATTGTTCTGGAGATGAATGTGCATTTGGTAAT
AA264         GTTGATAATGATAAACTTGTAGATACAGGAAAGGGAGATTATGAAGATCATCATACATATTGTTCTGGAGATGAATGTGCATTTGGTAAT
AM1737        GTTGATAATGATAAACTTGTAGATACAGGAAAGGGAGATTATGAAGATCATCATACATATTGTTCTGGAGATGAATGTGCATTTGGTAAT
SC74          GTTGATAATGATAAACTTGTAGATACAGGAAAGGGAGATTATGAAGATCATCATACATATTGTTCTGGAGATGAATGTGCATTTGGTAAT
TAB166        GTTGATAATGATAAACTTGTAGATACAGGAAAGGGAGATTATGAAGATCATCATACATATTGTTCTGGAGATGAATGTGCATTTGGTAAT
TAB138        GTTGATAATGATAAACTTGTAGATACAGGAAAGGGAGATTATGAAGATCATCATACATATTGTTCTGGAGATGAATGTGCATTTGGTAAT
TAB130        GTTGATAATGATAAACTTGTAGATACAGGAAAGGGAGATTATGAAGATCATCATACATATTGTTCTGGAGATGAATGTGCATTTGGTAAT
TAB131        GTTGATAATGATAAACTTGTAGATACAGGAAAGGGAGATTATGAAGATCATCATACATATTGTTCTGGAGATGAATGTGCATTTGGTAAT
TAB151        GTTGATAATGATAAACTTGTAGATACAGGAAAGGGAGATTATGAAGATCATCATACATATTGTTCTGGAGATGAATGTGCATTTGGTAAT
TAB152        GTTGATAATGATAAACTTGTAGATACAGGAAAGGGAGATTATGAAGATCATCATACATATTGTTCTGGAGATGAATGTGCATTTGGTAAT
SC52          GTTGATAATGATAAACTTGTAGATACAGGAAAGGGAGATTATGAAGATCATCATACATATTGTTCTGGAGATGAATGTGCATTTGGTAAT
SC55          GTTGATAATGATAAACTTGTAGATACAGGAAAGGGAGATTATGAAGATCATCATACATATTGTTCTGGAGATGAATGTGCATTTGGTAAT
SC03          GTTGATAATGATAAACTTGTAGATACAGGAAAGGGAGATTATGAAGATCATCATACATATTGTTCTGGAGATGAATGTGCATTTGGTAAT
SC53          GTTGATAATGATAAACTTGTAGATACAGGAAAGGGAGATTATGAAGATCATCATACATATTGTTCTGGAGATGAATGTGCATTTGGTAAT
TAB141        GTTGATAATGATAAACTTGTAGATACAGGAAAGGGAGATTATGAAGATCATCATACATATTGTTCTGGAGATGAATGTGCATTTGGTAAT
TAB123        GTTGATAATGATAAACTTGTAGATACAGGAAAGGGAGATTATGAAGATCATCATACATATTGTTCTGGAGATGAATGTGCATTTGGTAAT
AM1803        GTTGATAATGATAAACTTGTAGATACAGGAAAGGGAGATTATGAAGATCATCATACATATTGTTCTGGAGATGAATGTGCATTTGGTAAT
AM1814        GTTGATAATGATAAACTTGTAGATACAGGAAAGGGAGTTTATGAAGATCATCATACATATTGTTCTGGAGATGAATGTGCATTGGGTAAT
AM1802        GTTGATAATGATAAATTTGTAGATACAGGAAAGGGAGTTTATGAAGATCATCATACATATTGTTCTGGAGATGAATGTGCATTGGGTAAT
AM1811        GTTGATAATGATAAACTTGTAGATACAGGAAAGGGAGATTATGAAGATCATCATACATATTGTTCTGGAGATGAATGTGCATTTGGTAAT
SC50          GTTGATAATGATAAATTTGTAGATACAGGAAAGGGAGTTTATGAAGATCATCATACATATTGTTCTGGAGATGAATGTGCATTGGGTAAT
SC56          GTTGATAATGATAAACTTGTAGATACAGGAAAGGGAGATTATGAAGATCATCATACATATTGTTCTGGAGATGAATGTGCATTTGGTAAT
TAB153        GTTGATAATGATAAACTTGTAGATACAGGAAAGGGAGATTATGAAGATCATCATACATATTGTTCTGGAGATGAATGTGCATTTGGTAAT
TAB154        GTTGATAATGATAAACTTGTAGATACAGGAAAGGGAGATTATGAAGATCATCATACATATTGTTCTGGAGATGAATGTGCATTTGGTAAT
TAB156        GTTGATAATGATAAATTTGTAGATACAGGAAAGGGAGTTTATGAAGATCATCATACATATTGTTCTGGAGATGAATGTGCATTGGGTAAT
TAB136        GTTGATAATGATAAACTTGTAGATACAGGAAAGGGAGTTTATGAAGATTATAATAAATATTGTTCTGGAGATGAATGTGCATTGGGTAAT
AM1880        GTTGATAATGATAAACTTGTAGATACAGGAAAGGGAGTTTATGAAGATTATAATAAATATTGTTCTGGAGATGAATGTGCATTGGGTAAT
PF3D7_        GTTGATAATGATAAACTTGTAGATACAGGAAAGGGAGATTATGAAGATCATCATACATATTGTTCTGGAGATGAATGTGCATTTGGTAAT
PfSD01        GTTGATAATGATAAACTTGTAGATACAGGAAAGGGAGATTATGAAGATCATCATACATATTGTTCTGGAGATGAATGTGCATTTGGTAAT
Pf7G8         GTTGATAATGATAAACTTGTAGATACAGGAAAGGGAGTTTATGAAGATTATAATAAATATTGTTCTGGAGATGAATGTGCATTGGGTAAT
FCR3 GENE     GTTGATAATGATAAACTTGTAGATACAGGAAAGGGAGATTATGAAGATCATCATACATATTGTTCTGGAGATGAATGTGCATTTGGTAAT
MS822 GENE    GTTGATAATGATAAACTTGTAGATACAGGAAAGGGAGATTATGAAGATCATCATACATATTGTTCTGGAGATGAATGTGCATTTGGTAAT
PfCD01_       GTTGATAATGATAAACTTGTAGATACAGGAAAGGGAGTTTATGAAGATTATAATAAATATTGTTCTGGAGATGAATGTGCATTGGGTAAT
PfKH01__      GTTGATAATGATAAACTTGTAGATACAGGAAAGGGAGATTATGAAGATCATCATACATATTGTTCTGGAGATGAATGTGCATTTGGTAAT
PfDd2__       GTTGATAATGATAAACTTGTAGATACAGGAAAGGGAGATTATGAAGATCATCATACATATTGTTCTGGAGATGAATGTGCATTTGGTAAT
PfSN01_       GTTGATAATGATAAACTTGTAGATACAGGAAAGGGAGATTATGAAGATCATCATACATATTGTTCTGGAGATGAATGTGCATTTGGTAAT
PfGN01        GTTGATAATGATAAACTTGTAGATACAGGAAAGGGAGATTATGAAGATCATCATACATATTGTTCTGGAGATGAATGTGCATTTGGTAAT
PfKE01        GTTGATAATGATAAACTTGTAGATACAGGAAAGGGAGATTATGAAGATCATCATACATATTGTTCTGGAGATGAATGTGCATTTGGTAAT
PfKH02        GTTGATAATGATAAACTTGTAGATACAGGAAAGGGAGATTATGAAGATCATCATACATATTGTTCTGGAGATGAATGTGCATTTGGTAAT
PfGB4         GTTGATAATGATAAACTTGTAGATACAGGAAAGGGAGATTATGAAGATCATCATACATATTGTTCTGGAGATGAATGTGCATTTGGTAAT
PfGA01_       GTTGATAATGATAAACTTGTAGATACAGGAAAGGGAGATTATGAAGATCATCATACATATTGTTCTGGAGATGAATGTGCATTTGGTAAT
PfHB3_        GTTGATAATGATAAACTTGTAGATACAGGAAAGGGAGTTTATGAAGATTATAATAAATATTGTTCTGGAGATGAATGTGCATTGGGTAAT
PfIT_         GTTGATAATGATAAACTTGTAGATACAGGAAAGGGAGATTATGAAGATCATCATACATATTGTTCTGGAGATGAATGTGCATTTGGTAAT
```

```
                 1270      1280      1290      1300      1310      1320      1330      1340      1350
             ....|....|....|....|....|....|....|....|....|....|....|....|....|....|....|....|....|....|
PF3D7 CDS    GCTGTTGGGGGACAGTTTGACGAAAGCAGATCTCAAGGTAAGCAAAGAAGAAAAAAAGGGAAAGGAAAGGGTAGAAAAAAGGGTCATAAT
MS822 mRNA   GCTGTTGGGGGACAGTTTGACGAAAGCAGATCTCAAGGTAAGCAAAGAAGAAAAAAAGGGAAAGGAAAGGGTAGAAAAAAGGGTCATAAT
FCR3 mRNA    GCTGTTGGGGGACAGTTTGATGAAATAGGGTCGGAAGGTAATCTAAAAAGAAAAAAAGGGAAAGGAAAGGGTAGAAAAAAGGGTCATAAT
AM1898       GCTGTTGAGGGACAGTTTGACGAAAGCAGATCTCAAGGTAAGCAAAGAAGAAAAAAAGGGAAAGGAAAGGGTAGAAAAAAGGGTCAT---
AA235        GCTGTTGAGGGACAGTTTGACGAAAGCAGATCTCAAGGTAAGCAAAGAAGAAAAAAAGGGAAAGGAAAGGGTAGAAAAAAGGGTCATAAT
AA243        GCTGTTGAGGGACAGTTTGACGAAAGCAGATCTCAAGGTAAGCAAAGAAGAAAAAAAGGGAAAGGAAAGGGTAGAAAAAAGGGTCATAAT
AA258        GCTGTTGAGGGACAGTTTGACGAAAGCAGATCTCAAGGTAAGCAAAGAAGAAAAAAAGGGAAAGGAAAGGGTAGAAAAAAGGGTCATAAT
AA264        GCTGTTGAGGGACAGTTTGACGAAAGCAGATCTCAAGGTAAGCAAAGAAGAAAAAAAGGGAAAGGAAAGGGTAGAAAAAAGGGTCAT---
AM1737       GCTGTTGAGGGACAGTTTGACGAAAGCAGATCTCAAGGTAAGCAAAGAAGAAAAAAAGGGAAAGGAAAGGGTAGAAAAAAGGGTCAT---
SC74         GCTGTTGAGGGACAGTTTGACGAAAGCAGATCTCAAGGTAAGCAAAGAAGAAAAAAAGGGAAAGGAAAGGGTAGAAAAAAGGGTCAT---
TAB166       GCTGTTGAGGGACAGTTTGACGAAAGCAGATCTCAAGGTAAGCAAAGAAGAAAAAAAGGGAAAGGAAAGGGTAGAAAAAAGGGTCATAAT
TAB138       GCTGTTGAGGGACAGTTTGACGAAAGCAGATCTCAAGGTAAGCAAAGAAGAAAAAAAGGGAAAGGAAAGGGTAGAAAAAAGGGTCAT---
TAB130       GCTGTTGAGGGACAGTTTGACGAAAGCAGATCTCAAGGTAAGCAAAGAAGAAAAAAAGGGAAAGGAAAGGGTAGAAAAAAGGGTCAT---
TAB131       GCTGTTGAGGGACAGTTTGACGAAAGCAGATCTCAAGGTAAGCAAAGAAGAAAAAAAGGGAAAGGAAAGGGTAGAAAAAAGGGTCAT---
TAB151       GCTGTTGAGGGACAGTTTGACGAAAGCAGATCTCAAGGTAAGCAAAGAAGAAAAAAAGGGAAAGGAAAGGGTAGAAAAAAGGGTCAT---
TAB152       GCTGTTGAGGGACAGTTTGACGAAAGCAGATCTCAAGGTAAGCAAAGAAGAAAAAAAGGGAAAGGAAAGGGTAGAAAAAAGGGTCAT---
SC52         GCTGTTGGGGGACAGTTTGACGAAAGCAGATCTCAAGGTAAGCAAAGAAGAAAAAAAGGGAAAGGAAAGGGTAGAAAAAAGGGTCATAAT
SC55         GCTGTTGAGGGACAGTTTGACGAAAGCAGATCTCAAGGTAAGCAAAGAAGAAAAAAAGGGAAAGGAAAGGGTAGAAAAAAGGGTCATAAT
SC03         GCTGTTGAGGGACAGTTTGACGAAAGCAGATCTCAAGGTAAGCAAAGAAGAAAAAAAGGGAAAGGAAAGGGTAGAAAAAAGGGTCATAAT
SC53         GCTGTTGAGGGACAGTTTGACGAAAGCAGATCTCAAGGTAAGCAAAGAAGAAAAAAAGGGAAAGGAAAGGGTAGAAAAAAGGGTCAT---
TAB141       GCTGTTGAGGGACAGTTTGACGAAAGCAGATCTCAAGGTAAGCAAAGAAGAAAAAAAGGGAAAGGAAAGGGTAGAAAAAAGGGTCAT---
TAB123       GCTGTTGAGGGACAGTTTGACGAAAGCAGATCTCAAGGTAAGCAAAGAAGAAAAAAAGGGAAAGGAAAGGGTAGAAAAAAGGGTCAT---
AM1803       GCTGTTGAGGGACAGTTTGACGAAAGCAGATCTCAAGGTAAGCAAAGAAGAAAAAAAGGGAAAGGAAAGGGTAGAAAAAAGGGTCATAAT
AM1814       GCTGTTGAGGGACAGTTTGACGAAAGCAGATCTCAAGGTAAGCAAAGAAGAAAAAAAGGGAAAGGAAAGGGTAGAAAAAAGGGTCATAAT
AM1802       GCTGTCGAGGGACAGTTTGACGAAAGCAGATCTCAAGGTAAGAAAAGAAGAAAAAAAGTTAAAGGAGAGGGTAGAAAAAAGGGTCATAAT
AM1811       GCTGTTGAGGGACAGTTTGACGAAAGCAGATCTCAAGGTAAGCAAAGAAGAAAAAAAGGGAAAGGAAAGGGTAGAAAAAAGGGTCAT---
SC50         GCTGTCGAGGGACAGTTTGACGAAAGCAGATCTCAAGGTAAGAAAAGAAGAAAAAAAGTTAAAGGAGAGGGTAGAAAAAAGGGTCATAAT
SC56         GCTGTTGAGGGACAGTTTGACGAAAGCAGATCTCAAGGTAAGCAAAGAAGAAAAAAAGGGAAAGGAAAGGGTAGAAAAAAGGGTCAT---
TAB153       GCTGTTGAGGGACAGTTTGACGAAAGCAGATCTCAAGGTAAGCAAAGAAGAAAAAAAGGGAAAGGAAAGGGTAGAAAAAAGGGTCATAAT
TAB154       GCTGTTGAGGGACAGTTTGACGAAAGCAGATCTCAAGGTAAGCAAAGAAGAAAAAAAGGGAAAGGAAAGGGTAGAAAAAAGGGTCATAAT
TAB156       GCTGTCGAGGGACAGTTTGACGAAAGCAGATCTCAAGGTAAGAAAAGAAGAAAAAAAGTTAAAGGAGAGGGTAGAAAAAAGGGTCATAAT
TAB136       GCTGTTGAGGGACAGTTTGATGAAATAGGGTCGGAAGGTAATCTAAAAAGAAAAAAAGGGAAAGGAAAGGGTAGAAAAAAGGATCAT---
AM1880       GCTGTTGAGGGACAGTTTGATGAAATAGGGTCGGAAGGTAATCTAAAAAGAAAAAAAGGGAAAGGAAAGGGTAGAAAAAAGGATCAT---
PF3D7_       GCTGTTGGGGGACAGTTTGACGAAAGCAGATCTCAAGGTAAGCAAAGAAGAAAAAAAGGGAAAGGAAAGGGTAGAAAAAAGGGTCATAAT
PfSD01       GCTGTTGAGGGACAGTTTGACGAAAGCAGATCTCAAGGTAAGCAAAGAAGAAAAAAAGGGAAAGGAAAGGGTAGAAAAAAGGGTCAT---
Pf7G8        GCTGTTGAGGGACAGTTTGATGAAATAGGGTCGGAAGGTAATCTAAAAAGAAAAAAAGGGAAAGGAAAGGGTAGAAAAAAGGATCAT---
FCR3 GENE    GCTGTTGGGGGACAGTTTGATGAAATAGGGTCGGAAGGTAATCTAAAAAGAAAAAAAGGGAAAGGAAAGGGTAGAAAAAAGGGTCATAAT
MS822 GENE   GCTGTTGGGGGACAGTTTGACGAAAGCAGATCTCAAGGTAAGCAAAGAAGAAAAAAAGGGAAAGGAAAGGGTAGAAAAAAGGGTCATAAT
PfCD01_      GCTGTTGAGGGACAGTTTGATGAAATAGGGTCGGAAGGTAATCTAAAAAGAAAAAAAGGGAAAGGAAAGGGTAGAAAAAAGGATCAT---
PfKH01__     GCTGTTGAGGGACAGTTTGACGAAAGCAGATCTCAAGGTAAGCAAAGAAGAAAAAAAGGGAAAGGAAAGGGTAGAAAAAAGGGTCAT---
PfDd2__      GCTGTTGAGGGACAGTTTGACGAAAGCAGATCTCAAGGTAAGCAAAGAAGAAAAAAAGGGAAAGGAAAGGGTAGAAAAAAGGGTCAT---
PfSN01_      GCTGTTGAGGGACAGTTTGACGAAAGCAGATCTCAAGGTAAGCAAAGAAGAAAAAAAGGGAAAGGAAAGGGTAGAAAAAAGGGTCATAAT
PfGN01       GCTGTTGAGGGACAGTTTGACGAAAGCAGATCTCAAGGTAAGCAAAGAAGAAAAAAAGGGAAAGGAAAGGGTAGAAAAAAGGGTCATAAT
PfKE01       GCTGTTGAGGGACAGTTTGACGAAAGCAGATCTCAAGGTAAGCAAAGAAGAAAAAAAGGGAAAGGAAAGGGTAGAAAAAAGGGTCATAAT
PfKH02       GCTGTTGGGGGACAGTTTGATGAAATAGGGTCGGAAGGTAATCTAAAAAGAAAAAAAGGGAAAGGAAAGGGTAGAAAAAAGGGTCATAAT
PfGB4        GCTGTTGGGGGACAGTTTGACGAAAGCAGATCTCAAGGTAAGCAAAGAAGAAAAAAAGGGAAAGGAAAGGGTAGAAAAAAGGGTCATAAT
PfGA01_      GCTGTTGAGGGACAGTTTGACGAAAGCAGATCTCAAGGTAAGCAAAGAAGAAAAAAAGGGAAAGGAAAGGGTAGAAAAAAGGGTCATAAT
PfHB3_       GCTGTTGAGGGACAGTTTGATGAAATAGGGTCGGAAGGTAATCTAAAAAGAAAAAAAGGGAAAGGAAAGGGTAGAAAAAAGGATCAT---
PfIT_        GCTGTTGGGGGACAGTTTGATGAAATAGGGTCGGAAGGTAATCTAAAAAGAAAAAAAGGGAAAGGAAAGGGTAGAAAAAAGGGTCATAAT
```

```
                1360      1370      1380      1390      1400      1410      1420      1430      1440
            ....|....|....|....|....|....|....|....|....|....|....|....|....|....|....|....|....|....|
PF3D7 CDS   AAATTAGAAGGTAAAAGTTCACCTAATGTTGAAGGTACTCATTCTTATATAACAAATGGAGGTAATATAGAAGAAACAAAAAGCCAATCA
MS822 mRNA  AAATTAGAAGGTAAAAGTTCACCTAATGTTGAAGGTACTCATTCTTATATAACAAATGGAGGTAATATAGAAGAAACAAAAAGCCAATCA
FCR3 mRNA   AAATTAGAAGGTAAAAGTTCACCTAATGTTGAAGGTACTCATTCTTATATAACAAATGGAGGTAATATAGAAGAAACAAAAAGCCAATCA
AM1898      AAATCAGTAGGTAAAAGTTTACCTAATGGTGAAGGTACTCATTCTTATATGAGACATGGAGATAATAAAGAAGAAACAAAAAGCCAATCA
AA235       AAATTAGAAGGTAAAAGTTCACCTAATGGTGAAGGTACTCATTCTTATATAACAAATGGAGGTAATAGAGAAGAAACAAAAAGCCAATCA
AA243       AAATTAGAAGGTAAAAGTTCACCTAATGTTGAAGGTACTCATTCTTATATAACAAATGGAGGTAATATAGAAGAAACAAAAAGCCAATCA
AA258       AAATTAGAAGGTAAAAGTTCACCTAATGTTGAAGGTACTCATTCTTATATAACAAATGGAGGTAATATAGAAGAAACAAAAAGCCAATCA
AA264       AAATCAGTAGGTAAAAGTTTACCTAATGGTGAAGGTACTCATTCTTATATAACAAATGGAGGTAATAGAGAAGAAACAAAAAGCCAATCA
AM1737      AAATCAGTAGGTAAAAGTTTACCTAATGGTGAAGGTACTCATTCTTATATAACAAATGGAGGTAATAGAGAAGAAACAAAAAGCCAATCA
SC74        AAATCAGTAGGTAAAAGTTTACCTAATGGTGAAGGTACTCATTCTTATATAACAAATGGAGGTAATAGAGAAGAAACAAAAAGCCAATCA
TAB166      AAATTAGAAGGTAAAAGTTCACCTAATGTTGAAGGTACTCATTCTTATATAACAAATGGAGGTAATATAGAAGAAACAAAAAGCCAATCA
TAB138      AAATCAGTAGGTAAAAGTTTACCTAATGGTGAAGGTACTCATTCTTATATAACAAATGGAGGTAATAGAGAAGAAACAAAAAGCCAATCA
TAB130      AAATCAGTAGGTAAAAGTTTACCTAATGGTGAAGGTACTCATTCTTATATAACAAATGGAGGTAATAGAGAAGAAACAAAAAGCCAATCA
TAB131      AAATCAGTAGGTAAAAGTTTACCTAATGGTGAAGGTACTCATTCTTATATAACAAATGGAGGTAATAGAGAAGAAACAAAAAGCCAATCA
TAB151      AAATCAGTAGGTAAAAGTTTACCTAATGGTGAAGGTACTCATTCTTATATAACAAATGGAGGTAATAGAGAAGAAACAAAAAGCCAATCA
TAB152      AAATCAGTAGGTAAAAGTTTACCTAATGGTGAAGGTACTCATTCTTATATAACAAATGGAGGTAATAGAGAAGAAACAAAAAGCCAATCA
SC52        AAATTAGAAGGTAAAAGTTCACCTAATGTTGAAGGTACTCATTCTTATATAACAAATGGAGGTAATAGAGAAGAAACAAAAAGCCAATCA
SC55        AAATCAGTAGGTAAAAGTTTACCTAATGGTGAAGGTACTCATTCTTATATGAGACATGGAGATAATAAAGAAGAAACAAAAAGCCAATCA
SC03        AAATTAGAAGGTAAAAGTTCACCTAATGTTGAAGGTACTCATTCTTATATAACAAATGGAGGTAATATAGAAGAAACAAAAAGCCAATCA
SC53        AAATCAGTAGGTAAAAGTTTACCTAATGGTGAAGGTACTCATTCTTATATAACAAATGGAGGTAATAGAGAAGAAACAAAAAGCCAATCA
TAB141      AAATCAGTAGGTAAAAGTTTACCTAATGGTGAAGGTACTCATTCTTATATAACAAATGGAGGTAATAGAGAAGAAACAAAAAGCCAATCA
TAB123      AAATCAGTAGGTAAAAGTTTACCTAATGGTGAAGGTACTCATTCTTATATAACAAATGGAGGTAATAGAGAAGAAACAAAAAGCCAATCA
AM1803      AAATCAGTAGGTAAAAGTTTACCTAATGGTGAAGGTACTCATTCTTATATAACAAATGGAGGTAATAGAGAAGAAACAAAAAGCCAATCA
AM1814      AAATTAGAAGGTAAAAGTTCACCTAATGGTGAAGGTACTCATTCTTATATGAGACATGGAGATAATAAAGAAGAAACAAAAAGCCAATCA
AM1802      AAATTAGAAGGTAAAAGTTCACCTAATGGTGAAGGTACTCATTCTTATATGAGACATGGAGATAATAAAGAAGAAACAAAAAGCCAATCA
AM1811      AAATCAGTAGGTAAAAGTTTACCTAATGGTGAAGGTACTCATTCTTATATAACAAATGGAGGTAATAGAGAAGAAACAAAAAGCCAATCA
SC50        AAATTAGAAGGTAAAAGTTCACCTAATGGTGAAGGTACTCATTCTTATATGAGACATGGAGATAATAAAGAAGAAACAAAAAGCCAATCA
SC56        AAATCAGTAGGTAAAAGTTTACCTAATGGTGAAGGTACTCATTCTTATATAACAAATGGAGGTAATAGAGAAGAAACAAAAAGCCAATCA
TAB153      AAATTAGAAGGTAAAAGTTCACCTAATGTTGAAGGTACTCATTCTTATATAACAAATGGAGGTAATAGAGAAGAAACAAAAAGCCAATCA
TAB154      AAATTAGAAGGTAAAAGTTCACCTAATGTTGAAGGTACTCATTCTTATATAACAAATGGAGGTAATAGAGAAGAAACAATAAGCCAATCA
TAB156      AAATTAGAAGGTAAAAGTTCACCTAATGGTGAAGGTACTCATTCTTATATGAGACATGGAGATAATAAAGAAGAAACAAAAAGCCAATCA
TAB136      AAATCAGTAGGTAAAAGTTTACCTAATGGTGAAGGTACTCATTCTTATATGAGACATGGAGATAATAAAGAAGAAACAAAAAGCCAATCA
AM1880      AAATCAGTAGGTAAAAGTTTACCTAATGGTGAAGGTACTCATTCTTATATGAGACATGGAGATAATAAAGAAGAAACAAAAAGCCAATCA
PF3D7_      AAATTAGAAGGTAAAAGTTCACCTAATGTTGAAGGTACTCATTCTTATATAACAAATGGAGGTAATATAGAAGAAACAAAAAGCCAATCA
PfSD01      AAATCAGTAGGTAAAAGTTTACCTAATGGTGAAGGTACTCATTCTTATATAACAAATGGAGGTAATAGAGAAGAAACAAAAAGCCAATCA
Pf7G8       AAATCAGTAGGTAAAAGTTTACCTAATGGTGAAGGTACTCATTCTTATATGAGACATGGAGATAATAAAGAAGAAACAAAAAGCCAATCA
FCR3 GENE   AAATTAGAAGGTAAAAGTTCACCTAATGTTGAAGGTACTCATTCTTATATAACAAATGGAGGTAATATAGAAGAAACAAAAAGCCAATCA
MS822 GENE  AAATTAGAAGGTAAAAGTTCACCTAATGTTGAAGGTACTCATTCTTATATAACAAATGGAGGTAATATAGAAGAAACAAAAAGCCAATCA
PfCD01_     AAATCAGTAGGTAAAAGTTTACCTAATGGTGAAGGTACTCATTCTTATATGAGACATGGAGATAATAAAGAAGAAACAAAAAGCCAATCA
PfKH01__    AAATCAGTAGGTAAAAGTTTACCTAATGGTGAAGGTACTCATTCTTATATAACAAATGGAGGTAATAGAGAAGAAACAAAAAGCCAATCA
PfDd2__     AAATCAGTAGGTAAAAGTTTACCTAATGGTGAAGGTACTCATTCTTATATAACAAATGGAGGTAATAGAGAAGAAACAAAAAGCCAATCA
PfSN01_     AAATTAGAAGGTAAAAGTTCACCTAATGTTGAAGGTACTCATTCTTATATAACAAATGGAGGTAATAGAGAAGAAACAAAAAGCCAATCA
PfGN01      AAATTAGAAGGTAAAAGTTCACCTAATGTTGAAGGTACTCATTCTTATATAACAAATGGAGGTAATAGAGAAGAAACAAAAAGCCAATCA
PfKE01      AAATTAGAAGGTAAAAGTTCACCTAATGTTGAAGGTACTCATTCTTATATAACAAATGGAGGTAATATAGAAGAAACAAAAAGCCAATCA
PfKH02      AAATTAGAAGGTAAAAGTTCACCTAATGTTGAAGGTACTCATTCTTATATAACAAATGGAGGTAATATAGAAGAAACAAAAAGCCAATCA
PfGB4       AAATTAGAAGGTAAAAGTTCACCTAATGTTGAAGGTACTCATTCTTATATAACAAATGGAGGTAATAGAGAAGAAACAAAAAGCCAATCA
PfGA01_     AAATTAGAAGGTAAAAGTTCACCTAATGTTGAAGGTACTCATTCTTATATAACAAATGGAGGTAATAGAGAAGAAACAAAAAGCCAATCA
PfHB3_      AAATCAGTAGGTAAAAGTTTACCTAATGGTGAAGGTACTCATTCTTATATGAGACATGGAGATAATAAAGAAGAAACAAAAAGCCAATCA
PfIT_       AAATTAGAAGGTAAAAGTTCACCTAATGTTGAAGGTACTCATTCTTATATAACAAATGGAGGTAATATAGAAGAAACAAAAAGCCAATCA
```

```
                  1450      1460      1470      1480      1490      1500      1510      1520      1530
             ....|....|....|....|....|....|....|....|....|....|....|....|....|....|....|....|....|....|
PF3D7 CDS    TATTCTGTAACTGACAATTATGTTGAGGAAGAAGCTGCAAGTTGCCCAGAAGGTGACCAAAATTGCATTGATCTTATAAAAGACGAATTT
MS822 mRNA   TATTCTGTAACTGACAATTATGTTGAGGAAGAAGCTGCAAGTTGCCCAGAAGGTGACCAAAATTGCATTGATCTTATAAAAGACGAATTT
FCR3 mRNA    TATTCTGTAACTGACAATTATGTTGAGGAAGAAGCTGCAAGTTGCCCAGAAGGTGACCAAGATTGCATTGATCCTATAAAAGACGAATTT
AM1898       CATTCTGTAACTGACAATTATGTTGAGGAAGAAGCTGCAAGTTGCCCAGAAGGTGACCAAGATTGCATTGATCTTATAAAAGACGAATTT
AA235        TATTCTGTAACTGACAATTATGTTGAGGAAGAAGCTGCAAGTTGCCCAGAAGGTGACCAAGATTGCATTGATCTTATAAAAGACGAATTT
AA243        TATTCTGTAACTGACAATTATGTTGAGGAAGAAGCTGCAAGTTGCCCAGAAGGTGACCAAGATTGCATTGATCTTATAAAAGACGAATTT
AA258        TATTCTGTAACTGACAATTATGTTGAGGAAGAAGCTGCAAGTTGCCCAGAAGGTGACCAAAATTGCATTGATCTTATAAAAGACGAATTT
AA264        TATTCTGTAACTGACAATTATGTTGAGGAAGAAGCTGCAAGTTGCCCAGAAGGTGACCAAGATTGCATTGATCTTATAAAAGACGAATTT
AM1737       TATTCTGTAACTGACAATTATGTTGAGGAAGAAGCTGCAAGTTGCCCAGAAGGTGACCAAGATTGCATTGATCTTATAAAAGACGAATTT
SC74         TATTCTGTAACTGACAATTATGTTGAGGAAGAAGCTGCAAGTTGCCCAGAAGGTGACCAAAATTGCATTGATCTTATAAAAGACGAATTT
TAB166       TATTCTGTAACTGACAATTATGTTGAGGAAGAAGCTGCAAGTTGCCCAGAAGGTGACCAAAATTGCATTGATCTTATAAAAGACGAATTT
TAB138       TATTCTGTAACTGACAATTATGTTGAGGAAGAAGCTGCAAGTTGCCCAGAAGGTGACCAAGATTGCATTGATCTTATAAAAGACGAATTT
TAB130       TATTCTGTAACTGACAATTATGTTGAGGAAGAAGCTGCAAGTTGCCCAGAAGGTGACCAAAATTGCATTGATCTTATAAAAGACGAATTT
TAB131       TATTCTGTAACTGACAATTATGTTGAGGAAGAAGCTGCAAGTTGCCCAGAAGGTGACCAAGATTGCATTGATCTTATAAAAGACGAATTT
TAB151       TATTCTGTAACTGACAATTATGTTGAGGAAGAAGCTGCAAGTTGCCCAGAAGGTGACCAAGATTGCATTGATCTTATAAAAGACGAATTT
TAB152       TATTCTGTAACTGACAATTATGTTGAGGAAGAAGCTGCAAGTTGCCCAGAAGGTGACCAAGATTGCATTGATCTTATAAAAGACGAATTT
SC52         TATTCTGTAACTGACAATTATGTTGAGGAAGAAGCTGCAAGTTGCCCAGAAGGTGACGAAGATTGCATTGATCTTATAAAAGACGAATTT
SC55         CATTCTGTAACTGACAATTATGTTGAGGAAGAAGCTGCAAGTTGCCCAGAAGGTGACCAAAATTGCATTGATCTTATAAAAGACGAATTT
SC03         TATTCTGTAACTGACAATTATGTTGAGGAAGAAGCTGCAAGTTGCCCAGAAGGTGACCAAAATTGCATTGATCTTATAAAAGACGAATTT
SC53         TATTCTGTAACTGACAATTATGTTGAGGAAGAAGCTGCAAGTTGCCCAGAAGGTGACCAAGATTGCATTGATCTTATAAAAGACGAATTT
TAB141       TATTCTGTAACTGACAATTATGTTGAGGAAGAAGCTGCAAGTTGCCCAGAAGGTGACCAAAATTGCATTGATCTTATAAAAGACGAATTT
TAB123       TATTCTGTAACTGACAATTATGTTGAGGAAGAAGCTGCAAGTTGCCCAGAAGGTGACCAAGATTGCATTGATCTTATAAAAGACGAATTT
AM1803       TATTCTGTAACTGACAATTATGTTGAGGAAGAAGCTGCAAGTTGCCCAGAAGGTGACCAAGATTGCATTGATCTTATAAAAGACGAATTT
AM1814       CATTCTGTAACTGACCATGATGTTGAGGAAGAAGCTGCAAGTTGCCCAGAAGGTGACCAAGATTGCATTGATCTTATAAAAGACGAATTT
AM1802       CATTCTGTAAGTGAAATTTCTGCAGAGGAAGAAGCTGCGAGTTGCCCAGAAGGCGACCAAGGTTGCATTGATCCTACAAAACAAACATTT
AM1811       TATTCTGTAACTGACAATTATGTTGAGGAAGAAGCTGCAAGTTGCCCAGAAGGTGACCAAGATTGCATTGATCTTATAAAAGACGAATTT
SC50         CATTCTGTAAGTGAAATTTCTGCAGAGGAAGAAGCTGCGAGTTGCCCAGAAGGCGACCAAGGTTGCATTGATCCTACAAAACAAACATTT
SC56         TATTCTGTAACTGACAATTATGTTGAGGAAGAAGCTGCAAGTTGCCCAGAAGGTGACCAAGATTGCATTGATCTTATAAAAGACGAATTT
TAB153       TATTCTGTAACTGACAATTATGTTGAGGAAGAAGCTGCAAGTTGCCCAGAAGGTGACCAAGATTGCATTGATCTTATAAAAGACGAATTT
TAB154       TATTCTGTAACTGACAATTATGTTGAGGAAGAAGCTGCAAGTTGCCCAGAAGGTGACCAAGATTGCATTGATCTTATAAAAGACGAATTT
TAB156       CATTCTGTAAGTGAAATTTCTGCAGAGGAAGAAGCTGCGAGTTGCCCAGAAGGCGACCAAGGTTGCATTGATCCTACAAAACAAACATTT
TAB136       CATTCTGTAACTGACCATGATGTTGAGGAAGAAGCTGTAAGTTGCCCAGAGGGTGAAGAGAATTGCATTGATCCTATAAAAGACGAATTT
AM1880       CATTCTGTAACTGACCATGATGTTGAGGAAGAAGCTGTAAGTTGCCCAGAGGGTGAACAGAATTGCATTGATCTTATAAAAGACGAATTT
PF3D7_       TATTCTGTAACTGACAATTATGTTGAGGAAGAAGCTGCAAGTTGCCCAGAAGGTGACCAAAATTGCATTGATCTTATAAAAGACGAATTT
PfSD01       TATTCTGTAACTGACAATTATGTTGAGGAAGAAGCTGCAAGTTGCCCAGAAGGTGACCAAGATTGCATTGATCTTATAAAAGACGAATTT
Pf7G8        CATTCTGTAAGTGAAATTTCTGCAGAGGAAGAAGCTGCGAGTTGCCCAGAAGGCGACCAAAGTTGCATTGATCCTACAAAACAAACATTT
FCR3 GENE    TATTCTGTAACTGACAATTATGTTGAGGAAGAAGCTGCAAGTTGCCCAGAAGGTGACCAAGATTGCATTGATCCTATAAAAGACGAATTT
MS822 GENE   TATTCTGTAACTGACAATTATGTTGAGGAAGAAGCTGCAAGTTGCCCAGAAGGTGACCAAAATTGCATTGATCTTATAAAAGACGAATTT
PfCD01_      CATTCTGTAAGTGAAATTTCTGCAGAGGAAGAAGCTGCGAGTTGCCCAGAAGGCGACCAAAGTTGCATTGATCCTACAAAACAAACATTT
PfKH01__     TATTCTGTAACTGACAATTATGTTGAGGAAGAAGCTGCAAGTTGCCCAGAAGGTGACCAAGATTGCATTGATCTTATAAAAGACGAATTT
PfDd2__      TATTCTGTAACTGACAATTATGTTGAGGAAGAAGCTGCAAGTTGCCCAGAAGGTGACCAAGATTGCATTGATCTTATAAAAGACGAATTT
PfSN01_      TATTCTGTAACTGACAATTATGTTGAGGAAGAAGCTGCAAGTTGCCCAGAAGGTGACCAAGATTGCATTGATCTTATAAAAGACGAATTT
PfGN01       TATTCTGTAACTGACAATTATGTTGAGGAAGAAGCTGCAAGTTGCCAAGAAGGTGACCAAAATTGCATTGATCTTATAAAAGACGAATTT
PfKE01       TATTCTGTAACTGACAATTATGTTGAGGAAGAAGCTGCAAGTTGCCCAGAAGGTGACCAAGATTGCATTGATCTTATAAAAGACGAATTT
PfKH02       TATTCTGTAACTGACAATTATGTTGAGGAAGAAGCTGCAAGTTGCCCAGAAGGTGACCAAGATTGCATTGATCTTATAAAAGACGAATTT
PfGB4        TATTCTGTAACTGACAATTATGTTGAGGAAGAAGCTGCAAGTTGCCCAGAAGGTGACCAAGATTGCATTGATCTTATAAAAGACGAATTT
PfGA01_      TATTCTGTAACTGACAATTATGTTGAGGAAGAAGCTGCAAGTTGCCCAGAAGGTGACCAAGATTGCATTGATCTTATAAAAGACGAATTT
PfHB3_       CATTCTGTAACTGACCATGATGTTGAGGAAGAAGCTGTAAGTTGCCCAGAGGGTGAAGAGAATTGCATTGATCCTACAAAACAAATATTT
PfIT_        TATTCTGTAACTGACAATTATGTTGAGGAAGAAGCTGCAAGTTGCCCAGAAGGTGACCAAGATTGCATTGATCCTATAAAAGACGAATTT
```

```
                 1540      1550      1560      1570      1580      1590      1600      1610      1620
             ....|....|....|....|....|....|....|....|....|....|....|....|....|....|....|....|....|....|
PF3D7 CDS    ATCATAGAAGGTGGTCGTTTTATCCGTACATCAATTAATGATGTTCCACAAATGGAAGCTATAACTAACAAATATATGCCTGGATCCATT
MS822 mRNA   ATCATAGAAGGTGGTCGTTTTATCCGTACATCAATTAATGATGTTCCACAAATGGAAGCTATAACTAACAAATATATGCCTGGATCCATT
FCR3 mRNA    ATCATAGAAGGTGGTCGTGTTATCCGTACATCAATTAATGATGTTCCACAAATGGAAGCTATAACTAACAAATACATGCCTGGATCCATT
AM1898       ATCATAGAAGGTGGTCGTTTTATCCGTACATCAATTAATGATGTTCCACAAATGGAAGCTATAACTAACAAATATATGCCTGGATCCATT
AA235        ATCATAGAAGGTGGTCGTGTTATCCGTACATCAATTAATGATGTTCCACAAATGGAAGCTATAACTAACAAATATATGCCTGGATCCATT
AA243        ATCATAGAAGGTGGTCGTTTTATCCGTACATCAATTAATGATGTTCCACAAATGGAAGCTATAACTAACAAATATATGCCTGGATCCATT
AA258        ATCATAGAAGGTGGTCGTGTTATCCGTACATCAATTAATGATGTTCCACAAATGGAAGCTATAACTAACAAATATATGCCTGGATCCATT
AA264        ATCATAGAAGGTGGTCGTTTTATCCGTACATCAATTAATGATGTTCCACAAATGGAAGCTATAACTAACAAATATATGCCTGGATCCATT
AM1737       ATCATAGAAGGTGGTCGTTTTATCCGTACATCAATTAATGATGTTCCACAAATGGAAGCTATAACTAACAAATATATGCCTGGATCCATT
SC74         ATCATAGAAGGTGGTCGTTTTATCCGTACATCAATTAATGATGTTCCACAAATGGAAGCTATAACTAACAAATATATGCCTGGATCCATT
TAB166       ATCATAGAAGGTGGTCGTTTTATCCGTACATCAATTAATGATGTTCCACAAATGGAAGCTATAACTAACAAATATATGCCTGGATCCATT
TAB138       ATCATAGAAGGTGGTCGTGTTATCCGTACATCAATTAATGATGTTCCACAAATGGAAGCTATAACTAACAAATATATGCCTGGATCCATT
TAB130       ATCATAGAAGGTGGTCGTTTTATCCGTACATCAATTAATGATGTTCCACAAATGGAAGCTATAACTAACAAATATATGCCTGGATCCATT
TAB131       ATCATAGAAGGTGGTCGTTTTATCCGTACATCAATTAATGATGTTCCACAAATGGAAGCTATAACTAACAAATATATGCCTGGATCCATT
TAB151       ATCATAGAAGGTGGTCGTTTTATCCGTACATCAATTAATGATGTTCCACAAATGGAAGCTATAACTAACAAATATATGCCTGGATCCATT
TAB152       ATCATAGAAGGTGGTCGTTTTATCCGTACATCAATTAATGATGTTCCACAAATGGAAGCTATAACTAACAAATATATGCCTGGATCCATT
SC52         ATCATAGAAGGTGGTCGTGTTATCCGTACATCAATTAATGATGTTCCACAAATGGAAGCTATAACTAACAAATATATGCCTGGATCCATT
SC55         ATCATAGAAGGTGGTCGTTTTATCCGTACATCAATTAATGATGTTCCACAAATGGAAGCTATAACTAACAAATATATGCCTGGATCCATT
SC03         ATCATAGAAGGTGGTCGTGTTATCCGTACATCAATTAATGATGTTCCACAAATGGAAGCTATAACTAACAAATATATGCCTGGATCCATT
SC53         ATCATAGAAGGTGGTCGTTTTATCCGTACATCAATTAATGATGTTCCACAAATGGAAGCTATAACTAACAAATATATGCCTGGATCCATT
TAB141       ATCATAGAAGGTGGTCGTGTTATCCGTACATCAATTAATGATGTTCCACAAATGGAAGCTATAACTAACAAATATATGCCTGGATCCATT
TAB123       ATCATAGAAGGTGGTCGTGTTATCCGTACATCAATTAATGATGTTCCACAAATGGAAGCTATAACTAACAAATATATGCCTGGATCCATT
AM1803       ATCATAGAAGGTGGTCGTGTTATCCGTACATCAATTAATGATGTTCCACAAATGGAAGCTATAACTAACAAATATATGCCTGGATCCATT
AM1814       ATCATAGAAGGTGGTCGTTTTATCCGTACATCAATTAATGATGTTCCACAAATGGAAGCTATAACTAACAAATATATGCCTGGATCCATT
AM1802       GTCATAGAAGGAAATACTGTAAGAAGTATGGAAATTGGTGATGTTCCAAAAATGGAAGCTGTTACTAGTATAGATTTGCCCAAAGCCATT
AM1811       ATCATAGAAGGTGGTCGTGTTATCCGTACATCAATTAATGATGTTCCACAAATGGAAGCTATAACTAACAAATATATGCCTGGATCCATT
SC50         GTCATAGAAGGAAATACTGTAAGAAGTATGGAAATTGGTGATGTTCCAAAAATGGAAGCTGTTACTAGTATAGATTTGCCCAAAGCCATT
SC56         ATCATAGAAGGTGGTCGTGTTATCCGTACATCAATTAATGATGTTCCACAAATGGAAGCTATAACTAACAAATATATGCCTGGATCCATT
TAB153       ATCATAGAAGGTGGTCGTGTTATCCGTACATCAATTAATGATGTTCCACAAATGGAAGCTATAACTAACAAATATATGCCTGGATCCATT
TAB154       ATCATAGAAGGTGGTCGTTTTATCCGTACATCAATTAATGATGTTCCACAAATGGAAGCTATAACTAACAAATATATGCCTGGATCCATT
TAB156       GTCATAGAAGGAAATACTGTAAGAAGTATGGAAATTGGTGATGTTCCAAAAATGGAAGCTGTTACTAGTATAGATTTGCCCAAAGCCATT
TAB136       ATCATAGAAGGTGGTAGTTTTATCCGTACATCAATTAATGATGTTCCACAAATGGAAGCTATAACTAGCATAGACAGCCCCAAAGGCATT
AM1880       ATCATAGAAGGTGGTCGTTTTATCCGTACATCAATTAATGATGTTCCACAAATGGAAGCTATAACTAACAAATATATGCCTGGATCCATT
PF3D7_       ATCATAGAAGGTGGTCGTTTTATCCGTACATCAATTAATGATGTTCCACAAATGGAAGCTATAACTAACAAATATATGCCTGGATCCATT
PfSD01       ATCATAGAAGGTGGTCGTGTTATCCGTACATCAATTAATGATGTTCCACAAATGGAAGCTATAACTAACAAATATATGCCTGGATCCATT
Pf7G8        GTCATAGAAGGAAATACTGTAAGAAGTATGGAAATTGGTGATGTTCCAAAAATGGAAACTGTTACTAGTATAGATTTGCCCAAAGCCATT
FCR3 GENE    ATCATAGAAGGTGGTCGTGTTATCCGTACATCAATTAATGATGTTCCACAAATGGAAGCTATAACTAACAAATACATGCCTGGATCCATT
MS822 GENE   ATCATAGAAGGTGGTCGTTTTATCCGTACATCAATTAATGATGTTCCACAAATGGAAGCTATAACTAACAAATATATGCCTGGATCCATT
PfCD01_      GTCATAGAAGGAAATACTGTAAGAAGTATGGAAATTGGTGATGTTCCAAAAATGGAAACTGTTACTAGTATAGATTTGCCCAAAGCCATT
PfKH01__     ATCATAGAAGGTGGTCGTTTTATCCGTACATCAATTAATGATGTTCCACAAATGGAAGCTATAACTAACAAATATATGCCTGGATCCATT
PfDd2__      ATCATAGAAGGTGGTCGTTTTATCCGTACATCAATTAATGATGTTCCACAAATGGAAGCTATAACTAACAAATATATGCCTGGATCCATT
PfSN01_      ATCATAGAAGGTGGTCGTTTTATCCGTACATCAATTAATGATGTTCCACAAATGGAAGCTATAACTAACAAATATATGCCTGGATCCATT
PfGN01       ATCATAGAAGGTGGTCGTTTTATCCGTACATCAATTAATGATGTTCCACAAATGGAAGCTATAACTAACAAATATATGCCTGGATCCATT
PfKE01       ATCATAGAAGGTGGTCGTGTTATCCGTACATCAATTAATGATGTTCCACAAATGGAAGCTATAACTAACAAATATATGCCTGGATCCATT
PfKH02       ATCATAGAAGGTGGTCGTTTTATCCGTACATCAATTAATGATGTTCCACAAATGGAAGCTATAACTAACAAATATATGCCTGGATCCATT
PfGB4        ATCATAGAAGGTGGTCGTTTTATCCGTACATCAATTAATGATGTTCCACAAATGGAAGCTATAACTAACAAATATATGCCTGGATCCATT
PfGA01_      ATCATAGAAGGTGGTCGTTTTATCCGTACATCAATTAATGATGTTCCACAAATGGAAGCTATAACTAACAAATATATGCCTGGATCCATT
PfHB3_       GTCATAGAAGGTGATCATGTTAGGAGTATATCAATTAATAACATTCCAAAAATGGAAGCTGTTACTAGCATAGACAGCCCCAAAGGCATT
PfIT_        ATCATAGAAGGTGGTCGTGTTATCCGTACATCAATTAATGATGTTCCACAAATGGAAGCTATAACTAACAAATACATGCCTGGATCCATT
```

```
                1630      1640      1650      1660      1670      1680      1690      1700      1710
           ....|....|....|....|....|....|....|....|....|....|....|....|....|....|....|....|....|....|
PF3D7 CDS  TATGAAAATCCTTCATCTGGTAGTTCACATATTGAAGATAAAGGACAATTAAAAGATTTCACTATTGAAACAGTCAAATATACATCAGAT
MS822 mRNA TATGAAAATCCTTCATCTGGTAGTTCACATATTGAAGATAAAGGACAATTAAAAGATTTCACTATTGAAACAGTCAAATATACATCAGAT
FCR3 mRNA  TATGAAAATCCTTCATCTGGTAGTTCACATATTGAAGATAAAGGACAATTAAAAGATTTCACTATTGAAACAGTCAAATATACATCAGAT
AM1898     TATGAAAATCCTTCATCTGGTAGTTCACATATTGAAGATAAAGGACAATTAAAAGATTTCACTATTGAAACAGTCAAATATACATCAGAT
AA235      TATGAAAATCCTTCATCTGGTAGTTCACATATTGAAGATAAAGGACAATTATTAGATTTCACTATTGAAACAGTCAAATATACATCAGAT
AA243      TATGAAAATCCTTCATCTGGTAGTTCACATATTGAAGATAAAGGACAATTATTAGATTTCACTATTGAAACAGTCAAATATACATCAGAT
AA258      TATGAAAATCCTTCATCTGGTAGTTCACATATTGAAGATAAAGGACAATTATTAGATTTCACTATTGAAACAGTCAAATATACATCAGAT
AA264      TATGAAAATCCTTCATCTGGTAGTTCACATATTGAAGATAAAGGACAATTAAAAGATTTCACTATTGAAACAGTCAAATATACATCAGAT
AM1737     TATGAAAATCCTTCATCTGGTAGTTCACATATTGAAGATAAAGGACAATTAAAAGATTTCACTATTGAAACAGTCAAATATACATCAGAT
SC74       TATGAAAATCCTTCATCTGGTAGTTCACATATTGAAGATAAAGGACAATTAAAAGATTTCACTATTGAAACAGTCAAATATACATCAGAT
TAB166     TATGAAAATCCTTCATCTGGTAGTTCACATATTGAAGATAAAGGACAATTATTAGATTTCACCATTCAACCGGTTATATATCCATCTGAT
TAB138     TATGAAAATCCTTCATCTGGTAGTTCACATATTGAAGATAAAGGACAATTAAAAGATTTCACTATTGAAACAGTCAAATATACATCAGAT
TAB130     TATGAAAATCCTTCATCTGGTAGTTCACATATTGAAGATAAAGGACAATTAAAAGATTTCACTATTGAAACAGTCAAATATACATCAGAT
TAB131     TATGAAAATCCTTCATCTGGTAGTTCACATATTGAAGATAAAGGACAATTAAAAGATTTCACTATTGAAACAGTCAAATATACATCAGAT
TAB151     TATGAAAATCCTTCATCTGGTAGTTCACATATTGAAGATAAAGGACAATTAAAAGATTTCACTATTGAAACAGTCAAATATACATCAGAT
TAB152     TATGAAAATCCTTCATCTGGTAGTTCACATATTGAAGATAAAGGACAATTAAAAGATTTCACTATTGAAACAGTCAAATATACATCAGAT
SC52       TATGAAAATCCTTCATCTGGTAGTTCACATATTGAAGATAAAGGGCAATTATTAGATTTCACTATTGAAACAGTCAAATATACATCAGAT
SC55       TATGAAAATCCTTCATCTGGTAGTTCACATATTGAAGATAAAGGACAATTATTAGATTTCACTATTGAAACAGTCAAATATACATCAGAT
SC03       TATGAAAATCCTTCATCTGGTAGTTCACATATTGAAGATAAAGGACAATTAAAAGATTTCACTATTGAAACAGTCAAATATACATCAGAT
SC53       TATGAAAATCCTTCATCTGGTAGTTCACATATTGAAGATAAAGGACAATTAAAAGATTTCACTATTGAAACAGTCAAATATACATCAGAT
TAB141     TATGAAAATCCTTCATCTGGTAGTTCACATATTGAAGATAAAGGACAATTAAAAGATTTCACTATTGAAACAGTCAAATATACATCAGAT
TAB123     TATGAAAATCCTTCATCTGGTAGTTCACATATTGAAGATAAAGGACAATTAAAAGATTTCACTATTGAAACAGTCAAATATACATCAGAT
AM1803     TATGAAAATCCTTCATCTGGTAGTTCACATATTGAAGATAAAGGACAATTAAAAGATTTCACTATTGAAACAGTCAAATATACATCAGAT
AM1814     TATGAAAATCCTTCATCTGGTAGTTCACATATTGAAGATAAAGGACAATTAAAAGATTTCACTATTGAAACAGTCAAATATACATCAGAT
AM1802     AATGAAGTTCCTTCATCTGTTGATGCACTTGTTGAAGATAAAGGACCATTATTAGATTTCACTATTCAACCGGTTAAATATACATCTGTG
AM1811     TATGAAAATCCTTCATCTGGTAGTTCACATATTGAAGATAAAGGACAATTAAAAGATTTCACTATTGAAACAGTCAAATATACATCAGAT
SC50       AATGAAGTTCCTTCATCTGTTGATGCACTTGTTGAAGATAAAGGACCATTATTAGATTTCACTATTCAACCGGTTAAATATACATCTGTG
SC56       TATGAAAATCCTTCATCTGGTAGTTCACATATTGAAGATAAAGGACAATTAAAAGATTTCACTATTGAAACAGTCAAATATACATCAGAT
TAB153     TATGAAAATCCTTCATCTGGTAGTTCACATATTGAAGATAAAGGACAATTAAAAGATTTCACTATTGAAACAGTCAAATATACATCAGAT
TAB154     TATGAAAATCCTTCATCTGGTAGTTCACATATTGAAGATAAAGGACAATTAAAAGATTTCACTATTGAAACAGTCAAATATACATCAGAT
TAB156     AATGAAGTTCCTTCATCTGTTGATGCACTTGTTGAAGATAAAGGACCATTATTAGATTTCACTATTCAACCGGTTAAATATACATCTGTG
TAB136     AATGAAGTTCCTTCATCTGTTGGTGCACGTGTTGAAGATAAAGGGCAATTATTAGATTTCACTATTGAGCCGGTTATATATCAATCTGAT
AM1880     TATGAAAATCCTTCATCTGGTAGTTCACATATTGAAGATAAAGGACAATTAAAAGATTTCACTATTGAAACAGTCAAATATACATCAGAT
PF3D7_     TATGAAAATCCTTCATCTGGTAGTTCACATATTGAAGATAAAGGACAATTAAAAGATTTCACTATTGAAACAGTCAAATATACATCAGAT
PfSD01     TATGAAAATCCTTCATCTGGTAGTTCACATATTGAAGATAAAGGACAATTAAAAGATTTCACTATTGAAACAGTCAAATATACATCAGAT
Pf7G8      AATGAAGTTCCTTCATCTGTTGGTGCACATCTTGAAGATAAAGAACAATTATTAGATTTCACCATTCAACCGGTTATATATCCATCTGAT
FCR3 GENE  TATGAAAATCCTTCATCTGGTAGTTCACATATTGAAGATAAAGGACAATTAAAAGATTTCACTATTGAAACAGTCAAATATACATCAGAT
MS822 GENE TATGAAAATCCTTCATCTGGTAGTTCACATATTGAAGATAAAGGACAATTAAAAGATTTCACTATTGAAACAGTCAAATATACATCAGAT
PfCD01_    AATGAAGTTCCTTCATCTGTTGGTGCACATCTTGAAGATAAAGAACAATTATTAGATTTCACCATTCAACCGGTTATATATCCATCTGAT
PfKH01__   TATGAAAATCCTTCATCTGGTAGTTCACATATTGAAGATAAAGGACAATTAAAAGATTTCACTATTGAAACAGTCAAATATACATCAGAT
PfDd2__    TATGAAAATCCTTCATCTGGTAGTTCACATATTGAAGATAAAGGACAATTAAAAGATTTCACTATTGAAACAGTCAAATATACATCAGAT
PfSN01_    TATGAAAATCCTTCATCTGGTAGTTCACATATTGAAGATAAAGGACAATTAAAAGATTTCACTATTGAAACAGTCAAATATACATCAGAT
PfGN01     TATGAAAATCCTTCATCTGGTAGTTCACATATTGAAGATAAAGGGCAATTATTAGATTTCACTATTGAAACAGTCAAATATACATCAGAT
PfKE01     TATGAAAATCCTTCATCTGGTAGTTCACATATTGAAGATAAAGGACAATTAAAAGATTTCACTATTGAAACAGTCAAATATACATCAGAT
PfKH02     TATGAAAATCCTTCATCTGGTAGTTCACATATTGAAGATAAAGGACAATTAAAAGATTTCACTATTGAAACAGTCAAATATACATCAGAT
PfGB4      TATGAAAATCCTTCATCTGGTAGTTCACATATTGAAGATAAAGGGCAATTATTAGATTTCACTATTGAAACAGTCAAATATACATCAGAT
PfGA01_    TATGAAAATCCTTCATCTGGTAGTTCACATATTGAAGATAAAGGACAATTAAAAGATTTCACTATTGAAACAGTCAAATATACATCAGAT
PfHB3_     AATGAAGTTCCTTCATCTGTTGGTGCACGTGTTGAAGATAAAGGGCAATTATTAGATTTCACTATTGAGCCGGTTATATATCAATCTGAT
PfIT_      TATGAAAATCCTTCATCTGGTAGTTCACATATTGAAGATAAAGGACAATTAAAAGATTTCACTATTGAAACAGTCAAATATACATCAGAT
```

```
                  1720      1730      1740      1750      1760      1770      1780      1790      1800
              ....|....|....|....|....|....|....|....|....|....|....|....|....|....|....|....|....|....|
PF3D7 CDS     GGACTTAATGGATTTGATCATGAAGAACCAGTGACA---GATCCTAAATTATTACATGGTGAACAACTTATAGAATTTACGGGAAAAGTT
MS822 mRNA    GGACTTAATGGATTTGATCATGAAGAACCAGTGACA---GATCCTAAATTATTACATGGTGAACAACTTATAGAATTTACGGGAAAAGTT
FCR3 mRNA     GGACTTAATGGATTTGATCATGAAGTAGCAGTGACA---GATCCTAAATTATTACCTGGTGAACAACTTATAGCACTTACGGGAGAAGTT
AM1898        GGACTTAATGGATTTGATCATGAAGTAGCAGTGACA---GATCCTAAATTATTACCTGGTGAACAACTTATAGCACTTACGGGAAAAGTT
AA235         GGACTTAATGGATTTGTTCATGAAGAACCAGTGACA---GATCCTAAATTATTACCTGGTGAACAACTTATAGCACTTACGGGAAAAGTT
AA243         GGACTTAATGGATTTGATCATGAAGTAGCAGTGACA---GATCCTAAATTATTACCTGGTGAACAACTTATAGCACTTACGGGAGAAGTT
AA258         GGACTTAATGGATTTGATCATGAAGTAGCAGTGACA---GATCCTAAATTATTACCTGGTGAACAACTTATAGCACTTACGGGAGAAGTT
AA264         GGACTTAATGGATTTGATCATGAAGTAGCAGTGACA---GATCCTAAATTATTACCTGGTGAACAACTTATAGCACTTACGGGAGAAGTT
AM1737        GGACTTAATGGATTTGATCATGAAGTAGCAGTGACA---GATCCTAAATTATTACCTGGTGAACAACTTATAGCACTTACGGGAGAAGTT
SC74          GGACTTAATGGATTTGATCATGAAGTAGCAGTGACA---GATCCTAAATTATTACCTGGTGAACAACTTATAGCACTTACGGGAGAAGTT
TAB166        GAAAACGATGCATTTGTTCATGAAGTAGCAGTGACA---GATCCTAATTTATTACCTGGTCAAAAACTTATAACACCTACAGGAGAAGAT
TAB138        GGACTTAATGGATTTGTTCATGAAGAACCAGTGACA---GATCCTAAATTATTACCTGGTGAACAACTTATAGCACTTACGGGAAAAGTT
TAB130        GGACTTAATGGATTTGATCATGAAGTAGCAGTGACA---GATCCTAAATTATTACCTGGTGAACAACTTATAGCACTTACGGGAGAAGTT
TAB131        GGACTTAATGGATTTGATCATGAAGTAGCAGTGACA---GATCCTAAATTATTACCTGGTGAACAACTTATAGCACTTACGGGAGAAGTT
TAB151        GGACTTAATGGATTTGATCATGAAGTAGCAGTGACA---GATCCTAAATTATTACCTGGTGAACAACTTATAGCACTTACGGGAGAAGTT
TAB152        GGACTTAATGGATTTGATCATGAAGTAGCAGTGACA---GATCCTAAATTATTACCTGGTGAACAACTTATAGCACTTACGGGAAAAGTT
SC52          GGACTCAATGGATTTGTTCATGAAGAACCAGTGACA---GATCCTAAATTATTACCTGGTGAACAACTTATAGCACTTACGGGAAAAGTT
SC55          GGACTTAATGGATATGTTCATGAAGAACCAGTGACA---GATCCTAAATTATTACCTGGTGAACAACTTATAGCACTTACGGGAAAAGTT
SC03          GGACTTAATGGATTTGATCATGAAGTAGCAGTGACA---GATCCTAAATTATTACCTGGTGAACAACTTATAGCACTTACGGGAGAAGTT
SC53          GGACTTAATGGATTTGATCATGAAGTAGCAGTGACA---GATCCTAAATTATTACCTGGTGAACAACTTATAGCACTTACGGGAAAAGTT
TAB141        GGACTTAATGGATTTGATCATGAAGTAGCAGTGACA---GATCCTAAATTATTACCTGGTGAACAACTTATAGCACTTACGGGAGAAGTT
TAB123        GGACTTAATGGATTTGATCATGAAGTAGCAGTGACA---GATCCTAAATTATTACCTGGTGAACAACTTATAGCACTTACGGGAGAAGTT
AM1803        GGACTTAATGGATTTGATCATGAAGTAGCAGTGACA---GATCCTAAATTATTACCTGGTGAACAACTTATAGCACTTACGGGAGAAGTT
AM1814        GGACTTAATGGATTTGTTCATGAAGAACCAGTGACA---GATCCTAAATTATTACCTGGTGAACAACTTATAGCACTTACGGGAAAAGTT
AM1802        GATAACTATGCATTTGTTCATGAAGAACCAGTGACA---GATCCTAAATTATTACATGGTGAACAACTTATAGAATTTACGGGGGATGGA
AM1811        GGACTTAATGGATTTGATCATGAAGTAGCAGTGACA---GATCCTAAATTATTACCTGGTGAACAACTTATAGCACTTACGGGAGAAGTT
SC50          GATAACTATGCATTTGTTCATGAAGAACCAGTGACA---GATCCTAAATTATTACATGGTGAACAACTTATAGAATTTACGGGGGATGGA
SC56          GGACTTAATGGATTTGATCATGAAGTAGCAGTGACA---GATCCTAAATTATTACCTGGTGAACAACTTATAGCACTTACGGGAGAAGTT
TAB153        GGACTTAATGGATTTGATCATGAAGTAGCAGTGACA---GATCCTAAATTATTACCTGGTGAACAACTTATAGCACTTACGGGAGAAGTT
TAB154        GGACTTAATGGATTTGATCATGAAGTAGCAGTGACA---GATCCTAAATTATTACCTGGTGAACAACTTATAGCACTTACGGGAGAAGTT
TAB156        GATAACTATGCATTTGTTCATGAAGAACCAGTGACA---GATCCTAAATTATTACATGGTGAACAACTTATAGAATTTACGGGGGATGGA
TAB136        GAAAACAATGGATATGTTCATGAAGAACCAGTGACA---GATCCTAAATTATTACCTGGTGAACAACTTATAGCACTTACGGGAAAAGTT
AM1880        GGACTTAATGGATATGTTCATGAAGAACCAGTGACA---GATCCTAAATTATTACCTGGTGAACAACTTATAGCACTTACGGGAAAAGTT
PF3D7_        GGACTTAATGGATTTGATCATGAAGAACCAGTGACA---GATCCTAAATTATTACATGGTGAACAACTTATAGAATTTACGGGAAAAGTT
PfSD01        GGACTTAATGGATTTGATCATGAAGTAGCAGTGACA---GATCCTAAATTATTACCTGGTGAACAACTTATAGCACTTACGGGAGAAGTT
Pf7G8         GAAAACGATGCATTTGTTCATGAAGTAGCAGTGACATACGATCCTAATTTATTACCTGGTCAAAAACTTATAACACCTACAGGAGAAGAT
FCR3 GENE     GGACTTAATGGATTTGATCATGAAGTAGCAGTGACA---GATCCTAAATTATTACCTGGTGAACAACTTATAGCACTTACGGGAGAAGTT
MS822 GENE    GGACTTAATGGATTTGATCATGAAGAACCAGTGACA---GATCCTAAATTATTACATGGTGAACAACTTATAGAATTTACGGGAAAAGTT
PfCD01_       GAAAACGATGCATTTGTTCATGAAGTAGCAGTGACATACGATCCTAATTTATTACCTGGTCAAAAACTTATAACACCTACAGGAGAAGAT
PfKH01__      GGACTTAATGGATTTGATCATGAAGTAGCAGTGACA---GATCCTAAATTATTACCTGGTGAACAACTTATAGCACTTACGGGAGAAGTT
PfDd2__       GGACTTAATGGATTTGATCATGAAGTAGCAGTGACA---GATCCTAAATTATTACCTGGTGAACAACTTATAGCACTTACGGGAAAAGTT
PfSN01_       GGACTTAATGGATTTGTTCATGAAGAACCAGTGACA---GATCCTAAATTATTACCTGGTGAACAACTTATAGCACTTACGGGAAAAGTT
PfGN01        GGACTTAATGGATTTGATCATGAAGTAGCAGTGACA---GATCCTAAATTATTACCTGGTGAACAACTTATAGCACTTACGGGAGAAGTT
PfKE01        GGACTTAATGGATTTGATCATGAAGTAGCAGTGACA---GATCCTAAATTATTACCTGGTGAACAACTTATAGCACTTACGGGAGAAGTT
PfKH02        GGACTTAATGGATTTGATCATGAAGTAGCAGTGACA---GATCCTAAATTATTACCTGGTGAACAACTTATAGCACTTACGGGAGAAGTT
PfGB4         GGACTTAATGGATTTGATCATGAAGTAGCAGTGACA---GATCCTAAATTATTACCTGGTGAACAACTTATAGCACTTACGGGAGAAGTT
PfGA01_       GGACTTAATGGATTTGTTCATGAAGAACCAGTGACA---GATCCTAAATTATTACCTGGTGAACAACTTATAGCACTTACGGGAGAAGTT
PfHB3_        GAAAACAATGGATTTGTTCATGAAGAACCAGTGACA---GATCCTAAATTATTACCTGGTGAACAACTTATAGCACTTACGGGAAAAGTT
PfIT_         GGACTTAATGGATTTGATCATGAAGTAGCAGTGACA---GATCCTAAATTATTACCTGGTGAACAACTTATAGCACTTACGGGAGAAGTT
```

1810 1820 1830 1840 1850 1860 1870 1880 1890

PF3D7 CDS	TATGAATCTCTACCTCATCATAATGATATTGCGTTGCATGGTAGTCCAATACCACGTCGACGTTTTTCAAGAAGCTATCCATTAAATACT
MS822 mRNA	TATGAATCTCTACCTCATCATAATGATATTGCGTTGCATGGTAGTCCAATACCACGTCGACGTTTTTCAAGAAGCTATCCATTAAATACT
FCR3 mRNA	TATGAATCTCTACCTGATCATAATGAAATTGTCTTTTATGGTAGTCCAATACCACATCGACGTTTTGCAAGAAGCTATCCATTAAATACT
AM1898	TATGAATCTCTACCTCATCATAATGATATTGCGTTGCATGGTAGTCCAATACCACATCGACGTTTTGCAAGAAGCTATCCATTAAATACT
AA235	TATGAATCTCTACCTCATCATAATGATATTGCGTTGCATGGTAATCCAATACCACATCGACGTTTTGCAAGAAGCTATCCATTAAATACT
AA243	TATGAATCTCTACCTGATCATAATGAAATTGCGTTGCATGGTAATCCAATACCACATCGACGTTTTTCAAGAAGCTATCCATTAAATACT
AA258	TATGAATCTCTACCTGATCATAATGAAATTGCGTTGCATGGTAATCCAATACCACATCGACGTTTTTCAAGAAGCTATCCATTAAATACT
AA264	TATGAATCTCTACCTGATCATAATGAAATTGTCTTTTATGGTAGTCCAATACCACATCGACGTTTTGCAAGAAGCTATCCATTAAATACT
AM1737	TATGAATCTCTACCTGATCATAATGAAATTGTCTTTTATGGTAGTCCAATACCACATCGACGTTTTGCAAGAAGCTATCCATTAAATACT
SC74	TATGAATCTCTACCTGATCATAATGAAATTGCGTTGCATGGTAATCCAATACCACATCGACGTTTTTCAAGAAGCTATCCATTAAATACT
TAB166	TATACAGCTCTACCAGATTATAATGAAATTGCGTTGCATGGTAATCCAATACCACATCGACGTTTTTCAAGAAGCTATCCATTAAATACT
TAB138	TATGAATCTCTACCTCATCATAATGATATTGCGTTGCATGGTAGTCCAATACCACATCGACGTTTTGCAAGAAGCTATCCATTAAATACT
TAB130	TATGAATCTCTACCTGATCATAATGAAATTGTCTTTTATGGTAGTCCAATACCACATCGACGTTTTGCAAGAAGCTATCCATTAAATACT
TAB131	TATGAATCTCTACCTGATCATAATGAAATTGTCTTTTATGGTAGTCCAATACCACATCGACGTTTTGCAAGAAGCTATCCATTAAATACT
TAB151	TATGAATCTCTACCTGATCATAATGAAATTGTCTTTTATGGTAGTCCAATACCACATCGACGTTTTGCAAGAAGCTATCCATTAAATACT
TAB152	TATGAATCTCTACCTCATCATAATGATATTGCGTTGCATGGTAGTCCAATACCACGTCGACGTTTTTCAAGAAGCTATCCATTAAATACT
SC52	TATGAATCTCTACCTCATCATAATGATATTGCGTTGCATGGTAGTCCAATACCACATCGACGTTTTGCAAGAAGCTATCCATTAAATACT
SC55	TATGAATCTCTACCTCATCATAATGATATTGCGTTGCATGGTAATCCAATACCACATCGACGTTTTTCAAGAAGCTATCCATTAAATACT
SC03	TATGAATCTCTACCTGATCATAATGAAATTGTCTTGCATGGTAGTCCAATACCACATCGACGTTTTTCAAGAAGCTATCCATTAAATACT
SC53	TATGAATCTCTACCTCATCATAATGATATTGCGTTGCATGGTAGTCCAATACCACGTCGACGTTTTTCAAGAAGCTATCCATTAAATACT
TAB141	TATGAATCTCTACCTGATCATAATGAAATTGCGTTGCATGGTAATCCAATACCACATCGACGTTTTTCAAGAAGCTATCCATTAAATACT
TAB123	TATGAATCTCTACCTGATCATAATGAAATTGTCTTTTATGGTAGTCCAATACCACATCGACGTTTTGCAAGAAGCTATCCATTAAATACT
AM1803	TATGAATCTCTACCTGATCATAATGAAATTGTCTTTTATGGTAGTCCAATACCACATCGACGTTTTGCAAGAAGCTATCCATTAAATACT
AM1814	TATGAATCTCTACCTCATCATAATGATATTGCGTTGCATGGTAATCCAATACCACATCGACGTTTTGCAAGAAGCTATCCATTAAATACT
AM1802	TATAAAGTTTCTTCAGAAAACAATGAAATTGCGGTTTTTGATAGCCAAATATCACATAATCCCTTTTCACCAAGGTATTCATCAAATACT
AM1811	TATGAATCTCTACCTGATCATAATGAAATTGTCTTTTATGGTAGTCCAATACCACATCGACGTTTTGCAAGAAGCTATCCATTAAATACT
SC50	TATAAAGTTTCTTCAGAAAACAATGAAATTGCGGTTTTTGATAGCCAAATATCACATAATCCCTTTTCACCAAGGTATTCATCAAATACT
SC56	TATGAATCTCTACCTGATCATAATGAAATTGTCTTTTATGGTAGTCCAATACCACATCGACGTTTTGCAAGAAGCTATCCATTAAATACT
TAB153	TATGAATCTCTACCTGATCATAATGAAATTGTCTTTTATGGTAGTCCAATACCACATCGACGTTTTGCAAGAAGCTATCCATTAAATACT
TAB154	TATGAATCTCTACCTGATCATAATGAAATTGTCTTTTATGGTAGTCCAATACCACATCGACGTTTTGCAAGAAGCTATCCATTAAATACT
TAB156	TATAAAGTTTCTTCAGAAAACAATGAAATTGCGGTTTTTGATAGCCAAATATCACATAATCCCTTTTCACCAAGGTATTCATCAAATACT
TAB136	TATGAATCTCTACCTCATCATAATGATATTGCGTTGCATGGTAATCCAATACAATATCAAGGTTTTTCACCAAGGTATTCATCAAATAGT
AM1880	TATGAATCTCTACCTCATCATAATGATATTGCGTTGCATGGTAATCCAATACCACATCGACGTTTTGCAAGAAGCTATCCATTAAATACT
PF3D7_	TATGAATCTCTACCTCATCATAATGATATTGCGTTGCATGGTAGTCCAATACCACGTCGACGTTTTTCAAGAAGCTATCCATTAAATACT
PfSD01	TATGAATCTCTACCTGATCATAATGAAATTGTCTTTTATGGTAGTCCAATACCACATCGACGTTTTTCAAGAAGCTATCCATTAAATACT
Pf7G8	TATACAGCTCTACCAGATTATAATGAAATTGCGTTGCACGGTAATCCAATACCACATCAAGGTTTTTCACCAAGGTATTCATCAAATAGT
FCR3 GENE	TATGAATCTCTACCTGATCATAATGAAATTGTCTTTTATGGTAGTCCAATACCACATCGACGTTTTGCAAGAAGCTATCCATTAAATACT
MS822 GENE	TATGAATCTCTACCTCATCATAATGATATTGCGTTGCATGGTAGTCCAATACCACGTCGACGTTTTTCAAGAAGCTATCCATTAAATACT
PfCD01_	TATACAGCTCTACCAGATTATAATGAAATTGCGTTGCACGGTAATCCAATACCACATCAAGGTTTTTCACCAAGGTATTCATCAAATAGT
PfKH01__	TATGAATCTCTACCTGATCATAATGAAATTGTCTTTTATGGTAGTCCAATACCACATCGACGTTTTGCAAGAAGCTATCCATTAAATACT
PfDd2__	TATGAATCTCTACCTCATCATAATGATATTGCGTTGCATGGTAGTCCAATACCACATCGACGTTTTGCAAGAAGCTATCCATTAAATACT
PfSN01_	TATGAATCTCTACCTCATCATAATGATATTGCGTTGCATGGTAGTCCAATACCACATCGACGTTTTGCAAGAAGCTATCCATTAAATACT
PfGN01	TATGAATCTCTACCTGATCATAATGAAATTGTCTTTTATGGTAGTCCAATACCACATCGACGTTTTGCAAGAAGCTATCCATTAAATACT
PfKE01	TATGATTCTCTACCTGATCATAATGAAATTGTCTTTTATGGTAGTCCAATACCACATCGACGTTTTGCAAGAAGCTATCCATTAAATACT
PfKH02	TATGAATCTCTACCTGATCATAATGAAATTGTCTTTTATGGTAGTCCAATACCACATCGACGTTTTGCAAGAAGCTATCCATTAAATACT
PfGB4	TATGAATCTCTACCTGATCATAATGAAATTGTCTTTTATGGTAGTCCAATACCACATCGACGTTTTGCAAGAAGCTATCCATTAAATACT
PfGA01_	TATGAATCTCTACCTGATCATAATGAAATTGTCTTTTATGGTAGTCCAATACCACATCGACGTTTTGCAAGAAGCTATCCATTAAATACT
PfHB3_	TATGAATCTCTACCTCATCATAATGATATTGCGTTGCATGGTAATCCAATACAATATCAAGGTTTTTCACCAAGGTATTCATCAAATAGT
PfIT_	TATGAATCTCTACCTGATCATAATGAAATTGTCTTTTATGGTAGTCCAATACCACATCGACGTTTTGCAAGAAGCTATCCATTAAATACT

```
                    1900      1910      1920      1930      1940      1950      1960      1970      1980
            ....|....|....|....|....|....|....|....|....|....|....|....|....|....|....|....|....|....|
PF3D7 CDS   ACAACTACATTGGAAGGTACATCTCAAAAACCATCTGCAGTTGAAACAATAATGTCCCTATTTAGAAATATTTTTGATTCTATAAAAAGT
MS822 mRNA  ACAACTACATTGGAAGGTACATCTCAAAAACCATCTGCAGTTGAAACAATAATGTCCCTATTTAGAAATATTTTTGATTCTATAAAAAGT
FCR3 mRNA   ACAACTACATTGGAAGGTACATCTCAAAAACCATCTGCAGTTGAAACAATAATGTCCCTATTTAGAAATATTTTTGATTCTATAAAAAGT
AM1898      ACAACTACATTGGAAGGTACATCTAAAAAACCATCTACTATTGAATCATTAACGTCTCTATTTGAAAATTTTTTTGATTCTATAAAAAGT
AA235       ACAACTACATTGGAAGGTACATCTCAAAAACCATCTGCAGTTGAAACAATAATGTCCCTATTTAGTAATTTTTTTGATTCTATAAAAAGT
AA243       ACAACTACATTGGAAGGTACATCTCAAAAACCATCTGCAGTTGAAACAATAATGTCCCTATTTAGAAATTTTTTTGATTCTATAAAAAGT
AA258       ACAACTACATTGGAAGGTACATCTCAAAAACCATCTGCAGTTGAAACAATAATGTCCCTATTTAGAAATTTTTTTGATTCTATAAAAAGT
AA264       ACAACTACATTGGAAGGTACATCTAAAAAACCATCTACTATTGAATCATTAACGTCTCTATTTGAAAATTTTTTTGATTCTATAAAAAGT
AM1737      ACAACTACATTGGAAGGTACATCTAAAAAACCATCTACTATTGAATCATTAACGTCTCTATTTGAAAATTTTTTTGATTCTATAAAAAGT
SC74        ACAACTACATTGGAAGGTACATCTCAAAAACCATCTGCAGTTGAAACAATAATGTCCCTATTTAGTAATTTTTTTGATTCTATAAAAAGT
TAB166      ACAACTACATTGGAAGGTACATCTCAAAAACCATCTGCAGTTGAAACAATAATGTCCCTATTTAGAAATTTTTTTGATTCTATAAAAAGT
TAB138      ACAACTACATTGGAAGGTACATCTCAAAAACCATCTGCAGTTGAAACAATAATGTCCCTATTTAGAAATATTTTTGATTCTATAAAAAGT
TAB130      ACAACTACATTGGAAGGTACATCTAAAAAACCATCTACTATTGAATCATTAACGTCTCTATTTGAAAATTTTTTTGATTCTATAAAAAGT
TAB131      ACAACTACATTGGAAGGTACATCTCAAAAACCATCTGCAGTTGAAACAATAATGTCCCTATTTAGTAATTTTTTTGATTCTATAAAAAGT
TAB151      ACAACTACATTGGAAGGTACATCTCAAAAACCATCTGCAGTTGAAACAATAATGTCCCTATTTAGTAATTTTTTTGATTCTATAAAAAGT
TAB152      ACAACTACATTGGAAGGTACATCTCAAAAACCATCTGCAGTTGAAACAATAATGTCCCTATTTAGAAATATTTTTGATTCTATAAAAAGT
SC52        ACAACTACATTGGAAGGTACATCTCAAAAACCATCTGCAGTTGAAACAATAATGTCCCTATTTAGTAATTTTTTTGATTCTATAAAAAGT
SC55        ACAACTACATTGGAAGGTACATCTCAAAAACCATCTGCAGTTGAAACAATAATGTCCCTATTTAGAAATTTTTTTGATTCTATAAAAAGT
SC03        ACAACTACATTGGAAGGTACATCTCAAAAACCATCTGCAGTTGAAACAATAATGTCCCTATTTAGAAATTTTTTTGATTCTATAAAAAGT
SC53        ACAACTACATTGGAAGGTACATCTCAAAAACCATCTGCAGTTGAAACAATAATGTCCCTATTTAGAAATATTTTTGATTCTATAAAAAGT
TAB141      ACAACTACATTGGAAGGTACATCTCAAAAACCATCTGCAGTTGAAACAATAATGTCCCTATTTAGTAATTTTTTTGATTCTATAAAAAGT
TAB123      ACAACTACATTGGAAGGTACATCTAAAAAACCATCTACTATTGAATCATTAACGTCTCTATTTGAAAATTTTTTTGATTCTATAAAAAGT
AM1803      ACAACTACATTGGAAGGTACATCTCAAAAACCATCTGCAGTTGAAACAATAATGTCCCTATTTAGAAATTTTTTTGATTCTATAAAAAGT
AM1814      ACAACTACATTGGAAGGTACATCTCAAAAACCATCTGCAGTTGAAACAATAATGTCCCTATTTAGAAATTTTTTTGATTCTATAAAAAGT
AM1802      GTAACTTCAATTGGAGGTACATCTAAAAAACCATCTACTATTGAATCATTAACGTCTCTATTTGAAAATTTTGTTGATGCCATAAAAAGT
AM1811      ACAACTACATTGGAAGGTACATCTCAAAAACCATCTGCAGTTGAAACAATAATGTCCCTATTTAGAAATTTTTTTGATTCTATAAAAAGT
SC50        GTAACTTCAATTGGAGGTACATCTAAAAAACCATCTACTATTGAATCATTAACGTCTCTATTTGAAAATTTTGTTGATGCCATAAAAAGT
SC56        ACAACTACATTGGAAGGTACATCTCAAAAACCATCTGCAGTTGAAACAATAATGTCCCTATTTAGAAATTTTTTTGATTCTATAAAAAGT
TAB153      ACAACTACATTGGAAGGTACATCTCAAAAACCATCTGCAGTTGAAACAATAATGTCCCTATTTAGAAATTTTTTTGATTCTATAAAAAGT
TAB154      ACAACTACATTGGAAGGTACATCTCAAAAACCATCTGCAGTTGAAACAATAATGTCCCTATTTAGTAATTTTTTTGATTCTATAAAAAGT
TAB156      GTAACTTCAATTGGAGGTACATCTAAAAAACCATCTACTATTGAATCATTAACGTCTCTATTTGAAAATTTTGTTGATGCCATAAAAAGT
TAB136      ATAACTTCAATAGAAGGTACATCTAACAATGTATCTACAATTGAATCATTAACGTCTATAATTGGAAATTTTGTTGATGCCATAAAAAGT
AM1880      ACAACTACATTGGAAGGTACATCTCAAAAACCATCTGCAGTTGAAACAATAATGTCCCTATTTAGAAATTTTGTTGATGCCATAAAAAGT
PF3D7_      ACAACTACATTGGAAGGTACATCTCAAAAACCATCTGCAGTTGAAACAATAATGTCCCTATTTAGAAATATTTTTGATTCTATAAAAAGT
PfSD01      ACAACTACATTGGAAGGTACATCTCAAAAACCATCTGCAGTTGAAACAATAATGTCCCTATTTAGAAATATTTTTGATTCTATAAAAAGT
Pf7G8       ATAACTTCAATAGAAGGTACATCTAACAATGTATCTACAATTGAATCATTAACGTCTATAATTGGAAATTTTGTTGATGCCATAAGAAAT
FCR3 GENE   ACAACTACATTGGAAGGTACATCTCAAAAACCATCTGCAGTTGAAACAATAATGTCCCTATTTAGAAATATTTTTGATTCTATAAAAAGT
MS822 GENE  ACAACTACATTGGAAGGTACATCTCAAAAACCATCTGCAGTTGAAACAATAATGTCCCTATTTAGAAATATTTTTGATTCTATAAAAAGT
PfCD01_     ATAACTTCAATAGAAGGTACATCTAACAATGTATCTACAATTGAATCATTAACGTCTATAATTGGAAATTTTGTTGATGCCATAAGAAAT
PfKH01__    ACAACTACATTGGAAGGTACATCTCAAAAACCATCTGCAGTTGAAACAATAATGTCCCTATTTAGTAATTTTTTTGATTCTATAAAAAGT
PfDd2__     ACAACTACATTGGAAGGTACATCTCAAAAACCATCTGCAGTTGAAACAATAATGTCCCTATTTAGTAATTTTTTTGATTCTATAAAAAGT
PfSN01_     ACAACTACATTGGAAGGTACATCTCAAAAACCATCTGCAGTTGAAACAATAATGTCCCTATTTAGTAATTTTTTTGATTCTATAAAAAGT
PfGN01      ACAACTACATTGGAAGGTACATCTCAAAAACCATCTGCAGTTGAAACAATAATGTCCCTATTTAGTAATTTTTTTGATTCTATAAAAAGT
PfKE01      ACAACTACATTGGAAGGTACATCTAAAAAACCATCTACTATTGAATCATTAACGTCTCTATTTGAAAATTTTTTTGATTCTATAAAAAGT
PfKH02      ACAACTACATTGGAAGGTACATCTAAAAAACCATCTACTATTGAATCATTAACGTCTCTATTTGAAAATTTTTTTGATTCTATAAAAAGT
PfGB4       ACAACTACATTGGAAGGTACATCTCAAAAACCATCTGCAGTTGAAACAATAATGTCCCTATTTAGTAATTTTTTTGATTCTATAAAAAGT
PfGA01_     ACAACTACATTGGAAGGTACATCTAAAAAACCATCTACTATTGAATCATTAACGTCTCTATTTGAAAATTTTTTTGATTCTATAAAAAGT
PfHB3_      ATAACTTCAATAGAAGGTACATCTAACAATGTATCTACAATTGAATCATTAACGTCTATAATTGGAAATTTTGTTGATGCCATAAAAAGT
PfIT_       ACAACTACATTGGAAGGTACATCTCAAAAACCATCTGCAGTTGAAACAATAATGTCCCTATTTAGAAATATTTTTGATTCTATAAAAAGT
```

```
                   1990      2000      2010      2020      2030      2040      2050      2060      2070
           ....|....|....|....|....|....|....|....|....|....|....|....|....|....|....|....|....|....|
PF3D7 CDS  TCTTCAATAACTAGAAGAAGCGTTAATTCAGCTCTTGAATTTAGTGAATCTACAACTAGGCCTGGAAATTCATTTTCTTTGCCTCAAATG
MS822 mRNA TCTTCAATAACTAGAAGAAGCGTTAATTCAGCTCTTGAATTTAGTGAATCTACAACTAGGCCTGGAAATTCATTTTCTTTGCCTCAAATG
FCR3 mRNA  TCTTCAATAACTAGAAGAAGCGTTAATCAAGATTTGGAACTTAGTGTATCTACAACTAGTCCTACAAATTCATATTCTTTGCCTCAAATA
AM1898     TCTTCAAGAACTAAAAGAAGCATTAATTCAGCTCTTGAATTTAGTGAATTTACAACTAGGCCTGAAAGTTCATATTCTTTGCCTCAAATA
AA235      TCTTCAAGAACTAGAAGAAGCGTTAATCAAGATTTGGAACTTAGTGAATCTACAACTAGTCCTACAAATTCATATTCTTTGCCTCAAATG
AA243      TCTTCAAGAACTAGAAGAAGCGTTAATTCAGCTTTTGAACTTAATGAATCTACAACTATGCCTGCTAATTCATATACTTTGAATCAAATA
AA258      TCTTCAAGAACTAGAAGAAGCGTTAATTCAGCTTTTGAACTTAATGAATCTACAACTATGCCTGCTAATTCATATACTTTGAATCAAATA
AA264      TCTTCAAGAACTAGAAGAAGCGTTAATCAAGATTTGGAACTTAGTGAATCTGCAGCTATGACTTCCAGTACTTCTCCTTTGCGCCAAATA
AM1737     TCTTCAAGAACTAGAAGAAGCGTTAATCACGATTTGGAACTTAGTGAATCTACAACTAGTCCTACAAATTCATATTCTTTGCCTCAAATG
SC74       TCTTCAAGAACTAGAAGAAGCGTTAATCACGATTTGGAACTTAGTGAATCTACAACTAGTCCTACAAATTCATATTCTTTGCCTCAAATA
TAB166     TCTTCAAGAACTAGAAGAAGCGTTAATTCAGCTTTTGAACTTAATGAATCTACAACTATGCCTGCTAATTCATATACTTTGAATCAAATA
TAB138     TCTTCAAGAACTAGAAGAAGCGTTAATCAAGATTTGGAACTTAGTGAATCTACAACTAGTCCTACAAATTCATATTCTTTGCCTCAAATA
TAB130     TCTTCAAGAACTAGAAGAAGCGTTAATTCAGCTCTTGAATTTAGTGAATCTACAACTAGGCCTGAAAGTTCATATTCTTTGCCTCAAATA
TAB131     TCTTCAAGAACTAGAAGAAGCGTTAAACACGATTTGGAACTTAGTGAATCTACAACTAGTCCTACAAATTCATATTCTTTGCCTCAAATA
TAB151     TCTTCAAGAACTAGAAGAAGCGTTAAACACGATTTGGAACTTAGTGAATCTACAACTAGTCCTACAAATTCATATTCTTTGCCTCAAATA
TAB152     TCTTCAATAACTAGAAGAAGCGTTAATTCAGCTCTTGAATTTAGTGAATCTACAACTAGGCCTGGAAATTCATTTTCTTTGCCTCAAATG
SC52       TCTTCAAGAACTAGAAGAAGCGTTAAACACGATTTGGAACTTAGTGAATCTACAACTAGTCCTACAAATTCATATTCTTTGCCTCAAATG
SC55       TCTTCAAGAACTAGAAGAAGCATTAATTCAGCTCTTGAATTTAGTGAATTTACAACTAGGCCTGAAAGTTCATATTCTTTGCCTCAAATA
SC03       TCTTCAAGAACTAGAAGAAGCGTTAATTCAGCTTTTGAACTTAATGAATCTACAACTATGCCTGCTAATTCATATACTTTGAATCAAATA
SC53       TCTTCAATAACTAGAAGAAGCGTTAATTCAGCTCTTGAATTTAGTGAATCTACAACTAGGCCTGGAAATTCATTTTCTTTGCCTCAAATG
TAB141     TCTTCAAGAACTAGAAGAAGCGTTAATCAAGATTTGGAACTTAGTGTATCTACAACTAGTCCTACAAATTCATATTCTTTGCCTCAAATA
TAB123     TCTTCAAGAACTAGAAGAAGCGTTAATCAAGATTTGGAACTTAGTGAATCTACAACTAGTCCTACAAATTCATATTCTTTGCCTCAAATA
AM1803     TCTTCAATAACTAGAAGAAGCGTTAATCAAGATTTGGAACTTAGTGTATCTACAACTAGTCCTACAAATTCATATTCTTTGCCTCAAATG
AM1814     TCTGTAATATCTAAAAGAAGCATTAATTCAGCTCTTGAATTTAGTGAATTTACAACTAGGCCTGAAAGTTCATATTCTTTGCCTCAAATA
AM1802     TCTGTAATAACTAAAAGAAGTGTTAATTCAGCTTTTGAACTTAATGAATCTACAACTATGCCTGCCAATTCATATACTTTGAATCAAATA
AM1811     TCTTCAATAACTAGAAGAAGCGTTAATCAAGATTTGGAACTTAGTGTATCTACAACTAGTCCTACAAATTCATATTCTTTGCCTCAAATA
SC50       TCTGTAATAACTAAAAGAAGTGTTAATTCAGCTTTTGAACTTAATGAATCTACAACTATGCCTGCCAATTCATATACTTTGAATCAAATA
SC56       TCTTCAATAACTAGAAGAAGCGTTAATCAAGATTTGGAACTTAGTGTATCTACAACTAGTCCTACAAATTCATATTCTTTGCCTCAAATA
TAB153     TCTTCAAGAACTAGAAGAAGCGTTAATCAAGATTTGGAACTTAGTGAATCTACAACTAGTCCTACAAATTCATATTCTTTGCCTCAAATA
TAB154     TCTTCAAGAACTAGAAGAAGCGTTAAACACGATTTCGAACTTAGTGAATCTGCAGCTATGACTTCCAGTACTTCTCCTTTGCGCCAAATA
TAB156     TCTGTAATAACTAAAAGAAGTGTTAATTCAGCTTTTGAACTTAATGAATCTACAACTATGCCTGCCAATTCATATACTTTGAATCAAATA
TAB136     TCTGTAATATCTAAAAGAAGCATTAATTCAGCTCTTGAATTTAGTGAATTTACAACTAGGCCTGAAAGTTCATATTCTTTGCCTCAAATA
AM1880     TCTGTAATATCTAAAAGAAGCATTAATTCAGCTCTTGAATTTAGTGAATTTACAACTAGGCCTGAAAGTTCATATTCTTTGCCTCAAATA
PF3D7_     TCTTCAATAACTAGAAGAAGCGTTAATTCAGCTCTTGAATTTAGTGAATCTACAACTAGGCCTGGAAATTCATTTTCTTTGCCTCAAATG
PfSD01     TCTTCAATAACTAGAAGAAGCGTTAAACACGATTTGGAACTTAGTGTATCTACAACTAGTCCTACAAATTCATATTCTTTGCCTCAAATA
Pf7G8      TCTGTAATATCTAAAAGAAGCACTAATTCAGCTCTTGAATTTAGTGAATCTACAACTAGGCCTGAAAGTTCATATTCTTTGCCTCAAATA
FCR3 GENE  TCTTCAATAACTAGAAGAAGCGTTAATCAAGATTTGGAACTTAGTGTATCTACAACTAGTCCTACAAATTCATATTCTTTGCCTCAAATA
MS822 GENE TCTTCAATAACTAGAAGAAGCGTTAATTCAGCTCTTGAATTTAGTGAATCTACAACTAGGCCTGGAAATTCATTTTCTTTGCCTCAAATG
PfCD01_    TCTGTAATATCTAAAAGAAGCACTAATTCAGCTCTTGAATTTAGTGAATCTACAACTAGGCCTGAAAGTTCATATTCTTTGCCTCAAATA
PfKH01__   TCTTCAAGAACTAGAAGAAGCGTTAAACACGATTTGGAACTTAGTGAATCTACAACTAGTCCTACAAATTCATATTCTTTGCCTCAAATA
PfDd2__    TCTTCAATAACTAGAAGAAGCGTTAAACACGATTTGGAACTTAGTGAATCTACAACTAGTCCTACAAATTCATATTCTTTGCCTCAAATG
PfSN01_    TCTTCAAGAACTAGAAGAAGCGTTAAACACGATTTGGAACTTAGTGAATCTACAACTAGTCCTACAAATTCATATTCTTTGCCTCAAATA
PfGN01     TCTTCAAGAACTAGAAGAAGCGTTAAACACGATTTGGAACTTAGTGAATCTACAACTAGTCCTACAAATTCATATTCTTTGCCTCAAATA
PfKE01     TCTTCAAGAACTAGAAGAAGCGTTAATCAAGATTTGGAACTTAGTGAATCTGCAGCTATGACTTCCAGTACTTCTCCTTTGCGCCAAATA
PfKH02     TCTTCAAGAACTAGAAGAAGCGTTAATCAAGATTTGGAACTTAGTGAATCTGCAGCTATGACTTCCAGTACTTCTCCTTTGCGCCAAATA
PfGB4      TCTTCAAGAACTAGAAGAAGCGTTAAACACGATTTGGAACTTAGTGAATCTACAACTAGTCCTACAAATTCATATTCTTTGCCTCAAATG
PfGA01_    TCTTCAAGAACTAGAAGAAGCGTTAATCAAGATTTGGAACTTAGTGAATCTGCAGCTATGACTTCCAGTACTTCTCCTTTGCGCCAAATA
PfHB3_     TCTGTAATATCTAAAAGAAGCATTAATTCAGCTCTTGAATTTAGTGAATCTACAACTAGGCCTGAAAGTTCATATTCTTTGCCTCAAATA
PfIT_      TCTTCAATAACTAGAAGAAGCGTTAATCAAGATTTGGAACTTAGTGTATCTACAACTAGTCCTACAAATTCATATTCTTTGCCTCAAATA
```

```
                 2080      2090      2100      2110      2120      2130      2140      2150      2160
             ....|....|....|....|....|....|....|....|....|....|....|....|....|....|....|....|....|....|
PF3D7 CDS    ACTTGGGAATTTGAAGCTCCTGGTCAAGGAACGGTGATGAGAATGTTAGGACAGAAAAATTCTTTCAAAACACTTGCACAAACAAAACAG
MS822 mRNA   ACTTGGGAATTTGAAGCTCCTGGTCAAGGAACGGTGATGAGAATGTTAGGACAGAAAAATTCTTTCAAAACACTTGCACAAACAAAACAG
FCR3 mRNA    ACTTTGGAATTTGAAGCTCCTGCTCAAGGAACGGTGATGGGGATGTTAGGACAGGTAAATCCTTTCTCATCACTTGCACAAACAAGTCAA
AM1898       ATTTTAGATTTTGAAGCACCTGGTCAAGAAACAGTGATGAGAATGTTAGGAAATGGAAATTCTTACACATCACTTGCACAAAAAAGTCAA
AA235        ACTTGGGAATTTGAAGCTCCTGGTCAAGGAACGGTGATGGGAATGTTAGGACAGAAAAATTCTTTCAAAACACTTGCACAAACAAAACAG
AA243        ACTTTGGAATTTGAAGCTCCTGCTCAAGGAACGGTGGTGGGGAGGTTAGGACAGGTAAATTCTTTCTCATCACTTGCACAAACACGTCAA
AA258        ACTTTGGAATTTGAAGCTCCTGCTCAAGGAACGGTGGTGGGGAGGTTAGGACAGGTAAATTCTTTCTCATCACTTGCACAAACACGTCAA
AA264        ACTTTGGATATTAAATCACATGGAGAAAACATGGTGATGGGAATGTTAGGACATGGAAATCATTTCGAATCACTTGAACAAAAACGAGGA
AM1737       ACTTGGGAATTTGAAGCTCCTGGTCAAGGAACGGTGGTGGGGAGGTTAGGACAGGTAAATTCTTTCTCATCACTTGCACAAACAAGTCAA
SC74         ACTTTGGAATTTGAAGCTCCTGCTCAAGGAATGGTGATGGGAATGTTAGGACAGAAAAATTCTTTCAAAACACTTGCACAAACAAAACAG
TAB166       ACTTTGGAATTTGAAGCTCCTGCTCAAGGAACGGTGGTGGGGAGGTTAGGACAGGTAAATTCTTTCTCATCACTTGCACAAACACGTCAA
TAB138       ACTTTGGAATTTGAAGCTCCTGCTCAAGGAACGGTGGTGGGGAGGTTAGGACAGGTAAATTCTTTCTCATCACTTGCACAAACAAGTCAA
TAB130       ATTTTATATTTTGAAGCACCTGGTCAAGGAACGGTGGTGGGGAGGTTAGGACAGGTAAATTCTTTCTCATCACTTGCACAAACAAGTCAA
TAB131       ACTTTGGAATTTGAAGCTCCTGCTCAAGGAACGGTGATGGGGATGTTAGGACAGGTAAATTCTTTCTCATCACTTGCACAAACAAGTCAA
TAB151       ACTTTGGAATTTGAAGCTCCTGCTCAAGGAACGGTGATGGGGATGTTAGGACAGGTAAATTCTTTCTCATCACTTGCACAAACAAGTCAA
TAB152       ACTTGGGAATTTGAAGCTCCTGGTCAAGGAACGGTGATGAGAATGTTAGGACAGAAAAATTCTTTCAAAACACTTGCACAAACAAAACAG
SC52         ACTTGGGAATTTGAAGCTCCTGGTCAAGGAACGGTGATGAGAATGTTAGGACAGAAAAATTCTTTCAAAACACTTGCACAAACAAAACAG
SC55         ACTTTGGAATTTGAAGCACCTGGTCAAGAAACGGTGATGAGAATGTTAGGACAGGTAAATTCTTTCTCATCACTTGCACAAACAAGTCAA
SC03         ACTTTGGAATTTGAAGCTCCTGCTCAAGGAACGGTGGTGGGGAGGTTAGGACAGGTAAATTCTTTCTCATCACTTGCACAAACACGTCAA
SC53         ACTTGGGAATTTGAAGCTCCTGGTCAAGGAACGGTGATGAGAATGTTAGGACAGAAAAATTCTTTCAAAACACTTGCACAAACAAAACAG
TAB141       ACTTTGGAATTTGAAGCTCCTGCTCAAGGAACGGTGATGGGAATGTTAGGACAGAAAAATTCTTTCAAAACACTTGCACAAACAAAACAG
TAB123       ACTTTATATTTTGAAGCACCTGGTCAAGGAACGGTGATGGGGATGTTAGGACAGGTAAATTCTTACACATCACTTGGACAAACAAAACAG
AM1803       ACTTGGGAATTTGAAGCTCCTGGTCAAGGAACGGTGATGGGGATGTTAGGACAGGTAAATTCTTTCTCATCACTTGCACAAACAAGTCAA
AM1814       ATTTTAGATTTTGAAGCACCTGGTCAAGGAACGGTGATGGGGATGTTAGGACAGGTAAATTCTTACACATCACTTGGACAAACAAAACAG
AM1802       ACTTTGGATTCTGAGCCACCTCTTCAAGGAACGGTGATGGGGATGTTAGGACAGGTAAATTCTTACACATCACTTGGACAAACAAAACAG
AM1811       ACTTTGGAATTTGAAGCTCCTGCTCAAGGAACGGTGATGGGGATGTTAGGACAGGTAAATTCTTTCTCATCACTTGCACAAACAAGTCAA
SC50         ACTTTGGATTCTGAGCCACCTCTTCAAGGAACGGTGATGGGGATGTTAGGACAGGTAAATTCTTACACATCACTTGGACAAACAAAACAG
SC56         ACTTTGGAATTTGAAGCTCCTGCTCAAGGAACGGTGATGGGGATGTTAGGACAGGTAAATTCTTTCTCATCACTTGCACAAACAAGTCAA
TAB153       ACTTTGGAATTTGAAGCTCCTGCTCAAGGAACGGTGGTGGGGAGGTTAGGACAGGTAAATTCTTTCTCATCACTTGCACAAACAAGTCAA
TAB154       ACTTTGGATATTAAATCACATGGAGAAAACATGGTGATGGGAATGTTAGGACATGGAAATCATTTCGAATCACTTGAACAAAAACGAGGA
TAB156       ACTTTGGATTCTGAGCCACCTCTTCAAGGAACGGTGATGGGGATGTTAGGACAGGTAAATTCTTACACATCACTTGGACAAACAAAACAG
TAB136       ATTTTAGATTTTGAAGCACCTGGTCAAGAAACAGTGATGAGAATGTTAGGAAATGGAAATTCTTACACATCACTTGCACAAAAAAGTCAA
AM1880       ATTTTAGATTTTGAAGCACCTGGTCAAGAAACAGTGATGAGAATGTTAGGAAATGGAAATTCTTACACATCACTTGCACAAAAAAGTCAA
PF3D7_       ACTTGGGAATTTGAAGCTCCTGGTCAAGGAACGGTGATGAGAATGTTAGGACAGAAAAATTCTTTCAAAACACTTGCACAAACAAAACAG
PfSD01       ACTTTGGAATTTGAAGCTCCTGCTCAAGGAACGGTGATGGGGATGTTAGGACAGGTAAATTCTTTCTCATCACTTGCACAAACAAGTCAA
Pf7G8        ATTTTATATTTTCAAGCACCTGGTCAAGAAACAGTGATGGGAATGTTAGGAAATGGAAATTCTTACACATCACTTGCACAAAAAAGTCAA
FCR3 GENE    ACTTTGGAATTTGAAGCTCCTGCTCAAGGAACGGTGATGGGGATGTTAGGACAGGTAAATCCTTTCTCATCACTTGCACAAACAAGTCAA
MS822 GENE   ACTTGGGAATTTGAAGCTCCTGGTCAAGGAACGGTGATGAGAATGTTAGGACAGAAAAATTCTTTCAAAACACTTGCACAAACAAAACAG
PfCD01_      ATTTTATATTTTCAAGCACCTGGTCAAGAAACAGTGATGGGAATGTTAGGAAATGGAAATTCTTACACATCACTTGCACAAAAAAGTCAA
PfKH01__     ACTTTGGAATTTGAAGCTCCTGCTCAAGGAACGGTGATGGGGATGTTAGGACAGGTAAATTCTTTCTCATCACTTGCACAAACAAGTCAA
PfDd2__      ACTTGGGAATTTGAAGCTCCTGGTCAAGGAACGGTGATGAGAATGTTAGGACAGAAAAATTCTTTCAAAACACTTGCACAAACAAAACAG
PfSN01_      ACTTTGGAATTTGAAGCTCCTGCTCAAGGAACGGTGGTGGGGAGGTTAGGACAGGTAAATTCTTTCTCATCACTTGCACAAACAAGTCAA
PfGN01       ATTTTAGATTTTGAAGCACCTGGTCAAGAAACAGTGATGAGAATGTTAGGAAATGGAAATTCTTACACATCACTTGGACAAACAAAACAG
PfKE01       ACTTTGGATATTAAATCACATGGAGAAAACATGGTGATGGGAATGTTAGGACATGGAAATCATTTCGAATCACTTGAACAAAAACGAGGA
PfKH02       ACTTTGGATATTAAATCACATGGAGAAAACATGGTGATGGGAATGTTAGGACATGGAAATCATTTCGAATCACTTGAACAAAAACGAGGA
PfGB4        ACTTGGGAAATTGAAGATCCTGGTCAAGGAACGGTGATGAGAATGTTAGGACAGAAAAATTCTTTCAAAACACTTGCACAAACAAAACAG
PfGA01_      ACTTTGGATTCTGAGCCACCTCTTCAAGGAACGGTGATGGGGATGTTAGGACAGGTAAATTCTTACACATCACTTGGACAAACAAAACAG
PfHB3_       ATTTTAGATTTTGAAGCACCTGGTCAAGAAACAGTGATGAGAATGTTAGGAAATGGAAATTCTTACACATCACTTGCACAAAAAAGTCAA
PfIT_        ACTTTGGAATTTGAAGCTCCTGCTCAAGGAACGGTGATGGGGATGTTAGGACAGGTAAATCCTTTCTCATCACTTGCACAAACAAGTCAA
```

```
               2170      2180      2190      2200      2210      2220      2230      2240      2250
           ....|....|....|....|....|....|....|....|....|....|....|....|....|....|....|....|....|
PF3D7 CDS   GAAGTTTCTTCATCTAGTTCTGAATCACGTTCAAGTTCCTTCAACAATAACTCCCAAATAATTCCCCATATATTGAAAACGCAAGTTGAT
MS822 mRNA  GAAGTTTCTTCATCTAGTTCTGAATCACGTTCAAGTTCCTTCAACAATAACTCCCAAATAATTCCCCATATATTGAAAACGCAAGTTGAT
FCR3 mRNA   GAAGATTCTACATCTAGTTCTGAATCACGTTCAAGTTCCTTCAGCCATAACTCCCAAATAATGCCCACTATATTAAAAAGACCAATTAAC
AM1898      GAAGTTTCTTCATCTAGTCCTGTATCACGTTCAAGTTCCTTCAACCATAACTCCCAAACAATTAACCGTATATTAAAAGCACAAGTTGAT
AA235       GAAGTTTCTTCATCTAGTTCTGAATCACGTTCAAGTTCCTTCAACAATAACTCCAAAATAATTCCCCATATATTGAAAACGCAAGTTGAT
AA243       GAAGATTCTACATCTAGTTCTGAATCACGTTCAAGTTCCTTCAACAATAACTCCCAAATAATTCCCCATATATTGAAAACGCAAGTTGAT
AA258       GAAGATTCTACATCTAGTTCTGAATCACGTTCAAGTTCCTTCAACAATAACTCCCAAATAATTCCCCATATATTGAAAACGCAAGTTGAT
AA264       GAAGATTCTTCATCAAATCATGGATCACCTTCACATTCCTTCAGCCTAAGCTCCAAAACAATTCACCCTATATTGAAAACGCAAGTTGAT
AM1737      GAAGATTCTACATCTAGTTCTGAATCACGTTCAAGTTCCTTCAACAATAACTCCCAAATAATTCCCCATATATTGAAAACGCAAGTTGAT
SC74        GAAGTTTCTTCATCTAGTTCTGAATCACGTTCAAGTTCCTTCAACAATAACTCCAAAATAATTCCCCATATATTGAAAACGCAAGTTGAT
TAB166      GAAGATTCTACATCTAGTTCTGAATCACGTTCAAGTTCCTTCAACAATAACTCCCAAATAATTCCCCATATATTGAAAACGCAAGTTGAT
TAB138      GAAGATTCTACATCTAGTTCTGAATCACGTTCAAGTTCCTTCAACAATAACTCCCAAATAATTCCCCATATATTGAAAAGACCAGTTAAC
TAB130      GAAGATTCTACATCTAGTTCTGAATCACGTTCAAGTTCCTTCAACAATAACTCCCAAATAATTCCCCATATATTGAAAACGCAAGTTGAT
TAB131      GAAGATTCTACATCTAGTTCTGAATCACGTTCAAGTTCCTTCAACAATAACTCCCAAATAATTCCCCATATATTGAAAACGCAAGTTGAT
TAB151      GAAGATTCTACATCTAGTTCTGAATCACGTTCAAGTTCCTTCAACAATAACTCCCAAATAATTCCCCATATATTGAAAACGCAAGTTGAT
TAB152      GAAGTTTCTTCATCTAGTTCTGAATCACGTTCAAGTTCCTTCAACAATAACTCCCAAATAATTCCCCATATATTGAAAACGCAAGTTGAT
SC52        GAAGTTTCTTCATCTAGTTCTGAATCACGTTCAAGTTCCTTCAGCCATAACTCCCAAATAATGCCCACTATATTAAAAACGCAAGTTGAT
SC55        GAAGTTTCTTCATCTAGTTCTGAATCACGTTCAAGTTCCTTCAACCATAACTCCCAAATAATTCCCCATATATTGAAAACGCAAGTTGAT
SC03        GAAGATTCTACATCTAGTTCTGAATCACGTTCAAGTTCCTTCAACAATAACTCCCAAATAATTCCCCATATATTGAAAAGACCAGTTAAC
SC53        GAAGTTTCTTCATCTAGTTCTGAATCACGTTCAAGTTCCTTCAACAATAACTCCCAAATAATTCCCCATATATTGAAAACGCAAGTTGAT
TAB141      GAAGTTTCTTCATCTAGTTCTGAATCACGTTCAAGTTCCTTCAACAATAACTCCAAAATAATTCCCCATATATTGAAAACGCAAGTTGAT
TAB123      GAAGTTGCTTCATCTAGTCCTGGATCACGTTCAAGTTCCTTCAACCATAACTCCCAAACAATTCACCCTATATTGAAAACGCAAGTTGAT
AM1803      GAAGATTCTACATCTAGTTCTGAATCACGTTCAAGTTCCTTCAACAATAACTCCCAAATAATTCCCCATATATTGAAAAGACCAGTTAAC
AM1814      GAAGTTTCTTCATCTAGTCCTGTATCACGTTCAAGTTCCTTCAACCATAACTCCCAAACAATTAACCGTATATTAAAAGCACAAGTTGAT
AM1802      GAAGTTGCTTCATCTAGTCCTGGATCACCTTCACATTCCTTCACCCTAAGATCCAAAACAATGCACCCTATATTAAAAAGACAATTTGAA
AM1811      GAAGATTCTACATCTAGTTCTGAATCACGTTCAAGTTCCTTCAACAATAACTCCCAAATAATTCCCCATATATTGAAAAGACCAGTTAAC
SC50        GAAGTTGCTTCATCTAGTCCTGGATCACCTTCACATTCCTTCACCCTAAGATCCAAAACAATGCACCCTATATTAAAAAGACAATTTGAA
SC56        GAAGATTCTACATCTAGTTCTGAATCACGTTCAAGTTCCTTCAACAATAACTCCCAAATAATTCCCCATATATTGAAAAGACCAGTTAAC
TAB153      GAAGATTCTACATCTAGTTCTGAATCACGTTCAAGTTCCTTCAACAATAACTCCCAAATAATTCCCCATATATTGAAAAGACCAGTTAAC
TAB154      GAAGATTCTTCATCAAATCATGGATCACCTTCACATTCCTTCAGCCTAAGCTCCAAAACAATTCACCCTATATTGAAAACGCAAGTTGAT
TAB156      GAAGTTGCTTCATCTAGTCCTGGATCACCTTCACATTCCTTCACCCTAAGATCCAAAACAATGCACCCTATATTAAAAAGACAATTTGAA
TAB136      GAAGTTTCTTCATCTAGTCCTGTATCACGTTCAAGTTCCTTCAACCATAACTCCCAAACAATTAACCGTATATTAAAAGCACAAGTTGAT
AM1880      GAAGTTTCTTCATCTAGTCCTGTATCACGTTCAAGTTCCTTCAACCATAACTCCCAAACAATTAACCGTATATTAAAAGCACAAGTTGAT
PF3D7_      GAAGTTTCTTCATCTAGTTCTGAATCACGTTCAAGTTCCTTCAACAATAACTCCCAAATAATTCCCCATATATTGAAAACGCAAGTTGAT
PfSD01      GAAGATTCTACATCTAGTTCTGAATCACGTTCAAGTTCCTTCAACAATAACTCCCAAATAATTCCCCATATATTGAAAAGACCAGTTAAC
Pf7G8       GAAGTTTCTTCATCTAGTCCTGTATCACGTTCAAGTTCCTTCAACCATAACTCCCAAACAATTAACCGTATATTAAAAGCACAAGTTGAT
FCR3 GENE   GAAGATTCTACATCTAGTTCTGAATCACGTTCAAGTTCCTTCAGCCATAACTCCCAAATAATGCCCACTATATTAAAAAGACCAATTAAC
MS822 GENE  GAAGTTTCTTCATCTAGTTCTGAATCACGTTCAAGTTCCTTCAACAATAACTCCCAAATAATTCCCCATATATTGAAAACGCAAGTTGAT
PfCD01_     GAAGTTTCTTCATCTAGTCCTGTATCACGTTCAAGTTCCTTCAACCATAACTCCCAAACAATTAACCGTATATTAAAAGCACAAGTTGAT
PfKH01__    GAAGATTCTACATCTAGTTCTGAATCACGTTCAAGTTCCTTCAACAATAACTCCCAAATAATTCCCCATATATTGAAAACGCAAGTTGAT
PfDd2__     GAAGTTTCTTCATCTAGTTCTGAATCACGTTCAAGTTCCTTCAGCCATAACTCCCAAATAATGCCCACTATATTAAAAAGACCAATTAAC
PfSN01_     GAAGATTCTACATCTAGTTCTGAATCACGTTCAAGTTCCTTCAACAATAACTCCCAAATAATTCCCCATATATTGAAAACGCAAGTTGAT
PfGN01      GAAGTTTCTTCATCTAGTTCTGAATCACGTTCAAGTTCCTTCAACAATAACTCCCAAATAATTCCCCATATATTGAAAACGCAAGTTGAT
PfKE01      GAAGATTCTTCATCAAATCATGGATCACCTTCACATTCCTTCAGCCTAAGCTCCAAAACAATTCACCCTATATTGAAAACGCAAGTTGAT
PfKH02      GAAGATTCTTCATCAAATCATGGATCACCTTCACATTCCTTCAGCCTAAGCTCCAAAACAATTCACCCTATATTGAAAACGCAAGTTGAT
PfGB4       GAAGTTTCTTCATCTAGTTCTGAATCACGTTCAAGTTCCTTCAGCCATAACTCCCAAATAATGCCCACTATATTAAAAAGACCAATTAAC
PfGA01_     GAAGTTGCTTCATCTAGTCCTGGATCACCTTCACATTCCTTCAGCCTAAGCTCCAAAACAATTCCCCATATATTGAAAACGCAAGTTGAT
PfHB3_      GAAGTTTCTTCATCTAGTCCTGTATCACGTTCAAGTTCCTTCAACCATAACTCCCAAACAATTAACCGTATATTAAAAGCACAAGTTGAT
PfIT_       GAAGATTCTACATCTAGTTCTGAATCACGTTCAAGTTCCTTCAGCCATAACTCCCAAATAATGCCCACTATATTAAAAAGACCAATTAAC
```

```
                        2260      2270      2280      2290      2300      2310      2320      2330      2340
                    ....|....|....|....|....|....|....|....|....|....|....|....|....|....|....|....|....|....|
PF3D7 CDS     GGAACATATGTTGTGTCAGGA---CATAAAGCAAATGTTCCCAAAGGCATTGAACATTATATAAAGTATGTCCCCGTAGCTTTAGCAGTT
MS822 mRNA    GGAACATATGTTGTGTCAGGA---CATAAAGCAAATGTTCCCAAAGGCATTGAACATTATATAAAGTATGTCCCCGTAGCTTTAGCAGTT
FCR3 mRNA     GGAAATTATGTTGTGTCAGGAGGACATAAAACAAATGTTCCCAAAGGTATTGAACATTATATAAAGTCTGTCCCCGTAGCTTTAGCAGTT
AM1898        GGAACATATGTTGTGTCAGGA---CATAAAGTAAATGTTCCCAAAGGCATTGAACATTATATAAAGTATGTCCCCGTAGCTTTAGCAGTT
AA235         GGAACATATGTTGTGTCAGGA---CATAAAGCAAATGTTCCCAAAGGCATTGAACATTATATAAAGTATGTCCCCGTAGCTTTAGCAGTT
AA243         GGAACATATGTTGTGTCAGGA---CATAAAGCAAATGTTCCCAAAGGCATTGAACATTATATAAAGTATGTCCCCGTAGCTTTAGCAGTT
AA258         GGAACATATGTTGTGTCAGGA---CATAAAGCAAATGTTCCCAAAGGCATTGAACATTATATAAAGTATGTCCCCGTAGCTTTAGCAGTT
AA264         GGAACATATGTTGTGTCAGGA---CATAAAGCAAATGTTCCCAAAGGCATTGAACATTATATAAAGTATGTCCCCGTAGCTTTAGCAGTT
AM1737        GGAACATATGTTGTGTCAGGA---CATAAAGCAAATGTTCCCAAAGGCATTGAACATTATATAAAGTATGTCCCCGTAGCTTTAGCAGTT
SC74          GGAACATATGTTGTGTCAGGA---CATAAAGCAAATGTTCCCAAAGGCATTGAACATTATATAAAGTATGTCCCCGTAGCTTTAGCAGTT
TAB166        GGAACATATGTTGTGTCAGGA---CATAAAGCAAATGTTCCCAAAGGCATTGAACATTATATAAAGTATGTCCCCGTAGCTTTAGCAGTT
TAB138        GGAACTTATGTTGTGTCAGGAGGACATAAAGCAAATGTTCCCAAAGGCATTGAACATTATATAAAGTATGTCCCCGTAGCTTTAGCAGTT
TAB130        GGAACATATGTTGTGTCAGGA---CATAAAGCAAATGTTCCCAAAGGCATTGAACATTATATAAAGTATGTCCCCGTAGCTTTAGCAGTT
TAB131        GGAACATATGTTGTGTCAGGA---CATAAAGCAAATGTTCCCAAAGGCATTGAACATTATATAAAGTATGTCCCCGTAGCTTTAGCAGTT
TAB151        GGAACATATGTTGTGTCAGGA---CATAAAGCAAATGTTCCCAAAGGCATTGAACATTATATAAAGTATGTCCCCGTAGCTTTAGCAGTT
TAB152        GGAACATATGTTGTGTCAGGA---CATAAAGCAAATGTTCCCAAAGGCATTGAACATTATATAAAGTATGTCCCCGTAGCTTTAGCAGTT
SC52          GGAACATATGTTGTGTCAGGAGGACATAAAACAAATGTTCCCAAAGGTATTGAACATTATATAAAGTATGTCCCCGTAGCTTTAGCAGTT
SC55          GGAACATATGTTGTGTCAGGA---CATAAAGTAAATGTTCCCAAAGGCATTGAACATTATATAAAGTCTGTCCCCGTAGCTTTAGCAGTT
SC03          GGAACTTATGTTGTGTCAGGAGGACATAAAACAAATGTTCCCAAAGGTATTGAACATTATATAAAGTATGTCCCCGTAGCTTTAGCAGTT
SC53          GGAACATATGTTGTGTCAGGA---CATAAAGCAAATGTTCCCAAAGGCATTGAACATTATATAAAGTATGTCCCCGTAGCTTTAGCAGTT
TAB141        GGAACATATGTTGTGTCAGGA---CATAAAGCAAATGTTCCCAAAGGCATTGAACATTATATAAAGTATGTCCCCGTAGCTTTAGCAGTT
TAB123        GGAACATATGTTGTGTCAGGA---CATAAAGCAAATGTTCCCAAAGGCATTGAACATTATATAAAGTATGTCCCCGTAGCTTTAGCAGTT
AM1803        GGAACTTATGTTGTGTCAGGA---CATAAAGCAAATGTTCCCAAAGGTATTGAACATTATATAAAGTCTGTCCCCGTAGCTTTAGCAGTT
AM1814        GGAACATATGTTGTGTCAGGA---CATAAAGCAAATGTTCCCAAAGGCATTGAACATTATATAAAGTCTGTCCCCGTAGCTTTAGCAGTT
AM1802        GGAACATATGTTGTGTCAGGA---GATAAAGCAAATATTCCCAAAGGTATTGAACATTATATAAAGTCCGTCCCAGTAGCTTTAGCAGTT
AM1811        GGAACTTATGTTGTGTCAGGAGGACATAAAGCAAATGTTCCCAAAGGTATTGAACATTATATAAAGTCTGTCCCCGTAGCTTTAGCAGTT
SC50          GGAACATATGTTGTGTCAGGA---GATAAAGCAAATATTCCCAAAGGTATTGAACATTATATAAAGTCCGTCCCAGTAGCTTTAGCAGTT
SC56          GGAACTTATGTTGTGTCAGGAGGACATAAAGCAAATGTTCCCAAAGGTATTGAACATTATATAAAGTCTGTCCCCGTAGCTTTAGCAGTT
TAB153        GGAACTTATGTTGTGTCAGGAGGACATAAAGCAAATGTTCCCAAAGGTATTGAACATTATATAAAGTCTGTCCCCGTAGCTTTAGCAGTT
TAB154        GGAACATATGTTGTGTCAGGA---CATAAAGCAAATGTTCCCAAAGGCATTGAACATTATATAAAGTATGTCCCCGTAGCTTTAGCAGTT
TAB156        GGAACATATGTTGTGTCAGGA---GATAAAGCAAATATTCCCAAAGGTATTGAACATTATATAAAGTCCGTCCCAGTAGCTTTAGCAGTT
TAB136        GGAACATATGTTGTGTCAGGA---CATAAAGTAAATGTTCCCAAAGGCATTGAACATTATATAAAGTCTGTCCCCGTAGCTTTAGCAGTT
AM1880        GGAACATATGTTGTGTCAGGA---CATAAAGTAAATGTTCCCAAAGGCATTGAACATTATATAAAGTCTGTCCCCGTAGCTTTAGCAGTT
PF3D7_        GGAACATATGTTGTGTCAGGA---CATAAAGCAAATGTTCCCAAAGGCATTGAACATTATATAAAGTATGTCCCCGTAGCTTTAGCAGTT
PfSD01        GGAACTTATGTTGTGTCAGGAGGACATAAAGCAAATGTTCCCAAAGGTATTGAACATTATATAAAGTCTGTCCCCGTAGCTTTAGCAGTT
Pf7G8         GGAACATATGTTGTGTCAGGA---CATAAAGTAAATGTTCCCAAAGGCATTGAACATTATATAAAGTCTGTCCCCGTAGCTTTAGCAGTT
FCR3 GENE     GGAAATTATGTTGTGTCAGGAGGACATAAAACAAATGTTCCCAAAGGTATTGAACATTATATAAAGTCTGTCCCCGTAGCTTTAGCAGTT
MS822 GENE    GGAACATATGTTGTGTCAGGA---CATAAAGCAAATGTTCCCAAAGGCATTGAACATTATATAAAGTATGTCCCCGTAGCTTTAGCAGTT
PfCD01_       GGAACATATGTTGTGTCAGGA---CATAAAGTAAATGTTCCCAAAGGCATTGAACATTATATAAAGTCTGTCCCCGTAGCTTTAGCAGTT
PfKH01__      GGAACATATGTTGTGTCAGGA---CATAAAGCAAATGTTCCCAAAGGCATTGAACATTATATAAAGTATGTCCCCGTAGCTTTAGCAGTT
PfDd2__       GGAAATTATGTTGTGTCAGGAGGACATAAAACAAATGTTCCCAAAGGTATTGAACATTATATAAAGTATGTCCCCGTAGCTTTAGCAGTT
PfSN01_       GGAACATATGTTGTGTCAGGA---CATAAAGCAAATGTTCCCAAAGGCATTGAACATTATATAAAGTATGTCCCCGTAGCTTTAGCAGTT
PfGN01        GGAACATATGTTGTGTCAGGA---CATAAAGCAAATGTTCCCAAAGGCATTGAACATTATATAAAGTATGTCCCCGTAGCTTTAGCAGTT
PfKE01        GGAACATATGTTGTGTCAGGA---CATAAAGCAAATGTTCCCAAAGGCATTGAACATTATATAAAGTATGTCCCCGTAGCTTTAGCAGTT
PfKH02        GGAACATATGTTGTGTCAGGA---CATAAAGCAAATGTTCCCAAAGGCATTGAACATTATATAAAGTATGTCCCCGTAGCTTTAGCAGTT
PfGB4         GGAAATTATGTTGTGTCAGGAGGACATAAAACAAATGTTCCCAAAGGTATTGAACATTATATAAAGTATGTCCCCGTAGCTTTAGCAGTT
PfGA01_       GGAACATATGTTGTGTCAGGA---CATAAAGCAAATGTTCCCAAAGGCATTGAACATTATATAAAGTATGTCCCCGTAGCTTTAGCAGTT
PfHB3_        GGAACATATGTTGTGTCAGGA---CATAAAGTAAATGTTCCCAAAGGCATTGAACATTATATAAAGTCTGTCCCCGTAGCTTTAGCAGTT
PfIT_         GGAAATTATGTTGTGTCAGGAGGACATAAAACAAATGTTCCCAAAGGTATTGAACATTATATAAAGTCTGTCCCCGTAGCTTTAGCAGTT
```

```
                  2350      2360      2370      2380      2390      2400      2410      2420      2430
              ....|....|....|....|....|....|....|....|....|....|....|....|....|....|....|....|....|....|
PF3D7 CDS     TTTGGAGTTCTTTTCGTTTTTATATTATTTAACAAA------------------------------------------------------
MS822 mRNA    TTTGGAGTTCTTTTCGTTTTTATATTATTTAACAAA------------------------------------------------------
FCR3 mRNA     TTTGGAGTTCTTTTCGTTTTTATATTATTTAACAAA------------------------------------------------------
AM1898        TTTGGAGTTCTTTTCGTTTTTATATTATTTAACAAAGTAATAATAAAAAAA-----TATATACATATATATATATATTTTATTTTATTTC
AA235         TTTGGAGTTCTTTTCGTTTTTATATTATTTAACAAAGTAATAATAAAAAAA-----TATATACATATATATATATATTTCATTTCATTTC
AA243         TTTGGAGTTCTTTTCGTTTTTATATTATTTAACAAAGTAATAATAAAAAAA-----TATATACATATATATATATATTTCATTTCATTTC
AA258         TTTGGAGTTCTTTTCGTTTTTATATTATTTAACAAAGTAATAATAAAAAAA-----TATATACATATATATATATATTTCATTTCATTTC
AA264         TTTGGAGTTCTTTTCGTTTTTATATTATTTAACAAAGTAATAATAAAAAAA-----TATATACATATATATATATATTTCATTTCATTTC
AM1737        TTTGGAGTTCTTTTCGTTTTTATATTATTTAACAAAGTAATAATAAAAAAA-----TATATACATATATATATATATTTCATTTCATTTC
SC74          TTTGGAGTTCTTTTCGTTTTTATATTATTTAACAAAGTAATAATAAAAAAA-----TATATACATATATATATATATTTCATTTCATTTC
TAB166        TTTGGAGTTCTTTTCGTTTTTATATTATTTAACAAAGTAATAATAAAAAAA-----TATATACATATATATATATATTTCATTTCATTTC
TAB138        TTTGGAGTTCTTTTCGTTTTTATATTATTTAACAAAGTAATAATAAAAAAA-----TATATACATATATATATATATTTCATTTCATTTC
TAB130        TTTGGAGTTCTTTTCGTTTTTATATTATTTAACAAAGTAATAATAAAAAAA-----TATATACATATATATATATATTTCATTTCATTTC
TAB131        TTTGGAGTTCTTTTCGTTTTTATATTATTTAACAAAGTAATAATAAAAAAA-----TATATACATATATATATATATTTCATTTCATTTC
TAB151        TTTGGAGTTCTTTTCGTTTTTATATTATTTAACAAAGTAATAATAAAAAAA-----TATATACATATATATATATATTTCATTTCATTTC
TAB152        TTTGGAGTTCTTTTCGTTTTTATATTATTTAACAAAGTAATAATAAAAAAA-----TATATACATATATATATATATTTCATTTCATTTC
SC52          TTTGGAGTTCTTTTCGTTTTTATATTATTTAACAAAGTAATAATAAAAAAA-----TATATACATATATATATATATTTCATTTCATTTC
SC55          TTTGGAGTTCTTTTCGTTTTTATATTATTTAACAAAGTAATAATAAAAAAA-----TATATACATATATATATATATTTTATTTCATTTC
SC03          TTTGGAGTTCTTTTCGTTTTTATATTATTTAACAAAGTAATAATAAAAAAA-----TATATACATATATATATATATTTCATTTCATTTC
SC53          TTTGGAGTTCTTTTCGTTTTTATATTATTTAACAAAGTAATAATAAAAAAA-----TATATACATATATATATATATTTCATTTCATTTC
TAB141        TTTGGAGTTCTTTTCGTTTTTATATTATTTAACAAAGTAATAATAAAAAAA-----TATATACATATATATATATATTTCATTTCATTTC
TAB123        TTTGGAGTTCTTTTCGTTTTTATATTATTTAACAAAGTAATAATAAAAAAA-----TATATACATATATATATATATTTCATTTCATTTC
AM1803        TTTGGAGTTCTTTTCGTTTTTATATTATTTAACAAAGTAATAATAAAAAAA-----TATATACATATATATATATATTTCATTTCATTTC
AM1814        TTTGGAGTTCTTTTCGTTTTTATATTATTTAACAAAGTAATAATAAAAAAA-----TATATACATATATATATATATTTCATTTCATTTC
AM1802        TTTGGAGTTCTTTTCGTTTTTATATTATTTAACAAAGTAATAATAAAAAAAA----TATGTATATAAATATATATATTTCATTTCATTTC
AM1811        TTTGGAGTTCTTTTCGTTTTTATATTATTTAACAAAGTAATAATAAAAAAA-----TATATACATATATATATATATTTCATTTCATTTC
SC50          TTTGGAGTTCTTTTCGTTTTTATATTATTTAACAAAGTAATAATAAAAAAAA----TATGTATATAAATATATATATTTCATTTCATTTC
SC56          TTTGGAGTTCTTTTCGTTTTTATATTATTTAACAAAGTAATAATAAAAAAA-----TATATACATATATATATATATTTCATTTCATTTC
TAB153        TTTGGAGTTCTTTTCGTTTTTATATTATTTAACAAAGTAATAATAAAAAAA-----TATATACATATATATATATATTTCATTTCATTTC
TAB154        TTTGGAGTTCTTTTCGTTTTTATATTATTTAACAAAGTAATAATAAAAAAA-----TATATACATATATATATATATTTCATTTCATTTC
TAB156        TTTGGAGTTCTTTTCGTTTTTATATTATTTAACAAAGTAATAATAAAAAAA-----TATGTATATAAATATATATATTTCATTTCATTTC
TAB136        TTTGGAGTTCTTTTCGTTTTTATATTATTTAACAAAGTAATAATAAAAAAA-----TATATAAATAAATATATATATTTTATTTTATTTC
AM1880        TTTGGAGTTCTTTTCGTTTTTATATTATTTAACAAAGTAATAATAAAAAAA-----TATGTATATAAATATATATATTTCATTTCATTTC
PF3D7_        TTTGGAGTTCTTTTCGTTTTTATATTATTTAACAAAGTAATAATAAAAAAA-----TATATACATATATATATATATTTCATTTCATTTC
PfSD01        TTTGGAGTTCTTTTCGTTTTTATATTATTTAACAAAGTAATAATAAAAAAA-----TATATACATATATATATATATTTCATTTCATTTC
Pf7G8         TTTGGAGTTCTTTTCGTTTTTATATTATTTAACAAAGTAATAATAAAAAAA-----TATATACATATATATATATATTTCATTTC-----
FCR3 GENE     TTTGGAGTTCTTTTCGTTTTTATATTATTTAACAAAGTAATAATAAAAAAAA----TATGTATATAAATATATATATTTCATTTCATTTC
MS822 GENE     TTTGGAGTTCTTTTCGTTTTTATATTATTTAACAAAGTAATAATAAAAAAA-----TATATACATATATATATATATTTCATTTCATTTC
PfCD01_       TTTGGAGTTCTTTTCGTTTTTATATTATTTAACAAAGTAATAATAAAAAAA-----TATATACATATATATATATATTTCATTTC-----
PfKH01__      TTTGGAGTTCTTTTCGTTTTTATATTATTTAACAAAGTAATAATAAAAAAA-----TATATACATATATATATATAT---ATTTCATTTC
PfDd2__       TTTGGAGTTCTTTTCGTTTTTATATTATTTAACAAAGTAATAATAAAAAAA-----TATATACATATATATATATATTTCATTTCATTTC
PfSN01_       TTTGGAGTTCTTTTCGTTTTTATATTATTTAACAAAGTAATAATAAAAAAA-TATATACATATATATATATATATATTTCATTTCATTTC
PfGN01        TTTGGAGTTCTTTTCGTTTTTATATTATTTAACAAAGTAATAATAAAAAAA-----TATATACATATATATATATATTTCATTTCATTTC
PfKE01        TTTGGAGTTCTTTTCGTTTTTATATTATTTAACAAAGTAATAATAAAAAAA-----TATATACATATATATATATATTTCATTTCATTTC
PfKH02        TTTGGAGTTCTTTTCGTTTTTATATTATTTAACAAAGTAATAATAAAAAAA-----TATATACATATATATATATATTTCATTTCATTTC
PfGB4         TTTGGAGTTCTTTTCGTTTTTATATTATTTAACAAAGTAATAATAAAAAAA-----TATATACATATATATATATATTTCATTTCATTTC
PfGA01_       TTTGGAGTTCTTTTCGTTTTTATATTATTTAACAAAGTAATAATAAAAAAA-----TATATACATATATATATATATTTCATTTCATTTC
PfHB3_        TTTGGAGTTCTTTTCGTTTTTATATTATTTAACAAAGTAATAATAAAAAAA-----TATATACATATATATATATATTTCATTTCATTTC
PfIT_         TTTGGAGTTCTTTTCGTTTTTATATTATTTAACAAAGTAATAATAAAAAAAA----TATGTATATAAATATATATATTTCATTTCATTTC
```

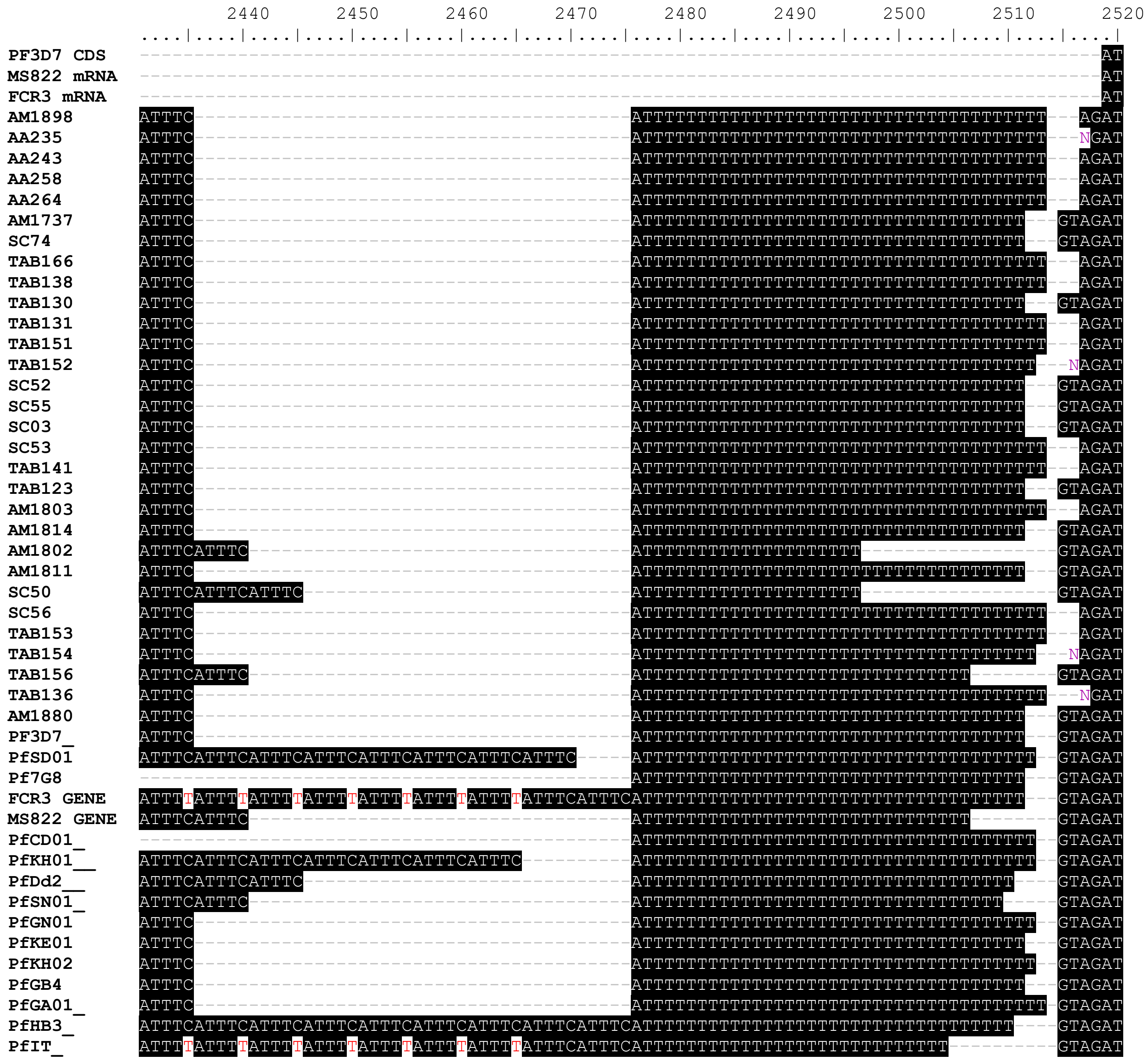
2440
2450
2460
2470
2480
2490
2500
2510
2520
PF3D7 CDS
MS822 mRNA
FCR3 mRNA
AM1898
AA235
AA243
AA258
AA264
AM1737
SC74
TAB166
TAB138
TAB130
TAB131
TAB151
TAB152
SC52
SC55
SC03
SC53
TAB141
TAB123
AM1803
AM1814
AM1802
AM1811
SC50
SC56
TAB153
TAB154
TAB156
TAB136
AM1880
PF3D7_
PfSD01
Pf7G8
FCR3 GENE
MS822 GENE
PfCD01_
PfKH01__
PfDd2__
PfSN01_
PfGN01
PfKE01
PfKH02
PfGB4
PfGA01_
PfHB3_
PfIT_

```
                   2530      2540      2550      2560      2570      2580      2590      2600      2610
             ....|....|....|....|....|....|....|....|....|....|....|....|....|....|....|....|....|....|
PF3D7 CDS    TAATCCTTTTGGAACATTTTCTTCCAAAAAGAAAAAA-GGAAAGTCAGACGAAGAAGGTATGATG--AAAATATTAAAAAGGGAAGGGAA
MS822 mRNA   TAATCCTTTTGGAACATTTTCTTCCAAAAAGAAAAAA-GGAAAGTCAGACGAAGAAGGTATGATGTGAAAATATTAAAAAGGGAAGGGAA
FCR3 mRNA    TAATCCTTTTGGAACATTTTCTTCCAAAAAGAAAAAAAGGAAAGTCAGACGAAGAAGGTATGATGAGAAAATATTAAAAAGGGAAGGGAA
AM1898       TAATCCTTTTGGAACATTTTCTTCCAAAAAGAAAAAA-GGAAAGTCAGACGAAGAAGGTATGATGAGAAAATATTAAAAAGGGAAGGGAA
AA235        TAATCCTTTTGGAACATTTTCTTCCAAAAAGAAAAAA-GGAAAGTCAGACGAAGAAGGTATGATGAGAAAATATTAAAAAGGGAAGGGAA
AA243        TAATCCTTTTGGAACATTTTCTTCCAAAAAGAAAAAA-GGAAAGTCAGACGAAGAAGGTATGATGAGAAAATATTAAAAAGGGAAGGGAA
AA258        TAATCCTTTTGGAACATTTTCTTCCAAAAAGAAAAAA-GGAAAGTCAGACGAAGAAGGTATGATGAGAAAATATTAAAAAGGGAAGGGAA
AA264        TAATCCTTTTGGAACATTTTCTTCCAAAAAGAAAAAA-GGAAAGTCAGACGAAGAAGGTATGATGTGAAAATATTAAAAAGGGAAGGGAA
AM1737       TAATCCTTTTGGAACATTTTCTTCCAAAAAGAAAAAA-GGAAAGTCAGACGAAGAAGGTATGATGTGAAAATATTAAAAAGGGAAGGGAA
SC74         TAATCCTTTTGGAACATTTTCTTCCAAAAAGAAAAAA-GGAAAGTCAGACGAAGAAGGTATGATGAGAAAATATTAAAAAGGGAAGGGAA
TAB166       TAATCCTTTTGGAACATTTTCTTCCAAAAAGAAAAAA-GGAAAGTCAGACGAAGAAGGTATGATGAGAAAATATTAAAAAGGGAAGGGAA
TAB138       TAATCCTTTTGGAACATTTTCTTCCAAAAAGAAAAAA-GGAAAGTCAGACGAAGAAGGTATGATGTGAAAATATTAAAAAGGGAAGGGAA
TAB130       TAATCCTTTTGGAACATTTTCTTCCAAAAAGAAAAAA-GGAAAGTCAGACGAAGAAGGTATGATGTGAAAATATTAAAAAGGGAAGGGAA
TAB131       TAATCCTTTTGGAACATTTTCTTCCAAAAAGAAAAAA-GGAAAGTCAGACGAAGAAGGTATGATGTGAAAATATTAAAAAGGGAAGGGAA
TAB151       TAATCCTTTTGGAACATTTTCTTCCAAAAAGAAAAAA-GGAAAGTCAGACGAAGAAGGTATGATGTGAAAATATTAAAAAGGGAAGGGAA
TAB152       TAATCCTTTTGGAACATTTTCTTCCAAAAAGAAAAAA-GGAAAGTCAGACGAAGAAGGTATGATGTGAAAATATTAAAAAGGGAAGGGAA
SC52         TAATCCTTTTGGAACATTTTCTTCCAAAAAGAAAAAA-GGAAAGTCAGACGAAGAAGGTATGATGTGAAAATATTAAAAAGGGAAGGGAA
SC55         TAATCCTTTTGGAACATTTTCTTCCAAAAAGAAAAAA-GGAAAGTCAGACGAAGAAGGTATGATGAGAAAATATTAAAAAGGGAAGGGAA
SC03         TAATCCTTTTGGAACATTTTCTTCCAAAAAGAAAAAA-GGAAAGTCAGACGAAGAAGGTATGATGTGAAAATATTAAAAAGGGAAGGGAA
SC53         TAATCCTTTTGGAACATTTTCTTCCAAAAAGAAAAAA-GGAAAGTCAGACGAAGAAGGTATGATGTGAAAATATTAAAAAGGGAAGGGAA
TAB141       TAATCCTTTTGGAACATTTTCTTCCAAAAAGAAAAAA-GGAAAGTCAGACGAAGAAGGTATGATGAGAAAATATTAAAAAGGGAAGGGAA
TAB123       TAATCCTTTTGGAACATTTTCTTCCAAAAAGAAAAAA-GGAAAGTCAGACGAAGAAGGTATGATGTGAAAATATTAAAAAGGGAAGGGAA
AM1803       TAATCCTTTTGGAACATTTTCTTCCAAAAAGAAAAAAAGGAAAGTCAGACGAAGAAGGTATGATGAGAAAATATTAAAAAGGGAAGGGAA
AM1814       TAATCCTTTTGGAACATTTTCTTCCAAAAAGAAAAAAAGGAAAGTCAGACGAAGAAGGTATGATGAGAAAATATTAAAAAGGGAAGGGAA
AM1802       TAATCCTTTTGGAACATTTTCTTCCAAAAAGAAAAAAAGGAAAGTCAGACGAAGAAGGTATGATGAGAAAATATTAAAAAGGGAAGGGAA
AM1811       TAATCCTTTTGGAACATTTTCTTCCAAAAAGAAAAAAAGGAAAGTCAGACGAAGAAGGTATGATGAGAAAATATTAAAAAGGGAAGGGAA
SC50         TAATCCTTTTGGAACATTTTCTTCCAAAAAGAAAAAAAGGAAAGTCAGACGAAGAAGGTATGATGAGAAAATATTAAAAAGGGAAGGGAA
SC56         TAATCCTTTTGGAACATTTTCTTCCAAAAAGAAAAAAAGGAAAGTCAGACGAAGAAGGTATGATGAGAAAATATTAAAAAGGGAAGGGAA
TAB153       TAATCCTTTTGGAACATTTTCTTCCAAAAAGAAAAAAAGGAAAGTCAGACGAAGAAGGTATGATGAGAAAATATTAAAAAGGGAAGGGAA
TAB154       TAATCCTTTTGAAACATTTTCTTCCAAAAAGAAAAAAAGGAAAGTCAGACGAAGAAGGTATGATGAGAAAATATTAAAAAGGGAAGGGAA
TAB156       TAATCCTTTTGGAACATTTTCTTCCAAAAAGAAAAAAAGGAAAGTCAGACGAAGAAGGTATGATGAGAAAATATTAAAAAGGGAAGGGAA
TAB136       TAATCCTTTTGGAACATTTTCTTCCAAAAAGAAAAAAAGGAAAGTCAGACGAAGAAGGTATGATGAGAAAATATTAAAAAGGGAAGGGAA
AM1880       TAATCCTTTTGGAACATTTTCTTCCAAAAAGAAAAAAAGGAAAGTCAGACGAAGAAGGTATGATGAGAAAATATTAAAAAGGGAAGGGAA
PF3D7_       TAATCCTTTTGGAACATTTTCTTCCAAAAAGAAAAAA-GGAAAGTCAGACGAAGAAGGTATGATGTGAAAATATTAAAAAGGGAAGGGAA
PfSD01       TAATCCTTTTGGAACATTTTCTTCCAAAAAGAAAAAAAGGAAAGTCAGACGAAGAAGGTATGATGAGAAAATATTAAAAAGGGAAGGGAA
Pf7G8        TAATCCTTTTGAAACATTTTCTTCCAAAAAGAAAAAAAGGAAAGTCAGACGAAGAAGGTATGATGAGAAAATATTAAAAAGGGAAGGGAA
FCR3 GENE    TAATCCTTTTGGAACATTTTCTTCCAAAAAGAAAAAAAGGAAAGTCAGACGAAGAAGGTATGATGAGAAAATATTAAAAAGGGAAGGGAA
MS822 GENE   TAATCCTTTTGGAACATTTTCTTCCAAAAAGAAAAAA-GGAAAGTCAGACGAAGAAGGTATGATGTGAAAATATTAAAAAGGGAAGGGAA
PfCD01_      TAATCCTTTTGAAACATTTTCTTCCAAAAAGAAAAAAAGGAAAGTCAGACGAAGAAGGTATGATGAGAAAATATTAAAAAGGGAAGGGAA
PfKH01__     TAATCCTTTTGGAACATTTTCTTCCAAAAAGAAAAAA-GGAAAGTCAGACGAAGAAGGTATGATGTGAAAATATTAAAAAGGGAAGGGAA
PfDd2__      TAATCCTTTTGGAACATTTTCTTCCAAAAAGAAAAAA-GGAAAGTCAGACGAAGAAGGTATGATGTGAAAATATTAAAAAGGGAAGGGAA
PfSN01_      TAATCCTTTTGGAACATTTTCTTCCAAAAAGAAAAAA-GGAAAGTCAGACGAAGAAGGTATGATGTGAAAATATTAAAAAGGGAAGGGAA
PfGN01       TAATCCTTTTGGAACATTTTCTTCCAAAAAGAAAAAA-GGAAAGTCAGACGAAGAAGGTATGATGTGAAAATATTAAAAAGGGAAGGGAA
PfKE01       TAATCCTTTTGGAACATTTTCTTCCAAAAAGAAAAAA-GGAAAGTCAGACGAAGAAGGTATGATGTGAAAATATTAAAAAGGGAAGGGAA
PfKH02       TAATCCTTTTGGAACATTTTCTTCCAAAAAGAAAAAA-GGAAAGTCAGACGAAGAAGGTATGATGTGAAAATATTAAAAAGGGAAGGGAA
PfGB4        TAATCCTTTTGGAACATTTTCTTCCAAAAAGAAAAAA-GGAAAGTCAGACGAAGAAGGTATGATGTGAAAATATTAAAAAGGGAAGGGAA
PfGA01_      TAATCCTTTTGGAACATTTTCTTCCAAAAAGAAAAAA-GGAAAGTCAGACGAAGAAGGTATGATGTGAAAATATTAAAAAGGGAAGGGAA
PfHB3_       TAATCCTTTTGAAACATTTTCTTCCAAAAAGAAAAAAAGGAAAGTCAGACGAAGAAGGTATGATGAGAAAATATTAAAAAGGGAAGGGAA
PfIT_        TAATCCTTTTGGAACATTTTCTTCCAAAAAGAAAAAAAGGAAAGTCAGACGAAGAAGGTATGATGAGAAAATATTAAAAAGGGAAGGGAA
```

```
                 2620      2630      2640      2650      2660      2670      2680      2690      2700
        ....|....|....|....|....|....|....|....|....|....|....|....|....|....|....|....|....|....|
PF3D7 CDS    AAAATGTTTTGAGAATTTAATGCAGCATTAAAAGGAATGGATTGATAAAGAAAGTCATGATATATTACCAAACAGTTTATATAATATTGA
MS822 mRNA   AAAATGTTTTGAGAATTTAATGCAGCATTAAAAGGAATGGATTGATAAAGAAAGTCATGATATATTACCAAACAGTTTATATAATATTGA
FCR3 mRNA    AAAATGTTTTGAGAATTTAATGCAGCATTCAAAGGAATGGATTGATAAAGAAAGATCTGATATATTACCAAACAGTTTATATAATATTGA
AM1898       AAAATGTTTTGAGAATTTAATGCAGCATTCAAAGGAATGGATTGATAAAGAAAGACATGATATATTACCAAACAGTTTATATAATATTGA
AA235        AAAATGTTTTGAGAATTTAATGCAGCATTCAAAGGAATGGATTGATAAAGAAAGTCATGATATATTACCAAACAGTTTATATAATATTGA
AA243        AAAATGTTTTGAGAATTTAATGCAGCATTCAAAGGAATGGATTGATAAAGAAAGTCATGATATATTACCAAACAGTTTATATAATATTGA
AA258        AAAATGTTTTGAGAATTTAATGCAGCATTCAAAGGAATGGATTGATAAAGAAAGTCATGATATATTACCAAACAGTTTATATAATATTGA
AA264        AAAATGTTTTGAGAATTTAATGCAGCATTAAAAGGAATGGATTGATAAAGAAAGTCATGATATATTACCAAACAGTTTATATAATATTGA
AM1737       AAAATGTTTTGAGAATTTAATGCAGCATTAAAAGGAATGGATTGATAAAGAAAGTCATGATATATTACCAAACAGTTTATATAATATTGA
SC74         AAAATGTTTTGAGAATTTAATGCAGCATTCAAAGGAATGGATTGATAAAGAAAGTCATGATATATTACCAAACAGTTTATATAATATTGA
TAB166       AAAATGTTTTGAGAATTTAATGCAGCATTCAAAGGAATGGATTGATAAAGAAAGTCATGATATATTACCAAACAGTTTATATAATATTGA
TAB138       AAAATGTTTTGAGAATTTAATGCAGCATTAAAAGGAATGGATTGATAAAGAAAGTCATGATATATTACCAAACAGTTTATATAATATTGA
TAB130       AAAATGTTTTGAGAATTTAATGCAGCATTAAAAGGAATGGATTGATAAAGAAAGTCATGATATATTACCAAACAGTTTATATAATATTGA
TAB131       AAAATGTTTTGAGAATTTAATGCAGCATTAAAAGGAATGGATTGATAAAGAAAGTCATGATATATTACCAAACAGTTTATATAATATTGA
TAB151       AAAATGTTTTGAGAATTTAATGCAGCATTAAAAGGAATGGATTGATAAAGAAAGTCATGATATATTACCAAACAGTTTATATAATATTGA
TAB152       AAAATGTTTTGAGAATTTAATGCAGCATTAAAAGGAATGGATTGATAAAGAAAGTCATGATATATTACCAAACAGTTTATATAATATTGA
SC52         AAAATGTTTTGAGAATTTAATGCAGCATTAAAAGGAATGGATTGATAAAGAAAGTCATGATATATTACCAAACAGTTTATATAATATTGA
SC55         AAAATGTTTTGAGAATTTAATGCAGCATTCAAAGGAATGGATTGATAAAGAAAGACATGATATATTACCAAACAGTTTATATAATATTGA
SC03         AAAATGTTTTGAGAATTTAATGCAGCATTAAAAGGAATGGATTGATAAAGAAAGTCATGATATATTACCAAACAGTTTATATAATATTGA
SC53         AAAATGTTTTGAGAATTTAATGCAGCATTAAAAGGAATGGATTGATAAAGAAAGTCATGATATATTACCAAACAGTTTATATAATATTGA
TAB141       AAAATGTTTTGAGAATTTAATGCAGCATTCAAAGGAATGGATTGATAAAGAAAGTCATGATATATTACCAAACAGTTTATATAATATTGA
TAB123       AAAATGTTTTGAGAATTTAATGCAGCATTAAAAGGAATGGATTGATAAAGAAAGTCATGATATATTACCAAACAGTTTATATAATATTGA
AM1803       AAAATGTTTTGAGAATTTAATGCAGCATTCAAAGGAATGGATTGATAAAGAAAGTCATGATATATTACCAAACAGTTTATATAATATTGA
AM1814       AAAATGTTTTGAGAATTTAATGCAGCATTCAAAGGAATGGATTGATAAAGAAAGTCATGATATATTACCAAACAGTTTATATAATATTGA
AM1802       AAAATGTTTTGAGAATTTAATGCAGCATTCAAAGGAATGGATTGATAAAGAAAGTCATGATATATTACCAAACAGTTTATATAATATTGA
AM1811       AAAATGTTTTGAGAATTTAATGCAGCATTCAAAGGAATGGATTGATAAAGAAAGTCATGATATATTACCAAACAGTTTATATAATATTGA
SC50         AAAATGTTTTGAGAATTTAATGCAGCATTCAAAGGAATGGATTGATAAAGAAAGTCATGATATATTACCAAACAGTTTATATAATATTGA
SC56         AAAATGTTTTGAGAATTTAATGCAGCATTCAAAGGAATGGATTGATAAAGAAAGTCATGATATATTACCAAACAGTTTATATAATATTGA
TAB153       AAAATGTTTTGAGAATTTAATGCAGCATTCAAAGGAATGGATTGATAAAGAAAGTCATGATATATTACCAAACAGTTTATATAATATTGA
TAB154       AAAATGTTTTGAGAATTTAATGCAGCATTCAAAGGAATGGATTGATAAAGAAAGTCATGATATATTACCAAACAGTTTATATAATATTGA
TAB156       AAAATGTTTTGAGAATTTAATGCAGCATTCAAAGGAATGGATTGATAAAGAAAGTCATGATATATTACCAAACAGTTTATATAATATTGA
TAB136       AAAATGTTTTGAGAATTTAATGCAGCATTCAAAGGAATGGATTGATAAAGAAAGACATGATATATTACCAAACAGTTTATATAATATTGA
AM1880       AAAATGTTTTGAGAATTTAATGCAGCATTCAAAGGAATGGATTGATAAAGAAAGACATGATATATTACCAAACAGTTTATATAATATTGA
PF3D7_       AAAATGTTTTGAGAATTTAATGCAGCATTAAAAGGAATGGATTGATAAAGAAAGTCATGATATATTACCAAACAGTTTATATAATATTGA
PfSD01       AAAATGTTTTGAGAATTTAATGCAGCATTCAAAGGAATGGATTGATAAAGAAAGTCATGATATATTACCAAACAGTTTATATAATATTGA
Pf7G8        AAAATGTTTTGAGAATTTAATGCAGCATTCAAAGGAATGGATTGATAAAGAAAGTCATGATATATTACCAAACAGTTTATATAATATTGA
FCR3 GENE    AAAATGTTTTGAGAATTTAATGCAGCATTCAAAGGAATGGATTGATAAAGAAAGATCTGATATATTACCAAACAGTTTATATAATATTGA
MS822 GENE   AAAATGTTTTGAGAATTTAATGCAGCATTAAAAGGAATGGATTGATAAAGAAAGTCATGATATATTACCAAACAGTTTATATAATATTGA
PfCD01_      AAAATGTTTTGAGAATTTAATGCAGCATTCAAAGGAATGGATTGATAAAGAAAGTCATGATATATTACCAAACAGTTTATATAATATTGA
PfKH01__     AAAATGTTTTGAGAATTTAATGCAGCATTAAAAGGAATGGATTGATAAAGAAAGTCATGATATATTACCAAACAGTTTATATAATATTGA
PfDd2__      AAAATGTTTTGAGAATTTAATGCAGCATTCAAAGGAATGGATTGATAAAGAAAGTCATGATATATTACCAAACAGTTTATATAATATTGA
PfSN01_      AAAATGTTTTGAGAATTTAATGCAGCATTAAAAGGAATGGATTGATAAAGAAAGTCATGATATATTACCAAACAGTTTATATAATATTGA
PfGN01       AAAATGTTTTGAGAATTTAATGCAGCATTAAAAGGAATGGATTGATAAAGAAAGTCATGATATATTACCAAACAGTTTATATAATATTGA
PfKE01       AAAATGTTTTGAGAATTTAATGCAGCATTAAAAGGAATGGATTGATAAAGAAAGTCATGATATATTACCAAACAGTTTATATAATATTGA
PfKH02       AAAATGTTTTGAGAATTTAATGCAGCATTAAAAGGAATGGATTGATAAAGAAAGTCATGATATATTACCAAACAGTTTATATAATATTGA
PfGB4        AAAATGTTTTGAGAATTTAATGCAGCATTAAAAGGAATGGATTGATAAAGAAAGTCATGATATATTACCAAACAGTTTATATAATATTGA
PfGA01_      AAAATGTTTTGAGAATTTAATGCAGCATTCAAAGGAATGGATTGATAAAGAAAGACATGATATATTACCAAACAGTTTATATAATATTGA
PfHB3_       AAAATGTTTTGAGAATTTAATGCAGCATTCAAAGGAATGGATTGATAAAGAAAGTCATGATATATTACCAAACAGTTTATATAATATTGA
PfIT_        AAAATGTTTTGAGAATTTAATGCAGCATTCAAAGGAATGGATTGATAAAGAAAGACATGATATATTACCAAACAGTTTATATAATATTGA
```

```
                2710      2720      2730      2740      2750      2760      2770      2780      2790
            ....|....|....|....|....|....|....|....|....|....|....|....|....|....|....|....|....|....|
PF3D7 CDS   AAAAAAAGAAGGAACGGAGTACAACGTTTTAGAGGAAAAGGAATATCCACTAATAGATGAAGTTTTGAAAGAAGAAGATATAAAAAAGGT
MS822 mRNA  AAAAAAAGAAGGAACGGAGTACAACGTTTTAGAGGAAAAGGAATATCCACTAATAGATGAAGTTTTGAAAGAAGAAGATATAAA------
FCR3 mRNA   AAAAAAGGAAGGAACGGAGTACAACGTTTTAGAGGAAAAGGAATATCCACTAATAGATGAAGTTTTGAATGAAGAAGATATAAAAAAGGT
AM1898      AAAAAAGGAAGGAACGGAGTACAACGTTTTAGAGGAAAAGGAATATCCACTAATAGATGAAGTTTTGAAAGAAGAAGATATAAAAAAGGT
AA235       AAAAAAAGAAGGAACGGAGTACAACGTTTTAGAGGAAAAGGAATATCCACTAATAGATGAAGTTTTGAAAGAAGAAGATATAAAAAAGGT
AA243       AAAAAAAGAAGGAACGGAGTACAACGTTTTAGAGGAAAAGGAATATCCACTAATAGATGAAGTTTTGAAAGAAGAAGATATAAAAAAGGT
AA258       AAAAAAAGAAGGAACGGAGTACAACGTTTTAGAGGAAAAGGAATATCCACTAATAGATGAAGTTTTGAAAGAAGAAGATATAAAAAAGGT
AA264       AAAAAAAGAAGGAACGGAGTACAACGTTTTAGAGGAAAAGGAATATCCACTAATAGATGAAGTTTTGAAAGAAGAAGATATAAAAAAGGT
AM1737      AAAAAAAGAAGGAACGGAGTACAACGTTTTAGAGGAAAAGGAATATCCACTAATAGATGAAGTTTTGAAAGAAGAAGATATAAAAAAGGT
SC74        AAAAAAAGAAGGAACGGAGTACAACGTTTTAGAGGAAAAGGAATATCCACTAATAGATGAAGTTTTGAAAGAAGAAGATATAAAAAAGGT
TAB166      AAAAAAAGAAGGAACGGAGTACAACGTTTTAGAGGAAAAGGAATATCCACTAATAGATGAAGTTTTGAAAGAAGAAGATATAAAAAAGGT
TAB138      AAAAAAAGAAGGAACGGAGTACAACGTTTTAGAGGAAAAGGAATATCCACTAATAGATGAAGTTTTGAAAGAAGAAGATATAAAAAAGGT
TAB130      AAAAAAAGAAGGAACGGAGTACAACGTTTTAGAGGAAAAGGAATATCCACTAATAGATGAAGTTTTGAAAGAAGAAGATATAAAAAAGGT
TAB131      AAAAAAAGAAGGAACGGAGTACAACGTTTTAGAGGAAAAGGAATATCCACTAATAGATGAAGTTTTGAAAGAAGAAGATATAAAAAAGGT
TAB151      AAAAAAAGAAGGAACGGAGTACAACGTTTTAGAGGAAAAGGAATATCCACTAATAGATGAAGTTTTGAAAGAAGAAGATATAAAAAAGGT
TAB152      AAAAAAAGAAGGAACGGAGTACAACGTTTTAGAGGAAAAGGAATATCCACTAATAGATGAAGTTTTGAAAGAAGAAGATATAAAAAAGGT
SC52        AAAAAAAGAAGGAACGGAGTACAACGTTTTAGAGGAAAAGGAATATCCACTAATAGATGAAGTTTTGAAAGAAGAAGATATAAAAAAGGT
SC55        AAAAAAGGAAGGAACGGAGTACAACGTTTTAGAGGAAAAGGAATATCCACTAATAGATGAAGTTTTGAAAGAAGAAGATATAAAAAAGGT
SC03        AAAAAAAGAAGGAACGGAGTACAACGTTTTAGAGGAAAAGGAATATCCACTAATAGATGAAGTTTTGAAAGAAGAAGATATAAAAAAGGT
SC53        AAAAAAAGAAGGAACGGAGTACAACGTTTTAGAGGAAAAGGAATATCCACTAATAGATGAAGTTTTGAAAGAAGAAGATATAAAAAAGGT
TAB141      AAAAAAAGAAGGAACGGAGTACAACGTTTTAGAGGAAAAGGAATATCCACTAATAGATGAAGTTTTGAAAGAAGAAGATATAAAAAAGGT
TAB123      AAAAAAAGAAGGAACGGAGTACAACGTTTTAGAGGAAAAGGAATATCCACTAATAGATGAAGTTTTGAAAGAAGAAGATATAAAAAAGGT
AM1803      AAAAAAAGAAGGAACGGAGTACAACGTTTTAGAGGAAAAGGAATATCCACTAATAGATGAAGTTTTGAAAGAAGAAGATATAAAAAAGGT
AM1814      AAAAAAAGAAGGAACGGAGTACAACGTTTTAGAGGAAAAGGAATATCCACTAATAGATGAAGTTTTGAAAGAAGAAGATATAAAAAAGGT
AM1802      AAAAAAAGAAGGAACGGAGTACAACGTTTTAGAGGAAAAGGAATATCCACTAATAGATGAAGTTTTGAAAGAAGAAGATATAAAAAAGGT
AM1811      AAAAAAAGAAGGAACGGAGTACAACGTTTTAGAGGAAAAGGAATATCCACTAATAGATGAAGTTTTGAAAGAAGAAGATATAAAAAAGGT
SC50        AAAAAAAGAAGGAACGGAGTACAACGTTTTAGAGGAAAAGGAATATCCACTAATAGATGAAGTTTTGAAAGAAGAAGATATAAAAAAGGT
SC56        AAAAAAAGAAGGAACGGAGTACAACGTTTTAGAGGAAAAGGAATATCCACTAATAGATGAAGTTTTGAAAGAAGAAGATATAAAAAAGGT
TAB153      AAAAAAAGAAGGAACGGAGTACAACGTTTTAGAGGAAAAGGAATATCCACTAATAGATGAAGTTTTGAAAGAAGAAGATATAAAAAAGGT
TAB154      AAAAAAAGAAGGAACGGAGTACAACGTTTTAGAGGAAAAGGAATATCCACTAATAGATGAAGTTTTGAAAGAAGAAGATATAAAAAAGGT
TAB156      AAAAAAAGAAGGAACGGAGTACAACGTTTTAGAGGAAAAGGAATATCCACTAATAGATGAAGTTTTGAAAGAAGAAGATATAAAAAAGGT
TAB136      AAAAAAGGAAGGAACGGAGTACAACGTTTTAGAGGAAAAGGAATATCCACTAATAGATGAAGTTTTGAAAGAAGAAGATATAAAAAAGGT
AM1880      AAAAAAGGAAGGAACGGAGTACAACGTTTTAGAGGAAAAGGAATATCCACTAATAGATGAAGTTTTGAAAGAAGAAGATATAAAAAAGGT
PF3D7_      AAAAAAAGAAGGAACGGAGTACAACGTTTTAGAGGAAAAGGAATATCCACTAATAGATGAAGTTTTGAAAGAAGAAGATATAAAAAAGGT
PfSD01      AAAAAAAGAAGGAACGGAGTACAACGTTTTAGAGGAAAAGGAATATCCACTAATAGATGAAGTTTTGAAAGAAGAAGATATAAAAAAGGT
Pf7G8       AAAAAAAGAAGGAACGGAGTACAACGTTTTAGAGGAAAAGGAATATCCACTAATAGATGAAGTTTTGAAAGAAGAAGATATAAAAAAGGT
FCR3 GENE   AAAAAAGGAAGGAACGGAGTACAACGTTTTAGAGGAAAAGGAATATCCACTAATAGATGAAGTTTTGAATGAAGAAGATATAAAAAAGGT
MS822 GENE  AAAAAAAGAAGGAACGGAGTACAACGTTTTAGAGGAAAAGGAATATCCACTAATAGATGAAGTTTTGAAAGAAGAAGATATAAA------
PfCD01_     AAAAAAAGAAGGAACGGAGTACAACGTTTTAGAGGAAAAGGAATATCCACTAATAGATGAAGTTTTGAAAGAAGAAGATATAAAAAAGGT
PfKH01__    AAAAAAAGAAGGAACGGAGTACAACGTTTTAGAGGAAAAGGAATATCCACTAATAGATGAAGTTTTGAAAGAAGAAGATATAAAAAAGGT
PfDd2__     AAAAAAAGAAGGAACGGAGTACAACGTTTTAGAGGAAAAGGAATATCCACTAATAGATGAAGTTTTGAAAGAAGAAGATATAAAAAAGGT
PfSN01_     AAAAAAAGAAGGAACGGAGTACAACGTTTTAGAGGAAAAGGAATATCCACTAATAGATGAAGTTTTGAAAGAAGAAGATATAAAAAAGGT
PfGN01      AAAAAAAGAAGGAACGGAGTACAACGTTTTAGAGGAAAAGGAATATCCACTAATAGATGAAGTTTTGAAAGAAGAAGATATAAAAAAGGT
PfKE01      AAAAAAAGAAGGAACGGAGTACAACGTTTTAGAGGAAAAGGAATATCCACTAATAGATGAAGTTTTGAAAGAAGAAGATATAAAAAAGGT
PfKH02      AAAAAAAGAAGGAACGGAGTACAACGTTTTAGAGGAAAAGGAATATCCACTAATAGATGAAGTTTTGAAAGAAGAAGATATAAAAAAGGT
PfGB4       AAAAAAAGAAGGAACGGAGTACAACGTTTTAGAGGAAAAGGAATATCCACTAATAGATGAAGTTTTGAAAGAAGAAGATATAAAAAAGGT
PfGA01_     AAAAAAAGAAGGAACGGAGTACAACGTTTTAGAGGAAAAGGAATATCCACTAATAGATGAAGTTTTGAAAGAAGAAGATATAAAAAAGGT
PfHB3_      AAAAAAAGAAGGAACGGAGTACAACGTTTTAGAGGAAAAGGAATATCCACTAATAGATGAAGTTTTGAAAGAAGAAGATATAAAAAAGGT
PfIT_       AAAAAAGGAAGGAACGGAGTACAACGTTTTAGAGGAAAAGGAATATCCACTAATAGATGAAGTTTTGAATGAAGAAGATATAAAAAAGGT
```

```
                       2800      2810      2820      2830      2840      2850      2860      2870      2880
              ....|....|....|....|....|....|....|....|....|....|....|....|....|....|....|....|....|....|
PF3D7 CDS     GAATAAAAAGAAAATAAAAAAGGGACATGTAAAAAGCGTTTTGATAGATAATGTAAAGAAAGATAGGGATATACCAAATCTTATAAAATT
MS822 mRNA    ------------------------------------------------------------------------------------------
FCR3 mRNA     GAATAAAAACAAAATAAAAAAGGGACATGTAAAAAGCGTTTTGATAGATAATGTAAAGAAAGATAGGGATATACCAAATCTTATAAAATT
AM1898        GAATAAAAAGAAAATAAAAAAGGGACATGTAAAAAGCGTTTTGATAGATAATGTAAAGAAAGATAGGGATATACCAAATCTTATAAAATT
AA235         GAATAAAAAGAAAATAAAAAAGGGACATGTAAAAAGCGTTTTGATAGATAATGTAAAGAAAGATAGGGATATACCAAATCTTATAAAATT
AA243         GAATAAAAAGAAAATAAAAAAGGGACATGTAAAAAGCGTTTTGATAGATAATGTAAAGAAAGATAGGGATATACCAAATCTTATAAAATT
AA258         GAATAAAAAGAAAATAAAAAAGGGACATGTAAAAAGCGTTTTGATAGATAATGTAAAGAAAGATAGGGATATACCAAATCTTATAAAATT
AA264         GAATAAAAAGAAAATAAAAAAGGGACATGTAAAAAGCGTTTTGATAGATAATGTAAAGAAAGATAGGGATATACCAAATCTTATAAAATT
AM1737        GAATAAAAAGAAAATAAAAAAGGGACATGTAAAAAGCGTTTTGATAGATAATGTAAAGAAAGATAGGGATATACCAAATCTTATAAAATT
SC74          GAATAAAAAGAAAATAAAAAAGGGACATGTAAAAAGCGTTTTGATAGATAATGTAAAGAAAGATAGGGATATACCAAATCTTATAAAATT
TAB166        GAATAAAAAGAAAATAAAAAAGGGACATGTAAAAAGCGTTTTGATAGATAATGTAAAGAAAGATAGGGATATACCAAATCTTATAAAATT
TAB138        GAATAAAAAGAAAATAAAAAAGGGACATGTAAAAAGCGTTTTGATAGATAATGTAAAGAAAGATAGGGATATACCAAATCTTATAAAATT
TAB130        GAATAAAAAGAAAATAAAAAAGGGACATGTAAAAAGCGTTTTGATAGATAATGTAAAGAAAGATAGGGATATACCAAATCTTATAAAATT
TAB131        GAATAAAAAGAAAATAAAAAAGGGACATGTAAAAAGCGTTTTGATAGATAATGTAAAGAAAGATAGGGATATACCAAATCTTATAAAATT
TAB151        GAATAAAAAGAAAATAAAAAAGGGACATGTAAAAAGCGTTTTGATAGATAATGTAAAGAAAGATAGGGATATACCAAATCTTATAAAATT
TAB152        GAATAAAAAGAAAATAAAAAAGGGACATGTAAAAAGCGTTTTGATAGATAATGTAAAGAAAGATAGGGATATACCAAATCTTATAAAATT
SC52          GAATAAAAAGAAAATAAAAAAGGGACATGTAAAAAGCGTTTTGATAGATAATGTAAAGAAAGATAGGGATATACCAAATCTTATAAAATT
SC55          GAATAAAAAGAAAATAAAAAAGGGACATGTAAAAAGCGTTTTGATAGATAATGTAAAGAAAGATAGGGATATACCAAATCTTATAAAATT
SC03          GAATAAAAAGAAAATAAAAAAGGGACATGTAAAAAGCGTTTTGATAGATAATGTAAAGAAAGATAGGGATATACCAAATCTTATAAAATT
SC53          GAATAAAAAGAAAATAAAAAAGGGACATGTAAAAAGCGTTTTGATAGATAATGTAAAGAAAGATAGGGATATACCAAATCTTATAAAATT
TAB141        GAATAAAAAGAAAATAAAAAAGGGACATGTAAAAAGCGTTTTGATAGATAATGTAAAGAAAGATAGGGATATACCAAATCTTATAAAATT
TAB123        GAATAAAAAGAAAATAAAAAAGGGACATGTAAAAAGCGTTTTGATAGATAATGTAAAGAAAGATAGGGATATACCAAATCTTATAAAATT
AM1803        GAATAAAAAGAAAATAAAAAAGGGACATGTAAAAAGCGTTTTGATAGATAATGTAAAGAAAGATAGGGATATACCAAATCTTATAAAATT
AM1814        GAATAAAAAGAAAATAAAAAAGGGACATGTAAAAAGCGTTTTGATAGATAATGTAAAGAAAGATAGGGATATACCAAATCTTATAAAATT
AM1802        GAATAAAAAGAAAATAAAAAAGGGACATGTAAAAAGCGTTTTGATAGATAATGTAAAGAAAGATAGGGATATACCAAATCTTATAAAATT
AM1811        GAATAAAAAGAAAATAAAAAAGGGACATGTAAAAAGCGTTTTGATAGATAATGTAAAGAAAGATAGGGATATACCAAATCTTATAAAATT
SC50          GAATAAAAAGAAAATAAAAAAGGGACATGTAAAAAGCGTTTTGATAGATAATGTAAAGAAAGATAGGGATATACCAAATCTTATAAAATT
SC56          GAATAAAAAGAAAATAAAAAAGGGACATGTAAAAAGCGTTTTGATAGATAATGTAAAGAAAGATAGGGATATACCAAATCTTATAAAATT
TAB153        GAATAAAAAGAAAATAAAAAAGGGACATGTAAAAAGCGTTTTGATAGATAATGTAAAGAAAGATAGGGATATACCAAATCTTATAAAATT
TAB154        GAATAAAAAGAAAATAAAAAAGGGACATGTAAAAAGCGTTTTGATAGATAATGTAAAGAAAGATAGGGATATACCAAATCTTATAAAATT
TAB156        GAATAAAAAGAAAATAAAAAAGGGACATGTAAAAAGCGTTTTGATAGATAATGTAAAGAAAGATAGGGATATACCAAATCTTATAAAATT
TAB136        GAATAAAAAGAAAATAAAAAAGGGACATGTAAAAAGCGTTTTGATAGATAATGTAAAGAAAGATAGGGATATACCAAATCTTATAAAATT
AM1880        GAATAAAAAGAAAATAAAAAAGGGACATGTAAAAAGCGTTTTGATAGATAATGTAAAGAAAGATAGGGATATACCAAATCTTATAAAATT
PF3D7_        GAATAAAAAGAAAATAAAAAAGGGACATGTAAAAAGCGTTTTGATAGATAATGTAAAGAAAGATAGGGATATACCAAATCTTATAAAATT
PfSD01        GAATAAAAAGAAAATAAAAAAGGGACATGTAAAAAGCGTTTTGATAGATAATGTAAAGAAAGATAGGGATATACCAAATCTTATAAAATT
Pf7G8         GAATAAAAAGAAAATAAAAAAGGGACATGTAAAAAGCGTTTTGATAGATAATGTAAAGAAAGATAGGGATATACCAAATCTTATAAAATT
FCR3 GENE     GAATAAAAACAAAATAAAAAAGGGACATGTAAAAAGCGTTTTGATAGATAATGTAAAGAAAGATAGGGATATACCAAATCTTATAAAATT
MS822 GENE    ------------------------------------------------------------------------------------------
PfCD01_       GAATAAAAAGAAAATAAAAAAGGGACATGTAAAAAGCGTTTTGATAGATAATGTAAAGAAAGATAGGGATATACCAAATCTTATAAAATT
PfKH01__      GAATAAAAAGAAAATAAAAAAGGGACATGTAAAAAGCGTTTTGATAGATAATGTAAAGAAAGATAGGGATATACCAAATCTTATAAAATT
PfDd2__       GAATAAAAAGAAAATAAAAAAGGGACATGTAAAAAGCGTTTTGATAGATAATGTAAAGAAAGATAGGGATATACCAAATCTTATAAAATT
PfSN01_       GAATAAAAAGAAAATAAAAAAGGGACATGTAAAAAGCGTTTTGATAGATAATGGAAAGAAAGATAGGGATATACCAAATCTTATAAAATT
PfGN01_       GAATAAAAAGAAAATAAAAAAGGGACATGTAAAAAGCGTTTTGATAGATAATGGAAAGAAAGATAGGGATATACCAAATCTTATAAAATT
PfKE01        GAATAAAAAGAAAATAAAAAAGGGACATGTAAAAAGCGTTTTGATAGATAATGTAAAGAAAGATAGGGATATACCAAATCTTATAAAATT
PfKH02        GAATAAAAAGAAAATAAAAAAGGGACATGTAAAAAGCGTTTTGATAGATAATGTAAAGAAAGATAGGGATATACCAAATCTTATAAAATT
PfGB4         GAATAAAAAGAAAATAAAAAAGGGACATGTAAAAAGCGTTTTGATAGATAATGTAAAGAAAGATAGGGATATACCAAATCTTATAAAATT
PfGA01_       GAATAAAAAGAAAATAAAAAAGGGACATGTAAAAAGCGTTTTGATAGATAATGTAAAGAAAGATAGGGATATACCAAATCTTATAAAATT
PfHB3_        GAATAAAAAGAAAATAAAAAAGGGACATGTAAAAAGCGTTTTGATAGATAATGTAAAGAAAGATAGGGATATACCAAATCTTATAAAATT
PfIT_         GAATAAAAACAAAATAAAAAAGGGACATGTAAAAAGCGTTTTGATAGATAATGTAAAGAAAGATAGGGATATACCAAATCTTATAAAATT
```

```
                        2890      2900      2910      2920      2930      2940      2950      2960      2970
               ....|....|....|....|....|....|....|....|....|....|....|....|....|....|....|....|....|....|
PF3D7 CDS      ACATTTGGATGCAATAGAAGAATGTAGTCATGATGAATGGGAAGAATATAAAATCGAATTTTTAGAAATATGTATTAAAGAATTTTTTAA
MS822 mRNA     ------------------------------------------------------------------------------------------
FCR3 mRNA      ACATTTGGATGCAATAGAAGAATGTAGTCATGATGAATGGGAAGAATATAAAATCGAATTTTTAGAAATATGTATTAAAGAATTTTTTAA
AM1898         ACATTTGGATGCAATAGAAGAATGTAGTCATGATGAATGGGAAGAATATAAAATCGAATTTTTAGAAATATGTATTAAAGAATTTTTTAA
AA235          ACATTTGGATGCAATAGAAGAATGTAGTCATGATGAATGGGAAGAATATAAAATCGAATTTTTAGAAATATGTATTAAAGAATTTTTTAA
AA243          ACATTTGGATGCAATAGAAGAATGTAGTCATGATGAATGGGAAGAATATAAAATCGAATTTTTAGAAATATGTATTAAAGAATTTTTTAA
AA258          ACATTTGGATGCAATAGAAGAATGTAGTCATGATGAATGGGAAGAATATAAAATCGAATTTTTAGAAATATGTATTAAAGAATTTTTTAA
AA264          ACATTTGGATGCAATAGAAGAATGTAGTCATGATGAATGGGAAGAATATAAAATCGAATTTTTAGAAATATGTATTAAAGAATTTTTTAA
AM1737         ACATTTGGATGCAATAGAAGAATGTAGTCATGATGAATGGGAAGAATATAAAATCGAATTTTTAGAAATATGTATTAAAGAATTTTTTAA
SC74           ACATTTGGATGCAATAGAAGAATGTAGTCATGATGAATGGGAAGAATATAAAATCGAATTTTTAGAAATATGTATTAAAGAATTTTTTAA
TAB166         ACATTTGGATGCAATAGAAGAATGTAGTCATGATGAATGGGAAGAATATAAAATCGAATTTTTAGAAATATGTATTAAAGAATTTTTTAA
TAB138         ACATTTGGATGCAATAGAAGAATGTAGTCATGATGAATGGGAAGAATATAAAATCGAATTTTTAGAAATATGTATTAAAGAATTTTTTAA
TAB130         ACATTTGGATGCAATAGAAGAATGTAGTCATGATGAATGGGAAGAATATAAAATCGAATTTTTAGAAATATGTATTAAAGAATTTTTTAA
TAB131         ACATTTGGATGCAATAGAAGAATGTAGTCATGATGAATGGGAAGAATATAAAATCGAATTTTTAGAAATATGTATTAAAGAATTTTTTAA
TAB151         ACATTTGGATGCAATAGAAGAATGTAGTCATGATGAATGGGAAGAATATAAAATCGAATTTTTAGAAATATGTATTAAAGAATTTTTTAA
TAB152         ACATTTGGATGCAATAGAAGAATGTAGTCATGATGAATGGGAAGAATATAAAATCGAATTTTTAGAAATATGTATTAAAGAATTTTTTAA
SC52           ACATTTGCATGCAATAGAAGAATGTAGTCATGATGAATGGGAAGAATATAAAATCGAATTTTTAGAAATATGTATTAAAGAATTTTTTAA
SC55           ACATTTGGATGCAATAGAAGAATGTAGTCATGATGAATGGGAAGAATATAAAATCGAATTTTTAGAAATATGTATTAAAGAATTTTTTAA
SC03           ACATTTGGATGCAATAGAAGAATGTAGTCATGATGAATGGGAAGAATATAAAATCGAATTTTTAGAAATATGTATTAAAGAATTTTTTAA
SC53           ACATTTGGATGCAATAGAAGAATGTAGTCATGATGAATGGGAAGAATATAAAATCGAATTTTTAGAAATATGTATTAAAGAATTTTTTAA
TAB141         ACATTTGGATGCAATAGAAGAATGTAGTCATGATGAATGGGAAGAATATAAAATCGAATTTTTAGAAATATGTATTAAAGAATTTTTTAA
TAB123         ACATTTGGATGCAATAGAAGAATGTAGTCATGATGAATGGGAAGAATATAAAATCGAATTTTTAGAAATATGTATTAAAGAATTTTTTAA
AM1803         ACATTTGGATGCAATAGAAGAATGTAGTCATGATGAATGGGAAGAATATAAAATCGAATTTTTAGAAATATGTATTAAAGAATTTTTTAA
AM1814         ACATTTGGATGCAATAGAAGAATGTAGTCATGATGAATGGGAAGAATATAAAATCGAATTTTTAGAAATATGTATTAAAGAATTTTTTAA
AM1802         ACATTTGGATGCAATAGAAGAATGTAGTCATGATGAATGGGAAGAATATAAAATCGAATTTTTAGAAATATGTATTAAAGAATTTTTTAA
AM1811         ACATTTGGATGCAATAGAAGAATGTAGTCATGATGAATGGGAAGAATATAAAATCGAATTTTTAGAAATATGTATTAAAGAATTTTTTAA
SC50           ACATTTGGATGCAATAGAAGAATGTAGTCATGATGAATGGGAAGAATATAAAATCGAATTTTTAGAAATATGTATTAAAGAATTTTTTAA
SC56           ACATTTGGATGCAATAGAAGAATGTAGTCATGATGAATGGGAAGAATATAAAATCGAATTTTTAGAAATATGTATTAAAGAATTTTTTAA
TAB153         ACATTTGGATGCAATAGAAGAATGTAGTCATGATGAATGGGAAGAATATAAAATCGAATTTTTAGAAATATGTATTAAAGAATTTTTTAA
TAB154         ACATTTGGATGCAATAGAAGAATGTAGTCATGATGAATGGGAAGAATATAAAATCGAATTTTTAGAAATATGTATTAAAGAATTTTTTAA
TAB156         ACATTTGGATGCAATAGAAGAATGTAGTCATGATGAATGGGAAGAATATAAAATCGAATTTTTAGAAATATGTATTAAAGAATTTTTTAA
TAB136         ACATTTGGATGCAATAGAAGAATGTAGTCATGATGAATGGGAAGAATATAAAATCGAATTTTTAGAAATATGTATTAAAGAATTTTTTAA
AM1880         ACATTTGGATGCAATAGAAGAATGTAGTCATGATGAATGGGAAGAATATAAAATCGAATTTTTAGAAATATGTATTAAAGAATTTTTTAA
PF3D7_         ACATTTGGATGCAATAGAAGAATGTAGTCATGATGAATGGGAAGAATATAAAATCGAATTTTTAGAAATATGTATTAAAGAATTTTTTAA
PfSD01         ACATTTGGATGCAATAGAAGAATGTAGTCATGATGAATGGGAAGAATATAAAATCGAATTTTTAGAAATATGTATTAAAGAATTTTTTAA
Pf7G8          ACATTTGGATGCAATAGAAGAATGTAGTCATGATGAATGGGAAGAATATAAAATCGAATTTTTAGAAATATGTATTAAAGAATTTTTTAA
FCR3 GENE      ACATTTGGATGCAATAGAAGAATGTAGTCATGATGAATGGGAAGAATATAAAATCGAATTTTTAGAAATATGTATTAAAGAATTTTTTAA
MS822 GENE     ------------------------------------------------------------------------------------------
PfCD01_        ACATTTGGATGCAATAGAAGAATGTAGTCATGATGAATGGGAAGAATATAAAATCGAATTTTTAGAAATATGTATTAAAGAATTTTTTAA
PfKH01__       ACATTTGGATGCAATAGAAGAATGTAGTCATGATGAATGGGAAGAATATAAAATCGAATTTTTAGAAATATGTATTAAAGAATTTTTTAA
PfDd2__        ACATTTGGATGCAATAGAAGAATGTAGTCATGATGAATGGGAAGAATATAAAATCGAATTTTTAGAAATATGTATTAAAGAATTTTTTAA
PfSN01_        ACATTTGGATGCAATAGAAGAATGTAGTCATGATGAATGGGAAGAATATAAAATCGAATTTTTAGAAATATGTATTAAAGAATTTTTTAA
PfGN01         ACATTTGGATGCAATAGAAGAATGTAGTCATGATGAATGGGAAGAATATAAAATCGAATTTTTAGAAATATGTATTAAAGAATTTTTTAA
PfKE01         ACATTTGGATGCAATAGAAGAATGTAGTCATGATGAATGGGAAGAATATAAAATCGAATTTTTAGAAATATGTATTAAAGAATTTTTTAA
PfKH02         ACATTTGGATGCAATAGAAGAATGTAGTCATGATGAATGGGAAGAATATAAAATCGAATTTTTAGAAATATGTATTAAAGAATTTTTTAA
PfGB4          ACATTTGGATGCAATAGAAGAATGTAGTCATGATGAATGGGAAGAATATAAAATCGAATTTTTAGAAATATGTATTAAAGAATTTTTTAA
PfGA01_        ACATTTGGATGCAATAGAAGAATGTAGTCATGATGAATGGGAAGAATATAAAATCGAATTTTTAGAAATATGTATTAAAGAATTTTTTAA
PfHB3_         ACATTTGGATGCAATAGAAGAATGTAGTCATTATGAATGGGAAGAATATAAAATCGAATTTTTAGAAATATGTATTAAAGAATTTTTTAA
PfIT_          ACATTTGGATGCAATAGAAGAATGTAGTCATGATGAATGGGAAGAATATAAAATCGAATTTTTAGAAATATGTATTAAAGAATTTTTTAA
```

```
                      2980      2990      3000      3010      3020      3030      3040      3050      3060
              ....|....|....|....|....|....|....|....|....|....|....|....|....|....|....|....|....|....|
PF3D7 CDS     AGAAAGGAAAATTGATGGACAAGGGAGAATGTTGGAAAAAAAGTATAAATGTGAAGATAATTTTTTAAATAATATTGATATATGGAAGAA
MS822 mRNA    ------------------------------------------------------------------------------------------
FCR3 mRNA     AGAAAGGAAAATTGATGGACAAGGGAGAATGTTGGAAAAAAAGTATAAATGTGAAGATAATTTTTTAAATAATATTGATATATGGAAGAA
AM1898        AGAAAGGAAAATTGATGGACAAGGGAGAATGTTGGAAAAAAAGTATAAATGTGAAGATAATTTTTTAAATAATGTTGATATATGGAAGAA
AA235         AGAAAGGAAAATTGATGGACAAGGGAGAATGTTGGAAAAAAAGTATAAATGTGAAGATAATTTTTTAAATAATATTGATATATGGAAGAA
AA243         AGAAAGGAAAATTGATGGACAAGGGAGAATGTTGGAAAAAAAGTATAAATGTGAAGATAATTTTTTAAATAATATTGATATATGGAAGAA
AA258         AGAAAGGAAAATTGATGGACAAGGGAGAATGTTGGAAAAAAAGTATAAATGTGAAGATAATTTTTTAAATAATATTGATATATGGAAGAA
AA264         AGAAAGGAAAATTGATGGACAAGGGAGAATGTTGGAAAAAAAGTATAAATGTGAAGATAATTTTTTAAATAATATTGATATATGGAAGAA
AM1737        AGAAAGGAAAATTGATGGACAAGGGAGAATGTTGGAAAAAAAGTATAAATGTGAAGATAATTTTTTAAATAATATTGATATATGGAAGAA
SC74          AGAAAGGAAAATTGATGGACAAGGGAGAATGTTGGAAAAAAAGTATAAATGTGAAGATAATTTTTTAAATAATATTGATATATGGAAGAA
TAB166        AGAAAGGAAAATTGATGGACAAGGGAGAATGTTGGAAAAAAAGTATAAATGTGAAGATAATTTTTTAAATAATATTGATATATGGAAGAA
TAB138        AGAAAGGAAAATTGATGGACAAGGGAGAATGTTGGAAAAAAAGTATAAATGTGAAGATAATTTTTTAAATAATATTGATATATGGAAGAA
TAB130        AGAAAGGAAAATTGATGGACAAGGGAGAATGTTGGAAAAAAAGTATAAATGTGAAGATAATTTTTTAAATAATATTGATATATGGAAGAA
TAB131        AGAAAGGAAAATTGATGGACAAGGGAGAATGTTGGAAAAAAAGTATAAATGTGAAGATAATTTTTTAAATAATATTGATATATGGAAGAA
TAB151        AGAAAGGAAAATTGATGGACAAGGGAGAATGTTGGAAAAAAAGTATAAATGTGAAGATAATTTTTTAAATAATATTGATATATGGAAGAA
TAB152        AGAAAGGAAAATTGATGGACAAGGGAGAATGTTGGAAAAAAAGTATAAATGTGAAGATAATTTTTTAAATAATATTGATATATGGAAGAA
SC52          AGAAAGGAAAATTGATGGACAAGGGAGAATGTTGGAAAAAAAGTATAAATGTGAAGATAATTTTTTAAATAATATTGATATATGGAAGAA
SC55          AGAAAGGAAAATTGATGGACAAGGGAGAATGTTGGAAAAAAAGTATAAATGTGAAGATAATTTTTTAAATAATATTGATATATGGAAGAA
SC03          AGAAAGGAAAATTGATGGACAAGGGAGAATGTTGGAAAAAAAGTATAAATGTGAAGATAATTTTTTAAATAATATTGATATATGGAAGAA
SC53          AGAAAGGAAAATTGATGGACAAGGGAGAATGTTGGAAAAAAAGTATAAATGTGAAGATAATTTTTTAAATAATATTGATATATGGAAGAA
TAB141        AGAAAGGAAAATTGATGGACAAGGGAGAATGTTGGAAAAAAAGTATAAATGTGAAGATAATTTTTTAAATAATATTGATATATGGAAGAA
TAB123        AGAAAGGAAAATTGATGGACAAGGGAGAATGTTGGAAAAAAAGTATAAATGTGAAGATAATTTTTTAAATAATATTGATATATGGAAGAA
AM1803        AGAAAGGAAAATTGATGGACAAGGGAGAATGTTGGAAAAAAAGTATAAATGTGAAGATAATTTTTTAAATAATATTGATATATGGAAGAA
AM1814        AGAAAGGAAAATTGATGGACAAGGGAGAATGTTGGAAAAAAAGTATAAATGTGAAGATAATTTTTTAAATAATATTGATATATGGAAGAA
AM1802        AGAAAGGAAAATTGATGGACAAGGGAAAATATTGGAAAAACAGTATAAATGTGAAGATAATTTTTTGAATAATATTGATATATGGAAGAA
AM1811        AGAAAGGAAAATTGATGGACAAGGGAGAATGTTGGAAAAAAAGTATAAATGTGAAGATAATTTTTTAAATAATATTGATATATGGAAGAA
SC50          AGAAAGGAAAATTGATGGACAAGGGAAAATATTGGAAAAACAGTATAAATGTGAAGATAATTTTTTGAATAATATTGATATATGGAAGAA
SC56          AGAAAGGAAAATTGATGGACAAGGGAGAATGTTGGAAAAAAAGTATAAATGTGAAGATAATTTTTTAAATAATATTGATATATGGAAGAA
TAB153        AGAAAGGAAAATTGATGGACAAGGGAGAATGTTGGAAAAAAAGTATAAATGTGAAGATAATTTTTTAAATAATATTGATATATGGAAGAA
TAB154        AGAAAGGAAAATTGATGGACAAGGGAGAATGTTGGAAAAAAAGTATAAATGTGAAGATAATTTTTTAAATAATATTGATATATGGAAGAA
TAB156        AGAAAGGAAAATTGATGGACAAGGGAAAATATTGGAAAAACAGTATAAATGTGAAGATAATTTTTTGAATAATATTGATATATGGAAGAA
TAB136        AGAAAGGAAAATTGATGGACAAGGGAGAATGTTGGAAAAAAAGTATAAATGTGAAGATAATTTTTTAAATAATATTGATATATGGAAGAA
AM1880        AGAAAGGAAAATTGATGGACAAGGGAGAATGTTGGAAAAAAAGTATAAATGTGAAGATAATTTTTTAAATAATATTGATATATGGAAGAA
PF3D7_        AGAAAGGAAAATTGATGGACAAGGGAGAATGTTGGAAAAAAAGTATAAATGTGAAGATAATTTTTTAAATAATATTGATATATGGAAGAA
PfSD01        AGAAAGGAAAATTGATGGACAAGGGAGAATGTTGGAAAAAAAGTATAAATGTGAAGATAATTTTTTAAATAATATTGATATATGGAAGAA
Pf7G8         AGAAAGGAAAATTGATGGACAAGGGAGAATGTTGGAAAAAAAGTATAAATGTGAAGATAATTTTTTAAATAATATTGATATATGGAAGAA
FCR3 GENE     AGAAAGGAAAATTGATGGACAAGGGAGAATGTTGGAAAAAAAGTATAAATGTGAAGATAATTTTTTAAATAATATTGATATATGGAAGAA
MS822 GENE    ------------------------------------------------------------------------------------------
PfCD01_       AGAAAGGAAAATTGATGGACAAGGGAGAATGTTGGAAAAAAAGTATAAATGTGAAGATAATTTTTTAAATAATATTGATATATGGAAGAA
PfKH01__      AGAAAGGAAAATTGATGGACAAGGGAGAATGTTGGAAAAAAAGTATAAATGTGAAGATAATTTTTTAAATAATATTGATATATGGAAGAA
PfDd2__       AGAAAGGAAAATTGATGGACAAGGGAGAATGTTGGAAAAAAAGTATAAATGTGAAGATAATTTTTTAAATAATATTGATATATGGAAGAA
PfSN01_       AGAAAGGAAAATTGATGGACAAGGGAGAATGTTGGAAAAAAAGTATAAATGTGAAGATAATTTTTTAAATAATATTGATATATGGAAGAA
PfGN01        AGAAAGGAAAATTGATGGACAAGGGAGAATGTTGGAAAAAAAGTATAAATGTGAAGATAATTTTTTAAATAATATTGATATATGGAAGAA
PfKE01        AGAAAGGAAAATTGATGGACAAGGGAGAATGTTGGAAAAAAAGTATAAATGTGAAGATAATTTTTTAAATAATATTGATATATGGAAGAA
PfKH02        AGAAAGGAAAATTGATGGACAAGGGAGAATGTTGGAAAAAAAGTATAAATGTGAAGATAATTTTTTAAATAATATTGATATATGGAAGAA
PfGB4         AGAAAGGAAAATTGATGGACAAGGGAGAATGTTGGAAAAAAAGTATAAATGTGAAGATAATTTTTTAAATAATATTGATATATGGAAGAA
PfGA01_       AGAAAGGAAAATTGATGGACAAGGGAGAATGTTGGAAAAAAAGTATAAATGTGAAGATAATTTTTTAAATAATATTGATATATGGAAGAA
PfHB3_        AGAAAGGAAAATTGATGGACAAGGGAGAATGTTGGAAAAAAAGTATAAATGTGAAGATAATTTTTTAAATAATATTGATATATGGAAGAA
PfIT_         AGAAAGGAAAATTGATGGACAAGGGAGAATGTTGGAAAAAAAGTATAAATGTGAAGATAATTTTTTAAATAATATTGATATATGGAAGAA
```

```
                3070      3080      3090      3100      3110      3120      3130      3140      3150
            ....|....|....|....|....|....|....|....|....|....|....|....|....|....|....|....|....|....|
PF3D7 CDS   AAAGAAATTAATGTGGAATAAATGGATTGAAAAAAAATAGATATATTATGAATAATTGGAAAGATGAAGAATGGTTTGATAATATAAAATG
MS822 mRNA  ------------------------------------------------------------------------------------------
FCR3 mRNA   AAAGAAATTAATGTGGAATAAATGGATTGAAAAAAAATAGATATATTATGAATAATTGGAAAGATGAAGAATGGTTTGATAATATAAAATG
AM1898      AAAGAAATTAATGTGGAATAAATGGATTGAAAAAAAATAGATATATTATGAATAATTGGAAAGATGAAGAATGGTTTGATAATATAAAATG
AA235       AAAGAAATTAATGTGGAATAAATGGATTGAAAAAAAATAGATATATTATGAATAATTGGAAAGATGAAGAATGGTTTGATAATATAAAATG
AA243       AAAGAAATTAATGTGGAATAAATGGATTGAAAAAAAATAGATATATTATGAATAATTGGAAAGATGAAGAATGGTTTGATAATATAAAATG
AA258       AAAGAAATTAATGTGGAATAAATGGATTGAAAAAAAATAGATATATTATGAATAATTGGAAAGATGAAGAATGGTTTGATAATATAAAATG
AA264       AAAGAAATTAATGTGGAATAAATGGATTGAAAAAAAATAGATATATTATGAATAATTGGAAAGATGAAGAATGGTTTGATAATATAAAATG
AM1737      AAAGAAATTAATGTGGAATAAATGGATTGAAAAAAAATAGATATATTATGAATAATTGGAAAGATGAAGAATGGTTTGATAATATAAAATG
SC74        AAAGAAATTAATGTGGAATAAATGGATTGAAAAAAAATAGATATATTATGAATAATTGGAAAGATGAAGAATGGTTTGATAATATAAAATG
TAB166      AAAGAAATTAATGTGGAATAAATGGATTGAAAAAAAATAGATATATTATGAATAATTGGAAAGATGAAGAATGGTTTGATAATATAAAATG
TAB138      AAAGAAATTAATGTGGAATAAATGGATTGAAAAAAAATAGATATATTATGAATAATTGGAAAGATGAAGAATGGTTTGATAATATAAAATG
TAB130      AAAGAAATTAATGTGGAATAAATGGATTGAAAAAAAATAGATATATTATGAATAATTGGAAAGATGAAGAATGGTTTGATAATATAAAATG
TAB131      AAAGAAATTAATGTGGAATAAATGGATTGAAAAAAAATAGATATATTATGAATAATTGGAAAGATGAAGAATGGTTTGATAATATAAAATG
TAB151      AAAGAAATTAATGTGGAATAAATGGATTGAAAAAAAATAGATATATTATGAATAATTGGAAAGATGAAGAATGGTTTGATAATATAAAATG
TAB152      AAAGAAATTAATGTGGAATAAATGGATTGAAAAAAAATAGATATATTATGAATAATTGGAAAGATGAAGAATGGTTTGATAATATAAAATG
SC52        AAAGAAATTAATGTGGAATAAATGGATTGAAAAAAAATAGATATATTATGAATAATTGGAAAGATGAAGAATGGTTTGATAATATAAAATG
SC55        AAAGAAATTAATGTGGAATAAATGGATTGAAAAAAAATAGATATATTATGAATAATTGGAAAGATGAAGAATGGTTTGATAATATAAAATG
SC03        AAAGAAATTAATGTGGAATAAATGGATTGAAAAAAAATAGATATATTATGAATAATTGGAAAGATGAAGAATGGTTTGATAATATAAAATG
SC53        AAAGAAATTAATGTGGAATAAATGGATTGAAAAAAAATAGATATATTATGAATAATTGGAAAGATGAAGAATGGTTTGATAATATAAAATG
TAB141      AAAGAAATTAATGTGGAATAAATGGATTGAAAAAAAATAGATATATTATGAATAATTGGAAAGATGAAGAATGGTTTGATAATATAAAATG
TAB123      AAAGAAATTAATGTGGAATAAATGGATTGAAAAAAAATAGATATATTATGAATAATTGGAAAGATGAAGAATGGTTTGATAATATAAAATG
AM1803      AAAGAAATTAATGTGGAATAAATGGATTGAAAAAAAATAGATATATTATGAATAATTGGAAAGATGAAGAATGGTTTGATAATATAAAATG
AM1814      AAAGAAATTAATGTGGAATAAATGGATTGAAAAAAAATAGATATATTATGAATAATTGGAAAGATGAAGAATGGTTTGATAATATAAAATG
AM1802      AAAGAAATTAATGTGGAATAAATGGATTGAAAGAAATAGATATATTATGAATAATTGGAAAGATGAAGAATGGTTTGATAATATAAAATG
AM1811      AAAGAAATTAATGTGGAATAAATGGATTGAAAAAAAATAGATATATTATGAATAATTGGAAAGATGAAGAATGGTTTGATAATATAAAATG
SC50        AAAGAAATTAATGTGGAATAAATGGATTGAAAGAAATAGATATATTATGAATAATTGGAAAGATGAAGAATGGTTTGATAATATAAAATG
SC56        AAAGAAATTAATGTGGAATAAATGGATTGAAAAAAAATAGATATATTATGAATAATTGGAAAGATGAAGAATGGTTTGATAATATAAAATG
TAB153      AAAGAAATTAATGTGGAATAAATGGATTGAAAAAAAATAGATATATTATGAATAATTGGAAAGATGAAGAATGGTTTGATAATATAAAATG
TAB154      AAAGAAATTAATGTGGAATAAATGGATTGAAAAAAAATAGATATATTATGAATAATTGGAAAGATGAAGAATGGTTTGATAATATAAAATG
TAB156      AAAGAAATTAATGTGGAATAAATGGATTGAAAGAAATAGATATATTATGAATAATTGGAAAGATGAAGAATGGTTTGATAATATAAAATG
TAB136      AAAGAAATTAATGTGGAATAAATGGATTGAAAAAAAATAGATATATTATGAATAATTGGAAAGATGAAGAATGGTTTGATAATATAAAATG
AM1880      AAAGAAATTAATGTGGAATAAATGGATTGAAAAAAAATAGATATATTATGAATAATTGGAAAGATGAAGAATGGTTTGATAATATAAAATG
PF3D7_      AAAGAAATTAATGTGGAATAAATGGATTGAAAAAAAATAGATATATTATGAATAATTGGAAAGATGAAGAATGGTTTGATAATATAAAATG
PfSD01      AAAGAAATTAATGTGGAATAAATGGATTGAAAAAAAATAGATATATTATGAATAATTGGAAAGATGAAGAATGGTTTGATAATATAAAATG
Pf7G8       AAAGAAATTAATGTGGAATAAATGGATTGAAAAAAAATAGATATATTATGAATAATTGGAAAGATGAAGAATGGTTTGATAATATAAAATG
FCR3 GENE   AAAGAAATTAATGTGGAATAAATGGATTGAAAAAAAATAGATATATTATGAATAATTGGAAAGATGAAGAATGGTTTGATAATATAAAATG
MS822 GENE  ------------------------------------------------------------------------------------------
PfCD01_     AAAGAAATTAATGTGGAATAAATGGATTGAAAAAAAATAGATATATTATGAATAATTGGAAAGATGAAGAATGGTTTGATAATATAAAATG
PfKH01__    AAAGAAATTAATGTGGAATAAATGGATTGAAAAAAAATAGATATATTATGAATAATTGGAAAGATGAAGAATGGTTTGATAATATAAAATG
PfDd2__     AAAGAAATTAATGTGGAATAAATGGATTGAAAAAAAATAGATATATTATGAATAATTGGAAAGATGAAGAATGGTTTGATAATATAAAATG
PfSN01_     AAAGAAATTAATGTGGAATAAATGGATTGAAAAAAAATAGATATATTATGAATAATTGGAAAGATGAAGAATGGTTTGATAATATAAAATG
PfGN01      AAAGAAATTAATGTGGAATAAATGGATTGAAAAAAAATAGATATATTATGAATAATTGGAAAGATGAAGAATGGTTTGATAATATAAAATG
PfKE01      AAAGAAATTAATGTGGAATAAATGGATTGAAAAAAAATAGATATATTATGAATAATTGGAAAGATGAAGAATGGTTTGATAATATAAAATG
PfKH02      AAAGAAATTAATGTGGAATAAATGGATTGAAAAAAAATAGATATATTATGAATAATTGGAAAGATGAAGAATGGTTTGATAATATAAAATG
PfGB4       AAAGAAATTAATGTGGAATAAATGGATTGAAAAAAAATAGATATATTATGAATAATTGGAAAGATGAAGAATGGTTTGATAATATAAAATG
PfGA01_     AAAGAAATTAATGTGGAATAAATGGATTGAAAAAAAATAGATATATTATGAATAATTGGAAAGATGAAGAATGGTTTGATAATATAAAATG
PfHB3_      AAAGAAATTAATGTGGAATAAATGGATTGAAAAAAAATAGATATATTATGAATAATTGGAAAGATGAAGAATGGTTTGATAATATAAAATG
PfIT_       AAAGAAATTAATGTGGAATAAATGGATTGAAAAAAAATAGATATATTATGAATAATTGGAAAGATGAAGAATGGTTTGATAATATAAAATG
```

```
                   3160      3170      3180      3190      3200      3210      3220      3230      3240
               ....|....|....|....|....|....|....|....|....|....|....|....|....|....|....|....|....|....|
PF3D7 CDS      TGAATGGGAGAATGAAATAGAGGAATATTTTAAATTAAAAAAATATGATAAAAATTTATTAGAAATTTTTGGAAAAAATAAAGAAAATAA
MS822 mRNA     ------------------------------------------------------------------------------------------
FCR3 mRNA      TGAATGGGAGAATGAAATAGAGGAATATTTTAAATTAAAAAAATATGATGAAAATTTATTAGAAATTTTTGGAAAAAATAAAGAAAATCA
AM1898         TGAATGGGAGAATGAAATAGAGGAATATTTTAAATTAAAAAAATATGATAAAAATTTATTAGAAATTTTTGGAAAAAATAAAGAAAATAA
AA235          TGAATGGGAGAATGAAATAGAGGAATATTTTAAATTAAAAAAATATGATAAAAATTTATTAGAAATTTTTGGAAAAAATAAAGAAAATAA
AA243          TGAATGGGAGAATGAAATAGAGGAATATTTTAAATTAAAAAAATATGATAAAAATTTATTAGAAATTTTTGGAAAAAATAAAGAAAATAA
AA258          TGAATGGGAGAATGAAATAGAGGAATATTTTAAATTAAAAAAATATGATAAAAATTTATTAGAAATTTTTGGAAAAAATAAAGAAAATAA
AA264          TGAATGGGAGAATGAAATAGAGGAATATTTTAAATTAAAAAAATATGATAAAAATTTATTAGAAATTTTTGGAAAAAATAAAGAAAATAA
AM1737         TGAATGGGAGAATGAAATAGAGGAATATTTTAAATTAAAAAAATATGATAAAAATTTATTAGAAATTTTTGGAAAAAATAAAGAAAATAA
SC74           TGAATGGGAGAATGAAATAGAGGAATATTTTAAATTAAAAAAATATGATAAAAATTTATTAGAAATTTTTGGAAAAAATAAAGAAAATAA
TAB166         TGAATGGGAGAATGAAATAGAGGAATATTTTAAATTAAAAAAATATGATAAAAATTTATTAGAAATTTTTGGAAAAAATAAAGAAAATAA
TAB138         TGAATGGGAGAATGAAATAGAGGAATATTTTAAATTAAAAAAATATGATAAAAATTTATTAGAAATTTTTGGAAAAAATAAAGAAAATAA
TAB130         TGAATGGGAGAATGAAATAGAGGAATATTTTAAATTAAAAAAATATGATAAAAATTTATTAGAAATTTTTGGAAAAAATAAAGAAAATAA
TAB131         TGAATGGGAGAATGAAATAGAGGAATATTTTAAATTAAAAAAATATGATAAAAATTTATTAGAAATTTTTGGAAAAAATAAAGAAAATAA
TAB151         TGAATGGGAGAATGAAATAGAGGAATATTTTAAATTAAAAAAATATGATAAAAATTTATTAGAAATTTTTGGAAAAAATAAAGAAAATAA
TAB152         TGAATGGGAGAATGAAATAGAGGAATATTTTAAATTAAAAAAATATGATAAAAATTTATTAGAAATTTTTGGAAAAAATAAAGAAAATAA
SC52           TGAATGGGAGAATGAAATAGAGGAATATTTTAAATTAAAAAAATATGATAAAAATTTATTAGAAATTTTTGAAAAAAATCGAGAAAATAA
SC55           TGAATGGGAGAATGAAATAGAGGAATATTTTAAATTAAAAAAATATGATAAAAATTTATTAGAAATTTTTGGAAAAAATAAAGAAAATAA
SC03           TGAATGGGAGAATGAAATAGAGGAATATTTTAAATTAAAAAAATATGATAAAAATTTATTAGAAATTTTTGGAAAAAATAAAGAAAATAA
SC53           TGAATGGGAGAATGAAATAGAGGAATATTTTAAATTAAAAAAATATGATAAAAATTTATTAGAAATTTTTGGAAAAAATAAAGAAAATAA
TAB141         TGAATGGGAGAATGAAATAGAGGAATATTTTAAATTAAAAAAATATGATAAAAATTTATTAGAAATTTTTGGAAAAAATAAAGAAAATAA
TAB123         TGAATGGGAGAATGAAATAGAGGAATATTTTAAATTAAAAAAATATGATAAAAATTTATTAGAAATTTTTGGAAAAAATAAAGAAAATAA
AM1803         TGAATGGGAGAATGAAATAGAGGAATATTTTAAATTAAAAAAATATGATAAAAATTTATTAGAAATTTTTGGAAAAAATAAAGAAAATAA
AM1814         TGAATGGGAGAATGAAATAGAGGAATATTTTAAATTAAAAAAATATGATAAAAATTTATTAGAAATTTTTGGAAAAAATAAAGAAAATAA
AM1802         TGAATGGGAGAATGAAATAGAGGAATATTTTAAATTAAAAAAATATGATAAAAATTTATTAGAAATTTTTGAAAAAAATCGAGAAAATAA
AM1811         TGAATGGGAGAATGAAATAGAGGAATATTTTAAATTAAAAAAATATGATAAAAATTTATTAGAAATTTTTGGAAAAAATAAAGAAAATAA
SC50           TGAATGGGAGAATGAAATAGAGGAATATTTTAAATTAAAAAAATATGATAAAAATTTATTAGAAATTTTTGAAAAAAATCGAGAAAATAA
SC56           TGAATGGGAGAATGAAATAGAGGAATATTTTAAATTAAAAAAATATGATAAAAATTTATTAGAAATTTTTGGAAAAAATAAAGAAAATAA
TAB153         TGAATGGGAGAATGAAATAGAGGAATATTTTAAATTAAAAAAATATGATAAAAATTTATTAGAAATTTTTGGAAAAAATAAAGAAAATAA
TAB154         TGAATGGGAGAATGAAATAGAGGAATATTTTAAATTAAAAAAATATGATAAAAATTTATTAGAAATTTTTGGAAAAAATAAAGAAAATAA
TAB156         TGAATGGGAGAATGAAATAGAGGAATATTTTAAATTAAAAAAATATGATAAAAATTTATTAGAAATTTTTGAAAAAAATCGAGAAAATAA
TAB136         TGAATGGGAGAATGAAATAGAGGAATATTTTAAATTAAAAAAATATGATAAAAATTTATTAGAAATTTTTGGAAAAAATAAAGAAAATAA
AM1880         TGAATGGGAGAATGAAATAGAGGAATATTTTAAATTAAAAAAATATGATAAAAATTTATTAGAAATTTTTGGAAAAAATAAAGAAAATAA
PF3D7_         TGAATGGGAGAATGAAATAGAGGAATATTTTAAATTAAAAAAATATGATAAAAATTTATTAGAAATTTTTGGAAAAAATAAAGAAAATAA
PfSD01         TGAATGGGAGAATGAAATAGAGGAATATTTTAAATTAAAAAAATATGATAAAAATTTATTAGAAATTTTTGGAAAAAATAAAGAAAATAA
Pf7G8          TGAATGGGAGAATGAAATAGAGGAATATTTTAAATTAAAAAAATATGATAAAAATTTATTAGAAATTTTTGAAAAAAATCGAGAAAATAA
FCR3 GENE      TGAATGGGAGAATGAAATAGAGGAATATTTTAAATTAAAAAAATATGATGAAAATTTATTAGAAATTTTTGGAAAAAATAAAGAAAATCA
MS822 GENE     ------------------------------------------------------------------------------------------
PfCD01_        TGAATGGGAGAATGAAATAGAGGAATATTTTAAATTAAAAAAATATGATAAAAATTTATTAGAAATTTTTGAAAAAAATCGAGAAAATAA
PfKH01__       TGAATGGGAGAATGAAATAGAGGAATATTTTAAATTAAAAAAATATGATAAAAATTTATTAGAAATTTTTGGAAAAAATAAAGAAAATAA
PfDd2__        TGAATGGGAGAATGAAATAGAGGAATATTTTAAATTAAAAAAATATGATAAAAATTTATTAGAAATTTTTGGAAAAAATAAAGAAAATAA
PfSN01_        TGAATGGGAGAATGAAATAGAGGAATATTTTAAATTAAAAAAATATGATAAAAATTTATTAGAAATTTTTGGAAAAAATAAAGAAAATAA
PfGN01         TGAATGGGAGAATGAAATAGAGGAATATTTTAAATTAAAAAAATATGATAAAAATTTATTAGAAATTTTTGGAAAAAATAAAGAAAATAA
PfKE01         TGAATGGGAGAATGAAATAGAGGAATATTTTAAATTAAAAAAATATGATAAAAATTTATTAGAAATTTTTGGAAAAAATAAAGAAAATAA
PfKH02         TGAATGGGAGAATGAAATAGAGGAATATTTTAAATTAAAAAAATATGATAAAAATTTATTAGAAATTTTTGGAAAAAATAAAGAAAATAA
PfGB4          TGAATGGGAGAATGAAATAGAGGAATATTTTAAATTAAAAAAATATGATAAAAATTTATTAGAAATTTTTGGAAAAAATAAAGAAAATAA
PfGA01_        TGAATGGGAGAATGAAATAGAGGAATATTTTAAATTAAAAAAATATGATAAAAATTTATTAGAAATTTTTGGAAAAAATAAAGAAAATAA
PfHB3_         TGAATGGGAGAATGAAATAGAGGAATATTTTAAATTAAAAAAATATGATAAAAATTTATTAGAAATTTTTGGAAAAAATAAAGAAAATAA
PfIT_          TGAATGGGAGAATGAAATAGAGGAATATTTTAAATTAAAAAAATATGATAAAAATTTATTAGAAATTTTTGGAAAAAATAAAGAAAATAA
```

```
                      3250      3260      3270      3280      3290      3300      3310      3320      3330
            ....|....|....|....|....|....|....|....|....|....|....|....|....|....|....|....|....|....|
PF3D7 CDS   CAAAAGTTTTATGATAGAAATATTAAAAATTATATGGAAAAAATGGATAGAAAAAAATGTAAAATATATAAATGAATCTGTTACAAATGA
MS822 mRNA  ------------------------------------------------------------------------------------------
FCR3 mRNA   CAAAAGTTTTATGATAGAAATATTAAAAATTATATGGAAAAAATGGATAGAAAAAAATGTAAAATATATAAATAAATCTGTTACAAATGA
AM1898      CAAAAGTTTTATGATAGAAATATTAAAAATTATATGGAAAAAATGGATAGAAAAAAATGTAAAATATATAAATGAATCTGTTACAAATGA
AA235       CAAAAGTTTTATGATAGAAATATTAAAAATTATATGGAAAAAATGGATAGAAAAAAATGTAAAATATATAAATGAATCTGTTACAAATGA
AA243       CAAAAGTTTTATGATAGAAATATTAAAAATTATATGGAAAAAATGGATAGAAAAAAATGTAAAATATATAAATAAATCTGTTACAAATGA
AA258       CAAAAGTTTTATGATAGAAATATTAAAAATTATATGGAAAAAATGGATAGAAAAAAATGTAAAATATATAAATGAATCTGTTACAAATGA
AA264       CAAAAGTTTTATGATAGAAATATTAAAAATTATATGGAAAAAATGGATAGAAAAAAATGTAAAATATATAAATGAATCTGTTACAAATGA
AM1737      CAAAAGTTTTATGATAGAAATATTAAAAATTATATGGAAAAAATGGATAGAAAAAAATGTAAAATATATAAATAAATCTGTTACAAATGA
SC74        CAAAAGTTTTATGATAGAAATATTAAAAATTATATGGAAAAAATGGATAGAAAAAAATGTAAAATATATAAATGAATCTGTTACAAATGA
TAB166      CAAAAGTTTTATGATAGAAATATTAAAAATTATATGGAAAAAATGGATAGAAAAAAATGTAAAATATATAAATAAATCTGTTACAAATGA
TAB138      CAAAAGTTTTATGATAGAAATATTAAAAATTATATGGAAAAAATGGATAGAAAAAAATGTAAAATATATAAATAAATCTGTTACAAATGA
TAB130      CAAAAGTTTTATGATAGAAATATTAAAAATTATATGGAAAAAATGGATAGAAAAAAATGTAAAATATATAAATAAATCTGTTACAAATGA
TAB131      CAAAAGTTTTATGATAGAAATATTAAAAATTATATGGAAAAAATGGATAGAAAAAAATGTAAAATATATAAATGAATCTGTTACAAATGA
TAB151      CAAAAGTTTTATGATAGAAATATTAAAAATTATATGGAAAAAATGGATAGAAAAAAATGTAAAATATATAAATGAATCTGTTACAAATGA
TAB152      CAAAAGTTTTATGATAGAAATATTAAAAATTATATGGAAAAAATGGATAGAAAAAAATGTAAAATATATAAATGAATCTGTTACAAATGA
SC52        CAAAAGTTTTATGATAGAAATATTAAAAATTATATGGAAAAAATGGATAGAAAAAAATGTAAAATATATAAATGAATCTGTTACAAATGA
SC55        CAAAAGTTTTATGATAGAAATATTAAAAATTATATGGAAAAAATGGATAGAAAAAAATGTAAAATATATAAATGAATCTGTTACAAATGA
SC03        CAAAAGTTTTATGATAGAAATATTAAAAATTATATGGAAAAAATGGATAGAAAAAAATGTAAAATATATAAATAAATCTGTTACAAATGA
SC53        CAAAAGTTTTATGATAGAAATATTAAAAATTATATGGAAAAAATGGATAGAAAAAAATGTAAAATATATAAATGAATCTGTTACAAATGA
TAB141      CAAAAGTTTTATGATAGAAATATTAAAAATTATATGGAAAAAATGGATAGAAAAAAATGTAAAATATATAAATAAATCTGTTACAAATGA
TAB123      CAAAAGTTTTATGATAGAAATATTAAAAATTATATGGAAAAAATGGATAGAAAAAAATGTAAAATATATAAATGAATCTGTTACAAATGA
AM1803      CAAAAGTTTTATGATAGAAATATTAAAAATTATATGGAAAAAATGGATAGAAAAAAATGTAAAATATATAAATAAATCTGTTACAAATGA
AM1814      CAAAAGTTTTATGATAGAAATATTAAAAATTATATGGAAAAAATGGATAGAAAAAAATGTAAAATATATAAATGAATCTGTTACAAATGA
AM1802      CAAAAGTTTTATGATAGAAATATTAAAAATTATATGGAAAAAATGGATAGAAAAAAATGTAAAATATATAAATGAATCTGTTACAAATGA
AM1811      CAAAAGTTTTATGATAGAAATATTAAAAATTATATGGAAAAAATGGATAGAAAAAAATGTAAAATATATAAATAAATCTGTTACAAATGA
SC50        CAAAAGTTTTATGATAGAAATATTAAAAATTATATGGAAAAAATGGATAGAAAAAAATGTAAAATATATAAATGAATCTGTTACAAATGA
SC56        CAAAAGTTTTATGATAGAAATATTAAAAATTATATGGAAAAAATGGATAGAAAAAAATGTAAAATATATAAATAAATCTGTTACAAATGA
TAB153      CAAAAGTTTTATGATAGAAATATTAAAAATTATATGGAAAAAATGGATAGAAAAAAATGTAAAATATATAAATGAATCTGTTACAAATGA
TAB154      CAAAAGTTTTATGATAGAAATATTAAAAATTATATGGAAAAAATGGATAGAAAAAAATGTAAAATATATAAATGAATCTGTTACAAATGA
TAB156      CAAAAGTTTTATGATAGAAATATTAAAAATTATATGGAAAAAATGGATAGAAAAAAATGTAAAATATATAAATGAATCTGTTACAAATGA
TAB136      CAAAAGTTTTATGATAGAAATATTAAAAATTATATGGAAAAAATGGATAGAAAAAAATGTAAAATATATAAATGAATCTGTTACAAATGA
AM1880      CAAAAGTTTTATGATAGAAATATTAAAAATTATATGGAAAAAATGGATAGAAAAAAATGTAAAATATATAAATGAATCTGTTACAAATGA
PF3D7_      CAAAAGTTTTATGATAGAAATATTAAAAATTATATGGAAAAAATGGATAGAAAAAAATGTAAAATATATAAATGAATCTGTTACAAATGA
PfSD01      CAAAAGTTTTATGATAGAAATATTAAAAATTATATGGAAAAAATGGATAGAAAAAAATGTAAAATATATAAATGAATCTGTTACAAATGA
Pf7G8       CAAAAGTTTTATGATAGAAATATTAAAAATTATATGGAAAAAATGGATAGAAAAAAATGTAAAATATATAAATGAATCTGTTACAAATGA
FCR3 GENE   CAAAAGTTTTATGATAGAAATATTAAAAATTATATGGAAAAAATGGATAGAAAAAAATGTAAAATATATAAATAAATCTGTTACAAATGA
MS822 GENE  ------------------------------------------------------------------------------------------
PfCD01_     CAAAAGTTTTATGATAGAAATATTAAAAATTATATGGAAAAAATGGATAGAAAAAAATGTAAAATATATAAATGAATCTGTTACAAATGA
PfKH01__    CAAAAGTTTTATGATAGAAATATTAAAAATTATATGGAAAAAATGGATAGAAAAAAATGTAAAATATATAAATGAATCTGTTACAAATGA
PfDd2__     CAAAAGTTTTATGATAGAAATATTAAAAATTATATGGAAAAAATGGATAGAAAAAAATGTAAAATATATAAATAAATCTGTTACAAATGA
PfSN01_     CAAAAGTTTTATGATAGAAATATTAAAAATTATATGGAAAAAATGGATAGAAAAAAATGTAAAATATATAAATGAATCTGTTACAAATGA
PfGN01      CAAAAGTTTTATGATAGAAATATTAAAAATTATATGGAAAAAATGGATAGAAAAAAATGTAAAATATATAAATAAATCTGTTACAAATGA
PfKE01      CAAAAGTTTTATGATAGAAATATTAAAAATTATATGGAAAAAATGGATAGAAAAAAATGTAAAATATATAAATGAATCTGTTACAAATGA
PfKH02      CAAAAGTTTTATGATAGAAATATTAAAAATTATATGGAAAAAATGGATAGAAAAAAATGTAAAATATATAAATGAATCTGTTACAAATGA
PfGB4       CAAAAGTTTTATGATAGAAATATTAAAAATTATATGGAAAAAATGGATAGAAAAAAATGTAAAATATATAAATGAATCTGTTACAAATGA
PfGA01_     CAAAAGTTTTATGATAGAAATATTAAAAATTATATGGAAAAAATGGATAGAAAAAAATGTAAAATATATAAATGAATCTGTTACAAATGA
PfHB3_      CAAAAGTTTTATGATAGAAATATTAAAAATTATATGGAAAAAATGGATAGAAAAAAATGTAAAATATATAAATGAATCTGTTACAAATGA
PfIT_       CAAAAGTTTTATGATAGAAATATTAAAAATTATATGGAAAAAATGGATAGAAAAAAATGTAAAATATATAAATAAATCTGTTACAAATGA
```

```
                  3340      3350      3360      3370      3380      3390      3400      3410      3420
             ....|....|....|....|....|....|....|....|....|....|....|....|....|....|....|....|....|....|
PF3D7 CDS    GTGGATCAATAAATTGGTAATAAAATATAAAAATGATTATATAGATCTAAAGAAAGATTATACAAGGTTTTTAAAAGATATATATGATAT
MS822 mRNA   ------------------------------------------------------------------------------------------
FCR3 mRNA    GTGGATCAATCAATTGGTAATAAAATATAAAAATGATTATATAGATCTAAAGAAAGATTATACAAGGTTTTTAAAAGATATATATGATAT
AM1898       GTGGATCAATAAATTGGTAATAAAATATAAAAATGATTATATAGATCTAAAGAAAGATTATACAAGGTTTTTAAAAGATATATATGATAT
AA235        GTGGATCAATAAATTGGTAATAAAATATAAAAATGATTATATAGATCTAAAGAAAGATTATACAAGGTTTTTAAAAGATATATATGATAT
AA243        GTGGATCAATAAATTGGTAATAAAATATAAAAATGATTATATAGATCTAAAGAAAGATTATACAAGGTTTTTAAAAGATATATATGATAT
AA258        GTGGATCAATAAATTGGTAATAAAATATAAAAATGATTATATAGATCTAAAGAAAGATTATACAAGGTTTTTAAAAGATATATATGATAT
AA264        GTGGATCAATAAATTGGTAATAAAATATAAAAATGATTATATAGATCTAAAGAAAGATTATACAAGGTTTTTAAAAGATATATATGATAT
AM1737       GTGGATCAATAAATTGGTAATAAAATATAAAAATGATTATATAGATCTAAAGAAAGATTATACAAGGTTTTTAAAAGATATATATGATAT
SC74         GTGGATCAATAAATTGGTAATAAAATATAAAAATGATTATATAGATCTAAAGAAAGATTATACAAGGTTTTTAAAAGATATATATGATAT
TAB166       GTGGATCAATAAATTGGTAATAAAATATAAAAATGATTATATAGATCTAAAGAAAGATTATACAAGGTTTTTAAAAGATATATATGATAT
TAB138       GTGGATCAATAAATTGGTAATAAAATATAAAAATGATTATATAGATCTAAAGAAAGATTATACAAGGTTTTTAAAAGATATATATGATAT
TAB130       GTGGATCAATAAATTGGTAATAAAATATAAAAATGATTATATAGATCTAAAGAAAGATTATACAAGGTTTTTAAAAGATATATATGATAT
TAB131       GTGGATCAATAAATTGGTAATAAAATATAAAAATGATTATATAGATCTAAAGAAAGATTATACAAGGTTTTTAAAAGATATATATGATAT
TAB151       GTGGATCAATAAATTGGTAATAAAATATAAAAATGATTATATAGATCTAAAGAAAGATTATACAAGGTTTTTAAAAGATATATATGATAT
TAB152       GTGGATCAATAAATTGGTAATAAAATATAAAAATGATTATATAGATCTAAAGAAAGATTATACAAGGTTTTTAAAAGATATATATGATAT
SC52         GTGGATCAATAAATTGGTAATAAAATATAAAAATGATTATATAGATCTAAAGAAAGATTATACAAGGTTTTTAAAAGATATATATGATAT
SC55         GTGGATCAATAAATTGGTAATAAAATATAAAAATGATTATATAGATCTAAAGAAAGATTATACAAGGTTTTTAAAAGATATATATGATAT
SC03         GTGGATCAATAAATTGGTAATAAAATATAAAAATGATTATATAGATCTAAAGAAAGATTATACAAGGTTTTTAAAAGATATATATGATAT
SC53         GTGGATCAATAAATTGGTAATAAAATATAAAAATGATTATATAGATCTAAAGAAAGATTATACAAGGTTTTTAAAAGATATATATGATAT
TAB141       GTGGATCAATAAATTGGTAATAAAATATAAAAATGATTATATAGATCTAAAGAAAGATTATACAAGGTTTTTAAAAGATATATATGATAT
TAB123       GTGGATCAATAAATTGGTAATAAAATATAAAAATGATTATATAGATCTAAAGAAAGATTATACAAGGTTTTTAAAAGATATATATGATAT
AM1803       GTGGATCAATAAATTGGTAATAAAATATAAAAATGATTATATAGATCTAAAGAAAGATTATACAAGGTTTTTAAAAGATATATATGATAT
AM1814       GTGGATCAATAAATTGGTAATAAAATATAAAAATGATTATATAGATCTAAAGAAAGATTATACAAGGTTTTTAAAAGATATATATGATAT
AM1802       GTGGATCAATAAATTGGTAATAAAATATAAAAATGATTATATAGATCTAAAGAAAGATTATACAAGGTTTTTAAAAGATATATATGATAT
AM1811       GTGGATCAATAAATTGGTAATAAAATATAAAAATGATTATATAGATCTAAAGAAAGATTATACAAGGTTTTTAAAAGATATATATGATAT
SC50         GTGGATCAATAAATTGGTAATAAAATATAAAAATGATTATATAGATCTAAAGAAAGATTATACAAGGTTTTTAAAAGATATATATGATAT
SC56         GTGGATCAATAAATTGGTAATAAAATATAAAAATGATTATATAGATCTAAAGAAAGATTATACAAGGTTTTTAAAAGATATATATGATAT
TAB153       GTGGATCAATAAATTGGTAATAAAATATAAAAATGATTATATAGATCTAAAGAAAGATTATACAAGGTTTTTAAAAGATATATATGATAT
TAB154       GTGGATCAATAAATTGGTAATAAAATATAAAAATGATTATATAGATCTAAAGAAAGATTATACAAGGTTTTTAAAAGATATATATGATAT
TAB156       GTGGATCAATAAATTGGTAATAAAATATAAAAATGATTATATAGATCTAAAGAAAGATTATACAAGGTTTTTAAAAGATATATATGATAT
TAB136       GTGGATCAATAAATTGGTAATAAAATATAAAAATGATTATATAGATCTAAAGAAAGATTATACAAGGTTTTTAAAAGATATATATGATAT
AM1880       GTGGATCAATAAATTGGTAATAAAATATAAAAATGATTATATAGATCTAAAGAAAGATTATACAAGGTTTTTAAAAGATATATATGATAT
PF3D7_       GTGGATCAATAAATTGGTAATAAAATATAAAAATGATTATATAGATCTAAAGAAAGATTATACAAGGTTTTTAAAAGATATATATGATAT
PfSD01       GTGGATCAATAAATTGGTAATAAAATATAAAAATGATTATATAGATCTAAAGAAAGATTATACAAGGTTTTTAAAAGATATATATGATAT
Pf7G8        GTGGATCAATAAATTGGTAATAAAATATAAAAATGATTATATAGATCTAAAGAAAGATTATACAAGGTTTTTAAAAGATATATATGATAT
FCR3 GENE    GTGGATCAATCAATTGGTAATAAAATATAAAAATGATTATATAGATCTAAAGAAAGATTATACAAGGTTTTTAAAAGATATATATGATAT
MS822 GENE   ------------------------------------------------------------------------------------------
PfCD01_      GTGGATCAATAAATTGGTAATAAAATATAAAAATGATTATATAGATCTAAAGAAAGATTATACAAGGTTTTTAAAAGATATATATGATAT
PfKH01__     GTGGATCAATAAATTGGTAATAAAATATAAAAATGATTATATAGATCTAAAGAAAGATTATACAAGGTTTTTAAAAGATATATATGATAT
PfDd2__      GTGGATCAATAAATTGGTAATAAAATATAAAAATGATTATATAGATCTAAAGAAAGATTATACAAGGTTTTTAAAAGATATATATGATAT
PfSN01_      GTGGATCAATAAATTGGTAATAAAATATAAAAATGATTATATAGATCTAAAGAAAGATTATACAAGGTTTTTAAAAGATATATATGATAT
PfGN01       GTGGATCAATAAATTGGTAATAAAATATAAAAATGATTATATAGATCTAAAGAAAGATTATACAAGGTTTTTAAAAGATATATATGATAT
PfKE01       GTGGATCAATAAATTGGTAATAAAATATAAAAATGATTATATAGATCTAAAGAAAGATTATACAAGGTTTTTAAAAGATATATATGATAT
PfKH02       GTGGATCAATAAATTGGTAATAAAATATAAAAATGATTATATAGATCTAAAGAAAGATTATACAAGGTTTTTAAAAGATATATATGATAT
PfGB4        GTGGATCAATAAATTGGTAATAAAATATAAAAATGATTATATAGATCTAAAGAAAGATTATACAAGGTTTTTAAAAGATATATATGATAT
PfGA01_      GTGGATCAATAAATTGGTAATAAAATATAAAAATGATTATATAGATCTAAAGAAAGATTATACAAGGTTTTTAAAAGATATATATGATAT
PfHB3_       GTGGATCAATAAATTGGTAATAAAATATAAAAATGATTATATAGATCTAAAGAAAGATTATACAAGGTTTTTAAAAGATATATATGATAT
PfIT_        GTGGATCAATAAATTGGTAATAAAATATAAAAATGATTATATAGATCTAAAGAAAGATTATACAAGGTTTTTAAAAGATATATATGATAT
```

```
                   3430      3440      3450      3460      3470      3480      3490      3500      3510
          ....|....|....|....|....|....|....|....|....|....|....|....|....|....|....|....|....|....|
PF3D7 CDS   TCCATTGGATAATAATAATAATAAAAATATAGAATATTTAGACAATGACAATTTGATAAAAATTATGTTTATACAAATATATATGCTTGC
MS822 mRNA  ------------------------------------------------------------------------------------------
FCR3 mRNA   TCCATTGGATAATAATAATAATAAAAATATAGAATATTTAGACAATGGCAATTTGATAAAAATTATGTTTATACAAATATATATGCTTGC
AM1898      TCCATTGGATAATAATAATAATAAAAATATAGAATATTTAGACAATGGCAATTTGATAAAAATTATGTTTATACAAATATATATGCTTGC
AA235       TCCATTGGATAATAATAATAATAAAAATATAGAATATTTAGACAATGACAATTTGATAAAAATTATGTTTATACAAATATATATGCTTGC
AA243       TCCATTGGATAATAATAATAATAAAAATATAGAATATTTAGACAATGGCAATTTGATAAAAATTATGTTTATACAAATATATATGCTTGC
AA258       TCCATTGGATAATAATAATAATAAAAATATAGAATATTTAGACAATGACAATTTGATAAAAATTATGTTTATACAAATATATATGCTTGC
AA264       TCCATTGGATAATAATAATAATAAAAATATAGAATATTTAGACAATGACAATTTGATAAAAATTATGTTTATACAAATATATATGCTTGC
AM1737      TCCATTGGATAATAATAATAATAAAAATATAGAATATTTAGACAATGGCAATTTGATAAAAATTATGTTTATACAAATATATATGCTTGC
SC74        TCCATTGGATAATAATAATAATAAAAATATAGAATATTTAGACAATGACAATTTGATAAAAATTATGTTTATACAAATATATATGCTTGC
TAB166      TCCATTGGATAATAATAATAATAAAAATATAGAATATTTAGACAATGGCAATTTGATAAAAATTATGTTTATACAAATATATATGCTTGC
TAB138      TCCATTGGATAATAATAATAATAAAAATATAGAATATTTAGACAATGGCAATTTGATAAAAATTATGTTTATACAAATATATATGCTTGC
TAB130      TCCATTGGATAATAATAATAATAAAAATATAGAATATTTAGACAATGGCAATTTGATAAAAATTATGTTTATACAAATATATATGCTTGC
TAB131      TCCATTGGATAATAATAATAATAAAAATATAGAATATTTAGACAATGACAATTTGATAAAAATTATGTTTATACAAATATATATGCTTGC
TAB151      TCCATTGGATAATAATAATAATAAAAATATAGAATATTTAGACAATGACAATTTGATAAAAATTATGTTTATACAAATATATATGCTTGC
TAB152      TCCATTGGATAATAATAATAATAAAAATATAGAATATTTAGACAATGACAATTTGATAAAAATTATGTTTATACAAATATATATGCTTGC
SC52        TCCATTGGATAATAATAATAATAAAAATATAGAATATTTAGACAATGACAATTTGATAAAAATTATGTTTATACAAATATATATGCTTGT
SC55        TCCATTGGATAATAATAATAATAAAAATATAGAATATTTAGACAATGGCAATTTGATAAAAATTATGTTTATACAAATATATATGCTTGC
SC03        TCCATTGGATAATAATAATAATAAAAATATAGAATATTTAGACAATGGCAATTTGATAAAAATTATGTTTATACAAATATATATGCTTGC
SC53        TCCATTGGATAATAATAATAATAAAAATATAGAATATTTAGACAATGACAATTTGATAAAAATTATGTTTATACAAATATATATGCTTGC
TAB141      TCCATTGGATAATAATAATAATAAAAATATAGAATATTTAGACAATGGCAATTTGATAAAAATTATGTTTATACAAATATATATGCTTGC
TAB123      TCCATTGGATAATAATAATAATAAAAATATAGAATATTTAGACAATGACAATTTGATAAAAATTATGTTTATACAAATATATATGCTTGC
AM1803      TCCATTGGATAATAATAATAATAAAAATATAGAATATTTAGACAATGACAATTTGATAAAAATTATGTTTATACAAATATATATGCTTGC
AM1814      TCCATTGGATAATAATAATAATAAAAATATAGAATATTTAGACAATGACAATTTGATAAAAATTATGTTTATACAAATATATATGCTTGC
AM1802      TCCATTGGATAATAATAATAATAAAAATATAGAATATTTAGACAATGACAATTTGATAAAAATTATGTTTATACAAATATATATGCTTGT
AM1811      TCCATTGGATAATAATAATAATAAAAATATAGAATATTTAGACAATGGCAATTTGATAAAAATTATGTTTATACAAATATATATGCTTGC
SC50        TCCATTGGATAATAATAATAATAAAAATATAGAATATTTAGACAATGACAATTTGATAAAAATTATGTTTATACAAATATATATGCTTGC
SC56        TCCATTGGATAATAATAATAATAAAAATATAGAATATTTAGACAATGGCAATTTGATAAAAATTATGTTTATACAAATATATATGCTTGC
TAB153      TCCATTGGATAATAATAATAATAAAAATATAGAATATTTAGACAATGACAATTTGATAAAAATTATGTTTATACAAATATATATGCTTGT
TAB154      TCCATTGGATAATAATAATAATAAAAATATAGAATATTTAGACAATGACAATTTGATAAAAATTATGTTTATACAAATATATATGCTTGC
TAB156      TCCATTGGATAATAATAATAATAAAAATATAGAATATTTAGACAATGACAATTTGATAAAAATTATGTTTATACAAATATATATGCTTGT
TAB136      TCCATTGGATAATAATAATAATAAAAATATAGAATATTTAGACAATGGCAATTTGATAAAAATTATGTTTATACAAATATATATGCTTGC
AM1880      TCCATTGGATAATAATAATAATAAAAATATAGAATATTTAGACAATGGCAATTTGATAAAAATTATGTTTATACAAATATATATGCTTGC
PF3D7_      TCCATTGGATAATAATAATAATAAAAATATAGAATATTTAGACAATGACAATTTGATAAAAATTATGTTTATACAAATATATATGCTTGC
PfSD01      TCCATTGGATAATAATAATAATAAAAATATAGAATATTTAGACAATGACAATTTGATAAAAATTATGTTTATACAAATATATATGCTTGC
Pf7G8       TCCATTGGATAATAATAATAATAAAAATATAGAATATTTAGACAATGACAATTTGATAAAAATTATGTTTATACAAATATATATGCTTGC
FCR3 GENE   TCCATTGGATAATAATAATAATAAAAATATAGAATATTTAGACAATGGCAATTTGATAAAAATTATGTTTATACAAATATATATGCTTGC
MS822 GENE  ------------------------------------------------------------------------------------------
PfCD01_     TCCATTGGATAATAATAATAATAAAAATATAGAATATTTAGACAATGACAATTTGATAAAAATTATGTTTATACAAATATATATGCTTGT
PfKH01__    TCCATTGGATAATAATAATAATAAAAATATAGAATATTTAGACAATGACAATTTGATAAAAATTATGTTTATACAAATATATATGCTTGC
PfDd2__     TCCATTGGATAATAATAATAATAAAAATATAGAATATTTAGACAATGGCAATTTGATAAAAATTATGTTTATACAAATATATATGCTTGC
PfSN01_     TCCATTGGATAATAATAATAATAAAAATATAGAATATTTAGACAATGACAATTTGATAAAAATTATGTTTATACAAATATATATGCTTGC
PfGN01      TCCATTGGATAATAATAATAATAAAAATATAGAATATTTAGACAATGACAATTTGATAAAAATTATGTTTATACAAATATATATGCTTGT
PfKE01      TCCATTGGATAATAATAATAATAAAAATATAGAATATTTAGACAATGACAATTTGATAAAAATTATGTTTATACAAATATATATGCTTGC
PfKH02      TCCATTGGATAATAATAATAATAAAAATATAGAATATTTAGACAATGACAATTTGATAAAAATTATGTTTATACAAATATATATGCTTGC
PfGB4       TCCATTGGATAATAATAATAATAAAAATATAGAATATTTAGACAATGGCAATTTGATAAAAATTATGTTTATACAAATATATATGCTTGC
PfGA01_     TCCATTGGATAATAATAATAATAAAAATATAGAATATTTAGACAATGACAATTTGATAAAAATTATGTTTATACAAATATATATGCTTGC
PfHB3_      TCCATTGGATAATAATAATAATAAAAATATAGAATATTTAGACAATGACAATTTGATAAAAATTATGTTTATACAAATATATATGCTTGC
PfIT_       TCCATTGGATAATAATAATAATAAAAATATAGAATATTTAGACAATGGCAATTTGATAAAAATTATGTTTATACAAATATATATGCTTGC
```

```
                        3520      3530      3540      3550      3560      3570      3580      3590      3600
               ....|....|....|....|....|....|....|....|....|....|....|....|....|....|....|....|....|....|
PF3D7 CDS      TTTAGAAGAATATAAAAAGGAACAATTTTTTCAAAACGTGAGCACATTTTTGGATATATGTTTGGATGATATAAAAGAGAAACAAACAAA
MS822 mRNA     ------------------------------------------------------------------------------------------
FCR3 mRNA      TTTAGAAGAATATAAAAAGGAACAATTTTTTCAAAACGTGAGCACATTTTTGGATATATGTTTGGATGATATAAAAGAGAAACAAACAAA
AM1898         TTTAGAAGAATATAAAAAGGAACAATTTTTTCAAAACGTGAGCACATTTTTGGATATATGTTTGGATGATATAAAAGAGAAACAAACAAA
AA235          TTTAGAAGAATATAAAAAGGAACAATTTTTTCAAAACGTGAGCACATTTTTGGATATATGTTTGGATGATATAAAAGAGAAACAAACAAA
AA243          TTTAGAAGAATATAAAAAGGAACAATTTTTTCAAAACGTGAGCACATTTTTGGATATATGTTTGGATGATATAAAAGAGAAACAAACAAA
AA258          TTTAGAAGAATATAAAAAGGAACAATTTTTTCAAAACGTGAGCACATTTTTGGATATATGTTTGGATGATATAAAAGAGAAACAAACAAA
AA264          TTTAGAAGAATATAAAAAGGAACAATTTTTTCAAAACGTGAGCACATTTTTGGATATATGTTTGGATGATATAAAAGAGAAACAAACAAA
AM1737         TTTAGAAGAATATAAAAAGGAACAATTTTTTCAAAACGTGAGCACATTTTTGGATATATGTTTGGATGATATAAAAGAGAAACAAACAAA
SC74           TTTAGAAGAATATAAAAAGGAACAATTTTTTCAAAACGTGAGCACATTTTTGGATATATGTTTGGATGATATAAAAGAGAAACAAACAAA
TAB166         TTTAGAAGAATATAAAAAGGAACAATTTTTTCAAAACGTGAGCACATTTTTGGATATATGTTTGGATGATATAAAAGAGAAACAAACAAA
TAB138         TTTAGAAGAATATAAAAAGGAACAATTTTTTCAAAACGTGAGCACATTTTTGGATATATGTTTGGATGATATAAAAGAGAAACAAACAAA
TAB130         TTTAGAAGAATATAAAAAGGAACAATTTTTTCAAAACGTGAGCACATTTTTGGATATATGTTTGGATGATATAAAAGAGAAACAAACAAA
TAB131         TTTAGAAGAATATAAAAAGGAACAATTTTTTCAAAACGTGAGCACATTTTTGGATATATGTTTGGATGATATAAAAGAGAAACAAACAAA
TAB151         TTTAGAAGAATATAAAAAGGAACAATTTTTTCAAAACGTGAGCACATTTTTGGATATATGTTTGGATGATATAAAAGAGAAACAAACAAA
TAB152         TTTAGAAGAATATAAAAAGGAACAATTTTTTCAAAACGTGAGCACATTTTTGGATATATGTTTGGATGATATAAAAGAGAAACAAACAAA
SC52           ATTAGAAGAATATAAAAAGGAACAATTTTTTCAAAACGTGAGCACATTTTTGGATATATGTTTGGATGATATAAAAGAGAAACAAACAAA
SC55           TTTAGAAGAATATAAAAAGGAACAATTTTTTCAAAACGTGAGCACATTTTTGGATATATGTTTGGATGATATAAAAGAGAAACAAACAAA
SC03           ATTAGAAGAATATAAAAAGGAACAATTTTTTCAAAACGTGAGCACATTTTTGGATATATGTTTGGATGATATAAAAGAGAAACAAACAAA
SC53           TTTAGAAGAATATAAAAAGGAACAATTTTTTCAAAACGTGAGCACATTTTTGGATATATGTTTGGATGATATAAAAGAGAAACAAACAAA
TAB141         TTTAGAAGAATATAAAAAGGAACAATTTTTTCAAAACGTGAGCACATTTTTGGATATATGTTTGGATGATATAAAAGAGAAACAAACAAA
TAB123         TTTAGAAGAATATAAAAAGGAACAATTTTTTCAAAACGTGAGCACATTTTTGGATATATGTTTGGATGATATAAAAGAGAAACAAACAAA
AM1803         TTTAGAAGAATATAAAAAGGAACAATTTTTTCAAAACGTGAGCACATTTTTGGATATATGTTTGGATGATATAAAAGAGAAACAAACAAA
AM1814         TTTAGAAGAATATAAAAAGGAACAATTTTTTCAAAACGTGAGCACATTTTTGGATATATGTTTGGATGATATAAAAGAGAAACAAACAAA
AM1802         ATTAGAAGAATATAAAAAGGAACAATTTTTTCAAAACGTGAGCACATTTTTGGATATATGTTTGGATGATATAAAAGAGAAACAAACAAA
AM1811         TTTAGAAGAATATAAAAAGGAACAATTTTTTCAAAACGTGAGCACATTTTTGGATATATGTTTGGATGATATAAAAGAGAAACAAACAAA
SC50           TTTAGAAGAATATAAAAAGGAACAATTTTTTCAAAACGTGAGCACATTTTTGGATATATGTTTGGATGATATAAAAGAGAAACAAACAAA
SC56           TTTAGAAGAATATAAAAAGGAACAATTTTTTCAAAACGTGAGCACATTTTTGGATATATGTTTGGATGATATAAAAGAGAAACAAACAAA
TAB153         ATTAGAAGAATATAAAAAGGAACAATTTTTTCAAAACGTGAGCACATTTTTGGATATATGTTTGGATGATATAAAAGAGAAACAAACAAA
TAB154         TTTAGAAGAATATAAAAAGGAACAATTTTTTCAAAACGTGAGCACATTTTTGGATATATGTTTGGATGATATAAAAGAGAAACAAACAAA
TAB156         ATTAGAAGAATATAAAAAGGAACAATTTTTTCAAAACGTGAGCACATTTTTGGATATATGTTTGGATGATATAAAAGAGAAACAAACAAA
TAB136         TTTAGAAGAATATAAAAAGGAACAATTTTTTCAAAACGTGAGCACATTTTTGGATATATGTTTGGATGATATAAAAGAGAAACAAACAAA
AM1880         TTTAGAAGAATATAAAAAGGAACAATTTTTTCAAAACGTGAGCACATTTTTGGATATATGTTTGGATGATATAAAAGAGAAACAAACAAA
PF3D7_         TTTAGAAGAATATAAAAAGGAACAATTTTTTCAAAACGTGAGCACATTTTTGGATATATGTTTGGATGATATAAAAGAGAAACAAACAAA
PfSD01         TTTAGAAGAATATAAAAAGGAACAATTTTTTCAAAACGTGAGCACATTTTTGGATATATGTTTGGATGATATAAAAGAGAAACAAACAAA
Pf7G8          TTTAGAAGAATATAAAAAGGAACAATTTTTTCAAAACGTGAGCACATTTTTGGATATATGTTTGGATGATATAAAAGAGAAACAAACAAA
FCR3 GENE      TTTAGAAGAATATAAAAAGGAACAATTTTTTCAAAACGTGAGCACATTTTTGGATATATGTTTGGATGATATAAAAGAGAAACAAACAAA
MS822 GENE     ------------------------------------------------------------------------------------------
PfCD01_        ATTAGAAGAATATAAAAAGGAACAATTTTTTCAAAACGTGAGCACATTTTTGGATATATGTTTGGATGATATAAAAGAGAAACAAACAAA
PfKH01__       TTTAGAAGAATATAAAAAGGAACAATTTTTTCAAAACGTGAGCACATTTTTGGATATATGTTTGGATGATATAAAAGAGAAACAAACAAA
PfDd2__        TTTAGAAGAATATAAAAAGGAACAATTTTTTCAAAACGTGAGCACATTTTTGGATATATGTTTGGATGATATAAAAGAGAAACAAACAAA
PfSN01_        TTTAGAAGAATATAAAAAGGAACAATTTTTTCAAAACGTGAGCACATTTTTGGATATATGTTTGGATGATATAAAAGAGAAACAAACAAA
PfGN01         ATTAGAAGAATATAAAAAGGAACAATTTTTTCAAAACGTGAGCACATTTTTGGATATATGTTTGGATGATATAAAAGAGAAACAAACAAA
PfKE01         TTTAGAAGAATATAAAAAGGAACAATTTTTTCAAAACGTGAGCACATTTTTGGATATATGTTTGGATGATATAAAAGAGAAACAAACAAA
PfKH02         TTTAGAAGAATATAAAAAGGAACAATTTTTTCAAAACGTGAGCACATTTTTGGATATATGTTTGGATGATATAAAAGAGAAACAAACAAA
PfGB4          TTTAGAAGAATATAAAAAGGAACAATTTTTTCAAAACGTGAGCACATTTTTGGATATATGTTTGGATGATATAAAAGAGAAACAAACAAA
PfGA01_        TTTAGAAGAATATAAAAAGGAACAATTTTTTCAAAACGTGAGCACATTTTTGGATATATGTTTGGATGATATAAAAGAGAAACAAACAAA
PfHB3_         TTTAGAAGAATATAAAAAGGAACAATTTTTTCAAAACGTGAGCACATTTTTGGATATATGTTTGGATGATATAAAAGAGAAACAAACAAA
PfIT_          TTTAGAAGAATATAAAAAGGAACAATTTTTTCAAAACGTGAGCACATTTTTGGATATATGTTTGGATGATATAAAAGAGAAACAAACAAA
```

```
                   3610      3620      3630      3640      3650      3660      3670      3680      3690
             ....|....|....|....|....|....|....|....|....|....|....|....|....|....|....|....|....|....|
PF3D7 CDS    TACTGAAGAAACGAATAATACGTTAAAATTAATAAGGGCAATGGAAAATAAATATATCTTGAAAAAGAATAAAGAGAAAGAAATTATTAA
MS822 mRNA   ------------------------------------------------------------------------------------------
FCR3 mRNA    TACTGATGAAACGAATAATACGTTAAAATTAATAAGGGCAATGGAAAATAAATATATCTTGAAAAAGAATAAAGAGAAAGAAATTATTAA
AM1898       TACTGAAGAAACGAATAATACGTTAAAATTAATAAGGGCAATGGAAAATAAATATATCTTGAAAAAGAATAAAGAGAAAGAAATTATTAA
AA235        TACTGAAGAAACGAATAATACGTTAAAATTAATAAGGGCAATGGAAAATAAATATATCTTGAAAAAGAATAAAGAGAAAGAAATTATTAA
AA243        TACTGAAGAAACGAATAATACGTTAAAATTAATAAGGGCAATGGAAAATAAATATATCTTGAAAAAGAATAAAGAGAAAGAAATTATTAA
AA258        TACTGAAGAAACGAATAATACGTTAAAATTAATAAGGGCAATGGAAAATAAATATATCTTGAAAAAGAATAAAGAGAAAGAAATTATTAA
AA264        TACTGAAGAAACGAATAATACGTTAAAATTAATAAGGGCAATGGAAAATAAATATATCTTGAAAAAGAATAAAGAGAAAGAAATTATTAA
AM1737       TACTGAAGAAACGAATAATACGTTAAAATTAATAAGGGCAATGGAAAATAAATATATCTTGAAAAAGAATAAAGAGAAAGAAATTATTAA
SC74         TACTGAAGAAACGAATAATACGTTAAAATTAATAAGGGCAATGGAAAATAAATATATCTTGAAAAAGAATAAAGAGAAAGAAATTATTAA
TAB166       TACTGAAGAAACGAATAATACGTTAAAATTAATAAGGGCAATGGAAAATAAATATATCTTGAAAAAGAATAAAGAGAAAGAAATTATTAA
TAB138       TACTGAAGAAACGAATAATACGTTAAAATTAATAAGGGCAATGGAAAATAAATATATCTTGAAAAAGAATAAAGAGAAAGAAATTATTAA
TAB130       TACTGAAGAAACGAATAATACGTTAAAATTAATAAGGGCAATGGAAAATAAATATATCTTGAAAAAGAATAAAGAGAAAGAAATTATTAA
TAB131       TACTGAAGAAACGAATAATACGTTAAAATTAATAAGGGCAATGGAAAATAAATATATCTTGAAAAAGAATAAAGAGAAAGAAATTATTAA
TAB151       TACTGAAGAAACGAATAATACGTTAAAATTAATAAGGGCAATGGAAAATAAATATATCTTGAAAAAGAATAAAGAGAAAGAAATTATTAA
TAB152       TACTGAAGAAACGAATAATACGTTAAAATTAATAAGGGCAATGGAAAATAAATATATCTTGAAAAAGAATAAAGAGAAAGAAATTATTAA
SC52         TACTGATGAAACGAATAATACGTTAAAATTAATAAGGGCAATGGAAAATAAATATATCTTGAAAAAGAATAAAGAGAAAGAAATTATTAA
SC55         TACTGAAGAAACGAATAATACGTTAAAATTAATAAGGGCAATGGAAAATAAATATATCTTGAAAAAGAATAAAGAGAAAGAAATTATTAA
SC03         TACTGAAGAAACGAATAATACGTTAAAATTAATAAGGGCAATGGAAAATAAATATATCTTGAAAAAGAATAAAGAGAAAGAAATTATTAA
SC53         TACTGAAGAAACGAATAATACGTTAAAATTAATAAGGGCAATGGAAAATAAATATATCTTGAAAAAGAATAAAGAGAAAGAAATTATTAA
TAB141       TACTGAAGAAACGAATAATACGTTAAAATTAATAAGGGCAATGGAAAATAAATATATCTTGAAAAAGAATAAAGAGAAAGAAATTATTAA
TAB123       TACTGAAGAAACGAATAATACGTTAAAATTAATAAGGGCAATGGAAAATAAATATATCTTGAAAAAGAATAAAGAGAAAGAAATTATTAA
AM1803       TACTGAAGAAACGAATAATACGTTAAAATTAATAAGGGCAATGGAAAATAAATATATCTTGAAAAAGAATAAAGAGAAAGAAATTATTAA
AM1814       TACTGAAGAAACGAATAATACGTTAAAATTAATAAGGGCAATGGAAAATAAATATATCTTGAAAAAGAATAAAGAGAAAGAAATTATTAA
AM1802       TACTGATGAAACGAATAATACGTTAAAATTAATAAGGGCAATGGAAAATAAATATATCTTGAAAAAGAATAAAGAGAAAGAAATTATTAA
AM1811       TACTGAAGAAACGAATAATACGTTAAAATTAATAAGGGCAATGGAAAATAAATATATCTTGAAAAAGAATAAAGAGAAAGAAATTATTAA
SC50         TACTGATGAAACGAATAATACGTTAAAATTAATAAGGGCAATGGAAAATAAATATATCTTGAAAAAGAATAAAGAGAAAGAAATTATTAA
SC56         TACTGAAGAAACGAATAATACGTTAAAATTAATAAGGGCAATGGAAAATAAATATATCTTGAAAAAGAATAAAGAGAAAGAAATTATTAA
TAB153       TACTGATGAAACGAATAATACGTTAAAATTAATAAGGGCAATGGAAAATAAATATATCTTGAAAAAGAATAAAGAGAAAGAAATTATTAA
TAB154       TACTGAAGAAACGAATAATACGTTAAAATTAATAAGGGCAATGGAAAATAAATATATCTTGAAAAAGAATAAAGAGAAAGAAATTATTAA
TAB156       TACTGATGAAACGAATAATACGTTAAAATTAATAAGGGCAATGGAAAATAAATATATCTTGAAAAAGAATAAAGAGAAAGAAATTATTAA
TAB136       TACTGAAGAAACGAATAATACGTTAAAATTAATAAGGGCAATGGAAAATAAATATATCTTGAAAAAGAATAAAGAGAAAGAAATTATTAA
AM1880       TACTGAAGAAACGAATAATACGTTAAAATTAATAAGGGCAATGGAAAATAAATATATCTTGAAAAAGAATAAAGAGAAAGAAATTATTAA
PF3D7_       TACTGAAGAAACGAATAATACGTTAAAATTAATAAGGGCAATGGAAAATAAATATATCTTGAAAAAGAATAAAGAGAAAGAAATTATTAA
PfSD01       TACTGAAGAAACGAATAATACGTTAAAATTAATAAGGGCAATGGAAAATAAATATATCTTGAAAAAGAATAAAGAGAAAGAAATTATTAA
Pf7G8        TACTGAAGAAACGAATAATACGTTAAAATTAATAAGGGCAATGGAAAATAAATATATCTTGAAAAAGAATAAAGAGAAAGAAATTATTAA
FCR3 GENE    TACTGATGAAACGAATAATACGTTAAAATTAATAAGGGCAATGGAAAATAAATATATCTTGAAAAAGAATAAAGAGAAAGAAATTATTAA
MS822 GENE   ------------------------------------------------------------------------------------------
PfCD01_      TACTGATGAAACGAATAATACGTTAAAATTAATAAGGGCAATGGAAAATAAATATATCTTGAAAAAGAATAAAGAGAAAGAAATTATTAA
PfKH01__     TACTGAAGAAACGAATAATACGTTAAAATTAATAAGGGCAATGGAAAATAAATATATCTTGAAAAAGAATAAAGAGAAAGAAATTATTAA
PfDd2__      TACTGAAGAAACGAATAATACGTTAAAATTAATAAGGGCAATGGAAAATAAATATATCTTGAAAAAGAATAAAGAGAAAGAAATTATTAA
PfSN01_      TACTGAAGAAACGAATAATACGTTAAAATTAATAAGGGCAATGGAAAATAAATATATCTTGAAAAAGAATAAAGAGAAAGAAATTATTAA
PfGN01       TACTGATGAAACGAATAATACGTTAAAATTAATAAGGGCAATGGAAAATAAATATATCTTGAAAAAGAATAAAGAGAAAGAAATTATTAA
PfKE01       TACTGAAGAAACGAATAATACGTTAAAATTAATAAGGGCAATGGAAAATAAATATATCTTGAAAAAGAATAAAGAGAAAGAAATTATTAA
PfKH02       TACTGAAGAAACGAATAATACGTTAAAATTAATAAGGGCAATGGAAAATAAATATATCTTGAAAAAGAATAAAGAGAAAGAAATTATTAA
PfGB4        TACTGAAGAAACGAATAATACGTTAAAATTAATAAGGGCAATGGAAAATAAATATATCTTGAAAAAGAATAAAGAGAAAGAAATTATTAA
PfGA01_      TACTGAAGAAACGAATAATACGTTAAAATTAATAAGGGCAATGGAAAATAAATATATCTTGAAAAAGAATAAAGAGAAAGAAATTATTAA
PfHB3_       TACTGAAGAAACGAATAATACGTTAAAATTAATAAGGGCAATGGAAAATAAATATATCTTGAAAAAGAATAAAGAGAAAGAAATTATTAA
PfIT_        TACTGATGAAACGAATAATACGTTAAAATTAATAAGGGCAATGGAAAATAAATATATCTTGAAAAAGAATAAAGAGAAAGAAATTATTAA
```

	3700 3710 3720 3730 3740 3750 3760 3770 3780
	....|....|....|....|....|....|....|....|....|....|....|....|....|....|....|....|....|....|
**PF3D7 CDS**	TCAATTTAAAAATGAAATGTTTTTTAAAAATATTATGTGCGACTGGAAAAATTGTGAGGATAAATTTATAAATGATCATTTTATTCAGGA
**MS822 mRNA**	--
**FCR3 mRNA**	TCAATTTAAAAATGAAATGTTTTTTAAAAATATTATGTGCGACTGGAAAAATTGTGAGGATAAATTTATAAATGATCATTTTATTCAGGA
**AM1898**	TCAATTTAAAAATGAAATGTTTTTTAAAAATATTATGTGCGACTGGAAAAATTGTGAGGATAAATTTATAAATGATCATTTTATTCAGGA
**AA235**	TCAATTTAAAAATGAAATGTTTTTTAAAAATATTATGTGCGACTGGAAAAATTGTGAGGATAAATTTATAAATGATCATTTTATTCAGGA
**AA243**	TCAATTTAAAAATGAAATGTTTTTTAAAAATATTATGTGCGACTGGAAAAATTGTGAGGATAAATTTATAAATGATCATTTTATTCAGGA
**AA258**	TCAATTTAAAAATGAAATGTTTTTTAAAAATATTATGTGCGACTGGAAAAATTGTGAGGATAAATTTATAAATGATCATTTTATTCAGGA
**AA264**	TCAATTTAAAAATGAAATGTTTTTTAAAAATATTATGTGCGACTGGAAAAATTGTGAGGATAAATTTATAAATGATCATTTTATTCAGGA
**AM1737**	TCAATTTAAAAATGAAATGTTTTTTAAAAATATTATGTGCGACTGGAAAAATTGTGAGGATAAATTTATAAATGATCATTTTATTCAGGA
**SC74**	TCAATTTAAAAATGAAATGTTTTTTAAAAATATTATGTGCGACTGGAAAAATTGTGAGGATAAATTTATAAATGATCATTTTATTCAGGA
**TAB166**	TCAATTTAAAAATGAAATGTTTTTTAAAAATATTATGTGCGACTGGAAAAATTGTGAGGATAAATTTATAAATGATCATTTTATTCAGGA
**TAB138**	TCAATTTAAAAATGAAATGTTTTTTAAAAATATTATGTGCGACTGGAAAAATTGTGAGGATAAATTTATAAATGATCATTTTATTCAGGA
**TAB130**	TCAATTTAAAAATGAAATGTTTTTTAAAAATATTATGTGCGACTGGAAAAATTGTGAGGATAAATTTATAAATGATCATTTTATTCAGGA
**TAB131**	TCAATTTAAAAATGAAATGTTTTTTAAAAATATTATGTGCGACTGGAAAAATTGTGAGGATAAATTTATAAATGATCATTTTATTCAGGA
**TAB151**	TCAATTTAAAAATGAAATGTTTTTTAAAAATATTATGTGCGACTGGAAAAATTGTGAGGATAAATTTATAAATGATCATTTTATTCAGGA
**TAB152**	TCAATTTAAAAATGAAATGTTTTTTAAAAATATTATGTGCGACTGGAAAAATTGTGAGGATAAATTTATAAATGATCATTTTATTCAGGA
**SC52**	TCAATTTAAAAATGAAATGTTTTTTAAAAATATTATGTGCGACTGGAAAAATTGTGAGGATAAATTTATAAATGATCATTTTATTCAGGA
**SC55**	TCAATTTAAAAATGAAATGTTTTTTAAAAATATTATGTGCGACTGGAAAAATTGTGAGGATAAATTTATAAATGATCATTTTATTCAGGA
**SC03**	TCAATTTAAAAATGAAATGTTTTTTAAAAATATTATGTGCGACTGGAAAAATTGTGAGGATAAATTTATAAATGATCATTTTATTCAGGA
**SC53**	TCAATTTAAAAATGAAATGTTTTTTAAAAATATTATGTGCGACTGGAAAAATTGTGAGGATAAATTTATAAATGATCATTTTATTCAGGA
**TAB141**	TCAATTTAAAAATGAAATGTTTTTTAAAAATATTATGTGCGACTGGAAAAATTGTGAGGATAAATTTATAAATGATCATTTTATTCAGGA
**TAB123**	TCAATTTAAAAATGAAATGTTTTTTAAAAATATTATGTGCGACTGGAAAAATTGTGAGGATAAATTTATAAATGATCATTTTATTCAGGA
**AM1803**	TCAATTTAAAAATGAAATGTTTTTTAAAAATATTATGTGCGACTGGAAAAATTGTGAGGATAAATTTATAAATGATCATTTTATTCAGGA
**AM1814**	TCAATTTAAAAATGAAATGTTTTTTAAAAATATTATGTGCGACTGGAAAAATTGTGAGGATAAATTTATAAATGATCATTTTATTCAGGA
**AM1802**	TCAATTTAAAAATGAAATGTTTTTTAAAAATATTATGTGCGACTGGAAAAATTGTGAGGATAAATTTATAAATGATCATTTTATTCAGGA
**AM1811**	TCAATTTAAAAATGAAATGTTTTTTAAAAATATTATGTGCGACTGGAAAAATTGTGAGGATAAATTTATAAATGATCATTTTATTCAGGA
**SC50**	TCAATTTAAAAATGAAATGTTTTTTAAAAATATTATGTGCGACTGGAAAAATTGTGAGGATAAATTTATAAATGATCATTTTATTCAGGA
**SC56**	TCAATTTAAAAATGAAATGTTTTTTAAAAATATTATGTGCGACTGGAAAAATTGTGAGGATAAATTTATAAATGATCATTTTATTCAGGA
**TAB153**	TCAATTTAAAAATGAAATGTTTTTTAAAAATATTATGTGCGACTGGAAAAATTGTGAGGATAAATTTATAAATGATCATTTTATTCAGGA
**TAB154**	TCAATTTAAAAATGAAATGTTTTTTAAAAATATTATGTGCGACTGGAAAAATTGTGAGGATAAATTTATAAATGATCATTTTATTCAGGA
**TAB156**	TCAATTTAAAAATGAAATGTTTTTTAAAAATATTATGTGCGACTGGAAAAATTGTGAGGATAAATTTATAAATGATCATTTTATTCAGGA
**TAB136**	TCAATTTAAAAATGAAATGTTTTTTAAAAATATTATGTGCGACTGGAAAAATTGTGAGGATAAATTTATAAATGATCATTTTATTCAGGA
**AM1880**	TCAATTTAAAAATGAAATGTTTTTTAAAAATATTATGTGCGACTGGAAAAATTGTGAGGATAAATTTATAAATGATCATTTTATTCAGGA
**PF3D7_**	TCAATTTAAAAATGAAATGTTTTTTAAAAATATTATGTGCGACTGGAAAAATTGTGAGGATAAATTTATAAATGATCATTTTATTCAGGA
**PfSD01**	TCAATTTAAAAATGAAATGTTTTTTAAAAATATTATGTGCGACTGGAAAAATTGTGAGGATAAATTTATAAATGATCATTTTATTCAGGA
**Pf7G8**	TCAATTTAAAAATGAAATGTTTTTTAAAAATATTATGTGCGACTGGAAAAATTGTGAGGATAAATTTATAAATGATCATTTTATTCAGGA
**FCR3 GENE**	TCAATTTAAAAATGAAATGTTTTTTAAAAATATTATGTGCGACTGGAAAAATTGTGAGGATAAATTTATAAATGATCATTTTATTCAGGA
**MS822 GENE**	--
**PfCD01_**	TCAATTTAAAAATGAAATGTTTTTTAAAAATATTATGTGCGACTGGAAAAATTGTGAGGATAAATTTATAAATGATCATTTTATTCAGGA
**PfKH01__**	TCAATTTAAAAATGAAATGTTTTTTAAAAATATTATGTGCGACTGGAAAAATTGTGAGGATAAATTTATAAATGATCATTTTATTCAGGA
**PfDd2__**	TCAATTTAAAAATGAAATGTTTTTTAAAAATATTATGTGCGACTGGAAAAATTGTGAGGATAAATTTATAAATGATCATTTTATTCAGGA
**PfSN01_**	TCAATTTAAAAATGAAATGTTTTTTAAAAATATTATGTGCGACTGGAAAAATTGTGAGGATAAATTTATAAATGATCATTTTATTCAGGA
**PfGN01**	TCAATTTAAAAATGAAATGTTTTTTAAAAATATTATGTGCGACTGGAAAAATTGTGAGGATAAATTTATAAATGATCATTTTATTCAGGA
**PfKE01**	TCAATTTAAAAATGAAATGTTTTTTAAAAATATTATGTGCGACTGGAAAAATTGTGAGGATAAATTTATAAATGATCATTTTATTCAGGA
**PfKH02**	TCAATTTAAAAATGAAATGTTTTTTAAAAATATTATGTGCGACTGGAAAAATTGTGAGGATAAATTTATAAATGATCATTTTATTCAGGA
**PfGB4**	TCAATTTAAAAATGAAATGTTTTTTAAAAATATTATGTGCGACTGGAAAAATTGTGAGGATAAATTTATAAATGATCATTTTATTCAGGA
**PfGA01_**	TCAATTTAAAAATGAAATGTTTTTTAAAAATATTATGTGCGACTGGAAAAATTGTGAGGATAAATTTATAAATGATCATTTTATTCAGGA
**PfHB3_**	TCAATTTAAAAATGAAATGTTTTTTAAAAATATTATGTGCGACTGGAAAAATTGTGAGGATAAATTTATAAATGATCATTTTATTCAGGA
**PfIT_**	TCAATTTAAAAATGAAATGTTTTTTAAAAATATTATGTGCGACTGGAAAAATTGTGAGGATAAATTTATAAATGATCATTTTATTCAGGA

```
                     3790      3800      3810      3820      3830      3840      3850      3860      3870
             ....|....|....|....|....|....|....|....|....|....|....|....|....|....|....|....|....|....|
PF3D7 CDS    AAAAATTAATATACATAACATGAAAAAGGATATTCCCATAAAACATTGGAATCATATATATCATAAATGGTTACATGAGGAGAACAAAAA
MS822 mRNA   ------------------------------------------------------------------------------------------
FCR3 mRNA    AAAAATTAATATACATAACATGAAAAAGGATATTCCCATAAAACATTGGAATCACATATATCATAAATGGTTACATGAGGAGAACAAAAA
AM1898       AAAAATTAATATACATAACATGAAAAAGGATATTCCCATAAAACATTGGAATCACATATATCATAAATGGTTACATGAGGAGAACAAAAA
AA235        AAAAATTAATATACATAACATGAAAAAGGATATTCCCATAAAACATTGGAATCATATATATCATAAATGGTTACATGAGGAGAACAAAAA
AA243        AAAAATTAATATACATAACATGAAAAAGGATATTCCCATAAAACATTGGAATCATATATATCATAAATGGTTACATGAGGAGAACAAAAA
AA258        AAAAATTAATATACATAACATGAAAAAGGATATTCCCATAAAACATTGGAATCATATATATCATAAATGGTTACATGAGGAGAACAAAAA
AA264        AAAAATTAATATACATAACATGAAAAAGGATATTCCCATAAAACATTGGAATCATATATATCATAAATGGTTACATGAGGAGAACAAAAA
AM1737       AAAAATTAATATACATAACATGAAAAAGGATATTCCCATAAAACATTGGAATCATATATATCATAAATGGTTACATGAGGAGAACAAAAA
SC74         AAAAATTAATATACATAACATGAAAAAGGATATTCCCATAAAACATTGGAATCATATATATCATAAATGGTTACATGAGGAGAACAAAAA
TAB166       AAAAATTAATATACATAACATGAAAAAGGATATTCCCATAAAACATTGGAATCATATATATCATAAATGGTTACATGAGGAGAACAAAAA
TAB138       AAAAATTAATATACATAACATGAAAAAGGATATTCCCATAAAACATTGGAATCATATATATCATAAATGGTTACATGAGGAGAACAAAAA
TAB130       AAAAATTAATATACATAACATGAAAAAGGATATTCCCATAAAACATTGGAATCATATATATCATAAATGGTTACATGAGGAGAACAAAAA
TAB131       AAAAATTAATATACATAACATGAAAAAGGATATTCCCATAAAACATTGGAATCATATATATCATAAATGGTTACATGAGGAGAACAAAAA
TAB151       AAAAATTAATATACATAACATGAAAAAGGATATTCCCATAAAACATTGGAATCATATATATCATAAATGGTTACATGAGGAGAACAAAAA
TAB152       AAAAATTAATATACATAACATGAAAAAGGATATTCCCATAAAACATTGGAATCATATATATCATAAATGGTTACATGAGGAGAACAAAAA
SC52         AAAAATTAATATACATAACATGAAAAAGGATATTCCCATAAAACATTGGAATCATATATATCATAAATGGTTACATGAGGAGAACAAAAA
SC55         AAAAATTAATATACATAACATGAAAAAGGATATTCCCATAAAACATTGGAATCATATATATCATAAATGGTTACATGAGGAGAACAAAAA
SC03         AAAAATTAATATACATAACATGAAAAAGGATATTCCCATAAAACATTGGAATCATATATATCATAAATGGTTACATGAGGAGAACAAAAA
SC53         AAAAATTAATATACATAACATGAAAAAGGATATTCCCATAAAACATTGGAATCATATATATCATAAATGGTTACATGAGGAGAACAAAAA
TAB141       AAAAATTAATATACATAACATGAAAAAGGATATTCCCATAAAACATTGGAATCATATATATCATAAATGGTTACATGAGGAGAACAAAAA
TAB123       AAAAATTAATATACATAACATGAAAAAGGATATTCCCATAAAACATTGGAATCATATATATCATAAATGGTTACATGAGGAGAACAAAAA
AM1803       AAAAATTAATATACATAACATGAAAAAGGATATTCCCATAAAACATTGGAATCACATATATCATAAATGGTTACATGAGGAGAACAAAAA
AM1814       AAAAATTAATATACATAACATGAAAAAGGATATTCCCATAAAACATTGGAATCATATATATCATAAATGGTTACATGAGGAGAACAAAAA
AM1802       AAAAATTAATATACATAACATGAAAAAGGATATTCCCATAAAACATTGGAATCATATATATCATAAATGGTTACATGAGGAGAACAAAAA
AM1811       AAAAATTAATATACATAACATGAAAAAGGATATTCCCATAAAACATTGGAATCACATATATCATAAATGGTTACATGAGGAGAACAAAAA
SC50         AAAAATTAATATACATAACATGAAAAAGGATATTCCCATAAAACATTGGAATCATATATATCATAAATGGTTACATGAGGAGAACAAAAA
SC56         AAAAATTAATATACATAACATGAAAAAGGATATTCCCATAAAACATTGGAATCACATATATCATAAATGGTTACATGAGGAGAACAAAAA
TAB153       AAAAATTAATATACATAACATGAAAAAGGATATTCCCATAAAACATTGGAATCATATATATCATAAATGGTTACATGAGGAGAACAAAAA
TAB154       AAAAATTAATATACATAACATGAAAAAGGATATTCCCATAAAACATTGGAATCATATATATCATAAATGGTTACATGAGGAGAACAAAAA
TAB156       AAAAATTAATATACATAACATGAAAAAGGATATTCCCATAAAACATTGGAATCATATATATCATAAATGGTTACATGAGGAGAACAAAAA
TAB136       AAAAATTAATATACATAACATGAAAAAGGATATTCCCATAAAACATTGGAATCATATATATCATAAATGGTTACATGAGGAGAACAAAAA
AM1880       AAAAATTAATATACATAACATGAAAAAGGATATTCCCATAAAACATTGGAATCACATATATCATAAATGGTTACATGAGGAGAACAAAAA
PF3D7_       AAAAATTAATATACATAACATGAAAAAGGATATTCCCATAAAACATTGGAATCATATATATCATAAATGGTTACATGAGGAGAACAAAAA
PfSD01       AAAAATTAATATACATAACATGAAAAAGGATATTCCCATAAAACATTGGAATCATATATATCATAAATGGTTACATGAGGAGAACAAAAA
Pf7G8        AAAAATTAATATACATAACATGAAAAAGGATATTCCCATAAAACATTGGAATCATATATATCATAAATGGTTACATGAGGAGAACAAAAA
FCR3 GENE    AAAAATTAATATACATAACATGAAAAAGGATATTCCCATAAAACATTGGAATCACATATATCATAAATGGTTACATGAGGAGAACAAAAA
MS822 GENE   ------------------------------------------------------------------------------------------
PfCD01_      AAAAATTAATATACATAACATGAAAAAGGATATTCCCATAAAACATTGGAATCATATATATCATAAATGGTTACATGAGGAGAACAAAAA
PfKH01__     AAAAATTAATATACATAACATGAAAAAGGATATTCCCATAAAACATTGGAATCATATATATCATAAATGGTTACATGAGGAGAACAAAAA
PfDd2__      AAAAATTAATATACATAACATGAAAAAGGATATTCCCATAAAACATTGGAATCATATATATCATAAATGGTTACATGAGGAGAACAAAAA
PfSN01_      AAAAATTAATATACATAACATGAAAAAGGATATTCCCATAAAACATTGGAATCATATATATCATAAATGGTTACATGAGGAGAACAAAAA
PfGN01       AAAAATTAATATACATAACATGAAAAAGGATATTCCCATAAAACATTGGAATCATATATATCATAAATGGTTACATGAGGAGAACAAAAA
PfKE01       AAAAATTAATATACATAACATGAAAAAGGATATTCCCATAAAACATTGGAATCATATATATCATAAATGGTTACATGAGGAGAACAAAAA
PfKH02       AAAAATTAATATACATAACATGAAAAAGGATATTCCCATAAAACATTGGAATCATATATATCATAAATGGTTACATGAGGAGAACAAAAA
PfGB4        AAAAATTAATATACATAACATGAAAAAGGATATTCCCATAAAACATTGGAATCATATATATCATAAATGGTTACATGAGGAGAACAAAAA
PfGA01_      AAAAATTAATATACATAACATGAAAAAGGATATTCCCATAAAACATTGGAATCATATATATCATAAATGGTTACATGAGGAGAACAAAAA
PfHB3_       AAAAATTAATATACATAACATGAAAAAGGATATTCCCATAAAACATTGGAATCATATATATCATAAATGGTTACATGAGGAGAACAAAAA
PfIT_        AAAAATTAATATACATAACATGAAAAAGGATATTCCCATAAAACATTGGAATCACATATATCATAAATGGTTACATGAGGAGAACAAAAA
```

```
              3880      3890      3900      3910      3920      3930      3940      3950      3960
          ....|....|....|....|....|....|....|....|....|....|....|....|....|....|....|....|....|....|
PF3D7 CDS   GGATGATATTATTGTTCCATTAAAAGGTAAAGAGAAAGAAAAGAAACAAAATATTATAGATGAATCATCATTAGTTACAAAAAAAAAACA
MS822 mRNA  ------------------------------------------------------------------------------------------
FCR3 mRNA   GGATGATATTATTGTTCCATTAAAAAGGAAAGAGAAAGAAAAAGAACAAAATGTTATAGATGAATCATCATTAGTTACAAAAAAAAA-CA
AM1898      GGATGATATTATTGTTCCATTAAAAAGGAAAGAGAAAGAAAAAGAACAAAATGTTATAGATGAATCATCATTAGTTACAAAAAAAAA-CA
AA235       GGATGATATTATTGTTCCATTAAAAGGTAAAGAGAAAGAAAAGAAACAAAATATTATAGATGAATCATCATTAGTTACAAAAAAAAA-CA
AA243       GGATGATATTATTGTTCCATTAAAAGGTAAAGAGAAAGAAAAGAAACAAAATATTATAGATGAATCATCATTAGTTACAAAAAAAAA-CA
AA258       GGATGATATTATTGTTCCATTAAAAGGTAAAGAGAAAGAAAAGAAACAAAATATTATAGATGAATCATCATTAGTTACAAAAAAAAA-CA
AA264       GGATGATATTATTGTTCCATTAAAAGGTAAAGAGAAAGAAAAGAAACAAAATATTATAGATGAATCATCATTAGTTACAAAAAAAAA-CA
AM1737      GGATGATATTATTGTTCCATTAAAAGGTAAAGAGAAAGAAAAGAAACAAAATATTATAGATGAATCATCATTAGTTACAAAAAAAAAACA
SC74        GGATGATATTATTGTTCCATTAAAAGGTAAAGAGAAAGAAAAGAAACAAAATATTATAGATGAATCATCATTAGTTACAAAAAAAAAACA
TAB166      GGATGATATTATTGTTCCATTAAAAGGTAAAGAGAAAGAAAAGAAACAAAATATTATAGATGAATCATCATTAGTTACAAAAAAAAA-CA
TAB138      GGATGATATTATTGTTCCATTAAAAGGTAAAGAGAAAGAAAAGAAACAAAATATTATAGATGAATCATCATTAGTTACAAAAAAAAA-CA
TAB130      GGATGATATTATTGTTCCATTAAAAGGTAAAGAGAAAGAAAAGAAACAAAATATTATAGATGAATCATCATTAGTTACAAAAAAAAAACA
TAB131      GGATGATATTATTGTTCCATTAAAAGGTAAAGAGAAAGAAAAGAAACAAAATATTATAGATGAATCATCATTAGTTACAAAAAAAAAACA
TAB151      GGATGATATTATTGTTCCATTAAAAGGTAAAGAGAAAGAAAAGAAACAAAATATTATAGATGAATCATCATTAGTTACAAAAAAAAAACA
TAB152      GGATGATATTATTGTTCCATTAAAAGGTAAAGAGAAAGAAAAGAAACAAAATATTATAGATGAATCATCATTAGTTACAAAAAAAAAACA
SC52        GGATGATATTATTGTTCCATTAAAAGGTAAAGAGAAAGAAAAGAAACAAAATATTATAGATGAATCATCATTAGTTACAAAAAAAAA-CA
SC55        GGATGATATTATTGTTCCATTAAAAAGGAAAGAGAAAGAAAAAGAACAAAATATTATAGATGAATCATCATTAGTTACAAAAAAAAA-CA
SC03        GGATGATATTATTGTTCCATTAAAAGGTAAAGAGAAAGAAAAGAAACAAAATATTATAGATGAATCATCATTAGTTACAAAAAAAAAACA
SC53        GGATGATATTATTGTTCCATTAAAAGGTAAAGAGAAAGAAAAGAAACAAAATATTATAGATGAATCATCATTAGTTACAAAAAAAAAACA
TAB141      GGATGATATTATTGTTCCATTAAAAGGTAAAGAGAAAGAAAAGAAACAAAATATTATAGATGAATCATCATTAGTTACAAAAAAAAAACA
TAB123      GGATGATATTATTGTTCCATTAAAAGGTAAAGAGAAAGAAAAGAAACAAAATATTATAGATGAATCATCATTAGTTACAAAAAAAAA-CA
AM1803      GGATGATATTATTGTTCCATTAAAAAGGAAAGAGAAAGAAAAAGAACAAAATGTTATAGATGAATCATCATTAGTTACAAAAAAAAA-CA
AM1814      GGATGATATTATTGTTCCATTAAAAGGTAAAGAGAAAGAAAAGAAACAAAATATTATAGATGAATCATCATTAGTTACAAAAAAAAA-CA
AM1802      GGATGATATTATTGTTCCATTAAAAGGTAAAGAGAAAGAAAAGAAACAAAATATTATAGATGAATCATCATTAGTTACAAAAAAAAA-CA
AM1811      GGATGATATTATTGTTCCATTAAAAAGGAAAGAGAAAGAAAAAGAACAAAATGTTATAGATGAATCATCATTAGTTACAAAAAAAAA-CA
SC50        GGATGATATTATTGTTCCATTAAAAGGTAAAGAGAAAGAAAAGAAACAAAATATTATAGATGAATCATCATTAGTTACAAAAAAAAA-CA
SC56        GGATGATATTATTGTTCCATTAAAAAGGAAAGAGAAAGAAAAAGAACAAAATGTTATAGATGAATCATCATTAGTTACAAAAAAAAA-CA
TAB153      GGATGATATTATTGTTCCATTAAAAGGTAAAGAGAAAGAAAAGAAACAAAATATTATAGATGAATCATCATTAGTTACAAAAAAAAA-CA
TAB154      GGATGATATTATTGTTCCATTAAAAGGTAAAGAGAAAGAAAAGAAACAAAATATTATAGATGAATCATCATTAGTTACAAAAAAAAA-CA
TAB156      GGATGATATTATTGTTCCATTAAAAGGTAAAGAGAAAGAAAAGAAACAAAATATTATAGATGAATCATCATTAGTTACAAAAAAAAA-CA
TAB136      GGATGATATTATTGTTCCATTAAAAGGTAAAGAGAAAGAAAAGAAACAAAATATTATAGATGAATCATCATTAGTTACAAAAAAAAAACA
AM1880      GGATGATATTATTGTTCCATTAAAAAGGAAAGAGAAAGAAAAAGAACAAAATGTTATAGATGAATCATCATTAGTTACAAAAAAAAA-CA
PF3D7_      GGATGATATTATTGTTCCATTAAAAGGTAAAGAGAAAGAAAAGAAACAAAATATTATAGATGAATCATCATTAGTTACAAAAAAAAAACA
PfSD01      GGATGATATTATTGTTCCATTAAAAGGTAAAGAGAAAGAAAAGAAACAAAATATTATAGATGAATCATCATTAGTTACAAAAAAAAA-CA
Pf7G8       GGATGATATTATTGTTCCATTAAAAGGTAAAGAGAAAGAAAAGAAACAAAATATTATAGATGAATCATCATTAGTTACAAAAAAAAA-CA
FCR3 GENE   GGATGATATTATTGTTCCATTAAAAAGGAAAGAGAAAGAAAAAGAACAAAATGTTATAGATGAATCATCATTAGTTACAAAAAAAAA-CA
MS822 GENE  ------------------------------------------------------------------------------------------
PfCD01_     GGATGATATTATTGTTCCATTAAAAGGTAAAGAGAAAGAAAAGAAACAAAATATTATAGATGAATCATCATTAGTTACAAAAAAAAAACA
PfKH01__    GGATGATATTATTGTTCCATTAAAAGGTAAAGAGAAAGAAAAGAAACAAAATATTATAGATGAATCATCATTAGTTACAAAAAAAAAACA
PfDd2__     GGATGATATTATTGTTCCATTAAAAGGTAAAGAGAAAGAAAAGAAACAAAATATTATAGATGAATCATCATTAGTTACAAAAAAAAAACA
PfSN01_     GGATGATATTATTGTTCCATTAAAAGGTAAAGAGAAAGAAAAGAAACAAAATATTATAGATGAATCATCATTAGTTACAAAAAAAAA-CA
PfGN01      GGATGATATTATTGTTCCATTAAAAGGTAAAGAGAAAGAAAAGAAACAAAATATTATAGATGAATCATCATTAGTTACAAAAAAAAA-CA
PfKE01      GGATGATATTATTGTTCCATTAAAAGGTAAAGAGAAAGAAAAGAAACAAAATATTATAGATGAATCATCATTAGTTACAAAAAAAAA-CA
PfKH02      GGATGATATTATTGTTCCATTAAAAGGTAAAGAGAAAGAAAAGAAACAAAATATTATAGATGAATCATCATTAGTTACAAAAAAAAA-CA
PfGB4       GGATGATATTATTGTTCCATTAAAAGGTAAAGAGAAAGAAAAGAAACAAAATATTATAGATGAATCATCATTAGTTACAAAAAAAAA-CA
PfGA01_     GGATGATATTATTGTTCCATTAAAAGGTAAAGAGAAAGAAAAGAAACAAAATATTATAGATGAATCATCATTAGTTACAAAAAAAAA-CA
PfHB3_      GGATGATATTATTGTTCCATTAAAAGGTAAAGAGAAAGAAAAGAAACAAAATATTATAGATGAATCATCATTAGTTACAAAAAAAAA-CA
PfIT_       GGATGATATTATTGTTCCATTAAAAAGGAAAGAGAAAGAAAAAGAACAAAATGTTATAGATGAATCATCATTAGTTACAAAAAAAAA-CA
```

```
                  3970      3980      3990      4000      4010      4020      4030      4040      4050
             ....|....|....|....|....|....|....|....|....|....|....|....|....|....|....|....|....|....|
PF3D7 CDS    AATGGAAAACCGTTATAGAAATATATATGGAAGTGATGAAT-AATGTAAAAGAGACGAGTGGGAAGAACATCGAGGAGATTTTTTACAAA
MS822 mRNA   ------------------------------------------------------------------------------------------
FCR3 mRNA    AATGGAAAACCGTTATAGAAATATATATGGAAGTGATGAATGAATGTAAAAGAGACGAGTGGGAAGAACATCGAGGAGATTTTTTACAAA
AM1898       AATGGAAAACCGTTATAGAAATATATATGGAAGTGATGAATGAATGTAAAAGAGACGAGTGGGAAGAACATCGAGGAGATTTTTTACAAA
AA235        AATGGAAAACCGTTATAGAAATATATATGGAAGTGATGAATGAATGTAAAAGAGACGAGTGGGAAGAACATCGAGTAGATTTTTTACAAA
AA243        AATGGAAAACCGTTATAGAAATATATATGGAAGTGATGAATGAATGTAAAAGAGACGAGTGGGAAGAACATCGAGGAGATTTTTTACAAA
AA258        AATGGAAAACCGTTATAGAAATATATATGGAAGTGATGAATGAATGTAAAAGAGACGAGTGGGAAGAACATCGAGGAGATTTTTTACAAA
AA264        AATGGAAAACCGTTATAGAAATATATATGGAAGTGATGAATGAATGTAAAAGAGACGAGTGGGAAGAACATCGAGGAGATTTTTTACAAA
AM1737       AATGGAAAACCGTTATAGAAATATATATGGAAGTGATGAATGAATGTAAAAGAGACGAGTGGGAAGAACATCGAGGAGATTTTTTACAAA
SC74         AATGGAAAACCGTTATAGAAATATATATGGAAGTGATGAATGAATGTAAAAGAGACGAGTGGGAAGAACATCGAGGAGATTTTTTACAAA
TAB166       AATGGAAAACCGTTATAGAAATATATATGGAAGTGATGAATGAATGTAAAAGAGACGAGTGGGAAGAACATCGAGGAGATTTTTTACAAA
TAB138       AATGGAAAACCGTTATAGAAATATATATGGAAGTGATGAATGAATGTAAAAGAGACGAGTGGGAAGAACATCGAGGAGATTTTTTACAAA
TAB130       AATGGAAAACCGTTATAGAAATATATATGGAAGTGATGAATGAATGTAAAAGAGACGAGTGGGAAGAACATCGAGGAGATTTTTTACAAA
TAB131       AATGGAAAACCGTTATAGAAATATATATGGAAGTGATGAATGAATGTAAAAGAGACGAGTGGGAAGAACATCGAGGAGATTTTTTACAAA
TAB151       AATGGAAAACCGTTATAGAAATATATATGGAAGTGATGAATGAATGTAAAAGAGACGAGTGGGAAGAACATCGAGGAGATTTTTTACAAA
TAB152       AATGGAAAACCGTTATAGAAATATATATGGAAGTGATGAATGAATGTAAAAGAGACGAGTGGGAAGAACATCGAGGAGATTTTTTACAAA
SC52         AATGGAAAACCGTTATAGAAATATATATGGAAGTGATGAATGAATGTAAAAGAGACGAGTGGGAAGAACATCGAGGAGATTTTTTACAAA
SC55         AATGGAAAACCGTTATAGAAATATATATGGAAGTGATGAATGAATGTAAAAGAGACGAGTGGGAAGAACATCGAGGAGATTTTTTACAAA
SC03         AATGGAAAACCGTTATAGAAATATATATGGAAGTGATGAATGAATGTAAAAGAGACGAGTGGGAAGAACATCGAGGAGATTTTTTACAAA
SC53         AATGGAAAACCGTTATAGAAATATATATGGAAGTGATGAATGAATGTAAAAGAGACGAGTGGGAAGAACATCGAGGAGATTTTTTACAAA
TAB141       AATGGAAAACCGTTATAGAAATATATATGGAAGTGATGAATGAATGTAAAAGAGACGAGTGGGAAGAACATCGAGGAGATTTTTTACAAA
TAB123       AATGGAAAACCGTTATAGAAATATATATGGAAGTGATGAATGAATGTAAAAGAGACGAGTGGGAAGAACATCGAGGAGATTTTTTACAAA
AM1803       AATGGAAAACCGTTATAGAAATATATATGGAAGTGATGAATGAATGTAAAAGAGACGAGTGGGAAGAACATCGAGGAGATTTTTTACAAA
AM1814       AATGGAAAACCGTTATAGAAATATATATGGAAGTGATGAATGAATGTAAAAGAGACGAGTGGGAAGAACATCGAGGAGATTTTTTACAAA
AM1802       AATGGAAAACCGTTATAGAAATATATATGGAAGTGATGAATGAATGTAAAAGAGACGAGTGGGAAGAACATCGAGGAGATTTTTTACAAA
AM1811       AATGGAAAACCGTTATAGAAATATATATGGAAGTGATGAATGAATGTAAAAGAGACGAGTGGGAAGAACATCGAGGAGATTTTTTACAAA
SC50         AATGGAAAACCGTTATAGAAATATATATGGAAGTGATGAATGAATGTAAAAGAGACGAGTGGGAAGAACATCGAGGAGATTTTTTACAAA
SC56         AATGGAAAACCGTTATAGAAATATATATGGAAGTGATGAATGAATGTAAAAGAGACGAGTGGGAAGAACATCGAGGAGATTTTTTACAAA
TAB153       AATGGAAAACCGTTATAGAAATATATATGGAAGTGATGAATGAATGTAAAAGAGACGAGTGGGAAGAACATCGAGGAGATTTTTTACAAA
TAB154       AATGGAAAACCGTTATAGAAATATATATGGAAGTGATGAATGAATGTAAAAGAGACGAGTGGGAAGAACATCGAGTAGATTTTTTACAAA
TAB156       AATGGAAAACCGTTATAGAAATATATATGGAAGTGATGAATGAATGTAAAAGAGACGAGTGGGAAGAACATCGAGGAGATTTTTTACAAA
TAB136       AATGGAAAACCGTTATAGAAATATATATGGAAGTGATGAATGAATGTAAAAGAGACGAGTGGGAAGAACATCGAGGAGATTTTTTACAAA
AM1880       AATGGAAAACCGTTATAGAAATATATATGGAAGTGATGAATGAATGTAAAAGAGACGAGTGGGAAGAACATCGAGGAGATTTTTTACAAA
PF3D7_       AATGGAAAACCGTTATAGAAATATATATGGAAGTGATGAATGAATGTAAAAGAGACGAGTGGGAAGAACATCGAGGAGATTTTTTACAAA
PfSD01       AATGGAAAACCGTTATAGAAATATATATGGAAGTGATGAATGAATGTAAAAGAGACGAGTGGGAAGAACATCGAGTAGATTTTTTACAAA
Pf7G8        AATGGAAAACCGTTATAGAAATATATATGGAAGTGATGAATGAATGTAAAAGAGACGAGTGGGAAGAACATCGAGGAGATTTTTTACAAA
FCR3 GENE    AATGGAAAACCGTTATAGAAATATATATGGAAGTGATGAATGAATGTAAAAGAGACGAGTGGGAAGAACATCGAGGAGATTTTTTACAAA
MS822 GENE   ------------------------------------------------------------------------------------------
PfCD01_      AATGGAAAACCGTTATAGAAATATATATGGAAGTGATGAATGAATGTAAAAGAGACGAGTGGGAAGAACATCGAGGAGATTTTTTACAAA
PfKH01__     AATGGAAAACCGTTATAGAAATATATATGGAAGTGATGAATGAATGTAAAAGAGACGAGTGGGAAGAACATCGAGGAGATTTTTTACAAA
PfDd2__      AATGGAAAACCGTTATAGAAATATATATGGAAGTGATGAATGAATGTAAAAGAGACGAGTGGGAAGAACATCGAGGAGATTTTTTACAAA
PfSN01_      AATGGAAAACCGTTATAGAAATATATATGGAAGTGATGAATGAATGTAAAAGAGACGAGTGGGAAGAACATCGAGGAGATTTTTTACAAA
PfGN01       AATGGAAAACCGTTATAGAAATATATATGGAAGTGATGAATGAATGTAAAAGAGACGAGTGGGAAGAACATCGAGGAGATTTTTTACAAA
PfKE01       AATGGAAAACCGTTATAGAAATATATATGGAAGTGATGAATGAATGTAAAAGAGACGAGTGGGAAGAACATCGAGGAGATTTTTTACAAA
PfKH02       AATGGAAAACCGTTATAGAAATATATATGGAAGTGATGAATGAATGTAAAAGAGACGAGTGGGAAGAACATCGAGGAGATTTTTTACAAA
PfGB4        AATGGAAAACCGTTATAGAAATATATATGGAAGTGATGAATGAATGTAAAAGAGACGAGTGGGAAGAACATCGAGGAGATTTTTTACAAA
PfGA01_      AATGGAAAACCGTTATAGAAATATATATGGAAGTGATGAATGAATGTAAAAGAGACGAGTGGGAAGAACATCGAGGAGATTTTTTACAAA
PfHB3_       AATGGAAAACCGTTATAGAAATATATATGGAAGTGATGAATGAATGTAAAAGAGACGAGTGGGAAGAACATCGAGGAGATTTTTTACAAA
PfIT_        AATGGAAAACCGTTATAGAAATATATATGGAAGTGATGAATGAATGTAAAAGAGACGAGTGGGAAGAACATCGAGGAGATTTTTTACAAA
```

```
                  4060      4070      4080      4090      4100      4110      4120      4130      4140
             ....|....|....|....|....|....|....|....|....|....|....|....|....|....|....|....|....|....|
PF3D7 CDS    TCTGTTTAGAGGAATTTATTAAGAAAGATAAGGATGAAATGAGAAATATAAATGAAGAACTATATATCGAACAAAGTGATCATATGGAAG
MS822 mRNA   ------------------------------------------------------------------------------------------
FCR3 mRNA    TCTGTTTAGAGGAATTTATTAAGAAAGATAAGGATGAAATGAGAAATATAAATGAAGAACTATATATCGAACAAAGTGATCATATGGAAG
AM1898       TCTGTTTAGAGGAATTTATTAAGAAAGATAAGGATGAAATGAGAAATATAAATGAAGAACTATATATCGAACAAAGTGATCATATGGAAG
AA235        TCTGTTTAGAGGAATTTATTAAGAAAGATAAGGATGAAATGAGAAATATAAATGAAGAACTATATATCGAACAAAGTGATCATATGGAAG
AA243        TCTGTTTAGAGGAATTTATTAAGAAAGATAAGGATGAAATGAGAAATATAAATGAAGAACTATATATCGAACAAAGTGATCATATGGAAG
AA258        TCTGTTTAGAGGAATTTATTAAGAAAGATAAGGATGAAATGAGAAATATAAATGAAGAACTATATATCGAACAAAGTGATCATATGGAAG
AA264        TCTGTTTAGAGGAATTTATTAAGAAAGATAATGATGAAGTGAGAAATATAAATGACGAATTATATATCGAACAAAGTGATCATATGGAAG
AM1737       TCTGTTTAGAGGAATTTATTAAGAAAGATAAGGATGAAATGAGAAATATAAATGAAGAACTATATATCGAACAAAGTGATCATATGGAAG
SC74         TCTGTTTAGAGGAATTTATTAAGAAAGATAAGGATGAAATGAGAAATATAAATGAAGAACTATATATCGAACAAAGTGATCATATGGAAG
TAB166       TCTGTTTAGAGGAATTTATTAAGAAAGATAAGGATGAAATGAGAAATATAAATGAAGAACTATATATCGAACAAAGTGATCATATGGAAG
TAB138       TCTGTTTAGAGGAATTTATTAAGAAAGATAAGGATGAAATGAGAAATATAAATGAAGAACTATATATCGAACAAAGTGATCATATGGAAG
TAB130       TCTGTTTAGAGGAATTTATTAAGAAAGATAAGGATGAAATGAGAAATATAAATGAAGAACTATATATCGAACAAAGTGATCATATGGAAG
TAB131       TCTGTTTAGAGGAATTTATTAAGAAAGATAAGGATGAAATGAGAAATATAAATGAAGAACTATATATCGAACAAAGTGATCATATGGAAG
TAB151       TCTGTTTAGAGGAATTTATTAAGAAAGATAAGGATGAAATGAGAAATATAAATGAAGAACTATATATCGAACAAAGTGATCATATGGAAG
TAB152       TCTGTTTAGAGGAATTTATTAAGAAAGATAAGGATGAAATGAGAAATATAAATGAAGAACTATATATCGAACAAAGTGATCATATGGAAG
SC52         TCTGTTTAGAGGAATTTATTAAGAAAGATAAGGATGAAATGAGAAATATAAATGAAGAACTATATATCGAACAAAGTGATCATATGGAAG
SC55         TCTGTTTAGAGGAATTTATTAAGAAAGATAAGGATGAAATGAGAAATATAAATGAAGAACTATATATCGAACAAAGTGATCATATGGAAG
SC03         TCTGTTTAGAGGAATTTATTAAGAAAGATAAGGATGAAATGAGAAATATAAATGAAGAACTATATATCGAACAAAGTGATCATATGGAAG
SC53         TCTGTTTAGAGGAATTTATTAAGAAAGATAAGGATGAAATGAGAAATATAAATGAAGAACTATATATCGAACAAAGTGATCATATGGAAG
TAB141       TCTGTTTAGAGGAATTTATTAAGAAAGATAAGGATGAAATGAGAAATATAAATGAAGAACTATATATCGAACAAAGTGATCATATGGAAG
TAB123       TCTGTTTAGAGGAATTTATTAAGAAAGATAAGGATGAAATGAGAAATATAAATGAAGAACTATATATCGAACAAAGTGATCATATGGAAG
AM1803       TCTGTTTAGAGGAATTTATTAAGAAAGATAATGATGAAGTGAGAAATATAAATGACGAATTATATATCGAACAAAGTGATCATATGGAAG
AM1814       TCTGTTTAGAGGAATTTATTAAGAAAGATAAGGATGAAATGAGAAATATAAATGAAGAACTATATATCGAACAAAGTGATCATATGGAAG
AM1802       TCTGTTTAGAGGAATTTATTAAGAAAGATAAGGATGAAATGAGAAATATAAATGAAGAACTATATATCGAACAAAGTGATCATATGGAAG
AM1811       TCTGTTTAGAGGAATTTATTAAGAAAGATAATGATGAAGTGAGAAATATAAATGACGAATTATATATCGAACAAAGTGATCATATGGAAG
SC50         TCTGTTTAGAGGAATTTATTAAGAAAGATAAGGATGAAATGAGAAATATAAATGAAGAACTATATATCGAACAAAGTGATCATATGGAAG
SC56         TCTGTTTAGAGGAATTTATTAAGAAAGATAATGATGAAGTGAGAAATATAAATGACGAATTATATATCGAACAAAGTGATCATATGGAAG
TAB153       TCTGTTTAGAGGAATTTATTAAGAAAGATAAGGATGAAATGAGAAATATAAATGAAGAACTATATATCGAACAAAGTGATCATATGGAAG
TAB154       TCTGTTTAGAGGAATTTATTAAGAAAGATAAGGATGAAATGAGAAATATAAATGAAGAACTATATATCGAACAAAGTGATCATATGGAAG
TAB156       TCTGTTTAGAGGAATTTATTAAGAAAGATAAGGATGAAATGAGAAATATAAATGAAGAACTATATATCGAACAAAGTGATCATATGGAAG
TAB136       TCTGTTTAGAGGAATTTATTAAGAAAGATAAGGATGAAATGAGAAATATAAATGAAGAACTATATATCGAACAAAGTGATCATATGGAAG
AM1880       TCTGTTTAGAGGAATTTATTAAGAAAGATAAGGATGAAATGAGAAATATAAATGAAGAACTATATATCGAACAAAGTGATCATATGGAAG
PF3D7_       TCTGTTTAGAGGAATTTATTAAGAAAGATAAGGATGAAATGAGAAATATAAATGAAGAACTATATATCGAACAAAGTGATCATATGGAAG
PfSD01       TCTGTTTAGAGGAATTTATTAAGAAAGATAAGGATGAAATGAGAAATATAAATGAAGAACTATATATCGAACAAAGTGATCATATGGAAG
Pf7G8        TCTGTTTAGAGGAATTTATTAAGAAAGATAAGGATGAAATGAGAAATATAAATGAAGAACTATATATCGAACAAAGTGATCATATGGAAG
FCR3 GENE    TCTGTTTAGAGGAATTTATTAAGAAAGATAAGGATGAAATGAGAAATATAAATGAAGAACTATATATCGAACAAAGTGATCATATGGAAG
MS822 GENE   ------------------------------------------------------------------------------------------
PfCD01_      TCTGTTTAGAGGAATTTATTAAGAAAGATAAGGATGAAATGAGAAATATAAATGAAGAACTATATATCGAACAAAGTGATCATATGGAAG
PfKH01__     TCTGTTTAGAGGAATTTATTAAGAAAGATAAGGATGAAATGAGAAATATAAATGAAGAACTATATATCGAACAAAGTGATCATATGGAAG
PfDd2__      TCTGTTTAGAGGAATTTATTAAGAAAGATAAGGATGAAATGAGAAATATAAATGAAGAACTATATATCGAACAAAGTGATCATATGGAAG
PfSN01_      TCTGTTTAGAGGAATTTATTAAGAAAGATAAGGATGAAATGAGAAATATAAATGAAGAACTATATATCGAACAAAGTGATCATATGGAAG
PfGN01       TCTGTTTAGAGGAATTTATTAAGAAAGATAAGGATGAAATGAGAAATATAAATGAAGAACTATATATCGAACAAAGTGATCATATGGAAG
PfKE01       TCTGTTTAGAGGAATTTATTAAGAAAGATAAGGATGAAATGAGAAATATAAATGAAGAACTATATATCGAACAAAGTGATCATATGGAAG
PfKH02       TCTGTTTAGAGGAATTTATTAAGAAAGATAAGGATGAAATGAGAAATATAAATGAAGAACTATATATCGAACAAAGTGATCATATGGAAG
PfGB4        TCTGTTTAGAGGAATTTATTAAGAAAGATAAGGATGAAATGAGAAATATAAATGAAGAACTATATATCGAACAAAGTGATCATATGGAAG
PfGA01_      TCTGTTTAGAGGAATTTATTAAGAAAGATAAGGATGAAATGAGAAATATAAATGAAGAACTATATATCGAACAAAGTGATCATATGGAAG
PfHB3_       TCTGTTTAGAGGAATTTATTAAGAAAGATAAGGATGAAATGAGAAATATAAATGAAGAACTATATATCGAACAAAGTGATCATATGGAAG
PfIT_        TCTGTTTAGAGGAATTTATTAAGAAAGATAAGGATGAAATGAGAAATATAAATGAAGAACTATATATCGAACAAAGTGATCATATGGAAG
```

```
                       4150      4160      4170      4180      4190      4200      4210      4220      4230
               ....|....|....|....|....|....|....|....|....|....|....|....|....|....|....|....|....|....|
PF3D7 CDS      ATATGCTTATGTTAGAGAGACAAAAAATTATATGGCTCCAATGGATTAATCGAAATAAATATATGTTAGAAAAGTGGAATAAGGAAGAAT
MS822 mRNA     ------------------------------------------------------------------------------------------
FCR3 mRNA      ATATGCTTATGTTAGAGAGACAAAAAATTATATGGCTCCAATGGATTAATCGAAATAAATATATGTTAGAAAAGTGGAATAAGGAAGAAT
AM1898         ATATGCTTATGTTAGAGAGACAAAAAATTATATGGCTCCAATGGATTAATCGAAATAAATATATGTTAGAAAAGTGGAATAAGGAAGAAT
AA235          ATATGCTTATGTTAGAGAGACAAAAAATTATATGGCTCCAATGGATTAATCGAAATAAATATATGTTAGAAAAGTGGAATAAGGAAGAAT
AA243          ATATGCTTATGTTAGAGAGACAAAAAATTATATGGCTCCAATGGATTAATCGAAATAAATATATGTTAGAAAAGTGGAATAAGGAAGAAT
AA258          ATATGCTTATGTTAGAGAGACAAAAAATTATATGGCTCCAATGGATTAATCGAAATAAATATATGTTAGAAAAGTGGAATAAGGAAGAAT
AA264          ATACGCTTATGTTAGAGAGACAAAAAATTATATGGCTTCAATGGATTAAACGAAATAAATATATGTTAGAAAAGTGGAATAAGGAAGAAT
AM1737         ATATGCTTATGTTAGAGAGACAAAAAATTATATGGCTCCAATGGATTAATCGAAATAAATATATGTTAGAAAAGTGGAATAAGGAAGAAT
SC74           ATATGCTTATGTTAGAGAGACAAAAAATTATATGGCTCCAATGGATTAATCGAAATAAATATATGTTAGAAAAGTGGAATAAGGAAGAAT
TAB166         ATATGCTTATGTTAGAGAGACAAAAAATTATATGGCTCCAATGGATTAATCGAAATAAATATATGTTAGAAAAGTGGAATAAGGAAGAAT
TAB138         ATATGCTTATGTTAGAGAGACAAAAAATTATATGGCTCCAATGGATTAATCGAAATAAATATATGTTAGAAAAGTGGAATAAGGAAGAAT
TAB130         ATATGCTTATGTTAGAGAGACAAAAAATTATATGGCTCCAATGGATTAATCGAAATAAATATATGTTAGAAAAGTGGAATAAGGAAGAAT
TAB131         ATATGCTTATGTTAGAGAGACAAAAAATTATATGGCTCCAATGGATTAATCGAAATAAATATATGTTAGAAAAGTGGAATAAGGAAGAAT
TAB151         ATATGCTTATGTTAGAGAGACAAAAAATTATATGGCTCCAATGGATTAATCGAAATAAATATATGTTAGAAAAGTGGAATAAGGAAGAAT
TAB152         ATATGCTTATGTTAGAGAGACAAAAAATTATATGGCTCCAATGGATTAATCGAAATAAATATATGTTAGAAAAGTGGAATAAGGAAGAAT
SC52           ATATGCTTATGTTAGAGAGACAAAAAATTATATGGCTCCAATGGATTAATCGAAATAAATATATGTTAGAAAAGTGGAATAAGGAAGAAT
SC55           ATATGCTTATGTTAGAGAGACAAAAAATTATATGGCTCCAATGGATTAATCGAAATAAATATATGTTAGAAAAGTGGAATAAGGAAGAAT
SC03           ATATGCTTATGTTAGAGAGACAAAAAATTATATGGCTCCAATGGATTAAACGAAATAAATATATGTTAGAAAAGTGGAATAAGGAAGAAT
SC53           ATATGCTTATGTTAGAGAGACAAAAAATTATATGGCTCCAATGGATTAATCGAAATAAATATATGTTAGAAAAGTGGAATAAGGAAGAAT
TAB141         ATATGCTTATGTTAGAGAGACAAAAAATTATATGGCTCCAATGGATTAATCGAAATAAATATATGTTAGAAAAGTGGAATAAGGAAGAAT
TAB123         ATATGCTTATGTTAGAGAGACAAAAAATTATATGGCTCCAATGGATTAATCGAAATAAATATATGTTAGAAAAGTGGAATAAGGAAGAAT
AM1803         ATACGCTTATGTTAGAGAGACAAAAAATTATATGGCTTCAATGGATTAAACGAAATAAATATATGTTAGAAAAGTGGAATAAGGAAGAAT
AM1814         ATATGCTTATGTTAGAGAGACAAAAAATTATATGGCTCCAATGGATTAATCGAAATAAATATATGTTAGAAAAGTGGAATAAGGAAGAAT
AM1802         ATATGCTTATGTTAGAGAGACAAAAAATTATATGGCTCCAATGGATTAATCGAAATAAATATATGTTAGAAAAGTGGAATAAGGAAGAAT
AM1811         ATACGCTTATGTTAGAGAGACAAAAAATTATATGGCTTCAATGGATTAAACGAAATAAATATATGTTAGAAAAGTGGAATAAGGAAGAAT
SC50           ATATGCTTATGTTAGAGAGACAAAAAATTATATGGCTCCAATGGATTAATCGAAATAAATATATGTTAGAAAAGTGGAATAAGGAAGAAT
SC56           ATACGCTTATGTTAGAGAGACAAAAAATTATATGGCTTCAATGGATTAAACGAAATAAATATATGTTAGAAAAGTGGAATAAGGAAGAAT
TAB153         ATATGCTTATGTTAGAGAGACAAAAAATTATATGGCTCCAATGGATTAATCGAAATAAATATATGTTAGAAAAGTGGAATAAGGAAGAAT
TAB154         ATATGCTTATGTTAGAGAGACAAAAAATTATATGGCTCCAATGGATTAATCGAAATAAATATATGTTAGAAAAGTGGAATAAGGAAGAAT
TAB156         ATATGCTTATGTTAGAGAGACAAAAAATTATATGGCTCCAATGGATTAATCGAAATAAATATATGTTAGAAAAGTGGAATAAGGAAGAAT
TAB136         ATATGCTTATGTTAGAGAGACAAAAAATTATATGGCTCCAATGGATTAATCGAAATAAATATATGTTAGAAAAGTGGAATAAGGAAGAAT
AM1880         ATATGCTTATGTTAGAGAGACAAAAAATTATATGGCTCCAATGGATTAATCGAAATAAATATATGTTAGAAAAGTGGAATAAGGAAGAAT
PF3D7_         ATATGCTTATGTTAGAGAGACAAAAAATTATATGGCTCCAATGGATTAATCGAAATAAATATATGTTAGAAAAGTGGAATAAGGAAGAAT
PfSD01         ATATGCTTATGTTAGAGAGACAAAAAATTATATGGCTCCAATGGATTAATCGAAATAAATATATGTTAGAAAAGTGGAATAAGGAAGAAT
Pf7G8          ATATGCTTATGTTAGAGAGACAAAAAATTATATGGCTCCAATGGATTAATCGAAATAAATATATGTTAGAAAAGTGGAATAAGGAAGAAT
FCR3 GENE      ATATGCTTATGTTAGAGAGACAAAAAATTATATGGCTCCAATGGATTAATCGAAATAAATATATGTTAGAAAAGTGGAATAAGGAAGAAT
MS822 GENE     ------------------------------------------------------------------------------------------
PfCD01_        ATATGCTTATGTTAGAGAGACAAAAAATTATATGGCTTCAATGGATTAAACGAAATAAATATATGTTAGAAAAGTGGAATAAGGAAGAAT
PfKH01__       ATATGCTTATGTTAGAGAGACAAAAAATTATATGGCTCCAATGGATTAATCGAAATAAATATATGTTAGAAAAGTGGAATAAGGAAGAAT
PfDd2__        ATATGCTTATGTTAGAGAGACAAAAAATTATATGGCTCCAATGGATTAATCGAAATAAATATATGTTAGAAAAGTGGAATAAGGAAGAAT
PfSN01_        ATATGCTTATGTTAGAGAGACAAAAAATTATATGGCTCCAATGGATTAATCGAAATAAATATATGTTAGAAAAGTGGAATAAGGAAGAAT
PfGN01         ATATGCTTATGTTAGAGAGACAAAAAATTATATGGCTCCAATGGATTAATCGAAATAAATATATGTTAGAAAAGTGGAATAAGGAAGAAT
PfKE01         ATATGCTTATGTTAGAGAGACAAAAAATTATATGGCTCCAATGGATTAATCGAAATAAATATATGTTAGAAAAGTGGAATAAGGAAGAAT
PfKH02         ATATGCTTATGTTAGAGAGACAAAAAATTATATGGCTCCAATGGATTAATCGAAATAAATATATGTTAGAAAAGTGGAATAAGGAAGAAT
PfGB4          ATATGCTTATGTTAGAGAGACAAAAAATTATATGGCTCCAATGGATTAATCGAAATAAATATATGTTAGAAAAGTGGAATAAGGAAGAAT
PfGA01_        ATATGCTTATGTTAGAGAGACAAAAAATTATATGGCTCCAATGGATTAATCGAAATAAATATATGTTAGAAAAGTGGAATAAGGAAGAAT
PfHB3_         ATATGCTTATGTTAGAGAGACAAAAAATTATATGGCTCCAATGGATTAATCGAAATAAATATATGTTAGAAAAGTGGAATAAGGAAGAAT
PfIT_          ATATGCTTATGTTAGAGAGACAAAAAATTATATGGCTCCAATGGATTAATCGAAATAAATATATGTTAGAAAAGTGGAATAAGGAAGAAT
```

```
                4240      4250      4260      4270      4280      4290      4300      4310      4320
            ....|....|....|....|....|....|....|....|....|....|....|....|....|....|....|....|....|....|
PF3D7 CDS   GGTTTCATAAATTAAAAAAGGATTGGGAAGATGAAAAGAATAGATATGACGAGAAATTATTTCTTTGGAAAAATACAGAAAATGATAAAT
MS822 mRNA  ------------------------------------------------------------------------------------------
FCR3 mRNA   GGTTTCATAAATTAAAAAAGGATTGGGAAGATGAAAAGAATAGATATGACGAGAAATTATTTCTTTGGAAAAATACAGAAAATGATAAAT
AM1898      GGTTTCATAAATTAAAAAAGGATTGGGAAGATGAAAAGAATAGATATGACGAGAAATTATTTCTTTGGAAAAATACAGAAAATGATAAAT
AA235       GGTTTTGTAAATTAAAAAAGGATTGGGAAGATGAAAAGAATAGATATGACGAGAAATTATTTCTTTGGAAAAATACAGAAAATGATAAAT
AA243       GGTTTCATAAATTAAAAAAGGATTGGGAAGATGAAAAGAATAGATATGACGAGAAATTATTTCTTTGGAAAAATACAGAAAATGATAAAT
AA258       GGTTTCATAAATTAAAAAAGGATTGGGAAGATGAAAAGAATAGATATGACGAGAAATTATTTCTTTGGAAAAATACAGAAAATGATAAAT
AA264       GGTTTCATAAATTAAAAAAGGATTGGGAAGATGAAAAGAATAGATATGACGAGAAATTATTTCTTTGGAAAAATACAGAAAATGATAAAT
AM1737      GGTTTTGTAAATTAAAAAAGGATTGGGAAGATGAAAAAAATAGATATGACGAGAAATTATTTCTTTGCAAAAATACAGAAAATGATAAAT
SC74        GGTTTTGTAAATTAAAAAAGGATTGGGAAGATGAAAAAAATAGATATGACGAGAAATTATTTCTTTGCAAAAATACAGAAAATGATAAAT
TAB166      GGTTTCATAAATTAAAAAAGGATTGGGAAGATGAAAAGAATAGATATGACGAGAAATTATTTCTTTGGAAAAATACAGAAAATGATAAAT
TAB138      GGTTTTGTAAATTAAAAAAGGATTGGGAAGATGAAAAAAATAGATATGACGAGAAATTATTTCTTTGCAAAAATACAGAAAATGATAAAT
TAB130      GGTTTTGTAAATTAAAAAAGGATTGGGAAGATGAAAAGAATAGATATGACGAGAAATTATTTCTTTGGAAAAATACAGAAAATGATAAAT
TAB131      GGTTTTGTAAATTAAAAAAGGATTGGGAAGATGAAAAAAATAGATATGACGAGAAATTATTTCTTTGCAAAAATACAGAAAATGATAAAT
TAB151      GGTTTTGTAAATTAAAAAAGGATTGGGAAGATGAAAAAAATAGATATGACGAGAAATTATTTCTTTGCAAAAATACAGAAAATGATAAAT
TAB152      CGTTTTGTAAATTAAAAAAGGATTGGGAAGATGAAAAAAATAGATATGACGAGAAATTATTTCTTTGCAAAAATACAGAAAATGATAAAT
SC52        GGTTTCATAAATTAAAAAAGGATTGGGAAGATGAAAAGAATAGATATGACGAGAAATTATTTCTTTGGAAAAATACAGAAAATGATAAAT
SC55        GGTTTCATAAATTAAAAAAGGATTGGGAAGATGAAAAGAATAGATATGACGAGAAATTATTTCTTTGGAAAAATACAGAAAATGATAAAT
SC03        GGTTTTGTAAATTAAAAAAGGATTGGGAAGATGAAAAAAATAGATATGACGAGAAATTATTTCTTTGCAAAAATACAGAAAATGATAAAT
SC53        CGTTTTGTAAATTAAAAAAGGATTGGGAAGATGAAAAAAATAGATATGACGAGAAATTATTTCTTTGCAAAAATACAGAAAATGATAAAT
TAB141      GGTTTTGTAAATTAAAAAAGGATTGGGAAGATGAAAAAAATAGATATGACGAGAAATTATTTCTTTGCAAAAATACAGAAAATGATAAAT
TAB123      GGTTTCATAAATTAAAAAAGGATTGGGAAGATGAAAAGAATAGATATGACGAGAAATTATTTCTTTGGAAAAATACAGAAAATGATAAAT
AM1803      GGTTTTGTAAATTAAAAAAGGATTGGGAAGATGAAAAAAATAGATATGACGAGAAATTATTTCTTTGCAAAAATACAGAAAATGATAAAT
AM1814      GGTTTCATAAATTAAAAAAGGATTGGGAAGATGAAAAGAATAGATATGACGAGAAATTATTTCTTTGGAAAAATACAGAAAATGATAAAT
AM1802      GGTTTCATAAATTAAAAAAGGATTGGGAAGATGAAAAGAATAGATATGACGAGAAATTATTTCTTTGGAAAAATACAGAAAATGATAAAT
AM1811      GGTTTTGTAAATTAAAAAAGGATTGGGAAGATGAAAAAAATAGATATGACGAGAAATTATTTCTTTGCAAAAATACAGAAAATGATAAAT
SC50        GGTTTCATAAATTAAAAAAGGATTGGGAAGATGAAAAGAATAGATATGACGAGAAATTATTTCTTTGGAAAAATACAGAAAATGATAAAT
SC56        GGTTTTGTAAATTAAAAAAGGATTGGGAAGATGAAAAAAATAGATATGACGAGAAATTATTTCTTTGCAAAAATACAGAAAATGATAAAT
TAB153      GGTTTTGTAAATTAAAAAAGGATTGGGAAGATGAAAAAAATAGATATGACGAGAAATTATTTCTTTGCAAAAATACAGAAAATGATAAAT
TAB154      GGTTTTGTAAATTAAAAAAGGATTGGGAAGATGAAAAGAATAGATATGACGAGAAATTATTTCTTTGGAAAAATACAGAAAATGATAAAT
TAB156      GGTTTCATAAATTAAAAAAGGATTGGGAAGATGAAAAGAATAGATATGACGAGAAATTATTTCTTTGGAAAAATACAGAAAATGATAAAT
TAB136      GGTTTTGTAAATTAAAAAAGGATTGGGAAGATGAAAAAAATAGATATGACGAGAAATTATTTCTTTGCAAAAATACAGAAAATGATAAAT
AM1880      GGTTTCATAAATTAAAAAAGGATTGGGAAGATGAAAAGAATAGATATGACGAGAAATTATTTCTTTGGAAAAATACAGAAAATGATAAAT
PF3D7_      GGTTTCATAAATTAAAAAAGGATTGGGAAGATGAAAAGAATAGATATGACGAGAAATTATTTCTTTGGAAAAATACAGAAAATGATAAAT
PfSD01      GGTTTTGTAAATTAAAAAAGGATTGGGAAGATGAAAAAAATAGATATGACGAGAAATTATTTCTTTGCAAAAATACAGAAAATGATAAAT
Pf7G8       GGTTTCATAAATTAAAAAAGGATTGGGAAGATGAAAAAAATAGATATGACGAGAAATTATTTCTTTGCAAAAATACAGAAAATGATAAAT
FCR3 GENE   GGTTTCATAAATTAAAAAAGGATTGGGAAGATGAAAAGAATAGATATGACGAGAAATTATTTCTTTGGAAAAATACAGAAAATGATAAAT
MS822 GENE  ------------------------------------------------------------------------------------------
PfCD01_     GGTTTTGTAAATTAAAAAAGGATTGGGAAGATGAAAAAAATAGATATGACGAGAAATTATTTCTTTGCAAAAATACAGAAAATGATAAAT
PfKH01__    GGTTTTGTAAATTAAAAAAGGATTGGGAAGATGAAAAAAATAGATATGACGAGAAATTATTTCTTTGCAAAAATACAGAAAATGATAAAT
PfDd2__     GGTTTTGTAAATTAAAAAAGGATTGGGAAGATGAAAAGAATAGATATGACGAGAAATTATTTCTTTGGAAAAATACAGAAAATGATAAAT
PfSN01_     GGTTTCATAAATTAAAAAAGGATTGGGAAGATGAAAAAAATAGATATGACGAGAAATTATTTCTTTGCAAAAATACAGAAAATGATAAAT
PfGN01      GGTTTTGTAAATTAAAAAAGGATTGGGAAGATGAAAAAAATAGATATGACGAGAAATTATTTCTTTGCAAAAATACAGAAAATGATAAAT
PfKE01      GGTTTTGTAAATTAAAAAAGGATTGGGAAGATGAAAAAAATAGATATGACGAGAAATTATTTCTTTGCAAAAATACAGAAAATGATAAAT
PfKH02      GGTTTTGTAAATTAAAAAAGGATTGGGAAGATGAAAAAAATAGATATGACGAGAAATTATTTCTTTGCAAAAATACAGAAAATGATAAAT
PfGB4       GGTTTTGTAAATTAAAAAAGGATTGGGAAGATGAAAAAAATAGATATGACGAGAAATTATTTCTTTGCAAAAATACAGAAAATGATAAAT
PfGA01_     GGTTTTGTAAATTAAAAAAGGATTGGGAAGATGAAAAAAATAGATATGACGAGAAATTATTTCTTTGCAAAAATACAGAAAATGATAAAT
PfHB3_      GGTTTTGTAAATTAAAAAAGGATTGGGAAGATGAAAAAAATAGATATGACGAGAAATTATTTCTTTGCAAAAATACAGAAAATGATAAAT
PfIT_       GGTTTCATAAATTAAAAAAGGATTGGGAAGATGAAAAGAATAGATATGACGAGAAATTATTTCTTTGGAAAAATACAGAAAATGATAAAT
```

```
                4330      4340      4350      4360      4370      4380      4390      4400      4410
            ....|....|....|....|....|....|....|....|....|....|....|....|....|....|....|....|....|....|
PF3D7 CDS   ATATAAATCCGATGTTAGAAAGACAAAAATATATATGGAGAAAATGGATTGCAAAACATTTATATCATATTGATGAGTGGGAAAATGAAG
MS822 mRNA  ------------------------------------------------------------------------------------------
FCR3 mRNA    ATATAAATCCGATGTTAGAAAGACAAAAATATATATGGAGAAAATGGATTGCAAAACATTTATATCATATTGATGAGTGGGAAAATGAAG
AM1898      ATATAAATCCGATGTTAGAAAGACAAAAATATATATGGAGAAAATGGATTGCAAAACATTTATATCATATTGATGAGTGGGAAAATGAAG
AA235       ATATAAATCCGATGTTAGAAAGACAAAAATATATATGGAGAAAATGGATTGCAAAACATTTATATCATATTGATGAGTGGGAAAATGAAG
AA243       ATATAAATCCGATGTTAGAAAGACAAAAATATATATGGAGAAAATGGATTGCAAAACATTTATATCATATTGATGAGTGGGAAAATGAAG
AA258       ATATAAATCCGATGTTAGAAAGACAAAAATATATATGGAGAAAATGGATTGCAAAACATTTATATCATATTGATGAGTGGGAAAATGAAG
AA264       ATATAAATCCGATGTTAGAAAGACAAAAATATATATGGAGAAAATGGATTGCAAAACATTTATATCATATTGATGAGTGGGAAAATGAAG
AM1737      ATATAAATCCAATGTTAGAAAGACAAAAATATATATGGAGAAAATGGATTGCAAAACATTTATATCATATTGATGAGTGGGAAAATGAAG
SC74        ATATAAATCCAATGTTAGAAAGACAAAAATATATATGGAGAAAATGGATTGCAAAACATTTATATCATATTGATGAGTGGGAAAATGAAG
TAB166      ATATAAATCCGATGTTAGAAAGACAAAAATATATATGGAGAAAATGGATTGCAAAACATTTATATCATATTGATGAGTGGGAAAATGAAG
TAB138      ATATAAATCCAATGTTAGAAAGACAAAAATATATATGGAGAAAATGGATTGCAAAACATTTATATCATATTGATGAGTGGGAAAATGAAG
TAB130      ATATAAATCCGATGTTAGAAAGACAAAAATATATATGGAGAAAATGGATTGCAAAACATTTATATCATATTGATGAGTGGGAAAATGAAG
TAB131      ATATAAATCCAATGTTAGAAAGACAAAAATATATATGGAGAAAATGGATTGCAAAACATTTATATCATATTGATGAGTGGGAAAATGAAG
TAB151      ATATAAATCCAATGTTAGAAAGACAAAAATATATATGGAGAAAATGGATTGCAAAACATTTATATCATATTGATGAGTGGGAAAATGAAG
TAB152      ATATAAATCCAATGTTAGAAAGACAAAAATATATATGGAGAAAATGGATTGCAAAACATTTATATCATATTGATGAGTGGGAAAATGAAG
SC52        ATATAAATCCGATGTTAGAAAGACAAAAATATATATGGAGAAAATGGATTGCAAAACATTTATATCATATTGATGAGTGGGAAAATGAAG
SC55        ATATAAATCCGATGTTAGAAAGACAAAAATATATATGGAGAAAATGGATTGCAAAACATTTATATCATATTGATGAGTGGGAAAATGAAG
SC03        ATATAAATCCAATGTTAGAAAGACAAAAATATATATGGAGAAAATGGATTGCAAAACATTTATATCATATTGATGAGTGGGAAAATGAAG
SC53        ATATAAATCCAATGTTAGAAAGACAAAAATATATATGGAGAAAATGGATTGCAAAACATTTATATCATATTGATGAGTGGGAAAATGAAG
TAB141      ATATAAATCCAATGTTAGAAAGACAAAAATATATATGGAGAAAATGGATTGCAAAACATTTATATCATATTGATGAGTGGGAAAATGAAG
TAB123      ATATAAATCCGATGTTAGAAAGACAAAAATATATATGGAGAAAATGGATTGCAAAACATTTATATCATATTGATGAGTGGGAAAATGAAG
AM1803      ATATAAATCCAATGTTAGAAAGACAAAAATATATATGGAGAAAATGGATTGCAAAACATTTATATCATATTGATGAGTGGGAAAATGAAG
AM1814      ATATAAATCCGATGTTAGAAAGACAAAAATATATATGGAGAAAATGGATTGCAAAACATTTATATCATATTGATGAGTGGGAAAATGAAG
AM1802      ATATAAATCCGATGTTAGAAAGACAAAAATATATATGGAGAAAATGGATTGCAAAACATTTATATCATATTGATGAGTGGGAAAATGAAG
AM1811      ATATAAATCCAATGTTAGAAAGACAAAAATATATATGGAGAAAATGGATTGCAAAACATTTATATCATATTGATGAGTGGGAAAATGAAG
SC50        ATATAAATCCGATGTTAGAAAGACAAAAATATATATGGAGAAAATGGATTGCAAAACATTTATATCATATTGATGAGTGGGAAAATGAAG
SC56        ATATAAATCCAATGTTAGAAAGACAAAAATATATATGGAGAAAATGGATTGCAAAACATTTATATCATATTGATGAGTGGGAAAATGAAG
TAB153      ATATAAATCCAATGTTAGAAAGACAAAAATATATATGGAGAAAATGGATTGCAAAACATTTATATCATATTGATGAGTGGGAAAATGAAG
TAB154      ATATAAATCCGATGTTAGAAAGACAAAAATATATATGGAGAAAATGGATTGCAAAACATTTATATCATATTGATGAGTGGGAAAATGAAG
TAB156      ATATAAATCCGATGTTAGAAAGACAAAAATATATATGGAGAAAATGGATTGCAAAACATTTATATCATATTGATGAGTGGGAAAATGAAG
TAB136      ATATAAATCCAATGTTAGAAAGACAAAAATATATATGGAGAAAATGGATTGCAAAACATTTATATCATATTGATGAGTGGGAAAATGAAG
AM1880      ATATAAATCCGATGTTAGAAAGACAAAAATATATATGGAGAAAATGGATTGCAAAACATTTATATCATATTGATGAGTGGGAAAATGAAG
PF3D7_      ATATAAATCCGATGTTAGAAAGACAAAAATATATATGGAGAAAATGGATTGCAAAACATTTATATCATATTGATGAGTGGGAAAATGAAG
PfSD01      ATATAAATCCAATGTTAGAAAGACAAAAATATATATGGAGAAAATGGATTGCAAAACATTTATATCATATTGATGAGTGGGAAAATGAAG
Pf7G8       ATATAAATCCAATGTTAGAAAGACAAAAATATATATGGAGAAAATGGATTGCAAAACATTTATATCATATTGATGAGTGGGAAAATGAAG
FCR3 GENE   ATATAAATCCGATGTTAGAAAGACAAAAATATATATGGAGAAAATGGATTGCAAAACATTTATATCATATTGATGAGTGGGAAAATGAAG
MS822 GENE  ------------------------------------------------------------------------------------------
PfCD01_     ATATAAATCCAATGTTAGAAAGACAAAAATATATATGGAGAAAATGGATTGCAAAACATTTATATCATATTGATGAGTGGGAAAATGAAG
PfKH01__    ATATAAATCCAATGTTAGAAAGACAAAAATATATATGGAGAAAATGGATTGCAAAACATTTATATCATATTGATGAGTGGGAAAATGAAG
PfDd2__     ATATAAATCCGATGTTAGAAAGACAAAAATATATATGGAGAAAATGGATTGCAAAACATTTATATCATATTGATGAGTGGGAAAATGAAG
PfSN01_     ATATAAATCCAATGTTAGAAAGACAAAAATATATATGGAGAAAATGGATTGCAAAACATTTATATCATATTGATGAGTGGGAAAATGAAG
PfGN01      ATATAAATCCAATGTTAGAAAGACAAAAATATATATGGAGAAAATGGATTGCAAAACATTTATATCATATTGATGAGTGGGAAAATGAAG
PfKE01      ATATAAATCCAATGTTAGAAAGACAAAAATATATATGGAGAAAATGGATTGCAAAACATTTATATCATATTGATGAGTGGGAAAATGAAG
PfKH02      ATATAAATCCAATGTTAGAAAGACAAAAATATATATGGAGAAAATGGATTGCAAAACATTTATATCATATTGATGAGTGGGAAAATGAAG
PfGB4       ATATAAATCCAATGTTAGAAAGACAAAAATATATATGGAGAAAATGGATTGCAAAACATTTATATCATATTGATGAGTGGGAAAATGAAG
PfGA01_     ATATAAATCCAATGTTAGAAAGACAAAAATATATATGGAGAAAATGGATTGCAAAACATTTATATCATATTGATGAGTGGGAAAATGAAG
PfHB3_      ATATAAATCCAATGTTAGAAAGACAAAAATATATATGGAGAAAATGGATTGCAAAACATTTATATCATATTGATGAGTGGGAAAATGAAG
PfIT_       ATATAAATCCGATGTTAGAAAGACAAAAATATATATGGAGAAAATGGATTGCAAAACATTTATATCATATTGATGAGTGGGAAAATGAAG
```

```
                     4420      4430      4440      4450      4460      4470      4480      4490      4500
             ....|....|....|....|....|....|....|....|....|....|....|....|....|....|....|....|....|....|
PF3D7 CDS    AATGGTTTAAATTGTTAATGAGTAAATATGAAAAAGATAGAGATGATTTGAAAAGAGATCATCCAATTAACAAAATGGATGATATGGAAA
MS822 mRNA   ------------------------------------------------------------------------------------------
FCR3 mRNA    AATGGTTTAAATTGTTAATGAGTAAATATGAAAAAGATAGAGATGATTTGAAAAGAGATCATCCAATTAACAAAATGGATGATATGGAAA
AM1898       AATGGTTTAAATTGTTAATGAGTAAATATGAAAAAGATAGAGATGATTTGAAAAGAGATCATCCAATTAACAAAATGGATGATATGGAAA
AA235        AATGGTTTAAATTGTTAATGAGTAAATATGAAAAAGATAGAGATGATTTGAAAAGAGATCATCCAATTAACAAAATGGATGATATGGAAA
AA243        AATGGTTTAAATTGTTAATGAGTAAATATGAAAAAGATAGAGATGATTTGAAAAGAGATCATCCAATTAACAAAATGGATGATATGGAAA
AA258        AATGGTTTAAATTGTTAATGAGTAAATATGAAAAAGATAGAGATGATTTGAAAAGAGATCATCCAATTAACAAAATGGATGATATGGAAA
AA264        AATGGTTTAAATTGTTAATGAGTAAATATGAAAAAGATAGAGATGATTTGAAAAGAGATCATCCAATTAACAAAATGGATGATATGGAAA
AM1737       AATGGTTTAAATTGTTAATGAGTAAATATGAAAAAGATAGAGATGATTTGAAAAGATATCATCCAATTAACAAAATGGATGATATGGAAA
SC74         AATGGTTTAAATTGTTAATGAGTAAATATGAAAAAGATAGAGATGATTTGAAAAGAGATCATCCAATTAACAAAATGGATGATATGGAAA
TAB166       AATGGTTTAAATTGTTAATGAGTAAATATGAAAAAGATAGAGATGATTTGAAAAGAGATCATCCAATTAACAAAATGGATGATATGGAAA
TAB138       AATGGTTTAAATTGTTAATGAGTAAATATGAAAAAGATAGAGATGATTTGAAAAGAGATCATCCAATTAACAAAATGGATGATATGGAAA
TAB130       AATGGTTTAAATTGTTAATGAGTAAATATGAAAAAGATAGAGATGATTTGAAAAGAGATCATCCAATTAACAAAATGGATGATATGGAAA
TAB131       AATGGTTTAAATTGTTAATGAGTAAATATGAAAAAGATAGAGATGATTTGAAAAGAGATCATCCAATTAACAAAATGGATGATATGGAAA
TAB151       AATGGTTTAAATTGTTAATGAGTAAATATGAAAAAGATAGAGATGATTTGAAAAGAGATCATCCAATTAACAAAATGGATGATATGGAAA
TAB152       AATGGTTTAAATTGTTAATGAGTAAATATGAAAAAGATAGAGATGATTTGAAAAGAGATCATCCAATTAACAAAATGGATGATATGGAAA
SC52         AATGGTTTAAATTGTTAATGAGTAAATATGAAAAAGATAGAGATGATTTGAAAAGAGATCATCCAATTAACAAAATGGATGATATGGAAA
SC55         AATGGTTTAAATTGTTAATGAGTAAATATGAAAAAGATAGAGATGATTTGAAAAGAGATCATCCAATTAACAAAATGGATGATATGGAAA
SC03         AATGGTTTAAATTGTTAATGAGTAAATATGAAAAAGATAGAGATGATTTGAAAAGAGATCATCCAATTAACAAAATGGATGATATGGAAA
SC53         AATGGTTTAAATTGTTAATGAGTAAATATGAAAAAGATAGAGATGATTTGAAAAGAGATCATCCAATTAACAAAATGGATGATATGGAAA
TAB141       AATGGTTTAAATTGTTAATGAGTAAATATGAAAAAGATAGAGATGATTTGAAAAGAGATCATCCAATTAACAAAATGGATGATATGGAAA
TAB123       AATGGTTTAAATTGTTAATGAGTAAATATGAAAAAGATAGAGATGATTTGAAAAGAGATCATCCAATTAACAAAATGGATGATATGGAAA
AM1803       AATGGTTTAAATTGTTAATGAGTAAATATGAAAAAGATAGAGATGATTTGAAAAGAGATCATCCAATTAACAAAATGGATGATATGGAAA
AM1814       AATGGTTTAAATTGTTAATGAGTAAATATGAAAAAGATAGAGATGATTTGAAAAGAGATCATCCAATTAACAAAATGGATGATATGGAAA
AM1802       AATGGTTTAAATTGTTAATGAGTAAATATGAAAAAGATAGAGATGATTTGAAAAGAGATCATCCAATTAACAAAATGGATGATATGGAAA
AM1811       AATGGTTTAAATTGTTAATGAGTAAATATGAAAAAGATAGAGATGATTTGAAAAGAGATCATCCAATTAACAAAATGGATGATATGGAAA
SC50         AATGGTTTAAATTGTTAATGAGTAAATATGAAAAAGATAGAGATGATTTGAAAAGAGATCATCCAATTAACAAAATGGATGATATGGAAA
SC56         AATGGTTTAAATTGTTAATGAGTAAATATGAAAAAGATAGAGATGATTTGAAAAGAGATCATCCAATTAACAAAATGGATGATATGGAAA
TAB153       AATGGTTTAAATTGTTAATGAGTAAATATGAAAAAGATAGAGATGATTTGAAAAGAGATCATCCAATTAACAAAATGGATGATATGGAAA
TAB154       AATGGTTTAAATTGTTAATGAGTAAATATGAAAAAGATAGAGATGATTTGAAAAGAGATCATCCAATTAACAAAATGGATGATATGGAAA
TAB156       AATGGTTTAAATTGTTAATGAGTAAATATGAAAAAGATAGAGATGATTTGAAAAGAGATCATCCAATTAACAAAATGGATGATATGGAAA
TAB136       AATGGTTTAAATTGTTAATGAGTAAATATGAAAAAGATAGAGATGATTTGAAAAGAGATCATCCAATTAACAAAATGGATGATATGGAAA
AM1880       AATGGTTTAAATTGTTAATGAGTAAATATGAAAAAGATAGAGATGATTTGAAAAGAGATCATCCAATTAACAAAATGGATGATATGGAAA
PF3D7_       AATGGTTTAAATTGTTAATGAGTAAATATGAAAAAGATAGAGATGATTTGAAAAGAGATCATCCAATTAACAAAATGGATGATATGGAAA
PfSD01       AATGGTTTAAATTGTTAATGAGTAAATATGAAAAAGATAGAGATGATTTGAAAAGAGATCATCCAATTAACAAAATGGATGATATGGAAA
Pf7G8        AATGGTTTAAATTGTTAATGAGTAAATATGAAAAAGATAGAGATGATTTGAAAAGAGATCATCCAATTAACAAAATGGATGATATGGAAA
FCR3 GENE    AATGGTTTAAATTGTTAATGAGTAAATATGAAAAAGATAGAGATGATTTGAAAAGAGATCATCCAATTAACAAAATGGATGATATGGAAA
MS822 GENE   ------------------------------------------------------------------------------------------
PfCD01_      AATGGTTTAAATTGTTAATGAGTAAATATGAAAAAGATAGAGATGATTTGAAAAGAGATCATCCAATTAACAAAATGGATGATATGGAAA
PfKH01__     AATGGTTTAAATTGTTAATGAGTAAATATGAAAAAGATAGAGATGATTTGAAAAGAGATCATCCAATTAACAAAATGGATGATATGGAAA
PfDd2__      AATGGTTTAAATTGTTAATGAGTAAATATGAAAAAGATAGAGATGATTTGAAAAGAGATCATCCAATTAACAAAATGGATGATATGGAAA
PfSN01_      AATGGTTTAAATTGTTAATGAGTAAATATGAAAAAGATAGAGATGATTTGAAAAGAGATCATCCAATTAACAAAATGGATGATATGGAAA
PfGN01       AATGGTTTAAATTGTTAATGAGTAAATATGAAAAAGATAGAGATGATTTGAAAAGAGATCATCCAATTAACAAAATGGATGATATGGAAA
PfKE01       AATGGTTTAAATTGTTAATGAGTAAATATGAAAAAGATAGAGATGATTTGAAAAGAGATCATCCAATTAACAAAATGGATGATATGGAAA
PfKH02       AATGGTTTAAATTGTTAATGAGTAAATATGAAAAAGATAGAGATGATTTGAAAAGAGATCATCCAATTAACAAAATGGATGATATGGAAA
PfGB4        AATGGTTTAAATTGTTAATGAGTAAATATGAAAAAGATAGAGATGATTTGAAAAGAGATCATCCAATTAACAAAATGGATGATATGGAAA
PfGA01_      AATGGTTTAAATTGTTAATGAGTAAATATGAAAAAGATAGAGATGATTTGAAAAGAGATCATCCAATTAACAAAATGGATGATATGGAAA
PfHB3_       AATGGTTTAAATTGTTAATGAGTAAATATGAAAAAGATAGAGATGATTTGAAAAGAGATCATCCAATTAACAAAATGGATGATATGGAAA
PfIT_        AATGGTTTAAATTGTTAATGAGTAAATATGAAAAAGATAGAGATGATTTGAAAAGAGATCATCCAATTAACAAAATGGATGATATGGAAA
```

```
                     4510      4520      4530      4540      4550      4560      4570      4580      4590
             ....|....|....|....|....|....|....|....|....|....|....|....|....|....|....|....|....|....|
PF3D7 CDS    AGAAAAAGTTGATAACAAAATTATTTATTGAAATACATATGATGGTAATTGAAAATTCTAAGGAGGAAGAATGTTATAGGAATAAAAAAA
MS822 mRNA   ------------------------------------------------------------------------------------------
FCR3 mRNA    AGAAAAAGTTGATAACAAAATTATTTATTGAAATACATATGATGGTAATTGAAAATTCTAAGGAGGAAGAATGTTATAGGAATAAAAAAA
AM1898       AGAAAAAGTTGATAACAAAATTATTTATTGAAATACATATGATGGTAATTGAAAATTCTAAGGAGGAAGAATGTTATAGGAATAAAAAAA
AA235        AGAAAAAGTTGATAACAAAATTATTTATTGAAATACATATGATGGTAATTGAAAATTCTAAGGAGGAAGAATGTTATAGGAATAAAAAAA
AA243        AGAAAAAGTTGATAACAAAATTATTTATTGAAATACATATGATGGTAATTGAAAATTCTAAGGAGGAAGAATGTTATAGGAATAAAAAAA
AA258        AGAAAAAGTTGATAACAAAATTATTTATTGAAATACATATGATGGTAATTGAAAATTCTAAGGAGGAAGAATGTTATAGGAATAAAAAAA
AA264        AGAAAAAGTTGATAACAAAATTATTTATTGAAATACATATGATGGTAATTGAAAATTCTAAGGAGGAAGAATGTTATAGGAATAAAAAAA
AM1737       AGAAAAAGTTGATAACAAAATTATTTATTGAAATTCATATGATGGTAATTGAAAATTCTAAGGAGGAAGAATGTTATAGGAATAAAAAAA
SC74         AGAAAAAGTTGATAACAAAATTATTTATTGAAATACATATGATGGTAATTGAAAATTCTAAGGAGGAAGAATGTTATAGGAATAAAAAAA
TAB166       AGAAAAAGTTGATAACAAAATTATTTATTGAAATACATATGATGGTAATTGAAAATTCTAAGGAGGAAGAATGTTATAGGAATAAAAAAA
TAB138       AGAAAAAGTTGATAACAAAATTATTTATTGAAATACATATGATGGTAATTGAAAATTCTAAGGAGGAAGAATGTTATAGGAATAAAAAAA
TAB130       AGAAAAAGTTGATAACAAAATTATTTATTGAAATTCATATGATGGTAATTGAAAATTCTAAGGAGGAAGAATGTTATAGGAATAAACAAA
TAB131       AGAAAAAGTTGATAACAAAATTATTTATTGAAATACATATGATGGTAATTGAAAATTCTAAGGAGGAAGAATGTTATAGGAATAAAAAAA
TAB151       AGAAAAAGTTGATAACAAAATTATTTATTGAAATACATATGATGGTAATTGAAAATTCTAAGGAGGAAGAATGTTATAGGAATAAAAAAA
TAB152       AGAAAAAGTTGATAACAAAATTATTTATTGAAATACATATGATGGTAATTGAAAATTCTAAGGAGGAAGAATGTTATAGGAATAAAAAAA
SC52         AGAAAAAGTTGATAACAAAATTATTTATTGAAATTCATATGATGGTAATTGAAAATTCTAAGGAGGAAGAATGTTATAGGAATAAAAAAA
SC55         AGAAAAAGTTGATAACAAAATTATTTATTGAAATACATATGATGGTAATTGAAAATTCTAAGGAGGAAGAATGTTATAGGAATAAAAAAA
SC03         AGAAAAAGTTGATAACAAAATTATTTATTGAAATTCATATGATGGTAATTGAAAATTCTAAGGAGGAAGAATGTTATAGGAATAAAAAAA
SC53         AGAAAAAGTTGATAACAAAATTATTTATTGAAATACATATGATGGTAATTGAAAATTCTAAGGAGGAAGAATGTTATAGGAATAAAAAAA
TAB141       AGAAAAAGTTGATAACAAAATTATTTATTGAAATACATATGATGGTAATTGAAAATTCTAAGGAGGAAGAATGTTATAGGAATAAAAAAA
TAB123       AGAAAAAGTTGATAACAAAATTATTTATTGAAATACATATGATGGTAATTGAAAATTCTAAGGAGGAAGAATGTTATAGGAATAAAAAAA
AM1803       AGAAAAAGTTGATAACAAAATTATTTATTGAAATTCATATGATGGTAATTGAAAATTCTAAGGAGGAAGAATGTTATAGGAATAAAAAAA
AM1814       AGAAAAAGTTGATAACAAAATTATTTATTGAAATACATATGATGGTAATTGAAAATTCTAAGGAGGAAGAATGTTATAGGAATAAAAAAA
AM1802       AGAAAAAGTTGATAACAAAATTATTTATTGAAATTCATATGATGGTAATTGAAAATTCTAAGGAGGAAGAATGTTATAGGAATAAAAAAA
AM1811       AGAAAAAGTTGATAACAAAATTATTTATTGAAATTCATATGATGGTAATTGAAAATTCTAAGGAGGAAGAATGTTATAGGAATAAAAAAA
SC50         AGAAAAAGTTGATAACAAAATTATTTATTGAAATTCATATGATGGTAATTGAAAATTCTAAGGAGGAAGAATGTTATAGGAATAAAAAAA
SC56         AGAAAAAGTTGATAACAAAATTATTTATTGAAATTCATATGATGGTAATTGAAAATTCTAAGGAGGAAGAATGTTATAGGAATAAAAAAA
TAB153       AGAAAAAGTTGATAACAAAATTATTTATTGAAATTCATATGATGGTAATTGAAAATTCTAAGGAGGAAGAATGTTATAGGAATAAAAAAA
TAB154       AGAAAAAGTTGATAACAAAATTATTTATTGAAATACATATGATGGTAATTGAAAATTCTAAGGAGGAAGAATGTTATAGGAATAAAAAAA
TAB156       AGAAAAAGTTGATAACAAAATTATTTATTGAAATTCATATGATGGTAATTGAAAATTCTAAGGAGGAAGAATGTTATAGGAATAAAAAAA
TAB136       AGAAAAAGTTGATAACAAAATTATTTATTGAAATTCATATGATGGTAATTGAAAATTCTAAGGAGGAAGAATGTTATAGGAATAAACAAA
AM1880       AGAAAAAGTTGATAACAAAATTATTTATTGAAATACATATGATGGTAATTGAAAATTCTAAGGAGGAAGAATGTTATAGGAATAAAAAAA
PF3D7_       AGAAAAAGTTGATAACAAAATTATTTATTGAAATACATATGATGGTAATTGAAAATTCTAAGGAGGAAGAATGTTATAGGAATAAAAAAA
PfSD01       AGAAAAAGTTGATAACAAAATTATTTATTGAAATTCATATGATGGTAATTGAAAATTCTAAGGAGGAAGAATGTTATAGGAATAAAAAAA
Pf7G8        AGAAAAAGTTGATAACAAAATTATTTATTGAAATTCATATGATGGTAATTGAAAATTCTAAGGAGGAAGAATGTTATAGGAATAAAAAAA
FCR3 GENE    AGAAAAAGTTGATAACAAAATTATTTATTGAAATACATATGATGGTAATTGAAAATTCTAAGGAGGAAGAATGTTATAGGAATAAAAAAA
MS822 GENE   ------------------------------------------------------------------------------------------
PfCD01_      AGAAAAAGTTGATAACAAAATTATTTATTGAAATTCATATGATGGTAATTGAAAATTCTAAGGAGGAAGAATGTTATAGGAATAAAAAAA
PfKH01__     AGAAAAAGTTGATAACAAAATTATTTATTGAAATACATATGATGGTAATTGAAAATTCTAAGGAGGAAGAATGTTATAGGAATAAAAAAA
PfDd2__      AGAAAAAGTTGATAACAAAATTATTTATTGAAATTCATATGATGGTAATTGAAAATTCTAAGGAGGAAGAATGTTATAGGAATAAAAAAA
PfSN01_      AGAAAAAGTTGATAACAAAATTATTTATTGAAATACATATGATGGTAATTGAAAATTCTAAGGAGGAAGAATGTTATAGGAATAAAAAAA
PfGN01_      AGAAAAAGTTGATAACAAAATTATTTATTGAAATACATATGATGGTAATTGAAAATTCTAAGGAGGAAGAATGTTATAGGAATAAAAAAA
PfKE01       AGAAAAAGTTGATAACAAAATTATTTATTGAAATTCATATGATGGTAATTGAAAATTCTAAGGAGGAAGAATGTTATAGGAATAAAAAAA
PfKH02       AGAAAAAGTTGATAACAAAATTATTTATTGAAATTCATATGATGGTAATTGAAAATTCTAAGGAGGAAGAATGTTATAGGAATAAAAAAA
PfGB4        AGAAAAAGTTGATAACAAAATTATTTATTGAAATACATATGATGGTAATTGAAAATTCTAAGGAGGAAGAATGTTATAGGAATAAAAAAA
PfGA01_      AGAAAAAGTTGATAACAAAATTATTTATTGAAATACATATGATGGTAATTGAAAATTCTAAGGAGGAAGAATGTTATAGGAATAAAAAAA
PfHB3_       AGAAAAAGTTGATAACAAAATTATTTATTGAAATTCATATGATGGTAATTGAAAATTCTAAGGAGGAAGAATGTTATAGGAATAAAAAAA
PfIT_        AGAAAAAGTTGATAACAAAATTATTTATTGAAATACATATGATGGTAATTGAAAATTCTAAGGAGGAAGAATGTTATAGGAATAAAAAAA
```

```
                     4600      4610      4620      4630      4640      4650      4660      4670      4680
             ....|....|....|....|....|....|....|....|....|....|....|....|....|....|....|....|....|....|
PF3D7 CDS    A-TTTTGTTCAAACATATATAGATGAATTGAAAAAAGAACAAAATTTAGAACAGAATAAATATATGATAAATATATTAAATGATATACAA
MS822 mRNA   ------------------------------------------------------------------------------------------
FCR3 mRNA    AATTTTGTTCAAACATATATAGATGAATTGAAAAAAGAACAAAATTTAGAACAGAATAAATATATGATAAATATATTAAATGATATACAA
AM1898       A-TTTTGTTCAAACATATATAGATGAATTGAAAAAAGAACAAAATTTAGAACAGAATAAATATATGATAAATATATTAAATGATATACAA
AA235        A-TTTTGTTCAAACATATATAGATGAATTGAAAAAAGAACAAAATTTAGAACAGAATAAATATATGATAAATATATTAAATGATATACAA
AA243        A-TTTTGTTCAAACATATATAGATGAATTGAAAAAAGAACAAAATTTAGAACAGAATAAATATATGATAAATATATTAAATGATATACAA
AA258        A-TTTTGTTCAAACATATATAGATGAATTGAAAAAAGAACAAAATTTAGAACAGAATAAATATATGATAAATATATTAAATGATATACAA
AA264        A-TTTTGTTCAAACATATATAGATGAATTGAAAAAAGAACAAAATTTAGAACAGAATAAATATATGATAAATATATTAAATGATATACAA
AM1737       A-TTTTGTTCAAACATATATAGATGAATTGAAAAAAGAACAAAATTTAGAACAGAATAAATATATGATAAATATATTAAATGATATACAA
SC74         A-TTTTGTTCAAACATATATAGATGAATTGAAAAAAGAACAAAATTTAGAACAGAATAAATATATGATAAATATATTAAATGATATACAA
TAB166       A-TTTTGTTCAAACATATATAGATGAATTGAAAAAAGAACAAAATTTAGAACAGAATAAATATATGATAAATATATTAAATGATATACAA
TAB138       A-TTTTGTTCAAACATATATAGATGAATTGAAAAAAGAACAAAATTTAGAACAGAATAAATATATGATAAATATATTAAATGATATACAA
TAB130       A-TTTTGTTCAAACATATATAGATGAATTGAAAAAAGAACAAAATTTGGAACAGAATAAATATATGATAAATATATTAAATGATATACAA
TAB131       A-TTTTGTTCAAACATATATAGATGAATTGAAAAAAGAACAAAATTTAGAACAGAATAAATATATGATAAATATATTAAATGATATACAA
TAB151       A-TTTTGTTCAAACATATATAGATGAATTGAAAAAAGAACAAAATTTAGAACAGAATAAATATATGATAAATATATTAAATGATATACAA
TAB152       A-TTTTGTTCAAACATATATAGATGAATTGAAAAAAGAACAAAATTTAGAACAGAATAAATATATGATAAATATATTAAATGATATACAA
SC52         A-TTTTGTTCAAACATATATAGATGAATTGAAAAAAGAACAAAATTTAGAACAGAATAAATATATGATAAATATATTAAATGATATACAA
SC55         A-TTTTGTTCAAACATATATAGATGAATTGAAAAAAGAACAAAATTTAGAACAGAATAAATATATGATAAATATATTAAATGATATACAA
SC03         A-TTTTGTTCAAACATATATAGATGAATTGAAAAAAGAACAAAATTTAGAACAGAATAAATATATGATAAATATATTAAATGATATACAA
SC53         A-TTTTGTTCAAACATATATAGATGAATTGAAAAAAGAACAAAATTTAGAACAGAATAAATATATGATAAATATATTAAATGATATACAA
TAB141       A-TTTTGTTCAAACATATATAGATGAATTGAAAAAAGAACAAAATTTAGAACAGAATAAATATATGATAAATATATTAAATGATATACAA
TAB123       A-TTTTGTTCAAACATATATAGATGAATTGAAAAAAGAACAAAATTTAGAACAGAATAAATATATGATAAATATATTAAATGATATACAA
AM1803       A-TTTTGTTCAAACATATATAGATGAATTGAAAAAAGAACAAAATTTAGAACAGAATAAATATATGATAAATATATTAAATGATATACAA
AM1814       A-TTTTGTTCAAACATATATAGATGAATTGAAAAAAGAACAAAATTTAGAACAGAATAAATATATGATAAATATATTAAATGATATACAA
AM1802       A-TTTTGTTCAAACATATATAGATGAATTGAAAAAAGAACAAAATTTAGAACAGAATAAATATATGATAAATATATTAAATGATATACAA
AM1811       A-TTTTGTTCAAACATATATAGATGAATTGAAAAAAGAACAAAATTTAGAACAGAATAAATATATGATAAATATATTAAATGATATACAA
SC50         A-TTTTGTTCAAACATATATAGATGAATTGAAAAAAGAACAAAATTTAGAACAGAATAAATATATGATAAATATATTAAATGATATACAA
SC56         A-TTTTGTTCAAACATATATAGATGAATTGAAAAAAGAACAAAATTTAGAACAGAATAAATATATGATAAATATATTAAATGATATACAA
TAB153       A-TTTTGTTCAAACATATATAGATGAATTGAAAAAAGAACAAAATTTAGAACAGAATAAATATATGATAAATATATTAAATGATATACAA
TAB154       A-TTTTGTTCAAACATATATAGATGAATTGAAAAAAGAACAAAATTTAGAACAGAATAAATATATGATAAATATATTAAATGATATACAA
TAB156       A-TTTTGTTCAAACATATATAGATGAATTGAAAAAAGAACAAAATTTAGAACAGAATAAATATATGATAAATATATTAAATGATATACAA
TAB136       A-TTTTGTTCAAACATATATAGATGAATTGAAAAAAGAACAAAATTTGGAACAGAATAAATATATGATAAATATATTAAATGATATACAA
AM1880       A-TTTTGTTCAAACATATATAGATGAATTGAAAAAAGAACAAAATTTAGAACAGAATAAATATATGATAAATATATTAAATGATATACAA
PF3D7_       A-TTTTGTTCAAACATATATAGATGAATTGAAAAAAGAACAAAATTTAGAACAGAATAAATATATGATAAATATATTAAATGATATACAA
PfSD01       A-TTTTGTTCAAACATATATAGATGAATTGAAAAAAGAACAAAATTTAGAACAGAATAAATATATGATAAATATATTAAATGATATACAA
Pf7G8        A-TTTTGTTCAAACATATATAGATGAATTGAAAAAAGAACAAAATTTAGAACAGAATAAATATATGATAAATATATTAAATGATATACAA
FCR3 GENE    AATTTTGTTCAAACATATATAGATGAATTGAAAAAAGAACAAAATTTAGAACAGAATAAATATATGATAAATATATTAAATGATATACAA
MS822 GENE   ------------------------------------------------------------------------------------------
PfCD01_      A-TTTTGTTCAAACATATATAGATGAATTGAAAAAAGAACAAAATTTAGAACAGAATAAATATATGATAAATATATTAAATGATATACAA
PfKH01__     A-TTTTGTTCAAACATATATAGATGAATTGAAAAAAGAACAAAATTTAGAACAGAATAAATATATGATAAATATATTAAATGATATACAA
PfDd2__      A-TTTTGTTCAAACATATATAGATGAATTGAAAAAAGAACAAAATTTAGAACAGAATAAATATATGATAAATATATTAAATGATATACAA
PfSN01_      A-TTTTGTTCAAACATATATAGATGAATTGAAAAAAGAACAAAATTTAGAACAGAATAAATATATGATAAATATATTAAATGATATACAA
PfGN01       A-TTTTGTTCAAACATATATAGATGAATTGAAAAAAGAACAAAATTTAGAACAGAATAAATATATGATAAATATATTAAATGATATACAA
PfKE01       A-TTTTGTTCAAACATATATAGATGAATTGAAAAAAGAACAAAATTTAGAACAGAATAAATATATGATAAATATATTAAATGATATACAA
PfKH02       A-TTTTGTTCAAACATATATAGATGAATTGAAAAAAGAACAAAATTTAGAACAGAATAAATATATGATAAATATATTAAATGATATACAA
PfGB4        A-TTTTGTTCAAACATATATAGATGAATTGAAAAAAGAACAAAATTTAGAACAGAATAAATATATGATAAATATATTAAATGATATACAA
PfGA01_      A-TTTTGTTCAAACATATATAGATGAATTGAAAAAAGAACAAAATTTAGAACAGAATAAATATATGATAAATATATTAAATGATATACAA
PfHB3_       A-TTTTGTTCAAACATATATAGATGAATTGAAAAAAGAACAAAATTTAGAACAGAATAAATATATGATAAATATATTAAATGATATACAA
PfIT_        A-TTTTGTTCAAACATATATAGATGAATTGAAAAAAGAACAAAATTTAGAACAGAATAAATATATGATAAATATATTAAATGATATACAA
```

```
                    4690      4700      4710      4720      4730      4740      4750      4760      4770
               ....|....|....|....|....|....|....|....|....|....|....|....|....|....|....|....|....|....|
PF3D7 CDS      AATGATATACAATTTGGGCATGATCATAATAATTACAATGAATGGAAACAGGAAAAATGGTTTAAAAATTTAAAAAAAGAATGGAAAGAA
MS822 mRNA     ------------------------------------------------------------------------------------------
FCR3 mRNA      AATGATATACAATTTGGGCATGATCATAATAATTACAATGAATGGAAACAGGAAAAATGGTTTAAAAATTTAAAAAAAGAATGGAAAGAA
AM1898         AATGATATACAATTTGGGCATGATCATAATAATTACAATGAATGGAAACAGGAAAAATGGTTTAAAAATTTAAAAAAAGAATGGAAAGAA
AA235          AATGATATACAATTTGGGCATGATCATAATAATTACAATGAATGGAAACAGGAAAAATGGTTTAAAAATTTAAAAAAAGAATGGAAAGAA
AA243          AATGATATACAATTTGGGCATGATCATAATAATTACAATGAATGGAAACAGGAAAAATGGTTTAAAAATTTAAAAAAAGAATGGAAAGAA
AA258          AATGATATACAATTTGGGCATGATCATAATAATTACAATGAATGGAAACAGGAAAAATGGTTTAAAAATTTAAAAAAAGAATGGAAAGAA
AA264          AATGATATACAATTTGGGCATGATCATAATAATTACAATGAATGGAAACAGGAAAAATGGTTTAAAAATTTAAAAAAAGAATGGAAAGAA
AM1737         AATGATATACAATTTGGGCATGATCATAATAATTACAATGAATGGAAACAGGAAAAATGGTTTAAAAATTTAAAAAAAGAATGGAAAGAA
SC74           AATGATATACAATTTGGGCATGATCATAATAATTACAATGAATGGAAACAGGAAAAATGGTTTAAAAATTTAAAAAAAGAATGGAAAGAA
TAB166         AATGATATACAATTTGGGCATGATCATAATAATTACAATGAATGGAAACAGGAAAAATGGTTTAAAAATTTAAAAAAAGAATGGAAAGAA
TAB138         AATGATATACAATTTGGGCATGATCATAATAATTACAATGAATGGAAACAGGAAAAATGGTTTAAAAATTTAAAAAAAGAATGGAAAGAA
TAB130         AATGATATACAATTTGGGCATGATCATAATAATTACAATGAATGGAAACAGGAAAAATGGTTTAAAAATTTAAAAAAAGAATGGAAAGAA
TAB131         AATGATATACAATTTGGGCATGATCATAATAATTACAATGAATGGAAACAGGAAAAATGGTTTAAAAATTTAAAAAAAGAATGGAAAGAA
TAB151         AATGATATACAATTTGGGCATGATCATAATAATTACAATGAATGGAAACAGGAAAAATGGTTTAAAAATTTAAAAAAAGAATGGAAAGAA
TAB152         AATGATATACAATTTGGGCATGATCATAATAATTACAATGAATGGAAACAGGAAAAATGGTTTAAAAATTTAAAAAAAGAATGGAAAGAA
SC52           AATGATATACAATTTGGGCATGATCATAATAATTACAATGAATGGAAACAGGAAAAATGGTTTAAAAATTTAAAAAAAGAATGGAAAGAA
SC55           AATGATATACAATTTGGGCATGATCATAATAATTACAATGAATGGAAACAGGAAAAATGGTTTAAAAATTTAAAAAAAGAATGGAAAGAA
SC03           AATGATATACAATTTGGGCATGATCATAATAATTACAATGAATGGAAACAGGAAAAATGGTTTAAAAATTTAAAAAAAGAATGGAAAGAA
SC53           AATGATATACAATTTGGGCATGATCATAATAATTACAATGAATGGAAACAGGAAAAATGGTTTAAAAATTTAAAAAAAGAATGGAAAGAA
TAB141         AATGATATACAATTTGGGCATGATCATAATAATTACAATGAATGGAAACAGGAAAAATGGTTTAAAAATTTAAAAAAAGAATGGAAAGAA
TAB123         AATGATATACAATTTGGGCATGATCATAATAATTACAATGAATGGAAACAGGAAAAATGGTTTAAAAATTTAAAAAAAGAATGGAAAGAA
AM1803         AATGATATACAATTTGGGCATGATCATAATAATTACAATGAATGGAAACAGGAAAAATGGTTTAAAAATTTAAAAAAAGAATGGAAAGAA
AM1814         AATGATATACAATTTGGGCATGATCATAATAATTACAATGAATGGAAACAGGAAAAATGGTTTAAAAATTTAAAAAAAGAATGGAAAGAA
AM1802         AATGATATACAATTTGGGCATGATCATAATAATTACAATGAATGGAAACAGGAAAAATGGTTTAAAAATTTAAAAAAAGAATGGAAAGAA
AM1811         AATGATATACAATTTGGGCATGATCATAATAATTACAATGAATGGAAACAGGAAAAATGGTTTAAAAATTTAAAAAAAGAATGGAAAGAA
SC50           AATGATATACAATTTGGGCATGATCATAATAATTACAATGAATGGAAACAGGAAAAATGGTTTAAAAATTTAAAAAAAGAATGGAAAGAA
SC56           AATGATATACAATTTGGGCATGATCATAATAATTACAATGAATGGAAACAGGAAAAATGGTTTAAAAATTTAAAAAAAGAATGGAAAGAA
TAB153         AATGATATACAATTTGGGCATGATCATAATAATTACAATGAATGGAAACAGGAAAAATGGTTTAAAAATTTAAAAAAAGAATGGAAAGAA
TAB154         AATGATATACAATTTGGGCATGATCATAATAATTACAATGAATGGAAACAGGAAAAATGGTTTAAAAATTTAAAAAAAGAATGGAAAGAA
TAB156         AATGATATACAATTTGGGCATGATCATAATAATTACAATGAATGGAAACAGGAAAAATGGTTTAAAAATTTAAAAAAAGAATGGAAAGAA
TAB136         AATGATATACAATTTGGGCATGATCATAATAATTACAATGAATGGAAACAGGAAAAATGGTTTAAAAATTTAAAAAAAGAATGGAAAGAA
AM1880         AATGATATACAATTTGGGCATGATCATAATAATTACAATGAATGGAAACAGGAAAAATGGTTTAAAAATTTAAAAAAAGAATGGAAAGAA
PF3D7_         AATGATATACAATTTGGGCATGATCATAATAATTACAATGAATGGAAACAGGAAAAATGGTTTAAAAATTTAAAAAAAGAATGGAAAGAA
PfSD01         AATGATATACAATTTGGGCATGATCATAATAATTACAATGAATGGAAACAGGAAAAATGGTTTAAAAATTTGAAAAAAGAATGGAAAGAA
Pf7G8          AATGATATACAATTTGGGCATGATCATAATAATTACAATGAATGGAAACAGGAAAAATGGTTTAAAAATTTAAAAAAAGAATGGAAAGAA
FCR3 GENE      AATGATATACAATTTGGGCATGATCATAATAATTACAATGAATGGAAACAGGAAAAATGGTTTAAAAATTTAAAAAAAGAATGGAAAGAA
MS822 GENE     ------------------------------------------------------------------------------------------
PfCD01_        AATGATATACAATTTGGGCATGATCATAATAATTACAATGAATGGAAACAGGAAAAATGGTTTAAAAATTTAAAAAAAGAATGGAAAGAA
PfKH01__       AATGATATACAATTTGGGCATGATCATAATAATTACAATGAATGGAAACAGGAAAAATGGTTTAAAAATTTAAAAAAAGAATGGAAAGAA
PfDd2__        AATGATATACAATTTGGGCATGATCATAATAATTACAATGAATGGAAACAGGAAAAATGGTTTAAAAATTTAAAAAAAGAATGGAAAGAA
PfSN01_        AATGATATACAATTTGGGCATGATCATAATAATTACAATGAATGGAAACAGGAAAAATGGTTTAAAAATTTAAAAAAAGAATGGAAAGAA
PfGN01         AATGATATACAATTTGGGCATGATCATAATAATTACAATGAATGGAAACAGGAAAAATGGTTTAAAAATTTAAAAAAAGAATGGAAAGAA
PfKE01         AATGATATACAATTTGGGCATGATCATAATAATTACAATGAATGGAAACAGGAAAAATGGTTTAAAAATTTAAAAAAAGAATGGAAAGAA
PfKH02         AATGATATACAATTTGGGCATGATCATAATAATTACAATGAATGGAAACAGGAAAAATGGTTTAAAAATTTAAAAAAAGAATGGAAAGAA
PfGB4          AATGATATACAATTTGGGCATGATCATAATAATTACAATGAATGGAAACAGGAAAAATGGTTTAAAAATTTAAAAAAAGAATGGAAAGAA
PfGA01_        AATGATATACAATTTGGGCATGATCATAATAATTACAATGAATGGAAACAGGAAAAATGGTTTAAAAATTTAAAAAAAGAATGGAAAGAA
PfHB3_         AATGATATACAATTTGGGCATGATCATAATAATTACAATGAATGGAAACAGGAAAAATGGTTTAAAAATTTAAAAAAAGAATGGAAAGAA
PfIT_          AATGATATACAATTTGGGCATGATCATAATAATTACAATGAATGGAAACAGGAAAAATGGTTTAAAAATTTAAAAAAAGAATGGAAAGAA
```

```
                        4780      4790      4800      4810      4820      4830      4840      4850      4860
               ....|....|....|....|....|....|....|....|....|....|....|....|....|....|....|....|....|....|
PF3D7 CDS      GGGGAAAGAAAAAACTTTCTTCATGTAGAAAACGAAAATTTAGATAACATACAAGCTAATAAACTTAATAATTATATATTAGAAATTCAA
MS822 mRNA     ------------------------------------------------------------------------------------------
FCR3 mRNA      GGGGAAAGAAAAAACTTTCTTCATGTAGAAAACGAAAATTTAGATAACATACAAGCTAATAAACTTAATAATTATATATTAGAAATTCAA
AM1898         GGGGAAAGAAAAAACTTTCTTCATGTAGAAAACGAAAATTTAGATAACATACAAGCTAATAAACTTAATAATTATATATTAGAAATTCAA
AA235          GGGGAAAGAAAAAACTTTCTTCATATAGAAAACGAAAATTTAGATAACATACAAGTTAATAAATTTAATAATTATATATTAGAAATTCAA
AA243          GGGGAAAGAAAAAACTTTCTTCATATAGAAAACGAAAATTTAGATAACATACAAGTTAATAAATTTAATAATTATATATTAGAAATTCAA
AA258          GGGGAAAGAAAAAACTTTCTTCATATAGAAAACGAAAATTTAGATAACATACAAGTTAATAAATTTAATAATTATATATTAGAAATTCAA
AA264          GGGGAAAGAAAAAACTTTCTTCATATAGAAAACGAAAATTTAGATAACATACAAGTTAATAAATTTAATAATTATATATTAGAAATTCAA
AM1737         GGGGAAAGAAAAAACTTTCTTCATATAGAAAACGAAAATTTAGATAACATACAAGTTAATAAATTTAATAATTATATATTAGAAATTCAA
SC74           GGGGAAAGAAAAAACTTTCTTCATGTAGAAAACGAAAATTTAGATAACATACAAGCTAATAAACTTAATAATTATATATTAGAAATTCAA
TAB166         GGGGAAAGAAAAAACTTTCTTCATGTAGAAAACGAAAATTTAGATAACATACAAGCTAATAAACTTAATAATTATATATTAGAAATTCAA
TAB138         GGGGAAAGAAAAAACTTTCTTCATGTAGAAAACGAAAATTTAGATAACATACAAGCTAATAAACTTAATAATTATATATTAGAAATTCAA
TAB130         GGGGAAAGAAAAAACTTTCTTCATATAGAAAACGAAAATTTAGATAACATACAAGTTAATAAATTTAATAATTATATATTAGAAATTCAA
TAB131         GGGGAAAGAAAAAACTTTCTTCATATAGAAAACGAAAATTTAGATAACATACAAGTTAATAAATTTAATAATTATATATTAGAAATTCAA
TAB151         GGGGAAAGAAAAAACTTTCTTCATATAGAAAACGAAAATTTAGATAACATACAAGTTAATAAATTTAATAATTATATATTAGAAATTCAA
TAB152         GGGGAAAGAAAAAACTTTCTTCATGTAGAAAACGAAAATTTAGATAACATACAAGCTAATAAACTTAATAATTATATATTAGAAATTCAA
SC52           GGGGAAAGAAAAAACTTTCTTCATATAGAAAACGAAAATTTAGATAACATACAAGTTAATAAATTTAATAATTATATATTAGAAATTCAA
SC55           GGGGAAAGAAAAAACTTTCTTCATGTAGAAAACGAAAATTTAGATAACATACAAGCTAATAAACTTAATAATTATATATTAGAAATTCAA
SC03           GGGGAAAGAAAAAACTTTCTTCATGTAGAAAACGAAAATTTAGATAACATACAAGCTAATAAACTTAATAATTATATATTAGAAATTCAA
SC53           GGGGAAAGAAAAAACTTTCTTCATGTAGAAAACGAAAATTTAGATAACATACAAGCTAATAAACTTAATAATTATATATTAGAAATTCAA
TAB141         GGGGAAAGAAAAAACTTTCTTCATGTAGAAAACGAAAATTTAGATAACATACAAGCTAATAAACTTAATAATTATATATTAGAAATTCAA
TAB123         GGGGAAAGAAAAAACTTTCTTCATGTAGAAAACGAAAATTTAGATAACATACAAGCTAATAAACTTAATAATTATATATTAGAAATTCAA
AM1803         GGGGAAAGAAAAAACTTTCTTCATATAGAAAACGAAAATTTAGATAACATACAAGTTAATAAATTTAATAATTATATATTAGAAATTCAA
AM1814         GGGGAAAGAAAAAACTTTCTTCATGTAGAAAACGAAAATTTAGATAACATACAAGCTAATAAACTTAATAATTATATATTAGAAATTCAA
AM1802         GGGGAAAGAAAAAACTTTCTTCATGTAGAAAACGAAAATTTAGATAACATACAAGCTAATAAACTTAATAATTATATATTAGAAATTCAA
AM1811         GGGGAAAGAAAAAACTTTCTTCATATAGAAAACGAAAATTTAGATAACATACAAGTTAATAAATTTAATAATTATATATTAGAAATTCAA
SC50           GGGGAAAGAAAAAACTTTCTTCATATAGAAAACGAAAATTTAGATAACATACAAGTTAATAAATTTAATAATTATATATTAGAAATTCAA
SC56           GGGGAAAGAAAAAACTTTCTTCATATAGAAAACGAAAATTTAGATAACATACAAGTTAATAAATTTAATAATTATATATTAGAAATTCAA
TAB153         GGGGAAAGAAAAAACTTTCTTCATATAGAAAACGAAAATTTAGATAACATACAAGTTAATAAATTTAATAATTATATATTAGAAATTCAA
TAB154         GGGGAAAGAAAAAACTTTCTTCATATAGAAAACGAAAATTTAGATAACATACAAGTTAATAAATTTAATAATTATATATTAGAAATTCAA
TAB156         GGGGAAAGAAAAAACTTTCTTCATGTAGAAAACGAAAATTTAGATAACATACAAGCTAATAAACTTAATAATTATATATTAGAAATTCAA
TAB136         GGGGAAAGAAAAAACTTTCTTCATATAGAAAACGAAAATTTAGATAACATACAAGTTAATAAATTTAATAATTATATATTAGAAATTCAA
AM1880         GGGGAAAGAAAAAACTTTCTTCATGTAGAAAACGAAAATTTAGATAACATACAAGCTAATAAACTTAATAATTATATATTAGAAATTCAA
PF3D7_         GGGGAAAGAAAAAACTTTCTTCATGTAGAAAACGAAAATTTAGATAACATACAAGCTAATAAACTTAATAATTATATATTAGAAATTCAA
PfSD01         GGGGAAAGAAAAAACTTTCTTCATGTAGAAAACGAAAATTTAGATAACATACAAGCTAATAAACTTAATAATTATATATTAGAAATTCAA
Pf7G8          GGGGAAAGAAAAAACTTTCTTCATATAGAAAACGAAAATTTAGATAACATACAAGTTAATAAATTTAATAATTATATATTAGAAATTCAA
FCR3 GENE      GGGGAAAGAAAAAACTTTCTTCATGTAGAAAACGAAAATTTAGATAACATACAAGCTAATAAACTTAATAATTATATATTAGAAATTCAA
MS822 GENE     ------------------------------------------------------------------------------------------
PfCD01_        GGGGAAAGAAAAAACTTTCTTCATATAGAAAACGAAAATTTAGATAACATACAAGTTAATAAATTTAATAATTATATATTAGAAATTCAA
PfKH01__       GGGGAAAGAAAAAACTTTCTTCATATAGAAAACGAAAATTTAGATAACATACAAGTTAATAAATTTAATAATTATATATTAGAAATTCAA
PfDd2__        GGGGAAAGAAAAAACTTTCTTCATGTAGAAAACGAAAATTTAGATAACATACAAGCTAATAAACTTAATAATTATATATTAGAAATTCAA
PfSN01_        GGGGAAAGAAAAAACTTTCTTCATGTAGAAAACGAAAATTTAGATAACATACAAGCTAATAAACTTAATAATTATATATTAGAAATTCAA
PfGN01         GGGGAAAGAAAAAACTTTCTTCATGTAGAAAACGAAAATTTAGATAACATACAAGCTAATAAACTTAATAATTATATATTAGAAATTCAA
PfKE01         GGGGAAAGAAAAAACTTTCTTCATGTAGAAAACGAAAATTTAGATAACATACAAGCTAATAAACTTAATAATTATATATTAGAAATTCAA
PfKH02         GGGGAAAGAAAAAACTTTCTTCATATAGAAAACGAAAATTTAGATAACATACAAGTTAATAAATTTAATAATTATATATTAGAAATTCAA
PfGB4          GGGGAAAGAAAAAACTTTCTTCATATAGAAAACGAAAATTTAGATAACATACAAGTTAATAAATTTAATAATTATATATTAGAAATTCAA
PfGA01_        GGGGAAAGAAAAAACTTTCTTCATGTAGAAAACGAAAATTTAGATAACATACAAGCTAATAAACTTAATAATTATATATTAGAAATTCAA
PfHB3_         GGGGAAAGAAAAAACTTTCTTCATATAGAAAACGAAAATTTAGATAACATACAAGTTAATAAATTTAATAATTATATATTAGAAATTCAA
PfIT_          GGGGAAAGAAAAAACTTTCTTCATGTAGAAAACGAAAATTTAGATAACATACAAGCTAATAAACTTAATAATTATATATTAGAAATTCAA
```

```
                    4870      4880      4890      4900      4910      4920      4930      4940      4950
            ....|....|....|....|....|....|....|....|....|....|....|....|....|....|....|....|....|....|
PF3D7 CDS   AAAGCTATATTAAAAAATTACTGGGAAGATATGCAAATAAAATGGATAGATGATGATAATAAAACAGATTGGTTAAAAATAGCAATGAAT
MS822 mRNA  ------------------------------------------------------------------------------------------
FCR3 mRNA   AAAGCTATATTAAAAAATTACTGGGAAGATATGCAAATAAAATGGATAGATGATGATAATAAAACAGAATGGTTAAAAATAGCAATGAAT
AM1898      AAAGCTATATTAAAAAATTACTGGGAAGATATGCAAATAAAATGGATAGATGATGATAATAAAACAGATTGGTTAAAAATAGCAATGAAT
AA235       AAAGCTATATTAAAAAATTACTGGGAAGATATGCAAATAAAATGGATAGATGATGATAATAAAACAGATTGGTTAAAAATAGCAATGAAT
AA243       AAAGCTATATTAAAAAATTACTGGGAAGATATGCAAATAAAATGGATAGATGATGATAATAAAACAGATTGGTTAAAAATAGCAATGAAT
AA258       AAAGCTATATTAAAAAATTACTGGGAAGATATGCAAATAAAATGGATAGATGATGATAATAAAACAGATTGGTTAAAAATAGCAATGAAT
AA264       AAAGCTATATTAAAAAATTACTGGGAAGATATGGAAATAAAATGGATAGATGATGATAATAAAACAGATTGGTTAAAAATAGCAATGAAT
AM1737      AAAGCTATATTAAAAAATTACTGGGAAGATATGGAAATAAAATGGATAGATGATGATAATAAAACAGATTGGTTAAAAATAGCAATGAAT
SC74        AAAGCTATATTAAAAAATTACTGGGAAGATATGCAAATAAAATGGATAGATGATGATAATAAAACAGATTGGTTAAAAATAGCAATGAAT
TAB166      AAAGCTATATTAAAAAATTACTGGGAAGATATGCAAATAAAATGGATAGATGATGATAATAAAACAGATTGGTTAAAAATAGCAATGAAT
TAB138      AAAGCTATATTAAAAAATTACTGGGAAGATATGCAAATAAAATGGATAGATGATGATAATAAAACAGATTGGTTAAAAATAGCAATGAAT
TAB130      AAAGCTATATTAAAAAATTACTGGGAAGATATGGAAATAAAATGGATAGATGATGATAATAAAACAGATTGGTTAAAAATAGCAATGAAT
TAB131      AAAGCTATATTAAAAAATTACTGGGAAGATATGCAAATAAAATGGATAGATGATGATAATAAAACAGATTGGTTAAAAATAGCAATGAAT
TAB151      AAAGCTATATTAAAAAATTACTGGGAAGATATGCAAATAAAATGGATAGATGATGATAATAAAACAGATTGGTTAAAAATAGCAATGAAT
TAB152      AAAGCTATATTAAAAAATTACTGGGAAGATATGCAAATAAAATGGATAGATGATGATAATAAAACAGATTGGTTAAAAATAGCAATGAAT
SC52        AAAGCTATATTAAAAAATTACTGGGAAGATATGCAAATAAAATGGATAGATGATGATAATAAAACAGATTGGTTAAAAATAGCAATGAAT
SC55        AAAGCTATATTAAAAAATTACTGGGAAGATATGCAAATAAAATGGATAGATGATGATAATAAAACAGATTGGTTAAAAATAGCAATGAAT
SC03        AAAGCTATATTAAAAAATTACTGGGAAGATATGCAAATAAAATGGATAGATGATGATAATAAAACAGATTGGTTAAAAATAGCAATGAAT
SC53        AAAGCTATATTAAAAAATTACTGGGAAGATATGCAAATAAAATGGATAGATGATGATAATAAAACAGATTGGTTAAAAATAGCAATGAAT
TAB141      AAAGCTATATTAAAAAATTACTGGGAAGATATGCAAATAAAATGGATAGATGATGATAATAAAACAGATTGGTTAAAAATAGCAATGAAT
TAB123      AAAGCTATATTAAAAAATTACTGGGAAGATATGCAAATAAAATGGATAGATGATGATAATAAAACAGATTGGTTAAAAATAGCAATGAAT
AM1803      AAAGCTATATTAAAAAATTACTGGGAAGATATGCAAATAAAATGGATAGATGATGATAATAAAACAGATTGGTTAAAAATAGCAATGAAT
AM1814      AAAGCTATATTAAAAAATTACTGGGAAGATATGCAAATAAAATGGATAGATGATGATAATAAAACAGATTGGTTAAAAATAGCAATGAAT
AM1802      AAAGCTATATTAAAAAATTACTGGGAAGATATGCAAATAAAATGGATAGATGATGATAATAAAACAGATTGGTTAAAAATAGCAATGAAT
AM1811      AAAGCTATATTAAAAAATTACTGGGAAGATATGGAAATAAAATGGATAGATGATGATAATAAAACAGATTGGTTAAAAATAGCAATGAAT
SC50        AAAGCTATATTAAAAAATTACTGGGAAGATATGGAAATAAAATGGATAGATGATGATAATAAAACAGATTGGTTAAAAATAGCAATGAAT
SC56        AAAGCTATATTAAAAAATTACTGGGAAGATATGGAAATAAAATGGATAGATGATGATAATAAAACAGATTGGTTAAAAATAGCAATGAAT
TAB153      AAAGCTATATTAAAAAATTACTGGGAAGATATGGAAATAAAATGGATAGATGATGATAATAAAACAGATTGGTTAAAAATAGCAATGAAT
TAB154      AAAGCTATATTAAAAAATTACTGGGAAGATATGCAAATAAAATGGATAGATGATGATAATAAAACAGATTGGTTAAAAATAGCAATGAAT
TAB156      AAAGCTATATTAAAAAATTACTGGGAAGATATGCAAATAAAATGGATAGATGATGATAATAAAACAGATTGGTTAAAAATAGCAATGAAT
TAB136      AAAGCTATATTAAAAAATTACTGGGAAGATATGCAAATAAAATGGATAGATGATGATAATAAAACAGATTGGTTAAAAATAGCAATGAAT
AM1880      AAAGCTATATTAAAAAATTACTGGGAAGATATGCAAATAAAATGGATAGATGATGATAATAAAACAGATTGGTTAAAAATAGCAATGAAT
PF3D7_      AAAGCTATATTAAAAAATTACTGGGAAGATATGCAAATAAAATGGATAGATGATGATAATAAAACAGATTGGTTAAAAATAGCAATGAAT
PfSD01      AAAGCTATATTAAAAAATTACTGGGAAGATATGCAAATAAAATGGATAGATGATGATAATAAAACAGATTGGTTAAAAATAGCAATGAAT
Pf7G8       AAAGCTATATTAAAAAATTACTGGGAAGATATGCAAATAAAATGGATAGATGATGATAATAAAACAGATTGGTTAAAAATAGCAATGAAT
FCR3 GENE   AAAGCTATATTAAAAAATTACTGGGAAGATATGCAAATAAAATGGATAGATGATGATAATAAAACAGAATGGTTAAAAATAGCAATGAAT
MS822 GENE  ------------------------------------------------------------------------------------------
PfCD01_     AAAGCTATATTAAAAAATTACTGGGAAGATATGGAAATAAAATGGATAGATGATGATAATAAAACAGATTGGTTAAAAATAGCAATGAAT
PfKH01__    AAAGCTATATTAAAAAATTACTGGGAAGATATGCAAATAAAATGGATAGATGATGATAATAAAACAGATTGGTTAAAAATAGCAATGAAT
PfDd2__     AAAGCTATATTAAAAAATTACTGGGAAGATATGCAAATAAAATGGATAGATGATGATAATAAAACAGATTGGTTAAAAATAGCAATGAAT
PfSN01_     AAAGCTATATTAAAAAATTACTGGGAAGATATGCAAATAAAATGGATAGATGATGATAATAAAACAGATTGGTTAAAAATAGCAATGAAT
PfGN01      AAAGCTATATTAAAAAATTACTGGGAAGATATGCAAATAAAATGGATAGATGATGATAATAAAACAGATTGGTTAAAAATAGCAATGAAT
PfKE01      AAAGCTATATTAAAAAATTACTGGGAAGATATGCAAATAAAATGGATAGATGATGATAATAAAACAGATTGGTTAAAAATAGCAATGAAT
PfKH02      AAAGCTATATTAAAAAATTACTGGGAAGATATGGAAATAAAATGGATAGATGATGATAATAAAACAGATTGGTTAAAAATAGCAATGAAT
PfGB4       AAAGCTATATTAAAAAATTACTGGGAAGATATGGAAATAAAATGGATAGATGATGATAATAAAACAGATTGGTTAAAAATAGCAATGAAT
PfGA01_     AAAGCTATATTAAAAAATTACTGGGAAGATATGCAAATAAAATGGATAGATGATGATAATAAAACAGATTGGTTAAAAATAGCAATGAAT
PfHB3_      AAAGCTATATTAAAAAATTACTGGGAAGATATGGAAATAAAATGGATAGATGATGATAATAAAACAGATTGGTTAAAAATAGCAATGAAT
PfIT_       AAAGCTATATTAAAAAATTACTGGGAAGATATGCAAATAAAATGGATAGATGATGATAATAAAACAGATTGGTTAAAAATAGCAATGAAT
```

```
                    4960      4970      4980      4990      5000      5010      5020      5030      5040
            ....|....|....|....|....|....|....|....|....|....|....|....|....|....|....|....|....|....|
PF3D7 CDS   TTGAATAACTATGATAATAATAATTTTAGAAAAAATATAAAATATGAGCAAAAAAAAAATAATTTCAAAGAAGAAATCGAAATGATTAAG
MS822 mRNA  ------------------------------------------------------------------------------------------
FCR3 mRNA   TTGAATAACTATGATAATAATAATTTTAGAAAAAATATAAAATATGAGCAAAAAAAAAATAATTTCAAAGAAGAAATCGAAATGATTAAG
AM1898      TTGAATAACTATGATAATAATAATTTTAGAAAAAATATAAAATATGAGCAAAAAAAAAATAATTTCAAAGAAGAAATCGAAATGATTAAG
AA235       TTGAATAACTATGATAATAATAATTTTAGAAAAAATATAAAATATGAGCAAAAAAAAAATAATTTCAAAGAAGAAATCGAAATGATTAAG
AA243       TTGAATAACTATGATAATAATAATTTTAGAAAAAATATAAAATATGAGCAAAAAAAAAATAATTTCAAAGAAGAAATCGAAATGATTAAG
AA258       TTGAATAACTATGATAATAATAATTTTAGAAAAAATATAAAATATGAGCAAAAAAAAAATAATTTCAAAGAAGAAATCGAAATGATTAAG
AA264       TTGAATAACTATGATAATAATAATTTTAGAAAAAATATAAAATATGAGCAAAAAAAAAATAATTTCAAAGAAGAAATCGAAATGATTAAG
AM1737      TTGAATAACTATGATAATAATAATTTTAGAAAAAATATAAAATATGAGCAAAAAAAAAATAATTTCAAAGAAGAAATCGAAATGATTAAG
SC74        TTGAATAACTATGATAATAATAATTTTAGAAAAAATATAAAATATGAGCAAAAAAAAAATAATTTCAAAGAAGAAATCGAAATGATTAAG
TAB166      TTGAATAACTATGATAATAATAATTTTAGAAAAAATATAAAATATGAGCAAAAAAAAAATAATTTCAAAGAAGAAATCGAAATGATTAAG
TAB138      TTGAATAACTATGATAATAATAATTTTAGAAAAAATATAAAATATGAGCAAAAAAAAAATAATTTCAAAGAAGAAATCGAAATGATTAAG
TAB130      TTGAATAACTATGATAATAATAATTTTAGAAAAAATATAAAATATGAGCAAAAAAAAAATAATTTCAAAGAAGAAATCGAAATGATTAAG
TAB131      TTGAATAACTATGATAATAATAATTTTAGAAAAAATATAAAATATGAGCAAAAAAAAAATAATTTCAAAGAAGAAATCGAAATGATTAAG
TAB151      TTGAATAACTATGATAATAATAATTTTAGAAAAAATATAAAATATGAGCAAAAAAAAAATAATTTCAAAGAAGAAATCGAAATGATTAAG
TAB152      TTGAATAACTATGATAATAATAATTTTAGAAAAAATATAAAATATGAGCAAAAAAAAAATAATTTCAAAGAAGAAATCGAAATGATTAAG
SC52        TTGAATAACTATGATAATAATAATTTTAGAAAAAATATAAAATATGAGCAAAAAAAAAATAATTTCAAAGAAGAAATCGAAATGATTAAG
SC55        TTGAATAACTATGATAATAATAATTTTAGAAAAAATATAAAATATGAGCAAAAAAAAAATAATTTCAAAGAAGAAATCGAAATGATTAAG
SC03        TTGAATAACTATGATAATAATAATTTTAGAAAAAATATAAAATATGAGCAAAAAAAAAATAATTTCAAAGAAGAAATCGAAATGATTAAG
SC53        TTGAATAACTATGATAATAATAATTTTAGAAAAAATATAAAATATGAGCAAAAAAAAAATAATTTCAAAGAAGAAATCGAAATGATTAAG
TAB141      TTGAATAACTATGATAATAATAATTTTAGAAAAAATATAAAATATGAGCAAAAAAAAAATAATTTCAAAGAAGAAATCGAAATGATTAAG
TAB123      TTGAATAACTATGATAATAATAATTTTAGAAAAAATATAAAATATGAGCAAAAAAAAAATAATTTCAAAGAAGAAATCGAAATGATTAAG
AM1803      TTGAATAACTATGATAATAATAATTTTAGAAAAAATATAAAATATGAGCAAAAAAAAAATAATTTCAAAGAAGAAATCGAAATGATTAAG
AM1814      TTGAATAACTATGATAATAATAATTTTAGAAAAAATATAAAATATGAGCAAAAAAAAAATAATTTCAAAGAAGAAATCGAAATGATTAAG
AM1802      TTGAATAACTATGATAATAATAATTTTAGAAAAAATATAAAATATGAGCAAAAAAAAAATAATTTCAAAGAAGAAATCGAAATGATTAAG
AM1811      TTGAATAACTATGATAATAATAATTTTAGAAAAAATATAAAATATGAGCAAAAAAAAAATAATTTCAAAGAAGAAATCGAAATGATTAAG
SC50        TTGAATAACTATGATAATAATAATTTTAGAAAAAATATAAAATATGAGCAAAAAAAAAATAATTTCAAAGAAGAAATCGAAATGATTAAG
SC56        TTGAATAACTATGATAATAATAATTTTAGAAAAAATATAAAATATGAGCAAAAAAAAAATAATTTCAAAGAAGAAATCGAAATGATTAAG
TAB153      TTGAATAACTATGATAATAATAATTTTAGAAAAAATATAAAATATGAGCAAAAAAAAAATAATTTCAAAGAAGAAATCGAAATGATTAAG
TAB154      TTGAATAACTATGATAATAATAATTTTAGAAAAAATATAAAATATGAGCAAAAAAAAAATAATTTCAAAGAAGAAATCGAAATGATTAAG
TAB156      TTGAATAACTATGATAATAATAATTTTAGAAAAAATATAAAATATGAGCAAAAAAAAAATAATTTCAAAGAAGAAATCGAAATGATTAAG
TAB136      TTGAATAACTATGATAATAATAATTTTAGAAAAAATATAAAATATGAGCAAAAAAAAAATAATTTCAAAGAAGAAATCGAAATGATTAAG
AM1880      TTGAATAACTATGATAATAATAATTTTAGAAAAAATATAAAATATGAGCAAAAAAAAAATAATTTCAAAGAAGAAATCGAAATGATTAAG
PF3D7_      TTGAATAACTATGATAATAATAATTTTAGAAAAAATATAAAATATGAGCAAAAAAAAAATAATTTCAAAGAAGAAATCGAAATGATTAAG
PfSD01      TTGAATAACTATGATAATAATAATTTTAGAAAAAATATAAAATATGAGCAAAAAAAAAATAATTTCAAAGAAGAAATCGAAATGATTAAG
Pf7G8       TTGAATAACTATGATAATAATAATTTTAGAAAAAATATAAAATATGAGCAAAAAAAAAATAATTTCAAAGAAGAAATCGAAATGATTAAG
FCR3 GENE   TTGAATAACTATGATAATAATAATTTTAGAAAAAATATAAAATATGAGCAAAAAAAAAATAATTTCAAAGAAGAAATCGAAATGATTAAG
MS822 GENE  ------------------------------------------------------------------------------------------
PfCD01_     TTGAATAACTATGATAATAATAATTTTAGAAAAAATATAAAATATGAGCAAAAAAAAAATAATTTCAAAGAAGAAATCGAAATGATTAAG
PfKH01__    TTGAATAACTATGATAATAATAATTTTAGAAAAAATATAAAATATGAGCAAAAAAAAAATAATTTCAAAGAAGAAATCGAAATGATTAAG
PfDd2__     TTGAATAACTATGATAATAATAATTTTAGAAAAAATATAAAATATGAGCAAAAAAAAAATAATTTCAAAGAAGAAATCGAAATGATTAAG
PfSN01_     TTGAATAACTATGATAATAATAATTTTAGAAAAAATATAAAATATGAGCAAAAAAAAAATAATTTCAAAGAAGAAATCGAAATGATTAAG
PfGN01      TTGAATAACTATGATAATAATAATTTTAGAAAAAATATAAAATATGAGCAAAAAAAAAATAATTTCAAAGAAGAAATCGAAATGATTAAG
PfKE01      TTGAATAACTATGATAATAATAATTTTAGAAAAAATATAAAATATGAGCAAAAAAAAAATAATTTCAAAGAAGAAATCGAAATGATTAAG
PfKH02      TTGAATAACTATGATAATAATAATTTTAGAAAAAATATAAAATATGAGCAAAAAAAAAATAATTTCAAAGAAGAAATCGAAATGATTAAG
PfGB4       TTGAATAACTATGATAATAATAATTTTAGAAAAAATATAAAATATGAGCAAAAAAAAAATAATTTCAAAGAAGAAATCGAAATGATTAAG
PfGA01_     TTGAATAACTATGATAATAATAATTTTAGAAAAAATATAAAATATGAGCAAAAAAAAAATAATTTCAAAGAAGAAATCGAAATGATTAAG
PfHB3_      TTGAATAACTATGATAATAATAATTTTAGAAAAAATATAAAATATGAGCAAAAAAAAAATAATTTCAAAGAAGAAATCGAAATGATTAAG
PfIT_       TTGAATAACTATGATAATAATAATTTTAGAAAAAATATAAAATATGAGCAAAAAAAAAATAATTTCAAAGAAGAAATCGAAATGATTAAG
```

```
                5050      5060      5070      5080      5090      5100      5110      5120      5130
           ....|....|....|....|....|....|....|....|....|....|....|....|....|....|....|....|....|....|
PF3D7 CDS  AAAATTAATAAAAAGGCACAATCGAAAGATACTGAAGAAGATATCATGTGGAAAACTGTCATTGAAATACATATGAAAATAATAGAAGAA
MS822 mRNA ------------------------------------------------------------------------------------------
FCR3 mRNA  AAAATCAATAAAAAG---------------------------------------------------------------------------
AM1898     AAAATTAATAAAAAGGCACAATCGAAAGATACTGAAGAAGATATCATGTGGAAAACTGTCATTGAAATACATATGAAAATAATAGAAGAA
AA235      AAAATTAATAAAAAGGCACAATCGAAAGATACTGAAGAAGATATCATGTGGAAAACTGTCATTGAAATACATATGAAAATAATAGAAGAA
AA243      AAAATTAATAAAAAGGCACAATCGAAAGATACTGAAAAAGATATCATGTGGAAAACTGTCATTGAAATACATATGAAAATAATAGAAGAA
AA258      AAAATTAATAAAAAGGCACAATCGAAAGATACTGAAAAAGATATCATGTGGAAAACTGTCATTGAAATACATATGAAAATAATAGAAGAA
AA264      AAAATTAATAAAAAGGCACAATCGAAAGATACTGAAAAAGATATCATGTGGAAAACTGTCATTGAAATACATATGAAAATAATAGAAGAA
AM1737     AAAATTAATAAAAAGGCACAATCGAAAGATACTGAAAAAGATATCATGTGGAAAACTGTCATTGAAATACATATGAAAATAATAGAAGAA
SC74       AAAATTAATAAAAAGGCACAATCGAAAGATACTGAAAAAGATATCATGTGGAAAACTGTCATTGAAATACATATGAAAATAATAGAAGAA
TAB166     AAAATTAATAAAAAGGCACAATCGAAAGATACTGAAAAAGATATCATGTGGAAAACTGTCATTGAAATACATATGAAAATAATAGAAGAA
TAB138     AAAATTAATAAAAAGGCACAATCGAAAGATACTGAAAAAGATATCATGTGGAAAACTGTCATTGAAATACATATGAAAATAATAGAAGAA
TAB130     AAAATTAATAAAAAGGCACAATCGAAAGATACTGAAAAAGATATCATGTGGAAAACTGTCATTGAAATACATATGAAAATAATAGAAGAA
TAB131     AAAATTAATAAAAAGGCACAATCGAAAGATACTGAAGAAGATATCATGTGGAAAACTGTCATTGAAATACATATGAAAATAATAGAAGAA
TAB151     AAAATTAATAAAAAGGCACAATCGAAAGATACTGAAGAAGATATCATGTGGAAAACTGTCATTGAAATACATATGAAAATAATAGAAGAA
TAB152     AAAATTAATAAAAAGGCACAATCGAAAGATACTGAAGAAGATATCATGTGGAAAACTGTCATTGAAATACATATGAAAATAATAGAAGAA
SC52       AAAATTAATAAAAAGGCACAATCGAAAGATACTGAAGAAGATATCATGTGGAAAACTGTCATTGAAATACATATGAAAATAATAGAAGAA
SC55       AAAATTAATAAAAAGGCACAATCGAAAGATACTGAAGAAGATATCATGTGGAAAACTGTCATTGAAATACATATGAAAATAATAGAAGAA
SC03       AAAATTAATAAAAAGGCACAATCGAAAGATACTGAAGAAGATATCATGTGGAAAACTGTCATTGAAATACATATGAAAATAATAGAAGAA
SC53       AAAATTAATAAAAAGGCACAATCGAAAGATACTGAAGAAGATATCATGTGGAAAACTGTCATTGAAATACATATGAAAATAATAGAAGAA
TAB141     AAAATTAATAAAAAGGCACAATCGAAAGATACTGAAGAAGATATCATGTGGAAAACTGTCATTGAAATACATATGAAAATAATAGAAGAA
TAB123     AAAATTAATAAAAAGGCACAATCGAAAGATACTGAAAAAGATATCATGTGGAAAACTGTCATTGAAATACATATGAAAATAATAGAAGAA
AM1803     AAAATTAATAAAAAGGCACAATCGAAAGATACTGAAAAAGATATCATGTGGAAAACTGTCATTGAAATACATATGAAAATAATAGAAGAA
AM1814     AAAATTAATAAAAAGGCACAATCGAAAGATACTGAAGAAGATATCATGTGGAAAACTGTCATTGAAATACATATGAAAATAATAGAAGAA
AM1802     AAAATTAATAAAAAGGCACAATCGAAAGATACTGAAGAAGATATCATGTGGAAAACTGTCATTGAAATACATATGAAAATAATAGAAGAA
AM1811     AAAATTAATAAAAAGGCACAATCGAAAGATACTGAAAAAGATATCATGTGGAAAACTGTCATTGAAATACATATGAAAATAATAGAAGAA
SC50       AAAATTAATAAAAAGGCACAATCGAAAGATACTGAAAAAGATATCATGTGGAAAACTGTCATTGAAATACATATGAAAATAATAGAAGAA
SC56       AAAATTAATAAAAAGGCACAATCGAAAGATACTGAAAAAGATATCATGTGGAAAACTGTCATTGAAATACATATGAAAATAATAGAAGAA
TAB153     AAAATTAATAAAAAGGCACAATCGAAAGATACTGAAAAAGATATCATGTGGAAAACTGTCATTGAAATACATATGAAAATAATAGAAGAA
TAB154     AAAATTAATAAAAAGGCACAATCGAAAGATACTGAAGAAGATATCATGTGGAAAACTGTCATTGAAATACATATGAAAATAATAGAAGAA
TAB156     AAAATTAATAAAAAGGCACAATCGAAAGATACTGAAGAAGATATCATGTGGAAAACTGTCATTGAAATACATATGAAAATAATAGAAGAA
TAB136     AAAATTAATAAAAAGGCACAATCGAAAGATACTGAAAAAGATATCATGTGGAAAACTGTCATTGAAATACATATGAAAATAATAGAAGAA
AM1880     AAAATTAATAAAAAGGCACAATCGAAAGATACTGAAGAAGATATCATGTGGAAAACTGTCATTGAAATACATATGAAAATAATAGAAGAA
PF3D7_     AAAATTAATAAAAAGGCACAATCGAAAGATACTGAAGAAGATATCATGTGGAAAACTGTCATTGAAATACATATGAAAATAATAGAAGAA
PfSD01     AAAATTAATAAAAAGGCACAATCGAAAGATACTGAAAAAGATATCATGTGGAAAACTGTCATTGAAATACATATGAAAATAATAGAAGAA
Pf7G8      AAAATTAATAAAAAGGCACAATCGAAAGATACTGAAAAAGATATCATGTGGAAAACTGTCATTGAAATACATATGAAAATAATAGAAGAA
FCR3 GENE  AAAATCAATAAAAAG---------------------------------------------------------------------------
MS822 GENE ------------------------------------------------------------------------------------------
PfCD01_    AAAATTAATAAAAAGGCACAATCGAAAGATACTGAAAAAGATATCATGTGGAAAACTGTCATTGAAATACATATGAAAATAATAGAAGAA
PfKH01__   AAAATTAATAAAAAGGCACAATCGAAAGATACTGAAGAAGATATCATGTGGAAAACTGTCATTGAAATACATATGAAAATAATAGAAGAA
PfDd2__    AAAATTAATAAAAAGGCACAATCGAAAGATACTGAAAAAGATATCATGTGGAAAACTGTCATTGAAATACATATGAAAATAATAGAAGAA
PfSN01_    AAAATTAATAAAAAGGCACAATCGAAAGATACTGAAGAAGATATCATGTGGAAAACTGTCATTGAAATACATATGAAAATAATAGAAGAA
PfGN01     AAAATTAATAAAAAGGCACAATCGAAAGATACTGAAGAAGATATCATGTGGAAAACTGTCATTGAAATACATATGAAAATAATAGAAGAA
PfKE01     AAAATTAATAAAAAGGCACAATCGAAAGATACTGAAGAAGATATCATGTGGAAAACTGTCATTGAAATACATATGAAAATAATAGAAGAA
PfKH02     AAAATTAATAAAAAGGCACAATCGAAAGATACTGAAAAAGATATCATGTGGAAAACTGTCATTGAAATACATATGAAAATAATAGAAGAA
PfGB4      AAAATTAATAAAAAGGCACAATCGAAAGATACTGAAAAAGATATCATGTGGAAAACTGTCATTGAAATACATATGAAAATAATAGAAGAA
PfGA01_    AAAATTAATAAAAAGGCACAATCGAAAGATACTGAAAAAGATATCATGTGGAAAACTGTCATTGAAATACATATGAAAATAATAGAAGAA
PfHB3_     AAAATTAATAAAAAGGCACAATCGAAAGATACTGAAAAAGATATCATGTGGAAAACTGTCATTGAAATACATATGAAAATAATAGAAGAA
PfIT_      AAAATTAATAAAAAGGCACAATCGAAAGATACTGAAGAAGATATCATGTGGAAAACTGTCATTGAAATACATATGAAAATAATAGAAGAA
```

Sequence	5140 … 5220
PF3D7 CDS	GATAAAAAACAAGAATGGGAAAAAAACAAAGGAGATTTTTTACAAATTTGTAGGCAAGAATTTTTAAATGAAAATCAATATAATCGTAAT
MS822 mRNA	--
FCR3 mRNA	--
AM1898	GATAAAAAACAAGAATGGGAAAAAAACAAAGGAGATTTTTTACAAATTTGTAGGCAAGAATTTTTAAATGAAAATCAATATAATCGTAAT
AA235	GATAAAAAACAAGAATGGGAAAAAAACAAAGGAGATTTTTTACAAATTTGTAGGCAAGAATTTTTAAATGAAAATCAATATAATCGTAAT
AA243	GATAAAAAACAAGAATGGGAAAAAAACAAAGGAGATTTTTTACAAATTTGTAGGCAAGAATTTTTAAATGAAAATCAATATAATCGTAAT
AA258	GATAAAAAACAAGAATGGGAAAAAAACAAAGGAGATTTTTTACAAATTTGTAGGCAAGAATTTTTAAATGAAAATCAATATAATCGTAAT
AA264	GATAAAAAACAAGAATGGGAAAAAAACAAAGGAGATTTTTTACAAATTTGTAGGCAAGAATTTTTAAATGAAAATCAATATAATCGTAAT
AM1737	GATAAAAAACAAGAATGGGAAAAAAACAAAGGAGATTTTTTACAAATTTGTAGGCAAGAATTTTTAAATGAAAATCAATATAATCGTAAT
SC74	GATAAAAAACAAGAATGGGAAAAAAACAAAGGAGATTTTTTACAAATTTGTAGGCAAGAATTTTTAAATGAAAATCAATATAATCGTAAT
TAB166	GATAAAAAACAAGAATGGGAAAAAAACAAAGGAGATTTTTTACAAATTTGTAGGCAAGAATTTTTAAATGAAAATCAATATAATCGTAAT
TAB138	GATAAAAAACAAGAATGGGAAAAAAACAAAGGAGATTTTTTACAAATTTGTAGGCAAGAATTTTTAAATGAAAATCAATATAATCGTAAT
TAB130	GATAAAAAACAAGAATGGGAAAAAAACAAAGGAGATTTTTTACAAATTTGTAGGCAAGAATTTTTAAATGAAAATCAATATAATCGTAAT
TAB131	GATAAAAAACAAGAATGGGAAAAAAACAAAGGAGATTTTTTACAAATTTGTAGGCAAGAATTTTTAAATGAAAATCAATATAATCGTAAT
TAB151	GATAAAAAACAAGAATGGGAAAAAAACAAAGGAGATTTTTTACAAATTTGTAGGCAAGAATTTTTAAATGAAAATCAATATAATCGTAAT
TAB152	GATAAAAAACAAGAATGGGAAAAAAACAAAGGAGATTTTTTACAAATTTGTAGGCAAGAATTTTTAAATGAAAATCAATATAATCGTAAT
SC52	GATAAAAAACAAGAATGGGAAAAAAACAAAGGAGATTTTTTACAAATTTGTAGGCAAGAATTTTTAAATGAAAATCAATATAATCGTAAT
SC55	GATAAAAAACAAGAATGGGAAAAAAACAAAGGAGATTTTTTACAAATTTGTAGGCAAGAATTTTTAAATGAAAATCAATATAATCGTAAT
SC03	GATAAAAAACAAGAATGGGAAAAAAACAAAGGAGATTTTTTACAAATTTGTAGGCAAGAATTTTTAAATGAAAATCAATATAATCGTAAT
SC53	GATAAAAAACAAGAATGGGAAAAAAACAAAGGAGATTTTTTACAAATTTGTAGGCAAGAATTTTTAAATGAAAATCAATATAATCGTAAT
TAB141	GATAAAAAACAAGAATGGGAAAAAAACAAAGGAGATTTTTTACAAATTTGTAGGCAAGAATTTTTAAATGAAAATCAATATAATCGTAAT
TAB123	GATAAAAAACAAGAATGGGAAAAAAACAAAGGAGATTTTTTACAAATTTGTAGGCAAGAATTTTTAAATGAAAATCAATATAATCGTAAT
AM1803	GATAAAAAACAAGAATGGGAAAAAAACAAAGGAGATTTTTTACAAATTTGTAGGCAAGAATTTTTAAATGAAAATCAATATAATCGTAAT
AM1814	GATAAAAAACAAGAATGGGAAAAAAACAAAGGAGATTTTTTACAAATTTGTAGGCAAGAATTTTTAAATGAAAATCAATATAATCGTAAT
AM1802	GATAAAAAACAAGAATGGGAAAAAAACAAAGGAGATTTTTTACAAATTTGTAGGCAAGAATTTTTAAATGAAAATCAATATAATCGTAAT
AM1811	GATAAAAAACAAGAATGGGAAAAAAACAAAGGAGATTTTTTACAAATTTGTAGGCAAGAATTTTTAAATGAAAATCAATATAATCGTAAT
SC50	GATAAAAAACAAGAATGGGAAAAAAACAAAGGAGATTTTTTACAAATTTGTAGGCAAGAATTTTTAAATGAAAATCAATATAATCGTAAT
SC56	GATAAAAAACAAGAATGGGAAAAAAACAAAGGAGATTTTTTACAAATTTGTAGGCAAGAATTTTTAAATGAAAATCAATATAATCGTAAT
TAB153	GATAAAAAACAAGAATGGGAAAAAAACAAAGGAGATTTTTTACAAATTTGTAGGCAAGAATTTTTAAATGAAAATCAATATAATCGTAAT
TAB154	GATAAAAAACAAGAATGGGAAAAAAACAAAGGAGATTTTTTACAAATTTGTAGGCAAGAATTTTTAAATGAAAATCAATATAATCGTAAT
TAB156	GATAAAAAACAAGAATGGGAAAAAAACAAAGGAGATTTTTTACAAATTTGTAGGCAAGAATTTTTAAATGAAAATCAATATAATCGTAAT
TAB136	GTTAAAAAACAAGAATGGGAAAAAAACAAAGGAGATTTTTTACAAATTTGTAGGCAAGAATTTTTAAATGAAAATCAATATAATCGTAAT
AM1880	GATAAAAAACAAGAATGGGAAAAAAACAAAGGAGATTTTTTACAAATTTGTAGGCAAGAATTTTTAAATGAAAATCAATATAATCGTAAT
`PF3D7_`	GATAAAAAACAAGAATGGGAAAAAAACAAAGGAGATTTTTTACAAATTTGTAGGCAAGAATTTTTAAATGAAAATCAATATAATCGTAAT
PfSD01	GATAAAAAACAAGAATGGGAAAAAAACAAAGGAGATTTTTTACAAATTTGTAGGCAAGAATTTTTAAATGAAAATCAATATAATCGTAAT
Pf7G8	GATAAAAAACAAGAATGGGAAAAAAACAAAGGAGATTTTTTACAAATTTGTAGGCAAGAATTTTTAAATGAAAATCAATATAATCGTAAT
FCR3 GENE	--
MS822 GENE	--
`PfCD01_`	GATAAAAAACAAGAATGGGAAAAAAACAAAGGAGATTTTTTACAAATTTGTAGGCAAGAATTTTTAAATGAAAATCAATATAATCGTAAT
`PfKH01__`	GATAAAAAACAAGAATGGGAAAAAAACAAAGGAGATTTTTTACAAATTTGTAGGCAAGAATTTTTAAATGAAAATCAATATAATCGTAAT
`PfDd2__`	GATAAAAAACAAGAATGGGAAAAAAACAAAGGAGATTTTTTACAAATTTGTAGGCAAGAATTTTTAAATGAAAATCAATATAATCGTAAT
`PfSN01_`	GATAAAAAACAAGAATGGGAAAAAAACAAAGGAGATTTTTTACAAATTTGTAGGCAAGAATTTTTAAATGAAAATCAATATAATCGTAAT
PfGN01	GATAAAAAACAAGAATGGGAAAAAAACAAAGGAGATTTTTTACAAATTTGTAGGCAAGAATTTTTAAATGAAAATCAATATAATCGTAAT
PfKE01	GATAAAAAACAAGAATGGGAAAAAAACAAAGGAGATTTTTTACAAATTTGTAGGCAAGAATTTTTAAATGAAAATCAATATAATCGTAAT
PfKH02	GATAAAAAACAAGAATGGGAAAAAAACAAAGGAGATTTTTTACAAATTTGTAGGCAAGAATTTTTAAATGAAAATCAATATAATCGTAAT
PfGB4	GATAAAAAACAAGAATGGGAAAAAAACAAAGGAGATTTTTTACAAATTTGTAGGCAAGAATTTTTAAATGAAAATCAATATAATCGTAAT
`PfGA01_`	GATAAAAAACAAGAATGGGAAAAAAACAAAGGAGATTTTTTACAAATTTGTAGGCAAGAATTTTTAAATGAAAATCAATATAATCGTAAT
`PfHB3_`	GATAAAAAACAAGAATGGGAAAAAAACAAAGGAGATTTTTTACAAATTTGTAGGCAAGAATTTTTAAATGAAAATCAATATAATCGTAAT
`PfIT_`	GATAAAAAACAAGAATGGGAAAAAAACAAAGGAGATTTTTTACAAATTTGTAGGCAAGAATTTTTAAATGAAAATCAATATAATCGTAAT

```
                   5230      5240      5250      5260      5270      5280      5290      5300      5310
           ....|....|....|....|....|....|....|....|....|....|....|....|....|....|....|....|....|....|
PF3D7 CDS  GAAAACAGCCGTTTTGTAAATGATTATATGGTACAGGACAATTATATGATAAAACGACAAAATGGAATTTGGAATAAATGTATAGAAAGA
MS822 mRNA ------------------------------------------------------------------------------------------
FCR3 mRNA  ------------------------------------------------------------------------------------------
AM1898     GAAAACAGCCGTTTTGTAAATGATTATATGGTACAGGACAATTATATGATAAAACGACAAAATGGAATTTGGAATAAATGTATAGAAAGA
AA235      GAAAACAGCCGTTTTGTAAATGATTATATGGTACAGGACAATTATATGATAAAACGACAAAATGGAATTTGGAATAAATGTATAGAAAGA
AA243      GAAAACAGCCGTTTTGTAAATGATTATATGGTACAGGACAATTATATGATAAAACGACAAAATGGAATTTGGAATAAATGTATAGAAAGA
AA258      GAAAACAGCCGTTTTGTAAATGATTATATGGTACAGGACAATTATATGATAAAACGACAAAATGGAATTTGGAATAAATGTATAGAAAGA
AA264      GAAAACAGCCGTTTTGTAAATGATTATATGGTACAGGACAATTATATGATAAAACGACAAAATGGAATTTGGAATAAATGTATAGAAAGA
AM1737     GAAAACAGCCGTTTTGTAAATGATTATATGGTACAGGACAATTATATGATAAAACGACAAAATGGAATTTGGAATAAATGTATAGAAAGA
SC74       GAAAACAGCCGTTTTGTAAATGATTATATGGTACAGGACAATTATATGATAAAACGACAAAATGGAATTTGGAATAAATGTATAGAAAGA
TAB166     GAAAACAGCCGTTTTGTAAATGATTATATGGTACAGGACAATTATATGATAAAACGACAAAATGGAATTTGGAATAAATGTATAGAAAGA
TAB138     GAAAACAGCCGTTTTGTAAATGATTATATGGTACAGGACAATTATATGATAAAACGACAAAATGGAATTTGGAATAAATGTATAGAAAGA
TAB130     GAAAACAGCCGTTTTGTAAATGATTATATGGTACAGGACAATTATATGATAAAACGACAAAATGGAATTTGGAATAAATGTATAGAAAGA
TAB131     GAAAACAGCCGTTTTGTAAATGATTATATGGTACAGGACAATTATATGATAAAACGACAAAATGGAATTTGGAATAAATGTATAGAAAGA
TAB151     GAAAACAGCCGTTTTGTAAATGATTATATGGTACAGGACAATTATATGATAAAACGACAAAATGGAATTTGGAATAAATGTATAGAAAGA
TAB152     GAAAACAGCCGTTTTGTAAATGATTATATGGTACAGGACAATTATATGATAAAACGACAAAATGGAATTTGGAATAAATGTATAGAAAGA
SC52       GAAAACAGCCGTTTTGTAAATGATTATATGGTACAGGACAATTATATGATAAAACGACAAAATGGAATTTGGAATAAATGTATAGAAAGA
SC55       GAAAACAGCCGTTTTGTAAATGATTATATGGTACAGGACAATTATATGATAAAACGACAAAATGGAATTTGGAATAAATGTATAGAAAGA
SC03       GAAAACAGCCGTTTTGTAAATGATTATATGGTACAGGACAATTATATGATAAAACGACAAAATGGAATTTGGAATAAATGTATAGAAAGA
SC53       GAAAACAGCCGTTTTGTAAATGATTATATGGTACAGGACAATTATATGATAAAACGACAAAATGGAATTTGGAATAAATGTATAGAAAGA
TAB141     GAAAACAGCCGTTTTGTAAATGATTATATGGTACAGGACAATTATATGATAAAACGACAAAATGGAATTTGGAATAAATGTATAGAAAGA
TAB123     GAAAACAGCCGTTTTGTAAATGATTATATGGTACAGGACAATTATATGATAAAACGACAAAATGGAATTTGGAATAAATGTATAGAAAGA
AM1803     GAAAACAGCCGTTTTGTAAATGATTATATGGTACAGGACAATTATATGATAAAACGACAAAATGGAATTTGGAATAAATGTATAGAAAGA
AM1814     GAAAACAGCCGTTTTGTAAATGATTATATGGTACAGGACAATTATATGATAAAACGACAAAATGGAATTTGGAATAAATGTATAGAAAGA
AM1802     GAAAACAGCCGTTTTGTAAATGATTATATGGTACAGGACAATTATATGATAAAACGACAAAATGGAATTTGGAATAAATGTATAGAAAGA
AM1811     GAAAACAGCCGTTTTGTAAATGATTATATGGTACAGGACAATTATATGATAAAACGACAAAATGGAATTTGGAATAAATGTATAGAAAGA
SC50       GAAAACAGCCGTTTTGTAAATGATTATATGGTACAGGACAATTATATGATAAAACGACAAAATGGAATTTGGAATAAATGTATAGAAAGA
SC56       GAAAACAGCCGTTTTGTAAATGATTATATGGTACAGGACAATTATATGATAAAACGACAAAATGGAATTTGGAATAAATGTATAGAAAGA
TAB153     GAAAACAGCCGTTTTGTAAATGATTATATGGTACAGGACAATTATATGATAAAACGACAAAATGGAATTTGGAATAAATGTATAGAAAGA
TAB154     GAAAACAGCCGTTTTGTAAATGATTATATGGTACAGGACAATTATATGATAAAACGACAAAATGGAATTTGGAATAAATGTATAGAAAGA
TAB156     GAAAACAGCCGTTTTGTAAATGATTATATGGTACAGGACAATTATATGATAAAACGACAAAATGGAATTTGGAATAAATGTATAGAAAGA
TAB136     GAAAACAGCCGTTTTGTAAATGATTATATGGTACAGGACAATTATATGATAAAACGACAAAATGGAATTTGGAATAAATGTATAGAAAGA
AM1880     GAAAACAGCCGTTTTGTAAATGATTATATGGTACAGGACAATTATATGATAAAACGACAAAATGGAATTTGGAATAAATGTATAGAAAGA
PF3D7_     GAAAACAGCCGTTTTGTAAATGATTATATGGTACAGGACAATTATATGATAAAACGACAAAATGGAATTTGGAATAAATGTATAGAAAGA
PfSD01     GAAAACAGCCGTTTTGTAAATGATTATATGGTACAGGACAATTATATGATAAAACGACAAAATGGAATTTGGAATAAATGTATAGAAAGA
Pf7G8      GAAAACAGCCGTTTTGTAAATGATTATATGGTACAGGACAATTATATGATAAAACGACAAAATGGAATTTGGAATAAATGTATAGAAAGA
FCR3 GENE  ------------------------------------------------------------------------------------------
MS822 GENE ------------------------------------------------------------------------------------------
PfCD01_    GAAAACAGCCGTTTTGTAAATGATTATATGGTACAGGACAATTATATGATAAAACGACAAAATGGAATTTGGAATAAATGTATAGAAAGA
PfKH01__   GAAAACAGCCGTTTTGTAAATGATTATATGGTACAGGACAATTATATGATAAAACGACAAAATGGAATTTGGAATAAATGTATAGAAAGA
PfDd2__    GAAAACAGCCGTTTTGTAAATGATTATATGGTACAGGACAATTATATGATAAAACGACAAAATGGAATTTGGAATAAATGTATAGAAAGA
PfSN01_    GAAAACAGCCGTTTTGTAAATGATTATATGGTACAGGACAATTATATGATAAAACGACAAAATGGAATTTGGAATAAATGTATAGAAAGA
PfGN01     GAAAACAGCCGTTTTGTAAATGATTATATGGTACAGGACAATTATATGATAAAACGACAAAATGGAATTTGGAATAAATGTATAGAAAGA
PfKE01     GAAAACAGCCGTTTTGTAAATGATTATATGGTACAGGACAATTATATGATAAAACGACAAAATGGAATTTGGAATAAATGTATAGAAAGA
PfKH02     GAAAACAGCCGTTTTGTAAATGATTATATGGTACAGGACAATTATATGATAAAACGACAAAATGGAATTTGGAATAAATGTATAGAAAGA
PfGB4      GAAAACAGCCGTTTTGTAAATGATTATATGGTACAGGACAATTATATGATAAAACGACAAAATGGAATTTGGAATAAATGTATAGAAAGA
PfGA01_    GAAAACAGCCGTTTTGTAAATGATTATATGGTACAGGACAATTATATGATAAAACGACAAAATGGAATTTGGAATAAATGTATAGAAAGA
PfHB3_     GAAAACAGCCGTTTTGTAAATGATTATATGGTACAGGACAATTATATGATAAAACGACAAAATGGAATTTGGAATAAATGTATAGAAAGA
PfIT_      GAAAACAGCCGTTTTGTAAATGATTATATGGTACAGGACAATTATATGATAAAACGACAAAATGGAATTTGGAATAAATGTATAGAAAGA
```

```
                        5320      5330      5340      5350      5360      5370      5380      5390      5400
             ....|....|....|....|....|....|....|....|....|....|....|....|....|....|....|....|....|....|
PF3D7 CDS    AATAGATATATACTTGAAAAATGGAAGAAAGAAAAATGGTTTGAAAAATTAAAAAATCAATGGAAAAATGAACAAAATATATATGTAAAC
MS822 mRNA   ------------------------------------------------------------------------------------------
FCR3 mRNA    ------------------------------------------------------------------------------------------
AM1898       AATAGATATATACTTGAAAAATGGAAGAAAGAAAAATGGTTTGAAAAATTAAAAAATCAATGGAAAAATGAACAAAATATATATGTAAAC
AA235        AATAGATATATACTTGAAAAATGGAAGAAAGAAAAATGGTTTGAAAAATTAAAAAATCAATGGAAAAATGAACAAAATATATATGTAAAC
AA243        AATAGATATATACTTGAAAAATGGAAGAAAGAAAAATGGTTTGAAAAATTAAAAAATCAATGGAAAAATGAACAAAATATATATGTAAAC
AA258        AATAGATATATACTTGAAAAATGGAAGAAAGAAAAATGGTTTGAAAAATTAAAAAATCAATGGAAAAATGAACAAAATATATATGTAAAC
AA264        AATAGATATATACTTGAAAAATGGAAGAAAGAAAAATGGTTTGAAAAATTAAAAAATCAATGGAAAAATGAACAAAATATATATGTAAAC
AM1737       AATAGATATATACTTGAAAAATGGAAGAAAGAAAAATGGTTTGAAAAATTAAAAAATCAATGGAAAAATGAACAAAATATATATGTAAAC
SC74         AATAGATATATACTTGAAAAATGGAAGAAAGAAAAATGGTTTGAAAAATTAAAAAATGAATGGAAAAATGAACAAAATATATATGTAAAC
TAB166       AATAGATATATACTTGAAAAATGGAAGAAAGAAAAATGGTTTGAAAAATTAAAAAATGAATGGAAAAATGAACAAAATATATATGTAAAC
TAB138       AATAGATATATACTTGAAAAATGGAAGAAAGAAAAATGGTTTGAAAAATTAAAAAATGAATGGAAAAATGAACAAAATATATATGTAAAC
TAB130       AATAGATATATACTTGAAAAATGGAAGAAAGAAAAATGGTTTGAAAAATTAAAAAATGAATGGAAAAATGAACAAAATATATATGTAAAC
TAB131       AATAGATATATACTTGAAAAATGGAAGAAAGAAAAATGGTTTGAAAAATTAAAAAATCAATGGAAAAATGAACAAAATATATATGTAAAC
TAB151       AATAGATATATACTTGAAAAATGGAAGAAAGAAAAATGGTTTGAAAAATTAAAAAATCAATGGAAAAATGAACAAAATATATATGTAAAC
TAB152       AATAGATATATACTTGAAAAATGGAAGAAAGAAAAATGGTTTGAAAAATTAAAAAATCAATGGAAAAATGAACAAAATATATATGTAAAC
SC52         AATAGATATATACTTGAAAAATGGAAGAAAGAAAAATGGTTTGAAAAATTAAAAAATCAATGGAAAAATGAACAAAATATATATGTAAAC
SC55         AATAGATATATACTTGAAAAATGGAAGAAAGAAAAATGGTTTGAAAAATTAAAAAATCAATGGAAAAATGAACAAAATATATATGTAAAC
SC03         AATAGATATATACTTGAAAAATGGAAGAAAGAAAAATGGTTTGAAAAATTAAAAAATCAATGGAAAAATGAACAAAATATATATGTAAAC
SC53         AATAGATATATACTTGAAAAATGGAAGAAAGAAAAATGGTTTGAAAAATTAAAAAATCAATGGAAAAATGAACAAAATATATATGTAAAC
TAB141       AATAGATATATACTTGAAAAATGGAAGAAAGAAAAATGGTTTGAAAAATTAAAAAATGAATGGAAAAATGAACAAAATATATATGTAAAC
TAB123       AATAGATATATACTTGAAAAATGGAAGAAAGAAAAATGGTTTGAAAAATTAAAAAATGAATGGAAAAATGAACAAAATATATATGTAAAC
AM1803       AATAGATATATACTTGAAAAATGGAAGAAAGAAAAATGGTTTGAAAAATTAAAAAATCAATGGAAAAATGAACAAAATATATATGTAAAC
AM1814       AATAGATATATACTTGAAAAATGGAAGAAAGAAAAATGGTTTGAAAAATTAAAAAATCAATGGAAAAATGAACAAAATATATATGTAAAC
AM1802       AATAGATATATACTTGAAAAATGGAAGAAAGAAAAATGGTTTGAAAAATTAAAAAATGAATGGAAAAATGAACAAAATATATATGTAAAC
AM1811       AATAGATATATACTTGAAAAATGGAAGAAAGAAAAATGGTTTGAAAAATTAAAAAATCAATGGAAAAATGAACAAAATATATATGTAAAC
SC50         AATAGATATATACTTGAAAAATGGAAGAAAGAAAAATGGTTTGAAAAATTAAAAAATCAATGGAAAAATGAACAAAATATATATGTAAAC
SC56         AATAGATATATACTTGAAAAATGGAAGAAAGAAAAATGGTTTGAAAAATTAAAAAATCAATGGAAAAATGAACAAAATATATATGTAAAC
TAB153       AATAGATATATACTTGAAAAATGGAAGAAAGAAAAATGGTTTGAAAAATTAAAAAATCAATGGAAAAATGAACAAAATATATATGTAAAC
TAB154       AATAGATATATACTTGAAAAATGGAAGAAAGAAAAATGGTTTGAAAAATTAAAAAATCAATGGAAAAATGAACAAAATATATATGTAAAC
TAB156       AATAGATATATACTTGAAAAATGGAAGAAAGAAAAATGGTTTGAAAAATTAAAAAATGAATGGAAAAATGAACAAAATATATATGTAAAC
TAB136       AATAGATATATACTTGAAAAATGGAAGAAAGAAAAATGGTTTGAAAAATTAAAAAATCAATGGAAAAATGAACAAAATATATATGTAAAC
AM1880       AATAGATATATACTTGAAAAATGGAAGAAAGAAAAATGGTTTGAAAAATTAAAAAATCAATGGAAAAATGAACAAAATATATATGTAAAC
PF3D7_       AATAGATATATACTTGAAAAATGGAAGAAAGAAAAATGGTTTGAAAAATTAAAAAATCAATGGAAAAATGAACAAAATATATATGTAAAC
PfSD01       AATAGATATATACTTGAAAAATGGAAGAAAGAAAAATGGTTTGAAAAATTAAAAAATCAATGGAAAAATGAACAAAATATATATGTAAAC
Pf7G8        AATAGATATATACTTGAAAAATGGAAGAAAGAAAAATGGTTTGAAAAATTAAAAAATCAATGGAAAAATGAACAAAATATATATGTAAAC
FCR3 GENE    ------------------------------------------------------------------------------------------
MS822 GENE   ------------------------------------------------------------------------------------------
PfCD01_      AATAGATATATACTTGAAAAATGGAAGAAAGAAAAATGGTTTGAAAAATTAAAAAATCAATGGAAAAATGAACAAAATATATATGTAAAC
PfKH01__     AATAGATATATACTTGAAAAATGGAAGAAAGAAAAATGGTTTGAAAAATTAAAAAATCAATGGAAAAATGAACAAAATATATATGTAAAC
PfDd2__      AATAGATATATACTTGAAAAATGGAAGAAAGAAAAATGGTTTGAAAAATTAAAAAATCAATGGAAAAATGAACAAAATATATATGTAAAC
PfSN01_      AATAGATATATACTTGAAAAATGGAAGAAAGAAAAATGGTTTGAAAAATTAAAAAATCAATGGAAAAATGAACAAAATATATATGTAAAC
PfGN01       AATAGATATATACTTGAAAAATGGAAGAAAGAAAAATGGTTTGAAAAATTAAAAAATCAATGGAAAAATGAACAAAATATATATGTAAAC
PfKE01       AATAGATATATACTTGAAAAATGGAAGAAAGAAAAATGGTTTGAAAAATTAAAAAATCAATGGAAAAATGAACAAAATATATATGTAAAC
PfKH02       AATAGATATATACTTGAAAAATGGAAGAAAGAAAAATGGTTTGAAAAATTAAAAAATCAATGGAAAAATGAACAAAATATATATGTAAAC
PfGB4        AATAGATATATACTTGAAAAATGGAAGAAAGAAAAATGGTTTGAAAAATTAAAAAATGAATGGAAAAATGAACAAAATATATATGTAAAC
PfGA01_      AATAGATATATACTTGAAAAATGGAAGAAAGAAAAATGGTTTGAAAAATTAAAAAATGAATGGAAAAATGAACAAAATATATATGTAAAC
PfHB3_       AATAGATATATACTTGAAAAATGGAAGAAAGAAAAATGGTTTGAAAAATTAAAAAATGAATGGAAAAATGAACAAAATATATATGTAAAC
PfIT_        AATAGATATATACTTGAAAAATGGAAGAAAGAAAAATGGTTTGAAAAATTAAAAAATCAATGGAAAAATGAACAAAATATATATGTAAAC
```

```
                     5410      5420      5430      5440      5450      5460      5470      5480      5490
             ....|....|....|....|....|....|....|....|....|....|....|....|....|....|....|....|....|....|
PF3D7 CDS    ACACGTGAAGTATATTCGAATATTAATGATGAAACTAATGTAAAAGAAATAAACCCTTTAATAGAAGGTGAAAAGGTTTTGTGGAAAAAA
MS822 mRNA   ------------------------------------------------------------------------------------------
FCR3 mRNA    ------------------------------------------------------------------------------------------
AM1898       ACACGTGAAGTATATTCGAATATTAATGATGAAACTAATGTAAAAGAAATAAACCCTTTAATAGAAGGTGAAAAGGTTTTGTGGAAAAAA
AA235        ACACGTGAAGTATATTCGAATATTAATGATGAAACTAATGTAAAAGAAATAAACCCTTTAATAGAAGGTGAAAAGGTTTTGTGGAAAAAA
AA243        ACACGTGAAGTATATTCGAATATTAATGATGAAACTAATGTAAAAGAAATAAACCCTTTAATAGAAGGTGAAAAGGTTTTGTGGAAAAAA
AA258        ACACGTGAAGTATATTCGAATATTAATGATGAAACTAATGTAAAAGAAATAAACCCTTTAATAGAAGGTGAAAAGGTTTTGTGGAAAAAA
AA264        ACACGTGAAGTATATTCGAATATTAATGATGAAACTAATGTAAAAGAAATAAACCCTTTAATAGAAGGTGAAAAGGTTTTGTGGAAAAAA
AM1737       ACACGTGAAGTATATTCGAATATTAATGATGAAACTAATGTAAAAGAAATAAACCCTTTAATAGAAGGTGAAAAGGTTTTGTGGAAAAAA
SC74         ACACGTGAAGTATATTCGAATATTAATGATGAAACTAATGTAAAAGAAATAAACCCTTTAATAGAAGGTGAAAAGGTTTTGTGGAAAAAA
TAB166       ACACGTGAAGTATATTCGAATATTAATGATGAAACTAATGTAAAAGAAATAAACCCTTTAATAGAAGGTGAAAAGGTTTTGTGGAAAAAA
TAB138       ACACGTGAAGTATATTCGAATATTAATGATGAAACTAATGTAAAAGAAATAAACCCTTTAATAGAAGGTGAAAAGGTTTTGTGGAAAAAA
TAB130       ACACGTGAAGTATATTCGAATATTAATGATGAAACTAATGTAAAAGAAATAAACCCTTTAATAGAAGGTGAAAAGGTTTTGTGGAAAAAA
TAB131       ACACGTGAAGTATATTCGAATATTAATGATGAAACTAATGTAAAAGAAATAAACCCTTTAATAGAAGGTGAAAAGGTTTTGTGGAAAAAA
TAB151       ACACGTGAAGTATATTCGAATATTAATGATGAAACTAATGTAAAAGAAATAAACCCTTTAATAGAAGGTGAAAAGGTTTTGTGGAAAAAA
TAB152       ACACGTGAAGTATATTCGAATATTAATGATGAAACTAATGTAAAAGAAATAAACCCTTTAATAGAAGGTGAAAAGGTTTTGTGGAAAAAA
SC52         ACACGTGAAGTATATTCGAATATTAATGATGAAACTAATGTAAAAGAAATAAACCCTTTAATAGAAGGTGAAAAGGTTTTGTGGAAAAAA
SC55         ACACGTGAAGTATATTCGAATATTAATGATGAAACTAATGTAAAAGAAATAAACCCTTTAATAGAAGGTGAAAAGGTTTTGTGGAAAAAA
SC03         ACACGTGAAGTATATTCGAATATTAATGATGAAACTAATGTAAAAGAAATAAACCCTTTAATAGAAGGTGAAAAGGTTTTGTGGAAAAAA
SC53         ACACGTGAAGTATATTCGAATATTAATGATGAAACTAATGTAAAAGAAATAAACCCTTTAATAGAAGGTGAAAAGGTTTTGTGGAAAAAA
TAB141       ACACGTGAAGTATATTCGAATATTAATGATGAAACTAATGTAAAAGAAATAAACCCTTTAATAGAAGGTGAAAAGGTTTTGTGGAAAAAA
TAB123       ACACGTGAAGTATATTCGAATATTAATGATGAAACTAATGTAAAAGAAATAAACCCTTTAATAGAAGGTGAAAAGGTTTTGTGGAAAAAA
AM1803       ACACGTGAAGTATATTCGAATATTAATGATGAAACTAATGTAAAAGAAATAAACCCTTTAATAGAAGGTGAAAAGGTTTTGTGGAAAAAA
AM1814       ACACGTGAAGTATATTCGAATATTAATGATGAAACTAATGTAAAAGAAATAAACCCTTTAATAGAAGGTGAAAAGGTTTTGTGGAAAAAA
AM1802       ACACGTGAAGTATATTCGAATATTAATGATGAAACTAATGTAAAAGAAATAAACCCTTTAATAGAAGGTGAAAAGGTTTTGTGGAAAAAA
AM1811       ACACGTGAAGTATATTCGAATATTAATGATGAAACTAATGTAAAAGAAATAAACCCTTTAATAGAAGGTGAAAAGGTTTTGTGGAAAAAA
SC50         ACACGTGAAGTATATTCGAATATTAATGATGAAACTAATGTAAAAGAAATAAACCCTTTAATAGAAGGTGAAAAGGTTTTGTGGAAAAAA
SC56         ACACGTGAAGTATATTCGAATATTAATGATGAAACTAATGTAAAAGAAATAAACCCTTTAATAGAAGGTGAAAAGGTTTTGTGGAAAAAA
TAB153       ACACGTGAAGTATATTCGAATATTAATGATGAAACTAATGTAAAAGAAATAAACCCTTTAATAGAAGGTGAAAAGGTTTTGTGGAAAAAA
TAB154       ACACGTGAAGTATATTCGAATATTAATGATGAAACTAATGTAAAAGAAATAAACCCTTTAATAGAAGGTGAAAAGGTTTTGTGGAAAAAA
TAB156       ACACGTGAAGTATATTCGAATATTAATGATGAAACTAATGTAAAAGAAATAAACCCTTTAATAGAAGGTGAAAAGGTTTTGTGGAAAAAA
TAB136       ACACGTGAAGTATATTCGAATATTAATGATGAAACTAATGTAAAAGAAATAAACCCTTTAATAGAAGGTGAAAAGGTTTTGTGGAAAAAA
AM1880       ACACGTGAAGTATATTCGAATATTAATGATGAAACTAATGTAAAAGAAATAAACCCTTTAATAGAAGGTGAAAAGGTTTTGTGGAAAAAA
PF3D7_       ACACGTGAAGTATATTCGAATATTAATGATGAAACTAATGTAAAAGAAATAAACCCTTTAATAGAAGGTGAAAAGGTTTTGTGGAAAAAA
PfSD01       ACACGTGAAGTATATTCGAATATTAATGATGAAACTAATGTAAAAGAAATAAACCCTTTAATAGAAGGTGAAAAGGTTTTGTGGAAAAAA
Pf7G8        ACACGTGAAGTATATTCGAATATTAATGATGAAACTAATGTAAAAGAAATAAACCCTTTAATAGAAGGTGAAAAGGTTTTGTGGAAAAAA
FCR3 GENE    ------------------------------------------------------------------------------------------
MS822 GENE   ------------------------------------------------------------------------------------------
PfCD01_      ACACGTGAAGTATATTCGAATATTAATGATGAAACTAATGTAAAAGAAATAAACCCTTTAATAGAAGGTGAAAAGGTTTTGTGGAAAAAA
PfKH01__     ACACGTGAAGTATATTCGAATATTAATGATGAAACTAATGTAAAAGAAATAAACCCTTTAATAGAAGGTGAAAAGGTTTTGTGGAAAAAA
PfDd2__      ACACGTGAAGTATATTCGAATATTAATGATGAAACTAATGTAAAAGAAATAAACCCTTTAATAGAAGGTGAAAAGGTTTTGTGGAAAAAA
PfSN01_      ACACGTGAAGTATATTCGAATATTAATGATGAAACTAATGTAAAAGAAATAAACCCTTTAATAGAAGGTGAAAAGGTTTTGTGGAAAAAA
PfGN01       ACACGTGAAGTATATTCGAATATTAATGATGAAACTAATGTAAAAGAAATAAACCCTTTAATAGAAGGTGAAAAGGTTTTGTGGAAAAAA
PfKE01       ACACGTGAAGTATATTCGAATATTAATGATGAAACTAATGTAAAAGAAATAAACCCTTTAATAGAAGGTGAAAAGGTTTTGTGGAAAAAA
PfKH02       ACACGTGAAGTATATTCGAATATTAATGATGAAACTAATGTAAAAGAAATAAACCCTTTAATAGAAGGTGAAAAGGTTTTGTGGAAAAAA
PfGB4        ACACGTGAAGTATATTCGAATATTAATGATGAAACTAATGTAAAAGAAATAAACCCTTTAATAGAAGGTGAAAAGGTTTTGTGGAAAAAA
PfGA01_      ACACGTGAAGTATATTCGAATATTAATGATGAAACTAATGTAAAAGAAATAAACCCTTTAATAGAAGGTGAAAAGGTTTTGTGGAAAAAA
PfHB3_       ACACGTGAAGTATATTCGAATATTAATGATGAAACTAATGTAAAAGAAATAAACCCTTTAATAGAAGGTGAAAAGGTTTTGTGGAAAAAA
PfIT_        ACACGTGAAGTATATTCGAATATTAATGATGAAACTAATGTAAAAGAAATAAACCCTTTAATAGAAGGTGAAAAGGTTTTGTGGAAAAAA
```

```
                   5500      5510      5520      5530      5540      5550      5560      5570      5580
           ....|....|....|....|....|....|....|....|....|....|....|....|....|....|....|....|....|....|
PF3D7 CDS  TGGTTAAGAAATCAAAAGGGATTGTTAGATAAATATAATGAAGAGTTTTGGTTCAAAAAATTGTTTGAGGATTATGAAAAAGAAGTCGAA
MS822 mRNA ------------------------------------------------------------------------------------------
FCR3 mRNA  ------------------------------------------------------------------------------------------
AM1898     TGGTTAAGAAATCAAAAGGGATTGTTAGATAAATATAATGAAGAGTTTTGGTTCAAAAAATTGTTTGAGGATTATGAAAAAGAAGTCGAA
AA235      TGGTTAAGAAATCAAAAGGGATTGTTAGATAAATATAATGAAGAGTTTTGGTTCAAAAAATTGTTTGAGGATTATGAAAAAGAAGTCGAA
AA243      TGGTTAAGAAATCAAAAGGGATTGTTAGATAAATATAATGAAGAGTTTTGGTTCAAAAAATTGTTTGAGGATTATGAAAAAGAAGTCGAA
AA258      TGGTTAAGAAATCAAAAGGGATTGTTAGATAAATATAATGAAGAGTTTTGGTTCAAAAAATTGTTTGAGGATTATGAAAAAGAAGTCGAA
AA264      TGGTTAAGAAATCAAAAGGGATTGTTAGATAAATATAATGAAGAGTTTTGGTTCAAAAAATTGTTTGAGGATTATGAAAAAGAAGTCGAA
AM1737     TGGTTAAGAAATCAAAAGGGATTGTTAGAGAAATATAATGAAGAGTTTTGGTTCAAAAAATTGTTTGAGGATTATGAAAAAGAAGTCGAA
SC74       TGGTTAAGAAATCAAAAGGGATTGTTAGATAAATATAATGAAGAGTTTTGGTTCAAAAAATTGTTTGAGGATTATGAAAAAGAAGTCGAA
TAB166     TGGTTAAGAAATCAAAAGGGATTGTTAGAGAAATATAATGAAGAGTTTTGGTTCAAAAAATTGTTTGAGGATTATGAAAAAGAAGTCGAA
TAB138     TGGTTAAGAAATCAAAAGGGATTGTTAGATAAATATAATGAAGAGTTTTGGTTCAAAAAATTGTTTGAGGATTATGAAAAAGAAGTCGAA
TAB130     TGGTTAAGAAATCAAAAGGGATTGTTAGATAAATATAATGAAGAGTTTTGGTTCAAAAAATTGTTTGAGGATTATGAAAAAGAAGTCGAA
TAB131     TGGTTAAGAAATCAAAAGGGATTGTTAGAGAAATATAATGAAGAGTTTTGGTTCAAAAAATTGTTTGAGGATTATGAAAAAGAAGTCGAA
TAB151     TGGTTAAGAAATCAAAAGGGATTGTTAGAGAAATATAATGAAGAGTTTTGGTTCAAAAAATTGTTTGAGGATTATGAAAAAGAAGTCGAA
TAB152     TGGTTAAGAAATCAAAAGGGATTGTTAGATAAATATAATGAAGAGTTTTGGTTCAAAAAATTGTTTGAGGATTATGAAAAAGAAGTCGAA
SC52       TGGTTAAGAAATCAAAAGGGATTGTTAGATAAATATAATGAAGAGTTTTGGTTCAAAAAATTGTTTGAGGATTATGAAAAAGAAGTCGAA
SC55       TGGTTAAGAAATCAAAAGGGATTGTTAGATAAATATAATGAAGAGTTTTGGTTCAAAAAATTGTTTGAGGATTATGAAAAAGAAGTCGAA
SC03       TGGTTAAGAAATCAAAAGGGATTGTTAGATAAATATAATGAAGAGTTTTGGTTCAAAAAATTGTTTGAGGATTATGAAAAAGAAGTCGAA
SC53       TGGTTAAGAAATCAAAAGGGATTGTTAGATAAATATAATGAAGAGTTTTGGTTCAAAAAATTGTTTGAGGATTATGAAAAAGAAGTCGAA
TAB141     TGGTTAAGAAATCAAAAGGGATTGTTAGATAAATATAATGAAGAGTTTTGGTTCAAAAAATTGTTTGAGGATTATGAAAAAGAAGTCGAA
TAB123     TGGTTAAGAAATCAAAAGGGATTGTTAGAGAAATATAATGAAGAGTTTTGGTTCAAAAAATTGTTTGAGGATTATGAAAAAGAAGTCGAA
AM1803     TGGTTAAGAAATCAAAAGGGATTGTTAGATAAATATAATGAAGAGTTTTGGTTCAAAAAATTGTTTGAGGATTATGAAAAAGAAGTCGAA
AM1814     TGGTTAAGAAATCAAAAGGGATTGTTAGATAAATATAATGAAGAGTTTTGGTTCAAAAAATTGTTTGAGGATTATGAAAAAGAAGTCGAA
AM1802     TGGTTAAGAAATCAAAAGGGATTGTTAGATAAATATAATGAAGAGTTTTGGTTCAAAAAATTGGTTGAGGATTATGAAAAAGAAGTCGAA
AM1811     TGGTTAAGAAATCAAAAGGGATTGTTAGATAAATATAATGAAGAGTTTTGGTTCAAAAAATTGTTTGAGGATTATGAAAAAGAAGTCGAA
SC50       TGGTTAAGAAATCAAAAGGGATTGTTAGATAAATATAATGAAGAGTTTTGGTTCAAAAAATTGTTTGAGGATTATGAAAAAGAAGTCGAA
SC56       TGGTTAAGAAATCAAAAGGGATTGTTAGATAAATATAATGAAGAGTTTTGGTTCAAAAAATTGTTTGAGGATTATGAAAAAGAAGTCGAA
TAB153     TGGTTAAGAAATCAAAAGGGATTGTTAGATAAATATAATGAAGAGTTTTGGTTCAAAAAATTGTTTGAGGATTATGAAAAAGAAGTCGAA
TAB154     TGGTTAAGAAATCAAAAGGGATTGTTAGATAAATATAATGAAGAGTTTTGGTTCAAAAAATTGTTTGAGGATTATGAAAAAGAAGTCGAA
TAB156     TGGTTAAGAAATCAAAAGGGATTGTTAGATAAATATAATGAAGAGTTTTGGTTCAAAAAATTGGTTGAGGATTATGAAAAAGAAGTCGAA
TAB136     TGGTTAAGAAATCAAAAGGGATTGTTAGAGAAATATAATGAAGAGTTTTGGTTCAAAAAATTGTTTGAGGATTATGAAAAAGAAGTCGAA
AM1880     TGGTTAAGAAATCAAAAGGGATTGTTAGATAAATATAATGAAGAGTTTTGGTTCAAAAAATTGTTTGAGGATTATGAAAAAGAAGTCGAA
PF3D7_     TGGTTAAGAAATCAAAAGGGATTGTTAGATAAATATAATGAAGAGTTTTGGTTCAAAAAATTGTTTGAGGATTATGAAAAAGAAGTCGAA
PfSD01     TGGTTAAGAAATCGAAAGGGATTGTTAGATAAATATAATGAAGAGTTTTGGTTCAAAAAATTGGTTGAGGATTATGAAAAAGAAGTCGAA
Pf7G8      TGGTTAAGAAATCAAAAGGGATTGTTAGATAAATATAATGAAGAGTTTTGGTTCAAAAAATTGTTTGAGGATTATGAAAAAGAAGTCGAA
FCR3 GENE  ------------------------------------------------------------------------------------------
MS822 GENE ------------------------------------------------------------------------------------------
PfCD01_    TGGTTAAGAAATCAAAAGGGATTGTTAGATAAATATAATGAAGAGTTTTGGTTCAAAAAATTGGTTGAGGATTATGAAAAAGAAGTCGAA
PfKH01__   TGGTTAAGAAATCAAAAGGGATTGTTAGAGAAATATAATGAAGAGTTTTGGTTCAAAAAATTGTTTGAGGATTATGAAAAAGAAGTCGAA
PfDd2__    TGGTTAAGAAATCAAAAGGGATTGTTAGATAAATATAATGAAGAGTTTTGGTTCAAAAAATTGTTTGAGGATTATGAAAAAGAAGTCGAA
PfSN01_    TGGTTAAGAAATCAAAAGGGATTGTTAGATAAATATAATGAAGAGTTTTGGTTCAAAAAATTGGTTGAGGATTATGAAAAAGAAGTCGAA
PfGN01     TGGTTAAGAAATCAAAAGGGATTGTTAGATAAATATAATGAAGAGTTTTGGTTCAAAAAATTGTTTGAGGATTATGAAAAAGAAGTCGAA
PfKE01     TGGTTAAGAAATCAAAAGGGATTGTTAGATAAATATAATGAAGAGTTTTGGTTCAAAAAATTGTTTGAGGATTATGAAAAAGAAGTCGAA
PfKH02     TGGTTAAGAAATCAAAAGGGATTGTTAGAGAAATATAATGAAGAGTTTTGGTTCAAAAAATTGTTTGAGGATTATGAAAAAGAAGTCGAA
PfGB4      TGGTTAAGAAATCAAAAGGGATTGTTAGATAAATATAATGAAGAGTTTTGGTTCAAAAAATTGGTTGAGGATTATGAAAAAGAAGTCGAA
PfGA01_    TGGTTAAGAAATCAAAAGGGATTGTTAGATAAATATAATGAAGAGTTTTTGTTCAAAAAATTGTTTGAGGATTATGAAAAAGAAGTCGAA
PfHB3_     TGGTTAAGAAATCGAAAGGGATTGTTAGAGAAATATAATGAAGAGTTTTGGTTCAAAAAATTGTTTGAGGATTATGAAAAAGAAGTCGAA
PfIT_      TGGTTAAGAAATCAAAAGGGATTGTTAGATAAATATAATGAAGAGTTTTGGTTCAAAAAATTGTTTGAGGATTATGAAAAAGAAGTCGAA
```

```
                   5590      5600      5610      5620      5630      5640      5650      5660      5670
              ....|....|....|....|....|....|....|....|....|....|....|....|....|....|....|....|....|....|
PF3D7 CDS     AA------------TGATGATGATGATGAT-----------------------TATTATTATTATCATATGAGTGTAAATCAACAAGGAA
MS822 mRNA    ------------------------------------------------------------------------------------------
FCR3 mRNA     ------------------------------------------------------------------------------------------
AM1898        AA------------TGATGATGATGATGAT-----------------------TATTATTATTATCATATGAGTGTAAATCAACAAGGAA
AA235         AA------------TGATGATGATGATGAT-----------------------TATTATTATTATCATATGAGTGTAAATCAACAAGGAA
AA243         AA------------TGATGATGATGATGAT-----------------------TATTATTATTATCATATGAGTGTAAATCAACAAGGAA
AA258         AA------------TGATGATGATGATGAT-----------------------TATTATTATTATCATATGAGTGTAAATCAACAAGGAA
AA264         AA------------TGATGATGATGATGAT-----------------------TATTATTATTATCATATGAGTGTAAATCAACAAGGAA
AM1737        AA------------TGATGATGATGATGAT-----------------------TATTATTATTATCATATGAGTGTAAATCAACAAGGAA
SC74          AA------------TGATGATGATGATGAT-----------------------TATTATTATTATCATATGAGTGTAAATCAACAAGGAA
TAB166        AA------------TGATGATGATGATGAT-----------------------TATTATTATTATCATATGAGTGTAAATCAACAAGGAA
TAB138        AA------------TGATGATGATGATGAT-----------------------TATTATTATTATCATATGAGTGTAAATCAACAAGGAA
TAB130        AA------------TGATGATGATGATGAT-----------------------TATTATTATTATCATATGAGTGTAAATCAACAAGGAA
TAB131        AA------------TGATGATGATGATGAT-----------------------TATTATTATTATCATATGAGTGTAAATCAACAAGGAA
TAB151        AA------------TGATGATGATGATGAT-----------------------TATTATTATTATCATATGAGTGTAAATCAACAAGGAA
TAB152        AA------------TGATGATGATGATGAT-----------------------TATTATTATTATCATATGAGTGTAAATCAACAAGGAA
SC52          AA------------TGATGATGATGATGAT-----------------------TATTATTATTATCATATGAGTGTAAATCAACAAGGAA
SC55          AA------------TGATGATGATGATGAT-----------------------TATTATTATTATCATATGAGTGTAAATCAACAAGGAA
SC03          AA------------TGATGATGATGATGAT-----------------------TATTATTATTATCATATGAGTGTAAATCAACAAGGAA
SC53          AA------------TGATGATGATGATGAT-----------------------TATTATTATTATCATATGAGTGTAAATCAACAAGGAA
TAB141        AA------------TGATGATGATGATGAT-----------------------TATTATTATTATCATATGAGTGTAAATCAACAAGGAA
TAB123        AA------------TGATGATGATGATGAT-----------------------TATTATTATTATCATATGAGTGTAAATCAACAAGGAA
AM1803        AA------------TGATGATGATGATGAT-----------------------TATTATTATTATCATATGAGTGTAAATCAACAAGGAA
AM1814        AA------------TGATGATGATGATGAT-----------------------TATTATTATTATCATATGAGTGTAAATCAACAAGGAA
AM1802        AA------------TGATGATGATGATGATGAT--------------------TATTATTATTATCATATGAGTGTAAATCAACAAGGAA
AM1811        AA------------TGATGATGATGATGAT-----------------------TATTATTATTATCATATGAGTGTAAATCAACAAGGAA
SC50          AA------------TGATGATGATGATGAT-----------------------TATTATTATTATCATATGAGTGTAAATCAACAAGGAA
SC56          AA------------TGATGATGATGATGAT-----------------------TATTATTATTATCATATGAGTGTAAATCAACAAGGAA
TAB153        AA------------TGATGATGATGATGAT-----------------------TATTATTATTATCATATGAGTGTAAATCAACAAGGAA
TAB154        AA------------TGATGATGATGATGAT-----------------------TATTATTATTATCATATGAGTGTAAATCAACAAGGAA
TAB156        AA------------TGATGATGATGATGATGAT--------------------TATTATTATTATCATATGAGTGTAAATCAACAAGGAA
TAB136        AA------------TGATGATGATGATGAT-----------------------TATTATTATTATCATATGAGTGTAAATCAACAAGGAA
AM1880        AA------------TGATGATGATGATGAT-----------------------TATTATTATTATCATATGAGTGTAAATCAACAAGGAA
PF3D7_        AA------------TGATGATGATGATGAT-----------------------TATTATTATTATCATATGAGTGTAAATCAACAAGGAA
PfSD01        AA------------TGATGATGAAGATGATGATGACAATGATGATTATTATCT--------------TATGAGTGTAAATCAACAAGGAA
Pf7G8         AA------------TGATGATGATGATGAT-----------------------TATTATTATTATCATATGAGTGTAAATCAACAAGGAA
FCR3 GENE     ------------------------------------------------------------------------------------------
MS822 GENE    ------------------------------------------------------------------------------------------
PfCD01_       AA------------TGATGATGAAGATGATGATGACAATGATGATTATTATCT--------------TATGAGTGTAAATCAACAAGGAA
PfKH01__      AA------------TGATGATGATGATGAT-----------------------TATTATTATTATCATATGAGTGTAAATCAACAAGGAA
PfDd2__       AA------------TGATGATGATGATGAT-----------------------TATTATTATTATCATATGAGTGTAAATCAACAAGGAA
PfSN01_       AA------------TGATGATGATGATGAT-----------------------TATTATTATTATCATATGAGTGTAAATCAACAAGGAA
PfGN01        AA------------TGATGATGATGATGAT-----------------------TATTATTATTATCATATGAGTGTAAATCAACAAGGAA
PfKE01        AA------------TGATGATGATGATGAT-----------------------TATTATTATTATCATATGAGTGTAAATCAACAAGGAA
PfKH02        AA------------TGATGATGATGATGAT-----------------------TATTATTATTATCATATGAGTGTAAATCAACAAGGAA
PfGB4         AA------------TGATGATGATGATGAT-----------------------TATTATTATTATCATATGAGTGTAAATCAACAAGGAA
PfGA01_       AAAGAAGTCGAAAATGATGATGATGATGAT-----------------------TATTATTATTATCATATGAGTGTAAATCAACAAGGAA
PfHB3_        AA------------TGATGATGATGATGAT-----------------------TATTATTATTATCATATGAGTGTAAATCAACAAGGAA
PfIT_         AA------------TGATGATGATGATGAT-----------------------TATTATTATTATCATATGAGTGTAAATCAACAAGGAA
```

```
                       5680      5690      5700      5710      5720      5730      5740      5750      5760
               ....|....|....|....|....|....|....|....|....|....|....|....|....|....|....|....|....|....|
PF3D7 CDS      AACACCCAGAAGACTTAATTACATTAAATAAAATATCTAATCAATCGTTACAAAAATTCAAAAAGAACAAATTAATAACGACAATGTGGA
MS822 mRNA     ------------------------------------------------------------------------------------------
FCR3 mRNA      ------------------------------------------------------------------------------------------
AM1898         AACACCCAGAAGACTTAATTACATTAAATAAAATATCTAATCAATCGTTACAAAAATTCAAAAAGAACAAATTAATAACGACAATGTGGA
AA235          AACACCCAGAAGACTTAATTACATTAAATAAAATATCTAATCAATCGTTACAAAAATTCAAAAAGAACAAATTAATAACGACAATGTGGA
AA243          AACACCCAGAAGACTTAATTACATTAAATAAAATATCTAATCAATCGTTACAAAAATTCAAAAAGAACAAATTAATAACGACAATGTGGA
AA258          AACACCCAGAAGACTTAATTACATTAAATAAAATATCTAATCAATCGTTACAAAAATTCAAAAAGAACAAATTAATAACGACAATGTGGA
AA264          AACACCCAGAAGACTTAATTACATTAAATAAAATATCTAATCAATCGTTACAAAAATTCAAAAAGAACAAATTAATAACGACAATGTGGA
AM1737         AACACCCAGAAGACTTAATTACATTAAATAAAATATCTAATCAATCGTTACAAAAATTCAAAAAGAACAAATTAATAACGACAATGTGGA
SC74           AACACCCAGAAGACTTAATTACATTAAATAAAATATCTAATCAATCGTTACAAAAATTCAAAAAGAACAAATTAATAACGACAATGTGGA
TAB166         AACACCCAGAAGACTTAATTACATTAAATAAAATATCTAATCAATCGTTACAAAAATTCAAAAAGAACAAATTAATAACGACAATGTGGA
TAB138         AACACCCAGAAGACTTAATTACATTAAATAAAATATCTAATCAATCGTTACAAAAATTCAAAAAGAACAAATTAATAACGACAATGTGGA
TAB130         AACACCCAGAAGACTTAATTACATTAAATAAAATATCTAATCAATCGTTACAAAAATTCAAAAAGAACAAATTAATAACGACAATGTGGA
TAB131         AACACCCAGAAGACTTAATTACATTAAATAAAATATCTAATCAATCGTTACAAAAATTCAAAAAGAACAAATTAATAACGACAATGTGGA
TAB151         AACACCCAGAAGACTTAATTACATTAAATAAAATATCTAATCAATCGTTACAAAAATTCAAAAAGAACAAATTAATAACGACAATGTGGA
TAB152         AACACCCAGAAGACTTAATTACATTAAATAAAATATCTAATCAATCGTTACAAAAATTCAAAAAGAACAAATTAATAACGACAATGTGGA
SC52           AACACCCAGAAGACTTAATTACATTAAATAAAATATCTAATCAATCGTTACAAAAATTCAAAAAGAACAAATTAATAACGACAATGTGGA
SC55           AACACCCAGAAGACTTAATTACATTAAATAAAATATCTAATCAATCGTTACAAAAATTCAAAAAGAACAAATTAATAACGACAATGTGGA
SC03           AACACCCAGAAGACTTAATTACATTAAATAAAATATCTAATCAATCGTTACAAAAATTCAAAAAGAACAAATTAATAACGACAATGTGGA
SC53           AACACCCAGAAGACTTAATTACATTAAATAAAATATCTAATCAATCGTTACAAAAATTCAAAAAGAACAAATTAATAACGACAATGTGGA
TAB141         AACACCCAGAAGACTTAATTACATTAAATAAAATATCTAATCAATCGTTACAAAAATTCAAAAAGAACAAATTAATAACGACAATGTGGA
TAB123         AACACCCAGAAGACTTAATTACATTAAATAAAATATCTAATCAATCGTTACAAAAATTCAAAAAGAACAAATTAATAACGACAATGTGGA
AM1803         AACACCCAGAAGACTTAATTACATTAAATAAAATATCTAATCAATCGTTACAAAAATTCAAAAAGAACAAATTAATAACGACAATGTGGA
AM1814         AACACCCAGAAGACTTAATTACATTAAATAAAATATCTAATCAATCGTTACAAAAATTCAAAAAGAACAAATTAATAACGACAATGTGGA
AM1802         AACACCCAGAAGACTTAATTACATTAAATAAAATATCTAATCAATCGTTACAAAAATTCAAAAAGAACAAATTAATAACGACAATGTGGA
AM1811         AACACCCAGAAGACTTAATTACATTAAATAAAATATCTAATCAATCGTTACAAAAATTCAAAAAGAACAAATTAATAACGACAATGTGGA
SC50           AACACCCAGAAGACTTAATTACATTAAATAAAATATCTAATCAATCGTTACAAAAATTCAAAAAGAACAAATTAATAACGACAATGTGGA
SC56           AACACCCAGAAGACTTAATTACATTAAATAAAATATCTAATCAATCGTTACAAAAATTCAAAAAGAACAAATTAATAACGACAATGTGGA
TAB153         AACACCCAGAAGACTTAATTACATTAAATAAAATATCTAATCAATCGTTACAAAAATTCAAAAAGAACAAATTAATAACGACAATGTGGA
TAB154         AACACCCAGAAGACTTAATTACATTAAATAAAATATCTAATCAATCGTTACAAAAATTCAAAAAGAACAAATTAATAACGACAATGTGGA
TAB156         AACACCCAGAAGACTTAATTACATTAAATAAAATATCTAATCAATCGTTACAAAAATTCAAAAAGAACAAATTAATAACGACAATGTGGA
TAB136         AACACCCAGAAGACTTAATTACATTAAATAAAATATCTAATCAATCGTTACAAAAATTCAAAAAGAACAAATTAATAACGACAATGTGGA
AM1880         AACACCCAGAAGACTTAATTACATTAAATAAAATATCTAATCAATCGTTACAAAAATTCAAAAAGAACAAATTAATAACGACAATGTGGA
PF3D7_         AACACCCAGAAGACTTAATTACATTAAATAAAATATCTAATCAATCGTTACAAAAATTCAAAAAGAACAAATTAATAACGACAATGTGGA
PfSD01         AACACCCAGAAGACTTAATTACATTAAATAAAATATCTAATCAATCGTTACAAAAATTCAAAAAGAACAAATTAATAACGACAATGTGGA
Pf7G8          AACACCCAGAAGACTTAATTACATTAAATAAAATATCTAATCAATCGTTACAAAAATTCAAAAAGAACAAATTAATAACGACAATGTGGA
FCR3 GENE      ------------------------------------------------------------------------------------------
MS822 GENE     ------------------------------------------------------------------------------------------
PfCD01_        AACATCCAGAAGACTTAATTACATTAAATAAAATATCTAATCAATCGTTACAAAAATTCAAAAAGAACAAATTAATAACGACAATGTGGA
PfKH01__       AACACCCAGAAGACTTAATTACATTAAATAAAATATCTAATCAATCGTTACAAAAATTCAAAAAGAACAAATTAATAACGACAATGTGGA
PfDd2__        AACACCCAGAAGACTTAATTACATTAAATAAAATATCTAATCAATCGTTACAAAAATTCAAAAAGAACAAATTAATAACGACAATGTGGA
PfSN01_        AACACCCAGAAGACTTAATTACATTAAATAAAATATCTAATCAATCGTTACAAAAATTCAAAAAGAACAAATTAATAACGACAATGTGGA
PfGN01         AACACCCAGAAGACTTAATTACATTAAATAAAATATCTAATCAATCGTTACAAAAATTCAAAAAGAACAAATTAATAACGACAATGTGGA
PfKE01         AACACCCAGAAGACTTAATTACATTAAATAAAATATCTAATCAATCGTTACAAAAATTCAAAAAGAACAAATTAATAACGACAATGTGGA
PfKH02         AACACCCAGAAGACTTAATTACATTAAATAAAATATCTAATCAATCGTTACAAAAATTCAAAAAGAACAAATTAATAACGACAATGTGGA
PfGB4          AACACCCAGAAGACTTAATTACATTAAATAAAATATCTAATCAATCGTTACAAAAATTCAAAAAGAACAAATTAATAACGACAATGTGGA
PfGA01_        AACACCCAGAAGACTTAATTACATTAAATAAAATATCTAATCAATCGTTACAAAAATTCAAAAAGAACAAATTAATAACGACAATGTGGA
PfHB3_         AACACCCAGAAGACTTAATTACATTAAATAAAATATCTAATCAATCGTTACAAAAATTCAAAAAGAACAAATTAATAACGACAATGTGGA
PfIT_          AACACCCAGAAGACTTAATTACATTAAATAAAATATCTAATCAATCGTTACAAAAATTCAAAAAGAACAAATTAATAACGACAATGTGGA
```

```
                  5770      5780      5790      5800      5810      5820      5830      5840      5850
             ....|....|....|....|....|....|....|....|....|....|....|....|....|....|....|....|....|....|
PF3D7 CDS    TAGAGATACACATGATGATATTAGAAGAATGTAAAGAGGAGGAAGTTCAATTGAACAAGGAATTGTTTTTGGATTCTTGTATAAAGGAAT
MS822 mRNA   ------------------------------------------------------------------------------------------
FCR3 mRNA    ------------------------------------------------------------------------------------------
AM1898       TAGAGATACACATGATGATATTAGAAGAATGTAAAGAGGAGGAAGTTCAATTGAACAAGGAATTGTTTTTGGATTCTTGTATAAAGGAAT
AA235        TAGAGATACACATGATGATATTAGAAGAATGTAAAGAGGAGGAAGTTCAATTGAACAAGGAATTGTTTTTGGATTCTTGTATAAAGGAAT
AA243        TAGAGATACACATGATGATATTAGAAGAATGTAAAGAGGAGGAAGTTCAATTGAACAAGGAATTGTTTTTGGATTCTTGTATAAAGGAAT
AA258        TAGAGATACACATGATGATATTAGAAGAATGTAAAGAGGAGGAAGTTCAATTGAACAAGGAATTGTTTTTGGATTCTTGTATAAAGGAAT
AA264        TAGAGATACACATGATGATATTAGAAGAATGTAAAGAGGAGGAAGTTCAATTGAACAAGGAATTGTTTTTGGATTCTTGTATAAAGGAAT
AM1737       TAGAGATACACATGATGATATTAGAAGAATGTAAAGAGGAGGAAGTTCAATTGAACAAGGAATTGTTTTTGGATTCTTGTATAAAGGAAT
SC74         TAGAGATACACATGATGATATTAGAAGAATGTAAAGAGGAGGAAGTTCAATTGAACAAGGAATTGTTTTTGGATTCTTGTATAAAGGAAT
TAB166       TAGAGATACACATGATGATATTAGAAGAATGTAAAGAGGAGGAAGTTCAATTGAACAAGGAATTGTTTTTGGATTCTTGTATAAAGGAAT
TAB138       TAGAGATACACATGATGATATTAGAAGAATGTAAAGAGGAGGAAGTTCAATTGAACAAGGAATTGTTTTTGGATTCTTGTATAAAGGAAT
TAB130       TAGAGATACACATGATGATATTAGAAGAATGTAAAGAGGAGGAAGTTCAATTGAACAAGGAATTGTTTTTGGATTCTTGTATAAAGGAAT
TAB131       TAGAGATACACATGATGATATTAGAAGAATGTAAAGAGGAGGAAGTTCAATTGAACAAGGAATTGTTTTTGGATTCTTGTATAAAGGAAT
TAB151       TAGAGATACACATGATGATATTAGAAGAATGTAAAGAGGAGGAAGTTCAATTGAACAAGGAATTGTTTTTGGATTCTTGTATAAAGGAAT
TAB152       TAGAGATACACATGATGATATTAGAAGAATGTAAAGAGGAGGAAGTTCAATTGAACAAGGAATTGTTTTTGGATTCTTGTATAAAGGAAT
SC52         TAGAGATACACATGATGATATTAGAAGAATGTAAAGAGGAGGAAGTTCAATTGAACAAGGAATTGTTTTTGGATTCTTGTATAAAGGAAT
SC55         TAGAGATACACATGATGATATTAGAAGAATGTAAAGAGGAGGAAGTTCAATTGAACAAGGAATTGTTTTTGGATTCTTGTATAAAGGAAT
SC03         TAGAGATACACATGATGATATTAGAAGAATGTAAAGAGGAGGAAGTTCAATTGAACAAGGAATTGTTTTTGGATTCTTGTATAAAGGAAT
SC53         TAGAGATACACATGATGATATTAGAAGAATGTAAAGAGGAGGAAGTTCAATTGAACAAGGAATTGTTTTTGGATTCTTGTATAAAGGAAT
TAB141       TAGAGATACACATGATGATATTAGAAGAATGTAAAGAGGAGGAAGTTCAATTGAACAAGGAATTGTTTTTGGATTCTTGTATAAAGGAAT
TAB123       TAGAGATACACATGATGATATTAGAAGAATGTAAAGAGGAGGAAGTTCAATTGAACAAGGAATTGTTTTTGGATTCTTGTATAAAGGAAT
AM1803       TAGAGATACACATGATGATATTAGAAGAATGTAAAGAGGAGGAAGTTCAATTGAACAAGGAATTGTTTTTGGATTCTTGTATAAAGGAAT
AM1814       TAGAGATACACATGATGATATTAGAAGAATGTAAAGAGGAGGAAGTTCAATTGAACAAGGAATTGTTTTTGGATTCTTGTATAAAGGAAT
AM1802       TAGAGATACACATGATGATATTAGAAGAATGTAAAGAGGAGGAAGTTCAATTGAACAAGGAATTGTTTTTGGATTCTTGTATAAAGGAAT
AM1811       TAGAGATACACATGATGATATTAGAAGAATGTAAAGAGGAGGAAGTTCAATTGAACAAGGAATTGTTTTTGGATTCTTGTATAAAGGAAT
SC50         TAGAGATACACATGATGATATTAGAAGAATGTAAAGAGGAGGAAGTTCAATTGAACAAGGAATTGTTTTTGGATTCTTGTATAAAGGAAT
SC56         TAGAGATACACATGATGATATTAGAAGAATGTAAAGAGGAGGAAGTTCAATTGAACAAGGAATTGTTTTTGGATTCTTGTATAAAGGAAT
TAB153       TAGAGATACACATGATGATATTAGAAGAATGTAAAGAGGAGGAAGTTCAATTGAACAAGGAATTGTTTTTGGATTCTTGTATAAAGGAAT
TAB154       TAGAGATACACATGATGATATTAGAAGAATGTAAAGAGGAGGAAGTTCAATTGAACAAGGAATTGTTTTTGGATTCTTGTATAAAGGAAT
TAB156       TAGAGATACACATGATGATATTAGAAGAATGTAAAGAGGAGGAAGTTCAATTGAACAAGGAATTGTTTTTGGATTCTTGTATAAAGGAAT
TAB136       TAGAGATACACATGATGATATTAGAAGAATGTAAAGAGGAGGAAGTTCAATTGAACAAGGAATTGTTTTTGGATTCTTGTATAAAGGAAT
AM1880       TAGAGATACACATGATGATATTAGAAGAATGTAAAGAGGAGGAAGTTCAATTGAACAAGGAATTGTTTTTGGATTCTTGTATAAAGGAAT
PF3D7_       TAGAGATACACATGATGATATTAGAAGAATGTAAAGAGGAGGAAGTTCAATTGAACAAGGAATTGTTTTTGGATTCTTGTATAAAGGAAT
PfSD01       TAGAGATACACATGATGATATTAGAAGAATGTAAAGAGGAGGAAGTTCAATTGAACAAGGAATTGTTTTTGGATTCTTGTATAAAGGAAT
Pf7G8        TAGAGATACACATGATGATATTAGAAGAATGTAAAGAGGAGGAAGTTCAATTGAACAAGGAATTGTTTTTGGATTTTTGTATAAAGGAAT
FCR3 GENE    ------------------------------------------------------------------------------------------
MS822 GENE   ------------------------------------------------------------------------------------------
PfCD01_      TAGAGATACACATGATGATATTAGAAGAATGTAAAGAGGAGGAAGTTCAATTGAACAAGGAATTGTTTTTGGATTCTTGTATAAAGGAAT
PfKH01__     TAGAGATACACATGATGATATTAGAAGAATGTAAAGAGGAGGAAGTTCAATTGAACAAGGAATTGTTTTTGGATTCTTGTATAAAGGAAT
PfDd2__      TAGAGATACACATGATGATATTAGAAGAATGTAAAGAGGAGGAAGTTCAATTGAACAAGGAATTGTTTTTGGATTCTTGTATAAAGGAAT
PfSN01_      TAGAGATACCCATGATGATATTAGAAGAATGTAAAGAGGAGGAAGTTCAATTGAACAAGGAATTGTTTTTGGATTCTTGTATAAAGGAAT
PfGN01       TAGAGATACACATGATGATATTAGAAGAATGTAAAGAGGAGGAAGTTCAATTGAACAAGGAATTGTTTTTGGATTCTTGTATAAAGGAAT
PfKE01       TAGAGATACACATGATGATATTAGAAGAATGTAAAGAGGAGGAAGTTCAATTGAACAAGGAATTGTTTTTGGATTCTTGTATAAAGGAAT
PfKH02       TAGAGATACACATGATGATATTAGAAGAATGTAAAGAGGAGGAAGTTCAATTGAACAAGGAATTGTTTTTGGATTCTTGTATAAAGGAAT
PfGB4        TAGAGATACACATGATGATATTAGAAGAATGTAAAGAGGAGGAAGTTCAATTGAACAAGGAATTGTTTTTGGATTCTTGTATAAAGGAAT
PfGA01_      TAGAGATACACATGATGATATTAGAAGAATGTAAAGAGGAGGAAGTTCAATTGAACAAGGAATTGTTTTTGGATTCTTGTATAAAGGAAT
PfHB3_       TAGAGATACACATGATGATATTAGAAGAATGTAAAGAGGAGGAAGTTCAATTGAACAAGGAATTGTTTTTGGATTCTTGTATAAAGGAAT
PfIT_        TAGAGATACACATGATGATATTAGAAGAATGTAAAGAGGAGGAAGTTCAATTGAACAAGGAATTGTTTTTGGATTCTTGTATAAAGGAAT
```

```
                 5860      5870      5880      5890      5900      5910      5920      5930      5940
            ....|....|....|....|....|....|....|....|....|....|....|....|....|....|....|....|....|....|
PF3D7 CDS   TAATCAAGGAAAAAGAATCTAAAGGAAAATGTAAAATGTTGGAAATTGTATTAGATTTGAAAGACAAACATGTATTAATAAATAAAAATT
MS822 mRNA  ------------------------------------------------------------------------------------------
FCR3 mRNA   ------------------------------------------------------------------------------------------
AM1898      TAATCAAGGAAAAAGAATCTAAAGGAAAATGTAAAATGTTGGAAATTGTATTAGATTTGAAAGACAAACATGTATTAATAAATAAAAATT
AA235       TAATCAAGGAAAAAGAATCTAAAGGAAAATGTAAAATGTTGGAAATTGTATTAGATTTGAAAGACAAACATGTATTAATAAATAAAAATT
AA243       TAATCAAGGAAAAAGAATCTAAAGGAAAATGTAAAATGTTGGAAATTGTATTAGATTTGAAAGACAAACATGTATTAATAAATAAAAATT
AA258       TAATCAAGGAAAAAGAATCTAAAGGAAAATGTAAAATGTTGGAAATTGTATTAGATTTGAAAGACAAACATGTATTAATAAATAAAAATT
AA264       TAATCAAGGAAAAAGAATCTAAAGGAAAATGTAAAATGTTGGAAATTGTATTAGATTTGAAAGACAAACATGTATTAATAAATAAAAATT
AM1737      TAATCAAGGAAAAAGAATCTAAAGGAAAATGTAAAATGTTGGAAATTGTATTAGATTTGAAAGACAAACATGTATTAATAAATAAAAATT
SC74        TAATCAAGGAAAAAGAATCTAAAGGAAAATGTAAAATGTTGGAAATTGTATTAGATTTGAAAGACAAACATGTATTAATAAATAAAAATT
TAB166      TAATCAAGGAAAAAGAATCTAAAGGAAAATGTAAAATGTTGGAAATTGTATTAGATTTGAAAGACAAACATGTATTAATAAATAAAAATT
TAB138      TAATCAAGGAAAAAGAATCTAAAGGAAAATGTAAAATGTTGGAAATTGTATTAGATTTGAAAGACAAACATGTATTAATAAATAAAAATT
TAB130      TAATCAAGGAAAAAGAATCTAAAGGAAAATGTAAAATGTTGGAAATTGTATTAGATTTGAAAGACAAACATGTATTAATAAATAAAAATT
TAB131      TAATCAAGGAAAAAGAATCTAAAGGAAAATGTAAAATGTTGGAAATTGTATTAGATTTGAAAGACAAACATGTATTAATAAATAAAAATT
TAB151      TAATCAAGGAAAAAGAATCTAAAGGAAAATGTAAAATGTTGGAAATTGTATTAGATTTGAAAGACAAACATGTATTAATAAATAAAAATT
TAB152      TAATCAAGGAAAAAGAATCTAAAGGAAAATGTAAAATGTTGGAAATTGTATTAGATTTGAAAGACAAACATGTATTAATAAATAAAAATT
SC52        TAATCAAGGAAAAAGAATCTAAAGGAAAATGTAAAATGTTGGAAATTGTATTAGATTTGAAAGACAAACATGTATTAATAAATAAAAATT
SC55        TAATCAAGGAAAAAGAATCTAAAGGAAAATGTAAAATGTTGGAAATTGTATTAGATTTGAAAGACAAACATGTATTAATAAATAAAAATT
SC03        TAATCAAGGAAAAAGAATCTAAAGGAAAATGTAAAATGTTGGAAATTGTATTAGATTTGAAAGACAAACATGTATTAATAAATAAAAATT
SC53        TAATCAAGGAAAAAGAATCTAAAGGAAAATGTAAAATGTTGGAAATTGTATTAGATTTGAAAGACAAACATGTATTAATAAATAAAAATT
TAB141      TAATCAAGGAAAAAGAATCTAAAGGAAAATGTAAAATGTTGGAAATTGTATTAGATTTGAAAGACAAACATGTATTAATAAATAAAAATT
TAB123      TAATCAAGGAAAAAGAATCTAAAGGAAAATGTAAAATGTTGGAAATTGTATTAGATTTGAAAGACAAACATGTATTAATAAATAAAAATT
AM1803      TAATCAAGGAAAAAGAATCTAAAGGAAAATGTAAAATGTTGGAAATTGTATTAGATTTGAAAGACAAACATGTATTAATAAATAAAAATT
AM1814      TAATCAAGGAAAAAGAATCTAAAGGAAAATGTAAAATGTTGGAAATTGTATTAGATTTGAAAGACAAACATGTATTAATAAATAAAAATT
AM1802      TAATCAAGGAAAAAGAATCTAAAGGAAAATGTAAAATGTTGGAAATTGTATTAGATTTGAAAGACAAACATGTATTAATAAATAAAAATT
AM1811      TAATCAAGGAAAAAGAATCTAAAGGAAAATGTAAAATGTTGGAAATTGTATTAGATTTGAAAGACAAACATGTATTAATAAATAAAAATT
SC50        TAATCAAGGAAAAAGAATCTAAAGGAAAATGTAAAATGTTGGAAATTGTATTAGATTTGAAAGACAAACATGTATTAATAAATAAAAATT
SC56        TAATCAAGGAAAAAGAATCTAAAGGAAAATGTAAAATGTTGGAAATTGTATTAGATTTGAAAGACAAACATGTATTAATAAATAAAAATT
TAB153      TAATCAAGGAAAAAGAATCTAAAGGAAAATGTAAAATGTTGGAAATTGTATTAGATTTGAAAGACAAACATGTATTAATAAATAAAAATT
TAB154      TAATCAAGGAAAAAGAATCTAAAGGAAAATGTAAAATGTTGGAAATTGTATTAGATTTGAAAGACAAACATGTATTAATAAATAAAAATT
TAB156      TAATCAAGGAAAAAGAATCTAAAGGAAAATGTAAAATGTTGGAAATTGTATTAGATTTGAAAGACAAACATGTATTAATAAATAAAAATT
TAB136      TAATCAAGGAAAAAGAATCTAAAGGAAAATGTAAAATGTTGGAAATTGTATTAGATTTGAAAGACAAACATGTATTAATAAATAAAAATT
AM1880      TAATCAAGGAAAAAGAATCTAAAGGAAAATGTAAAATGTTGGAAATTGTATTAGATTTGAAAGACAAACATGTATTAATAAATAAAAATT
PF3D7_      TAATCAAGGAAAAAGAATCTAAAGGAAAATGTAAAATGTTGGAAATTGTATTAGATTTGAAAGACAAACATGTATTAATAAATAAAAATT
PfSD01      TAATCAAGGAAAAAGAATCTAAAGGAAAATGTAAAATGTTGGAAATTGTATTAGATTTGAAAGACAAACATGTATTAATAAATAAAAATT
Pf7G8       TAATCAAGGAAAAAGAATCTAAAGGAAAATGTAAAATGTTGGAAATTGTATTAGATTTGAAAGACAAACATGTATTAATAAATAAAAATT
FCR3 GENE   ------------------------------------------------------------------------------------------
MS822 GENE  ------------------------------------------------------------------------------------------
PfCD01_     TAATCAAGGAAAAAGAATCTAAAGGAAAATGTAAAATGTTGGAAATTGTATTAGATTTGAAAGACAAACATGTATTAATAAATAAAAATT
PfKH01__    TAATCAAGGAAAAAGAATCTAAAGGAAAATGTAAAATGTTGGAAATTGTATTAGATTTGAAAGACAAACATGTATTAATAAATAAAAATT
PfDd2__     TAATCAAGGAAAAAGAATCTAAAGGAAAATGTAAAATGTTGGAAATTGTATTAGATTTGAAAGACAAACATGTATTAATAAATAAAAATT
PfSN01_     TAATCAAGGAAAAAGAATCTAAAGGAAAATGTAAAATGTTGGAAATTGTATTAGATTTGAAAGACAAACATGTATTAATAAATAAAAATT
PfGN01      TAATCAAGGAAAAAGAATCTAAAGGAAAATGTAAAATGTTGGAAATTGTATTAGATTTGAAAGACAAACATGTATTAATAAATAAAAATT
PfKE01      TAATCAAGGAAAAAGAATCTAAAGGAAAATGTAAAATGTTGGAAATTGTATTAGATTTGAAAGACAAACATGTATTAATAAATAAAAATT
PfKH02      TAATCAAGGAAAAAGAATCTAAAGGAAAATGTAAAATGTTGGAAATTGTATTAGATTTGAAAGACAAACATGTATTAATAAATAAAAATT
PfGB4       TAATCAAGGAAAAAGAATCTAAAGGAAAATGTAAAATGTTGGAAATTGTATTAGATTTGAAAGACAAACATGTATTAATAAATAAAAATT
PfGA01_     TAATCAAGGAAAAAGAATCTAAAGGAAAATGTAAAATGTTGGAAATTGTATTAGATTTGAAAGACAAACATGTATTAATAAATAAAAATT
PfHB3_      TAATCAAGGAAAAAGAATCTAAAGGAAAATGTAAAATGTTGGAAATTGTATTAGATTTGAAAGACAAACATGTATTAATAAATAAAAATT
PfIT_       TAATCAAGGAAAAAGAATCTAAAGGAAAATGTAAAATGTTGGAAATTGTATTAGATTTGAAAGACAAACATGTATTAATAAATAAAAATT
```

```
                      5950      5960      5970      5980      5990      6000      6010      6020      6030
              ....|....|....|....|....|....|....|....|....|....|....|....|....|....|....|....|....|....|
PF3D7 CDS     TGGAAATGAATAAAAGGATAGGAAAAAATAGTAATAATTTGTTTGAAAATATTAAAACCCAAGTGAAAAATAATGAAAATAATTACATAT
MS822 mRNA    ------------------------------------------------------------------------------------------
FCR3 mRNA     ------------------------------------------------------------------------------------------
AM1898        TGGAAATGAATAAAAGGATAGGAAAAAATAGTAATAATTTGTTTGAAAATATTAAAACCCAAGTGAAAAATAATGAAAATAATTACATAT
AA235         TGGAAATGAATAAAAGGATAGGAAAAAATAGTAATAATTTGTTTGAAAATATTAAAACCCAAGTGAAAAATAATGAAAATAATTACATAT
AA243         TGGAAATGAATAAAAGGATAGGAAAAAATAGTAATAATTTGTTTGAAAATATTAAAACCCAAGTGAAAAATAATGAAAATAATTACATAT
AA258         TGGAAATGAATAAAAGGATAGGAAAAAATAGTAATAATTTGTTTGAAAATATTAAAACCCAAGTGAAAAATAATGAAAATAATTACATAT
AA264         TGGAAATGAATAAAAGGATAGGAAAAAATAGTAATAATTTGTTTGAAAATATTAAAACCCAAGTGAAAAATAATGAAAATAATTACATAT
AM1737        TGGAAATGAATAAAAGGATAGGAAAAAATAGTAATAATTTGTTTGAAAATGTTAAAACCCAAGTGAAAAATAATGAAAATAATTACATAT
SC74          TGGAAATGAATAAAAGGATAGGAAAAAATAGTAATAATTTGTTTGAAAATATTAAAACCCAAGTGAAAAATAATGAAAATAATTACATAT
TAB166        TGGAAATGAATAAAAGGATAGGAAAAAATAGTAATAATTTGTTTGAAAATATTAAAACCCAAGTGAAAAATAATGAAAATAATTACATAT
TAB138        TGGAAATGAATAAAAGGATAGGAAAAAATAGTAATAATTTGTTTGAAAATATTAAAACCCAAGTGAAAAATAATGAAAATAATTACATAT
TAB130        TGGAAATGAATAAAAGGATAGGAAAAAATAGTAATAATTTGTTTGAAAATATTAAAACCCAAGTGAAAAATAATGAAAATAATTACATAT
TAB131        TGGAAATGAATAAAAGGATAGGAAAAAATAGTAATAATTTGTTTGAAAATATTAAAACCCAAGTGAAAAATAATGAAAATAATTACATAT
TAB151        TGGAAATGAATAAAAGGATAGGAAAAAATAGTAATAATTTGTTTGAAAATATTAAAACCCAAGTGAAAAATAATGAAAATAATTACATAT
TAB152        TGGAAATGAATAAAAGGATAGGAAAAAATAGTAATAATTTGTTTGAAAATATTAAAACCCAAGTGAAAAATAATGAAAATAATTACATAT
SC52          TGGAAATGAATAAAAGGATAGGAAAAAATAGTAATAATTTGTTTGAAAATATTAAAACCCAAGTGAAAAATAATGAAAATAATTACATAT
SC55          TGGAAATGAATAAAAGGATAGGAAAAAATAGTAATAATTTGTTTGAAAATATTAAAACCCAAGTGAAAAATAATGAAAATAATTACATAT
SC03          TGGAAATGAATAAAAGGATAGGAAAAAATAGTAATAATTTGTTTGAAAATATTAAAACCCAAGTGAAAAATAATGAAAATAATTACATAT
SC53          TGGAAATGAATAAAAGGATAGGAAAAAATAGTAATAATTTGTTTGAAAATATTAAAACCCAAGTGAAAAATAATGAAAATAATTACATAT
TAB141        TGGAAATGAATAAAAGGATAGGAAAAAATAGTAATAATTTGTTTGAAAATATTAAAACCCAAGTGAAAAATAATGAAAATAATTACATAT
TAB123        TGGAAATGAATAAAAGGATAGGAAAAAATAGTAATAATTTGTTTGAAAATATTAAAACCCAAGTGAAAAATAATGAAAATAATTACATAT
AM1803        TGGAAATGAATAAAAGGATAGGAAAAAATAGTAATAATTTGTTTGAAAATATTAAAACCCAAGTGAAAAATAATGAAAATAATTACATAT
AM1814        TGGAAATGAATAAAAGGATAGGAAAAAATAGTAATAATTTGTTTGAAAATATTAAAACCCAAGTGAAAAATAATGAAAATAATTACATAT
AM1802        TGGAAATGAATAAAAGGATAGGAAAAAATAGTAATAATTTGTTTGAAAATATTAAAACCCAAGTGAAAAATAATGAAAATAATTACATAT
AM1811        TGGAAATGAATAAAAGGATAGGAAAAAATAGTAATAATTTGTTTGAAAATATTAAAACCCAAGTGAAAAATAATGAAAATAATTACATAT
SC50          TGGAAATGAATAAAAGGATAGGAAAAAATAGTAATAATTTGTTTGAAAATATTAAAACCCAAGTGAAAAATAATGAAAATAATTACATAT
SC56          TGGAAATGAATAAAAGGATAGGAAAAAATAGTAATAATTTGTTTGAAAATATTAAAACCCAAGTGAAAAATAATGAAAATAATTACATAT
TAB153        TGGAAATGAATAAAAGGATAGGAAAAAATAGTAATAATTTGTTTGAAAATGTTAAAACCCAAGTGAAAAATAATGAAAATAATTACATAT
TAB154        TGGAAATGAATAAAAGGATAGGAAAAAATAGTAATAATTTGTTTGAAAATATTAAAACCCAAGTGAAAAATAATGAAAATAATTACATAT
TAB156        TGGAAATGAATAAAAGGATAGGAAAAAATAGTAATAATTTGTTTGAAAATATTAAAACCCAAGTGAAAAATAATGAAAATAATTACATAT
TAB136        TGGAAATGAATAAAAGGATAGGAAAAAATAGTAATAATTTGTTTGAAAATATTAAAACCCAAGTGAAAAATAATGAAAATAATTACATAT
AM1880        TGGAAATGAATAAAAGGATAGGAAAAAATAGTAATAATTTGTTTGAAAATATTAAAACCCAAGTGAAAAATAATGAAAATAATTACATAT
PF3D7_        TGGAAATGAATAAAAGGATAGGAAAAAATAGTAATAATTTGTTTGAAAATATTAAAACCCAAGTGAAAAATAATGAAAATAATTACATAT
PfSD01        TGGAAATGAATAAAAGGATAGGAAAAAATAGTAATAATTTGTTTGAAAATATTAAAACCCAAGTGAAAAATAATGAAAATAATTACATAT
Pf7G8         TGGAAATGAATAAAAGGATAGGAAAAAATAGTAATAATTTGTTTGAAAATATTAAAACCCAAGTGAAAAATAATGAAAATAATTACATAT
FCR3 GENE     ------------------------------------------------------------------------------------------
MS822 GENE    ------------------------------------------------------------------------------------------
PfCD01_       TGGAAATGAATAAAAGGATAGGAAAAAATAGTAATAATTTGTTTGAAAATATTAAAACCCAAGTGAAAAATAATGAAAATAATTACATAT
PfKH01__      TGGAAATGAATAAAAGGATAGGAAAAAATAGTAATAATTTGTTTGAAAATATTAAAACCCAAGTGAAAAATAATGAAAATAATTACATAT
PfDd2__       TGGAAATGAATAAAAGGATAGGAAAAAATAGTAATAATTTGTTTGAAAATATTAAAACCCAAGTGAAAAATAATGAAAATAATTACATAT
PfSN01_       TGGAAATGAATAAAAGGATAGGAAAAAATAGTAATAATTTGTTTGAAAATATTAAAACCCAAGTGAAAAATAATGAAAATAATTACATAT
PfGN01        TGGAAATGAATAAAAGGATAGGAAAAAATAGTAATAATTTGTTTGAAAATATTAAAACCCAAGTGAAAAATAATGAAAATAATTACATAT
PfKE01        TGGAAATGAATAAAAGGATAGGAAAAAATAGTAATAATTTGTTTGAAAATATTAAAACCCAAGTGAAAAATAATGAAAATAATTACATAT
PfKH02        TGGAAATGAATAAAAGGATAGGAAAAAATAGTAATAATTTGTTTGAAAATATTAAAACCCAAGTGAAAAATAATGAAAATAATTACATAT
PfGB4         TGGAAATGAATAAAAGGATAGGAAAAAATAGTAATAATTTGTTTGAAAATATTAAAACCCAAGTGAAAAATAATGAAAATAATTACATAT
PfGA01_       TGGAAATGAATAAAAGGATAGGAAAAAATAGTAATAATTTGTTTGAAAATATTAAAACCCAAGTGAAAAATAATGAAAATAATTACATAT
PfHB3_        TGGAAATGAATAAAAGGATAGGAAAAAATAGTAATAATTTGTTTGAAAATATTAAAACCCAAGTGAAAAATAATGAAAATAATTACATAT
PfIT_         TGGAAATGAATAAAAGGATAGGAAAAAATAGTAATAATTTGTTTGAAAATATTAAAACCCAAGTGAAAAATAATGAAAATAATTACATAT
```

```
                       6040      6050      6060      6070      6080      6090      6100      6110      6120
              ....|....|....|....|....|....|....|....|....|....|....|....|....|....|....|....|....|....|
PF3D7 CDS     ATGAAATGATGTTAAACAATGACATGAATAAGTCAGGCAAAATGAGTGAAAATGATTGTATATTTGATGAAATGTTAATAAATAATATGA
MS822 mRNA    ------------------------------------------------------------------------------------------
FCR3 mRNA     ------------------------------------------------------------------------------------------
AM1898        ATGAAATGATGTTAAACAATGACATGAATAAGTCAGGCAAAATGAGTGAAAATGATTGTATATGTGATGAAATGTTAATAAATAATATGA
AA235         ATGAAATGATGTTAAACAATGACATGAATAAGTCAGGCAAAATGAGTGAAAATGATTGTATATTTGATGAAATGTTAATAAATAATATGA
AA243         ATGAAATGATGTTAAACAATGACATGAATAAGTCAGGCAAAATGAGTGAAAATGATTGTATATTTGATGAAATGTTAATAAATAATATGA
AA258         ATGAAATGATGTTAAACAATGACATGAATAAGTCAGGCAAAATGAGTGAAAATGATTGTATATTTGATGAAATGTTAATAAATAATATGA
AA264         ATGAAATGATGTTAAACAATGACATGAATAAGTCAGGCAAAATGAGTGAAAATGATTGTATATTTGATGAAATGTTAATAAATAATATGA
AM1737        ATGAAATGATGTTAAACAATGACATGAATAAGTCAGGCAAAATGAGTGAAAATGATTGTATATTTGATGAAATGTTAATAAATAATATGA
SC74          ATGAAATGATGTTAAACAATGACATGAATAAGTCAGGCAAAATGAGTGAAAATGATTGTATATTTGATGAAATGTTAATAAATAATATGA
TAB166        ATGAAATGATGTTAAACAATGACATGAATAAGTCAGGCAAAATGAGTGAAAATGATTGTATATTTGATGAAATGTTAATAAATAATATGA
TAB138        ATGAAATGATGTTAAACAATGACATGAATAAGTCAGGCAAAATGAGTGAAAATGATTGTATATTTGATGAAATGTTAATAAATAATATGA
TAB130        ATGAAATGATGTTAAACAATGACATGAATAAGTCAGGCAAAATGAGTGAAAATGATTGTATATTTGATGAAATGTTAATAAATAATATGA
TAB131        ATGAAATGATGTTAAACAATGACATGAATAAGTCAGGCAAAATGATCGAAAATGATTGTATATGTGATGAAATGTTAATAAATAATATGA
TAB151        ATGAAATGATGTTAAACAATGACATGAATAAGTCAGGCAAAATGATCGAAAATGATTGTATATGTGATGAAATGTTAATAAATAATATGA
TAB152        ATGAAATGATGTTAAACAATGACATGAATAAGTCAGGCAAAATGAGTGAAAATGATTGTATATTTGATGAAATGTTAATAAATAATATGA
SC52          ATGAAATGATGTTAAACAATGACATGAATAAGTCAGGCAAAATGAGTGAAAATGATTGTATATTTGATGAAATGTTAATAAATAATATGA
SC55          ATGAAATGATGTTAAACAATGACATGAATAAGTCAGGCAAAATGATCGAAAATGATTGTATATGTGATGAAATGTTAATAAATAATATGA
SC03          ATGAAATGATGTTAAACAATGACATGAATAAGTCAGGCAAAATGAGTGAAAATGATTGTATATTTGATGAAATGTTAATAAATAATATGA
SC53          ATGAAATGATGTTAAACAATGACATGAATAAGTCAGGCAAAATGAGTGAAAATGATTGTATATTTGATGAAATGTTAATAAATAATATGA
TAB141        ATGAAATGATGTTAAACAATGACATGAATAAGTCAGGCAAAATGATCGAAAATGATTGTATATGTGATGAAATGTTAATAAATAATATGA
TAB123        ATGAAATGATGTTAAACAATGACATGAATAAGTCAGGCAAAATGAGTGAAAATGATTGTATATTTGATGAAATGTTAATAAATAATATGA
AM1803        ATGAAATGATGTTAAACAATGACATGAATAAGTCAGGCAAAATGAGTGAAAATGATTGTATATTTGATGAAATGTTAATAAATAATATGA
AM1814        ATGAAATGATGTTAAACAATGACATGAATAAGTCAGGCAAAATGATCGAAAATGATTGTATATGTGATGAAATGTTAATAAATAATATGA
AM1802        ATGAAATGATGTTAAACAATGACATGAATAAGTCAGGCAAAATGATCGAAAATGATTGTATATGTGATGAAATGTTAATAAATAATATGA
AM1811        ATGAAATGATGTTAAACAATGACATGAATAAGTCAGGCAAAATGAGTGAAAATGATTGTATATTTGATGAAATGTTAATAAATAATATGA
SC50          ATGAAATGATGTTAAACAATGACATGAATAAGTCAGGCAAAATGAGTGAAAATGATTGTATATTTGATGAAATGTTAATAAATAATATGA
SC56          ATGAAATGATGTTAAACAATGACATGAATAAGTCAGGCAAAATGAGTGAAAATGATTGTATATTTGATGAAATGTTAATAAATAATATGA
TAB153        ATGAAATGATGTTAAACAATGACATGAATAAGTCAGGCAAAATGAGTGAAAATGATTGTATATTTGATGAAATGTTAATAAATAATATGA
TAB154        ATGAAATGATGTTAAACAATGACATGAATAAGTCAGGCAAAATGAGTGAAAATGATTGTATATTTGATGAAATGTTAATAAATAATATGA
TAB156        ATGAAATGATGTTAAACAATGACATGAATAAGTCAGGCAAAATGATCGAAAATGATTGTATATGTGATGAAATGTTAATAAATAATATGA
TAB136        ATGAAATGATGTTAAACAATGACATGAATAAGTCAGGCAAAATGAGTGAAAATGATTGTATATTTGATGAAATGTTAATAAATAATATGA
AM1880        ATGAAATGATGTTAAACAATGACATGAATAAGTCAGGCAAAATGATCGAAAATGATTGTATATGTGATGAAATGTTAATAAATAATATGA
PF3D7_        ATGAAATGATGTTAAACAATGACATGAATAAGTCAGGCAAAATGAGTGAAAATGATTGTATATTTGATGAAATGTTAATAAATAATATGA
PfSD01        ATGAAATGATGTTAAACAATGACATGAATAAGTCAGGCAAAATGATCGAAAATGATTGTATATGTGATGAAATGTTAATAAATAATATGA
Pf7G8         ATGAAATGATGTTAAACAATGACATGAATAAGTCAGGCAAAATGAGTGAAAATGATTGTATATTTGATGAAATGTTAATAAATAATATGA
FCR3 GENE     ------------------------------------------------------------------------------------------
MS822 GENE    ------------------------------------------------------------------------------------------
PfCD01_       ATGAAATGATGTTAAACAATGACATGAATAAGTCAGGCAAAATGAGTGAAAATGATTGTATATTTGATGAAATGTTAATAAATAATATGA
PfKH01__      ATGAAATGATGTTAAACAATGACATGAATAAGTCAGGCAAAATGAGTGAAAATGATTGTATATTTGATGAAATGTTAATAAATAATATGA
PfDd2__       ATGAAATGATGTTAAACAATGACATGAATAAGTCAGGCAAAATGATCGAAAATGATTGTATATGTGATGAAATGTTAATAAATAATATGA
PfSN01_       ATGAAATGATGTTAAACAATGACATGAATAAGTCAGGCAAAATGAGTGAAAATGATTGTATATTTGATGAAATGTTAATAAATAATATGA
PfGN01        ATGAAATGATGTTAAACAATGACATGAATAAGTCAGGCAAAATGAGTGAAAATGATTGTATATTTGATGAAATGTTAATAAATAATATGA
PfKE01        ATGAAATGATGTTAAACAATGACATGAATAAGTCAGGCAAAATGAGTGAAAATGATTGTATATTTGATGAAATGTTAATAAATAATATGA
PfKH02        ATGAAATGATGTTAAACAATGACATGAATAAGTCAGGCAAAATGAGTGAAAATGATTGTATATTTGATGAAATGTTAATAAATAATATGA
PfGB4         ATGAAATGATGTTAAACAATGACATGAATAAGTCAGGCAAAATGAGTGAAAATGATTGTATATTTGATGAAATGTTAATAAATAATATGA
PfGA01_       ATGAAATGATGTTAAACAATGACATGAATAAG----------------------------------------------------------
PfHB3_        ATGAAATGATGTTAAACAATGACATGAATAAGTCAGGCAAAATGATCGAAAATGATTGTATATGTGATGAAATGTTAATAAATAATATGA
PfIT_         ATGAAATGATGTTAAACAATGACATGAATAAGTCAGGCAAAATGAGTGAAAATGATTGTATATTTGATGAAATGTTAATAAATAATATGA
```

```
                  6130      6140      6150      6160      6170      6180      6190      6200      6210
              ....|....|....|....|....|....|....|....|....|....|....|....|....|....|....|....|....|....|
PF3D7 CDS     ACAAGTCAGATAGAATTGGACAAAAAAAGGATAATACAATTGAAGATAATTTAAAAAGCAATATGAACAAGTTAGGTGATGTAAATGAAA
MS822 mRNA    ------------------------------------------------------------------------------------------
FCR3 mRNA     ------------------------------------------------------------------------------------------
AM1898        ACAAGTCAGATAGAATTGGACAAAAAAATGATAATACAATTGAAGATAATTTAAAAAGCAATATGAACAAGTTAGGTGATGTAAATGAAA
AA235         ACAAGTCAGATAGAATTGGACAAAAAAAGGATAATACAATTGAAGATAATTTAAAAAGCAATATGAACAAGTTAGGTGATGTAAATGAAA
AA243         ACAAGTCAGATAGAATTGGACAAAAAAAGGATAATACAATTGAAGATAATTTAAAAAGCAATATGAACAAGTTAGGTGATGTAAATGAAA
AA258         ACAAGTCAGATAGAATTGGACAAAAAAAGGATAATACAATTGAAGATAATTTAAAAAGCAATATGAACAAGTTAGGTGATGTAAATGAAA
AA264         ACAAGTCAGATAGAATTGGACAAAAAAAGGATAATACAATTGAAGATAATTTAAAAAGCAATATGAACAAGTTAGGTGATGTAAATGAAA
AM1737        ACAAGTCAGATAGAATTGGACAAAAAAAGGATAATACAATTGAAGATAATTTAAAAAGCAATATGAACAAGTTAGGTGATGTAAATGAAA
SC74          ACAAGTCAGATAGAATTGGACAAAAAAAGGATAATACAATTGAAGATAATTTAAAAAGCAATATGAACAAGTTAGGTGATGTAAATGAAA
TAB166        ACAAGTCAGATAGAATTGGACAAAAAAAGGATAATACAATTGAAGATAATTTAAAAAGCAATATGAACAAGTTAGGTGATGTAAATGAAA
TAB138        ACAAGTCAGATAGAATTGGACAAAAAAAGGATAATACAATTGAAGATAATTTAAAAAGCAATATGAACAAGTTAGGTGATGTAAATGAAA
TAB130        ACAAGTCAGATAGAATTGGACAAAAAAAGGATAATACAATTGAAGATAATTTAAAAAGCAATATGAACAAGTTAGGTGATGTAAATGAAA
TAB131        ACAAGTCAGATAGAATTGGACAAAAAAATGATAATACAATTGAAGATAATTTAAAAAGCAATATGAACAAGTTAGGTGATGTAAATGAAA
TAB151        ACAAGTCAGATAGAATTGGACAAAAAAATGATAATACAATTGAAGATAATTTAAAAAGCAATATGAACAAGTTAGGTGATGTAAATGAAA
TAB152        ACAAGTCAGATAGAATTGGACAAAAAAAGGATAATACAATTGAAGATAATTTAAAAAGCAATATGAACAAGTTAGGTGATGTAAATGAAA
SC52          ACAAGTCAGATAGAATTGGACAAAAAAATGATAATACAATTGAAGATAATTTAAAAAGCAATATGAACAAGTTAGGTGATGTAAATGAAA
SC55          ACAAGTCAGATAGAATTGGACAAAAAAAGGATAATACAATTGAAGATAATTTAAAAAGCAATATGAACAAGTTAGGTGATGTAAATGAAA
SC03          ACAAGTCAGATAGAATTGGACAAAAAAAGGATAATACAATTGAAGATAATTTAAAAAGCAATATGAACAAGTTAGGTGATGTAAATGAAA
SC53          ACAAGTCAGATAGAATTGGACAAAAAAAGGATAATACAATTGAAGATAATTTAAAAAGCAATATGAACAAGTTAGGTGATGTAAATGAAA
TAB141        ACAAGTCAGATAGAATTGGACAAAAAAATGATAATACAATTGAAGATAATTTAAAAAGCAATATGAACAAGTTAGGTGATGTAAATGAAA
TAB123        ACAAGTCAGATAGAATTGGACAAAAAAAGGATAATACAATTGAAGATAATTTAAAAAGCAATATGAACAAGTTAGGTGATGTAAATGAAA
AM1803        ACAAGTCAGATAGAATTGGACAAAAAAAGGATAATACAATTGAAGATAATTTAAAAAGCAATATGAACAAGTTAGGTGATGTAAATGAAA
AM1814        ACAAGTCAGATAGAATTGGACAAAAAAATGATAATACAATTGAAGATAATTTAAAAAGCAATATGAACAAGTTAGGTGATGTAAATGAAA
AM1802        ACAAGTCAGATAGAATTGGACAAAAAAATGATAATACAATTGAAGATAATTTAAAAAGCAATATGAACAAGTTAGGTGATGTAAATGAAA
AM1811        ACAAGTCAGATAGAATTGGACAAAAAAAGGATAATACAATTGAAGATAATTTAAAAAGCAATATGAACAAGTTAGGTGATGTAAATGAAA
SC50          ACAAGTCAGATAGAATTGGACAAAAAAAGGATAATACAATTGAAGATAATTTAAAAAGCAATATGAACAAGTTAGGTGATGTAAATGAAA
SC56          ACAAGTCAGATAGAATTGGACAAAAAAAGGATAATACAATTGAAGATAATTTAAAAAGCAATATGAACAAGTTAGGTGATGTAAATGAAA
TAB153        ACAAGTCAGATAGAATTGGACAAAAAAAGGATAATACAATTGAAGATAATTTAAAAAGCAATATGAACAAGTTAGGTGATGTAAATGAAA
TAB154        ACAAGTCAGATAGAATTGGACAAAAAAAGGATAATACAATTGAAGATAATTTAAAAAGCAATATGAACAAGTTAGGTGATGTAAATGAAA
TAB156        ACAAGTCAGATAGAATTGGACAAAAAAATGATAATACAATTGAAGATAATTTAAAAAGCAATATGAACAAGTTAGGTGATGTAAATGAAA
TAB136        ACAAGTCAGATAGAATTGGACAAAAAAAGGATAATACAATTGAAGATAATTTAAAAAGCAATATGAACAAGTTAGGTGATGTAAATGAAA
AM1880        ACAAGTCAGATAGAATTGGACAAAAAAATGATAATACAATTGAAGATAATTTAAAAAGCAATATGAACAAGTTAGGTGATGTAAATGAAA
PF3D7_        ACAAGTCAGATAGAATTGGACAAAAAAAGGATAATACAATTGAAGATAATTTAAAAAGCAATATGAACAAGTTAGGTGATGTAAATGAAA
PfSD01        ACAAGTCAGATAGAATTGGACAAAAAAATGATAATACAATTGAAGATAATTTAAAAAGCAATATGAACAAGTTAGGTGATGTAAATGAAA
Pf7G8         ACAAGTCAGATAGAATTGGACAAAAAAAGGATAATACAATTGAAGATAATTTAAAAAGCAATATGAACAAGTTAGGTGATGTAAATGAAA
FCR3 GENE     ------------------------------------------------------------------------------------------
MS822 GENE    ------------------------------------------------------------------------------------------
PfCD01_       ACAAGTCAGATAGAATTGGACAAAAAAAGGATAATACAATTGAAGATAATTTAAAAAGCAATATGAACAAGTTAGGTGATGTAAATGAAA
PfKH01__      ACAAGTCAGATAGAATTGGACAAAAAAAGGATAATACAATTGAAGATAATTTAAAAAGCAATATGAACAAGTTAGGTGATGTAAATGAAA
PfDd2__       ACAAGTCAGATAGAATTGGACAAAAAAAGGATAATACAATTGAAGATAATTTAAAAAGCAATATGAACAAGTTAGGTGATGTAAATGAAA
PfSN01_       ACAAGTCAGATAGAATTGGACAAAAAAAGGATAATACAATTGAAGATAATTTAAAAAGCAATATGAACAAGTTAGGTGATGTAAATGAAA
PfGN01        ACAAGTCAGATAGAATTGGACAAAAAAAGGATAATACAATTGAAGATAATTTAAAAAGCAATATGAACAAGTTAGGTGATGTAAATGAAA
PfKE01        ACAAGTCAGATAGAATTGGACAAAAAAAGGATAATACAATTGAAGATAATTTAAAAAGCAATATGAACAAGTTAGGTGATGTAAATGAAA
PfKH02        ACAAGTCAGATAGAATTGGACAAAAAAAGGATAATACAATTGAAGATAATTTAAAAAGCAATATGAACAAGTTAGGTGATGTAAATGAAA
PfGB4         ACAAGTCAGATAGAATTGGACAAAAAAAGGATAATACAATTGAAGATAATTTAAAAAGCAATATGAACAAGTTAGGTGATGTAAATGAAA
PfGA01_       ------------------------------------------------------------------------------------------
PfHB3_        ACAAGTCAGATAGAATTGGACAAAAAAATGATAATACAATTGAAGATAATTTAAAAAGCAATATGAACAAGTTAGGTGATGTAAATGAAA
PfIT_         ACAAGTCAGATAGAATTGGACAAAAAAAGGATAATACAATTGAAGATAATTTAAAAAGCAATATGAACAAGTTAGGTGATGTAAATGAAA
```

```
                     6220      6230      6240      6250      6260      6270      6280      6290      6300
             ....|....|....|....|....|....|....|....|....|....|....|....|....|....|....|....|....|....|
PF3D7 CDS    AATGTATTCTAGAAATGGAGAAGAATATATCCATGAGGCATTGGGAAAATATTAAAAAAAATTGTACAGATGGAAAAGAAAAAGAGAAAA
MS822 mRNA   ------------------------------------------------------------------------------------------
FCR3 mRNA    ------------------------------------------------------------------------------------------
AM1898       AATGTATTCTAGAAATGGAGAAGAATATATCCATGAGGCATTGGGAAAATATTAAAAAAAATTGTACAGATGGAAAAGAAAAAGAGAAAA
AA235        AATGTATTCTAGAAATGGAGAAGAATATATCCATGAGGCATTGGGAAAATATTAAAAAAAATTGTACAGATGGAAAAGAAAAAGAGAAAA
AA243        AATGTATTCTAGAAATGGAGAAGAATATATCCATGAGGCATTGGGAAAATATTAAAAAAAATTGTACAGATGGAAAAGAAAAAGAGAAAA
AA258        AATGTATTCTAGAAATGGAGAAGAATATATCCATGAGGCATTGGGAAAATATTAAAAAAAATTGTACAGATGGAAAAGAAAAAGAGAAAA
AA264        AATGTATTCTAGAAATGGAGAAGAATATATCCATGAGGCATTGGGAAAATATTAAAAAAAATTGTACAGATGGAAAAGAAAAAGAGAAAA
AM1737       AATGTATTCTAGAAATGGAGAAGAATATATCCATGAGGCATTGGGAAAATATTAAAAAAAATTGTACAGATGGAAAAGAAAAAGAGAAAA
SC74         AATGTATTCTAGAAATGGAGAAGAATATATCCATGAGGCATTGGGAAAATATTAAAAAAAATTGTACAGATGGAAAAGAAAAAGAGAAAA
TAB166       AATGTATTCTAGAAATGGAGAAGAATATATCCATGAGGCATTGGGAAAATATTAAAAAAAATTGTACAGATGGAAAAGAAAAAGAGAAAA
TAB138       AATGTATTCTAGAAATGGAGAAGAATATATCCATGAGGCATTGGGAAAATATTAAAAAAAATTGTACAGATGGAAAAGAAAAAGAGAAAA
TAB130       AATGTATTCTAGAAATGGAGAAGAATATATCCATGAGGCATTGGGAAAATATTAAAAAAAATTGTACAGATGGAAAAGAAAAAGAGAAAA
TAB131       AATGTATTCTAGAAATGGAGAAGAATATATCCATGAGGCATTGGGAAAATATTAAAAAAAATTGTACAGATGGAAAAGAAAAAGAGAAAA
TAB151       AATGTATTCTAGAAATGGAGAAGAATATATCCATGAGGCATTGGGAAAATATTAAAAAAAATTGTACAGATGGAAAAGAAAAAGAGAAAA
TAB152       AATGTATTCTAGAAATGGAGAAGAATATATCCATGAGGCATTGGGAAAATATTAAAAAAAATTGTACAGATGGAAAAGAAAAAGAGAAAA
SC52         AATGTATTCTAGAAATGGAGAAGAATATATCCATGAGGCATTGGGAAAATATTAAAAAAAATTGTACAGATGGAAAAGAAAAAGAGAAAA
SC55         AATGTATTCTAGAAATGGAGAAGAATATATCCATGAGGCATTGGGAAAATATTAAAAAAAATTGTACAGATGGAAAAGAAAAAGAGAAAA
SC03         AATGTATTCTAGAAATGGAGAAGAATATATCCATGAGGCATTGGGAAAATATTAAAAAAAATTGTACAGATGGAAAAGAAAAAGAGAAAA
SC53         AATGTATTCTAGAAATGGAGAAGAATATATCCATGAGGCATTGGGAAAATATTAAAAAAAATTGTACAGATGGAAAAGAAAAAGAGAAAA
TAB141       AATGTATTCTAGAAATGGAGAAGAATATATCCATGAGGCATTGGGAAAATATTAAAAAAAATTGTACAGATGGAAAAGAAAAAGAGAAAA
TAB123       AATGTATTCTAGAAATGGAGAAGAATATATCCATGAGGCATTGGGAAAATATTAAAAAAAATTGTACAGATGGAAAAGAAAAAGAGAAAA
AM1803       AATGTATTCTAGAAATGGAGAAGAATATATCCATGAGGCATTGGGAAAATATTAAAAAAAATTGTACAGATGGAAAAGAAAAAGAGAAAA
AM1814       AATGTATTCTAGAAATGGAGAAGAATATATCCATGAGGCATTGGGAAAATATTAAAAAAAATTGTACAGATGGAAAAGAAAAAGAGAAAA
AM1802       AATGTATTCTAGAAATGGAGAAGAATATATCCATGAGGCATTGGGAAAATATTAAAAAAAATTGTACAGATGGAAAAGAAAAAGAGAAAA
AM1811       AATGTATTCTAGAAATGGAGAAGAATATATCCATGAGGCATTGGGAAAATATTAAAAAAAATTGTACAGATGGAAAAGAAAAAGAGAAAA
SC50         AATGTATTCTAGAAATGGAGAAGAATATATCCATGAGGCATTGGGAAAATATTAAAAAAAATTGTACAGATGGAAAAGAAAAAGAGAAAA
SC56         AATGTATTCTAGAAATGGAGAAGAATATATCCATGAGGCATTGGGAAAATATTAAAAAAAATTGTACAGATGGAAAAGAAAAAGAGAAAA
TAB153       AATGTATTCTAGAAATGGAGAAGAATATATCCATGAGGCATTGGGAAAATATTAAAAAAAATTGTACAGATGGAAAAGAAAAAGAGAAAA
TAB154       AATGTATTCTAGAAATGGAGAAGAATATATCCATGAGGCATTGGGAAAATATTAAAAAAAATTGTACAGATGGAAAAGAAAAAGAGAAAA
TAB156       AATGTATTCTAGAAATGGAGAAGAATATATCCATGAGGCATTGGGAAAATATTAAAAAAAATTGTACAGATGGAAAAGAAAAAGAGAAAA
TAB136       AATGTATTCTAGAAATGGAGAAGAATATATCCATGAGGCATTGGGAAAATATTAAAAAAAATTGTACAGATGGAAAAGAAAAAGAGAAAA
AM1880       AATGTATTCTAGAAATGGAGAAGAATATATCCATGAGGCATTGGGAAAATATTAAAAAAAATTGTACAGATGGAAAAGAAAAAGAGAAAA
PF3D7_       AATGTATTCTAGAAATGGAGAAGAATATATCCATGAGGCATTGGGAAAATATTAAAAAAAATTGTACAGATGGAAAAGAAAAAGAGAAAA
PfSD01       AATGTATTCTAGAAATGGAGAAGAATATATCCATGAGGCATTGGGAAAATATTAAAAAAAATTGTACAGATGGAAAAGAAAAAGAGAAAA
Pf7G8        AATGTATTCTAGAAATGGAGAAGAATATATCCATGAGGCATTGGGAAAATATTAAAAAAAATTGTACAGATGGAAAAGAAAAAGAGAAAA
FCR3 GENE    ------------------------------------------------------------------------------------------
MS822 GENE   ------------------------------------------------------------------------------------------
PfCD01_      AATGTATTCTAGAAATGGAGAAGAATATATCCATGAGGCATTGGGAAAATATTAAAAAAAATTGTACAGATGGAAAAGAAAAAGAGAAAA
PfKH01__     AATGTATTCTAGAAATGGAGAAGAATATATCCATGAGGCATTGGGAAAATATTAAAAAAAATTGTACAGATGGAAAAGAAAAAGAGAAAA
PfDd2__      AATGTATTCTAGAAATGGAGAAGAATATATCCATGAGGCATTGGGAAAATATTAAAAAAAATTGTACAGATGGAAAAGAAAAAGAGAAAA
PfSN01_      AATGTATTCTAGAAATGGAGAAGAATATATCCATGAGGCATTGGGAAAATATTAAAAAAAATTGTACAGATGGAAAAGAAAAAGAGAAAA
PfGN01       AATGTATTCTAGAAATGGAGAAAAATATATCCATGAGGCATTGGGAAAATATTAAAAAAAATTGTACAGATGGAAAAGAAAAAGAGAAAA
PfKE01       AATGTATTCTAGAAATGGAGAAGAATATATCCATGAGGCATTGGGAAAATATTAAAAAAAATTGTACAGATGGAAAAGAAAAAGAGAAAA
PfKH02       AATGTATTCTAGAAATGGAGAAGAATATATCCATGAGGCATTGGGAAAATATTAAAAAAAATTGTACAGATGGAAAAGAAAAAGAGAAAA
PfGB4        AATGTATTCTAGAAATGGAGAAGAATATATCCATGAGGCATTGGGAAAATATTAAAAAAAATTGTACAGATGGAAAAGAAAAAGAGAAAA
PfGA01_      ------------------------------------------------------------------------------------------
PfHB3_       AATGTATTCTAGAAATGGAGAAGAATATATCCATGAGGCATTGGGAAAATATTAAAAAAAATTGTACAGATGGAAAAGAAAAAGAGAAAA
PfIT_        AATGTATTCTAGAAATGGAGAAGAATATATCCATGAGGCATTGGGAAAATATTAAAAAAAATTGTACAGATGGAAAAGAAAAAGAGAAAA
```

```
                    6310      6320      6330      6340      6350      6360      6370      6380      6390
            ....|....|....|....|....|....|....|....|....|....|....|....|....|....|....|....|....|....|
PF3D7 CDS   ATATTAAAAATGTGGAAATGATTGAAAATATTGAAAATATTGGAAGCACTGGAAAGATTGAAAATATTA---------GAAACATTGATG
MS822 mRNA  ------------------------------------------------------------------------------------------
FCR3 mRNA   ------------------------------------------------------------------------------------------
AM1898      ATATTAAAAATGTGGAAATGATTGAAAATATTGAAAATATTGGAAGCACTGGAAAGATTGAAAATATTAAAAATATTAGAAACATTGATG
AA235       ATATTAAAAATGTGGAAATGATTGAAAATATTGAAAATATTGGAAGCACTGGAAAGATTGAAAATATTA---------GAAACATTGATG
AA243       ATATTAAAAATGTGGAAATGATTGAAAATATTGAAAATATTGGAAGCACTGGAAAGATTGAAAATATTAAAAATATTAGAAACATTGATG
AA258       ATATTAAAAATGTGGAAATGATTGAAAATATTGAAAATATTGGAAGCACTGGAAAGATTGAAAATATTAAAAATATTAGAAACATTGATG
AA264       ATATTAAAAATGTGGAAATGATTGAAAATATTGAAAATATTGGAAGCACTGGAAAGATTGAAAATATTA---------GAAACATTGATG
AM1737      ATATTAAAAATGTGGAAATGATTGAAAATATTGAAAATATTGGAAGCACTGGAAAGATTGAAAATATTA---------GAAACATTGATG
SC74        ATATTAAAAATGTGGAAATGATTGAAAATATTGGAAGCACTGGAAAGATTGAAAATATTAAAAATATTA---------GAAACATTGATG
TAB166      ATATTAAAAATGTGGAAATGATTGAAAATATTGGAAGCACTGGAAAGATTGAAAATATTAAAAATATTA---------GAAACATTGATG
TAB138      ATATTAAAAATGTGGAAATGATTGAAAATATTGGAAGCACTGGAAAGATTGAAAATATTAAAAATATTA---------GAAACATTGATG
TAB130      ATATTAAAAATGTGGAAATGATTGAAAATATTGAAAATATTGGAAGCACTGGAAAGATTGAAAATATTAAAAATATTAGAAACATTGATG
TAB131      ATATTAAAAATGTGGAAATGATTGAAAATATTGAAAATATTGGAAGCACTGGAAAGATTGAAAATATTA---------GAAACATTGATG
TAB151      ATATTAAAAATGTGGAAATGATTGAAAATATTGAAAATATTGGAAGCACTGGAAAGATTGAAAATATTA---------GAAACATTGATG
TAB152      ATATTAAAAATGTGGAAATGATTGAAAATATTGAAAATATTGGAAGCACTGGAAAGATTGAAAATATTA---------GAAACATTGATG
SC52        ATATTAAAAATGTGGAAATGATTGAAAATATTGAAAATATTGGAAGCACTGGAAAGATTGAAAATATTA---------GAAACATTGATG
SC55        ATATTAAAAATGTGGAAATGATTGAAAATATTGGAAGCACTGGAAAGATTGAAAATATTAAAAATATTA---------GAAACATTGATG
SC03        ATATTAAAAATGTGGAAATGATTGAAAATATTGAAAATATTGGAAGCACTGGAAAGATTGAAAATATTA---------GAAACATTGATG
SC53        ATATTAAAAATGTGGAAATGATTGAAAATATTGAAAATATTGGAAGCACTGGAAAGATTGAAAATATTA---------GAAACATTGATG
TAB141      ATATTAAAAATGTGGAAATGATTGAAAATATTGAAAATATTGGAAGCACTGGAAAGATTGAAAATATTA---------GAAACATTGATG
TAB123      ATATTAAAAATGTGGAAATGATTGAAAATATTGAAAATATTGGAAGCACTGGAAAGATTGAAAATATTAAAAATATTAGAAACATTGATG
AM1803      ATATTAAAAATGTGGAAATGATTGAAAATATTGAAAATATTGGAAGCACTGGAAAGATTGAAAATATTA---------GAAACATTGATG
AM1814      ATATTAAAAATGTGGAAATGATTGAAAATATTGAAAATATTGGAAGCACTGGAAAGATTGAAAATATTAAAAATATTAGAAACATTGATG
AM1802      ATATTAAAAATGTGGAAATGATTGAAAATATTGAAAATATTGGAAGCACTGGAAAGATTGAAAATATTAAAAATATTAGAAACATTGATG
AM1811      ATATTAAAAATGTGGAAATGATTGAAAATATTGAAAATATTGGAAGCACTGGAAAGATTGAAAATATTA---------GAAACATTGATG
SC50        ATATTAAAAATGTGGAAATGATTGAAAATATTGAAAATATTGGAAGCACTGGAAAGATTGAAAATATTA---------GAAACATTGATG
SC56        ATATTAAAAATGTGGAAATGATTGAAAATATTGAAAATATTGGAAGCACTGGAAAGATTGAAAATATTA---------GAAACATTGATG
TAB153      ATATTAAAAATGTGGAAATGATTGAAAATATTGAAAATATTGGAAGCACTGGAAAGATTGAAAATATTA---------GAAACATTGATG
TAB154      ATATTAAAAATGTGGAAATGATTGAAAATATTGAAAATATTGGAAGCACTGGAAAGATTGAAAATATTAAAAATATTAGAAACATTGATG
TAB156      ATATTAAAAATGTGGAAATGATTGAAAATATTGAAAATATTGGAAGCACTGGAAAGATTGAAAATATTAAAAATATTAGAAACATTGATG
TAB136      ATATTAAAAATGTGGAAATGATTGAAAATATTGAAAATATTGGAAGCACTGGAAAGATTGAAAATATTA---------GAAACATTGATG
AM1880      ATATTAAAAATGTGGAAATGATTGAAAATATTGGAAGCACTGGAAAGATTGAAAATATTAAAAATATTA---------GAAACATTGATG
PF3D7_      ATATTAAAAATGTGGAAATGATTGAAAATATTGAAAATATTGGAAGCACTGGAAAGATTGAAAATATTA---------GAAACATTGATG
PfSD01      ATATTAAAAATGTGGAAATGATTGAAAATATTGGAAGCACTGGAAAGATTGAAAATATTAAAAATATTA---------GAAACATTGATG
Pf7G8       ATATTAAAAATGTGGAAATGATTGAAAATATTGAAAATATTGGAAGCACTGGAAAGATTGAAAATATTA---------GAAACATTGATG
FCR3 GENE   ------------------------------------------------------------------------------------------
MS822 GENE  ------------------------------------------------------------------------------------------
PfCD01_     ATATTAAAAATGTGGAAATGATTGAAAATATTGAAAATATTGGAAGCACTGGAAAGATTGAAAATATTA---------GAAACATTGATG
PfKH01__    ATATTAAAAATGTGGAAATGATTGAAAATATTGAAAATATTGGAAGCACTGGAAAGATTGAAAATATTA---------GAAACATTGATG
PfDd2__     ATATTAAAAATGTGGAAATGATTGAAAATATTGAAAATATTGGAAGCACTGGAAAGATTGAAAATATTA---------GAAACATTGATG
PfSN01_     ATATTAAAAATGTGGAAATGATTGAAAATATTGAAAATATTGGAAGCACTGGAAAGATTGAAAATATTA---------GAAACATTGATG
PfGN01      ATATTAAAAATGTGGAAATGATTGAAAATATTGAAAATATTGGAAGCACTGGAAAGATTGAAAATATTA---------GAAACATTGATG
PfKE01      ATATTAAAAATGTGGAAATGATTGAAAATATTGAAAATATTGGAAGCACTGGAAAGATTGAAAATATTA---------GAAACATTGATG
PfKH02      ATATTAAAAATGTGGAAATGATTGAAAATATTGAAAATATTGGAAGCACTGGAAAGATTGAAAATATTA---------GAAACATTGATG
PfGB4       ATATTAAAAATGTGGAAATGATTGAAAATATTGAAAATATTGGAAGCACTGGAAAGATTGAAAATATTA---------GAAACATTGATG
PfGA01_     ------------------------------------------------------------------------------------------
PfHB3_      ATATTAAAAATGTGGAAATGATTGAAAATATTGGAAGCACTGGAAAGATTGAAAATATTAAAAATATTA---------GAAACATTGATG
PfIT_       ATATTAAAAATGTGGAAATGATTGAAAATATTGGAAGCACTGGAAAGATTGAAAATATTAAAAATATTA---------GAAACATTGATG
```

```
                      6400      6410      6420      6430      6440      6450      6460      6470      6480
            ....|....|....|....|....|....|....|....|....|....|....|....|....|....|....|....|....|....|
PF3D7 CDS   AAATAGTAGAAGAAAAATATAAAAGTTTTAATGTAGAACCTTATGAAGAAAACGATTATGAGGAAAAAAGTAAAATATTAAACAGATCAA
MS822 mRNA  ------------------------------------------------------------------------------------------
FCR3 mRNA   ------------------------------------------------------------------------------------------
AM1898      AAATACTAGAAGAAAAATATAAAAGTTTTAATATAGAACCTTATGAAGAAAACAATTATGAGGAAAAAAGTAAAATATTAAACAGATCAA
AA235       AAATACTAGAAGAAAAATATAAAAGTTTTAATGTAGAACCTTATGAAGAAAACAATTATGAGGAAAAAAGTAAAATATTAAACAGATCAA
AA243       AAATACTAGAAGAAAAATATAAAAGTTTTAATGTAGAACCTTATGAAGAAAACAATTATGAGGAAAAAAGTAAAATATTAAACAGATCAA
AA258       AAATACTAGAAGAAAAATATAAAAGTTTTAATGTAGAACCTTATGAAGAAAACAATTATGAGGAAAAAAGTAAAATATTAAACAGATCAA
AA264       AAATAGTAGAAGAAAAATATAAAAGTTTTAATGTAGAACCTTATGAAGAAAACGATTATGAGGAAAAAAGTAAAATATTAAACAGATCAA
AM1737      AAATAGTAGAAGAAAAATATAAAAGTTTTAATGTAGAACCTTATGAAGAAAACGATTATGAGGAAAAAAGTAAAATATTAAACAGATCAA
SC74        AAATACTAGAAGAAAAATATAAAAGTTTTAATATAGAACCTTATGAAGAAAACAATTATGAGGAAAAAAGTAAAATATTAAACAGATCAA
TAB166      AAATACTAGAAGAAAAATATAAAAGTTTTAATATAGAACCTTATGAAGAAAACAATTATGAGGAAAAAAGTAAAATATTAAACAGATCAA
TAB138      AAATACTAGAAGAAAAATATAAAAGTTTTAATATAGAACCTTATGAAGAAAACAATTATGAGGAAAAAAGTAAAATATTAAACAGATCAA
TAB130      AAATACTAGAAGAAAAATATAAAAGTTTTAATGTAGAACCTTATGAAGAAAACAATTATGAGGAAAAAAGTAAAATATTAAACAGATCAA
TAB131      AAATAGTAGAAGAAAAATATAAAAGTTTTAATGTAGAACCTTATGAAGAAAACGATTATGAGGAAAAAAGTAAAATATTAAACAGATCAA
TAB151      AAATAGTAGAAGAAAAATATAAAAGTTTTAATGTAGAACCTTATGAAGAAAACGATTATGAGGAAAAAAGTAAAATATTAAACAGATCAA
TAB152      AAATAGTAGAAGAAAAATATAAAAGTTTTAATGTAGAACCTTATGAAGAAAACGATTATGAGGAAAAAAGTAAAATATTAAACAGATCAA
SC52        AAATAGTAGAAGAAAAATATAAAAGTTTTAATGTAGAACCTTATGAAGAAAACGATTATGAGGAAAAAAGTAAAATATTAAACAGATCAA
SC55        AAATACTAGAAGAAAAATATAAAAGTTTTAATATAGAACCTTATGAAGAAAACAATTATGAGGAAAAAAGTAAAATATTAAACAGATCAA
SC03        AAATACTAGAAGAAAAATATAAAAGTTTTAATGTAGAACCTTATGAAGAAAACAATTATGAGGAAAAAAGTAAAATATTAAACAGATCAA
SC53        AAATAGTAGAAGAAAAATATAAAAGTTTTAATGTAGAACCTTATGAAGAAAACGATTATGAGGAAAAAAGTAAAATATTAAACAGATCAA
TAB141      AAATACTAGAAGAAAAATATAAAAGTTTTAATATAGAACCTTATGAAGAAAACAATTATGAGGAAAAAAGTAAAATATTAAACAGATCAA
TAB123      AAATACTAGAAGAAAAATATAAAAGTTTTAATATAGAACCTTATGAAGAAAACAATTATGAGGAAAAAAGTAAAATATTAAACAGATCAA
AM1803      AAATAGTAGAAGAAAAATATAAAAGTTTTAATGTAGAACCTTATGAAGAAAACGATTATGAGGAAAAAAGTAAAATATTAAACAGATCAA
AM1814      AAATACTAGAAGAAAAATATAAAAGTTTTAATATAGAACCTTATGAAGAAAACAATTATGAGGAAAAAAGTAAAATATTAAACAGATCAA
AM1802      AAATACTAGAAGAAAAATATAAAAGTTTTAATGTAGAACCTTATGAAGAAAACAATTATGAGGAAAAAAGTAAAATATTAAACAGATCAA
AM1811      AAATAGTAGAAGAAAAATATAAAAGTTTTAATGTAGAACCTTATGAAGAAAACGATTATGAGGAAAAAAGTAAAATATTAAACAGATCAA
SC50        AAATAGTAGAAGAAAAATATAAAAGTTTTAATGTAGAACCTTATGAAGAAAACGATTATGAGGAAAAAAGTAAAATATTAAACAGATCAA
SC56        AAATAGTAGAAGAAAAATATAAAAGTTTTAATGTAGAACCTTATGAAGAAAACGATTATGAGGAAAAAAGTAAAATATTAAACAGATCAA
TAB153      AAATAGTAGAAGAAAAATATAAAAGTTTTAATGTAGAACCTTATGAAGAAAACGATTATGAGGAAAAAAGTAAAATATTAAACAGATCAA
TAB154      AAATACTAGAAGAAAAATATAAAAGTTTTAATGTAGAACCTTATGAAGAAAACAATTATGAGGAAAAAAGTAAAATATTAAACAGATCAA
TAB156      AAATACTAGAAGAAAAATATAAAAGTTTTAATGTAGAACCTTATGAAGAAAACAATTATGAGGAAAAAAGTAAAATATTAAACAGATCAA
TAB136      AAATAGTAGAAGAAAAATATAAAAGTTTTAATGTAGAACCTTATGAAGAAAACGATTATGAGGAAAAAAGTAAAATATTAAACAGATCAA
AM1880      AAATACTAGAAGAAAAATATAAAAGTTTTAATATAGAACCTTATGAAGAAAACAATTATGAGGAAAAAAGTAAAATATTAAACAGATCAA
PF3D7_      AAATAGTAGAAGAAAAATATAAAAGTTTTAATGTAGAACCTTATGAAGAAAACGATTATGAGGAAAAAAGTAAAATATTAAACAGATCAA
PfSD01      AAATACTAGAAGAAAAATATAAAAGTTTTAATATAGAACCTTATGAAGAAAACAATTATGAGGAAAAAAGTAAAATATTAAACAGATCAA
Pf7G8       AAATAGTAGAAGAAAAATATAAAAGTTTTAATGTAGAACCTTATGAAGAAAACGATTATGAGGAAAAAAGTAAAATATTAAACAGATCAA
FCR3 GENE   ------------------------------------------------------------------------------------------
MS822 GENE  ------------------------------------------------------------------------------------------
PfCD01_     AAATAGTAGAAGAAAAATATAAAAGTTTTAATGTAGAACCTTATGAAGAAAACGATTATGAGGAAAAAAGTAAAATATTAAACAGATCAA
PfKH01__    AAATAGTAGAAGAAAAATATAAAAGTTTTAATGTAGAACCTTATGAAGAAAACGATTATGAGGAAAAAAGTAAAATATTAAACAGATCAA
PfDd2__     AAATAGTAGAAGAAAAATATAAAAGTTTTAATGTAGAACCTTATGAAGAAAACGATTATGAGGAAAAAAGTAAAATATTAAACAGATCAA
PfSN01_     AAATAGTAGAAGAAAAATATAAAAGTTTTAATGTAGAACCTTATGAAGAAAACGATTATGAGGAAAAAAGTAAAATATTAAACAGATCAA
PfGN01      AAATAGTAGAAGAAAAATATAAAAGTTTTAATGTAGAACCTTATGAAGAAAACGATTATGAGGAAAAAAGTAAAATATTAAACAGATCAA
PfKE01      AAATAGTAGAAGAAAAATATAAAAGTTTTAATGTAGAACCTTATGAAGAAAACGATTATGAGGAAAAAAGTAAAATATTAAACAGATCAA
PfKH02      AAATAGTAGAAGAAAAATATAAAAGTTTTAATGTAGAACCTTATGAAGAAAACGATTATGAGGAAAAAAGTAAAATATTAAACAGATCAA
PfGB4       AAATAGTAGAAGAAAAATATAAAAGTTTTAATGTAGAACCTTATGAAGAAAACGATTATGAGGAAAAAAGTAAAATATTAAACAGATCAA
PfGA01_     ------------------------------------------------------------------------------------------
PfHB3_      AAATACTAGAAGAAAAATATAAAAGTTTTAATATAGAACCTTATGAAGAAAACAATTATGAGGAAAAAAGTAAAATATTAAACAGATCAA
PfIT_       AAATACTAGAAGAAAAATATAAAAGTTTTAATATAGAACCTTATGAAGAAAACAATTATGAGGAAAAAAGTAAAATATTAAACAGATCAA
```

```
                     6490      6500      6510      6520      6530      6540      6550      6560      6570
             ....|....|....|....|....|....|....|....|....|....|....|....|....|....|....|....|....|....|
PF3D7 CDS    ATATCCAGAGTAATAAAATTATAAGCCCATTGATAGATAACGAAAAGGGAATAGATGAAAAAAAGAGTGATAATCAAAATGAAGAAATAT
MS822 mRNA   ------------------------------------------------------------------------------------------
FCR3 mRNA    ------------------------------------------------------------------------------------------
AM1898       ATATCCAAAGTAATAAAATTATAAGCCCATTGATAGATAACGAAAAGGGAATAGATGAAAAAAAGAGTGATAATCAAAATGAAGAAATAT
AA235        ATATCCAAAGTAATAAAATTATAAGCCCATTGATAGATAACGAAAAGGGAATAGATGAAAAAAAGAGTGATAATCAAAATGAAGAAATAT
AA243        ATATCCAAAGTAATAAAATTATAAGCCCATTGATAGATAACGAAAAGGGAATAGATGAAAAAAAGAGTGATAATCAAAATGAAGAAATAT
AA258        ATATCCAAAGTAATAAAATTATAAGCCCATTGATAGATAACGAAAAGGGAATAGATGAAAAAAAGAGTGATAATCAAAATGAAGAAATAT
AA264        ATATCCAAAGTAATAAAATTATAAGCCCATTGATAGATAACGAAAAGGGAATAGATGAAAAAAAGAGTGATAATCAAAATGAAGAAATAT
AM1737       ATATCCAAAGTAATAAAATTATAAGCCCATTGATAGATAACGAAAAGGGAATAGATGAAAAAAAGAGTGATAATCAAAATGAAGAAATAT
SC74         ATATCCAAAGTAATAAAATTATAAGCCCATTGATAGATAACGAAAAGGGAATAGATGAAAAAAAGAGTGATAATCAAAATGAAGAAATAT
TAB166       ATATCCAAAGTAATAAAATTATAAGCCCATTGATAGATAACGAAAAGGGAATAGATGAAAAAAAGAGTGATAATCAAAATGAAGAAATAT
TAB138       ATATCCAAAGTAATAAAATTATAAGCCCATTGATAGATAACGAAAAGGGAATAGATGAAAAAAAGAGTGATAATCAAAATGAAGAAATAT
TAB130       ATATCCAAAGTAATAAAATTATAAGCCCATTGATAGATAACGAAAAGGGAATAGATGAAAAAAAGAGTGATAATCAAAATGAAGAAATAT
TAB131       ATATCCAAAGTAATAAAATTATAAGCCCATTGATAGATAACGAAAAGGGAATAGATGAAAAAAAGAGTGATAATCAAAATGAAGAAATAT
TAB151       ATATCCAAAGTAATAAAATTATAAGCCCATTGATAGATAACGAAAAGGGAATAGATGAAAAAAAGAGTGATAATCAAAATGAAGAAATAT
TAB152       ATATCCAAAGTAATAAAATTATAAGCCCATTGATAGATAACGAAAAGGGAATAGATGAAAAAAAGAGTGATAATCAAAATGAAGAAATAT
SC52         ATATCCAAAGTAATAAAATTATAAGCCCATTGATAGATAACGAAAAGGGAATAGATGAAAAAAAGAGTGATAATCAAAATGAAGAAATAT
SC55         ATATCCAAAGTAATAAAATTATAAGCCCATTGATAGATAACGAAAAGGGAATAGATGAAAAAAAGAGTGATAATCAAAATGAAGAAATAT
SC03         ATATCCAAAGTAATAAAATTATAAGCCCATTGATAGATAACGAAAAGGGAATAGATGAAAAAAAGAGTGATAATCAAAATGAAGAAATAT
SC53         ATATCCAAAGTAATAAAATTATAAGCCCATTGATAGATAACGAAAAGGGAATAGATGAAAAAAAGAGTGATAATCAAAATGAAGAAATAT
TAB141       ATATCCAAAGTAATAAAATTATAAGCCCATTGATAGATAACGAAAAGGGAATAGATGAAAAAAAGAGTGATAATCAAAATGAAGAAATAT
TAB123       ATATCCAAAGTAATAAAATTATAAGCCCATTGATAGATAACGAAAAGGGAATAGATGAAAAAAAGAGTGATAATCAAAATGAAGAAATAT
AM1803       ATATCCAAAGTAATAAAATTATAAGCCCATTGATAGATAACGAAAAGGGAATAGATGAAAAAAAGAGTGATAATCAAAATGAAGAAATAT
AM1814       ATATCCAGAGTAATAAAATTATAAGCCCATTGATAGATAACGAAAAGGGAATAGATGAAAAAAAGAGTGATAATCAAAATGAAGAAATAT
AM1802       ATATCCAAAGTAATAAAATTATAAGCCCATTGATAGATAACGAAAAGGGAATAGATGAAAAAAAGAGTGATAATCAAAATGAAGAAATAT
AM1811       ATATCCAAAGTAATAAAATTATAAGCCCATTGATAGATAACGAAAAGGGAATAGATGAAAAAAAGAGTGATAATCAAAATGAAGAAATAT
SC50         ATATCCAAAGTAATAAAATTATAAGCCCATTGATAGATAACGAAAAGGGAATAGATGAAAAAAAGAGTGATAATCAAAATGAAGAAATAT
SC56         ATATCCAAAGTAATAAAATTATAAGCCCATTGATAGATAACGAAAAGGGAATAGATGAAAAAAAGAGTGATAATCAAAATGAAGAAATAT
TAB153       ATATCCAAAGTAATAAAATTATAAGCCCATTGATAGATAACGAAAAGGGAATAGATGAAAAAAAGAGTGATAATCAAAATGAAGAAATAT
TAB154       ATATCCAAAGTAATAAAATTATAAGCCCATTGATAGATAACGAAAAGGGAATAGATGAAAAAAAGAGTGATAATCAAAATGAAGAAATAT
TAB156       ATATCCAAAGTAATAAAATTATAAGCCCATTGATAGATAACGAAAAGGGAATAGATGAAAAAAAGAGTGATAATCAAAATGAAGAAATAT
TAB136       ATATCCAAAGTAATAAAATTATAAGCCCATTGATAGATAACGAAAAGGGAATAGATGAAAAAAAGAGTGATAATCAAAATGAAGAAATAT
AM1880       ATATCCAGAGTAATAAAATTATAAGCCCATTGATAGATAACGAAAAGGGAATAGATGAAAAAAAGAGTGATAATCAAAATGAAGAAATAT
PF3D7_       ATATCCAGAGTAATAAAATTATAAGCCCATTGATAGATAACGAAAAGGGAATAGATGAAAAAAAGAGTGATAATCAAAATGAAGAAATAT
PfSD01       ATATCCAGAGTAATAAAATTATAAGCCCATTGATAGATAACGAAAAGGGAATAGATGAAAAAAAGAGTGATAATCAAAATGAAGAAATAT
Pf7G8        ATATCCAAAGTAATAAAATTATAAGCCCATTGATAGATAACGAAAAGGGAATAGATGAAAAAAAGAGTGATAATCAAAATGAAGAAATAT
FCR3 GENE    ------------------------------------------------------------------------------------------
MS822 GENE   ------------------------------------------------------------------------------------------
PfCD01_      ATATCCAAAGTAATAAAATTATAAGCCCATTGATAGATAACGAAAAGGGAATAGATGAAAAAAAGAGTGATAATCAAAATGAAGAAATAT
PfKH01__     ATATCCAAAGTAATAAAATTATAAGCCCATTGATAGATAACGAAAAGGGAATAGATGAAAAAAAGAGTGATAATCAAAATGAAGAAATAT
PfDd2__      ATATCCAAAGTAATAAAATTATAAGCCCATTGATAGATAACGAAAAGGGAATAGATGAAAAAAAGAGTGATAATCAAAATGAAGAAATAT
PfSN01_      ATATCCAAAGTAATAAAATTATAAGCCCATTGATAGATAACGAAAAGGGAATAGATGAAAAAAAGAGTGATAATCAAAATGAAGAAATAT
PfGN01       ATATCCAAAGTAATAAAATTATAAGCCCATTGATAGATAACGAAAAGGGAATAGATGAAAAAAAGAGTGATAATCAAAATGAAGAAATAT
PfKE01       ATATCCAAAGTAATAAAATTATAAGCCCATTGATAGATAACGAAAAGGGAATAGATGAAAAAAAGAGTGATAATCAAAATGAAGAAATAT
PfKH02       ATATCCAAAGTAATAAAATTATAAGCCCATTGATAGATAACGAAAAGGGAATAGATGAAAAAAAGAGTGATAATCAAAATGAAGAAATAT
PfGB4        ATATCCAAAGTAATAAAATTATAAGCCCATTGATAGATAACGAAAAGGGAATAGATGAAAAAAAGAGTGATAATCAAAATGAAGAAATAT
PfGA01_      ------------------------------------------------------------------------------------------
PfHB3_       ATATCCAGAGTAATAAAATTATAAGCCCATTGATAGATAACGAAAAGGGAATAGATGAAAAAAAGAGTGATAATCAAAATGAAGAAATAT
PfIT_        ATATCCAGAGTAATAAAATTATAAGCCCATTGATAGATAACGAAAAGGGAATAGATGAAAAAAAGAGTGATAATCAAAATGAAGAAATAT
```

```
                   6580      6590      6600      6610      6620      6630      6640      6650      6660
           ....|....|....|....|....|....|....|....|....|....|....|....|....|....|....|....|....|....|
PF3D7 CDS  GGTATAACGGATTAACATTAGAAGAGATATATAAAAATTCGTATACAAGAAACTCAATAGAATACATTCCATATATTGACGAAGAGGAAT
MS822 mRNA ------------------------------------------------------------------------------------------
FCR3 mRNA  ------------------------------------------------------------------------------------------
AM1898     GGTATAACGGATTAACATTAGAAGAGATATATAAAAATTCGTATACAAGAAACTCAATAGAATACATTCCATATATTGACGAAGAGGAAT
AA235      GGTATAACGGATTAACATTAGAAGAGATATATAAAAATTCGTATACAAGAAACTCAATAGAATACATTCCATATATTGACGAAGAGGAAT
AA243      GGTATAACGGATTAACATTAGAAGAGATATATAAAAATTCGTATACAAGAAACTCAATAGAATACATTCCATATATTGACGAAGAGGAAT
AA258      GGTATAACGGATTAACATTAGAAGAGATATATAAAAATTCGTATACAAGAAACTCAATAGAATACATTCCATATATTGACGAAGAGGAAT
AA264      GGTATAACGGATTAACATTAGAAGAGATATATAAAAATTCGTATACAAGAAACTCAATAGAATACATTCCATATATTGACGAAGAGGAAT
AM1737     GGTATAACGGATTAACATTAGAAGAGATATATAAAAATTCGTATACAAGAAACTCAATAGAATACATTCCATATATTGACGAAGAGGAAT
SC74       GGTATAACGGATTAACATTAGAAGAGATATATAAAAATTCGTATACAAGAAACTCAATAGAATACATTCCATATATTGACGAAGAGGAAT
TAB166     GGTATAACGGATTAACATTAGAAGAGATATATAAAAATTCGTATACAAGAAACTCAATAGAATACATTCCATATATTGACGAAGAGGAAT
TAB138     GGTATAACGGATTAACATTAGAAGAGATATATAAAAATTCGTATACAAGAAACTCAATAGAATACATTCCATATATTGACGAAGAGGAAT
TAB130     GGTATAACGGATTAACATTAGAAGAGATATATAAAAATTCGTATACAAGAAACTCAATAGAATACATTCCATATATTGACGAAGAGGAAT
TAB131     GGTATAACGGATTAACATTAGAAGAGATATATAAAAATTCGTATACAAGAAACTCAATAGAATACATTCCATATATTGACGAAGAGGAAT
TAB151     GGTATAACGGATTAACATTAGAAGAGATATATAAAAATTCGTATACAAGAAACTCAATAGAATACATTCCATATATTGACGAAGAGGAAT
TAB152     GGTATAACGGATTAACATTAGAAGAGATATATAAAAATTCGTATACAAGAAACTCAATAGAATACATTCCATATATTGACGAAGAGGAAT
SC52       GGTATAACGGATTAACATTAGAAGAGATATATAAAAATTCGTATACAAGAAACTCAATAGAATACATTCCATATATTGACGAAGAGGAAT
SC55       GGTATAACGGATTAACATTAGAAGAGATATATAAAAATTCGTATACAAGAAACTCAATAGAATACATTCCATATATTGACGAAGAGGAAT
SC03       GGTATAACGGATTAACATTAGAAGAGATATATAAAAATTCGTATACAAGAAACTCAATAGAATACATTCCATATATTGACGAAGAGGAAT
SC53       GGTATAACGGATTAACATTAGAAGAGATATATAAAAATTCGTATACAAGAAACTCAATAGAATACATTCCATATATTGACGAAGAGGAAT
TAB141     GGTATAACGGATTAACATTAGAAGAGATATATAAAAATTCGTATACAAGAAACTCAATAGAATACATTCCATATATTGACGAAGAGGAAT
TAB123     GGTATAACGGATTAACATTAGAAGAGATATATAAAAATTCGTATACAAGAAACTCAATAGAATACATTCCATATATTGACGAAGAGGAAT
AM1803     GGTATAACGGATTAACATTAGAAGAGATATATAAAAATTCGTATACAAGAAACTCAATAGAATACATTCCATATATTGACGAAGAGGAAT
AM1814     GGTATAACGGATTAACATTAGAAGAGATATATAAAAATTCGTATACAAGAAACTCAATAGAATACATTCCATATATTGACGAAGAGGAAT
AM1802     GGTATAACGGATTAACATTAGAAGAGATATATAAAAATTCGTATACAAGAAACTCAATAGAATACATTCCATATATTGACGAAGAGGAAT
AM1811     GGTATAACGGATTAACATTAGAAGAGATATATAAAAATTCGTATACAAGAAACTCAATAGAATACATTCCATATATTGACGAAGAGGAAT
SC50       GGTATAACGGATTAACATTAGAAGAGATATATAAAAATTCGTATACAAGAAACTCAATAGAATACATTCCATATATTGACGAAGAGGAAT
SC56       GGTATAACGGATTAACATTAGAAGAGATATATAAAAATTCGTATACAAGAAACTCAATAGAATACATTCCATATATTGACGAAGAGGAAT
TAB153     GGTATAACGGATTAACATTAGAAGAGATATATAAAAATTCGTATACAAGAAACTCAATAGAATACATTCCATATATTGACGAAGAGGAAT
TAB154     GGTATAACGGATTAACATTAGAAGAGATATATAAAAATTCGTATACAAGAAACTCAATAGAATACATTCCATATATTGACGAAGAGGAAT
TAB156     GGTATAACGGATTAACATTAGAAGAGATATATAAAAATTCGTATACAAGAAACTCAATAGAATACATTCCATATATTGACGAAGAGGAAT
TAB136     GGTATAACGGATTAACATTAGAAGAGATATATAAAAATTCGTATACAAGAAACTCAATAGAATACATTCCATATATTGACGAAGAGGAAT
AM1880     GGTATAACGGATTAACATTAGAAGAGATATATAAAAATTCGTATACAAGAAACTCAATAGAATACATTCCATATATTGACGAAGAGGAAT
PF3D7_     GGTATAACGGATTAACATTAGAAGAGATATATAAAAATTCGTATACAAGAAACTCAATAGAATACATTCCATATATTGACGAAGAGGAAT
PfSD01     GGTATAACGGATTAACATTAGAAGAGATATATAAAAATTCGTATACAAGAAACTCAATAGAATACATTCCATATATTGACGAAGAGGAAT
Pf7G8      GGTATAACGGATTAACATTAGAAGAGATATATAAAAATTCGTATACAAGAAACTCAATAGAATACATTCCATATATTGACGAAGAGGAAT
FCR3 GENE  ------------------------------------------------------------------------------------------
MS822 GENE ------------------------------------------------------------------------------------------
PfCD01_    GGTATAACGGATTAACATTAGAAGAGATATATAAAAATTCGTATACAAGAAACTCAATAGAATACATTCCATATATTGACGAAGAGGAAT
PfKH01__   GGTATAACGGATTAACATTAGAAGAGATATATAAAAATTCGTATACAAGAAACTCAATAGAATACATTCCATATATTGACGAAGAGGAAT
PfDd2__    GGTATAACGGATTAACATTAGAAGAGATATATAAAAATTCGTATACAAGAAACTCAATAGAATACATTCCATATATTGACGAAGAGGAAT
PfSN01_    GGTATAACGGATTAACATTAGAAGAGATATATAAAAATTCGTATACAAGAAACTCAATAGAATACATTCCATATATTGACGAAGAGGAAT
PfGN01     GGTATAACGGATTAACATTAGAAGAGATATATAAAAATTCGTATACAAGAAACTCAATAGAATACATTCCATATATTGACGAAGAGGAAT
PfKE01     GGTATAACGGATTAACATTAGAAGAGATATATAAAAATTCGTATACAAGAAACTCAATAGAATACATTCCATATATTGACGAAGAGGAAT
PfKH02     GGTATAACGGATTAACATTAGAAGAGATATATAAAAATTCGTATACAAGAAACTCAATAGAATACATTCCATATATTGACGAAGAGGAAT
PfGB4      GGTATAACGGATTAACATTAGAAGAGATATATAAAAATTCGTATACAAGAAACTCAATAGAATACATTCCATATATTGACGAAGAGGAAT
PfGA01_    ------------------------------------------------------------------------------------------
PfHB3_     GGTATAACGGATTAACATTAGAAGAGATATATAAAAATTCGTATACAAGAAACTCAATAGAATACATTCCATATATTGACGAAGAGGAAT
PfIT_      GGTATAACGGATTAACATTAGAAGAGATATATAAAAATTCGTATACAAGAAACTCAATAGAATACATTCCATATATTGACGAAGAGGAAT
```

```
                  6670      6680      6690      6700      6710      6720      6730      6740      6750
             ....|....|....|....|....|....|....|....|....|....|....|....|....|....|....|....|....|....|
PF3D7 CDS    ATAGTTCATTAGATGAACTGGATCAAAAAAACAATGATTTTGAAAGCGATGATATGTTTTATAAAGGTTTAACATTAGAAGAGATATATA
MS822 mRNA   ------------------------------------------------------------------------------------------
FCR3 mRNA    ------------------------------------------------------------------------------------------
AM1898       ATAGTTCATTAGATGAACTGGATCAAAAAAACAATGATTTTGAAAGCGATGATATGTTTTATAAAGGTTTAACATTAGAAGAGATATATA
AA235        ATAGTTCATTAGATGAACTGGATCAAAAAAACAATGATTTTGAAAGCGATGATATGTTTTATAAAGGTTTAACATTAGAAGAGATATATA
AA243        ATAGTTCATTAGATGAACTGGATCAAAAAAACAATGATTTTGAAAGCGATGATATGTTTTATAAAGGTTTAACATTAGAAGAGATATATA
AA258        ATAGTTCATTAGATGAACTGGATCAAAAAAACAATGATTTTGAAAGCGATGATATGTTTTATAAAGGTTTAACATTAGAAGAGATATATA
AA264        ATAGTTCATTAGATGAACTGGATCAAAAAAACAATGATTTTGAAAGCGATGATATGTTTTATAAAGGTTTAACATTAGAAGAGATATATA
AM1737       ATAGTTCATTAGATGAACTGGATCAAAAAAACAATGATTTTGAAAGCGATGATATGTTTTATAAAGGTTTAACATTAGAAGAGATATATA
SC74         ATAGTTCATTAGATGAACTGGATCAAAAAAACAATGATTTTGAAAGCGATGATATGTTTTATAAAGGTTTAACATTAGAAGAGATATATA
TAB166       ATAGTTCATTAGATGAACTGGATCAAAAAAACAATGATTTTGAAAGCGATGATATGTTTTATAAAGGTTTAACATTAGAAGAGATATATA
TAB138       ATAGTTCATTAGATGAACTGGATCAAAAAAACAATGATTTTGAAAGCGATGATATGTTTTATAAAGGTTTAACATTAGAAGAGATATATA
TAB130       ATAGTTCATTAGATGAACTGGATCAAAAAAACAATGATTTTGAAAGCGATGATATGTTTTATAAAGGTTTAACATTAGAAGAGATATATA
TAB131       ATAGTTCATTAGATGAACTGGATCAAAAAAACAATGATTTTGAAAGCGATGATATGTTTTATAAAGGTTTAACATTAGAAGAGATATATA
TAB151       ATAGTTCATTAGATGAACTGGATCAAAAAAACAATGATTTTGAAAGCGATGATATGTTTTATAAAGGTTTAACATTAGAAGAGATATATA
TAB152       ATAGTTCATTAGATGAACTGGATCAAAAAAACAATGATTTTGAAAGCGATGATATGTTTTATAAAGGTTTAACATTAGAAGAGATATATA
SC52         ATAGTTCATTAGATGAACTGGATCAAAAAAACAATGATTTTGAAAGCGATGATATGTTTTATAAAGGTTTAACATTAGAAGAGATATATA
SC55         ATAGTTCATTAGATGAACTGGATCAAAAAAACAATGATTTTGAAAGCGATGATATGTTTTATAAAGGTTTAACATTAGAAGAGATATATA
SC03         ATAGTTCATTAGATGAACTGGATCAAAAAAACAATGATTTTGAAAGCGATGATATGTTTTATAAAGGTTTAACATTAGAAGAGATATATA
SC53         ATAGTTCATTAGATGAACTGGATCAAAAAAACAATGATTTTGAAAGCGATGATATGTTTTATAAAGGTTTAACATTAGAAGAGATATATA
TAB141       ATAGTTCATTAGATGAACTGGATCAAAAAAACAATGATTTTGAAAGCGATGATATGTTTTATAAAGGTTTAACATTAGAAGAGATATATA
TAB123       ATAGTTCATTAGATGAACTGGATCAAAAAAACAATGATTTTGAAAGCGATGATATGTTTTATAAAGGTTTAACATTAGAAGAGATATATA
AM1803       ATAGTTCATTAGATGAACTGGATCAAAAAAACAATGATTTTGAAAGCGATGATATGTTTTATAAAGGTTTAACATTAGAAGAGATATATA
AM1814       ATAGTTCATTAGATGAACTGGATCAAAAAAACAATGATTTTGAAAGCGATGATATGTTTTATAAAGGTTTAACATTAGAAGAGATATATA
AM1802       ATAGTTCATTAGATGAACTGGATCAAAAAAACAATGATTTTGAAAGCGATGATATGTTTTATAAAGGTTTAACATTAGAAGAGATATATA
AM1811       ATAGTTCATTAGATGAACTGGATCAAAAAAACAATGATTTTGAAAGCGATGATATGTTTTATAAAGGTTTAACATTAGAAGAGATATATA
SC50         ATAGTTCATTAGATGAACTGGATCAAAAAAACAATGATTTTGAAAGCGATGATATGTTTTATAAAGGTTTAACATTAGAAGAGATATATA
SC56         ATAGTTCATTAGATGAACTGGATCAAAAAAACAATGATTTTGAAAGCGATGATATGTTTTATAAAGGTTTAACATTAGAAGAGATATATA
TAB153       ATAGTTCATTAGATGAACTGGATCAAAAAAACAATGATTTTGAAAGCGATGATATGTTTTATAAAGGTTTAACATTAGAAGAGATATATA
TAB154       ATAGTTCATTAGATGAACTGGATCAAAAAAACAATGATTTTGAAAGCGATGATATGTTTTATAAAGGTTTAACATTAGAAGAGATATATA
TAB156       ATAGTTCATTAGATGAACTGGATCAAAAAAACAATGATTTTGAAAGCGATGATATGTTTTATAAAGGTTTAACATTAGAAGAGATATATA
TAB136       ATAGTTCATTAGATGAACTGGATCAAAAAAACAATGATTTTGAAAGCGATGATATGTTTTATAAAGGTTTAACATTAGAAGAGATATATA
AM1880       ATAGTTCATTAGATGAACTGGATCAAAAAAACAATGATTTTGAAAGCGATGATATGTTTTATAAAGGTTTAACATTAGAAGAGATATATA
PF3D7_       ATAGTTCATTAGATGAACTGGATCAAAAAAACAATGATTTTGAAAGCGATGATATGTTTTATAAAGGTTTAACATTAGAAGAGATATATA
PfSD01       ATAGTTCATTAGATGAACTGGATCAAAAAAACAATGATTTTGAAAGCGATGATATGTTTTATAAAGGTTTAACATTAGAAGAGATATATA
Pf7G8        ATAGTTCATTAGATGAACTGGATCAAAAAAACAATGATTTTGAAAGCGATGATATGTTTTATAAAGGTTTAACATTAGAAGAGATATATA
FCR3 GENE    ------------------------------------------------------------------------------------------
MS822 GENE   ------------------------------------------------------------------------------------------
PfCD01_      ATAGTTCATTAGATGAACTGGATCAAAAAAACAATGATTTTGAAAGCGATGATATGTTTTATAAAGGTTTAACATTAGAAGAGATATATA
PfKH01__     ATAGTTCATTAGATGAACTGGATCAAAAAAACAATGATTTTGAAAGCGATGATATGTTTTATAAAGGTTTAACATTAGAAGAGATATATA
PfDd2__      ATAGTTCATTAGATGAACTGGATCAAAAAAACAATGATTTTGAAAGCGATGATATGTTTTATAAAGGTTTAACATTAGAAGAGATATATA
PfSN01_      ATAGTTCATTAGATGAACTGGATCAAAAAAACAATGATTTTGAAAGCGATGATATGTTTTATAAAGGTTTAACATTAGAAGAGATATATA
PfGN01       ATAGTTCATTAGATGAACTGGATCAAAAAAACAATGATTTTGAAAGCGATGATATGTTTTATAAAGGTTTAACATTAGAAGAGATATATA
PfKE01       ATAGTTCATTAGATGAACTGGATCAAAAAAACAATGATTTTGAAAGCGATGATATGTTTTATAAAGGTTTAACATTAGAAGAGATATATA
PfKH02       ATAGTTCATTAGATGAACTGGATCAAAAAAACAATGATTTTGAAAGCGATGATATGTTTTATAAAGGTTTAACATTAGAAGAGATATATA
PfGB4        ATAGTTCATTAGATGAACTGGATCAAAAAAACAATGATTTTGAAAGCGATGATATGTTTTATAAAGGTTTAACATTAGAAGAGATATATA
PfGA01_      ------------------------------------------------------------------------------------------
PfHB3_       ATAGTTCATTAGATGAACTGGATCAAAAAAACAATGATTTTGAAAGCGATGATATGTTTTATAAAGGTTTAACATTAGAAGAGATATATA
PfIT_        ATAGTTCATTAGATGAACTGGATCAAAAAAACAATGATTTTGAAAGCGATGATATGTTTTATAAAGGTTTAACATTAGAAGAGATATATA
```

```
                  6760      6770      6780      6790      6800      6810      6820      6830      6840
         ....|....|....|....|....|....|....|....|....|....|....|....|....|....|....|....|....|....|
PF3D7 CDS   AAAATGCGTATACAAGAAACTCTACAGAATACATTCCTTATATTGACGAAGAGGAATATAGTTCTATGGATGAATTTGATAAACATAATA
MS822 mRNA  ------------------------------------------------------------------------------------------
FCR3 mRNA   ------------------------------------------------------------------------------------------
AM1898      AAAATGCGTATACAAGAAACTCTACAGAATACATTCCTTATATTGACGAAGAGGAATATAGTTCTATGGATGAATTTGATAAACATAATA
AA235       AAAATGCGTATACAAGAAACTCTACAGAATACATTCCTTATATTGACGAAGAGGAATATAGTTCTATGGATGAATTTGATAAACATAATA
AA243       AAAATGCGTATACAAGAAACTCTACAGAATACATTCCTTATATTGACGAAGAGGAATATAGTTCTATGGATGAATTTGATAAACATAATA
AA258       AAAATGCGTATACAAGAAACTCTACAGAATACATTCCTTATATTGACGAAGAGGAATATAGTTCTATGGATGAATTTGATAAACATAATA
AA264       AAAATGCGTATACAAGAAACTCTACAGAATACATTCCTTATATTGACGAAGAGGAATATAGTTCTATGGATGAATTTGATAAACATAATA
AM1737      AAAATGCGTATACAAGAAACTCTACAGAATACATTCCTTATATTGACGAAGAGGAATATAGTTCTATGGATGAATTTGATAAACATAATA
SC74        AAAATGCGTATACAAGAAACTCTACAGAATACATTCCTTATATTGACGAAGAGGAATATAGTTCTATGGATGAATTTGATAAACATAATA
TAB166      AAAATGCGTATACAAGAAACTCTACAGAATACATTCCTTATATTGACGAAGAGGAATATAGTTCTATGGATGAATTTGATAAACATAATA
TAB138      AAAATGCGTATACAAGAAACTCTACAGAATACATTCCTTATATTGACGAAGAGGAATATAGTTCTATGGATGAATTTGATAAACATAATA
TAB130      AAAATGCGTATACAAGAAACTCTACAGAATACATTCCTTATATTGACGAAGAGGAATATAGTTCTATGGATGAATTTGATAAACATAATA
TAB131      AAAATGCGTATACAAGAAACTCTACAGAATACATTCCTTATATTGACGAAGAGGAATATAGTTCTATGGATGAATTTGATAAACATAATA
TAB151      AAAATGCGTATACAAGAAACTCTACAGAATACATTCCTTATATTGACGAAGAGGAATATAGTTCTATGGATGAATTTGATAAACATAATA
TAB152      AAAATGCGTATACAAGAAACTCTACAGAATACATTCCTTATATTGACGAAGAGGAATATAGTTCTATGGATGAATTTGATAAACATAATA
SC52        AAAATGCGTATACAAGAAACTCTACAGAATACATTCCTTATATTGACGAAGAGGAATATAGTTCTATGGATGAATTTGATAAACATAATA
SC55        AAAATGCGTATACAAGAAACTCTACAGAATACATTCCTTATATTGACGAAGAGGAATATAGTTCTATGGATGAATTTGATAAACATAATA
SC03        AAAATGCGTATACAAGAAACTCTACAGAATACATTCCTTATATTGACGAAGAGGAATATAGTTCTATGGATGAATTTGATAAACATAATA
SC53        AAAATGCGTATACAAGAAACTCTACAGAATACATTCCTTATATTGACGAAGAGGAATATAGTTCTATGGATGAATTTGATAAACATAATA
TAB141      AAAATGCGTATACAAGAAACTCTACAGAATACATTCCTTATATTGACGAAGAGGAATATAGTTCTATGGATGAATTTGATAAACATAATA
TAB123      AAAATGCGTATACAAGAAACTCTACAGAATACATTCCTTATATTGACGAAGAGGAATATAGTTCTATGGATGAATTTGATAAACATAATA
AM1803      AAAATGCGTATACAAGAAACTCTACAGAATACATTCCTTATATTGACGAAGAGGAATATAGTTCTATGGATGAATTTGATAAACATAATA
AM1814      AAAATGCGTATACAAGAAACTCTACAGAATACATTCCTTATATTGACGAAGAGGAATATAGTTCTATGGATGAATTTGATAAACATAATA
AM1802      AAAATGCGTATACAAGAAACTCTACAGAATACATTCCTTATATTGACGAAGAGGAATATAGTTCTATGGATGAATTTGATAAACATAATA
AM1811      AAAATGCGTATACAAGAAACTCTACAGAATACATTCCTTATATTGACGAAGAGGAATATAGTTCTATGGATGAATTTGATAAACATAATA
SC50        AAAATGCGTATACAAGAAACTCTACAGAATACATTCCTTATATTGACGAAGAGGAATATAGTTCTATGGATGAATTTGATAAACATAATA
SC56        AAAATGCGTATACAAGAAACTCTACAGAATACATTCCTTATATTGACGAAGAGGAATATAGTTCTATGGATGAATTTGATAAACATAATA
TAB153      AAAATGCGTATACAAGAAACTCTACAGAATACATTCCTTATATTGACGAAGAGGAATATAGTTCTATGGATGAATTTGATAAACATAATA
TAB154      AAAATGCGTATACAAGAAACTCTACAGAATACATTCCTTATATTGACGAAGAGGAATATAGTTCTATGGATGAATTTGATAAACATAATA
TAB156      AAAATGCGTATACAAGAAACTCTACAGAATACATTCCTTATATTGACGAAGAGGAATATAGTTCTATGGATGAATTTGATAAACATAATA
TAB136      AAAATGCGTATACAAGAAACTCTACAGAATACATTCCTTATATTGACGAAGAGGAATATAGTTCTATGGATGAATTTGATAAACATAATA
AM1880      AAAATGCGTATACAAGAAACTCTACAGAATACATTCCTTATATTGACGAAGAGGAATATAGTTCTATGGATGAATTTGATAAACATAATA
PF3D7_      AAAATGCGTATACAAGAAACTCTACAGAATACATTCCTTATATTGACGAAGAGGAATATAGTTCTATGGATGAATTTGATAAACATAATA
PfSD01      AAAATGCGTATACAAGAAACTCTACAGAATACATTCCTTATATTGACGAAGAGGAATATAGTTCTATGGATGAATTTGATAAACATAATA
Pf7G8       AAAATGCGTATACAAGAAACTCTACAGAATACATTCCTTATATTGACGAAGAGGAATATAGTTCTATGGATGAATTTGATAAACATAATA
FCR3 GENE   ------------------------------------------------------------------------------------------
MS822 GENE  ------------------------------------------------------------------------------------------
PfCD01_     AAAATGCGTATACAAGAAACTCTACAGAATACATTCCTTATATTGACGAAGAGGAATATAGTTCTATGGATGAATTTGATAAACATAATA
PfKH01__    AAAATGCGTATACAAGAAACTCTACAGAATACATTCCTTATATTGACGAAGAGGAATATAGTTCTATGGATGAATTTGATAAACATAATA
PfDd2__     AAAATGCGTATACAAGAAACTCTACAGAATACATTCCTTATATTGACGAAGAGGAATATAGTTCTATGGATGAATTTGATAAACATAATA
PfSN01_     AAAATGCGTATACAAGAAACTCTACAGAATACATTCCTTATATTGACGAAGAGGAATATAGTTCTATGGATGAATTTGATAAACATAATA
PfGN01      AAAATGCGTATACAAGAAACTCTACAGAATACATTCCTTATATTGACGAAGAGGAATATAGTTCTATGGATGAATTTGATAAACATAATA
PfKE01      AAAATGCGTATACAAGAAACTCTACAGAATACATTCCTTATATTGACGAAGAGGAATATAGTTCTATGGATGAATTTGATAAACATAATA
PfKH02      AAAATGCGTATACAAGAAACTCTACAGAATACATTCCTTATATTGACGAAGAGGAATATAGTTCTATGGATGAATTTGATAAACATAATA
PfGB4       AAAATGCGTATACAAGAAACTCTACAGAATACATTCCTTATATTGACGAAGAGGAATATAGTTCTATGGATGAATTTGATAAACATAATA
PfGA01_     ------------------------------------------------------------------------------------------
PfHB3_      AAAATGCGTATACAAGAAACTCTACAGAATACATTCCTTATATTGACGAAGAGGAATATAGTTCTATGGATGAATTTGATAAACATAATA
PfIT_       AAAATGCGTATACAAGAAACTCTACAGAATACATTCCTTATATTGACGAAGAGGAATATAGTTCTATGGATGAATTTGATAAACATAATA
```

```
                          6850
               ....|....|....|..
PF3D7 CDS      ATAAAAGAAAAAAATAA
MS822 mRNA     -----------------
FCR3 mRNA      -----------------
AM1898         ATAAAAGAAAAAAATAA
AA235          ATAAAAGAAAAAAATAA
AA243          ATAAAAGAAAAAAATAA
AA258          ATAAAAGAAAAAAATAA
AA264          ATAAAAGAAAAAAATAA
AM1737         ATAAAAGAAAAAAATAA
SC74           ATAAAAGAAAAAAATAA
TAB166         ATAAAAGAAAAAAATAA
TAB138         ATAAAAGAAAAAAATAA
TAB130         ATAAAAGAAAAAAATAA
TAB131         ATAAAAGAAAAAAATAA
TAB151         ATAAAAGAAAAAAATAA
TAB152         ATAAAAGAAAAAAATAA
SC52           ATAAAAGAAAAAAATAA
SC55           ATAAAAGAAAAAAATAA
SC03           ATAAAAGAAAAAAATAA
SC53           ATAAAAGAAAAAAATAA
TAB141         ATAAAAGAAAAAAATAA
TAB123         ATAAAAGAAAAAAATAA
AM1803         ATAAAAGAAAAAAATAA
AM1814         ATAAAAGAAAAAAATAA
AM1802         ATAAAAGAAAAAAATAA
AM1811         ATAAAAGAAAAAAATAA
SC50           ATAAAAGAAAAAAATAA
SC56           ATAAAAGAAAAAAATAA
TAB153         ATAAAAGAAAAAAATAA
TAB154         ATAAAAGAAAAAAATAA
TAB156         ATAAAAGAAAAAAATAA
TAB136         ATAAAAGAAAAAAATAA
AM1880         ATAAAAGAAAAAAATAA
PF3D7_         ATAAAAGAAAAAAATAA
PfSD01         ATAAAAGAAAAAAATAA
Pf7G8          ATAAAAGAAAAAAATAA
FCR3 GENE      -----------------
MS822 GENE     -----------------
PfCD01_        ATAAAAGAAAAAAATAA
PfKH01__       ATAAAAGAAAAAAATAA
PfDd2__        ATAAAAGAAAAAAATAA
PfSN01_        ATAAAAGAAAAAAATAA
PfGN01         ATAAAAGAAAAAAATAA
PfKE01         ATAAAAGAAAAAAATAA
PfKH02         ATAAAAGAAAAAAATAA
PfGB4          ATAAAAGAAAAAAATAA
PfGA01_        -----------------
PfHB3_         ATAAAAGAAAAAAATAA
PfIT_          ATAAAAGAAAAAAATAA
```
