# Supplementary material for: Plasmodium falciparum surf4.1 in clinical isolates: From genetic variation and variant diversity to in silico design immunopeptides for vaccine development
Source: PLoS One. 2024 Dec 30;19(12):e0312091. doi: 10.1371/journal.pone.0312091 (PMC11684625; doi:10.1371/journal.pone.0312091)
Supplement: S2 File — (PDF) [file pone.0312091.s006.pdf]

```
                 10        20        30        40        50        60        70        80        90
         ....|....|....|....|....|....|....|....|....|....|....|....|....|....|....|....|....|....|
TAB130   MHFVVELDNTGDDFNDKAISTERFRNVFEVYVEDKIDELDTKPSEILNKECRHFNYFIDDMKDEFLTSSLIRLPKKLRKQLWESEVDENL
TAB131   MHFVVELDNTGDDFNDKAISTERFRNVFEVYVEDKIDELDTKPSEILNKECRHFNYFIDDMKDEFLTSSLIRLPKKLRKQLWESEVDENL
TAB151   MHFVVELDNTGDDFNDKAISTERFRNVFEVYVEDKIDELDTKPSEILNKECRHFNYFIDDMKDEFLTSSLIRLPKKLRKQLWESEVDENL
TAB152   MHFVVELDNTGDDFNDKAISIERFRNVFEVYVEDKIDELDTKPSEILNKECRHFNYFIDDMKDEFLTSSLIRLPKKLRKQLWESEVDENL
TAB138   MHFVVELDNTGDDFNDKAISTERFRNVFEVYVEDKIDELDTKPSEILNKECRHFNYFIDDMKDEFLTSSLIRLPKKLRKQLWESEVDENL
TAB123   MHFVVELDNTGDDFNDKAISTERFRNVFEVYVEDKIDELDTKPSEILNKECRHFNYFIDDMKDEFLTSSLIRLPKKLRKQLWESEVDENL
AA264    MHFVVELDNTGDDFNDKAISTERFRNVFEVYVEDKIDELDTKPSEILNKECRHFNYFIDDMKDEFLTSSLIRLPKKLRKQLWESEVDENL
SC52     MHFVVELDNTGDDFNDKAISTERFRNVFEVYVEDKIDELDTKPSEILNKECRHFNYFIDDMKDEFLTSSLIRLPKKLRKQLWESEVDENL
SC53     MHFVVELDNTGDDFNDKAISIERFRNVFEVYVEDKIDELDTKPSEILNKECRHFNYFIDDMKDEFLTSSLIRLPKKLRKQLWESEVDENL
SC03     MHFVVELDNTGDDFNDKAISIERFRNVFEVYVEDKIDELDTKPSEILNKECRHFNYFIDDMKDEFLTSSLIRLPKKLRKQLWESEVDKNL
AM1737   MHFVVELDNTGDDFNDKAISTERFRNVFEVYVEDKIDELDTKPSEILNKECRHFNYFIDDMKDEFLTSSLIRLPKKLRKQLWESEVDENL
AA235    MHFVVELDNTGDDFNDKAISIERFRNVFEVYVEDKIDELDTKPSEILNKECRHFNYFIDDMKDEFLTSSLIRLPKKLRKQLWESEVDENL
TAB141   MHFVVELDNTGDDFNDKAISIERFRNVFEVYVEDKIDELDTKPSEILNKECRHFNYFIDDMKDEFLTSSLIRLPKKLRKQLWESEVDENL
TAB166   MHFVVELDNTGDDFNDKAISTERFRNVFEVYVEDKIDELDTKPSEILNKECRHFNYFIDDMKDEFLTSSLIRLPKKLRKQLWESEVDENL
AA243    MHFVVELDNTGDDFNDKAISTERFRNVFEVYVEDKIDELDTKPSEILNKECRHFNYFIDDMKDEFLTSSLIRLPKKLRKQLWESEVDENL
AA258    MHFVVELDNTGDDFNDKAISTERFRNVFEVYVEDKIDELDTKPSEILNKECRHFNYFIDDMKDEFLTSSLIRLPKKLRKQLWESEVDENL
SC74     MHFVVELDNTGDDFNDKAISTERFRNVFEVYVEDKIDELDTKPSEILNKECRHFNYFIDDMKDEFLTSSLIRLPKKLRKQLWESEVDENL
AM1898   MHFVVELDNTGDDFNDKAISTERFRNVFEVYVEDKIDELDTKPSEILNKECRHFNYFIDDMKDEFLTSSLIRLSKELRKQLWESEVDEKL
SC55     MHFVVALDNTGDDFNDKAISTERFRNVFEVYVEDKIDELDTKPSEILNKECRHFNYFIDDMKDEFLTSSLIRLPKKLRKQLWESEVDENL
AM1880   MHFVVALDNTGDDFNDKAISTERFRNVFEVYVEDKIDELDTKPSEILNKECRHFNYFIDDMKDEFLTSSLIRLSKELRKQLWESEVDEKL
AM1802   MHFVVELDNTGDDFNDKAISTERFRNVFEVYVEDKIDELDTKPSEILNKECRHFNYFIDDMKDEFLTSSLIRLPKKLRKQLWESEVDENL
AM1803   MHFVVALDNTGDDFNDKAISTERFRNVFEVYVEDKIDELDTKPSEILNKECRHFNYFIDDMKDEFLKSSLIRLSKELRKQLWESEVDEKL
AM1811   MHFVVALDNTGDDFNDKAISTERFRNVFEVYVEDKIDELDTKPSEILNKECRHFNYFIDDMKDEFLKSSLIRLSKELRKQLWESEVDEKL
AM1814   MHFVVALDNTGDDFNDKAISTERFRNVFEVYVEDKIDELDTKPSEILNKECRHFNYFIDDMKDEFLTSSLIRLSKELRKQLWESEVDEKL
SC50     MHFVVALDNTGDDFNDKAISTERFRNVFEVYVEDKIDELDTKPSEILNKECRHFNYFIDDMKDEFLKSSLIRLSKELRKQLWESEVDEKL
SC56     MHFVVALDNTGDDFNDKAISTERFRNVFEVYVEDKIDELDTKPSEILNKECRHFNYFIDDMKDEFLKSSLIRLSKELRKQLWESEVDEKL
TAB153   MHFVVELDNTGDDFNDKAISTERFRNVFEVYVEDKIDELDTKPSEILNKECRHFNYFIDDMKDEFLTSSLIRLPKKLRKQLWESEVDENL
TAB154   MHFVVELDNTGDDFNDKAISTERFRNVFEVYVEDKIDELDTKPSEILNKECRHFNYFIDDMKDEFLTSSLIRLPKKLRKQLWESEVDENL
TAB156   MHFVVALDNTGDDFNDKAISTERFRNVFEVYVEDKIDELDTKPSEILNKECRHFNYFIDDMKDEFLKSSLIRLSKELRKQLWESEVDEKL
TAB136   MHFVVALDNTGDDFNDKAISTERFRNVFEVYVEDKIDELDTKPSEILNKECRHFNYFIDDMKDEFLTSSLIRLSKELRKQLWESEVDEKL
7G8      MHFVVELDNTGDDFNDKAISIERFRNVFEVYVEDKIDELDTKPSEILNKECRHFNYFIDDMKDEFLTSSLIRLPKKLRKQLWESEVDENL
IT       MHFVVALDNTGDDFNDKAISTERFRNVFEVYVEDKIDELDRKPSEILNKECRHFNYFIDDMKDEFLTSSLIRLPKELRKQLWESEVDEKL
HB3      MHFVVALDNTGDDFNDKAISTERFRNVFEVYVEDKIDELDTKPSEILNKECRHFNYFIDDMKDEFLKSSLIRLSKELRKQLWESEVDEKL
SD01     MHFVVALDNTGDDFNDKAISTERFRNVFEVYVEDKIDELDTKPSEILNKECRHFNYFIDDMKDEFLKSSLIRLSKELRKQLWESEVDEKL
FCR3     -HFVVELDNTGDDFNDKAISIERFRNVFEVYVEDKIDELDRKPSEILNKECRHFNYFIDDMKDEFLTSSLIRLPKELRKQLWESEVDEKL
CD01     MHFVVELDNTGDDFNDKAISTERFRNVFEVYVEDKIDELDTKPSEILNKECRHFNYFIDDMKDEFLTSSLIRLPKELRKQLWESEVDKNL
3D7      MHFVVELDNTGDDFNDKAISIERFRNVFEVYVEDKIDELDTKPSEILNKECRHFNYFIDDMKDEFLTSSLIRLPKKLRKQLWESEVDENL
MS822    -HFVVELDNTGDDFNDKAISIERFRNVFEVYVEDKIDELDTKPSEILNKECRHFNYFIDDMKDEFLTSSLIRLPKKLRKQLWESEVDENL
KH01     MHFVVELDNTGDDFNDKAISTERFRNVFEVYVEDKIDELDTKPSEILNKECRHFNYFIDDMKDEFLTSSLIRLPKKLRKQLWESEVDENL
Dd2      MHFVVELDNTGDDFNDKAISTERFRNVFEVYVEDKIDELDTKPSEILNKECRHFNYFIDDMKDEFLTSSLIRLPKKLRKQLWESEVDENL
GA01     MHFVVELDNTGDDFNDKAISTERFRNVFEVYVEDKIDELDTTPSEILNKECRHFNYFIDDMKDEFLTSSLIRLPKKLRKQLWESEVDKNL
SN01     MHFVVELDNTGDDFNDKAISTERFRNVFEVYVEDKIDELDTTPSEILNKECRHFNYFIDDMKDEFLTSSLIRLPKKLRKQLWESEVDENL
GN01     MHFVVELDNTGDDFNDKAISIERFRNVFEVYVEDKIDELDTKPSEILNKECRHFNYFIDDMKDEFLTSSLIRLPKKLRKQLWESEVDKNL
KE01     MHFVVELDNTGDDFNDKAISIERFRNVFEVYVEDKIDELDTKPSEILNKECRHFNYFIDDMKDEFLTSSLIRLPKKLRKQLWESEVDENL
KH02     MHFVVELDNTGDDFNDKAISIERFRNVFEVYVEDKIDELDTKPSEILNKECRHFNYFIDDMKDEFLTSSLIRLPKKLRKQLWESEVDKNL
GB4      MHFVVELDNTGDDFNDKAISIERFRNVFEVYVEDKIDELDTKPSEILNKECRHFNYFIDDMKDEFLTSSLIRLPKKLRKQLWESEVDENL
```

```
                 100       110       120       130       140       150       160       170       180
         ....|....|....|....|....|....|....|....|....|....|....|....|....|....|....|....|....|....|
TAB130   PNLMATTTHNKCLRTEHNYDKKYRDVIKILEDYCEDRATKLRDLKAIKYAEQDCINFNTWVSSWDYEIKRQMNKLDISKIKQYLEKSKFK
TAB131   PNLMATTTHNKCLRTEHNYDKKYRDVIKILEDYCEDRATKLRDLKAIKYAEQDCINFNTWVSSWDYEIKRQMNKLDISKIKQYLEKSKFK
TAB151   PNLMATTTHNKCLRTEHNYDKKYRDVIKILEDYCEDRATKLRDLKAIKYAEQDCINFNTWVSSWDYEIKRQMNKLDISKIKQYLEKSKFK
TAB152   PNLMATTTHNKCLRTEHNYDKKYRDVIKILEDYCEDRATKLRDLKAIKYAEQDCINFNTWVSSWDYEIKRQMNKLDISKIKQYLEKSKFK
TAB138   PNLMATTTHNKCLRTEHNYDKKYRDVIKILEDYCEDRATKLRDLKAIKYAEQDCINFNTWVSSWDYEIKRQMNKLDISKIKQYLEKSKFK
TAB123   PNLMATTTHNKCLRTEHNYDKKYRDVIKILEDYCEDRATKLRDLKAIKYAEQDCINFNTWVSSWDYEIKRQMNKLDISKIKQYLEKSKFK
AA264    PNLMATTTHNKCLRTEHNYDKKYRDVIKILEDYCEDRATKLRDLKAIKYAEQDCINFNTWVSSWDYEIKRQMNKLDISKIKQYLEKSKFK
SC52     PNLMATTTHNKCLRTEHNYDKKYRDVIKILEDYCEDRATKLRDLKAIKYAEQDCINFNTWVSSWDYEIKRQMNKLDISKIKQYLEKSKFK
SC53     PNLMATTTHNKCLRTEHNYDKKYRDVIKILEDYCEDRATKLRDLKAIKYAEQDCINFNTWVSSWDYEIKRQMNKLDISKIKQYLEKSKFK
SC03     PNLMATTTHNKCLRTEHNYDKKYRDVIKILEDYCEDRATKLRDLKAIKYAEQDCINFNTWVSSWDYEIKRQMNKLDISKIKQYLEKSKFK
AM1737   PNLMATTTHNKCLRTEHNYDKKYRDVIKILEDYCEDRATKLRDLKAIKYAEQDCINFNTWVSSWDYEIKRQMNKLDISKIKQYLEKSKFK
AA235    PNLMATTTHNKCLRTEHNYDKKYRDVIKILEDYCEDRATKLRDLKAIKYAEQDCINFNTWVSSWDYEIKRQMNKLDISKIKQYLEKSKFK
TAB141   PNLMATTTHNKCLRTEHNYDKKYRDVIKILEDYCEDRATKLRDLKAIKYAEQDCINFNTWVSSWDYEIKRQMNKLDISKIKQYLEKSKFK
TAB166   PNLMATTTHNKCLRTEHNYDKKYRDVIKILEDYCEDRATKLRDLKAIKYAEQDCINFNTWVSSWDYEIKRQMNKLDISKIKQYLEKSKFK
AA243    PNLMATTTHNKCLRTEHNYDKKYRDVIKILEDYCEDRATKLRDLKAIKYAEQDCINFNTWVSSWDYEIKRQMDKLDISKIKQYLEKSKFK
AA258    PNLMATTTHNKCLRTEHNYDKKYRDVIKILEDYCEDRATKLRDLKAIKYAEQDCINFNTWVSSWDYEIKRQMNKLDISKIKQYLEKSKFK
SC74     PNLMATTTHNKCLRTEHNYDKKYRDVIKILEDYCEDRATKLRDLKAIKYAEQDCINFNTWVSSWDYEIKRQMNKLDISKIKQYLEKSKFK
AM1898   PNLMATTTHNKCLRTEHNYDKKYRDVIKILEDYCEDRATKLRDLKAIKYAEQDCINFNTWVSSWDYEIKRQMNKLDISKIKQYLEKSKFK
SC55     PNLMATTTHNKCLRTEHNYDKKYRDVIKILEDYCEDRATKLRDLKAIKYAEQDCINFNTWVSSWDYEIKRQMNKLDISKIKQYLEKSKFK
AM1880   PNLMATTTHNKCLRTEHNYDKKYRDVIKILEDYCEDRATKLRDLKAIKYAEQDCINFNTWVSSWDYEIKRQMDKLDISKIKQYLEKSKFK
AM1802   PNLMATTTHNKCLRTEHNYDKKYRDVIKILEDYCEDRATKLRDLKAIKYAEQDCINFNTWVSSWDYEIKRQMNKLDISKIKQYLEKSKFK
AM1803   PNLMATTTHNKCLRTEHNYDKKYRDVIKILEDYCEDRATKLRDLKAIKYAEQDCINFNTWVSSWDYEIKRQMDKLDISKIKQYLEKSKFK
AM1811   PNLMATTTHNKCLRTEHNYDKKYRDVIKILEDYCEDRATKLRDLKAIKYAEQDCINFNTWVSSWDYEIKRQMDKLDISKIKQYLEKSKFK
AM1814   PNLMATTTHNKCLRTEHNYDKKYRDVIKILEDYCEDRATKLRDLKAIKYAEQDCINFNTWVSSWDYEIKRQMDKLDISKIKQYLEKSKFK
SC50     PNLMATTTHNKCLRTEHNYDKKYRDVIKILEDYCEDRATKLRDLKAIKYAEQDCINFNTWVSSWDYEIKRQMDKLDISKIKQYLEKSKFK
SC56     PNLMATTTHNKCLRTEHNYDKKYRDVIKILEDYCEDRATKLRDLKAIKYAEQDCINFNTWVSSWDYEIKRQMDKLDISKIKQYLEKSKFK
TAB153   PNLMATTTHNKCLRTEHNYDKKYRDVIKILEDYCEDRATKLRDLKAIKYAEQDCINFNTWVSSWDYEIKRQMDKLDISKIKQYLEKSKFK
TAB154   PNLMATTTHNKCLRTEHNYDKKYRDVIKILEDYCEDRATKLRDLKAIKYAEQDCINFNTWVSSWDYEIKRQMNKLDISKIKQYLEKSKFK
TAB156   PNLMATTTHNKCLRTEHNYDKKYRDVIKILEDYCEDRATKLRDLKAIKYAEQDCINFNTWVSSWDYEIKRQMDKLDISKIKQYLEKSKFK
TAB136   PNLMATTTHNKCLRTEHNYDKKYRDVIKILEDYCEDRATKLRDLKAIKYAEQDCINFNTWVSSWDYEIKRQMDKLDISKIKQYLEKSKFK
7G8      PNLMATTTHNKCLRTEHNYDKKYRDVIKILEDYCEDRATKLRDLKAIKYAEQDCINFNTWVSSWDYEIKRQMDKLDISKIKQYLEKSKFK
IT       PNLMATTTHNKCLRTEHNYDKKYRDVIKILEDYCEDRATKLRDLKAIKYAEQDCINFNTWVSSWDYEIKRQMNKLDISKIKQYLEKSKFK
HB3      PNLMATTTHNKCLRTEHNYDKKYRDVIKILEDYCEDRATKLRDLKAIKYAEQDCINFNTWVSSWDYEIKRQMDKLDISKIKQYLEKSKFK
SD01     PNLMATTTHNKCLRTEHNYDKKYRDVIKILEDYCEDRATKLRDLKAIKYAEQDCINFNTWVSSWDYEIKRQMNKLDISKIKQYLEKSKFK
FCR3     PNLMATTTHNKCLRTEHNYDKKYRDVIKILEDYCEDRATKLRDLKAIKYAEQDCINFNTWVSSWDYEIKRQMNKLDISKIKQYLEKSKFK
CD01     PNLMATTTHNKCLRTEHNYDKKYRDVIKILEDYCEDRATKLRDLKAIKYAEQDCINFNTWVSSWDYEIKRQMNKLDISKIKQYLEKSKFK
3D7      PNLMATTTHNKCLRTEHNYDKKYRDVIKILEDYCEDRATKLRDLKAIKYAEQDCINFNTWVSSWDYEIKRQMNKLDISKIKQYLEKSKFK
MS822    PNLMATTTHNKCLRTEHNYDKKYRDVIKILEDYCEDRATKLRDLKAIKYAEQDCINFNTWVSSWDYEIKRQMNKLDISKIKQYLEKSKFK
KH01     PNLMATTTHNKCLRTEHNYDKKYRDVIKILEDYCEDRATKLRDLKAIKYAEQDCINFNTWVSSWDYEIKRQMNKLDISKIKQYLEKSKFK
Dd2      PNLMATTTHNKCLRTEHNYDKKYRDVIKILEDYCEDRATKLRDLKAIKYAEQDCINFNTWVSSWDYEIKRQMNKLDISKIKQYLEKSKFK
GA01     PNLMATTTHNKCLRTEHNYDKKYRDVIKILEDYCEDRATKLRDLKAIKYAEQDCINFNTWVSSWDYEIKRQMNKLDISKIKQYLEKSKFK
SN01     PNLMATTTHNKCLRTEHNYDKKYRDVIKILEDYCEDRATKLRDLKAIKYAEQDCINFNTWVSSWDYEIKRQMDKLDISKIKQYLEKSKFK
GN01     PNLMATTTHNKCLRTEHNYDKKYRDVIKILEDYCEDRATKLRDLKAIKYAEQDCINFNTWVSSWDYEIKRQMNKLDISKIKQYLEKSKFK
KE01     PNLMATTTHNKCLRTEHNYDKKYRDVIKILEDYCEDRATKLRDLKAIKYAEQDCINFNTWVSSWDYEIKRQMNKLDISKIKQYLEKSKFK
KH02     PNLMATTTHNKCLRTEHNYDKKYRDVIKILEDYCEDRATKLRDLKAIKYAEQDCINFNTWVSSWDYEIKRQMNKLDISKIKQYLEKSKFK
GB4      PNLMATTTHNKCLRTEHNYDKKYRDVIKILEDYCEDRATKLRDLKAIKYAEQDCINFNTWVSSWDYEIKRQMNKLDISKIKQYLEKSKFK
```

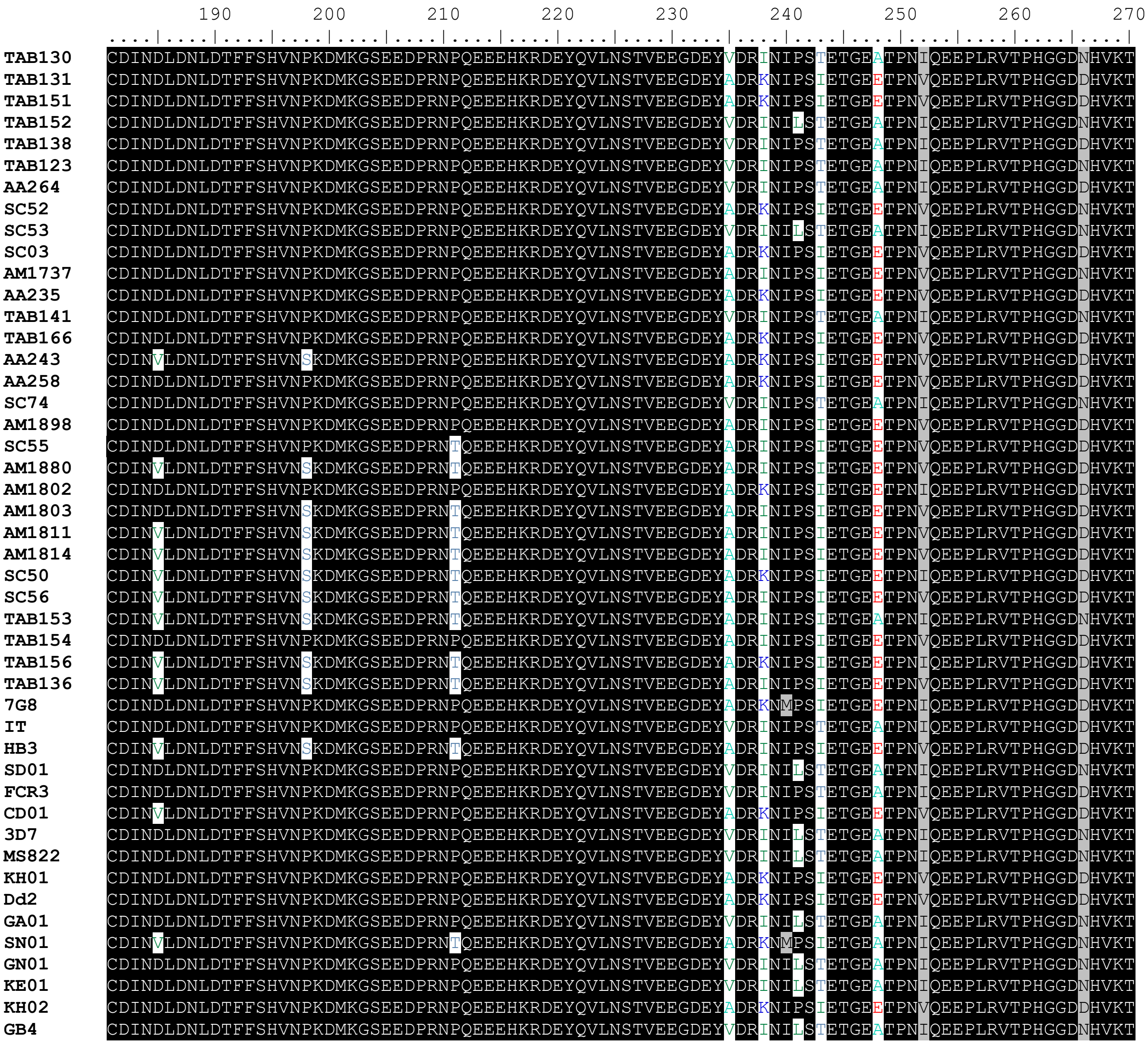
190 200 210 220 230 240 250 260 270
TAB130
TAB131
TAB151
TAB152
TAB138
TAB123
AA264
SC52
SC53
SC03
AM1737
AA235
TAB141
TAB166
AA243
AA258
SC74
AM1898
SC55
AM1880
AM1802
AM1803
AM1811
AM1814
SC50
SC56
TAB153
TAB154
TAB156
TAB136
7G8
IT
HB3
SD01
FCR3
CD01
3D7
MS822
KH01
Dd2
GA01
SN01
GN01
KE01
KH02
GB4

```
                     280       290       300       310       320       330       340       350       360
           ....|....|....|....|....|....|....|....|....|....|....|....|....|....|....|....|....|....|
TAB130     PPTIVKTEVGNDNRENVVSTAGKRLNRGKANKDGVPHTTKFKTPKVRAPAEGKKSKNARDSTEPKHQKEKRPYDRIKKTSEQINKYAHPD
TAB131     SPTIVKTEVGNDNRENVVSTAGKRLNRGKANKDGVPHTTKFKTPKVRAPAEGKKSKNARDSTEPKHQKEKRPYDRIKKTREQINKYAHPD
TAB151     SPTIVKTEVGNDNRENVVSTAGKRLNRGKANKDGVPHTTKFKTPKVRAPAEGKKSKNARDSTEPKHQKEKRPYDRIKKTREQINKYAHPD
TAB152     PPTIVKTEVGNDNRENVVSTAGKRLNRGKPNKDGVPHTTKFKTPKVRAPAEGKKSKNARDSTEPKHQKEKRPYDRIKKTSEQINKYAHPD
TAB138     PPTIVKTEEGNDNRENVVSTAGKRLNRGKPNKEGVPHTTKFKTPKVRAPAEGKKSKNARDSTEPKHQKEKRPYDRIKKTSEQINKYAHPD
TAB123     PPTIVKTEVGNGNRENVVSTAGKRLNRGKANKDGVPHTTKFKTPKVRAPAEGKKSKNARDSTEPKHQKEKRPYDRIKKTREQINKYAHPD
AA264      PPTIVKTEEGNDNRENVVSTAGKRLNRGKPNKEGGPHTTKFKTPKVSAPAGGKKSKNARDSTEPKHQKEKRPYDRIKKTSEQINKYAHPD
SC52       PPTIVKTEVGNDNRENVVSTAGKRLNRGKANKDGVPHTTKFKTPKVRAPAEGKKSKNARDSTEPKHQKEKRPYDRIKKTSEQINKYAHPN
SC53       PPTIVKTEVGNDNRENVVSTAGKRLNRGKPNKDGVPHTTKFKTPKVRAPAEGKKSKNARDSTEPKHQKEKRPYDRIKKTSEQINKYAHPD
SC03       PPTIVKTEVGNDNRENVVSTAGKRLNRGKANKDGVPHTTKFKTPKVRAPAEGKKSKNARDSTEPKHQKEKRPYDRIKKTSEQINKYAHPD
AM1737     PPTIVKTEVGNDNRENVVSTAGKRLNRGKANKDGVPHTTKFKTPKVRAPAEGKKSKNARDSTEPKHQKEKRPYDRIKKTSEQINKYAHPN
AA235      PPTIVKTEVGNDNRENVVSTAGKRLNRGKANKDGVPHTTKFKTPKVRAPAEGKKSKNARDSTEPKHQKEKRPYDRIKKTSEQINKYAHPD
TAB141     PPTIVKTEVGNDNRENVVSTAGKRLNRGKANKDGVPHTTKFKTPKVRAPAEGKKSKNARDSTEPKHQKEKRPYDRIKKTSEQINKYAHPD
TAB166     PPTIVKTEVGNDNRENVVSTAGKRLNRGKANKDGVPHTTKFKTPKVRAPAEGKKSKNARDSTEPKHQKEKRPYDRIKKTSEQINKYAHPD
AA243      PPTIVKTEVGNDNRENVVSTAGKRLNRGKANKDGVPHTTKFKTPKVRAPAEGKKSKNARDSTEPKHQKEKRPYDRIKKTSEQINKYAHPD
AA258      PPTIVKTEVGNDNRENVVSTAGKRLNRGKANKDGVPHTTKFKTPKVRAPAEGKKSKNARDSTEPKHQKEKRPYDRIKKTSEQINKYAHPD
SC74       PPTIVKTEVGNDNRENVVSTAGKRLNRGKANKDGVPHTTKFKTPKVRAPAEGKKSKNARDSTEPKHQKEKRPYDRIKKTSEQINKYAHPD
AM1898     PPTIVKTEVGNDNRENVVSTAGKRLNKGKVNKDGVPHTTKFKTPKVSASAEGKKSKNARDSTEPKHQKEKRPYDRIKKTSEQINKYAHPN
SC55       PPTIVKTEVGNDNRENVVSTAGKRLNRGKANKDGVPHTTKFKTPKVSASAEGKKSKNARDSTEPKHQKEKRPYDRIKKTSEQINKYAHPN
AM1880     PPTIVKTEVGNDNRENVVSTAGKRLNKGKLNNEGGQHTTKFKTPKVSASVGGKKSKNLRGNTEPKHQKEKTSYDRTRKTSEQINKYAHPN
AM1802     PPTIVKTEEGNDNRENVVSTAGKRLNRGKPNKEGGPHTTKFKTPKVSAPAGGKKSKNLRGNTEPKHQKAKTSYDRTRKTSEQINKYAHPN
AM1803     SPIIVKTEEGNDNRENVVSTAGKRLNGGKLNKEGGPHTTKFRTPKVRAPAEGKKSKNARDSTEPKHQKEKRPYDRIKKTSEQINKYAHPD
AM1811     SPIIVKTEEGNDNRENVVSTAGKRLNGGKLNKEGGPHTTKFRTPKVRAPAEGKKSKNARDSTEPKHQKEKRPYDRIKKTSEQINKYAHPD
AM1814     PPTIVKTEVGNDNRENVVSTAGKRLNRGKPNKEGGPHTTKFKTPKVSAPAGGKKSKNLRDNTEPKHQKEKRPYDRTRKTSEQINKYAHPN
SC50       PPTIVKTEEGNDNRENVVSTAGKRLNRGKPNKEGGPHTTKFKTPKVSAPAGGKKSKNLRGNTEPKHQKAKTSYDRTRKTSEQINKYAHPN
SC56       SPIIVKTEEGNDNRENVVSTAGKRLNGGKLNKEGGPHTTKFRTPKVRAPAEGKKSKNARDSTEPKHQKEKRPYDRIKKTSEQINKYAHPD
TAB153     PPTIVKTEVGNDNRENVVSTAGKRLNRGKANKDGVPHTTKFKTPKVRAPAEGKKSKNARDSTEPKHQKEKRPYDRIKKTSEQINKYAHPN
TAB154     PPTIVKTEVGNDNRENVVSTAGKRLNRGKANKDGVPHTTKFKTPKVRAPAEGKKSKNARDSTEPKHQKEKRPYDRIKKTSEQINKYAHPN
TAB156     PPTIVKTEEGNDNRENVVSTAGKRLNRGKPNKEGGPHTTKFKTPKVSAPAGGKKSKNLRGNTEPKHQKAKTSYDRTRKTSEQINKYAHPN
TAB136     PPTIVKTEVGNDNRENVVSTAGKRLNKGKLNNEGGQHTTKFKTPKVSASVGGKKSKNLRGNTEPKHQKEKTSYDRTRKTSEQINKYAHPN
7G8        PPTIVKTEVGNDNRENVVSTAGKRLNKGKLNKEGGQHTTKFKTPKVSASVGGKKSKNLRGNTEPKHQKEKTSYDRTRKTSEQINKYAHPN
IT         PPTIVKTEEGNDNRENVVSTAGKRLNRGKPNKEGGPHTSRFKTPKVSAPAGGKKSKNARDSTEPKHHKEKGKYDRIKKTSEQINKYAHPD
HB3        PPTIVKTEVGNDNRENVVSTAGKRLNKGKLNKEGGQHTTKFKTPKVSASVGGKKSKNLRGNTEPKHQKEKTSYDRTRKTSEQINKYAHPN
SD01       PPTIVKTEVGNDNRENVVSTAGKRLNRGKANKDGVPHTSRFKTPKVSAPAGGKKSKNARDSTEPKHHKEKGKYDRIKKTSEQINKYAHPN
FCR3       PPTIVKTEEGNDNRENVVSTAGKRLNRGKPNKEGGPHTSRFKTPKVSAPAGGKKSKNARDSTEPKHHKEKGKYDRIKKTSEQINKYAHPD
CD01       SPIIVKTEEGNDNRENVVSTAGKRLNGGKLNKEGGQHTTKFKTPKVSASVGGKKSKNLRGNTEPKHQKEKTSYDRTRKTSEQINKYAHPN
3D7        PPTIVKTEVGNDNRENVVSTAGKRLNRGKPNKDGVPHTSRFKTPKVSAPAGGKKSKNARDSTEPKHHKEKGKYDRIKKTSEQINKYAHPN
MS822      PPTIVKTEVGNDNRENVVSTAGKRLNRGKPNKDGVPHTSRFKTPKVSAPAGGKKSKNARDSTEPKHHKEKGKYDRIKKTSEQINKYAHPN
KH01       SPTIVKTEVGNDNRENVVSTAGKRLNRGKANKDGVPHTTKFKTPKVRAPAEGKKSKNARDSTEPKHQKEKRPYDRIKKTREQINKYAHPD
Dd2        PPTIVKTEVGNDNRENVVSTAGKRLNRGKANKDGVPHTTKFKTPKVRAPAEGKKSKNARDSTEPKHHKEKGKYDRIKKTSEQINKYAHPN
GA01       PPTIVKTEVGNDNRENVVSTAGKRLNRGKANKDGVPHTTKFKTPKVRAPAEGKKSKNARDSTEPKHQKEKRPYDRIKKTREQINKYAHPN
SN01       PPTIVKTEVGNDNRENVVSTAGKRLNRGKANKDGVPHTTKFKTPKVRAPAEGKKSKNARDNTEPKHQKEKRPYDRIKKTREQINKYAHPN
GN01       PPTIVKTEVGNDNRENVVSTAGKRLNRGKPNKDGVPHTTKFKTPKVRAPAEGKKSKNARDNTEPKHQKEKRPYDRIKKTREQINKYAHPN
KE01       PPTIVKTEVGNDNRENVVSTAGKRLNRGKPNKDGVPHTTKFKTPKVRAPAEGKKSKNARDSTEPKHQKEKRPYDRIKKTREQINKYAHPD
KH02       PPTIVKTEVGNDNRENVVSTAGKRLNRGKANKDGVPHTTKFKTPKVRAPAEGKKSKNARDSTEPKHQKEKRPYDRIKKTREQINKYAHPD
GB4        PPTIVKTEVGNDNRENVVSTAGKRLNRGKPNKDGVPHTTKFKTPKVRAPAEGKKSKNARDSTEPKHQKEKRPYDRIKKTREQINKYAHPD
```

	370 380 390 400 410 420 430 440 450
TAB130	NKYTIFQCTDLDCKWLKPKHIKYPYEGYDIVDNDKLVDTGKGDYEDHHTYCSGDECAFGNAVEGQFDESRSQGKQRRKKGKGKGRKKGH-
TAB131	NKYTIFQCTDLDCKWLKPKHIKYPYEGYDIVDNDKLVDTGKGDYEDHHTYCSGDECAFGNAVEGQFDESRSQGKQRRKKGKGKGRKKGH-
TAB151	NKYTIFQCTDLDCKWLKPKHIKYPYEGYDIVDNDKLVDTGKGDYEDHHTYCSGDECAFGNAVEGQFDESRSQGKQRRKKGKGKGRKKGH-
TAB152	NKYTIFQCTDLDCKWLKPKHIKYPYEGYDIVDNDKLVDTGKGDYEDHHTYCSGDECAFGNAVEGQFDESRSQGKQRRKKGKGKGRKKGH-
TAB138	NKYTIFQCTDLDCKWLKPKHIKYPYEGYDIVDNDKLVDTGKGDYEDHHTYCSGDECAFGNAVEGQFDESRSQGKQRRKKGKGKGRKKGH-
TAB123	NKYTIFQCTDLDCKWLKPKHIKYPYEGYDIVDNDKLVDTGKGDYEDHHTYCSGDECAFGNAVEGQFDESRSQGKQRRKKGKGKGRKKGH-
AA264	NKYTIFQCTDLDCKWLKPKHIKYPYEGYDIVDNDKLVDTGKGDYEDHHTYCSGDECAFGNAVEGQFDESRSQGKQRRKKGKGKGRKKGH-
SC52	NKYTIFQCTDLDCKWLKPKHIKYPYEGYDIVDNDKLVDTGKGDYEDHHTYCSGDECAFGNAVGGQFDESRSQGKQRRKKGKGKGRKKGHN
SC53	NKYTIFQCTDLDCKWLKPKHIKYPYEGYDIVDNDKLVDTGKGDYEDHHTYCSGDECAFGNAVEGQFDESRSQGKQRRKKGKGKGRKKGH-
SC03	NKYTIFQCTDLDCKWLKPKHIKYPYEGYDIVDNDKLVDTGKGDYEDHHTYCSGDECAFGNAVEGQFDESRSQGKQRRKKGKGKGRKKGHN
AM1737	NKYTIFQCTDLDCKRLKPKHIKYPYEGYDIVDNDKLVDTGKGDYEDHHTYCSGDECAFGNAVEGQFDESRSQGKQRRKKGKGKGRKKGH-
AA235	NKYTIFQCTDLDCKWLKPKHIKYPYEGYDIVDNDKLVDTGKGDYEDHHTYCSGDECAFGNAVEGQFDESRSQGKQRRKKGKGKGRKKGHN
TAB141	NKYTIFQCTDLDCKWLKPKHIKYPYEGYDIVDNDKLVDTGKGDYEDHHTYCSGDECAFGNAVEGQFDESRSQGKQRRKKGKGKGRKKGH-
TAB166	NKYTIFQCTDLDCKWLKPKHIKYPYEGYDIVDNDKLVDTGKGDYEDHHTYCSGDECAFGNAVEGQFDESRSQGKQRRKKGKGKGRKKGHN
AA243	NKYTIFQCTDLDCKWLKPKHIKYPYEGYDIVDNDKLVDTGKGDYEDHHTYCSGDECAFGNAVEGQFDESRSQGKQRRKKGKGKGRKKGHN
AA258	NKYTIFQCTDLDCKWLKPKHIKYPYEGYDIVDNDKLVDTGKGDYEDHHTYCSGDECAFGNAVEGQFDESRSQGKQRRKKGKGKGRKKGHN
SC74	NKYTIFQCTDLDCKWLKPKHIKYPYEGYDIVDNDKLVDTGKGDYEDHHTYCSGDECAFGNAVEGQFDESRSQGKQRRKKGKGKGRKKGH-
AM1898	NKYTIFQCTDLDCKWLKPKHIKYPYEGYDIVDNDKLVDTGKGVYEDHHTYCSGDECAFGNAVEGQFDESRSQGKQRRKKGKGKGRKKGH-
SC55	NKYTIFQCTDLDCKWLKPKHIKYPYEGYDIVDNDKLVDTGKGDYEDHHTYCSGDECAFGNAVEGQFDESRSQGKQRRKKGKGKGRKKGHN
AM1880	NKYTIFQCTDLDCKWLKPKNIKYTYEGYDIVDNDKLVDTGKGVYEDYNKYCSGDECALGNAVEGQFDEIGSEGNLKRKKGKGKGRKKDH-
AM1802	NKYTIFQCTDLDCKWLKPKNIKYPYESYDIVDNDKFVDTGKGVYEDHHTYCSGDECALGNAVEGQFDESRSQGKKRRKKVKGEGRKKGHN
AM1803	NKYTIFQCTDLDCKCLKPKNIKYPYEGYDIVDNDKLVDTGKGDYEDHHTYCSGDECAFGNAVEGQFDESRSQGKQRRKKGKGKGRKKGHN
AM1811	NKYTIFQCTDLDCKCLKPKNIKYPYEGYDIVDNDKLVDTGKGDYEDHHTYCSGDECAFGNAVEGQFDESRSQGKQRRKKGKGKGRKKGH-
AM1814	NKYTIFQCTDLDCKWLKPKNIKYPYEGYDIVDNDKLVDTGKGVYEDHHTYCSGDECALGNAVEGQFDESRSQGKQRRKKGKGKGRKKGHN
SC50	NKYTIFQCTDLDCKWLKPKNIKYPYESYDIVDNDKFVDTGKGVYEDHHTYCSGDECALGNAVEGQFDESRSQGKKRRKKVKGEGRKKGHN
SC56	NKYTIFQCTDLDCKCLKPKNIKYPYEGYDIVDNDKLVDTGKGDYEDHHTYCSGDECAFGNAVEGQFDESRSQGKQRRKKGKGKGRKKGH-
TAB153	NKYTIFQCTDLDCKRLKPKHIKYPYEGYDIVDNDKLVDTGKGDYEDHHTYCSGDECAFGNAVEGQFDESRSQGKQRRKKGKGKGRKKGHN
TAB154	NKYTIFQCTDLDCKWLKPKHIKYPYEGYDIVDNDKLVDTGKGDYEDHHTYCSGDECAFGNAVEGQFDESRSQGKQRRKKGKGKGRKKGHN
TAB156	NKYTIFQCTDLDCKWLKPKNIKYPYESYDIVDNDKFVDTGKGVYEDHHTYCSGDECALGNAVEGQFDESRSQGKKRRKKVKGEGRKKGHN
TAB136	NKYTIFQCTDLDCKWLKPKNIKYTYEGYDIVDNDKLVDTGKGVYEDYNKYCSGDECALGNAVEGQFDEIGSEGNLKRKKGKGKGRKKDH-
7G8	NKYTIFQCTDLDCKWLKPKNIKYTYEGHDIVDNDKLVDTGKGVYEDYNKYCSGDECALGNAVEGQFDEIGSEGNLKRKKGKGKGRKKDH-
IT	NKYTIFQCTDLDCKWLKPKNIKYPYEGYDIVDNDKLVDTGKGDYEDHHTYCSGDECAFGNAVGGQFDEIGSEGNLKRKKGKGKGRKKGHN
HB3	NKYTIFQCTDLDCKWLKPKNIKYTYEGYDIVDNDKLVDTGKGVYEDYNKYCSGDECALGNAVEGQFDEIGSEGNLKRKKGKGKGRKKDH-
SD01	NKYTIFQCTDLDCKWLKPKHIKYPYEGYDIVDNDKLVDTGKGDYEDHHTYCSGDECAFGNAVEGQFDESRSQGKQRRKKGKGKGRKKGH-
FCR3	NKYTIFQCTDLDCKWLKPKNIKYPYEGYDIVDNDKLVDTGKGDYEDHHTYCSGDECAFGNAVGGQFDEIGSEGNLKRKKGKGKGRKKGHN
CD01	NKYTIFQCTDLDCKWLKPKNIKYTYEGHDIVDNDKLVDTGKGVYEDYNKYCSGDECALGNAVEGQFDEIGSEGNLKRKKGKGKGRKKDH-
3D7	NKYTIFQCTDLDCKWLKPKHIKYPYEGYDIVDNDKLVDTGKGDYEDHHTYCSGDECAFGNAVGGQFDESRSQGKQRRKKGKGKGRKKGHN
MS822	NKYTIFQCTDLDCKWLKPKHIKYPYEGYDIVDNDKLVDTGKGDYEDHHTYCSGDECAFGNAVGGQFDESRSQGKQRRKKGKGKGRKKGHN
KH01	NKYTIFQCTDLDCKWLKPKHIKYPYEGYDIVDNDKLVDTGKGDYEDHHTYCSGDECAFGNAVEGQFDESRSQGKQRRKKGKGKGRKKGH-
Dd2	NKYTIFQCTDLDCKWLKPKHIKYPYEGYDIVDNDKLVDTGKGDYEDHHTYCSGDECAFGNAVEGQFDESRSQGKQRRKKGKGKGRKKGH-
GA01	NKYTIFQCTDLDCKWLKPKHIKYPYEGYDIVDNDKLVDTGKGDYEDHHTYCSGDECAFGNAVEGQFDESRSQGKQRRKKGKGKGRKKGHN
SN01	NKYTIFQCTDLDCKWLKPKHIKYPYEGYDIVDNDKLVDTGKGDYEDHHTYCSGDECAFGNAVEGQFDESRSQGKQRRKKGKGKGRKKGHN
GN01	NKYTIFQCTDLDCKWLKPKHIKYPYEGYDIVDNDKLVDTGKGDYEDHHTYCSGDECAFGNAVEGQFDESRSQGKQRRKKGKGKGRKKGHN
KE01	NKYTIFQCTDLDCKWLKPKHIKYPYEGYDIVDNDKLVDTGKGDYEDHHTYCSGDECAFGNAVEGQFDESRSQGKQRRKKGKGKGRKKGHN
KH02	NKYTIFQCTDLDCKWLKPKHIKYPYEGYDIVDNDKLVDTGKGDYEDHHTYCSGDECAFGNAVGGQFDEIGSEGNLKRKKGKGKGRKKGHN
GB4	NKYTIFQCTDLDCKWLKPKHIKYPYEGYDIVDNDKLVDTGKGDYEDHHTYCSGDECAFGNAVGGQFDESRSQGKQRRKKGKGKGRKKGHN

```
              460       470       480       490       500       510       520       530       540
     ....|....|....|....|....|....|....|....|....|....|....|....|....|....|....|....|....|....|
TAB130  KSVGKSLPNGEGTHSYITNGGNREETKSQSYSVTDNYVEEEAASCPEGDQNCIDLIKDEFIIEGGRFIRTSINDVPQMEAITNKYMPGSI
TAB131  KSVGKSLPNGEGTHSYITNGGNREETKSQSYSVTDNYVEEEAASCPEGDQDCIDLIKDEFIIEGGRFIRTSINDVPQMEAITNKYMPGSI
TAB151  KSVGKSLPNGEGTHSYITNGGNREETKSQSYSVTDNYVEEEAASCPEGDQDCIDLIKDEFIIEGGRFIRTSINDVPQMEAITNKYMPGSI
TAB152  KSVGKSLPNGEGTHSYITNGGNREETKSQSYSVTDNYVEEEAASCPEGDQDCIDLIKDEFIIEGGRFIRTSINDVPQMEAITNKYMPGSI
TAB138  KSVGKSLPNGEGTHSYITNGGNREETKSQSYSVTDNYVEEEAASCPEGDQDCIDLIKDEFIIEGGRVIRTSINDVPQMEAITNKYMPGSI
TAB123  KSVGKSLPNGEGTHSYITNGGNREETKSQSYSVTDNYVEEEAASCPEGDQDCIDLIKDEFIIEGGRVIRTSINDVPQMEAITNKYMPGSI
AA264   KSVGKSLPNGEGTHSYITNGGNREETKSQSYSVTDNYVEEEAASCPEGDQDCIDLIKDEFIIEGGRFIRTSINDVPQMEAITNKYMPGSI
SC52    KLEGKSSPNVEGTHSYITNGGNREETKSQSYSVTDNYVEEEAASCPEGDEDCIDLIKDEFIIEGGRVIRTSINDVPQMEAITNKYMPGSI
SC53    KSVGKSLPNGEGTHSYITNGGNREETKSQSYSVTDNYVEEEAASCPEGDQDCIDLIKDEFIIEGGRFIRTSINDVPQMEAITNKYMPGSI
SC03    KLEGKSSPNVEGTHSYITNGGNIEETKSQSYSVTDNYVEEEAASCPEGDQNCIDLIKDEFIIEGGRVIRTSINDVPQMEAITNKYMPGSI
AM1737  KSVGKSLPNGEGTHSYITNGGNREETKSQSYSVTDNYVEEEAASCPEGDQDCIDLIKDEFIIEGGRFIRTSINDVPQMEAITNKYMPGSI
AA235   KLEGKSSPNGEGTHSYITNGGNREETKSQSYSVTDNYVEEEAASCPEGDQDCIDLIKDEFIIEGGRVIRTSINDVPQMEAITNKYMPGSI
TAB141  KSVGKSLPNGEGTHSYITNGGNREETKSQSYSVTDNYVEEEAASCPEGDQNCIDLIKDEFIIEGGRVIRTSINDVPQMEAITNKYMPGSI
TAB166  KLEGKSSPNVEGTHSYITNGGNIEETKSQSYSVTDNYVEEEAASCPEGDQNCIDLIKDEFIIEGGRFIRTSINDVPQMEAITNKYMPGSI
AA243   KLEGKSSPNVEGTHSYITNGGNIEETKSQSYSVTDNYVEEEAASCPEGDQDCIDLIKDEFIIEGGRFIRTSINDVPQMEAITNKYMPGSI
AA258   KLEGKSSPNVEGTHSYITNGGNIEETKSQSYSVTDNYVEEEAASCPEGDQNCIDLIKDEFIIEGGRVIRTSINDVPQMEAITNKYMPGSI
SC74    KSVGKSLPNGEGTHSYITNGGNREETKSQSYSVTDNYVEEEAASCPEGDQNCIDLIKDEFIIEGGRFIRTSINDVPQMEAITNKYMPGSI
AM1898  KSVGKSLPNGEGTHSYMRHGDNKEETKSQSHSVTDNYVEEEAASCPEGDQDCIDLIKDEFIIEGGRFIRTSINDVPQMEAITNKYMPGSI
SC55    KSVGKSLPNGEGTHSYMRHGDNKEETKSQSHSVTDNYVEEEAASCPEGDQNCIDLIKDEFIIEGGRFIRTSINDVPQMEAITNKYMPGSI
AM1880  KSVGKSLPNGEGTHSYMRHGDNKEETKSQSHSVTDHDVEEEAVSCPEGEQNCIDLIKDEFIIEGGRFIRTSINDVPQMEAITNKYMPGSI
AM1802  KLEGKSSPNGEGTHSYMRHGDNKEETKSQSHSVSEISAEEEAASCPEGDQGCIDPTKQTFVIEGNTVRSMEIGDVPKMEAVTSIDLPKAI
AM1803  KSVGKSLPNGEGTHSYITNGGNREETKSQSYSVTDNYVEEEAASCPEGDQDCIDLIKDEFIIEGGRVIRTSINDVPQMEAITNKYMPGSI
AM1811  KSVGKSLPNGEGTHSYITNGGNREETKSQSYSVTDNYVEEEAASCPEGDQDCIDLIKDEFIIEGGRVIRTSINDVPQMEAITNKYMPGSI
AM1814  KLEGKSSPNGEGTHSYMRHGDNKEETKSQSHSVTDHDVEEEAASCPEGDQDCIDLIKDEFIIEGGRFIRTSINDVPQMEAITNKYMPGSI
SC50    KLEGKSSPNGEGTHSYMRHGDNKEETKSQSHSVSEISAEEEAASCPEGDQGCIDPTKQTFVIEGNTVRSMEIGDVPKMEAVTSIDLPKAI
SC56    KSVGKSLPNGEGTHSYITNGGNREETKSQSYSVTDNYVEEEAASCPEGDQDCIDLIKDEFIIEGGRVIRTSINDVPQMEAITNKYMPGSI
TAB153  KLEGKSSPNVEGTHSYITNGGNREETKSQSYSVTDNYVEEEAASCPEGDQDCIDLIKDEFIIEGGRVIRTSINDVPQMEAITNKYMPGSI
TAB154  KLEGKSSPNVEGTHSYITNGGNREETISQSYSVTDNYVEEEAASCPEGDQDCIDLIKDEFIIEGGRFIRTSINDVPQMEAITNKYMPGSI
TAB156  KLEGKSSPNGEGTHSYMRHGDNKEETKSQSHSVSEISAEEEAASCPEGDQGCIDPTKQTFVIEGNTVRSMEIGDVPKMEAVTSIDLPKAI
TAB136  KSVGKSLPNGEGTHSYMRHGDNKEETKSQSHSVTDHDVEEEAVSCPEGEENCIDPIKDEFIIEGGSFIRTSINDVPQMEAITSIDSPKGI
7G8     KSVGKSLPNGEGTHSYMRHGDNKEETKSQSHSVSEISAEEEAASCPEGDQSCIDPTKQTFVIEGNTVRSMEIGDVPKMETVTSIDLPKAI
IT      KLEGKSSPNVEGTHSYITNGGNIEETKSQSYSVTDNYVEEEAASCPEGDQDCIDPIKDEFIIEGGRVIRTSINDVPQMEAITNKYMPGSI
HB3     KSVGKSLPNGEGTHSYMRHGDNKEETKSQSHSVTDHDVEEEAVSCPEGEENCIDPTKQIFVIEGDHVRSISINNIPKMEAVTSIDSPKGI
SD01    KSVGKSLPNGEGTHSYITNGGNREETKSQSYSVTDNYVEEEAASCPEGDQDCIDLIKDEFIIEGGRVIRTSINDVPQMEAITNKYMPGSI
FCR3    KLEGKSSPNVEGTHSYITNGGNIEETKSQSYSVTDNYVEEEAASCPEGDQDCIDPIKDEFIIEGGRVIRTSINDVPQMEAITNKYMPGSI
CD01    KSVGKSLPNGEGTHSYMRHGDNKEETKSQSHSVSEISAEEEAASCPEGDQSCIDPTKQTFVIEGNTVRSMEIGDVPKMETVTSIDLPKAI
3D7     KLEGKSSPNVEGTHSYITNGGNIEETKSQSYSVTDNYVEEEAASCPEGDQNCIDLIKDEFIIEGGRFIRTSINDVPQMEAITNKYMPGSI
MS822   KLEGKSSPNVEGTHSYITNGGNIEETKSQSYSVTDNYVEEEAASCPEGDQNCIDLIKDEFIIEGGRFIRTSINDVPQMEAITNKYMPGSI
KH01    KSVGKSLPNGEGTHSYITNGGNREETKSQSYSVTDNYVEEEAASCPEGDQDCIDLIKDEFIIEGGRFIRTSINDVPQMEAITNKYMPGSI
Dd2     KSVGKSLPNGEGTHSYITNGGNREETKSQSYSVTDNYVEEEAASCPEGDQDCIDLIKDEFIIEGGRFIRTSINDVPQMEAITNKYMPGSI
GA01    KLEGKSSPNVEGTHSYITNGGNREETKSQSYSVTDNYVEEEAASCPEGDQDCIDLIKDEFIIEGGRFIRTSINDVPQMEAITNKYMPGSI
SN01    KLEGKSSPNVEGTHSYITNGGNREETKSQSYSVTDNYVEEEAASCPEGDQDCIDLIKDEFIIEGGRFIRTSINDVPQMEAITNKYMPGSI
GN01    KLEGKSSPNVEGTHSYITNGGNREETKSQSYSVTDNYVEEEAASCQEGDQNCIDLIKDEFIIEGGRFIRTSINDVPQMEAITNKYMPGSI
KE01    KLEGKSSPNVEGTHSYITNGGNIEETKSQSYSVTDNYVEEEAASCPEGDQDCIDLIKDEFIIEGGRVIRTSINDVPQMEAITNKYMPGSI
KH02    KLEGKSSPNVEGTHSYITNGGNIEETKSQSYSVTDNYVEEEAASCPEGDQDCIDLIKDEFIIEGGRFIRTSINDVPQMEAITNKYMPGSI
GB4     KLEGKSSPNVEGTHSYITNGGNREETKSQSYSVTDNYVEEEAASCPEGDQDCIDLIKDEFIIEGGRFIRTSINDVPQMEAITNKYMPGSI
```

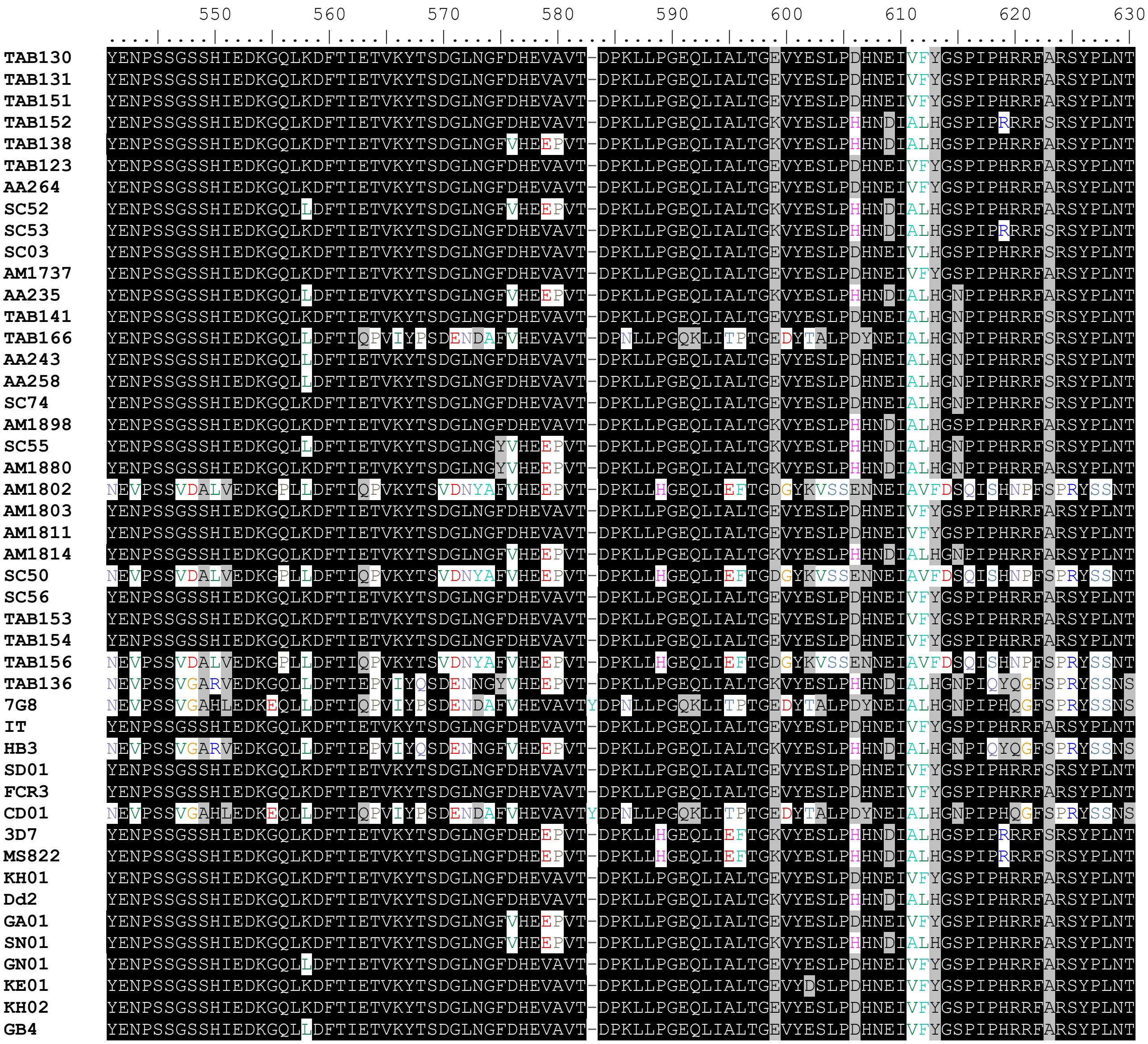
550 560 570 580 590 600 610 620 630
TAB130 YENPSSGSSHIEDKGQLKDFTIETVKYTSDGLNGFDHEVAVT-DPKLLPGEQLIALTGEVYESLPDHNEIVFYGSPIPHRRFARSYPLNT
TAB131 YENPSSGSSHIEDKGQLKDFTIETVKYTSDGLNGFDHEVAVT-DPKLLPGEQLIALTGEVYESLPDHNEIVFYGSPIPHRRFARSYPLNT
TAB151 YENPSSGSSHIEDKGQLKDFTIETVKYTSDGLNGFDHEVAVT-DPKLLPGEQLIALTGEVYESLPDHNEIVFYGSPIPHRRFARSYPLNT
TAB152 YENPSSGSSHIEDKGQLKDFTIETVKYTSDGLNGFDHEVAVT-DPKLLPGEQLIALTGKVYESLPHHNDIALHGSPIPRRRFSRSYPLNT
TAB138 YENPSSGSSHIEDKGQLKDFTIETVKYTSDGLNGFVHEEPVT-DPKLLPGEQLIALTGKVYESLPHHNDIALHGSPIPHRRFARSYPLNT
TAB123 YENPSSGSSHIEDKGQLKDFTIETVKYTSDGLNGFDHEVAVT-DPKLLPGEQLIALTGEVYESLPDHNEIVFYGSPIPHRRFARSYPLNT
AA264 YENPSSGSSHIEDKGQLKDFTIETVKYTSDGLNGFDHEVAVT-DPKLLPGEQLIALTGEVYESLPDHNEIVFYGSPIPHRRFARSYPLNT
SC52 YENPSSGSSHIEDKGQLLDFTIETVKYTSDGLNGFVHEEPVT-DPKLLPGEQLIALTGKVYESLPHHNDIALHGSPIPHRRFARSYPLNT
SC53 YENPSSGSSHIEDKGQLKDFTIETVKYTSDGLNGFDHEVAVT-DPKLLPGEQLIALTGKVYESLPHHNDIALHGSPIPRRRFSRSYPLNT
SC03 YENPSSGSSHIEDKGQLKDFTIETVKYTSDGLNGFDHEVAVT-DPKLLPGEQLIALTGEVYESLPDHNEIVLHGSPIPHRRFSRSYPLNT
AM1737 YENPSSGSSHIEDKGQLKDFTIETVKYTSDGLNGFDHEVAVT-DPKLLPGEQLIALTGEVYESLPDHNEIVFYGSPIPHRRFARSYPLNT
AA235 YENPSSGSSHIEDKGQLLDFTIETVKYTSDGLNGFVHEEPVT-DPKLLPGEQLIALTGKVYESLPHHNDIALHGNPIPHRRFARSYPLNT
TAB141 YENPSSGSSHIEDKGQLKDFTIETVKYTSDGLNGFDHEVAVT-DPKLLPGEQLIALTGEVYESLPDHNEIALHGNPIPHRRFSRSYPLNT
TAB166 YENPSSGSSHIEDKGQLLDFTIQPVIYPSDENDAFVHEVAVT-DPNLLPGQKLITPTGEDYTALPDYNEIALHGNPIPHRRFSRSYPLNT
AA243 YENPSSGSSHIEDKGQLLDFTIETVKYTSDGLNGFDHEVAVT-DPKLLPGEQLIALTGEVYESLPDHNEIALHGNPIPHRRFSRSYPLNT
AA258 YENPSSGSSHIEDKGQLLDFTIETVKYTSDGLNGFDHEVAVT-DPKLLPGEQLIALTGEVYESLPDHNEIALHGNPIPHRRFSRSYPLNT
SC74 YENPSSGSSHIEDKGQLKDFTIETVKYTSDGLNGFDHEVAVT-DPKLLPGEQLIALTGEVYESLPDHNEIALHGNPIPHRRFSRSYPLNT
AM1898 YENPSSGSSHIEDKGQLKDFTIETVKYTSDGLNGFDHEVAVT-DPKLLPGEQLIALTGKVYESLPHHNDIALHGSPIPHRRFARSYPLNT
SC55 YENPSSGSSHIEDKGQLLDFTIETVKYTSDGLNGYVHEEPVT-DPKLLPGEQLIALTGKVYESLPHHNDIALHGNPIPHRRFSRSYPLNT
AM1880 YENPSSGSSHIEDKGQLKDFTIETVKYTSDGLNGYVHEEPVT-DPKLLPGEQLIALTGKVYESLPHHNDIALHGNPIPHRRFARSYPLNT
AM1802 NEVPSSVDALVEDKGPLLDFTIQPVKYTSVDNYAFVHEEPVT-DPKLLHGEQLIEFTGDGYKVSSENNEIAVFDSQISHNPFSPRYSSNT
AM1803 YENPSSGSSHIEDKGQLKDFTIETVKYTSDGLNGFDHEVAVT-DPKLLPGEQLIALTGEVYESLPDHNEIVFYGSPIPHRRFARSYPLNT
AM1811 YENPSSGSSHIEDKGQLKDFTIETVKYTSDGLNGFDHEVAVT-DPKLLPGEQLIALTGEVYESLPDHNEIVFYGSPIPHRRFARSYPLNT
AM1814 YENPSSGSSHIEDKGQLKDFTIETVKYTSDGLNGFVHEEPVT-DPKLLPGEQLIALTGKVYESLPHHNDIALHGNPIPHRRFARSYPLNT
SC50 NEVPSSVDALVEDKGPLLDFTIQPVKYTSVDNYAFVHEEPVT-DPKLLHGEQLIEFTGDGYKVSSENNEIAVFDSQISHNPFSPRYSSNT
SC56 YENPSSGSSHIEDKGQLKDFTIETVKYTSDGLNGFDHEVAVT-DPKLLPGEQLIALTGEVYESLPDHNEIVFYGSPIPHRRFARSYPLNT
TAB153 YENPSSGSSHIEDKGQLKDFTIETVKYTSDGLNGFDHEVAVT-DPKLLPGEQLIALTGEVYESLPDHNEIVFYGSPIPHRRFARSYPLNT
TAB154 YENPSSGSSHIEDKGQLKDFTIETVKYTSDGLNGFDHEVAVT-DPKLLPGEQLIALTGEVYESLPDHNEIVFYGSPIPHRRFARSYPLNT
TAB156 NEVPSSVDALVEDKGPLLDFTIQPVKYTSVDNYAFVHEEPVT-DPKLLHGEQLIEFTGDGYKVSSENNEIAVFDSQISHNPFSPRYSSNT
TAB136 NEVPSSVGARVEDKGQLLDFTIEPVIYQSDENNGYVHEEPVT-DPKLLPGEQLIALTGKVYESLPHHNDIALHGNPIQYQGFSPRYSSNS
7G8 NEVPSSVGAHLEDKEQLLDFTIQPVIYPSDENDAFVHEVAVTYDPNLLPGQKLITPTGEDYTALPDYNEIALHGNPIPHQGFSPRYSSNS
IT YENPSSGSSHIEDKGQLKDFTIETVKYTSDGLNGFDHEVAVT-DPKLLPGEQLIALTGEVYESLPDHNEIVFYGSPIPHRRFARSYPLNT
HB3 NEVPSSVGARVEDKGQLLDFTIEPVIYQSDENNGFVHEEPVT-DPKLLPGEQLIALTGKVYESLPHHNDIALHGNPIQYQGFSPRYSSNS
SD01 YENPSSGSSHIEDKGQLKDFTIETVKYTSDGLNGFDHEVAVT-DPKLLPGEQLIALTGEVYESLPDHNEIVFYGSPIPHRRFSRSYPLNT
FCR3 YENPSSGSSHIEDKGQLKDFTIETVKYTSDGLNGFDHEVAVT-DPKLLPGEQLIALTGEVYESLPDHNEIVFYGSPIPHRRFARSYPLNT
CD01 NEVPSSVGAHLEDKEQLLDFTIQPVIYPSDENDAFVHEVAVTYDPNLLPGQKLITPTGEDYTALPDYNEIALHGNPIPHQGFSPRYSSNS
3D7 YENPSSGSSHIEDKGQLKDFTIETVKYTSDGLNGFDHEEPVT-DPKLLHGEQLIEFTGKVYESLPHHNDIALHGSPIPRRRFSRSYPLNT
MS822 YENPSSGSSHIEDKGQLKDFTIETVKYTSDGLNGFDHEEPVT-DPKLLHGEQLIEFTGKVYESLPHHNDIALHGSPIPRRRFSRSYPLNT
KH01 YENPSSGSSHIEDKGQLKDFTIETVKYTSDGLNGFDHEVAVT-DPKLLPGEQLIALTGEVYESLPDHNEIVFYGSPIPHRRFARSYPLNT
Dd2 YENPSSGSSHIEDKGQLKDFTIETVKYTSDGLNGFDHEVAVT-DPKLLPGEQLIALTGKVYESLPHHNDIALHGSPIPHRRFARSYPLNT
GA01 YENPSSGSSHIEDKGQLKDFTIETVKYTSDGLNGFVHEEPVT-DPKLLPGEQLIALTGEVYESLPDHNEIVFYGSPIPHRRFARSYPLNT
SN01 YENPSSGSSHIEDKGQLKDFTIETVKYTSDGLNGFVHEEPVT-DPKLLPGEQLIALTGKVYESLPHHNDIALHGSPIPHRRFARSYPLNT
GN01 YENPSSGSSHIEDKGQLLDFTIETVKYTSDGLNGFDHEVAVT-DPKLLPGEQLIALTGEVYESLPDHNEIVFYGSPIPHRRFARSYPLNT
KE01 YENPSSGSSHIEDKGQLKDFTIETVKYTSDGLNGFDHEVAVT-DPKLLPGEQLIALTGEVYDSLPDHNEIVFYGSPIPHRRFARSYPLNT
KH02 YENPSSGSSHIEDKGQLKDFTIETVKYTSDGLNGFDHEVAVT-DPKLLPGEQLIALTGEVYESLPDHNEIVFYGSPIPHRRFARSYPLNT
GB4 YENPSSGSSHIEDKGQLLDFTIETVKYTSDGLNGFDHEVAVT-DPKLLPGEQLIALTGEVYESLPDHNEIVFYGSPIPHRRFARSYPLNT

```
                640       650       660       670       680       690       700       710       720
        ....|....|....|....|....|....|....|....|....|....|....|....|....|....|....|....|
TAB130  TTTLEGTSKKPSTIESLTSLFENFFDSIKSSSRTRRSVNSALEFSESTTRPESSYSLPQIILYFEAPGQGTVVGRLGQVNSFSSLAQTSQ
TAB131  TTTLEGTSQKPSAVETIMSLFSNFFDSIKSSSRTRRSVKHDLELSESTTSPTNSYSLPQITLEFEAPAQGTVMGMLGQVNSFSSLAQTSQ
TAB151  TTTLEGTSQKPSAVETIMSLFSNFFDSIKSSSRTRRSVKHDLELSESTTSPTNSYSLPQITLEFEAPAQGTVMGMLGQVNSFSSLAQTSQ
TAB152  TTTLEGTSQKPSAVETIMSLFRNIFDSIKSSSITRRSVNSALEFSESTTRPGNSFSLPQMTWEFEAPGQGTVMRMLGQKNSFKTLAQTKQ
TAB138  TTTLEGTSQKPSAVETIMSLFRNIFDSIKSSSRTRRSVNQDLELSESTTSPTNSYSLPQITLEFEAPAQGTVVGRLGQVNSFSSLAQTSQ
TAB123  TTTLEGTSKKPSTIESLTSLFENFFDSIKSSSRTRRSVNQDLELSESTTSPTNSYSLPQITLYFEAPGQGTVMGMLGQVNSYTSLGQTKQ
AA264   TTTLEGTSKKPSTIESLTSLFENFFDSIKSSSRTRRSVNQDLELSESAAMTSSTSPLRQITLDIKSHGENMVMGMLGHGNHFESLEQKRG
SC52    TTTLEGTSQKPSAVETIMSLFSNFFDSIKSSSRTRRSVKHDLELSESTTSPTNSYSLPQMTWEFEAPGQGTVMRMLGQKNSFKTLAQTKQ
SC53    TTTLEGTSQKPSAVETIMSLFRNIFDSIKSSSITRRSVNSALEFSESTTRPGNSFSLPQMTWEFEAPGQGTVMRMLGQKNSFKTLAQTKQ
SC03    TTTLEGTSQKPSAVETIMSLFRNFFDSIKSSSRTRRSVNSAFELNESTTMPANSYTLNQITLEFEAPAQGTVVGRLGQVNSFSSLAQTRQ
AM1737  TTTLEGTSKKPSTIESLTSLFENFFDSIKSSSRTRRSVNHDLELSESTTSPTNSYSLPQMTWEFEAPGQGTVVGRLGQVNSFSSLAQTSQ
AA235   TTTLEGTSQKPSAVETIMSLFSNFFDSIKSSSRTRRSVNQDLELSESTTSPTNSYSLPQMTWEFEAPGQGTVMGMLGQKNSFKTLAQTKQ
TAB141  TTTLEGTSQKPSAVETIMSLFSNFFDSIKSSSRTRRSVNQDLELSVSTTSPTNSYSLPQITLEFEAPAQGTVMGMLGQKNSFKTLAQTKQ
TAB166  TTTLEGTSQKPSAVETIMSLFRNFFDSIKSSSRTRRSVNSAFELNESTTMPANSYTLNQITLEFEAPAQGTVVGRLGQVNSFSSLAQTRQ
AA243   TTTLEGTSQKPSAVETIMSLFRNFFDSIKSSSRTRRSVNSAFELNESTTMPANSYTLNQITLEFEAPAQGTVVGRLGQVNSFSSLAQTRQ
AA258   TTTLEGTSQKPSAVETIMSLFRNFFDSIKSSSRTRRSVNSAFELNESTTMPANSYTLNQITLEFEAPAQGTVVGRLGQVNSFSSLAQTRQ
SC74    TTTLEGTSQKPSAVETIMSLFSNFFDSIKSSSRTRRSVNHDLELSESTTSPTNSYSLPQITLEFEAPAQGMVMGMLGQKNSFKTLAQTKQ
AM1898  TTTLEGTSKKPSTIESLTSLFENFFDSIKSSSRTKRSINSALEFSEFTTRPESSYSLPQIILDFEAPGQETVMRMLGNGNSYTSLAQKSQ
SC55    TTTLEGTSQKPSAVETIMSLFRNFFDSIKSSSRTRRSINSALEFSEFTTRPESSYSLPQITLEFEAPGQETVMRMLGQVNSFSSLAQTSQ
AM1880  TTTLEGTSQKPSAVETIMSLFRNFVDAIKSSVISKRSINSALEFSEFTTRPESSYSLPQIILDFEAPGQETVMRMLGNGNSYTSLAQKSQ
AM1802  VTSIGGTSKKPSTIESLTSLFENFVDAIKSSVITKRSVNSAFELNESTTMPANSYTLNQITLDSEPPLQGTVMGMLGQVNSYTSLGQTKQ
AM1803  TTTLEGTSQKPSAVETIMSLFRNFFDSIKSSSITRRSVNQDLELSVSTTSPTNSYSLPQMTWEFEAPGQGTVMGMLGQVNSFSSLAQTSQ
AM1811  TTTLEGTSQKPSAVETIMSLFRNFFDSIKSSSITRRSVNQDLELSVSTTSPTNSYSLPQITLEFEAPAQGTVMGMLGQVNSFSSLAQTSQ
AM1814  TTTLEGTSQKPSAVETIMSLFRNFFDSIKSSVISKRSINSALEFSEFTTRPESSYSLPQIILDFEAPGQGTVMGMLGQVNSYTSLGQTKQ
SC50    VTSIGGTSKKPSTIESLTSLFENFVDAIKSSVITKRSVNSAFELNESTTMPANSYTLNQITLDSEPPLQGTVMGMLGQVNSYTSLGQTKQ
SC56    TTTLEGTSQKPSAVETIMSLFRNFFDSIKSSSITRRSVNQDLELSVSTTSPTNSYSLPQITLEFEAPAQGTVMGMLGQVNSFSSLAQTSQ
TAB153  TTTLEGTSQKPSAVETIMSLFRNFFDSIKSSSRTRRSVNQDLELSESTTSPTNSYSLPQITLEFEAPAQGTVVGRLGQVNSFSSLAQTSQ
TAB154  TTTLEGTSQKPSAVETIMSLFSNFFDSIKSSSRTRRSVKHDFELSESAAMTSSTSPLRQITLDIKSHGENMVMGMLGHGNHFESLEQKRG
TAB156  VTSIGGTSKKPSTIESLTSLFENFVDAIKSSVITKRSVNSAFELNESTTMPANSYTLNQITLDSEPPLQGTVMGMLGQVNSYTSLGQTKQ
TAB136  ITSIEGTSNNVSTIESLTSIIGNFVDAIKSSVISKRSINSALEFSEFTTRPESSYSLPQIILDFEAPGQETVMRMLGNGNSYTSLAQKSQ
7G8     ITSIEGTSNNVSTIESLTSIIGNFVDAIRNSVISKRSTNSALEFSESTTRPESSYSLPQIILYFQAPGQETVMGMLGNGNSYTSLAQKSQ
IT      TTTLEGTSQKPSAVETIMSLFRNIFDSIKSSSITRRSVNQDLELSVSTTSPTNSYSLPQITLEFEAPAQGTVMGMLGQVNPFSSLAQTSQ
HB3     ITSIEGTSNNVSTIESLTSIIGNFVDAIKSSVISKRSINSALEFSESTTRPESSYSLPQIILDFEAPGQETVMRMLGNGNSYTSLAQKSQ
SD01    TTTLEGTSQKPSAVETIMSLFRNIFDSIKSSSITRRSVKHDLELSVSTTSPTNSYSLPQITLEFEAPAQGTVMGMLGQVNSFSSLAQTSQ
FCR3    TTTLEGTSQKPSAVETIMSLFRNIFDSIKSSSITRRSVNQDLELSVSTTSPTNSYSLPQITLEFEAPAQGTVMGMLGQVNPFSSLAQTSQ
CD01    ITSIEGTSNNVSTIESLTSIIGNFVDAIRNSVISKRSTNSALEFSESTTRPESSYSLPQIILYFQAPGQETVMGMLGNGNSYTSLAQKSQ
3D7     TTTLEGTSQKPSAVETIMSLFRNIFDSIKSSSITRRSVNSALEFSESTTRPGNSFSLPQMTWEFEAPGQGTVMRMLGQKNSFKTLAQTKQ
MS822   TTTLEGTSQKPSAVETIMSLFRNIFDSIKSSSITRRSVNSALEFSESTTRPGNSFSLPQMTWEFEAPGQGTVMRMLGQKNSFKTLAQTKQ
KH01    TTTLEGTSQKPSAVETIMSLFSNFFDSIKSSSRTRRSVKHDLELSESTTSPTNSYSLPQITLEFEAPAQGTVMGMLGQVNSFSSLAQTSQ
Dd2     TTTLEGTSQKPSAVETIMSLFSNFFDSIKSSSITRRSVKHDLELSESTTSPTNSYSLPQMTWEFEAPGQGTVMRMLGQKNSFKTLAQTKQ
GA01    TTTLEGTSKKPSTIESLTSLFENFFDSIKSSSRTRRSVNQDLELSESAAMTSSTSPLRQITLDSEPPLQGTVMGMLGQVNSYTSLGQTKQ
SN01    TTTLEGTSQKPSAVETIMSLFSNFFDSIKSSSRTRRSVKHDLELSESTTSPTNSYSLPQITLEFEAPAQGTVVGRLGQVNSFSSLAQTSQ
GN01    TTTLEGTSQKPSAVETIMSLFSNFFDSIKSSSRTRRSVKHDLELSESTTSPTNSYSLPQIILDFEAPGQETVMRMLGNGNSYTSLGQTKQ
KE01    TTTLEGTSKKPSTIESLTSLFENFFDSIKSSSRTRRSVNQDLELSESAAMTSSTSPLRQITLDIKSHGENMVMGMLGHGNHFESLEQKRG
KH02    TTTLEGTSKKPSTIESLTSLFENFFDSIKSSSRTRRSVNQDLELSESAAMTSSTSPLRQITLDIKSHGENMVMGMLGHGNHFESLEQKRG
GB4     TTTLEGTSQKPSAVETIMSLFSNFFDSIKSSSRTRRSVKHDLELSESTTSPTNSYSLPQMTWEIEDPGQGTVMRMLGQKNSFKTLAQTKQ
```

```
                 730       740       750       760       770       780       790       800       810
            ....|....|....|....|....|....|....|....|....|....|....|....|....|....|....|....|....|
TAB130      EDSTSSSESRSSSFNNNSQIIPHILKTQVDGTYVVSG-HKANVPKGIEHYIKYVPVALAVFGVLFVFILFNKINPFGTFSSKKKKGKSDE
TAB131      EDSTSSSESRSSSFNNNSQIIPHILKTQVDGTYVVSG-HKANVPKGIEHYIKYVPVALAVFGVLFVFILFNKINPFGTFSSKKKKGKSDE
TAB151      EDSTSSSESRSSSFNNNSQIIPHILKTQVDGTYVVSG-HKANVPKGIEHYIKYVPVALAVFGVLFVFILFNKINPFGTFSSKKKKGKSDE
TAB152      EVSSSSSESRSSSFNNNSQIIPHILKTQVDGTYVVSG-HKANVPKGIEHYIKYVPVALAVFGVLFVFILFNKINPFGTFSSKKKKGKSDE
TAB138      EDSTSSSESRSSSFNNNSQIIPHILKRPVNGTYVVSGGHKANVPKGIEHYIKYVPVALAVFGVLFVFILFNKINPFGTFSSKKKKGKSDE
TAB123      EVASSSPGSRSSSFNHNSQTIHPILKTQVDGTYVVSG-HKANVPKGIEHYIKYVPVALAVFGVLFVFILFNKINPFGTFSSKKKKGKSDE
AA264       EDSSSNHGSPSHSFSLSSKTIHPILKTQVDGTYVVSG-HKANVPKGIEHYIKYVPVALAVFGVLFVFILFNKINPFGTFSSKKKKGKSDE
SC52        EVSSSSSESRSSSFSHNSQIMPTILKTQVDGTYVVSGGHKTNVPKGIEHYIKYVPVALAVFGVLFVFILFNKINPFGTFSSKKKKGKSDE
SC53        EVSSSSSESRSSSFNNNSQIIPHILKTQVDGTYVVSG-HKANVPKGIEHYIKYVPVALAVFGVLFVFILFNKINPFGTFSSKKKKGKSDE
SC03        EDSTSSSESRSSSFNNNSQIIPHILKRPVNGTYVVSGGHKTNVPKGIEHYIKYVPVALAVFGVLFVFILFNKINPFGTFSSKKKKGKSDE
AM1737      EDSTSSSESRSSSFNNNSQIIPHILKTQVDGTYVVSG-HKANVPKGIEHYIKYVPVALAVFGVLFVFILFNKINPFGTFSSKKKKGKSDE
AA235       EVSSSSSESRSSSFNNNSKIIPHILKTQVDGTYVVSG-HKANVPKGIEHYIKYVPVALAVFGVLFVFILFNKINPFGTFSSKKKKGKSDE
TAB141      EVSSSSSESRSSSFNNNSKIIPHILKTQVDGTYVVSG-HKANVPKGIEHYIKYVPVALAVFGVLFVFILFNKINPFGTFSSKKKKGKSDE
TAB166      EDSTSSSESRSSSFNNNSQIIPHILKTQVDGTYVVSG-HKANVPKGIEHYIKYVPVALAVFGVLFVFILFNKINPFGTFSSKKKKGKSDE
AA243       EDSTSSSESRSSSFNNNSQIIPHILKTQVDGTYVVSG-HKANVPKGIEHYIKYVPVALAVFGVLFVFILFNKINPFGTFSSKKKKGKSDE
AA258       EDSTSSSESRSSSFNNNSQIIPHILKTQVDGTYVVSG-HKANVPKGIEHYIKYVPVALAVFGVLFVFILFNKINPFGTFSSKKKKGKSDE
SC74        EVSSSSSESRSSSFNNNSKIIPHILKTQVDGTYVVSG-HKANVPKGIEHYIKYVPVALAVFGVLFVFILFNKINPFGTFSSKKKKGKSDE
AM1898      EVSSSSPVSRSSSFNHNSQTINRILKAQVDGTYVVSG-HKVNVPKGIEHYIKYVPVALAVFGVLFVFILFNKINPFGTFSSKKKKGKSDE
SC55        EVSSSSSESRSSSFNHNSQIIPHILKTQVDGTYVVSG-HKVNVPKGIEHYIKSVPVALAVFGVLFVFILFNKINPFGTFSSKKKKGKSDE
AM1880      EVSSSSPVSRSSSFNHNSQTINRILKAQVDGTYVVSG-HKVNVPKGIEHYIKSVPVALAVFGVLFVFILFNKINPFGTFSSKKKKRKVRR
AM1802      EVASSSPGSPSHSFTLRSKTMHPILKRQFEGTYVVSG-DKANIPKGIEHYIKSVPVALAVFGVLFVFILFNKINPFGTFSSKKKKRKVRR
AM1803      EDSTSSSESRSSSFNNNSQIIPHILKRPVNGTYVVSG-HKANVPKGIEHYIKSVPVALAVFGVLFVFILFNKINPFGTFSSKKKKRKVRR
AM1811      EDSTSSSESRSSSFNNNSQIIPHILKRPVNGTYVVSGGHKANVPKGIEHYIKSVPVALAVFGVLFVFILFNKINPFGTFSSKKKKRKVRR
AM1814      EVSSSSPVSRSSSFNHNSQTINRILKAQVDGTYVVSG-HKANVPKGIEHYIKSVPVALAVFGVLFVFILFNKINPFGTFSSKKKKRKVRR
SC50        EVASSSPGSPSHSFTLRSKTMHPILKRQFEGTYVVSG-DKANIPKGIEHYIKSVPVALAVFGVLFVFILFNKINPFGTFSSKKKKRKVRR
SC56        EDSTSSSESRSSSFNNNSQIIPHILKRPVNGTYVVSGGHKANVPKGIEHYIKSVPVALAVFGVLFVFILFNKINPFGTFSSKKKKRKVRR
TAB153      EDSTSSSESRSSSFNNNSQIIPHILKRPVNGTYVVSGGHKANVPKGIEHYIKSVPVALAVFGVLFVFILFNKINPFGTFSSKKKKRKVRR
TAB154      EDSSSNHGSPSHSFSLSSKTIHPILKTQVDGTYVVSG-HKANVPKGIEHYIKYVPVALAVFGVLFVFILFNKINPFETFSSKKKKRKVRR
TAB156      EVASSSPGSPSHSFTLRSKTMHPILKRQFEGTYVVSG-DKANIPKGIEHYIKSVPVALAVFGVLFVFILFNKINPFGTFSSKKKKRKVRR
TAB136      EVSSSSPVSRSSSFNHNSQTINRILKAQVDGTYVVSG-HKVNVPKGIEHYIKSVPVALAVFGVLFVFILFNKINPFGTFSSKKKKRKVRR
7G8         EVSSSSPVSRSSSFNHNSQTINRILKAQVDGTYVVSG-HKVNVPKGIEHYIKSVPVALAVFGVLFVFILFNKINPFETFSSKKKKRKVRR
IT          EDSTSSSESRSSSFSHNSQIMPTILKRPINGNYVVSGGHKTNVPKGIEHYIKSVPVALAVFGVLFVFILFNKINPFGTFSSKKKKRKVRR
HB3         EVSSSSPVSRSSSFNHNSQTINRILKAQVDGTYVVSG-HKVNVPKGIEHYIKSVPVALAVFGVLFVFILFNKINPFETFSSKKKKRKVRR
SD01        EDSTSSSESRSSSFNNNSQIIPHILKRPVNGTYVVSGGHKANVPKGIEHYIKSVPVALAVFGVLFVFILFNKINPFGTFSSKKKKRKVRR
FCR3        EDSTSSSESRSSSFSHNSQIMPTILKRPINGNYVVSGGHKTNVPKGIEHYIKSVPVALAVFGVLFVFILFNKINPFGTFSSKKKKRKVRR
CD01        EVSSSSPVSRSSSFNHNSQTINRILKAQVDGTYVVSG-HKVNVPKGIEHYIKSVPVALAVFGVLFVFILFNKINPFETFSSKKKKRKVRR
3D7         EVSSSSSESRSSSFNNNSQIIPHILKTQVDGTYVVSG-HKANVPKGIEHYIKYVPVALAVFGVLFVFILFNKINPFGTFSSKKKKGKSDE
MS822       EVSSSSSESRSSSFNNNSQIIPHILKTQVDGTYVVSG-HKANVPKGIEHYIKYVPVALAVFGVLFVFILFNKINPFGTFSSKKKKGKSDE
KH01        EDSTSSSESRSSSFNNNSQIIPHILKTQVDGTYVVSG-HKANVPKGIEHYIKYVPVALAVFGVLFVFILFNKINPFGTFSSKKKKGKSDE
Dd2         EVSSSSSESRSSSFSHNSQIMPTILKRPINGNYVVSGGHKTNVPKGIEHYIKYVPVALAVFGVLFVFILFNKINPFGTFSSKKKKGKSDE
GA01        EVASSSPGSPSHSFSLSSKTIPHILKTQVDGTYVVSG-HKANVPKGIEHYIKYVPVALAVFGVLFVFILFNKINPFGTFSSKKKKGKSDE
SN01        EDSTSSSESRSSSFNNNSQIIPHILKTQVDGTYVVSG-HKANVPKGIEHYIKYVPVALAVFGVLFVFILFNKINPFGTFSSKKKKGKSDE
GN01        EVSSSSSESRSSSFNNNSQIIPHILKTQVDGTYVVSG-HKANVPKGIEHYIKYVPVALAVFGVLFVFILFNKINPFGTFSSKKKKGKSDE
KE01        EDSSSNHGSPSHSFSLSSKTIHPILKTQVDGTYVVSG-HKANVPKGIEHYIKYVPVALAVFGVLFVFILFNKINPFGTFSSKKKKGKSDE
KH02        EDSSSNHGSPSHSFSLSSKTIHPILKTQVDGTYVVSG-HKANVPKGIEHYIKYVPVALAVFGVLFVFILFNKINPFGTFSSKKKKGKSDE
GB4         EVSSSSSESRSSSFSHNSQIMPTILKRPINGNYVVSGGHKTNVPKGIEHYIKYVPVALAVFGVLFVFILFNKINPFGTFSSKKKKGKSDE
```

```
                820       830       840       850       860       870       880       890       900
        ....|....|....|....|....|....|....|....|....|....|....|....|....|....|....|....|....|....|
TAB130  EGMM--------------------------------------------------------------------------------------
TAB131  EGMM--------------------------------------------------------------------------------------
TAB151  EGMM--------------------------------------------------------------------------------------
TAB152  EGMM--------------------------------------------------------------------------------------
TAB138  EGMM--------------------------------------------------------------------------------------
TAB123  EGMM--------------------------------------------------------------------------------------
AA264   EGMM--------------------------------------------------------------------------------------
SC52    EGMM--------------------------------------------------------------------------------------
SC53    EGMM--------------------------------------------------------------------------------------
SC03    EGMM--------------------------------------------------------------------------------------
AM1737  EGMM--------------------------------------------------------------------------------------
AA235   EGMMRKY-----------------------------------------------------------------------------------
TAB141  EGMMRKY-----------------------------------------------------------------------------------
TAB166  EGMMRKY-----------------------------------------------------------------------------------
AA243   EGMMRKY-----------------------------------------------------------------------------------
AA258   EGMMRKY-----------------------------------------------------------------------------------
SC74    EGMMRKY-----------------------------------------------------------------------------------
AM1898  EGMMRKY-----------------------------------------------------------------------------------
SC55    EGMMRKY-----------------------------------------------------------------------------------
AM1880  RRYDEKILKREGKKCFENLMQHSKEWIDKERHDILPNSLYNIEKKEGTEYNVLEEKEYPLIDEVLKEEDIKKVNKKKIKKGHVKSVLIDN
AM1802  RRYDEKILKREGKKCFENLMQHSKEWIDKESHDILPNSLYNIEKKEGTEYNVLEEKEYPLIDEVLKEEDIKKVNKKKIKKGHVKSVLIDN
AM1803  RRYDEKILKREGKKCFENLMQHSKEWIDKESHDILPNSLYNIEKKEGTEYNVLEEKEYPLIDEVLKEEDIKKVNKKKIKKGHVKSVLIDN
AM1811  RRYDEKILKREGKKCFENLMQHSKEWIDKESHDILPNSLYNIEKKEGTEYNVLEEKEYPLIDEVLKEEDIKKVNKKKIKKGHVKSVLIDN
AM1814  RRYDEKILKREGKKCFENLMQHSKEWIDKESHDILPNSLYNIEKKEGTEYNVLEEKEYPLIDEVLKEEDIKKVNKKKIKKGHVKSVLIDN
SC50    RRYDEKILKREGKKCFENLMQHSKEWIDKESHDILPNSLYNIEKKEGTEYNVLEEKEYPLIDEVLKEEDIKKVNKKKIKKGHVKSVLIDN
SC56    RRYDEKILKREGKKCFENLMQHSKEWIDKESHDILPNSLYNIEKKEGTEYNVLEEKEYPLIDEVLKEEDIKKVNKKKIKKGHVKSVLIDN
TAB153  RRYDEKILKREGKKCFENLMQHSKEWIDKESHDILPNSLYNIEKKEGTEYNVLEEKEYPLIDEVLKEEDIKKVNKKKIKKGHVKSVLIDN
TAB154  RRYDEKILKREGKKCFENLMQHSKEWIDKESHDILPNSLYNIEKKEGTEYNVLEEKEYPLIDEVLKEEDIKKVNKKKIKKGHVKSVLIDN
TAB156  RRYDEKILKREGKKCFENLMQHSKEWIDKESHDILPNSLYNIEKKEGTEYNVLEEKEYPLIDEVLKEEDIKKVNKKKIKKGHVKSVLIDN
TAB136  RRYDEKILKREGKKCFENLMQHSKEWIDKERHDILPNSLYNIEKKEGTEYNVLEEKEYPLIDEVLKEEDIKKVNKKKIKKGHVKSVLIDN
7G8     RRYDEKILKREGKKCFENLMQHSKEWIDKESHDILPNSLYNIEKKEGTEYNVLEEKEYPLIDEVLKEEDIKKVNKKKIKKGHVKSVLIDN
IT      RRYDEKILKREGKKCFENLMQHSKEWIDKERHDILPNSLYNIEKKEGTEYNVLEEKEYPLIDEVLNEEDIKKVNKNKIKKGHVKSVLIDN
HB3     RRYDEKILKREGKKCFENLMQHSKEWIDKESHDILPNSLYNIEKKEGTEYNVLEEKEYPLIDEVLKEEDIKKVNKKKIKKGHVKSVLIDN
SD01    RRYDEKILKREGKKCFENLMQHSKEWIDKESHDILPNSLYNIEKKEGTEYNVLEEKEYPLIDEVLKEEDIKKVNKKKIKKGHVKSVLIDN
FCR3    RRYDEKILKREGKKCFENLMQHSKEWIDKERSDILPNSLYNIEKKEGTEYNVLEEKEYPLIDEVLNEEDIKKVNKNKIKKGHVKSVLIDN
CD01    RRYDEKILKREGKKCFENLMQHSKEWIDKESHDILPNSLYNIEKKEGTEYNVLEEKEYPLIDEVLKEEDIKKVNKKKIKKGHVKSVLIDN
3D7     EGMM--------------------------------------------------------------------------------------
MS822   EGMM--------------------------------------------------------------------------------------
KH01    EGMM--------------------------------------------------------------------------------------
Dd2     EGMM--------------------------------------------------------------------------------------
GA01    EGMM--------------------------------------------------------------------------------------
SN01    EGMM--------------------------------------------------------------------------------------
GN01    EGMM--------------------------------------------------------------------------------------
KE01    EGMM--------------------------------------------------------------------------------------
KH02    EGMM--------------------------------------------------------------------------------------
GB4     EGMM--------------------------------------------------------------------------------------
```

```
                  910       920       930       940       950       960       970       980       990
          ....|....|....|....|....|....|....|....|....|....|....|....|....|....|....|....|....|....|
TAB130    ------------------------------------------------------------------------------------------
TAB131    ------------------------------------------------------------------------------------------
TAB151    ------------------------------------------------------------------------------------------
TAB152    ------------------------------------------------------------------------------------------
TAB138    ------------------------------------------------------------------------------------------
TAB123    ------------------------------------------------------------------------------------------
AA264     ------------------------------------------------------------------------------------------
SC52      ------------------------------------------------------------------------------------------
SC53      ------------------------------------------------------------------------------------------
SC03      ------------------------------------------------------------------------------------------
AM1737    ------------------------------------------------------------------------------------------
AA235     ------------------------------------------------------------------------------------------
TAB141    ------------------------------------------------------------------------------------------
TAB166    ------------------------------------------------------------------------------------------
AA243     ------------------------------------------------------------------------------------------
AA258     ------------------------------------------------------------------------------------------
SC74      ------------------------------------------------------------------------------------------
AM1898    ------------------------------------------------------------------------------------------
SC55      ------------------------------------------------------------------------------------------
AM1880    VKKDRDIPNLIKLHLDAIEECSHDEWEEYKIEFLEICIKEFFKERKIDGQGRMLEKKYKCEDNFLNNIDIWKKKKLMWNKWIEKNRYIMN
AM1802    VKKDRDIPNLIKLHLDAIEECSHDEWEEYKIEFLEICIKEFFKERKIDGQGKILEKQYKCEDNFLNNIDIWKKKKLMWNKWIERNRYIMN
AM1803    VKKDRDIPNLIKLHLDAIEECSHDEWEEYKIEFLEICIKEFFKERKIDGQGRMLEKKYKCEDNFLNNIDIWKKKKLMWNKWIEKNRYIMN
AM1811    VKKDRDIPNLIKLHLDAIEECSHDEWEEYKIEFLEICIKEFFKERKIDGQGRMLEKKYKCEDNFLNNIDIWKKKKLMWNKWIEKNRYIMN
AM1814    VKKDRDIPNLIKLHLDAIEECSHDEWEEYKIEFLEICIKEFFKERKIDGQGRMLEKKYKCEDNFLNNIDIWKKKKLMWNKWIEKNRYIMN
SC50      VKKDRDIPNLIKLHLDAIEECSHDEWEEYKIEFLEICIKEFFKERKIDGQGKILEKQYKCEDNFLNNIDIWKKKKLMWNKWIERNRYIMN
SC56      VKKDRDIPNLIKLHLDAIEECSHDEWEEYKIEFLEICIKEFFKERKIDGQGRMLEKKYKCEDNFLNNIDIWKKKKLMWNKWIEKNRYIMN
TAB153    VKKDRDIPNLIKLHLDAIEECSHDEWEEYKIEFLEICIKEFFKERKIDGQGRMLEKKYKCEDNFLNNIDIWKKKKLMWNKWIEKNRYIMN
TAB154    VKKDRDIPNLIKLHLDAIEECSHDEWEEYKIEFLEICIKEFFKERKIDGQGRMLEKKYKCEDNFLNNIDIWKKKKLMWNKWIEKNRYIMN
TAB156    VKKDRDIPNLIKLHLDAIEECSHDEWEEYKIEFLEICIKEFFKERKIDGQGKILEKQYKCEDNFLNNIDIWKKKKLMWNKWIERNRYIMN
TAB136    VKKDRDIPNLIKLHLDAIEECSHDEWEEYKIEFLEICIKEFFKERKIDGQGRMLEKKYKCEDNFLNNIDIWKKKKLMWNKWIEKNRYIMN
7G8       VKKDRDIPNLIKLHLDAIEECSHDEWEEYKIEFLEICIKEFFKERKIDGQGRMLEKKYKCEDNFLNNIDIWKKKKLMWNKWIEKNRYIMN
IT        VKKDRDIPNLIKLHLDAIEECSHDEWEEYKIEFLEICIKEFFKERKIDGQGRMLEKKYKCEDNFLNNIDIWKKKKLMWNKWIEKNRYIMN
HB3       VKKDRDIPNLIKLHLDAIEECSHYEWEEYKIEFLEICIKEFFKERKIDGQGRMLEKKYKCEDNFLNNIDIWKKKKLMWNKWIEKNRYIMN
SD01      VKKDRDIPNLIKLHLDAIEECSHDEWEEYKIEFLEICIKEFFKERKIDGQGRMLEKKYKCEDNFLNNIDIWKKKKLMWNKWIEKNRYIMN
FCR3      VKKDRDIPNLIKLHLDAIEECSHDEWEEYKIEFLEICIKEFFKERKIDGQGRMLEKKYKCEDNFLNNIDIWKKKKLMWNKWIEKNRYIMN
CD01      VKKDRDIPNLIKLHLDAIEECSHDEWEEYKIEFLEICIKEFFKERKIDGQGRMLEKKYKCEDNFLNNIDIWKKKKLMWNKWIEKNRYIMN
3D7       ------------------------------------------------------------------------------------------
MS822     ------------------------------------------------------------------------------------------
KH01      ------------------------------------------------------------------------------------------
Dd2       ------------------------------------------------------------------------------------------
GA01      ------------------------------------------------------------------------------------------
SN01      ------------------------------------------------------------------------------------------
GN01      ------------------------------------------------------------------------------------------
KE01      ------------------------------------------------------------------------------------------
KH02      ------------------------------------------------------------------------------------------
GB4       ------------------------------------------------------------------------------------------
```

```
              1000      1010      1020      1030      1040      1050      1060      1070      1080
          ....|....|....|....|....|....|....|....|....|....|....|....|....|....|....|....|....|....|
TAB130    ------------------------------------------------------------------------------------------
TAB131    ------------------------------------------------------------------------------------------
TAB151    ------------------------------------------------------------------------------------------
TAB152    ------------------------------------------------------------------------------------------
TAB138    ------------------------------------------------------------------------------------------
TAB123    ------------------------------------------------------------------------------------------
AA264     ------------------------------------------------------------------------------------------
SC52      ------------------------------------------------------------------------------------------
SC53      ------------------------------------------------------------------------------------------
SC03      ------------------------------------------------------------------------------------------
AM1737    ------------------------------------------------------------------------------------------
AA235     ------------------------------------------------------------------------------------------
TAB141    ------------------------------------------------------------------------------------------
TAB166    ------------------------------------------------------------------------------------------
AA243     ------------------------------------------------------------------------------------------
AA258     ------------------------------------------------------------------------------------------
SC74      ------------------------------------------------------------------------------------------
AM1898    ------------------------------------------------------------------------------------------
SC55      ------------------------------------------------------------------------------------------
AM1880    NWKDEEWFDNIKCEWENEIEEYFKLKKYDKNLLEIFGKNKENNKSFMIEILKIIWKKWIEKNVKYINESVTNEWINKLVIKYKNDYIDLK
AM1802    NWKDEEWFDNIKCEWENEIEEYFKLKKYDKNLLEIFEKNRENNKSFMIEILKIIWKKWIEKNVKYINESVTNEWINKLVIKYKNDYIDLK
AM1803    NWKDEEWFDNIKCEWENEIEEYFKLKKYDKNLLEIFGKNKENNKSFMIEILKIIWKKWIEKNVKYINESVTNEWINKLVIKYKNDYIDLK
AM1811    NWKDEEWFDNIKCEWENEIEEYFKLKKYDKNLLEIFGKNKENNKSFMIEILKIIWKKWIEKNVKYINKSVTNEWINKLVIKYKNDYIDLK
AM1814    NWKDEEWFDNIKCEWENEIEEYFKLKKYDKNLLEIFGKNKENNKSFMIEILKIIWKKWIEKNVKYINESVTNEWINKLVIKYKNDYIDLK
SC50      NWKDEEWFDNIKCEWENEIEEYFKLKKYDKNLLEIFEKNRENNKSFMIEILKIIWKKWIEKNVKYINESVTNEWINKLVIKYKNDYIDLK
SC56      NWKDEEWFDNIKCEWENEIEEYFKLKKYDKNLLEIFGKNKENNKSFMIEILKIIWKKWIEKNVKYINKSVTNEWINKLVIKYKNDYIDLK
TAB153    NWKDEEWFDNIKCEWENEIEEYFKLKKYDKNLLEIFGKNKENNKSFMIEILKIIWKKWIEKNVKYINESVTNEWINKLVIKYKNDYIDLK
TAB154    NWKDEEWFDNIKCEWENEIEEYFKLKKYDKNLLEIFGKNKENNKSFMIEILKIIWKKWIEKNVKYINESVTNEWINKLVIKYKNDYIDLK
TAB156    NWKDEEWFDNIKCEWENEIEEYFKLKKYDKNLLEIFEKNRENNKSFMIEILKIIWKKWIEKNVKYINESVTNEWINKLVIKYKNDYIDLK
TAB136    NWKDEEWFDNIKCEWENEIEEYFKLKKYDKNLLEIFGKNKENNKSFMIEILKIIWKKWIEKNVKYINESVTNEWINKLVIKYKNDYIDLK
7G8       NWKDEEWFDNIKCEWENEIEEYFKLKKYDKNLLEIFEKNRENNKSFMIEILKIIWKKWIEKNVKYINESVTNEWINKLVIKYKNDYIDLK
IT        NWKDEEWFDNIKCEWENEIEEYFKLKKYDKNLLEIFGKNKENNKSFMIEILKIIWKKWIEKNVKYINKSVTNEWINKLVIKYKNDYIDLK
HB3       NWKDEEWFDNIKCEWENEIEEYFKLKKYDKNLLEIFGKNKENNKSFMIEILKIIWKKWIEKNVKYINESVTNEWINKLVIKYKNDYIDLK
SD01      NWKDEEWFDNIKCEWENEIEEYFKLKKYDKNLLEIFGKNKENNKSFMIEILKIIWKKWIEKNVKYINESVTNEWINKLVIKYKNDYIDLK
FCR3      NWKDEEWFDNIKCEWENEIEEYFKLKKYDENLLEIFGKNKENHKSFMIEILKIIWKKWIEKNVKYINKSVTNEWINQLVIKYKNDYIDLK
CD01      NWKDEEWFDNIKCEWENEIEEYFKLKKYDKNLLEIFEKNRENNKSFMIEILKIIWKKWIEKNVKYINESVTNEWINKLVIKYKNDYIDLK
3D7       ------------------------------------------------------------------------------------------
MS822     ------------------------------------------------------------------------------------------
KH01      ------------------------------------------------------------------------------------------
Dd2       ------------------------------------------------------------------------------------------
GA01      ------------------------------------------------------------------------------------------
SN01      ------------------------------------------------------------------------------------------
GN01      ------------------------------------------------------------------------------------------
KE01      ------------------------------------------------------------------------------------------
KH02      ------------------------------------------------------------------------------------------
GB4       ------------------------------------------------------------------------------------------
```

```
                1090      1100      1110      1120      1130      1140      1150      1160      1170
        ....|....|....|....|....|....|....|....|....|....|....|....|....|....|....|....|....|....|
TAB130  ------------------------------------------------------------------------------------------
TAB131  ------------------------------------------------------------------------------------------
TAB151  ------------------------------------------------------------------------------------------
TAB152  ------------------------------------------------------------------------------------------
TAB138  ------------------------------------------------------------------------------------------
TAB123  ------------------------------------------------------------------------------------------
AA264   ------------------------------------------------------------------------------------------
SC52    ------------------------------------------------------------------------------------------
SC53    ------------------------------------------------------------------------------------------
SC03    ------------------------------------------------------------------------------------------
AM1737  ------------------------------------------------------------------------------------------
AA235   ------------------------------------------------------------------------------------------
TAB141  ------------------------------------------------------------------------------------------
TAB166  ------------------------------------------------------------------------------------------
AA243   ------------------------------------------------------------------------------------------
AA258   ------------------------------------------------------------------------------------------
SC74    ------------------------------------------------------------------------------------------
AM1898  ------------------------------------------------------------------------------------------
SC55    ------------------------------------------------------------------------------------------
AM1880  KDYTRFLKDIYDIPLDNNNNKNIEYLDNGNLIKIMFIQIYMLALEEYKKEQFFQNVSTFLDICLDDIKEKQTNTEETNNTLKLIRAMENK
AM1802  KDYTRFLKDIYDIPLDNNNNKNIEYLDNDNLIKIMFIQIYMLVLEEYKKEQFFQNVSTFLDICLDDIKEKQTNTDETNNTLKLIRAMENK
AM1803  KDYTRFLKDIYDIPLDNNNNKNIEYLDNDNLIKIMFIQIYMLALEEYKKEQFFQNVSTFLDICLDDIKEKQTNTEETNNTLKLIRAMENK
AM1811  KDYTRFLKDIYDIPLDNNNNKNIEYLDNGNLIKIMFIQIYMLALEEYKKEQFFQNVSTFLDICLDDIKEKQTNTEETNNTLKLIRAMENK
AM1814  KDYTRFLKDIYDIPLDNNNNKNIEYLDNDNLIKIMFIQIYMLALEEYKKEQFFQNVSTFLDICLDDIKEKQTNTEETNNTLKLIRAMENK
SC50    KDYTRFLKDIYDIPLDNNNNKNIEYLDNDNLIKIMFIQIYMLALEEYKKEQFFQNVSTFLDICLDDIKEKQTNTDETNNTLKLIRAMENK
SC56    KDYTRFLKDIYDIPLDNNNNKNIEYLDNGNLIKIMFIQIYMLALEEYKKEQFFQNVSTFLDICLDDIKEKQTNTEETNNTLKLIRAMENK
TAB153  KDYTRFLKDIYDIPLDNNNNKNIEYLDNDNLIKIMFIQIYMLVLEEYKKEQFFQNVSTFLDICLDDIKEKQTNTDETNNTLKLIRAMENK
TAB154  KDYTRFLKDIYDIPLDNNNNKNIEYLDNDNLIKIMFIQIYMLALEEYKKEQFFQNVSTFLDICLDDIKEKQTNTEETNNTLKLIRAMENK
TAB156  KDYTRFLKDIYDIPLDNNNNKNIEYLDNDNLIKIMFIQIYMLVLEEYKKEQFFQNVSTFLDICLDDIKEKQTNTDETNNTLKLIRAMENK
TAB136  KDYTRFLKDIYDIPLDNNNNKNIEYLDNGNLIKIMFIQIYMLALEEYKKEQFFQNVSTFLDICLDDIKEKQTNTEETNNTLKLIRAMENK
7G8     KDYTRFLKDIYDIPLDNNNNKNIEYLDNDNLIKIMFIQIYMLALEEYKKEQFFQNVSTFLDICLDDIKEKQTNTEETNNTLKLIRAMENK
IT      KDYTRFLKDIYDIPLDNNNNKNIEYLDNGNLIKIMFIQIYMLALEEYKKEQFFQNVSTFLDICLDDIKEKQTNTDETNNTLKLIRAMENK
HB3     KDYTRFLKDIYDIPLDNNNNKNIEYLDNDNLIKIMFIQIYMLALEEYKKEQFFQNVSTFLDICLDDIKEKQTNTEETNNTLKLIRAMENK
SD01    KDYTRFLKDIYDIPLDNNNNKNIEYLDNDNLIKIMFIQIYMLALEEYKKEQFFQNVSTFLDICLDDIKEKQTNTEETNNTLKLIRAMENK
FCR3    KDYTRFLKDIYDIPLDNNNNKNIEYLDNGNLIKIMFIQIYMLALEEYKKEQFFQNVSTFLDICLDDIKEKQTNTDETNNTLKLIRAMENK
CD01    KDYTRFLKDIYDIPLDNNNNKNIEYLDNDNLIKIMFIQIYMLVLEEYKKEQFFQNVSTFLDICLDDIKEKQTNTDETNNTLKLIRAMENK
3D7     ------------------------------------------------------------------------------------------
MS822   ------------------------------------------------------------------------------------------
KH01    ------------------------------------------------------------------------------------------
Dd2     ------------------------------------------------------------------------------------------
GA01    ------------------------------------------------------------------------------------------
SN01    ------------------------------------------------------------------------------------------
GN01    ------------------------------------------------------------------------------------------
KE01    ------------------------------------------------------------------------------------------
KH02    ------------------------------------------------------------------------------------------
GB4     ------------------------------------------------------------------------------------------
```

```
                  1180      1190      1200      1210      1220      1230      1240      1250      1260
          ....|....|....|....|....|....|....|....|....|....|....|....|....|....|....|....|....|....|
TAB130    ------------------------------------------------------------------------------------------
TAB131    ------------------------------------------------------------------------------------------
TAB151    ------------------------------------------------------------------------------------------
TAB152    ------------------------------------------------------------------------------------------
TAB138    ------------------------------------------------------------------------------------------
TAB123    ------------------------------------------------------------------------------------------
AA264     ------------------------------------------------------------------------------------------
SC52      ------------------------------------------------------------------------------------------
SC53      ------------------------------------------------------------------------------------------
SC03      ------------------------------------------------------------------------------------------
AM1737    ------------------------------------------------------------------------------------------
AA235     ------------------------------------------------------------------------------------------
TAB141    ------------------------------------------------------------------------------------------
TAB166    ------------------------------------------------------------------------------------------
AA243     ------------------------------------------------------------------------------------------
AA258     ------------------------------------------------------------------------------------------
SC74      ------------------------------------------------------------------------------------------
AM1898    ------------------------------------------------------------------------------------------
SC55      ------------------------------------------------------------------------------------------
AM1880    YILKKNKEKEIINQFKNEMFFKNIMCDWKNCEDKFINDHFIQEKINIHNMKKDIPIKHWNHIYHKWLHEENKKDDIIVPLKRKEKEKEQN
AM1802    YILKKNKEKEIINQFKNEMFFKNIMCDWKNCEDKFINDHFIQEKINIHNMKKDIPIKHWNHIYHKWLHEENKKDDIIVPLKGKEKEKKQN
AM1803    YILKKNKEKEIINQFKNEMFFKNIMCDWKNCEDKFINDHFIQEKINIHNMKKDIPIKHWNHIYHKWLHEENKKDDIIVPLKRKEKEKEQN
AM1811    YILKKNKEKEIINQFKNEMFFKNIMCDWKNCEDKFINDHFIQEKINIHNMKKDIPIKHWNHIYHKWLHEENKKDDIIVPLKRKEKEKEQN
AM1814    YILKKNKEKEIINQFKNEMFFKNIMCDWKNCEDKFINDHFIQEKINIHNMKKDIPIKHWNHIYHKWLHEENKKDDIIVPLKGKEKEKKQN
SC50      YILKKNKEKEIINQFKNEMFFKNIMCDWKNCEDKFINDHFIQEKINIHNMKKDIPIKHWNHIYHKWLHEENKKDDIIVPLKGKEKEKKQN
SC56      YILKKNKEKEIINQFKNEMFFKNIMCDWKNCEDKFINDHFIQEKINIHNMKKDIPIKHWNHIYHKWLHEENKKDDIIVPLKRKEKEKEQN
TAB153    YILKKNKEKEIINQFKNEMFFKNIMCDWKNCEDKFINDHFIQEKINIHNMKKDIPIKHWNHIYHKWLHEENKKDDIIVPLKGKEKEKKQN
TAB154    YILKKNKEKEIINQFKNEMFFKNIMCDWKNCEDKFINDHFIQEKINIHNMKKDIPIKHWNHIYHKWLHEENKKDDIIVPLKGKEKEKKQN
TAB156    YILKKNKEKEIINQFKNEMFFKNIMCDWKNCEDKFINDHFIQEKINIHNMKKDIPIKHWNHIYHKWLHEENKKDDIIVPLKGKEKEKKQN
TAB136    YILKKNKEKEIINQFKNEMFFKNIMCDWKNCEDKFINDHFIQEKINIHNMKKDIPIKHWNHIYHKWLHEENKKDDIIVPLKGKEKEKKQN
7G8       YILKKNKEKEIINQFKNEMFFKNIMCDWKNCEDKFINDHFIQEKINIHNMKKDIPIKHWNHIYHKWLHEENKKDDIIVPLKGKEKEKKQN
IT        YILKKNKEKEIINQFKNEMFFKNIMCDWKNCEDKFINDHFIQEKINIHNMKKDIPIKHWNHIYHKWLHEENKKDDIIVPLKRKEKEKEQN
HB3       YILKKNKEKEIINQFKNEMFFKNIMCDWKNCEDKFINDHFIQEKINIHNMKKDIPIKHWNHIYHKWLHEENKKDDIIVPLKGKEKEKKQN
SD01      YILKKNKEKEIINQFKNEMFFKNIMCDWKNCEDKFINDHFIQEKINIHNMKKDIPIKHWNHIYHKWLHEENKKDDIIVPLKGKEKEKKQN
FCR3      YILKKNKEKEIINQFKNEMFFKNIMCDWKNCEDKFINDHFIQEKINIHNMKKDIPIKHWNHIYHKWLHEENKKDDIIVPLKRKEKEKEQN
CD01      YILKKNKEKEIINQFKNEMFFKNIMCDWKNCEDKFINDHFIQEKINIHNMKKDIPIKHWNHIYHKWLHEENKKDDIIVPLKGKEKEKKQN
3D7       ------------------------------------------------------------------------------------------
MS822     ------------------------------------------------------------------------------------------
KH01      ------------------------------------------------------------------------------------------
Dd2       ------------------------------------------------------------------------------------------
GA01      ------------------------------------------------------------------------------------------
SN01      ------------------------------------------------------------------------------------------
GN01      ------------------------------------------------------------------------------------------
KE01      ------------------------------------------------------------------------------------------
KH02      ------------------------------------------------------------------------------------------
GB4       ------------------------------------------------------------------------------------------
```

```
                 1270      1280      1290      1300      1310      1320      1330      1340      1350
         ....|....|....|....|....|....|....|....|....|....|....|....|....|....|....|....|....|....|
TAB130   ------------------------------------------------------------------------------------------
TAB131   ------------------------------------------------------------------------------------------
TAB151   ------------------------------------------------------------------------------------------
TAB152   ------------------------------------------------------------------------------------------
TAB138   ------------------------------------------------------------------------------------------
TAB123   ------------------------------------------------------------------------------------------
AA264    ------------------------------------------------------------------------------------------
SC52     ------------------------------------------------------------------------------------------
SC53     ------------------------------------------------------------------------------------------
SC03     ------------------------------------------------------------------------------------------
AM1737   ------------------------------------------------------------------------------------------
AA235    ------------------------------------------------------------------------------------------
TAB141   ------------------------------------------------------------------------------------------
TAB166   ------------------------------------------------------------------------------------------
AA243    ------------------------------------------------------------------------------------------
AA258    ------------------------------------------------------------------------------------------
SC74     ------------------------------------------------------------------------------------------
AM1898   ------------------------------------------------------------------------------------------
SC55     ------------------------------------------------------------------------------------------
AM1880   VIDESSLVTKKNKWKTVIEIYMEVMNECKRDEWEEHRGDFLQICLEEFIKKDKDEMRNINEELYIEQSDHMEDMLMLERQKIIWLQWINR
AM1802   IIDESSLVTKKNKWKTVIEIYMEVMNECKRDEWEEHRGDFLQICLEEFIKKDKDEMRNINEELYIEQSDHMEDMLMLERQKIIWLQWINR
AM1803   VIDESSLVTKKNKWKTVIEIYMEVMNECKRDEWEEHRGDFLQICLEEFIKKDNDEVRNINDELYIEQSDHMEDTLMLERQKIIWLQWIKR
AM1811   VIDESSLVTKKNKWKTVIEIYMEVMNECKRDEWEEHRGDFLQICLEEFIKKDNDEVRNINDELYIEQSDHMEDTLMLERQKIIWLQWIKR
AM1814   IIDESSLVTKKNKWKTVIEIYMEVMNECKRDEWEEHRGDFLQICLEEFIKKDKDEMRNINEELYIEQSDHMEDMLMLERQKIIWLQWINR
SC50     IIDESSLVTKKNKWKTVIEIYMEVMNECKRDEWEEHRGDFLQICLEEFIKKDKDEMRNINEELYIEQSDHMEDMLMLERQKIIWLQWINR
SC56     VIDESSLVTKKNKWKTVIEIYMEVMNECKRDEWEEHRGDFLQICLEEFIKKDNDEVRNINDELYIEQSDHMEDTLMLERQKIIWLQWIKR
TAB153   IIDESSLVTKKNKWKTVIEIYMEVMNECKRDEWEEHRGDFLQICLEEFIKKDKDEMRNINEELYIEQSDHMEDMLMLERQKIIWLQWINR
TAB154   IIDESSLVTKKNKWKTVIEIYMEVMNECKRDEWEEHRVDFLQICLEEFIKKDKDEMRNINEELYIEQSDHMEDMLMLERQKIIWLQWINR
TAB156   IIDESSLVTKKNKWKTVIEIYMEVMNECKRDEWEEHRGDFLQICLEEFIKKDKDEMRNINEELYIEQSDHMEDMLMLERQKIIWLQWINR
TAB136   IIDESSLVTKKKQMENRYRNIYGSDE----------------------------------------------------------------
7G8      IIDESSLVTKKNKWKTVIEIYMEVMNECKRDEWEEHRGDFLQICLEEFIKKDKDEMRNINEELYIEQSDHMEDMLMLERQKIIWLQWINR
IT       VIDESSLVTKKNKWKTVIEIYMEVMNECKRDEWEEHRGDFLQICLEEFIKKDKDEMRNINEELYIEQSDHMEDMLMLERQKIIWLQWINR
HB3      IIDESSLVTKKNKWKTVIEIYMEVMNECKRDEWEEHRGDFLQICLEEFIKKDKDEMRNINEELYIEQSDHMEDMLMLERQKIIWLQWINR
SD01     IIDESSLVTKKNKWKTVIEIYMEVMNECKRDEWEEHRVDFLQICLEEFIKKDKDEMRNINEELYIEQSDHMEDMLMLERQKIIWLQWINR
FCR3     VIDESSLVTKKNKWKTVIEIYMEVMNECKRDEWEEHRGDFLQICLEEFIKKDKDEMRNINEELYIEQSDHMEDMLMLERQKIIWLQWINR
CD01     IIDESSLVTKKKQMENRYRNIYGSDE----------------------------------------------------------------
3D7      ------------------------------------------------------------------------------------------
MS822    ------------------------------------------------------------------------------------------
KH01     ------------------------------------------------------------------------------------------
Dd2      ------------------------------------------------------------------------------------------
GA01     ------------------------------------------------------------------------------------------
SN01     ------------------------------------------------------------------------------------------
GN01     ------------------------------------------------------------------------------------------
KE01     ------------------------------------------------------------------------------------------
KH02     ------------------------------------------------------------------------------------------
GB4      ------------------------------------------------------------------------------------------
```

```
                 1360      1370      1380      1390      1400      1410      1420      1430      1440
          ....|....|....|....|....|....|....|....|....|....|....|....|....|....|....|....|....|....|
TAB130    ------------------------------------------------------------------------------------------
TAB131    ------------------------------------------------------------------------------------------
TAB151    ------------------------------------------------------------------------------------------
TAB152    ------------------------------------------------------------------------------------------
TAB138    ------------------------------------------------------------------------------------------
TAB123    ------------------------------------------------------------------------------------------
AA264     ------------------------------------------------------------------------------------------
SC52      ------------------------------------------------------------------------------------------
SC53      ------------------------------------------------------------------------------------------
SC03      ------------------------------------------------------------------------------------------
AM1737    ------------------------------------------------------------------------------------------
AA235     ------------------------------------------------------------------------------------------
TAB141    ------------------------------------------------------------------------------------------
TAB166    ------------------------------------------------------------------------------------------
AA243     ------------------------------------------------------------------------------------------
AA258     ------------------------------------------------------------------------------------------
SC74      ------------------------------------------------------------------------------------------
AM1898    ------------------------------------------------------------------------------------------
SC55      ------------------------------------------------------------------------------------------
AM1880    NKYMLEKWNKEEWFHKLKKDWEDEKNRYDEKLFLWKNTENDKYINPMLERQKYIWRKWIAKHLYHIDEWENEEWFKLLMSKYEKDRDDLK
AM1802    NKYMLEKWNKEEWFHKLKKDWEDEKNRYDEKLFLWKNTENDKYINPMLERQKYIWRKWIAKHLYHIDEWENEEWFKLLMSKYEKDRDDLK
AM1803    NKYMLEKWNKEEWFCKLKKDWEDEKNRYDEKLFLCKNTENDKYINPMLERQKYIWRKWIAKHLYHIDEWENEEWFKLLMSKYEKDRDDLK
AM1811    NKYMLEKWNKEEWFCKLKKDWEDEKNRYDEKLFLCKNTENDKYINPMLERQKYIWRKWIAKHLYHIDEWENEEWFKLLMSKYEKDRDDLK
AM1814    NKYMLEKWNKEEWFHKLKKDWEDEKNRYDEKLFLWKNTENDKYINPMLERQKYIWRKWIAKHLYHIDEWENEEWFKLLMSKYEKDRDDLK
SC50      NKYMLEKWNKEEWFHKLKKDWEDEKNRYDEKLFLWKNTENDKYINPMLERQKYIWRKWIAKHLYHIDEWENEEWFKLLMSKYEKDRDDLK
SC56      NKYMLEKWNKEEWFCKLKKDWEDEKNRYDEKLFLCKNTENDKYINPMLERQKYIWRKWIAKHLYHIDEWENEEWFKLLMSKYEKDRDDLK
TAB153    NKYMLEKWNKEEWFCKLKKDWEDEKNRYDEKLFLCKNTENDKYINPMLERQKYIWRKWIAKHLYHIDEWENEEWFKLLMSKYEKDRDDLK
TAB154    NKYMLEKWNKEEWFCKLKKDWEDEKNRYDEKLFLWKNTENDKYINPMLERQKYIWRKWIAKHLYHIDEWENEEWFKLLMSKYEKDRDDLK
TAB156    NKYMLEKWNKEEWFHKLKKDWEDEKNRYDEKLFLWKNTENDKYINPMLERQKYIWRKWIAKHLYHIDEWENEEWFKLLMSKYEKDRDDLK
TAB136    ------------------------------------------------------------------------------------------
7G8       NKYMLEKWNKEEWFHKLKKDWEDEKNRYDEKLFLCKNTENDKYINPMLERQKYIWRKWIAKHLYHIDEWENEEWFKLLMSKYEKDRDDLK
IT        NKYMLEKWNKEEWFHKLKKDWEDEKNRYDEKLFLWKNTENDKYINPMLERQKYIWRKWIAKHLYHIDEWENEEWFKLLMSKYEKDRDDLK
HB3       NKYMLEKWNKEEWFCKLKKDWEDEKNRYDEKLFLCKNTENDKYINPMLERQKYIWRKWIAKHLYHIDEWENEEWFKLLMSKYEKDRDDLK
SD01      NKYMLEKWNKEEWFCKLKKDWEDEKNRYDEKLFLCKNTENDKYINPMLERQKYIWRKWIAKHLYHIDEWENEEWFKLLMSKYEKDRDDLK
FCR3      NKYMLEKWNKEEWFHKLKKDWEDEKNRYDEKLFLWKNTENDKYINPMLERQKYIWRKWIAKHLYHIDEWENEEWFKLLMSKYEKDRDDLK
CD01      ------------------------------------------------------------------------------------------
3D7       ------------------------------------------------------------------------------------------
MS822     ------------------------------------------------------------------------------------------
KH01      ------------------------------------------------------------------------------------------
Dd2       ------------------------------------------------------------------------------------------
GA01      ------------------------------------------------------------------------------------------
SN01      ------------------------------------------------------------------------------------------
GN01      ------------------------------------------------------------------------------------------
KE01      ------------------------------------------------------------------------------------------
KH02      ------------------------------------------------------------------------------------------
GB4       ------------------------------------------------------------------------------------------
```

```
                   1450      1460      1470      1480      1490      1500      1510      1520      1530
          ....|....|....|....|....|....|....|....|....|....|....|....|....|....|....|....|....|....|
TAB130    ------------------------------------------------------------------------------------------
TAB131    ------------------------------------------------------------------------------------------
TAB151    ------------------------------------------------------------------------------------------
TAB152    ------------------------------------------------------------------------------------------
TAB138    ------------------------------------------------------------------------------------------
TAB123    ------------------------------------------------------------------------------------------
AA264     ------------------------------------------------------------------------------------------
SC52      ------------------------------------------------------------------------------------------
SC53      ------------------------------------------------------------------------------------------
SC03      ------------------------------------------------------------------------------------------
AM1737    ------------------------------------------------------------------------------------------
AA235     ------------------------------------------------------------------------------------------
TAB141    ------------------------------------------------------------------------------------------
TAB166    ------------------------------------------------------------------------------------------
AA243     ------------------------------------------------------------------------------------------
AA258     ------------------------------------------------------------------------------------------
SC74      ------------------------------------------------------------------------------------------
AM1898    ------------------------------------------------------------------------------------------
SC55      ------------------------------------------------------------------------------------------
AM1880    RDHPINKMDDMEKKKLITKLFIEIHMMVIENSKEEECYRNKKNFVQTYIDELKKEQNLEQNKYMINILNDIQNDIQFGHDHNNYNEWKQE
AM1802    RDHPINKMDDMEKKKLITKLFIEIHMMVIENSKEEECYRNKKNFVQTYIDELKKEQNLEQNKYMINILNDIQNDIQFGHDHNNYNEWKQE
AM1803    RDHPINKMDDMEKKKLITKLFIEIHMMVIENSKEEECYRNKKNFVQTYIDELKKEQNLEQNKYMINILNDIQNDIQFGHDHNNYNEWKQE
AM1811    RDHPINKMDDMEKKKLITKLFIEIHMMVIENSKEEECYRNKKNFVQTYIDELKKEQNLEQNKYMINILNDIQNDIQFGHDHNNYNEWKQE
AM1814    RDHPINKMDDMEKKKLITKLFIEIHMMVIENSKEEECYRNKKNFVQTYIDELKKEQNLEQNKYMINILNDIQNDIQFGHDHNNYNEWKQE
SC50      RDHPINKMDDMEKKKLITKLFIEIHMMVIENSKEEECYRNKKNFVQTYIDELKKEQNLEQNKYMINILNDIQNDIQFGHDHNNYNEWKQE
SC56      RDHPINKMDDMEKKKLITKLFIEIHMMVIENSKEEECYRNKKNFVQTYIDELKKEQNLEQNKYMINILNDIQNDIQFGHDHNNYNEWKQE
TAB153    RDHPINKMDDMEKKKLITKLFIEIHMMVIENSKEEECYRNKKNFVQTYIDELKKEQNLEQNKYMINILNDIQNDIQFGHDHNNYNEWKQE
TAB154    RDHPINKMDDMEKKKLITKLFIEIHMMVIENSKEEECYRNKKNFVQTYIDELKKEQNLEQNKYMINILNDIQNDIQFGHDHNNYNEWKQE
TAB156    RDHPINKMDDMEKKKLITKLFIEIHMMVIENSKEEECYRNKKNFVQTYIDELKKEQNLEQNKYMINILNDIQNDIQFGHDHNNYNEWKQE
TAB136    ------------------------------------------------------------------------------------------
7G8       RDHPINKMDDMEKKKLITKLFIEIHMMVIENSKEEECYRNKKNFVQTYIDELKKEQNLEQNKYMINILNDIQNDIQFGHDHNNYNEWKQE
IT        RDHPINKMDDMEKKKLITKLFIEIHMMVIENSKEEECYRNKKNFVQTYIDELKKEQNLEQNKYMINILNDIQNDIQFGHDHNNYNEWKQE
HB3       RDHPINKMDDMEKKKLITKLFIEIHMMVIENSKEEECYRNKKNFVQTYIDELKKEQNLEQNKYMINILNDIQNDIQFGHDHNNYNEWKQE
SD01      RDHPINKMDDMEKKKLITKLFIEIHMMVIENSKEEECYRNKKNFVQTYIDELKKEQNLEQNKYMINILNDIQNDIQFGHDHNNYNEWKQE
FCR3      RDHPINKMDDMEKKKLITKLFIEIHMMVIENSKEEECYRNKKKFCSNIYR----------------------------------------
CD01      ------------------------------------------------------------------------------------------
3D7       ------------------------------------------------------------------------------------------
MS822     ------------------------------------------------------------------------------------------
KH01      ------------------------------------------------------------------------------------------
Dd2       ------------------------------------------------------------------------------------------
GA01      ------------------------------------------------------------------------------------------
SN01      ------------------------------------------------------------------------------------------
GN01      ------------------------------------------------------------------------------------------
KE01      ------------------------------------------------------------------------------------------
KH02      ------------------------------------------------------------------------------------------
GB4       ------------------------------------------------------------------------------------------
```

```
                1540      1550      1560      1570      1580      1590      1600      1610      1620
         ....|....|....|....|....|....|....|....|....|....|....|....|....|....|....|....|....|....|
TAB130   ------------------------------------------------------------------------------------------
TAB131   ------------------------------------------------------------------------------------------
TAB151   ------------------------------------------------------------------------------------------
TAB152   ------------------------------------------------------------------------------------------
TAB138   ------------------------------------------------------------------------------------------
TAB123   ------------------------------------------------------------------------------------------
AA264    ------------------------------------------------------------------------------------------
SC52     ------------------------------------------------------------------------------------------
SC53     ------------------------------------------------------------------------------------------
SC03     ------------------------------------------------------------------------------------------
AM1737   ------------------------------------------------------------------------------------------
AA235    ------------------------------------------------------------------------------------------
TAB141   ------------------------------------------------------------------------------------------
TAB166   ------------------------------------------------------------------------------------------
AA243    ------------------------------------------------------------------------------------------
AA258    ------------------------------------------------------------------------------------------
SC74     ------------------------------------------------------------------------------------------
AM1898   ------------------------------------------------------------------------------------------
SC55     ------------------------------------------------------------------------------------------
AM1880   KWFKNLKKEWKEGERKNFLHVENENLDNIQANKLNNYILEIQKAILKNYWEDMQIKWIDDDNKTDWLKIAMNLNNYDNNNFRKNIKYEQK
AM1802   KWFKNLKKEWKEGERKNFLHVENENLDNIQANKLNNYILEIQKAILKNYWEDMQIKWIDDDNKTDWLKIAMNLNNYDNNNFRKNIKYEQK
AM1803   KWFKNLKKEWKEGERKNFLHIENENLDNIQVNKFNNYILEIQKAILKNYWEDMQIKWIDDDNKTDWLKIAMNLNNYDNNNFRKNIKYEQK
AM1811   KWFKNLKKEWKEGERKNFLHIENENLDNIQVNKFNNYILEIQKAILKNYWEDMEIKWIDDDNKTDWLKIAMNLNNYDNNNFRKNIKYEQK
AM1814   KWFKNLKKEWKEGERKNFLHVENENLDNIQANKLNNYILEIQKAILKNYWEDMQIKWIDDDNKTDWLKIAMNLNNYDNNNFRKNIKYEQK
SC50     KWFKNLKKEWKEGERKNFLHIENENLDNIQVNKFNNYILEIQKAILKNYWEDMEIKWIDDDNKTDWLKIAMNLNNYDNNNFRKNIKYEQK
SC56     KWFKNLKKEWKEGERKNFLHIENENLDNIQVNKFNNYILEIQKAILKNYWEDMEIKWIDDDNKTDWLKIAMNLNNYDNNNFRKNIKYEQK
TAB153   KWFKNLKKEWKEGERKNFLHIENENLDNIQVNKFNNYILEIQKAILKNYWEDMEIKWIDDDNKTDWLKIAMNLNNYDNNNFRKNIKYEQK
TAB154   KWFKNLKKEWKEGERKNFLHIENENLDNIQVNKFNNYILEIQKAILKNYWEDMQIKWIDDDNKTDWLKIAMNLNNYDNNNFRKNIKYEQK
TAB156   KWFKNLKKEWKEGERKNFLHVENENLDNIQANKLNNYILEIQKAILKNYWEDMQIKWIDDDNKTDWLKIAMNLNNYDNNNFRKNIKYEQK
TAB136   ------------------------------------------------------------------------------------------
7G8      KWFKNLKKEWKEGERKNFLHIENENLDNIQVNKFNNYILEIQKAILKNYWEDMQIKWIDDDNKTDWLKIAMNLNNYDNNNFRKNIKYEQK
IT       KWFKNLKKEWKEGERKNFLHVENENLDNIQANKLNNYILEIQKAILKNYWEDMQIKWIDDDNKTDWLKIAMNLNNYDNNNFRKNIKYEQK
HB3      KWFKNLKKEWKEGERKNFLHIENENLDNIQVNKFNNYILEIQKAILKNYWEDMEIKWIDDDNKTDWLKIAMNLNNYDNNNFRKNIKYEQK
SD01     KWFKNLKKEWKEGERKNFLHVENENLDNIQANKLNNYILEIQKAILKNYWEDMQIKWIDDDNKTDWLKIAMNLNNYDNNNFRKNIKYEQK
FCR3     ------------------------------------------------------------------------------------------
CD01     ------------------------------------------------------------------------------------------
3D7      ------------------------------------------------------------------------------------------
MS822    ------------------------------------------------------------------------------------------
KH01     ------------------------------------------------------------------------------------------
Dd2      ------------------------------------------------------------------------------------------
GA01     ------------------------------------------------------------------------------------------
SN01     ------------------------------------------------------------------------------------------
GN01     ------------------------------------------------------------------------------------------
KE01     ------------------------------------------------------------------------------------------
KH02     ------------------------------------------------------------------------------------------
GB4      ------------------------------------------------------------------------------------------
```

```
                 1630      1640      1650      1660      1670      1680      1690      1700      1710
         ....|....|....|....|....|....|....|....|....|....|....|....|....|....|....|....|....|....|
TAB130   ------------------------------------------------------------------------------------------
TAB131   ------------------------------------------------------------------------------------------
TAB151   ------------------------------------------------------------------------------------------
TAB152   ------------------------------------------------------------------------------------------
TAB138   ------------------------------------------------------------------------------------------
TAB123   ------------------------------------------------------------------------------------------
AA264    ------------------------------------------------------------------------------------------
SC52     ------------------------------------------------------------------------------------------
SC53     ------------------------------------------------------------------------------------------
SC03     ------------------------------------------------------------------------------------------
AM1737   ------------------------------------------------------------------------------------------
AA235    ------------------------------------------------------------------------------------------
TAB141   ------------------------------------------------------------------------------------------
TAB166   ------------------------------------------------------------------------------------------
AA243    ------------------------------------------------------------------------------------------
AA258    ------------------------------------------------------------------------------------------
SC74     ------------------------------------------------------------------------------------------
AM1898   ------------------------------------------------------------------------------------------
SC55     ------------------------------------------------------------------------------------------
AM1880   KNNFKEEIEMIKKINKKAQSKDTEEDIMWKTVIEIHMKIIEEDKKQEWEKNKGDFLQICRQEFLNENQYNRNENSRFVNDYMVQDNYMIK
AM1802   KNNFKEEIEMIKKINKKAQSKDTEEDIMWKTVIEIHMKIIEEDKKQEWEKNKGDFLQICRQEFLNENQYNRNENSRFVNDYMVQDNYMIK
AM1803   KNNFKEEIEMIKKINKKAQSKDTEKDIMWKTVIEIHMKIIEEDKKQEWEKNKGDFLQICRQEFLNENQYNRNENSRFVNDYMVQDNYMIK
AM1811   KNNFKEEIEMIKKINKKAQSKDTEKDIMWKTVIEIHMKIIEEDKKQEWEKNKGDFLQICRQEFLNENQYNRNENSRFVNDYMVQDNYMIK
AM1814   KNNFKEEIEMIKKINKKAQSKDTEEDIMWKTVIEIHMKIIEEDKKQEWEKNKGDFLQICRQEFLNENQYNRNENSRFVNDYMVQDNYMIK
SC50     KNNFKEEIEMIKKINKKAQSKDTEKDIMWKTVIEIHMKIIEEDKKQEWEKNKGDFLQICRQEFLNENQYNRNENSRFVNDYMVQDNYMIK
SC56     KNNFKEEIEMIKKINKKAQSKDTEKDIMWKTVIEIHMKIIEEDKKQEWEKNKGDFLQICRQEFLNENQYNRNENSRFVNDYMVQDNYMIK
TAB153   KNNFKEEIEMIKKINKKAQSKDTEKDIMWKTVIEIHMKIIEEDKKQEWEKNKGDFLQICRQEFLNENQYNRNENSRFVNDYMVQDNYMIK
TAB154   KNNFKEEIEMIKKINKKAQSKDTEEDIMWKTVIEIHMKIIEEDKKQEWEKNKGDFLQICRQEFLNENQYNRNENSRFVNDYMVQDNYMIK
TAB156   KNNFKEEIEMIKKINKKAQSKDTEEDIMWKTVIEIHMKIIEEDKKQEWEKNKGDFLQICRQEFLNENQYNRNENSRFVNDYMVQDNYMIK
TAB136   ------------------------------------------------------------------------------------------
7G8      KNNFKEEIEMIKKINKKAQSKDTEKDIMWKTVIEIHMKIIEEDKKQEWEKNKGDFLQICRQEFLNENQYNRNENSRFVNDYMVQDNYMIK
IT       KNNFKEEIEMIKKINKKAQSKDTEEDIMWKTVIEIHMKIIEEDKKQEWEKNKGDFLQICRQEFLNENQYNRNENSRFVNDYMVQDNYMIK
HB3      KNNFKEEIEMIKKINKKAQSKDTEKDIMWKTVIEIHMKIIEEDKKQEWEKNKGDFLQICRQEFLNENQYNRNENSRFVNDYMVQDNYMIK
SD01     KNNFKEEIEMIKKINKKAQSKDTEKDIMWKTVIEIHMKIIEEDKKQEWEKNKGDFLQICRQEFLNENQYNRNENSRFVNDYMVQDNYMIK
FCR3     ------------------------------------------------------------------------------------------
CD01     ------------------------------------------------------------------------------------------
3D7      ------------------------------------------------------------------------------------------
MS822    ------------------------------------------------------------------------------------------
KH01     ------------------------------------------------------------------------------------------
Dd2      ------------------------------------------------------------------------------------------
GA01     ------------------------------------------------------------------------------------------
SN01     ------------------------------------------------------------------------------------------
GN01     ------------------------------------------------------------------------------------------
KE01     ------------------------------------------------------------------------------------------
KH02     ------------------------------------------------------------------------------------------
GB4      ------------------------------------------------------------------------------------------
```

```
                    1720      1730      1740      1750      1760      1770      1780      1790      1800
          ....|....|....|....|....|....|....|....|....|....|....|....|....|....|....|....|....|....|
TAB130    ------------------------------------------------------------------------------------------
TAB131    ------------------------------------------------------------------------------------------
TAB151    ------------------------------------------------------------------------------------------
TAB152    ------------------------------------------------------------------------------------------
TAB138    ------------------------------------------------------------------------------------------
TAB123    ------------------------------------------------------------------------------------------
AA264     ------------------------------------------------------------------------------------------
SC52      ------------------------------------------------------------------------------------------
SC53      ------------------------------------------------------------------------------------------
SC03      ------------------------------------------------------------------------------------------
AM1737    ------------------------------------------------------------------------------------------
AA235     ------------------------------------------------------------------------------------------
TAB141    ------------------------------------------------------------------------------------------
TAB166    ------------------------------------------------------------------------------------------
AA243     ------------------------------------------------------------------------------------------
AA258     ------------------------------------------------------------------------------------------
SC74      ------------------------------------------------------------------------------------------
AM1898    ------------------------------------------------------------------------------------------
SC55      ------------------------------------------------------------------------------------------
AM1880    RQNGIWNKCIERNRYILEKWKKEKWFEKLKNQWKNEQNIYVNTREVYSNINDETNVKEINPLIEGEKVLWKKWLRNQKGLLDKYNEEFWF
AM1802    RQNGIWNKCIERNRYILEKWKKEKWFEKLKNEWKNEQNIYVNTREVYSNINDETNVKEINPLIEGEKVLWKKWLRNQKGLLDKYNEEFWF
AM1803    RQNGIWNKCIERNRYILEKWKKEKWFEKLKNQWKNEQNIYVNTREVYSNINDETNVKEINPLIEGEKVLWKKWLRNQKGLLDKYNEEFWF
AM1811    RQNGIWNKCIERNRYILEKWKKEKWFEKLKNQWKNEQNIYVNTREVYSNINDETNVKEINPLIEGEKVLWKKWLRNQKGLLDKYNEEFWF
AM1814    RQNGIWNKCIERNRYILEKWKKEKWFEKLKNQWKNEQNIYVNTREVYSNINDETNVKEINPLIEGEKVLWKKWLRNQKGLLDKYNEEFWF
SC50      RQNGIWNKCIERNRYILEKWKKEKWFEKLKNQWKNEQNIYVNTREVYSNINDETNVKEINPLIEGEKVLWKKWLRNQKGLLDKYNEEFWF
SC56      RQNGIWNKCIERNRYILEKWKKEKWFEKLKNQWKNEQNIYVNTREVYSNINDETNVKEINPLIEGEKVLWKKWLRNQKGLLDKYNEEFWF
TAB153    RQNGIWNKCIERNRYILEKWKKEKWFEKLKNQWKNEQNIYVNTREVYSNINDETNVKEINPLIEGEKVLWKKWLRNQKGLLDKYNEEFWF
TAB154    RQNGIWNKCIERNRYILEKWKKEKWFEKLKNQWKNEQNIYVNTREVYSNINDETNVKEINPLIEGEKVLWKKWLRNQKGLLDKYNEEFWF
TAB156    RQNGIWNKCIERNRYILEKWKKEKWFEKLKNEWKNEQNIYVNTREVYSNINDETNVKEINPLIEGEKVLWKKWLRNQKGLLDKYNEEFWF
TAB136    ------------------------------------------------------------------------------------------
7G8       RQNGIWNKCIERNRYILEKWKKEKWFEKLKNQWKNEQNIYVNTREVYSNINDETNVKEINPLIEGEKVLWKKWLRNQKGLLDKYNEEFWF
IT        RQNGIWNKCIERNRYILEKWKKEKWFEKLKNQWKNEQNIYVNTREVYSNINDETNVKEINPLIEGEKVLWKKWLRNQKGLLDKYNEEFWF
HB3       RQNGIWNKCIERNRYILEKWKKEKWFEKLKNEWKNEQNIYVNTREVYSNINDETNVKEINPLIEGEKVLWKKWLRNRKGLLEKYNEEFWF
SD01      RQNGIWNKCIERNRYILEKWKKEKWFEKLKNQWKNEQNIYVNTREVYSNINDETNVKEINPLIEGEKVLWKKWLRNRKGLLDKYNEEFWF
FCR3      ------------------------------------------------------------------------------------------
CD01      ------------------------------------------------------------------------------------------
3D7       ------------------------------------------------------------------------------------------
MS822     ------------------------------------------------------------------------------------------
KH01      ------------------------------------------------------------------------------------------
Dd2       ------------------------------------------------------------------------------------------
GA01      ------------------------------------------------------------------------------------------
SN01      ------------------------------------------------------------------------------------------
GN01      ------------------------------------------------------------------------------------------
KE01      ------------------------------------------------------------------------------------------
KH02      ------------------------------------------------------------------------------------------
GB4       ------------------------------------------------------------------------------------------
```

```
                1810      1820      1830      1840      1850      1860      1870      1880      1890
        ....|....|....|....|....|....|....|....|....|....|....|....|....|....|....|....|....|....|
TAB130  ------------------------------------------------------------------------------------------
TAB131  ------------------------------------------------------------------------------------------
TAB151  ------------------------------------------------------------------------------------------
TAB152  ------------------------------------------------------------------------------------------
TAB138  ------------------------------------------------------------------------------------------
TAB123  ------------------------------------------------------------------------------------------
AA264   ------------------------------------------------------------------------------------------
SC52    ------------------------------------------------------------------------------------------
SC53    ------------------------------------------------------------------------------------------
SC03    ------------------------------------------------------------------------------------------
AM1737  ------------------------------------------------------------------------------------------
AA235   ------------------------------------------------------------------------------------------
TAB141  ------------------------------------------------------------------------------------------
TAB166  ------------------------------------------------------------------------------------------
AA243   ------------------------------------------------------------------------------------------
AA258   ------------------------------------------------------------------------------------------
SC74    ------------------------------------------------------------------------------------------
AM1898  ------------------------------------------------------------------------------------------
SC55    ------------------------------------------------------------------------------------------
AM1880  KKLFEDYEKEVENDDDDD---YYYYHMSVNQQGKHPEDLITLNKISNQSLQKFKKNKLITTMWIEIHMMILEECKEEEVQLNKELFLDSC
AM1802  KKLVEDYEKEVENDDDDDD--YYYYHMSVNQQGKHPEDLITLNKISNQSLQKFKKNKLITTMWIEIHMMILEECKEEEVQLNKELFLDSC
AM1803  KKLFEDYEKEVENDDDDD---YYYYHMSVNQQGKHPEDLITLNKISNQSLQKFKKNKLITTMWIEIHMMILEECKEEEVQLNKELFLDSC
AM1811  KKLFEDYEKEVENDDDDD---YYYYHMSVNQQGKHPEDLITLNKISNQSLQKFKKNKLITTMWIEIHMMILEECKEEEVQLNKELFLDSC
AM1814  KKLFEDYEKEVENDDDDD---YYYYHMSVNQQGKHPEDLITLNKISNQSLQKFKKNKLITTMWIEIHMMILEECKEEEVQLNKELFLDSC
SC50    KKLFEDYEKEVENDDDDD---YYYYHMSVNQQGKHPEDLITLNKISNQSLQKFKKNKLITTMWIEIHMMILEECKEEEVQLNKELFLDSC
SC56    KKLFEDYEKEVENDDDDD---YYYYHMSVNQQGKHPEDLITLNKISNQSLQKFKKNKLITTMWIEIHMMILEECKEEEVQLNKELFLDSC
TAB153  KKLFEDYEKEVENDDDDD---YYYYHMSVNQQGKHPEDLITLNKISNQSLQKFKKNKLITTMWIEIHMMILEECKEEEVQLNKELFLDSC
TAB154  KKLFEDYEKEVENDDDDD---YYYYHMSVNQQGKHPEDLITLNKISNQSLQKFKKNKLITTMWIEIHMMILEECKEEEVQLNKELFLDSC
TAB156  KKLVEDYEKEVENDDDDDD--YYYYHMSVNQQGKHPEDLITLNKISNQSLQKFKKNKLITTMWIEIHMMILEECKEEEVQLNKELFLDSC
TAB136  ------------------------------------------------------------------------------------------
7G8     KKLFEDYEKEVENDDDDD---YYYYHMSVNQQGKHPEDLITLNKISNQSLQKFKKNKLITTMWIEIHMMILEECKEEEVQLNKELFLDFC
IT      KKLFEDYEKEVENDDDDD---YYYYHMSVNQQGKHPEDLITLNKISNQSLQKFKKNKLITTMWIEIHMMILEECKEEEVQLNKELFLDSC
HB3     KKLFEDYEKEVENDDDDD---YYYYHMSVNQQGKHPEDLITLNKISNQSLQKFKKNKLITTMWIEIHMMILEECKEEEVQLNKELFLDSC
SD01    KKLVEDYEKEVENDDEDDDDNDDYYLMSVNQQGKHPEDLITLNKISNQSLQKFKKNKLITTMWIEIHMMILEECKEEEVQLNKELFLDSC
FCR3    ------------------------------------------------------------------------------------------
CD01    ------------------------------------------------------------------------------------------
3D7     ------------------------------------------------------------------------------------------
MS822   ------------------------------------------------------------------------------------------
KH01    ------------------------------------------------------------------------------------------
Dd2     ------------------------------------------------------------------------------------------
GA01    ------------------------------------------------------------------------------------------
SN01    ------------------------------------------------------------------------------------------
GN01    ------------------------------------------------------------------------------------------
KE01    ------------------------------------------------------------------------------------------
KH02    ------------------------------------------------------------------------------------------
GB4     ------------------------------------------------------------------------------------------
```

```
                  1900      1910      1920      1930      1940      1950      1960      1970      1980
          ....|....|....|....|....|....|....|....|....|....|....|....|....|....|....|....|....|....|
TAB130    ------------------------------------------------------------------------------------------
TAB131    ------------------------------------------------------------------------------------------
TAB151    ------------------------------------------------------------------------------------------
TAB152    ------------------------------------------------------------------------------------------
TAB138    ------------------------------------------------------------------------------------------
TAB123    ------------------------------------------------------------------------------------------
AA264     ------------------------------------------------------------------------------------------
SC52      ------------------------------------------------------------------------------------------
SC53      ------------------------------------------------------------------------------------------
SC03      ------------------------------------------------------------------------------------------
AM1737    ------------------------------------------------------------------------------------------
AA235     ------------------------------------------------------------------------------------------
TAB141    ------------------------------------------------------------------------------------------
TAB166    ------------------------------------------------------------------------------------------
AA243     ------------------------------------------------------------------------------------------
AA258     ------------------------------------------------------------------------------------------
SC74      ------------------------------------------------------------------------------------------
AM1898    ------------------------------------------------------------------------------------------
SC55      ------------------------------------------------------------------------------------------
AM1880    IKELIKEKESKGKCKMLEIVLDLKDKHVLINKNLEMNKRIGKNSNNLFENIKTQVKNNENNYIYEMMLNNDMNKSGKMIENDCICDEMLI
AM1802    IKELIKEKESKGKCKMLEIVLDLKDKHVLINKNLEMNKRIGKNSNNLFENIKTQVKNNENNYIYEMMLNNDMNKSGKMIENDCICDEMLI
AM1803    IKELIKEKESKGKCKMLEIVLDLKDKHVLINKNLEMNKRIGKNSNNLFENIKTQVKNNENNYIYEMMLNNDMNKSGKMSENDCIFDEMLI
AM1811    IKELIKEKESKGKCKMLEIVLDLKDKHVLINKNLEMNKRIGKNSNNLFENIKTQVKNNENNYIYEMMLNNDMNKSGKMSENDCIFDEMLI
AM1814    IKELIKEKESKGKCKMLEIVLDLKDKHVLINKNLEMNKRIGKNSNNLFENIKTQVKNNENNYIYEMMLNNDMNKSGKMIENDCICDEMLI
SC50      IKELIKEKESKGKCKMLEIVLDLKDKHVLINKNLEMNKRIGKNSNNLFENIKTQVKNNENNYIYEMMLNNDMNKSGKMSENDCIFDEMLI
SC56      IKELIKEKESKGKCKMLEIVLDLKDKHVLINKNLEMNKRIGKNSNNLFENIKTQVKNNENNYIYEMMLNNDMNKSGKMSENDCIFDEMLI
TAB153    IKELIKEKESKGKCKMLEIVLDLKDKHVLINKNLEMNKRIGKNSNNLFENVKTQVKNNENNYIYEMMLNNDMNKSGKMSENDCIFDEMLI
TAB154    IKELIKEKESKGKCKMLEIVLDLKDKHVLINKNLEMNKRIGKNSNNLFENIKTQVKNNENNYIYEMMLNNDMNKSGKMSENDCIFDEMLI
TAB156    IKELIKEKESKGKCKMLEIVLDLKDKHVLINKNLEMNKRIGKNSNNLFENIKTQVKNNENNYIYEMMLNNDMNKSGKMIENDCICDEMLI
TAB136    ------------------------------------------------------------------------------------------
7G8       IKELIKEKESKGKCKMLEIVLDLKDKHVLINKNLEMNKRIGKNSNNLFENIKTQVKNNENNYIYEMMLNNDMNKSGKMSENDCIFDEMLI
IT        IKELIKEKESKGKCKMLEIVLDLKDKHVLINKNLEMNKRIGKNSNNLFENIKTQVKNNENNYIYEMMLNNDMNKSGKMSENDCIFDEMLI
HB3       IKELIKEKESKGKCKMLEIVLDLKDKHVLINKNLEMNKRIGKNSNNLFENIKTQVKNNENNYIYEMMLNNDMNKSGKMIENDCICDEMLI
SD01      IKELIKEKESKGKCKMLEIVLDLKDKHVLINKNLEMNKRIGKNSNNLFENIKTQVKNNENNYIYEMMLNNDMNKSGKMIENDCICDEMLI
FCR3      ------------------------------------------------------------------------------------------
CD01      ------------------------------------------------------------------------------------------
3D7       ------------------------------------------------------------------------------------------
MS822     ------------------------------------------------------------------------------------------
KH01      ------------------------------------------------------------------------------------------
Dd2       ------------------------------------------------------------------------------------------
GA01      ------------------------------------------------------------------------------------------
SN01      ------------------------------------------------------------------------------------------
GN01      ------------------------------------------------------------------------------------------
KE01      ------------------------------------------------------------------------------------------
KH02      ------------------------------------------------------------------------------------------
GB4       ------------------------------------------------------------------------------------------
```

```
                  1990      2000      2010      2020      2030      2040      2050      2060      2070
         ....|....|....|....|....|....|....|....|....|....|....|....|....|....|....|....|....|....|
TAB130   ------------------------------------------------------------------------------------------
TAB131   ------------------------------------------------------------------------------------------
TAB151   ------------------------------------------------------------------------------------------
TAB152   ------------------------------------------------------------------------------------------
TAB138   ------------------------------------------------------------------------------------------
TAB123   ------------------------------------------------------------------------------------------
AA264    ------------------------------------------------------------------------------------------
SC52     ------------------------------------------------------------------------------------------
SC53     ------------------------------------------------------------------------------------------
SC03     ------------------------------------------------------------------------------------------
AM1737   ------------------------------------------------------------------------------------------
AA235    ------------------------------------------------------------------------------------------
TAB141   ------------------------------------------------------------------------------------------
TAB166   ------------------------------------------------------------------------------------------
AA243    ------------------------------------------------------------------------------------------
AA258    ------------------------------------------------------------------------------------------
SC74     ------------------------------------------------------------------------------------------
AM1898   ------------------------------------------------------------------------------------------
SC55     ------------------------------------------------------------------------------------------
AM1880   NNMNKSDRIGQKNDNTIEDNLKSNMNKLGDVNEKCILEMEKNISMRHWENIKKNCTDGKEKEKNIKNVEMIENI---GSTGKIENIKNIR
AM1802   NNMNKSDRIGQKNDNTIEDNLKSNMNKLGDVNEKCILEMEKNISMRHWENIKKNCTDGKEKEKNIKNVEMIENIENIGSTGKIENIKNIR
AM1803   NNMNKSDRIGQKKDNTIEDNLKSNMNKLGDVNEKCILEMEKNISMRHWENIKKNCTDGKEKEKNIKNVEMIENIENIGSTGKIENIR---
AM1811   NNMNKSDRIGQKKDNTIEDNLKSNMNKLGDVNEKCILEMEKNISMRHWENIKKNCTDGKEKEKNIKNVEMIENIENIGSTGKIENIR---
AM1814   NNMNKSDRIGQKNDNTIEDNLKSNMNKLGDVNEKCILEMEKNISMRHWENIKKNCTDGKEKEKNIKNVEMIENIENIGSTGKIENIKNIR
SC50     NNMNKSDRIGQKKDNTIEDNLKSNMNKLGDVNEKCILEMEKNISMRHWENIKKNCTDGKEKEKNIKNVEMIENIENIGSTGKIENIR---
SC56     NNMNKSDRIGQKKDNTIEDNLKSNMNKLGDVNEKCILEMEKNISMRHWENIKKNCTDGKEKEKNIKNVEMIENIENIGSTGKIENIR---
TAB153   NNMNKSDRIGQKKDNTIEDNLKSNMNKLGDVNEKCILEMEKNISMRHWENIKKNCTDGKEKEKNIKNVEMIENIENIGSTGKIENIR---
TAB154   NNMNKSDRIGQKKDNTIEDNLKSNMNKLGDVNEKCILEMEKNISMRHWENIKKNCTDGKEKEKNIKNVEMIENIENIGSTGKIENIKNIR
TAB156   NNMNKSDRIGQKNDNTIEDNLKSNMNKLGDVNEKCILEMEKNISMRHWENIKKNCTDGKEKEKNIKNVEMIENIENIGSTGKIENIKNIR
TAB136   ------------------------------------------------------------------------------------------
7G8      NNMNKSDRIGQKKDNTIEDNLKSNMNKLGDVNEKCILEMEKNISMRHWENIKKNCTDGKEKEKNIKNVEMIENIENIGSTGKIE---NIR
IT       NNMNKSDRIGQKKDNTIEDNLKSNMNKLGDVNEKCILEMEKNISMRHWENIKKNCTDGKEKEKNIKNVEMIENI---GSTGKIENIKNIR
HB3      NNMNKSDRIGQKNDNTIEDNLKSNMNKLGDVNEKCILEMEKNISMRHWENIKKNCTDGKEKEKNIKNVEMIENI---GSTGKIENIKNIR
SD01     NNMNKSDRIGQKNDNTIEDNLKSNMNKLGDVNEKCILEMEKNISMRHWENIKKNCTDGKEKEKNIKNVEMIENI---GSTGKIENIKNIR
FCR3     ------------------------------------------------------------------------------------------
CD01     ------------------------------------------------------------------------------------------
3D7      ------------------------------------------------------------------------------------------
MS822    ------------------------------------------------------------------------------------------
KH01     ------------------------------------------------------------------------------------------
Dd2      ------------------------------------------------------------------------------------------
GA01     ------------------------------------------------------------------------------------------
SN01     ------------------------------------------------------------------------------------------
GN01     ------------------------------------------------------------------------------------------
KE01     ------------------------------------------------------------------------------------------
KH02     ------------------------------------------------------------------------------------------
GB4      ------------------------------------------------------------------------------------------
```

```
                 2080      2090      2100      2110      2120      2130      2140      2150      2160
         ....|....|....|....|....|....|....|....|....|....|....|....|....|....|....|....|....|....|
TAB130   ------------------------------------------------------------------------------------------
TAB131   ------------------------------------------------------------------------------------------
TAB151   ------------------------------------------------------------------------------------------
TAB152   ------------------------------------------------------------------------------------------
TAB138   ------------------------------------------------------------------------------------------
TAB123   ------------------------------------------------------------------------------------------
AA264    ------------------------------------------------------------------------------------------
SC52     ------------------------------------------------------------------------------------------
SC53     ------------------------------------------------------------------------------------------
SC03     ------------------------------------------------------------------------------------------
AM1737   ------------------------------------------------------------------------------------------
AA235    ------------------------------------------------------------------------------------------
TAB141   ------------------------------------------------------------------------------------------
TAB166   ------------------------------------------------------------------------------------------
AA243    ------------------------------------------------------------------------------------------
AA258    ------------------------------------------------------------------------------------------
SC74     ------------------------------------------------------------------------------------------
AM1898   ------------------------------------------------------------------------------------------
SC55     ------------------------------------------------------------------------------------------
AM1880   NIDEILEEKYKSFNIEPYEENNYEEKSKILNRSNIQSNKIISPLIDNEKGIDEKKSDNQNEEIWYNGLTLEEIYKNSYTRNSIEYIPYID
AM1802   NIDEILEEKYKSFNVEPYEENNYEEKSKILNRSNIQSNKIISPLIDNEKGIDEKKSDNQNEEIWYNGLTLEEIYKNSYTRNSIEYIPYID
AM1803   NIDEIVEEKYKSFNVEPYEENDYEEKSKILNRSNIQSNKIISPLIDNEKGIDEKKSDNQNEEIWYNGLTLEEIYKNSYTRNSIEYIPYID
AM1811   NIDEIVEEKYKSFNVEPYEENDYEEKSKILNRSNIQSNKIISPLIDNEKGIDEKKSDNQNEEIWYNGLTLEEIYKNSYTRNSIEYIPYID
AM1814   NIDEILEEKYKSFNIEPYEENNYEEKSKILNRSNIQSNKIISPLIDNEKGIDEKKSDNQNEEIWYNGLTLEEIYKNSYTRNSIEYIPYID
SC50     NIDEIVEEKYKSFNVEPYEENDYEEKSKILNRSNIQSNKIISPLIDNEKGIDEKKSDNQNEEIWYNGLTLEEIYKNSYTRNSIEYIPYID
SC56     NIDEIVEEKYKSFNVEPYEENDYEEKSKILNRSNIQSNKIISPLIDNEKGIDEKKSDNQNEEIWYNGLTLEEIYKNSYTRNSIEYIPYID
TAB153   NIDEIVEEKYKSFNVEPYEENDYEEKSKILNRSNIQSNKIISPLIDNEKGIDEKKSDNQNEEIWYNGLTLEEIYKNSYTRNSIEYIPYID
TAB154   NIDEILEEKYKSFNVEPYEENNYEEKSKILNRSNIQSNKIISPLIDNEKGIDEKKSDNQNEEIWYNGLTLEEIYKNSYTRNSIEYIPYID
TAB156   NIDEILEEKYKSFNVEPYEENNYEEKSKILNRSNIQSNKIISPLIDNEKGIDEKKSDNQNEEIWYNGLTLEEIYKNSYTRNSIEYIPYID
TAB136   ------------------------------------------------------------------------------------------
7G8      NIDEIVEEKYKSFNVEPYEENDYEEKSKILNRSNIQSNKIISPLIDNEKGIDEKKSDNQNEEIWYNGLTLEEIYKNSYTRNSIEYIPYID
IT       NIDEILEEKYKSFNIEPYEENNYEEKSKILNRSNIQSNKIISPLIDNEKGIDEKKSDNQNEEIWYNGLTLEEIYKNSYTRNSIEYIPYID
HB3      NIDEILEEKYKSFNIEPYEENNYEEKSKILNRSNIQSNKIISPLIDNEKGIDEKKSDNQNEEIWYNGLTLEEIYKNSYTRNSIEYIPYID
SD01     NIDEILEEKYKSFNIEPYEENNYEEKSKILNRSNIQSNKIISPLIDNEKGIDEKKSDNQNEEIWYNGLTLEEIYKNSYTRNSIEYIPYID
FCR3     ------------------------------------------------------------------------------------------
CD01     ------------------------------------------------------------------------------------------
3D7      ------------------------------------------------------------------------------------------
MS822    ------------------------------------------------------------------------------------------
KH01     ------------------------------------------------------------------------------------------
Dd2      ------------------------------------------------------------------------------------------
GA01     ------------------------------------------------------------------------------------------
SN01     ------------------------------------------------------------------------------------------
GN01     ------------------------------------------------------------------------------------------
KE01     ------------------------------------------------------------------------------------------
KH02     ------------------------------------------------------------------------------------------
GB4      ------------------------------------------------------------------------------------------
```

```
                2170      2180      2190      2200      2210      2220
        ....|....|....|....|....|....|....|....|....|....|....|....|....|...
TAB130  --------------------------------------------------------------------
TAB131  --------------------------------------------------------------------
TAB151  --------------------------------------------------------------------
TAB152  --------------------------------------------------------------------
TAB138  --------------------------------------------------------------------
TAB123  --------------------------------------------------------------------
AA264   --------------------------------------------------------------------
SC52    --------------------------------------------------------------------
SC53    --------------------------------------------------------------------
SC03    --------------------------------------------------------------------
AM1737  --------------------------------------------------------------------
AA235   --------------------------------------------------------------------
TAB141  --------------------------------------------------------------------
TAB166  --------------------------------------------------------------------
AA243   --------------------------------------------------------------------
AA258   --------------------------------------------------------------------
SC74    --------------------------------------------------------------------
AM1898  --------------------------------------------------------------------
SC55    --------------------------------------------------------------------
AM1880  EEEYSSLDELDQKNNDFESDDMFYKGLTLEEIYKNAYTRNSTEYIPYIDEEEYSSMDEFDKHNNKRKK
AM1802  EEEYSSLDELDQKNNDFESDDMFYKGLTLEEIYKNAYTRNSTEYIPYIDEEEYSSMDEFDKHNNKRKK
AM1803  EEEYSSLDELDQKNNDFESDDMFYKGLTLEEIYKNAYTRNSTEYIPYIDEEEYSSMDEFDKHNNKRKK
AM1811  EEEYSSLDELDQKNNDFESDDMFYKGLTLEEIYKNAYTRNSTEYIPYIDEEEYSSMDEFDKHNNKRKK
AM1814  EEEYSSLDELDQKNNDFESDDMFYKGLTLEEIYKNAYTRNSTEYIPYIDEEEYSSMDEFDKHNNKRKK
SC50    EEEYSSLDELDQKNNDFESDDMFYKGLTLEEIYKNAYTRNSTEYIPYIDEEEYSSMDEFDKHNNKRKK
SC56    EEEYSSLDELDQKNNDFESDDMFYKGLTLEEIYKNAYTRNSTEYIPYIDEEEYSSMDEFDKHNNKRKK
TAB153  EEEYSSLDELDQKNNDFESDDMFYKGLTLEEIYKNAYTRNSTEYIPYIDEEEYSSMDEFDKHNNKRKK
TAB154  EEEYSSLDELDQKNNDFESDDMFYKGLTLEEIYKNAYTRNSTEYIPYIDEEEYSSMDEFDKHNNKRKK
TAB156  EEEYSSLDELDQKNNDFESDDMFYKGLTLEEIYKNAYTRNSTEYIPYIDEEEYSSMDEFDKHNNKRKK
TAB136  --------------------------------------------------------------------
7G8     EEEYSSLDELDQKNNDFESDDMFYKGLTLEEIYKNAYTRNSTEYIPYIDEEEYSSMDEFDKHNNKRKK
IT      EEEYSSLDELDQKNNDFESDDMFYKGLTLEEIYKNAYTRNSTEYIPYIDEEEYSSMDEFDKHNNKRKK
HB3     EEEYSSLDELDQKNNDFESDDMFYKGLTLEEIYKNAYTRNSTEYIPYIDEEEYSSMDEFDKHNNKRKK
SD01    EEEYSSLDELDQKNNDFESDDMFYKGLTLEEIYKNAYTRNSTEYIPYIDEEEYSSMDEFDKHNNKRKK
FCR3    --------------------------------------------------------------------
CD01    --------------------------------------------------------------------
3D7     --------------------------------------------------------------------
MS822   --------------------------------------------------------------------
KH01    --------------------------------------------------------------------
Dd2     --------------------------------------------------------------------
GA01    --------------------------------------------------------------------
SN01    --------------------------------------------------------------------
GN01    --------------------------------------------------------------------
KE01    --------------------------------------------------------------------
KH02    --------------------------------------------------------------------
GB4     --------------------------------------------------------------------
```
