# Supplementary material for: Plasmodium falciparum surf4.1 in clinical isolates: From genetic variation and variant diversity to in silico design immunopeptides for vaccine development
Source: PLoS One. 2024 Dec 30;19(12):e0312091. doi: 10.1371/journal.pone.0312091 (PMC11684625; doi:10.1371/journal.pone.0312091)
Supplement: Attachment — Submitted filename: Response to Reviewers.docx [file pone.0312091.s008.docx]

Chalongkrung 1 Rd., Ladkrang

Bangkok 10520, Thailand

[noranate@gmail.com](mailto:noranate@gmail.com)

September 15, 2024

Dear Editors

We would like to thank the reviewers for their generous comments on the manuscript and have edited the manuscript to address their concerns.

We believe that the manuscript is now suitable for publication in PLOS ONE.

Kind regards,

Nitchakarn Noranate

On behalf of all authors.

PONE-D-24-13672

Plasmodium falciparum surf4.1 in clinical isolates: from genetic variation and variant diversity to in silico design immunopeptides for vaccine development

PLOS ONE

Dear Dr. Noranate,

Thank you for submitting your manuscript to PLOS ONE. After careful consideration, we feel that it has merit but does not fully meet PLOS ONE’s publication criteria as it currently stands. Therefore, we invite you to submit a revised version of the manuscript that addresses the points raised during the review process.

Please submit your revised manuscript by Sep 30 2024 11:59PM. If you will need more time than this to complete your revisions, please reply to this message or contact the journal office at [plosone@plos.org](mailto:plosone@plos.org). When you're ready to submit your revision, log on to <https://www.editorialmanager.com/pone/> and select the 'Submissions Needing Revision' folder to locate your manuscript file.

Please include the following items when submitting your revised manuscript:

- A rebuttal letter that responds to each point raised by the academic editor and reviewer(s). You should upload this letter as a separate file labeled 'Response to Reviewers'.
- A marked-up copy of your manuscript that highlights changes made to the original version. You should upload this as a separate file labeled 'Revised Manuscript with Track Changes'.
- An unmarked version of your revised paper without tracked changes. You should upload this as a separate file labeled 'Manuscript'.

If you would like to make changes to your financial disclosure, please include your updated statement in your cover letter. Guidelines for resubmitting your figure files are available below the reviewer comments at the end of this letter.

If applicable, we recommend that you deposit your laboratory protocols in [protocols.io](http://protocols.io/) to enhance the reproducibility of your results. Protocols.io assigns your protocol its own identifier (DOI) so that it can be cited independently in the future. For instructions see: [https://journals.plos.org/plosone/s/submission-guidelines#loc-laboratory-protocols](https://c05y1x9s.r.us-east-2.awstrack.me/L0/https:%2F%2Fjournals.plos.org%2Fplosone%2Fs%2Fsubmission-guidelines%23loc-laboratory-protocols/1/010f01915be3f3f1-2fa62929-f00f-496e-8fb9-5886da0bd1d7-000000/Z7AsD_ymST1zURf9tiffWkKlM3s=172). Additionally, PLOS ONE offers an option for publishing peer-reviewed Lab Protocol articles, which describe protocols hosted on [protocols.io](http://protocols.io/). Read more information on sharing protocols at [https://plos.org/protocols?utm_medium=editorial-email&utm_source=authorletters&utm_campaign=protocols](https://c05y1x9s.r.us-east-2.awstrack.me/L0/https:%2F%2Fplos.org%2Fprotocols%3Futm_medium=editorial-email%26utm_source=authorletters%26utm_campaign=protocols/1/010f01915be3f3f1-2fa62929-f00f-496e-8fb9-5886da0bd1d7-000000/MVa7ymvQansohh2NamFQlMD8EYk=172).

We look forward to receiving your revised manuscript.

Kind regards,

Aditya K. Panda, Ph.D.
Academic Editor
PLOS ONE

Journal Requirements:

When submitting your revision, we need you to address these additional requirements.

1. Please ensure that your manuscript meets PLOS ONE's style requirements, including those for file naming. The PLOS ONE style templates can be found at 
<https://journals.plos.org/plosone/s/file?id=wjVg/PLOSOne_formatting_sample_main_body.pdf> and 
<https://journals.plos.org/plosone/s/file?id=ba62/PLOSOne_formatting_sample_title_authors_affiliations.pdf>

Answer: Following the guidelines, the authors have modified the manuscript according to PLOS ONE's style requirements i.e.

Formatting sample title authors affiliation

1. Delete postal codes and street address
2. Correcting format corresponding address
3. Remove Keywords, short running title, abbreviations

Formatting sample main body

4. Rename supplementary files
5. Place supplementary files after Reference section
6. Adding acknowledgements

2. In the online submission form, you indicated that "The data will be available upon request"

All PLOS journals now require all data underlying the findings described in their manuscript to be freely available to other researchers, either 1. In a public repository, 2. Within the manuscript itself, or 3. Uploaded as supplementary information.
This policy applies to all data except where public deposition would breach compliance with the protocol approved by your research ethics board. If your data cannot be made publicly available for ethical or legal reasons (e.g., public availability would compromise patient privacy), please explain your reasons on resubmission and your exemption request will be escalated for approval.

Answer: The authors will upload the data as supplementary information S3 file.

3. PLOS requires an ORCID iD for the corresponding author in Editorial Manager on papers submitted after December 6th, 2016. Please ensure that you have an ORCID iD and that it is validated in Editorial Manager. To do this, go to ‘Update my Information’ (in the upper left-hand corner of the main menu), and click on the Fetch/Validate link next to the ORCID field. This will take you to the ORCID site and allow you to create a new iD or authenticate a pre-existing iD in Editorial Manager. Please see the following video for instructions on linking an ORCID iD to your Editorial Manager account: <https://www.youtube.com/watch?v=_xcclfuvtxQ>

Answer: The corresponding author had provided the ORCID iD.

4. Please include your full ethics statement in the ‘Methods’ section of your manuscript file. In your statement, please include the full name of the IRB or ethics committee who approved or waived your study, as well as whether or not you obtained informed written or verbal consent. If consent was waived for your study, please include this information in your statement as well.

Answer: The author had written sentence” This study was approved (exempted) by the institutional review boards of King’s Mongkut Institute of Technology (EC-KMITL_63_054), Thailand. “

5. Please include a separate caption for each figure in your manuscript.

Answer: The author has added a separate caption for each figure in the manuscript.

6. Please review your reference list to ensure that it is complete and correct. If you have cited papers that have been retracted, please include the rationale for doing so in the manuscript text, or remove these references and replace them with relevant current references. Any changes to the reference list should be mentioned in the rebuttal letter that accompanies your revised manuscript. If you need to cite a retracted article, indicate the article’s retracted status in the References list and also include a citation and full reference for the retraction notice.

Answer: The author has reviewed the reference list and made a change according to reviewer #2 suggested.

[Note: HTML markup is below. Please do not edit.]
Reviewers' comments:

Reviewer's Responses to Questions

**Comments to the Author**

1. Is the manuscript technically sound, and do the data support the conclusions?

The manuscript must describe a technically sound piece of scientific research with data that supports the conclusions. Experiments must have been conducted rigorously, with appropriate controls, replication, and sample sizes. The conclusions must be drawn appropriately based on the data presented.

Reviewer #1: Yes

Reviewer #2: Yes

2. Has the statistical analysis been performed appropriately and rigorously?

Reviewer #1: Yes

Reviewer #2: Yes

3. Have the authors made all data underlying the findings in their manuscript fully available?

The [PLOS Data policy](http://c05y1x9s.r.us-east-2.awstrack.me/L0/http:%2F%2Fwww.plosone.org%2Fstatic%2Fpolicies.action%23sharing/1/010f01915be3f3f1-2fa62929-f00f-496e-8fb9-5886da0bd1d7-000000/Y5z2iDdbDKS23c7KcEIiwH3YvaU=172) requires authors to make all data underlying the findings described in their manuscript fully available without restriction, with rare exception (please refer to the Data Availability Statement in the manuscript PDF file). The data should be provided as part of the manuscript or its supporting information, or deposited to a public repository. For example, in addition to summary statistics, the data points behind means, medians and variance measures should be available. If there are restrictions on publicly sharing data—e.g. participant privacy or use of data from a third party—those must be specified.

Reviewer #1: No

Reviewer #2: Yes

4. Is the manuscript presented in an intelligible fashion and written in standard English?

PLOS ONE does not copyedit accepted manuscripts, so the language in submitted articles must be clear, correct, and unambiguous. Any typographical or grammatical errors should be corrected at revision, so please note any specific errors here.

Reviewer #1: Yes

Reviewer #2: Yes

5. Review Comments to the Author

Please use the space provided to explain your answers to the questions above. You may also include additional comments for the author, including concerns about dual publication, research ethics, or publication ethics. (Please upload your review as an attachment if it exceeds 20,000 characters)

Reviewer #1: Comments to PONE-D-24-13672
Plasmodium falciparum surf4.1 in clinical isolates: from genetic variation and variant diversity to in silico design immunopeptides for vaccine development
The authors amplified and sequenced 76 clinical isolates of the Plasmodium falciparum surf4.1 gene obtained from patients in Thailand. The resulting sequences were analyzed for genetic diversity and epitope prediction. Upon reviewing the study, I found it potentially valuable for readers. Overall, the methodology, data analysis, and interpretation are adequate. However, several issues must be addressed before the manuscript is suitable for publication.

Abstract:
Line 25: "SURFINs family expressed…." should be "SURFINs protein family expressed…". Alternatively, please consider rewriting this sentence.
Answer: Corrected accordingly.

Line 28: "P. falciparum" should be italicized.
Answer: Corrected accordingly.

Lines 75-76: “Furthermore, we have identified four conserved and four dimorphic predicted epitopes as proposed vaccine candidates.” Please consider rewriting this sentence.
Answer: The sentense is in lines 45-46 and corrected accordingly.

Introduction:
Line 50: "Erythrocytic" should be "erythrocytic".
Answer: Corrected accordingly.

Line 53: "P falciparum" should be "P. falciparum".
Answer: Corrected accordingly.

Lines 50-52 and 60-61: "Parasitized red blood cells……." are repetitive.
Answer: Corrected accordingly.

Lines 75-76: "High diversity of variable regions in exon 1 were reported in P. falciparum field isolates," please clarify the origin of these isolates.
Answer: Corrected accordingly.

Lines 80-81: "Still the causal of CNV has not been verified," please rewrite to improve clarity.

Answer: corrected and rewritten.

Materials and Methods:
Line 93: "P. falciparum infection was diagnosed by microscopic…." How did the authors exclude co-infection with other Plasmodium species, given that microscopic examination is not entirely reliable for species identification? Additionally, underlying diseases causing anemia might also play a role. Therefore, the clinical outcomes classified as complicated and uncomplicated malaria might be compromised.
Answer: All samples are mono *P. falciparum* infection, based on microscopic blood examination. As the reviewer mentioned, we cannot exclude the co-infection with other species. However, the main objective of this study is to analyze the genetic diversity and identify immunogenic epitope for the vaccine candidate. We think the coinfection with other malaria species may not confound our main findings. Moreover, the role of mixed spp. infection on the clinical outcome remained unclear. Nonetheless, the underlining conditions of the patients may confound the clinical outcomes, this is a limitation of this study.

Lines 118, 120, 128, 129, and possibly elsewhere throughout the manuscript: "Takarabio," please provide the country of the product manufacturer.
Answer: corrected-the author have provided the country of product manufacturer.

Line 139: "and" should not be italicized.
Answer: Corrected accordingly.

Line 155: ")" should be ".".
Answer: Corrected accordingly.

Line 175: "3bp" should be "3 bp".
Answer: Corrected accordingly.

Line 187: "Standard" should be "standard".
Answer: Corrected accordingly.

Lines 211, 218, and elsewhere: "outside domain," I suggest using "extracellular domain."
Answer: Corrected accordingly.

Results:
Lines 243-244: This sentence may not be necessary.
Table 1: "DNASP analysis nucleotide diversity," consider rewriting this table caption, e.g., "Nucleotide diversity indices of…."
Answer: Corrected accordingly.

Table 2: "Plasmodium falciparum" should be italicized, and "90bp, 3bp" should include a space, e.g., "90 bp, 3 bp".
Answer: Corrected accordingly.

Line 324: "substitution of 247 and 26 and synonymous," please correct.
Answer: Corrected accordingly.

Line 395: "Clade" should be "clade".
Answer: Corrected accordingly.

Line 398: "(" should be deleted.
Answer: corrected- the author have deleted the bracket.

Line 407: "conserved constructed among," please rewrite.
Answer: corrected-the author have re-written the sentence.

Discussion:
Line 465: “infected red cells” should be “infected red blood cells”

Answer: Corrected accordingly.

Reviewer #2: Dear Author
The research work was executed well and described the current frequency of genetic variation in SURFIN 4.1 in Thai clinical isolates. As this protein is an important vaccine candidate, such study is need of the moment. However, few clarifications on below facts will augment the quality of this research work;

1. Line 115 mentioned the PCR thermal profile in method section; it is not clear about the annealing time period, is it for 7-minutes @ 58 degree Celsius?
Answer: Yes. The PCR condition is following the polymerase enzyme manufacturer’s manual instruction.

2. Line 140 mentioned about the concentration of dilutions used for PCR efficiency evaluation; Is the provided concentration of GENOMIC-DNA belongs to culture adapted falciparum or MR4 g-DNA is used here. If it is culture adapted falciparum, then please described how such concentration is achieved or if it is MR4 g-DNA, please acknowledge that.
Answer: Corrected by adding sentences below in the material and method section.

Culture adapted *P. falciparum* 3D7 with 2% parasitemia were used to prepare genomic DNA using QIAamp DNA Blood mini kit following the manufacture’s manual instruction (Hilden, Germany). DNA concentration was measured by Implean NanoPhotometer N60/N50 (Munich, Germany).

3. It is not clear why n=46 was not sequenced for Exon-1 and performed for exon-2 only. The sequencing data of exon-1 could have provided comparable insight to the previous published sequence from Thai-Myanmar areas. Why this study is so focused on exon-2, when exon-1 is more variable, this can be discussed as a limitation of the study.
Answer: The amplified PCR products of full length surf_4.1_ (6757 bp) of 76 samples were subjected for sequencing. We were able to obtain complete full-length (exon1-intron-exon2) in 30 samples and partial sequences in 46 samples. Of those partial sequences, the obtained sequencing results showed a stretch of ambiguous code (NNNNNN) in variable region of exon1 and complete nucleotide sequence of intron and exon 2 (partial exon1-intron-exon2. The sequence of full-length nucleotide sequences (n=30) were used to analyse genetic variation and provided comparable insight to the previous published sequences from Thailand and worldwide. The exon2 provide insight information to identify type of SURFIN_4.1_ variants, truncated or contain different number of tryptophan rich domain. This will affect location of SURFIN_4.1_ protein expression. The TM variants will only expressed on the merozoite surface while WDs variants will expressed on both merozoite and infected red blood cell surface.

To clarify this point , we’ve re-written the sentence to “From 76 clinical isolates, 30 complete full-length and 46 partial nucleotide sequences of *surf_4.1_* gene were obtained in this study.” in results under section: The organization of full-length *surf_4.1_* gene (exon1-intron-exon2) and genetic variation of coding sequence.

4. Line 523-524 and 530-531; cited Reference no 7 is not the correct one for the given statements/facts and can be replaced by reference no.6 the study related to Thai isolates.
Answer: Corrected- reference 7 has been replaced by reference 6

5. Involvement of Thai isolates from other parts of the country for this type of study towards establishment of the SURFIN 4.1 as vaccine candidate for whole country may be discussed.

Answer: The author have added the involvement of Thai isolates in the discussion section.

6. PLOS authors have the option to publish the peer review history of their article ([what does this mean?](https://c05y1x9s.r.us-east-2.awstrack.me/L0/https:%2F%2Fjournals.plos.org%2Fplosone%2Fs%2Feditorial-and-peer-review-process%23loc-peer-review-history/1/010f01915be3f3f1-2fa62929-f00f-496e-8fb9-5886da0bd1d7-000000/BSbYpB0NT99qKB6prkm6tBXxsO4=172)). If published, this will include your full peer review and any attached files.

If you choose “no”, your identity will remain anonymous but your review may still be made public.

**Do you want your identity to be public for this peer review?** For information about this choice, including consent withdrawal, please see our [Privacy Policy](https://c05y1x9s.r.us-east-2.awstrack.me/L0/https:%2F%2Fwww.plos.org%2Fprivacy-policy/1/010f01915be3f3f1-2fa62929-f00f-496e-8fb9-5886da0bd1d7-000000/qDKtT-5Jcc0NmPfvIZWRUxg1UkA=172).

Reviewer #1: **Yes:** Morakot Kaewthamasorn

Reviewer #2: **Yes:** PRASHANT MALLICK

[NOTE: If reviewer comments were submitted as an attachment file, they will be attached to this email and accessible via the submission site. Please log into your account, locate the manuscript record, and check for the action link "View Attachments". If this link does not appear, there are no attachment files.]

While revising your submission, please upload your figure files to the Preflight Analysis and Conversion Engine (PACE) digital diagnostic tool, [https://pacev2.apexcovantage.com/](https://c05y1x9s.r.us-east-2.awstrack.me/L0/https:%2F%2Fpacev2.apexcovantage.com%2F/1/010f01915be3f3f1-2fa62929-f00f-496e-8fb9-5886da0bd1d7-000000/bawgNBMoFWHranMyuPK7pxMvId0=172). PACE helps ensure that figures meet PLOS requirements. To use PACE, you must first register as a user. Registration is free. Then, login and navigate to the UPLOAD tab, where you will find detailed instructions on how to use the tool. If you encounter any issues or have any questions when using PACE, please email PLOS at [figures@plos.org](mailto:figures@plos.org). Please note that Supporting Information files do not need this step.
